# Scalable and selective deuteration of (hetero)arenes

**DOI:** 10.1038/s41557-021-00846-4

**Published:** 2022-01-13

**Authors:** Wu Li, Jabor Rabeah, Florian Bourriquen, Dali Yang, Carsten Kreyenschulte, Nils Rockstroh, Henrik Lund, Stephan Bartling, Annette-Enrica Surkus, Kathrin Junge, Angelika Brückner, Aiwen Lei, Matthias Beller

**Affiliations:** 1 Leibniz-Institut für Katalyse e.V., Rostock, Germany; 2 Institute for Advanced Studies (IAS), Wuhan University, Wuhan, P. R. China

**Keywords:** Homogeneous catalysis, Synthetic chemistry methodology, Synthetic chemistry methodology, Heterogeneous catalysis

## Abstract

Isotope labelling, particularly deuteration, is an important tool for the development of new drugs, specifically for identification and quantification of metabolites. For this purpose, many efficient methodologies have been developed that allow for the small-scale synthesis of selectively deuterated compounds. Due to the development of deuterated compounds as active drug ingredients, there is a growing interest in scalable methods for deuteration. The development of methodologies for large-scale deuterium labelling in industrial settings requires technologies that are reliable, robust and scalable. Here we show that a nanostructured iron catalyst, prepared by combining cellulose with abundant iron salts, permits the selective deuteration of (hetero)arenes including anilines, phenols, indoles and other heterocycles, using inexpensive D_2_O under hydrogen pressure. This methodology represents an easily scalable deuteration (demonstrated by the synthesis of deuterium-containing products on the kilogram scale) and the air- and water-stable catalyst enables efficient labelling in a straightforward manner with high quality control.

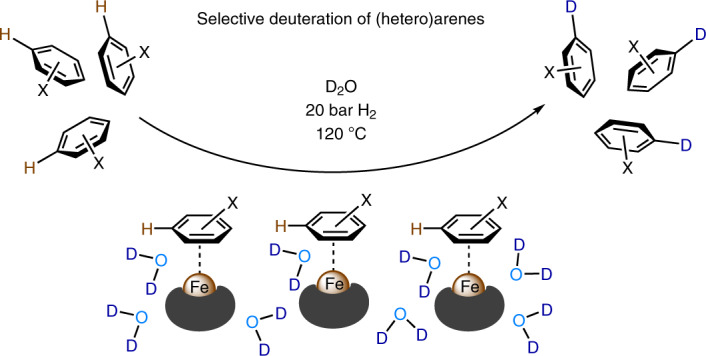

## Abstract (web summary)

A method for the selective deuteration of anilines, indoles, phenols and heterocyclic compounds, including natural products and other bioactive molecules, has been developed. The nanostructured iron catalyst that underpins this process is prepared by combining cellulose with iron salts and has been used for the preparation of deuterated compounds on up to a kilogram scale.

## Main

**Fig. 1:**
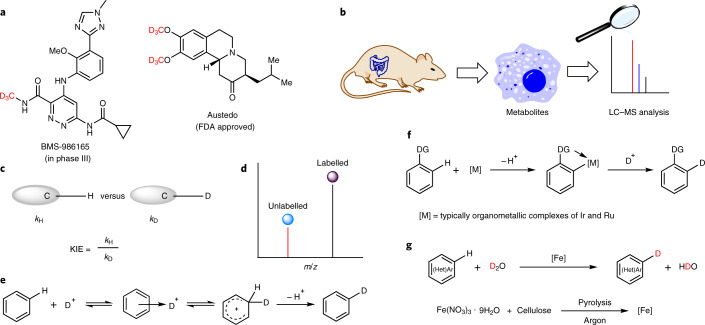
Applications of deuteration and methods for H/D exchange. **a**, Deuterated drug molecules: Austedo has been approved by the FDA and BMS-986165 is under trial. **b**, Identification of specific metabolites using LC–MS: accurate analysis of metabolites based on stable isotope labelling coupled with LC–MS analysis. **c**, KIE investigations based on C–H versus C–D bond cleavage. **d**, Preparation of internal standards for LC–MS analysis. The isotopically labelled internal standards can be used for quantitative determination. **e**, Acid-mediated H/D exchange. **f**, Transition-metal-catalysed H/D exchange. DG, directing group. **g**, This work: nanostructured iron catalyst for H/D exchange of (hetero)arenes using D_2_O.

Isotope labelling methodologies play an essential part in the development of new pharmaceuticals and agrochemicals^1^ (Fig. 1a). For example, in the pharmaceutical industry, isotopes of active drugs are commonly prepared to understand their metabolism and to identify specific metabolites (Fig. 1b). The most common isotopic labels are deuterium atoms, which are also well suited to determine kinetic isotope effects (KIEs) in fundamental mechanistic investigations that result from differences in the rate of C–H versus C–D bond cleavage^2,3^ (Fig. 1c). Deuterium-labelled compounds show virtually identical physical behaviour to that of their hydrogen analogues, whilst differing in molecular mass, and thus are the primary source for the preparation of internal standards for liquid chromatography–mass spectrometry (LC–MS) analysis in the investigation of environmental, animal and human samples^4,5^ (Fig. 1d). Accordingly, deuteration facilitates advancements in metabolomics including metabolite identification and quantification, in toxicogenomic studies for the related reactive metabolites and in proteomics studies.

Due to the potentially improved pharmacokinetic and pharmacological properties of deuterium-labelled compounds, while retaining almost the same chemical structure and physical properties as the unlabelled counterparts, in recent years this class of compounds has gained increasing interest as actual medications^6,7^. Notably, in 2017 the Food and Drug Administration (FDA) cleared the first deuterated drug, Austedo, for the treatment of Huntington’s-disease-related disorders^8^. Meanwhile, several deuterated compounds are in clinical trials for various applications. Based on these developments, there is growing potential and interest in accessing selectively deuterated building blocks on a larger scale. In this context, specific and practical labelling methodologies for arenes/heteroarenes as well as amines, which are found in most small-molecule-based drugs, are of increasing importance. Critical parameters for such applications are the availability and price of the labelling reagent, catalysts and so on, and the feasibility of the process in an industrial setting. Notably, precise control of impurities, degree of deuteration and consistency are also prerequisites for any real-life implementation^9^.

Acid-mediated hydrogen–deuterium exchange reactions (H/D exchange) are among the oldest methods known for labelling of arenes. Nevertheless, they allow selective incorporation of deuterium only for simple substrates following an electrophilic aromatic substitution mechanism (Fig. 1e)^10^. In most of the known protocols, the necessity to use high temperatures and stoichiometric amounts of concentrated strong acids leads to poor functional group tolerances and safety risks, especially on a larger scale^11^.

Based on advances in homogeneous metal-catalysed C–H activation, a variety of organometallic complexes have evolved for catalytic H/D exchange reactions of arenes, as well as aliphatic amines in the α or β positions (Fig. 1f)^12–15^. For example, homogeneous iridium-based Crabtree and Kerr catalysts have been used for C(*sp*^2^)–H hydrogen isotope exchange reactions using D_2_ gas^16,17^. Chirik and co-workers first reported the use of a molecularly defined iron catalyst for the tritiation/deuteration of pharmaceutical drugs at aromatic C–H moieties using D_2_ and T_2_ gas^18^. More recently, the MacMillan group developed a photochemical-catalysed hydrogen-isotope-exchange (HIE) method for the selective labelling of *N*-alkylamine-based drugs^19^.

Apart from photocatalysts and defined organometallic complexes, heterogeneous materials have also been studied in labelling reactions. So far, Pd/C and Pt/C are known to catalyse multi H/D exchange of arenes and heterocyclic amines^20^. In addition, ruthenium- and iridium-based catalysts in the presence of D_2_ have been developed with promising activity^21,22^. Unfortunately, in all these cases the selectivity and the tolerance of easily reducible functional groups and halogens is challenging. Furthermore, besides those based on nickel^23^, all heterogeneous catalysts known for deuterations rely on expensive precious metals, which hamper their use in the agrochemical, pharmaceutical or food industries as those metals must be removed completely from the final products according to regulations. Apart from the catalyst and the reaction conditions, the source of deuterium is critical for the application of such methodologies. This is especially true for the preparation of labelled building blocks on the multi-gram or even kilogram scale. In this respect, cheap, safe and operationally convenient D_2_O is the ideal source for such transformations because it is basically the parent compound for all other deuteration reagents, including D_2_.

In this article we report a unique heterogeneous iron catalyst for general and practical deuteration of arenes and heteroarenes. Using D_2_O in the presence of a biomass-derived iron catalyst under hydrogen pressure allows for the preparation of >90 selectively deuterated building blocks, representative drugs and natural products with high and reliable deuterium incorporation (Fig. 1g).

## Results and discussion

### Reaction development

Initially, we tested standard, commercially available heterogeneous catalysts and tailor-made supported nanoparticles (NPs) for selective deuteration of 4-phenylmorpholine in D_2_O (Supplementary Table 1). This benchmark substrate was chosen because it permits labelling both at the nitrogen-containing heterocycle and the phenyl ring. In all cases, the extent of isotopic exchange was determined using ^1^H NMR spectroscopy. In agreement with previous works^24^, Pd/C led to deuterium incorporation at the α position of the nitrogen atom on the morpholine ring (Supplementary Table 1, entry 1). Recently, we introduced a variety of supported 3*d*-metal NPs for selective hydrogenation and oxidation reactions^25,26^. In this context, iron-based NPs are particularly attractive to us due to the abundance, low cost and negligible safety concerns of iron salts. Much to our surprise, pyrolysis of Fe(NO_3_)_3_·9H_2_O with cellulose resulted in highly active and selective catalytic systems for deuteration of the phenyl ring in the *ortho* and *para* positions, which even outperformed commercial catalysts such as Pt/C, Au/C and Ru/C (Supplementary Table 1, entry 10 versus entries 2 and 4). The deuterium incorporation could be further improved under hydrogen pressure (Supplementary Table 1, entry 12). Furthermore, the stability and recyclability of the catalyst under H_2_ are better (Supplementary Information, section 5). Pseudo in situ X-ray photoelectron spectroscopy (XPS) studies show that the iron oxides formed on the surface of the catalyst during the catalysis could be partially reduced to Fe(0) when heating the sample under H_2_ (Supplementary Fig. 6). Finally, the benchmark reaction performed with D_2_O in the presence of hydrogen at 120 °C gave nearly quantitative deuteration (see Supplementary Table 1 for more details).

### Catalyst characterization

To understand the structure of the most active material (Fe-Cellulose-1000), powder X-ray diffraction, XPS, scanning transmission electron microscopy (STEM) and X-ray absorption spectroscopy (XAS) investigations were performed. These results show that the freshly pyrolysed catalyst consists of Fe/Fe_3_C particles 20–50 nm in size, covered by a shell of up to 30 graphene layers with a overall thickness of 6–10 nm. Embedding the particles in the carbon matrix prevents them from undesired aggregation. During the early stages of the catalytic reaction, the graphene cover is partly removed, thus enabling the contact of the iron surface with reactants. Then, during the reaction, Fe_3_C is partly converted to metallic iron which is considered as active phase in the reaction (for details see Supplementary Information, section 6).

### Mechanistic studies

**Fig. 2:**
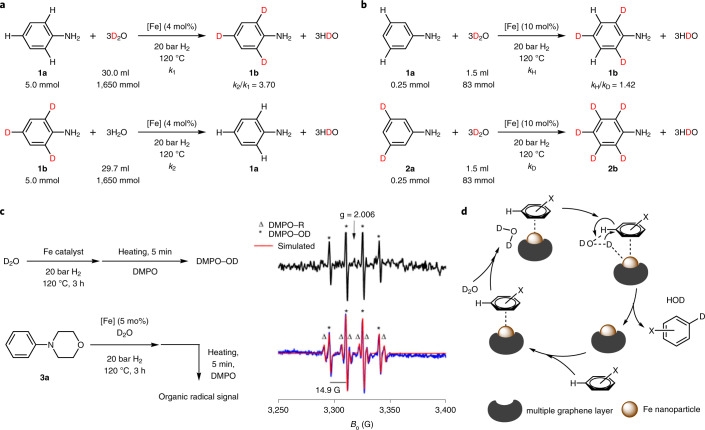
Mechanistic studies and proposed mechanism. **a**, Aniline in D_2_O and aniline-2,4,6-*d*_3_ in H_2_O were used for the KIE studies. The reaction in H_2_O is roughly four times faster than the reaction in D_2_O. **b**, Deuteration of aniline and 3,5-dideuterioaniline in D_2_O were performed for KIE studies, and a minor secondary KIE was observed. **c**, EPR studies: ^•^OD radical trapping using DMPO and detection of the DMPO–R spin adduct. This indicates that formation of radical intermediates is a consequence of homolytic D–OD scission initiated by the iron catalyst. **d**, Proposed mechanism: the multiple graphene layer is removed during the early stages of the catalytic reaction, thus enabling contact of the iron surface with D_2_O and reactants. The key step of the reaction is D_2_O splitting by the iron catalyst. The resulting radicals are not liberated into the solution but remain adsorbed on the catalyst surface.

Next, investigations of KIE and electron paramagnetic resonance (EPR) studies were performed to obtain a better mechanistic understanding (Supplementary Information, section 7). According to comparison experiments of the model substrate, the reaction is roughly four times faster in H_2_O than in D_2_O. Apparently, the cleavage of the D–OD bond is the rate-limiting step in this process (Fig. 2a and Supplementary Fig. 13). Interestingly, by comparing the deuteration of aniline and 3,5-dideuterioaniline, a minor secondary KIE is also observed (Fig. 2b and Supplementary Fig. 14). This kinetically relevant result may be explained by the slightly different coordination of the deuterated and non-deuterated substrate to active catalyst centres on the surface. To check whether radicals are formed upon cleavage of the D–OD bond, EPR measurements with the spin trap 5,5-dimethyl-1-pyrroline*-N*-oxide (DMPO) have been performed (Supplementary Information, section 7.2). In a blind experiment, the catalyst was heated in D_2_O before DMPO was added, and an EPR spectrum was recorded immediately after quenching the reaction to room temperature. This spectrum showed only a weak but characteristic hyperfine structure quartet signal of DMPO–OD spin adducts^27^ which results from trapping ^•^OD radicals by DMPO (Fig. 2c black line). This shows that the iron catalyst can promote to a small extent homolytic splitting of D_2_O. However, in the presence of 4-phenylmorpholine, an additional hyperfine structure sextet characteristic of a DMPO–R spin adduct^28^ was detected (Fig. 2c, blue line), suggesting that *OD subtract hydrogen atoms from the substrate, forming HOD, leaving behind ^•^R radicals. Interestingly, no ^•^R radicals were detected when the iron catalyst and the 4-phenylmorpholine substrate were heated in toluene, which indicates that formation of radical intermediates is a consequence of homolytic D–OD scission initiated by the iron catalyst. Considering the well-known fact that ^•^OD radicals are very reactive and unselective, the observed high selectivity is surprising.

### Proposed mechanism

We propose a concerted mechanism in which D_2_O is split homogeneously by the iron catalyst, yet the resulting radicals are not liberated into the solution but remain adsorbed on the catalyst surface as activated D* and *OD species. *OD abstracts a hydrogen atom from the phenyl ring, forming HDO and the corresponding phenyl* species^29,30^, which subsequently produces D–R (Fig. 2c,d). The observed high *ortho*/*para* selectivity may be a result of electron density transfer from the electron-rich metallic iron particle to the adsorbed aromatic ring because this is well known to promote electrophilic *ortho*/*para* substitutions.

### Synthetic scope

#### Building blocks

Having established the optimized conditions, we then evaluated the substrate scope of the system and its tolerance towards functional groups. Because anilines are used for the synthesis of diverse building blocks for agrochemicals and pharmaceuticals, we started to explore the deuteration of functionalized anilines. Indeed, Fe-Cellulose-1000 permitted smooth deuteration of 35 different anilines with excellent chemo- and regioselectivity (Table 1, **3–**37b).

**Table 1:**
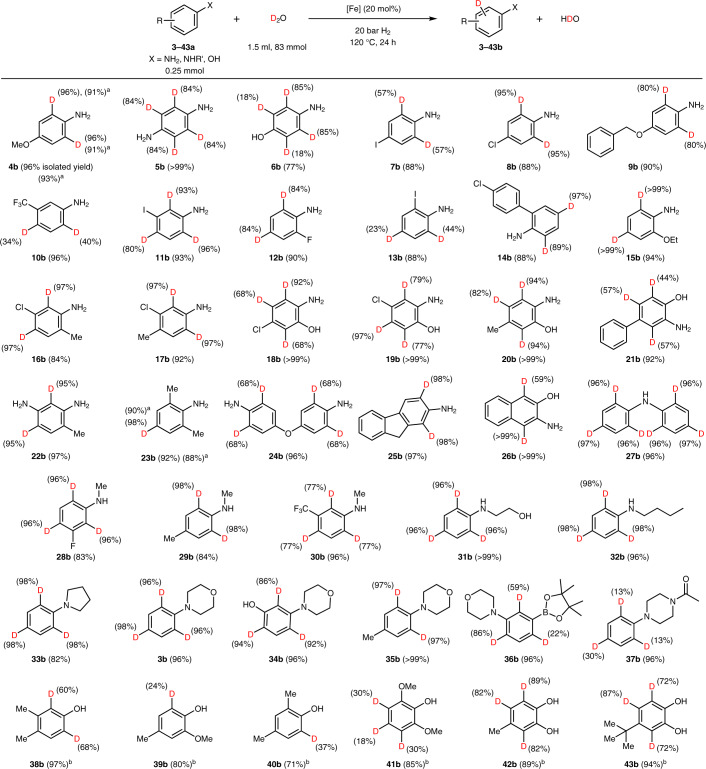
Nanostructured iron catalyst for deuteration of anilines and phenols

Deuterium content determined by quantitative ^1^H NMR. ^a^The reaction was performed on the gram scale. ^b^The reaction time was 72 h.

In general, the transformations can be easily run on the gram-scale (4b and 23b). Diverse halogen-containing (for example, chlorine and iodine) anilines afforded the deuterated products (7b, 8b, **11–**14b and **16–**19b) without notable dehalogenation side-reactions, which is a common problem of precious-metal-based catalysts. We demonstrated the isotopic purity of chloro-, bromo- and iodo-containing compounds by LC–MS (Supplementary Information, section 8.3). Furthermore, deuteration of some phenols in the presence of the iron catalyst showed deuterium incorporation after an extended time (Table 1, **38–**43b). Like the model system, negligible or no deuterium incorporation is observed for several representative substrates (**3a**, **4a**, **38a**, **44a**, **52a** and **67a**) in the absence of the iron catalyst (Supplementary Fig. 16).

Next, deuteration of different nitrogen-containing heteroarenes such as pyridines, tetrahydroquinolines, phenothiazines, phenoxazines, indoles, indolines and quinolines, and even those bearing two nitrogen atoms, were investigated. Such heterocycles are representative structural components of modern pharmaceuticals: 59% of FDA-approved drugs contain a nitrogen heterocyclic motif^31^. As shown in Table 2, catalytic labelling provided the corresponding products (**44–**66b). Notably, for anilines and phenols the regioselectivity of the labelling reaction is in accordance with an electrophilic aromatic substitution mechanism. However, using pyridines, indoles or indolines, different reactivity patterns are observed, which hints towards a different process.

**Table 2:**
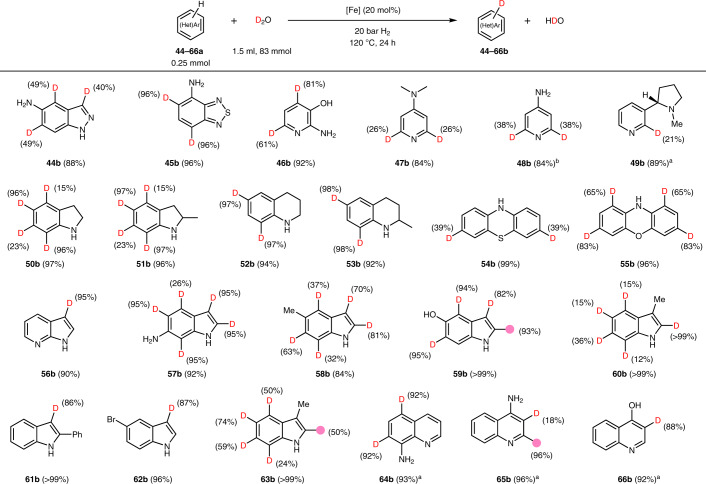
Nanostructured iron catalyst for deuteration of heteroarenes

Deuterium content determined by quantitative ^1^H NMR. ^a^The reaction was performed at 140 °C for 24 h. The pink circles and numbers denote the positions of the C–H bonds that are labelled and the percentage incorporation of the hydrogen isotope, respectively.

#### Natural products and pharmaceuticals

To further showcase the utility of iron-catalysed H/D exchange reactions, late-stage deuteration of representative drugs and natural products was investigated (Table 3). For example, melatonin is converted into the deuterated 67b with high levels of deuterium incorporation. Similarly, *N*-acetylserotonin is deuterated to give 68b. Purine-containing compounds, such as nucleoside analogues kinetin, inosine, pentoxifylline and the nucleobase adenine, are labelled to the products **69**–71b and 73b. Alkaloids, for example, brucine and strychnine, are deuterated at both aromatic rings and in the heterocyclic ring in the α position to the nitrogen (74b and 75b). In the case of nicotinic acid, labelling occurred with relatively lower deuterium incorporation selectively to give 76b. Furthermore, natural phenol derivatives, for example, tyrosol, resveratrol, thymol, arbutin and piceid, were evaluated (**77–**81b**)**. Tetrahydroquinoline alkaloids, for example, augustureine and galipinine, also proceeded with deuterium labelling (82b and 83b). Aniline derivatives such as dropropizine, dl-aminoglutethimide and nimodipine-NH_2_ were deuterated with good selectivity (**84**–86b). Notably, medications such as carvacrol, estradiol and *O*-desmethylvenlafaxine gave the deuterium analogues **87**–89b. As an example, for selective deuteration of an aromatic amino acid, l-tryptophan was employed to directly give 90b. In all cases shown above the standard catalytic protocol was used and no further optimization was performed. However, it should be noted that the deuterium incorporation can be substantially improved at higher temperature (84b and 90b).

**Table 3:**
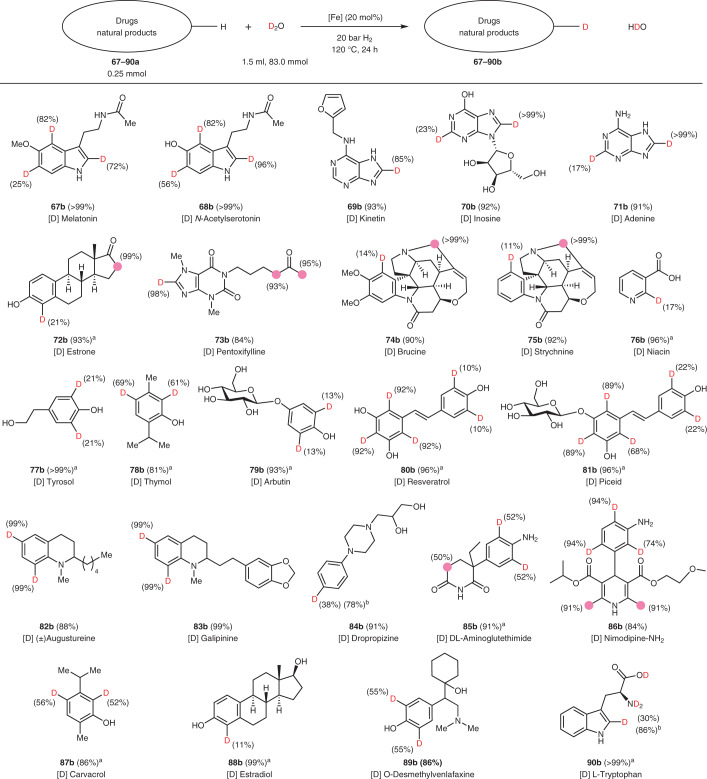
Nanostructured iron catalyst for deuteration of natural products and pharmaceuticals

Representative drugs, hormones, nucleobases, steroids, alkaloids, amino acids, upscale and applications. Deuterium content determined by quantitative ^1^H NMR. ^a^The reaction time was 72 h. ^b^The reaction was performed at 140 °C for 24 h. The pink circles and numbers denote the positions of the C–H bonds that are labelled and the percentage incorporation of the hydrogen isotope, respectively.

**Fig. 3:**
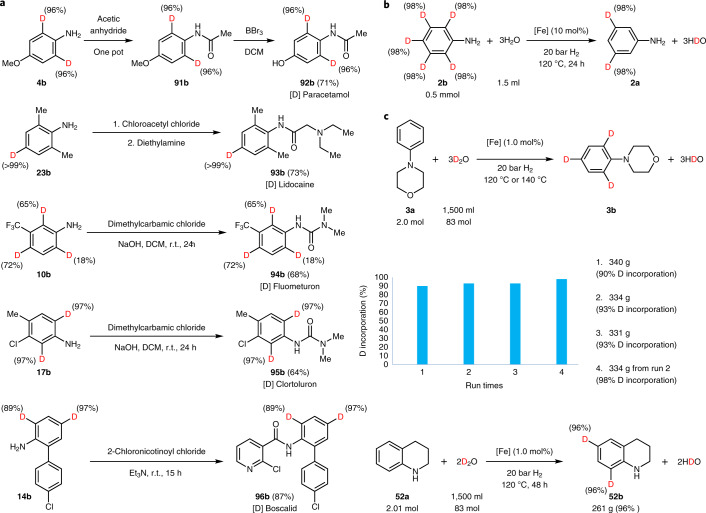
Synthetic applications and large-scale synthesis. **a**, Preparation of deuterated *N*-acylated anilines. Deuterated drugs, herbicides and fungicide were prepared from the corresponding deuterated anilines. **b**, Dedeuteration of perdeuterated aniline using H_2_O to 3,5-dideuterioaniline. **c**, Scale-up reactions: reuse of the iron catalyst on the 2.0 mol scale. Overall, 1.005 kg 4-phenylmorpholine (6.17 mol) was deuterated using the same catalyst batch; >95% deuterium-labelled 4-phenylmorpholine and 1,2,3,4-tetrahydroquinoline were produced.

Following standard conditions, deuteration of *N*-acylated anilines is more difficult; however, such labelled products can be conveniently prepared from the corresponding deuterated anilines (Fig. 3a). For instance, 2,6-D*-*labelled paracetamol 92b was readily synthesized with excellent deuterium incorporation. Deuterated lidocaine 93b, herbicides fluometuron 94b and chlortoluron 95b as well as fungicide boscalid 96b were obtained from the corresponding deuterated anilines.

All these cases demonstrate that the presented methodology works well with amino- and/or hydroxyl-substituted arenes as well as heteroarenes; however, less electron-rich benzenes, for example, 1-bromo-, 1-chloro- and 1-fluoro-4-methoxybenzene and 1-methyl-4-(trifluoromethyl)benzene, showed no notable deuterium incorporation under standard conditions. In case of successful labelling as described above deuteration occurred at the most electron-rich positions. Obviously, this limits the possibilities to obtain deuterated compounds at other positions. For example, 2,4,6-trideuterated anilines are conveniently available, while selective deuteration at the 3- and 5-positions are apparently not possible. However, we envisioned a convenient strategy to overcome this limitation: non-selective deuteration of anilines is known in the presence of Pt/C (ref. ^32^). Subsequent selective D/H exchange using our Fe-Cellulose-1000 catalyst in water makes it possible to selectively obtain deuterium isomers at positions that are not prone to electrophilic substitution reactions. Thus, 3,5-dideuterioaniline **2a** is obtained from aniline via the perdeuterated derivative (Fig. 3b).

Having established complementary approaches for selective deuteration of a variety of substrates, we then evaluated some practical aspects of our catalyst system. It should be noted that most of the reactions shown above have been performed on a scale typically used in drug discovery. However, as shown in Fig. 3c it is possible to conveniently run such labelling reactions on the 20 to >300 g scale (Supplementary Information, section 9), with the only limitation being the size of the commercial autoclave. In general, stability, reusability and avoidance of metal contamination are intrinsic advantages to the use of any heterogeneous catalyst. To demonstrate these benefits, the iron catalyst was recycled up to five times for the benchmark reaction. As depicted in Supplementary Fig. 18, no substantial loss of activity was observed. During deuteration reactions, an oxidic structure composed of Fe^2+^ and Fe^3+^ was formed as proven by XPS (Supplementary Fig. 5). With respect to metal contamination of the product, inductively coupled plasma optical emission spectrometry measurements of the D_2_O solution detected no iron leaching in any of these runs (Supplementary Table 8). Notably, the recycled catalyst system can also be used for different substrates, which is important in the context of multipurpose batch reactors, which dominate in the pharmaceutical industry. Thus, labelled products with high deuterium incorporation were produced on >1 kg scale from the same catalyst batch (Fig. 3c and Supplementary Information, section 9).

In summary, we have developed a general methodology for heterogeneous iron-catalysed deuteration reactions. We believe this protocol paves the way for practical labelling processes and the large-scale synthesis of specific deuterated building blocks. Although the quality of different deuteration reactions was performed as accurately as possible (Supplementary Tables 5 and 6), all presented developments took place under conditions that did not conform to good manufacturing practice. The optimal biomass-derived catalyst allows for an activation and utilization of low-cost D_2_O. The performance of this catalyst system is improved in the presence of hydrogen, which leads to in situ reduction of iron oxides on the surface as indicated by pseudo in situ XPS measurements (Supplementary Fig. 6). The presented system is effective for the selective deuteration of anilines, indoles, phenols and other heterocyclic compounds, including late-stage labelling of natural products and bioactive molecules, and can be readily used for the preparation of deuterated compounds on the kilogram scale. By using complementary approaches, different positional deuterated products can also be obtained in a practical manner.

## Methods

### Determination of deuterium incorporation

The positions and percentage of deuterium incorporation were determined by ^1^H NMR. The equation below was used to determine the degree of deuterium incorporation; peaks were calibrated against a signal corresponding to a unlabelled position. The labelling position was determined by ^1^H NMR according to the chemical shifts and peak multiplicity. In addition, deuterium incorporations were confirmed using high-resolution MS by comparison of all the labelled and unlabelled compounds (note that high-resolution MS serves here to substantiate the results of quantitative NMR analysis).

$$\%\, {{{\mathrm{deuteration}}}} = 100 - \left[ {\left( {\frac{{{{{\mathrm{residual}}}}\,{{{\mathrm{integral}}}}}}{{{{{\mathrm{number}}\,{\mathrm{of}}}}\,{{{\mathrm{labelling}}}}\,{{{\mathrm{sites}}}}}} \times 100} \right)} \right]$$

### Procedure for the preparation of the catalyst

A 250 ml oven-dried single-necked round-bottomed flask equipped with a reflux condenser and a Teflon-coated, egg-shaped magnetic stir bar (40 mm × 18 mm) was charged with Fe(NO_3_)_3_·9H_2_O (404 mg, 1.0 mmol) dissolved in ethanol (150 ml). Then, this solution was heated to 80 °C (r.t. to 80 °C) in an oil bath and stirred for 1 h. To the reaction solution, 6.0 g cellulose was added via a glass funnel, and the resulting heterogeneous mixture was stirred at 450 r.p.m. for 15 h at 80 °C. The flask was taken out from the bath and cooled to ambient temperature. The solvent was removed in vacuum and then dried under an oil pump vacuum for 4 h to give a yellow solid. The sample was transferred to a ceramic crucible and placed in an oven. The latter was evacuated to ∼5 mbar and then flushed with argon three times. The furnace was heated to 1,000 °C at a rate of 25 °C min^−1^ and held at 1,000 °C for 2 h under argon atmosphere. After the heating was switched off, the oven was allowed to reach r.t., giving the Fe-Cellulose-1000 catalyst as a black powder (note that throughout the process, argon was constantly passed through the oven).

### General procedure for the deuteration reactions

In a 4 ml vial fitted with a magnetic stir bar and septum cap, iron catalyst (60 mg, 20 mol%) and substrate (0.25 mmol) were added. Then, a needle was inserted in the septum, allowing gaseous reagents to enter. After adding the solvent deuterium oxide (1.5 ml), the vials (up to eight) were set in an alloy plate and then placed into a 300 ml steel Parr autoclave. The autoclave was flushed with hydrogen six times at 10 bar and finally pressurized to the desired value (20 bar). Then, it was placed into an aluminium block and heated to the desired temperature. At the end of the reaction, the autoclave was quickly cooled down to r.t. with an ice bath and vented. Finally, the samples were removed from the autoclave, and ethyl acetate was added to the crude mixture. This mixture was centrifuged, and the organic layer was removed from the vials (three times). After removal of all volatiles in vacuo, the desired products were obtained. In case of anilines with deuterium labelling on the nitrogen, 1 ml H_2_O was added during work up and N–D was replaced by N–H.

### General procedure for the >300 g scale reactions

To a 2 l steel Parr autoclave containing 28.0 g Fe-Cellulose-1000 catalyst (1 mol%), 340 g 4-phenylmorpholine (2.09 mol) and deuterium oxide (1,500 ml, 83 mol) were added. The autoclave was flushed with hydrogen six times at 10 bar and finally pressurized to the desired value (20 bar). Then, it was placed into an equipment (see Supplementary Section 9 for more details) for the autoclave and heated to 120 °C, and then held at 120 °C for 120 h. At the end of the reaction, the autoclave was quickly cooled down to r.t. with an ice bath and vented. Finally, the crude reaction was added to ethyl acetate (1.5 l). The reaction mixture was filtered with filter paper. The D_2_O layer was removed from the mixture and washed with ethyl acetate (1.5 l, three times). After removal of all volatiles in vacuo, 341 g of deuterium product was obtained. It is important to note that during the cool down, trace amounts of gas can stay in the solid phase (product and catalyst). Thus, the solid phase should be dissolved slowly in ethyl acetate.

## Online content

Any methods, additional references, Nature Research reporting summaries, source data, extended data, supplementary information, acknowledgements, peer review information; details of author contributions and competing interests; and statements of data and code availability are available at 10.1038/s41557-021-00846-4.

### Supplementary information

Supplementary Information General remarks, procedure details for the preparation of the catalyst, deuteration reactions, reaction development and optimization, catalyst characterization, mechanistic studies, control experiments, scale-up reactions, catalyst recycling and characterization data of the substrates and products, Supplementary Figs. 1–18 and Tables 1–8.

## Data Availability

All data generated or analysed in this study are available in this article and its Supplementary Information.

## benzen-2,4,6-*d*_3_-amine

PubChemID: 445468662
InChIKey: PAYRUJLWNCNPSJ-NHPOFCFZSA-N
MDL Molfile
Chemdraw file

## benzen-*d*_5_-amine

PubChemID: 445468663
InChIKey: PAYRUJLWNCNPSJ-RALIUCGRSA-N
MDL Molfile
Chemdraw file

## 4-(phenyl-2,4,6-*d*_3_)morpholine

PubChemID: 445468664
InChIKey: FHQRDEDZJIFJAL-NHPOFCFZSA-N
MDL Molfile
Chemdraw file

## 4-methoxybenzen-2,6-*d*_2_-amine

PubChemID: 445468665
InChIKey: BHAAPTBBJKJZER-PBNXXWCMSA-N
MDL Molfile
Chemdraw file

## benzene-*d*_4_-1,4-diamine

PubChemID: 445468666
InChIKey: CBCKQZAAMUWICA-RHQRLBAQSA-N
MDL Molfile
Chemdraw file

## 4-aminophen-2,3,5,6-*d*_4_-ol

PubChemID: 445468667
InChIKey: PLIKAWJENQZMHA-RHQRLBAQSA-N
MDL Molfile
Chemdraw file

## 4-iodobenzen-2,6-*d*_2_-amine

PubChemID: 445468668
InChIKey: VLVCDUSVTXIWGW-NMQOAUCRSA-N
MDL Molfile
Chemdraw file

## 4-chlorobenzen-2,6-*d*_2_-amine

PubChemID: 445468669
InChIKey: QSNSCYSYFYORTR-NMQOAUCRSA-N
MDL Molfile
Chemdraw file

## 4-(benzyloxy)benzen-2,6-*d*_2_-amine

PubChemID: 445468670
InChIKey: FIIDVVUUWRJXLF-QFIQSOQBSA-N
MDL Molfile
Chemdraw file

## 3-(trifluoromethyl)benzen-4,6-*d*_2_-amine

PubChemID: 445468671
InChIKey: VIUDTWATMPPKEL-PBNXXWCMSA-N
MDL Molfile
Chemdraw file

## 3-iodobenzen-2,4,6-*d*_3_-amine

PubChemID: 445468672
InChIKey: FFCSRWGYGMRBGD-NRUYWUNFSA-N
MDL Molfile
Chemdraw file

## 2-fluorobenzen-4,6-*d*_2_-amine

PubChemID: 445468673
InChIKey: FTZQXOJYPFINKJ-DRSKVUCWSA-N
MDL Molfile
Chemdraw file

## 2-iodobenzen-4,6-*d*_2_-amine

PubChemID: 445468674
InChIKey: UBPDKIDWEADHPP-DRSKVUCWSA-N
MDL Molfile
Chemdraw file

## 4'-chloro-[1,1'-biphenyl]-3,5-*d*_2_-2-amine

PubChemID: 445468675
InChIKey: JPBWZIPCMDZOPM-DRSKVUCWSA-N
MDL Molfile
Chemdraw file

## 2-ethoxybenzen-4,6-*d*_2_-amine

PubChemID: 445468676
InChIKey: ULHFFAFDSSHFDA-KFRNQKGQSA-N
MDL Molfile
Chemdraw file

## 3-chloro-2,6-dimethylbenzen-4-*d*-amine

PubChemID: 445468677
InChIKey: FGMXFOTYCHZCLA-QYKNYGDISA-N
MDL Molfile
Chemdraw file

## 3-chloro-4-methylbenzen-2,6-*d*_2_-amine

PubChemID: 445468678
InChIKey: RQKFYFNZSHWXAW-NMQOAUCRSA-N
MDL Molfile
Chemdraw file

## 2-amino-5-chlorophen-3,4,6-*d*_3_-ol

PubChemID: 445468679
InChIKey: FZCQMIRJCGWWCL-CBYSEHNBSA-N
MDL Molfile
Chemdraw file

## 2-amino-4-chlorophen-3,5,6-*d*_3_-ol

PubChemID: 445468680
InChIKey: SWFNPENEBHAHEB-CBYSEHNBSA-N
MDL Molfile
Chemdraw file

## 2-amino-5-methylphen-3,4,6-*d*_3_-ol

PubChemID: 445468681
InChIKey: HCPJEHJGFKWRFM-NRUYWUNFSA-N
MDL Molfile
Chemdraw file

## 3-amino-[1,1'-biphenyl]-2,5,6-*d*_3_-4-ol

PubChemID: 445468682
InChIKey: IGIDZGNPFWGICD-AYBVGXBASA-N
MDL Molfile
Chemdraw file

## 4-methylbenzene-2,6-*d*_2_-1,3-diamine

PubChemID: 445468683
InChIKey: VOZKAJLKRJDJLL-NMQOAUCRSA-N
MDL Molfile
Chemdraw file

## 2,6-dimethylbenzen-4-*d*-amine

PubChemID: 445468684
InChIKey: UFFBMTHBGFGIHF-WFVSFCRTSA-N
MDL Molfile
Chemdraw file

## 4,4'-oxybis(benzen-2,6-*d*_2_-amine)

PubChemID: 445468685
InChIKey: HLBLWEWZXPIGSM-RHQRLBAQSA-N
MDL Molfile
Chemdraw file

## 9*H*-fluoren-1,3-*d*_2_-2-amine

PubChemID: 445468686
InChIKey: CFRFHWQYWJMEJN-VBCVTXNDSA-N
MDL Molfile
Chemdraw file

## 3-aminonaphthalen-1,4-*d*_2_-2-ol

PubChemID: 445468687
InChIKey: ZHVPTERSBUMMHK-KCZCTXNHSA-N
MDL Molfile
Chemdraw file

## bis(phenyl-2,4,6-*d*_3_)amine

PubChemID: 445468688
InChIKey: DMBHHRLKUKUOEG-MWJWXFQUSA-N
MDL Molfile
Chemdraw file

## 3-fluoro-*N*-methylbenzen-2,4,6-*d*_3_-amine

PubChemID: 445468689
InChIKey: FHYDHJXZZQCXOX-QGZYMEECSA-N
MDL Molfile
Chemdraw file

## *N*,4-dimethylbenzen-2,6-*d*_2_-amine

PubChemID: 445468690
InChIKey: QCIFLGSATTWUQJ-KCZCTXNHSA-N
MDL Molfile
Chemdraw file

## *N*-methyl-3-(trifluoromethyl)benzen-2,4,6-*d*_3_-amine

PubChemID: 445468691
InChIKey: SRTKIHVQZYXHHJ-QGZYMEECSA-N
MDL Molfile
Chemdraw file

## 2-((phenyl-2,4,6-*d*_3_)amino)ethan-1-ol

PubChemID: 445468692
InChIKey: MWGATWIBSKHFMR-NHPOFCFZSA-N
MDL Molfile
Chemdraw file

## *N*-butylbenzen-2,4,6-*d*_3_-amine

PubChemID: 445468693
InChIKey: VSHTWPWTCXQLQN-WUNMZEPUSA-N
MDL Molfile
Chemdraw file

## 1-(phenyl-2,4,6-*d*_3_)pyrrolidine

PubChemID: 445468694
InChIKey: VDQQJMHXZCMNMU-HVRNSXJSSA-N
MDL Molfile
Chemdraw file

## 3-morpholinophen-2,4,6-*d*_3_-ol

PubChemID: 445468695
InChIKey: BMGSGGYIUOQZBZ-HRYXNQDDSA-N
MDL Molfile
Chemdraw file

## 4-(4-methylphenyl-2,6-*d*_2_)morpholine

PubChemID: 445468696
InChIKey: OLAFVASCPJETBP-KFRNQKGQSA-N
MDL Molfile
Chemdraw file

## 4-(3-(4,4,5,5-tetramethyl-1,3,2-dioxaborolan-2-yl)phenyl-2,4,6-*d*_3_)morpholine

PubChemID: 445468697
InChIKey: NCJDKFFODGZRRL-DHMPXPLHSA-N
MDL Molfile
Chemdraw file

## 1-(4-(phenyl-2,4,6-*d*_3_)piperazin-1-yl)ethan-1-one

PubChemID: 445468698
InChIKey: YFBOBXSXWBMZCY-UJESMPABSA-N
MDL Molfile
Chemdraw file

## 3,4-dimethylphen-2,6-*d*_2_-ol

PubChemID: 445468699
InChIKey: YCOXTKKNXUZSKD-KFRNQKGQSA-N
MDL Molfile
Chemdraw file

## 2-methoxy-4-methylphen-6-*d*-ol

PubChemID: 445468700
InChIKey: PETRWTHZSKVLRE-QYKNYGDISA-N
MDL Molfile
Chemdraw file

## 2,4-dimethylphen-6-*d*-ol

PubChemID: 445468701
InChIKey: KUFFULVDNCHOFZ-QYKNYGDISA-N
MDL Molfile
Chemdraw file

## 2,6-dimethoxyphen-3,4,5-*d*_3_-ol

PubChemID: 445468702
InChIKey: KLIDCXVFHGNTTM-QGZYMEECSA-N
MDL Molfile
Chemdraw file

## 4-methylbenzene-3,5,6-*d*_3_-1,2-diol

PubChemID: 445468703
InChIKey: ZBCATMYQYDCTIZ-NRUYWUNFSA-N
MDL Molfile
Chemdraw file

## 4-(*tert*-butyl)benzene-3,5,6-*d*_3_-1,2-diol

PubChemID: 445468704
InChIKey: XESZUVZBAMCAEJ-WVALGTIDSA-N
MDL Molfile
Chemdraw file

## 1*H*-indazol-3,4,6-*d*_3_-5-amine

PubChemID: 445468705
InChIKey: XBTOSRUBOXQWBO-IWDQAABOSA-N
MDL Molfile
Chemdraw file

## benzo[*c*][1,2,5]thiadiazol-5,7-*d*_2_-4-amine

PubChemID: 445468706
InChIKey: DRLGIZIAMHIQHL-PBNXXWCMSA-N
MDL Molfile
Chemdraw file

## 2-aminopyridin-4,6-*d*_2_-3-ol

PubChemID: 445468707
InChIKey: BMTSZVZQNMNPCT-PBNXXWCMSA-N
MDL Molfile
Chemdraw file

## *N*,*N*-dimethylpyridin-4-amine-2,6-*d*_2_

PubChemID: 445468708
InChIKey: VHYFNPMBLIVWCW-KCZCTXNHSA-N
MDL Molfile
Chemdraw file

## pyridin-2,6-*d*_2_-4-amine

PubChemID: 445468709
InChIKey: NUKYPUAOHBNCPY-NMQOAUCRSA-N
MDL Molfile
Chemdraw file

## (*S*)-3-(1-methylpyrrolidin-2-yl)pyridine-2-*d*

PubChemID: 445468710
InChIKey: SNICXCGAKADSCV-QNICBWFLSA-N
MDL Molfile
Chemdraw file

## indoline-4,5,6,7-*d*_4_

PubChemID: 445468711
InChIKey: LPAGFVYQRIESJQ-RHQRLBAQSA-N
MDL Molfile
Chemdraw file

## 2-methylindoline-4,5,6,7-*d*_4_

PubChemID: 445468712
InChIKey: QRWRJDVVXAXGBT-QFFDRWTDSA-N
MDL Molfile
Chemdraw file

## 1,2,3,4-tetrahydroquinoline-6,8-*d*_2_

PubChemID: 445468713
InChIKey: LBUJPTNKIBCYBY-UPXKZQJYSA-N
MDL Molfile
Chemdraw file

## 2-methyl-1,2,3,4-tetrahydroquinoline-6,8-*d*_2_

PubChemID: 445468714
InChIKey: JZICUKPOZUKZLL-GSUVGXDCSA-N
MDL Molfile
Chemdraw file

## 10*H*-phenothiazine-3,7-*d*_2_

PubChemID: 445468715
InChIKey: WJFKNYWRSNBZNX-NMQOAUCRSA-N
MDL Molfile
Chemdraw file

## 10*H*-phenoxazine-1,3,7,9-*d*_4_

PubChemID: 445468716
InChIKey: TZMSYXZUNZXBOL-LNFUJOGGSA-N
MDL Molfile
Chemdraw file

## 1*H*-pyrrolo[2,3-*b*]pyridine-3-*d*

PubChemID: 445468717
InChIKey: MVXVYAKCVDQRLW-WFVSFCRTSA-N
MDL Molfile
Chemdraw file

## 1*H*-indol-2,3,4,5,7-*d*_5_-6-amine

PubChemID: 445468718
InChIKey: MIMYTSWNVBMNRH-RALIUCGRSA-N
MDL Molfile
Chemdraw file

## 5-methyl-1*H*-indole-2,3,4,6,7-*d*_5_

PubChemID: 445468719
InChIKey: YPKBCLZFIYBSHK-VIQYUKPQSA-N
MDL Molfile
Chemdraw file

## 2-methyl-1*H*-indol-3,4,6-*d*_3_-5-ol

PubChemID: 445468720
InChIKey: MDWJZBVEVLTXDE-KXXDXSMKSA-N
MDL Molfile
Chemdraw file

## 3-methyl-1*H*-indole-2,4,5,6,7-*d*_5_

PubChemID: 445468721
InChIKey: ZFRKQXVRDFCRJG-VIQYUKPQSA-N
MDL Molfile
Chemdraw file

## 2-phenyl-1*H*-indole-3-*d*

PubChemID: 445468722
InChIKey: KLLLJCACIRKBDT-MMIHMFRQSA-N
MDL Molfile
Chemdraw file

## 5-bromo-1*H*-indole-3-*d*

PubChemID: 445468723
InChIKey: VXWVFZFZYXOBTA-WFVSFCRTSA-N
MDL Molfile
Chemdraw file

## 2,3-dimethyl-1*H*-indole-4,5,6,7-*d*_4_

PubChemID: 445468724
InChIKey: PYFVEIDRTLBMHG-LNFUJOGGSA-N
MDL Molfile
Chemdraw file

## quinolin-5,7-*d*_2_-8-amine

PubChemID: 445468725
InChIKey: WREVVZMUNPAPOV-XBTCESIDSA-N
MDL Molfile
Chemdraw file

## 2-methylquinolin-3-*d*-4-amine

PubChemID: 445468726
InChIKey: COCFIBRMFPWUDW-RAMDWTOOSA-N
MDL Molfile
Chemdraw file

## quinolin-3-*d*-4-ol

PubChemID: 445468727
InChIKey: PMZDQRJGMBOQBF-UICOGKGYSA-N
MDL Molfile
Chemdraw file

## [D] Melatonin

PubChemID: 445468728
InChIKey: DRLFMBDRBRZALE-LESCGEGLSA-N
MDL Molfile
Chemdraw file

## [D] *N*-Acetylserotonin

PubChemID: 445468729
InChIKey: MVAWJSIDNICKHF-COJKPNSOSA-N
MDL Molfile
Chemdraw file

## [D] Kinetin

PubChemID: 445468730
InChIKey: QANMHLXAZMSUEX-UICOGKGYSA-N
MDL Molfile
Chemdraw file

## [D] Inosine

PubChemID: 445468731
InChIKey: UGQMRVRMYYASKQ-MEGDDXBKSA-N
MDL Molfile
Chemdraw file

## [D] Adenine

PubChemID: 445468732
InChIKey: GFFGJBXGBJISGV-QDNHWIQGSA-N
MDL Molfile
Chemdraw file

## [D] Estrone

PubChemID: 445468733
InChIKey: DNXHEGUUPJUMQT-AAKDDEGHSA-N
MDL Molfile
Chemdraw file

## [D] Pentoxifylline

PubChemID: 445468734
InChIKey: BYPFEZZEUUWMEJ-BNEYPBHNSA-N
MDL Molfile
Chemdraw file

## [D] Brucine

PubChemID: 445468735
InChIKey: RRKTZKIUPZVBMF-OCUHVGHDSA-N
MDL Molfile
Chemdraw file

## [D] Strychnine

PubChemID: 445468736
InChIKey: QMGVPVSNSZLJIA-UQYZGTHLSA-N
MDL Molfile
Chemdraw file

## [D] Niacin

PubChemID: 445468737
InChIKey: PVNIIMVLHYAWGP-QYKNYGDISA-N
MDL Molfile
Chemdraw file

## [D] Tyrosol

PubChemID: 445468738
InChIKey: YCCILVSKPBXVIP-NMQOAUCRSA-N
MDL Molfile
Chemdraw file

## [D] Thymol

PubChemID: 445468739
InChIKey: MGSRCZKZVOBKFT-QRZVFWKLSA-N
MDL Molfile
Chemdraw file

## [D] Arbutin

PubChemID: 445468740
InChIKey: BJRNKVDFDLYUGJ-RCBWPJCFSA-N
MDL Molfile
Chemdraw file

## [D] Resveratrol

PubChemID: 445468741
InChIKey: LUKBXSAWLPMMSZ-BIFZJDEASA-N
MDL Molfile
Chemdraw file

## [D] Piceid

PubChemID: 445468742
InChIKey: HSTZMXCBWJGKHG-XNFYHSJESA-N
MDL Molfile
Chemdraw file

## [D] (±)Augustureine

PubChemID: 445468743
InChIKey: KSQZVAWGIAAZHJ-NXTQXPHYSA-N
MDL Molfile
Chemdraw file

## [D] Galipinine

PubChemID: 445468744
InChIKey: QRYQKXWZOXSQAJ-GSUVGXDCSA-N
MDL Molfile
Chemdraw file

## [D] Dropropizine

PubChemID: 445468745
InChIKey: PTVWPYVOOKLBCG-MICDWDOJSA-N
MDL Molfile
Chemdraw file

## [D] DL-Aminoglutethimide

PubChemID: 445468746
InChIKey: ROBVIMPUHSLWNV-KCZCTXNHSA-N
MDL Molfile
Chemdraw file

## [D] Nimodipine-*NH*2

PubChemID: 445468747
InChIKey: GUJDTMSUOZMUQI-LAJMJUAESA-N
MDL Molfile
Chemdraw file

## [D] Carvacrol

PubChemID: 445468748
InChIKey: RECUKUPTGUEGMW-KCZCTXNHSA-N
MDL Molfile
Chemdraw file

## [D] Estradiol

PubChemID: 445468749
InChIKey: VOXZDWNPVJITMN-JZTNAQEWSA-N
MDL Molfile
Chemdraw file

## [D] *O*-Desmethylvenlafaxine

PubChemID: 445468750
InChIKey: KYYIDSXMWOZKMP-XETGXLELSA-N
MDL Molfile
Chemdraw file

## [D] L-Tryptophan

PubChemID: 445468751
InChIKey: QIVBCDIJIAJPQS-RZRKSHIUSA-N
MDL Molfile
Chemdraw file

## *N*-(4-methoxyphenyl-2,6-*d*_2_)acetamide

PubChemID: 445468752
InChIKey: XVAIDCNLVLTVFM-NMQOAUCRSA-N
MDL Molfile
Chemdraw file

## [D] Paracetamol

PubChemID: 445468753
InChIKey: RZVAJINKPMORJF-PBNXXWCMSA-N
MDL Molfile
Chemdraw file

## [D] Lidocaine

PubChemID: 445468754
InChIKey: NNJVILVZKWQKPM-WHRKIXHSSA-N
MDL Molfile
Chemdraw file

## [D] Fluometuron

PubChemID: 445468755
InChIKey: RZILCCPWPBTYDO-WVALGTIDSA-N
MDL Molfile
Chemdraw file

## [D] Clortoluron

PubChemID: 445468756
InChIKey: JXCGFZXSOMJFOA-KCZCTXNHSA-N
MDL Molfile
Chemdraw file

## [D] Boscalid

PubChemID: 445468757
InChIKey: WYEMLYFITZORAB-UPXKZQJYSA-N
MDL Molfile
Chemdraw file
