# Supplementary material for: Scalable and selective deuteration of (hetero)arenes
Source: Nat Chem. 2022 Jan 13;14(3):334–41. doi: 10.1038/s41557-021-00846-4 (PMC8898765; doi:10.1038/s41557-021-00846-4)
Supplement: Supplementary file 1 — General remarks, procedure details for the preparation of the catalyst, deuteration reactions, reaction development and optimization, catalyst characterization, mechanistic studies, control experiments, scale-up reactions, catalyst recycling and characterization data of the substrates and products, Supplementary Figs. 1–18 and Tables 1–8. [file 41557_2021_846_MOESM193_ESM.pdf]

nature portfolio

https://doi.org/10.1038/s41557-021-00846-4

Supplementary information

Scalable and selective deuteration of (hetero)arenes

In the format provided by the authors and unedited

Supplementary Information for

Scalable and selective deuteration of (hetero)arenes

Wu Li[1], Jabor Rabeah[1], Florian Bourriquen[1], Dali Yang[2], Carsten Kreyenschulte[1], Nils Rockstroh[1], Henrik Lund[1], Stephan Bartling[1], Annette-Enrica Surkus[1], Kathrin Junge[1], Angelika Brückner[1*], Aiwen Lei[2*] and Matthias Beller[1*]

[1]Leibniz-Institut für Katalyse e.V., Albert-Einstein-Straße 29a, 18059 Rostock, Germany

[2]Institute for Advanced Studies (IAS), Wuhan University, 430072 Wuhan, Hubei (P. R. China)

[*]Corresponding author. Email: angelika.brueckner@catalysis.de; aiwenlei@whu.edu.cn; matthias.beller@catalysis.de

Contents

1. General remarks
2. Procedure for the preparation of the catalyst
3. General procedure for the deuteration reactions (GP)
4. Reaction development and optimisation
5. Comparison of H_2 and N_2
6. Catalyst characterisation by XRD, XPS, STEM and XANES/EXAFS
7. Mechanistic studies
   7.1. Kinetic isotope effect investigations
   7.2. EPR studies
8. Control experiments
   8.1. Control experiments without catalyst
   8.2. Control the quality
   8.3. Dehalogenation measurement
9. Scale up reactions
   9.1. 20 g and 50 g scale in 300 mL autoclave
   9.2. >300 g scale reactions in 2 L autoclave
10. Procedure for catalyst recycling
11. ICP-OES analysis
12. Characterisation data for substrates and products
13. ^1H NMR, ^{13}C NMR and ^{19}F NMR spectra for substrates and products
14. References

1. General remarks

Deuterated solvents were ordered from Deutero GmbH. NMR spectra were received using Bruker 300 Fourier, Bruker AV 300 and Bruker AV 400 spectrometers. Chemical shifts are reported in ppm relative to the deuterated solvent. Coupling constants are expressed in Hertz (Hz). The following abbreviations are used: s = singlet, d = doublet, t = triplet and m = multiplet. The residual solvent signals were used as references for ^{1}H and ^{13}C NMR spectra ($CDCl_3$: δH = 7.26 ppm, δC = 77.12 ppm; DMSO-d_6: δH = 2.50 ppm, δC = 39.52 ppm). The peak at 3.33 in DMSO-d_6 is from water. All measurements were carried out at room temperature unless otherwise stated. High resolution mass spectra (HRMS) were obtained either from a MAT 95 XP from Thermo (EI) or from an HPLC system 1200 and downstream ESI-TOF-MS 6210 from Agilent (ESI). Unless otherwise mentioned, all catalytic reactions were carried out in 4 mL vials, which were set in an alloy plate and placed inside 300 mL autoclave (PARR Instrument Company). 5,5-Dimethyl-1-pyrroline-*N*-oxide is from Dojindo Europe. Deuterium oxide (99.90% D) supplier is Eurisotop. The supplier of aniline-2,3,4,5,6-*d*5 (98% D) is Sigma-Aldrich. All other reagents and substrates including simple arenes, heteroarenes and complex drug molecules were purchased either from Sigma Aldrich, Alfa Aesar, Tokyo Chemical Industry (TCI) or Acros Organics and used directly. The ^{1}H NMR of the substrates were measured directly without further purification. The degree of deuterium incorporation was determined by the decrease of ^{1}H NMR signal intensities compare with the unlabelled one. In order to get a precise integration by quantitative NMRs. We did as follow: a) check for a proper phase correction. b) take care of appropriate baseline correction (baseline must be flat and, most important, situated at zero value of the vertical (y) axis). c) use always the same integration region, try to choose regions of a width that is defined by a constant multiple of the signal half-width. d) use the signals of the undeuterated part as internal reference which have equal intensity. In cases where deuterium exchange occurred in without the unlabelled part as internal reference, *e.g.* **6b**, **12b** and **19b**, we have introduced an acetyl group to corroborate the deuterium content. Internal standard trimethoxybenzene was also used to confirm the deuterium content of **5b** and **18b**.

2. Procedure for the preparation of the catalyst

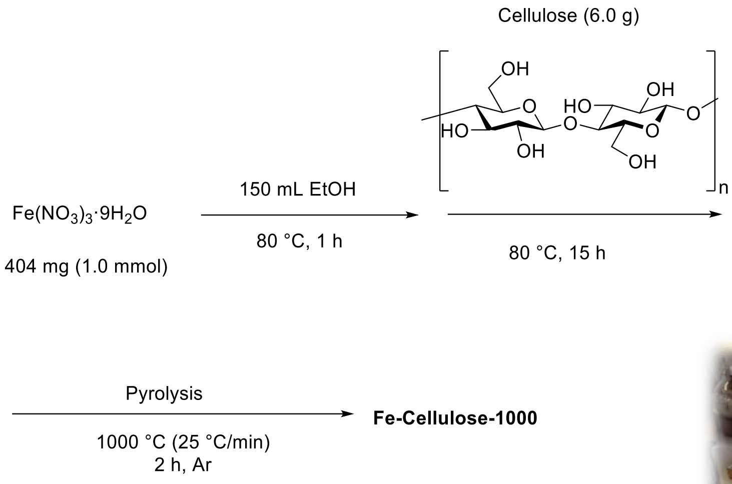

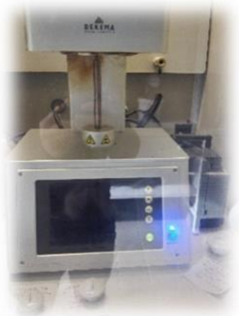

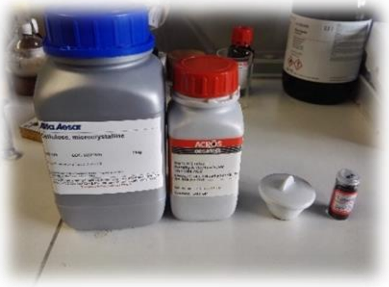

A 250-mL oven-dried single-necked round-bottomed flask equipped with a reflux condenser and a Teflon-coated, egg-shaped magnetic stir bar (40 mm× 18 mm) was charged with $Fe(NO_3)_3·9H_2O$ (404 mg, 1.0 mmol) and dissolved in ethanol (150 mL). Then, this mixture was heated to 80 °C (rt to 80 °C) in an oil bath and stirred for 1 hour. To the reaction solution, 6.0 g cellulose was added via a glass funnel, and the resulting heterogeneous mixture was stirred at 450 rpm for 15 hours at 80 °C. The flask was taken out from the bath and cooled to ambient temperature. The solvent was removed in vacuum and then dried under an oil pump vacuum for 4 hours to give a yellow solid. The sample was transferred to a ceramic crucible and placed in an oven (see above). The latter was evacuated to ca. 5 mbar and then flushed with argon three times. The furnace was heated to 1000 °C at a rate of 25 °C/min and held at 1000 °C for 2 hours under argon atmosphere. After the heating was switched off, the oven was allowed to reach room temperature, giving the Fe-Cellulose-1000 catalyst as a black powder (note that during the whole process, argon was constantly passed through the oven). (Elemental analysis: Fe 4.9 wt%).

Elemental analysis:

Instrument: ContrAA 800D #10-1610D-AR149

Tech: Flamme

SW-Version: ASpect CS 2.2.1.0 Created:

22.11.2019 12:12

Datum	Time	Name (2)	Line	Conc.2	Unit
11/22/2019	11:31	Wu Li WU-7-391	Fe248	4.902	M- %
11/22/2019	11:59	Wu Li WU-7-391	Fe248	4.951	M- %

3. General procedure for the deuteration reactions (GP)

In a 4 mL vial fitted with magnetic stirring bar and septum cap, iron catalyst (60 mg) and substrate (0.25 mmol) were added. Then, a needle was inserted in the septum which allows gaseous reagents to enter. After adding the solvent deuterium oxide (1.5 mL), the vials (up to eight) were set in an alloy plate and then placed into a 300 mL steel Parr autoclave. The autoclave was flushed with hydrogen 6 times at 10 bar and finally pressurized to the desired value (20 bar). Then, it was placed into an aluminum block and heated to the desired temperature. At the end of the reaction, the autoclave was quickly cooled down to room temperature with an ice bath and vented. Finally, the samples were removed from the autoclave, and ethyl acetate was added to the crude mixture. This mixture was centrifuged, and the organic layer was removed from the vials (3 times). After removal of all volatiles in vacuo, the desired products were obtained. In case of anilines with D-labelling on the nitrogen, 1 mL H_2O was added during work up and N-D was replaced by N-H.

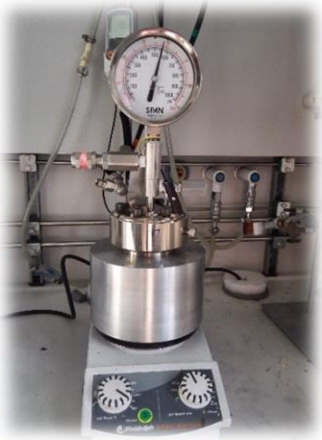
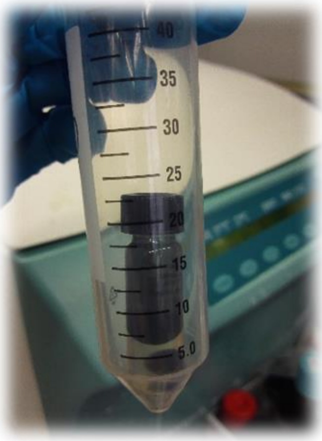
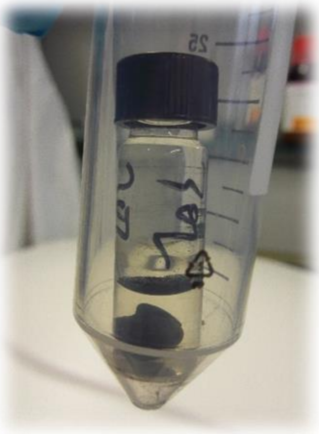
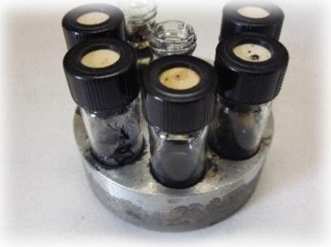
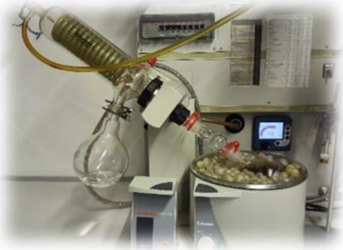
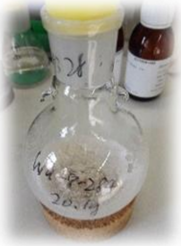

4. Reaction development and optimisation

Supplementary Table 1 Catalyst screening

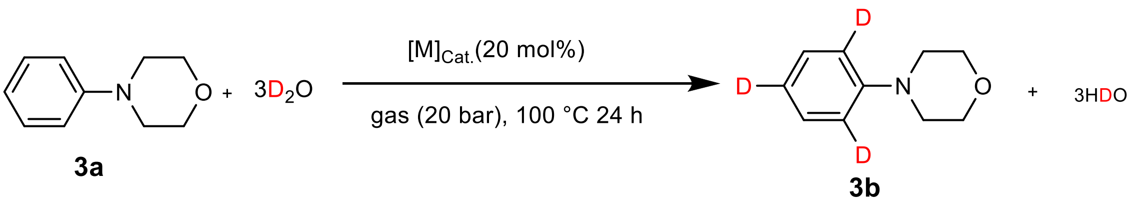

3a **3b**

Entry	Catalyst	Gas	Solvent	D Total/molecular
1	Pd/C	N_2	D_2O	0
2	Pt/C	N_2	D_2O	1.41
3	Au/C	N_2	D_2O	0.90
4	Ru/C	N_2	D_2O	0.93
5	Co-Phen/C-800	N_2	D_2O	0
6	Cu-Phen/C-800	N_2	D_2O	0
7	Fe-Phen/C-800	N_2	D_2O	0
8	Co-Cellulose-800	N_2	D_2O	0.24
9	Cu-Cellulose-800	N_2	D_2O	0
10	Fe-Cellulose-800	N_2	D_2O	1.62
11	Fe-Cellulose-1000	N_2	D_2O	2.01
12	Fe-Cellulose-1000	**H_2**	D_2O	2.43
**13**	**Fe-Cellulose-1000**	**H_2**	**D_2O**	**2.88[†]**
14	Fe-Cellulose-1000	D_2	H_2O	0
15	$Fe(OAc)_2$	N_2	D_2O	0
16	$Fe(NO_3)_3$	N_2	D_2O	0
17	Cellulose-1000	N_2	D_2O	0
18	HCl aqueous	N_2	D_2O	0
19	NaOH	N_2	D_2O	0
20	Fe-Cellulose-1000	N_2	DMSO-*d6*	0
21	Fe-Cellulose-1000	N_2	MeOH-*d4*	0
22	Fe-Cellulose-1000	N_2	THF-*d8*	0
23	Fe-Cellulose-1000	N_2	Toluene-*d8*	0
24	Pd/C	H_2	D_2O	0
25	Pt/C	H_2	D_2O	0
26	Ru/C	H_2	D_2O	0
27	Fe-Phen/C-800	H_2	D_2O	0
28	Co-Cellulose-1000	H_2	D_2O	0.27
29	Cu-Cellulose-1000	H_2	D_2O	0

Standard conditions: 4-Phenylmorpholine (0.25 mmol, 1 equiv.), [M] catalyst (20 mol%), D_2O (1.5 mL), 20 bar gas, 100 °C, 24 h. [†]120 °C. Deuterium content determined by quantitative ^1H NMR: decrease of the signal corresponding to the aromatic protons, using the morpholine signals as internal calibration.

Initially, we tested standard, commercially available heterogeneous catalysts and tailor-made supported nanoparticles for selective deuteration of 4-phenylmorpholine in D_2O (Supplementary Table 1). This benchmark substrate was chosen because it permits labelling both at the *N*-containing heterocycle and the phenyl ring. In all cases, the extent of isotopic exchange was determined using ^1H NMR spectroscopy. In agreement with previous works[1], palladium on carbon (10 weight-%) led to moderate deuterium incorporation (64%) at the α-position of the nitrogen atom on the morpholine ring. In contrast, in the presence of other supported noble metal catalysts such as Pt/C, Au/C and Ru/C deuterium labelling was observed in the *ortho*- and *para*-positions of the phenyl ring with 1.41, 0.90 and 0.93 D/molecule, respectively (Supplementary Table 1, entries 2-4).

Recently, we introduced a variety of supported 3d-metal nanoparticles (NPs) for selective hydrogenation and oxidation reactions[2-5]. In this context, iron-based NPs are particularly attractive to us due to the abundance, low cost and negligible safety concerns of Fe salts. Typically, these materials are prepared by pyrolysis of *N*-ligated metal complexes in the presence of carbon (Vulcan XC72R) or standard inorganic supports. Unfortunately, none of them including Co-Phen/C-800, Cu-Phen/C-800, Fe-Phen/C-800 (Phen = 1,10-phenanthroline; 800 refers to the pyrolysis temperature) showed any labelling activity for our benchmark system (Supplementary Table 1, entries 5-7). Interestingly, in the presence of a cellulose-derived cobalt material, small amounts of deuteration of the phenyl ring (8%, 0.24 D/molecule) are achieved (Table 1, entries 6 and 7). Much to our surprise, pyrolysis of iron(II) acetate or iron(III) nitrate nonahydrate with cellulose resulted in highly active and selective catalytic systems (1.62 D/molecule) for deuteration of the phenyl ring in the *ortho*- and *para*-positions, which even outperformed all the commercial platinum-group metal catalysts (Table 1, entry 10 *vs* entries 2 and 4). Performing pyrolysis of iron(III) nitrate nonahydrate and cellulose at 1000 °C furnished the best catalyst (Fe-Cellulose-1000), which gave increased deuterium incorporation (2.01 D/molecule) (Supplementary Table 1, entry 11). The activity could be further improved by activation of the pre-catalyst with hydrogen, which likely generates active Fe(0) species *vide infra* (see catalyst characterisation) (Supplementary Table 1, entry 12). The activity of the iron catalyst is improved in the presence of hydrogen (the presence of H_2 accelerates the reaction and increases the D-incorporation). Furthermore, the stability and recyclability of the catalyst under H_2 are better (Supplementary Information, section 5). Finally, performing the benchmark reaction on D_2O in the presence of hydrogen at 120 °C gave nearly quantitative deuteration (96%, 2.88 D/molecule), whilst no deuterium-labelled product was obtained using D_2 in H_2O (Table 1, entries 13 and 14). We have performed additional experiments with the catalysts under H_2 pressure, too (entries 24 and 29). There is no formation of D-labelled products observed in the presence of Pd/C, Pt/C, and Ru/C except for hydrogenated products on the aromatic ring (entries 24-26). Furthermore, there is no improvement observed using Fe-Phen/C-800, Co-Cellulose-1000 and Cu-Cellulose-1000 under H_2 pressure (entries 27-29).

As expected, comparison experiments under identical conditions revealed no labelling products in the presence of homogeneous $Fe(OAc)_2$, $Fe(NO_3)_3 \cdot 9H_2O$ or simple

pyrolyzed cellulose (Supplementary Table 1, entries 15-17). Similarly, using acid (20 mol% of HCl) or base (20 mol% NaOH) under the standard conditions gave no deuterium-incorporated products (Supplementary Table 1, entries 18 and 19). Furthermore, we have studied the additional solvents like toluene, EtOH, THF and dioxane (Supplementary Table 2, entries 2-5), but significantly lower D-product was detected.

Supplementary Table 2 Co-solvents

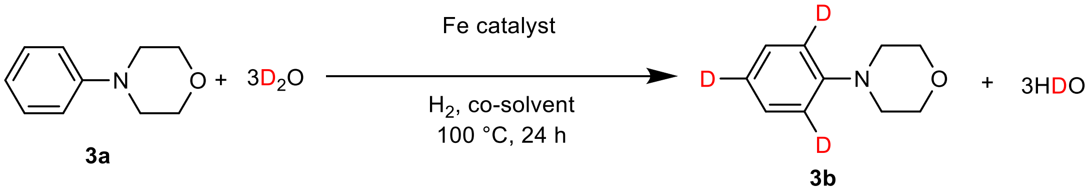

3a

3b

Entry	D_2O	Co-solvent	Total-D (%)
1	1.5 mL	--	81
2	0.5 mL	Toluene (1.5 mL)	34
3	0.5 mL	EtOH (1.5 mL)	23
4	0.5 mL	THF (1.5 mL)	17
5	0.5 mL	1, 4-Dioxane (1.5 mL)	12

Standard conditions: 4-Phenylmorpholine (0.25 mmol, 1 equiv.), [M] catalyst (20 mol%), D_2O (0.5 mL), co-solvents (1.5 mL), 20 bar H_2, 100 °C, 24 h. Deuterium content determined by quantitative ^{1}H NMR: decrease of the signal corresponding to the aromatic protons, using the morpholine signals as internal calibration.

Procedure for Supplementary Table 2:

In a 4 mL vial fitted with magnetic stirring bar and septum cap, iron catalyst (60 mg) and substrate (0.25 mmol) was added. Then, a needle was inserted in the septum which allows gaseous reagents to enter. After adding the deuterium oxide (0.5 mL) and co-solvents, the vials (up to eight) were set in an alloy plate and then placed into a 300 mL steel Parr autoclave. The autoclave was flushed with hydrogen 6 times at 10 bar and finally pressurized to the desired value (20 bar). Then, it was placed into an aluminum block and heated to the desired temperature. At the end of the reaction, the autoclave was quickly cooled down to room temperature with an ice bath and vented. Finally, the samples were removed from the autoclave, and ethyl acetate was added to the crude mixture. This mixture was centrifuged, and the organic layer was removed from the vials (3 times). After removal of all volatiles in vacuo, the desired products were obtained for NMR measurements.

5. Comparison of H_2 and N_2

The activity of the iron catalyst is improved in the presence of hydrogen (the presence of H_2 accelerates the reaction and increases the D-incorporation; see Supplementary Table 3 and Fig. 2). Furthermore, the stability and recyclability of the catalyst under H_2 are better (see Supplementary Fig. 1, and the procedure in Section 10).

Supplementary Table 3

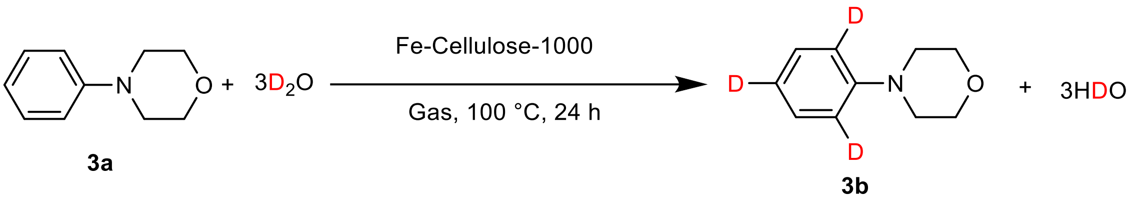

Entry	Gas	Total-D (%)
1	N_2 (20 bar)	67
2	H_2 (20 bar)	81
3	H_2 (5 bar)	75

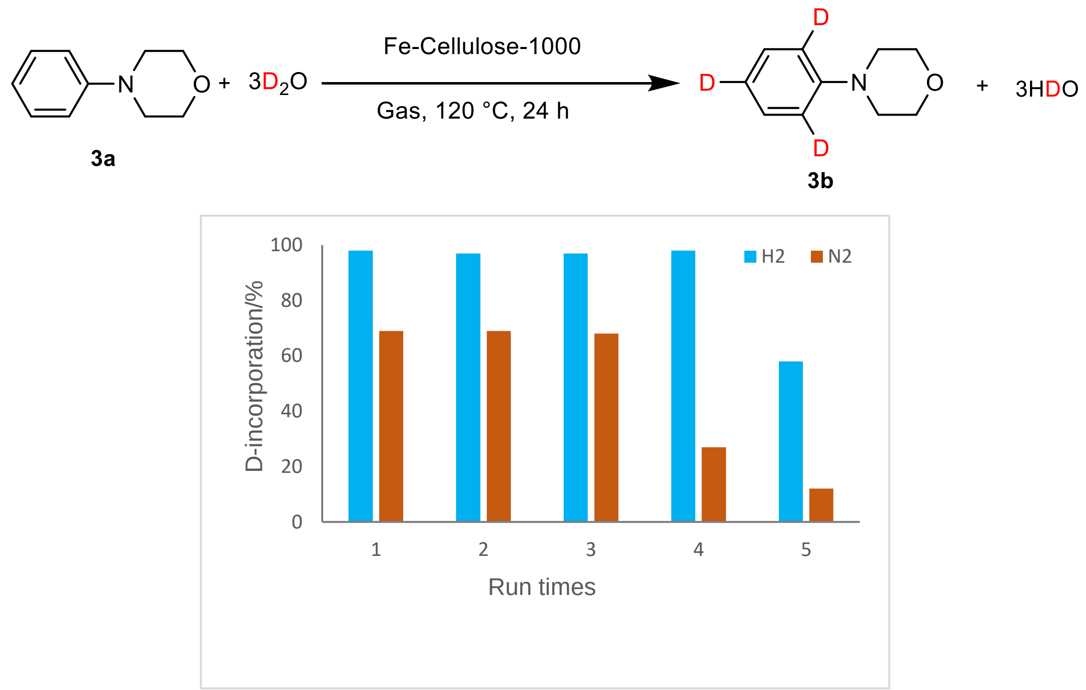

Supplementary Fig. 1 Recycling experiments

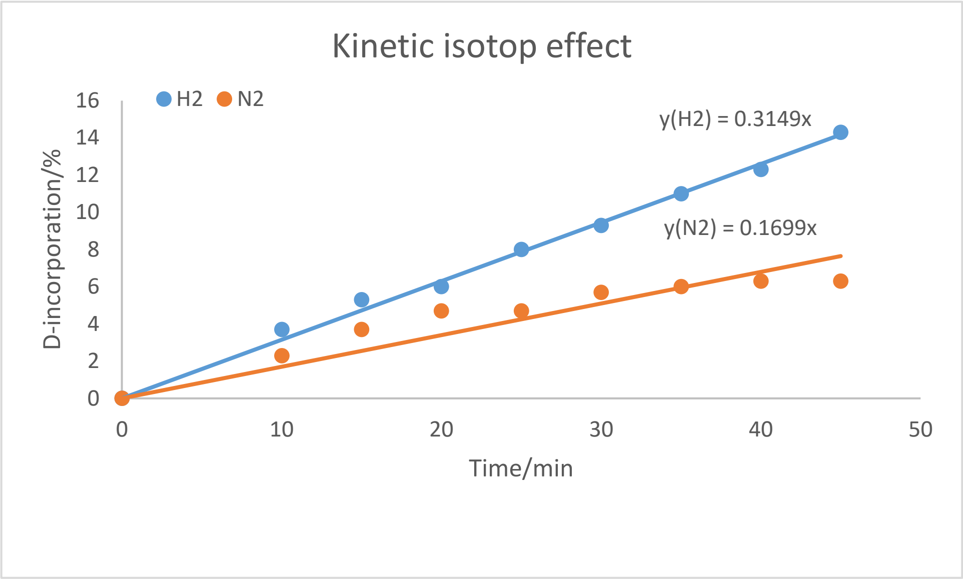

Supplementary Fig. 2 Kinetic studies

Under H_2:

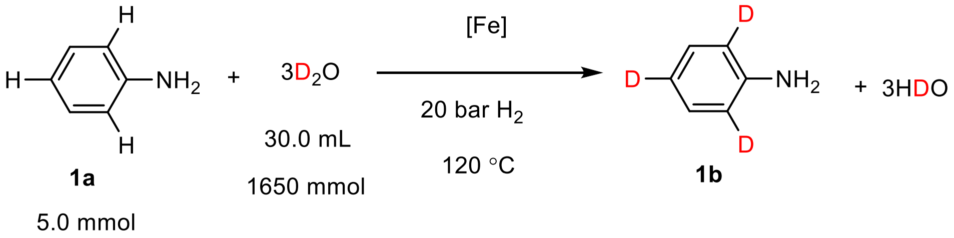

In a 50 mL steel Parr autoclave (see below) loaded with Fe-Cellulose-1000 catalyst (300 mg, 0.21 mmol), aniline (465 mg, 5.0 mmol) and deuterium oxide 30.0 mL (1650 mmol) were added. The autoclave was flushed with hydrogen 6 times at 10 bar and finally pressurized to the desired value (20 bar). Then, it was it placed into an equipment for the autoclave and heated to 120 °C (about 10 min). The first sample was taken 10 min after heating. Then, the other 7 samples were taken each 5 min. The 8 samples were measured directly in D_2O by ^1H NMR (see below).

Under N_2:

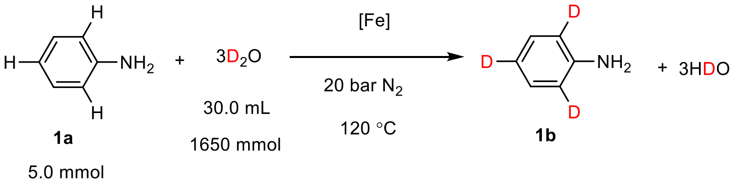

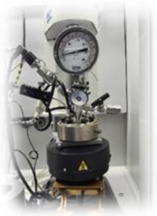

In a 50 mL steel Parr autoclave (see above) loaded with Fe-Cellulose-1000 catalyst (300 mg, 0.21 mmol), aniline (465 mg, 5.0 mmol) and deuterium oxide 30.0 mL (1650 mmol) were added. The autoclave was flushed with N_2 6 times at 10 bar and finally pressurized to the desired value (20 bar). Then, it was it placed into an equipment for the autoclave and heated to 120 °C (about 10 min). The first sample was taken 10 min after heating. Then, the other 7 samples were taken each 5 min. The 8 samples were measured directly in D_2O by ^1H NMR (see below).

NMR results under H_2:

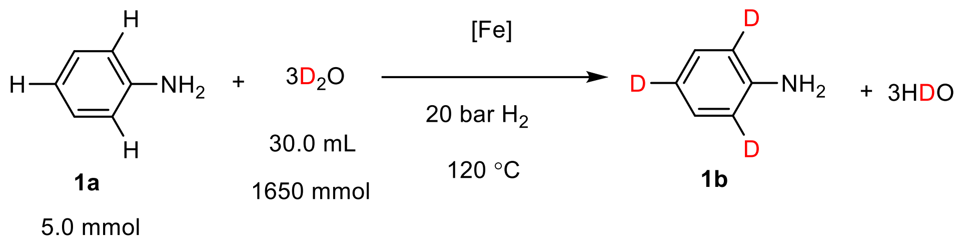

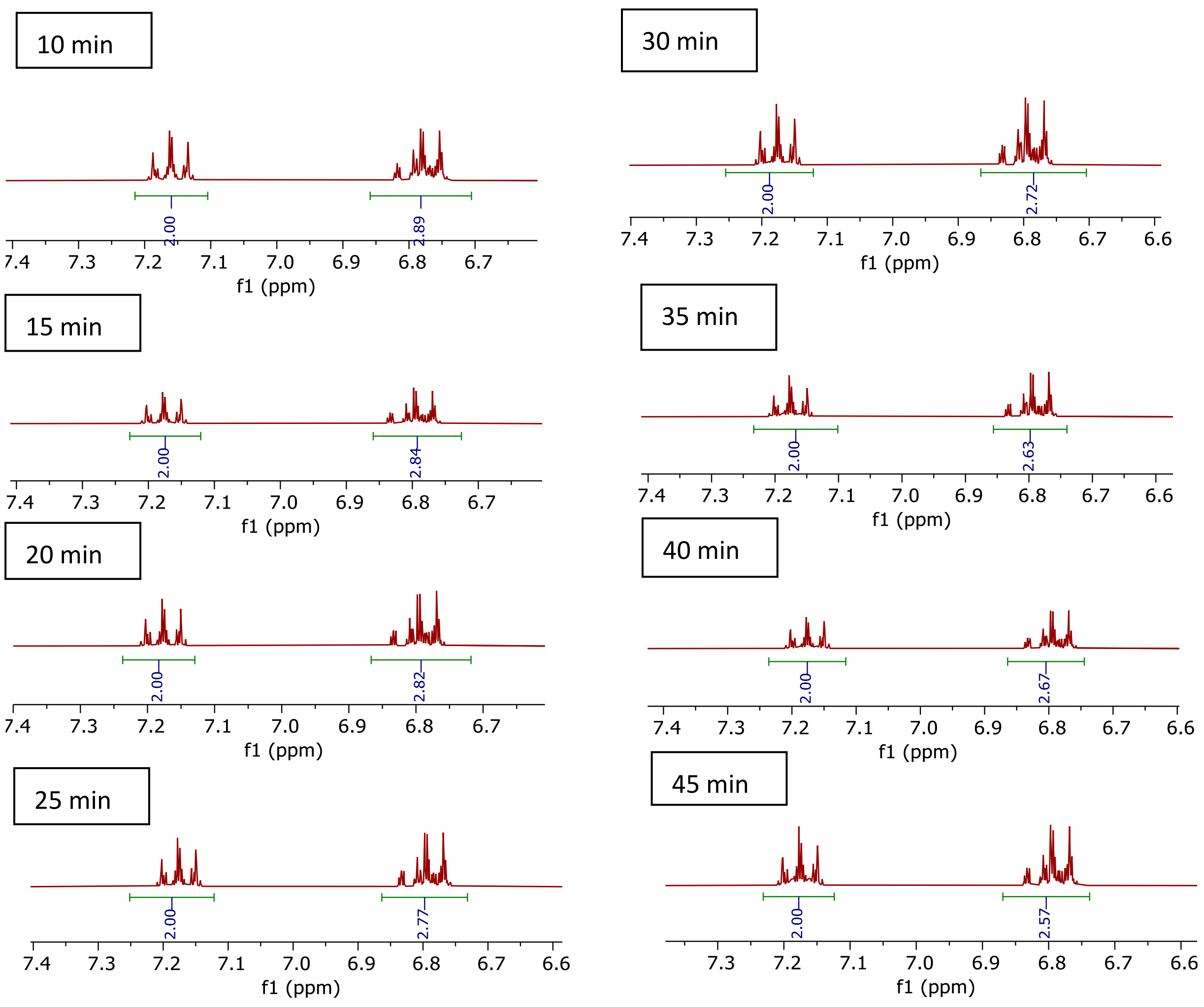

NMR results under N_2:

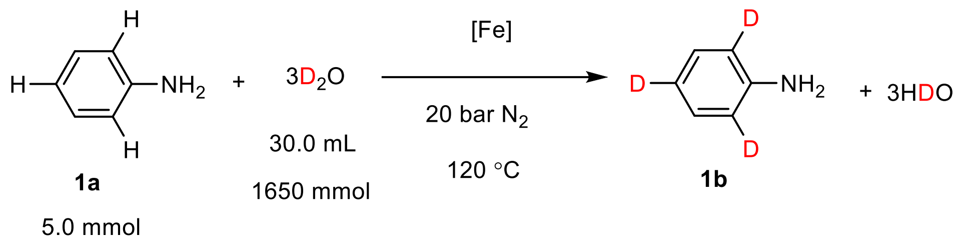

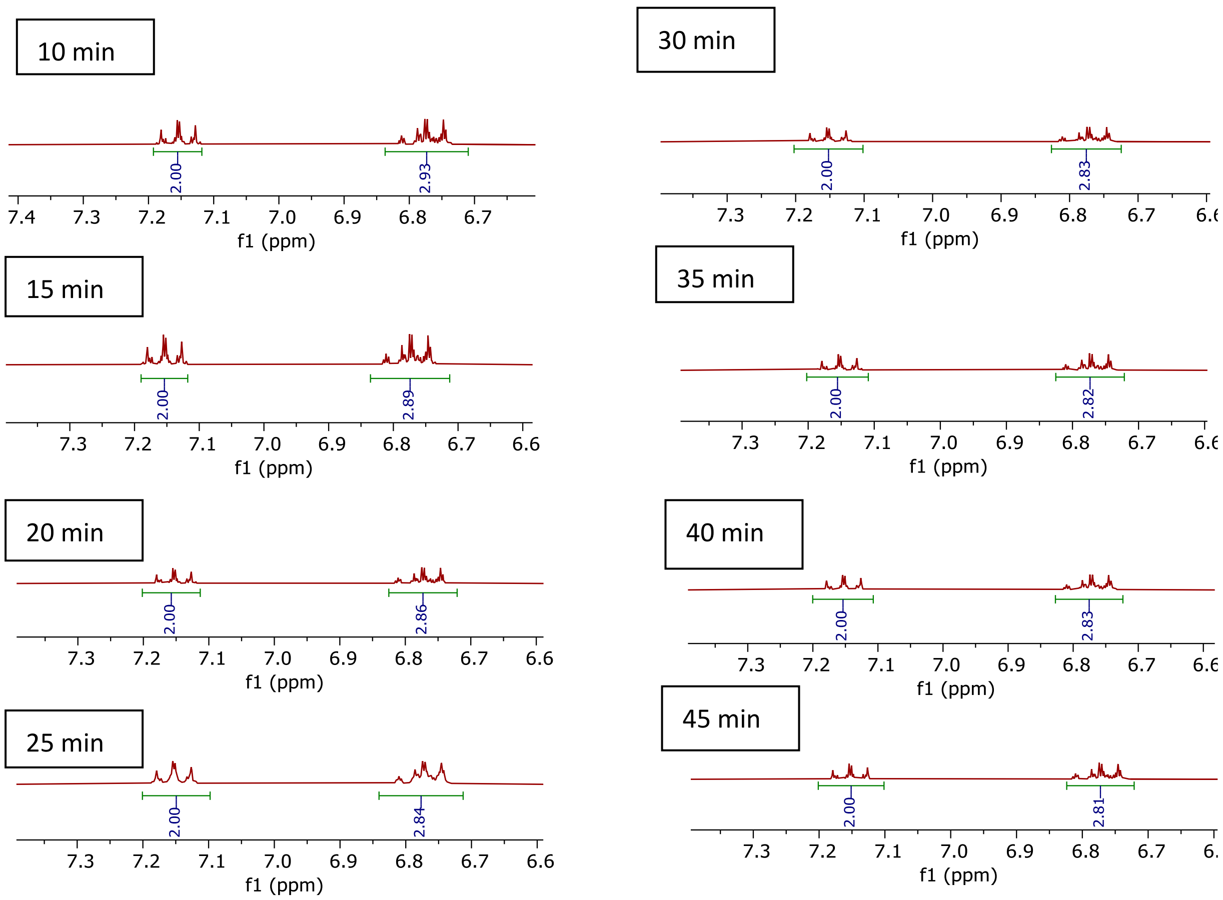

6. Catalyst characterisation by XRD, XPS, STEM and XANES/EXAFS

Experimental procedures

To understand the structural features of the most active material (Fe-Cellulose-1000), powder X-Ray diffraction (PXRD), X-Ray photoelectron spectroscopy (XPS), scanning transmission electron microscopy (STEM) and X-Ray absorption spectroscopy (XAS) investigations were performed.

XRD powder patterns were recorded on a Panalytical X'Pert diffractometer equipped with a Xcelerator detector using automatic divergence slits and Cu kα1/α2 radiation (40 kV, 40 mA; λ= 0.15406 nm, 0.154443 nm). Cu beta-radiation was excluded using a nickel filter foil. After mounting the sample on silicon zero background holders the measurement was performed on air at room temperature with either 0.0211°s-1 or 0.005°s-1 scan velocity. The obtained intensities were converted from automatic to fixed divergence slits (0.25°) for further analysis. Peak positions and profile were fitted with Pseudo-Voigt function using the HighScore Plus software package (Panalytical). Phase identification was done by using the PDF-2 database of the International Center of Diffraction Data (ICDD).

The XPS (X-ray Photoelectron Spectroscopy) measurements were performed on an ESCALAB 220iXL (Thermo Fisher Scientific) with monochromated Al Kα radiation (E = 1486.6 eV, 125 W, spot size 400 µm) and a base pressure of 2×10-9 mbar. All the samples are prepared as received on a stainless-steel holder with conductive double-sided adhesive carbon tape, and no further pretreatment has been applied before analysis. The electron binding energies were referenced to the C 1s peak at 284.3 eV. For quantitative analysis the peaks were deconvoluted with Gaussian-Lorentzian curves using the software Unifit 2020. The peak areas were normalized by the transmission function of the spectrometer and the element specific sensitivity factor of Scofield[6]. For pseudo in situ XPS measurements under 20 bar H_2 atmosphere, a laboratory based Near Ambient Pressure X-ray photoelectron spectroscopy system (NAP-XPS, SPECS Surface Nano Analysis GmbH, Germany) was used. The setup is equipped with a differentially pumped Phoibos 150 electron energy analyzer with a nozzle of 500 µm and a monochromated Al Kα radiation source (E = 1486.6 eV, 70 W, spot size about 300 µm). The system is connected to a separate high-pressure cell (HPC 20, SPECS Surface Nano Analysis GmbH, Germany). After treatment at 20 bar H_2 at the desired temperature the sample was cooled to room temperature and transferred under vacuum to the analysis chamber with a base pressure of 2×10^{-9} mbar.

Scanning transmission electron microscopy (STEM) was performed in a probe aberration corrected JEM-ARM200F (JEOL, Japan) using high angle annular dark field (HAADF) and annular bright field (ABF) detectors for imaging. Annular dark field (ADF) imaging was used as positional reference when spectrum imaging (SI) with the electron energy loss spectrometer (EELS) (Enfinium ER, Gatan, USA) was applied for chemical analysis. The specimen was dry deposited onto a Cu grid (mesh 300) covered by a holey carbon film and then transferred into the microscope.

X-Ray absorption measurement was acquired at the beamline 44A of Taiwan Photon

Source (TPS), National Synchrotron Radiation Research Center (NSSRC) in Taiwan. The data was collected in transmission mode. The beamline utilizes a water cooled double-crystal Si(111) monochromator and grid ionization chambers, which were optimized for the maximum current with linear response (~10^{10} photons detected/sec) with 3 bar N_2 in all three of them (I_0, I_t and I_{ref}). A Fe foil spectrum (edge energy 7112 eV) was acquired simultaneously with the measurement for energy calibration. Multiple scans were taken to improve the signal-to-noise ratio. The edge energy of the X-Ray absorption near edge structure (XANES) spectra was determined from the first maximum of inflection point in the edge. Background removal and normalization procedures were carried out using the Athena software (Demeter 0.9.26) package. EXAFS fitting was performed using Artemis software (Demeter 0.9.26) by a least square fit in R-space of the nearest neighbor, k^2-weighted Fourier transform data. Samples for XAS analysis were prepared by finely grinding 10 mg catalyst powder together with 20 mg boron nitride. Then, 15 mg of the grinded solid mixture was loaded into a 4 mm diameter sample holder, then pressed into a sample wafer. The XAFS measurement was done under air at room temperature. The edge step is around 0.5 and the total absorption length is below 2.

EPR spectra were recorded on a Bruker EMX-micro cw-EPR spectrometer (X-band, $\nu \approx 9.7$ GHz) at different temperature with a microwave power of 6.9 mW, a modulation frequency of 100 kHz and modulation amplitude up to 5G. The EPR spectrometer is equipped with a variable temperature control unit including a liquid N_2 cryostat and a temperature controller. EPR spectra were simulated using EasySpin.

Elemental analysis was measured by Instrument: ContrAA 800D #10-1610D-AR149 (see section 3). An Fe content of 4.9 % was determined in the fresh catalyst after pyrolysis.

Results and discussion

PXRD measurements were carried out to check for crystalline components of the fresh catalyst after pyrolysis and the used one after the catalytic reaction. Supplementary Fig. 3 shows a comparison of the fresh catalyst with those obtained after use in reactions 1 and 2:

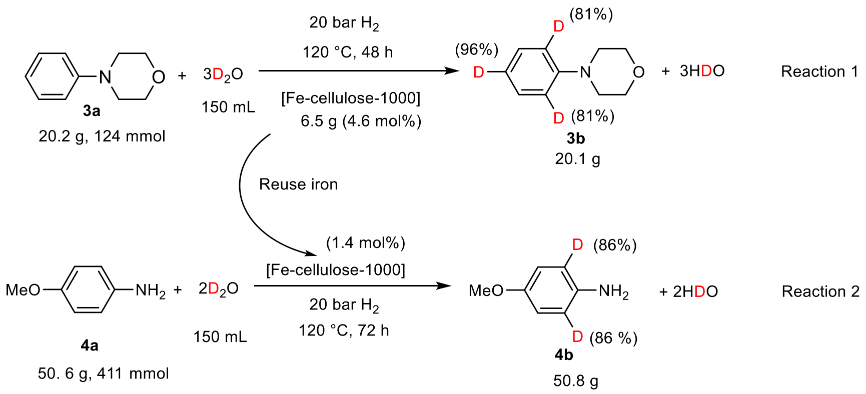

The powder patterns of all three samples exhibit a broad peak around 26° 2theta which indicates the presence of a disordered carbon phase formed during the decomposition

of cellulose. Iron is present mainly as Fe_3C. Besides, peaks at 44.62° and 65.00° are assigned to α-Fe while those at 50.94° and 74.94° might stem from γ-Fe[7]. These results suggest that the $Fe(NO_3)_3$ precursor is reduced during pyrolysis under an argon atmosphere. Most probably, cellulose and/or products of its pyrolysis such as char, aerosols as well as levoglucosan, furans and pyrans act as reductants[8]. Interestingly, the peaks of Fe_3C decrease after use in the catalytic reaction for 48 h and even more after additional 72 h (Supplementary Fig. 3). This is also true for the peaks of metallic Fe while weak peaks of Fe_3O_4 are observed in the patterns of the used catalyst. The latter phase is most probably formed by oxidation of metallic Fe during exposure of the used samples to ambient atmosphere, as discussed below.

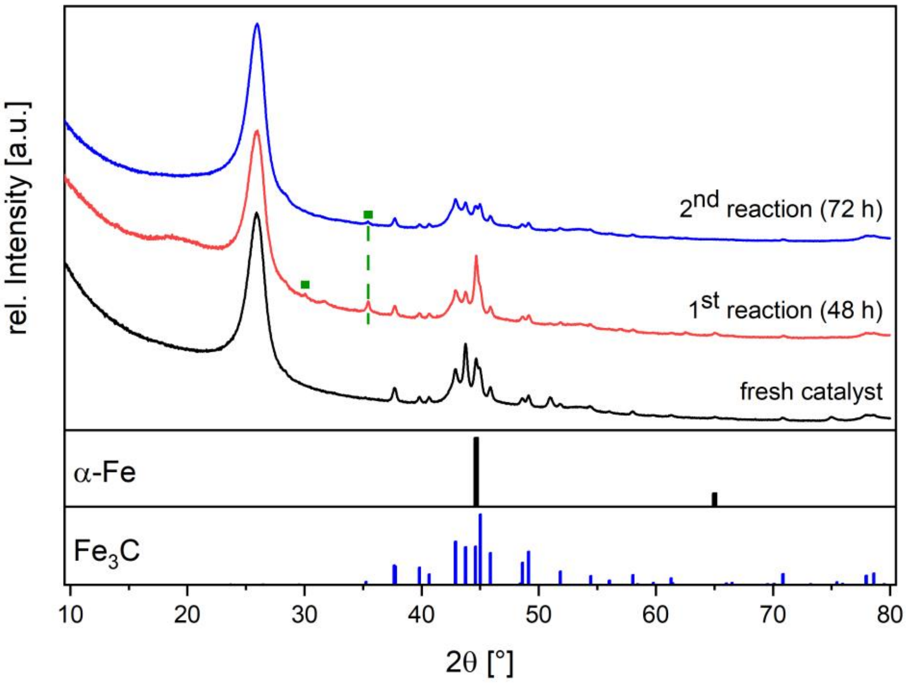

Supplementary Fig. 3 Normalized powder patterns of fresh and used Fe-Cellulose-1000 after 48 h and additional 72 h. References including peak positions and relative intensities below: Fe_3C (pdf 01-074-6457) and α-Fe (pdf 00-006-0696). Green squares indicate reflection peaks of Fe_3O_4 (pdf 01-079-0418).

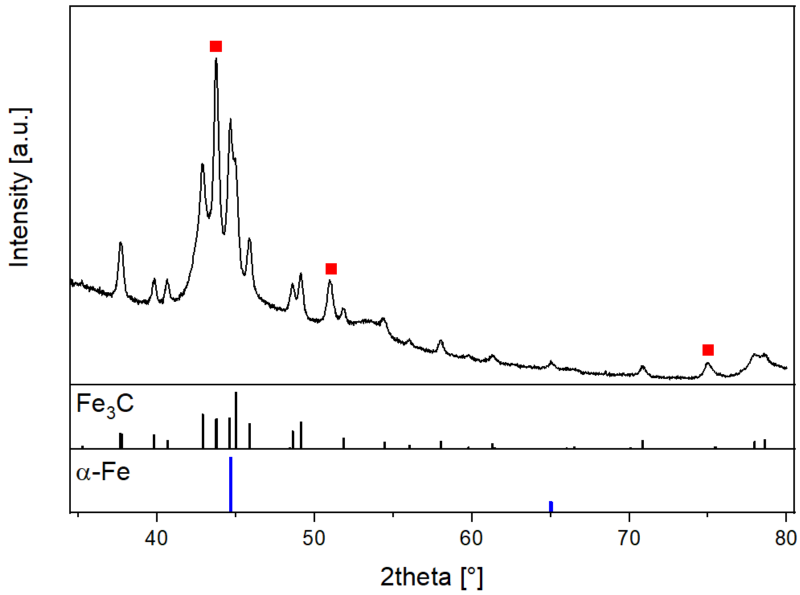

Supplementary Fig. 4 Magnification of PXRD pattern of fresh Fe-Cellulose-1000 sample. Red squares represent peak positions of γ-Fe.

To obtain further information about the surface valence states, XPS measurements were performed on the same catalysts (Supplementary Fig. 5). The fresh catalyst shows a sharp Fe $2p_{3/2}$ peak at around 707.0 eV, characteristic for zerovalent iron. In contrast, broad features starting at around 711.5 eV can be observed for the used catalysts, indicating the formation of oxidized Fe^{2+} and Fe^{3+} species. The Fe surface content in the fresh catalyst is with about 0.1 atom-% rather low. This suggest that Fe is widely embedded in the carbon matrix, as confirmed by STEM results described below.

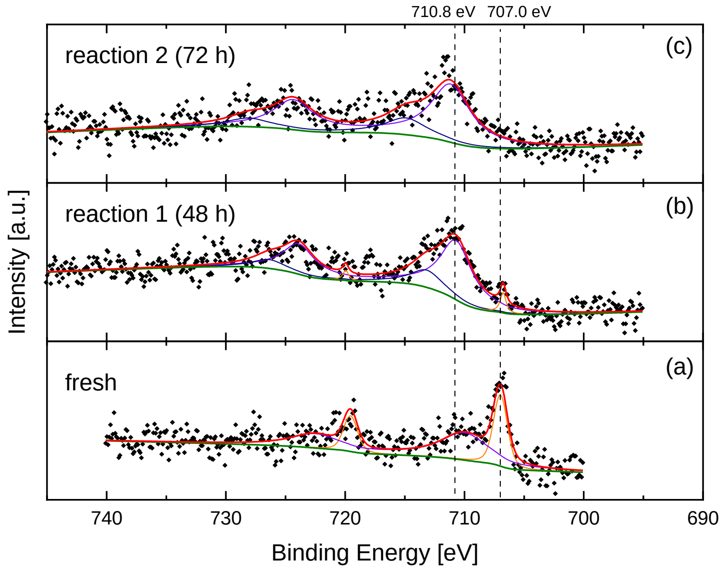

Supplementary Fig. 5 XPS spectra in the Fe 2p range of fresh Fe-Cellulose-1000 (a), used after 48 h in reaction 1 (b), and additional 72 h in reaction 2 (c).

The oxide phase in the used catalysts is most probably formed by oxidation of metallic Fe after removal from the reactor and exposure to ambient atmosphere. This can be concluded from a pseudo in situ XPS experiment in which the catalyst used for 72 h in reaction 2 (Supplementary Fig. 5c and 6a) was first treated at 200 °C for 6 h in 20 bar H_2 (Supplementary Fig. 6b) and then exposed to ambient atmosphere for 2 h (Supplementary Fig. 6c). It can be clearly seen that metallic Fe formed in H_2 (peak at 706.9 eV in Fig. 6b) disappeared completely in ambient air (Supplementary Fig. 6c)

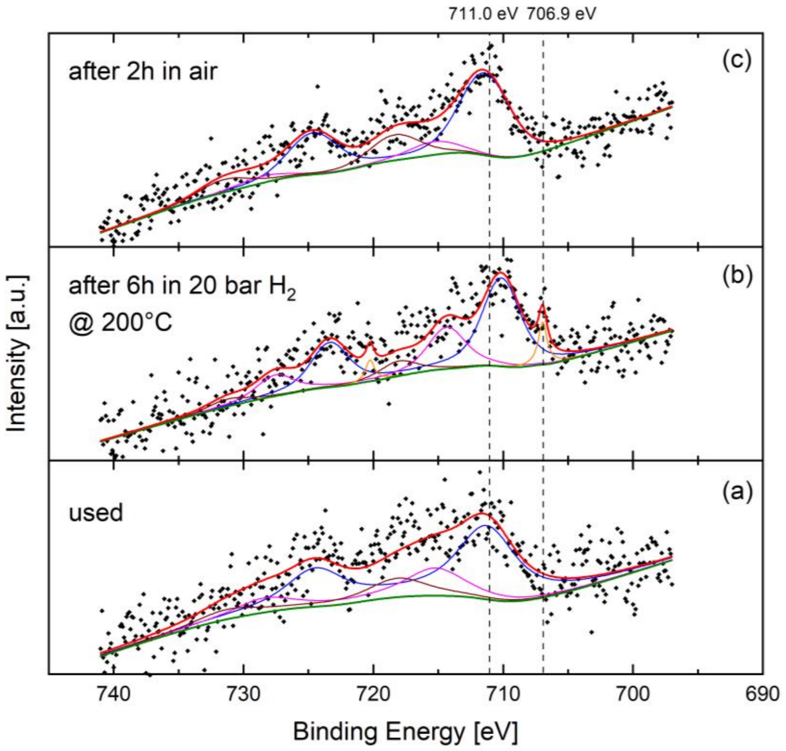

Supplementary Fig. 6 XPS spectra in the Fe 2p region of the catalyst used for 72 h in reaction 2 (a), after in situ treatment under 20 bar H_2 at 200 °C for 6 h (b) and after exposure to ambient air for about 2 h (c).

STEM micrographs of the fresh Fe-Cellulose-1000 catalyst revealed that Fe-containing particles are distributed in the carbon matrix (Supplementary Fig. 7c and d) and surrounded by a shell of up to 30 graphene layers with a thickness of 6-10 nm (Supplementary Fig. 7a and b). Most of the observed Fe(0) nanoparticles were within a range of 20 to 50 nm in diameter (Supplementary Fig. 7). Interestingly, no oxygen could be detected by EELS on the surface of the Fe particles (Supplementary Fig. 7f) which is in good agreement with XRD and XPS results, containing negligible indication for iron oxides. This is different for the catalysts used for 48 h in reaction 1 (Supplementary Fig. 8) and 72 h in reaction 2 (Supplementary Fig. 9). While the size of the Fe containing particles and also their distribution within the carbon matrix does not change much, their carbon cover layer as observed in the fresh catalyst

(Supplementary Fig. 7a and b) disappeared widely. Instead, the particles are covered by an iron oxide phase, clearly evidenced by oxygen in the EELS spectra (Supplementary Fig. 8b and 9b) and by elemental mapping (Supplementary Fig. 8d and 9d). However, as suggested by pseudo in situ XPS (Supplementary Fig. 6), this oxide layer might have been formed by exposure of the used catalysts to ambient air.

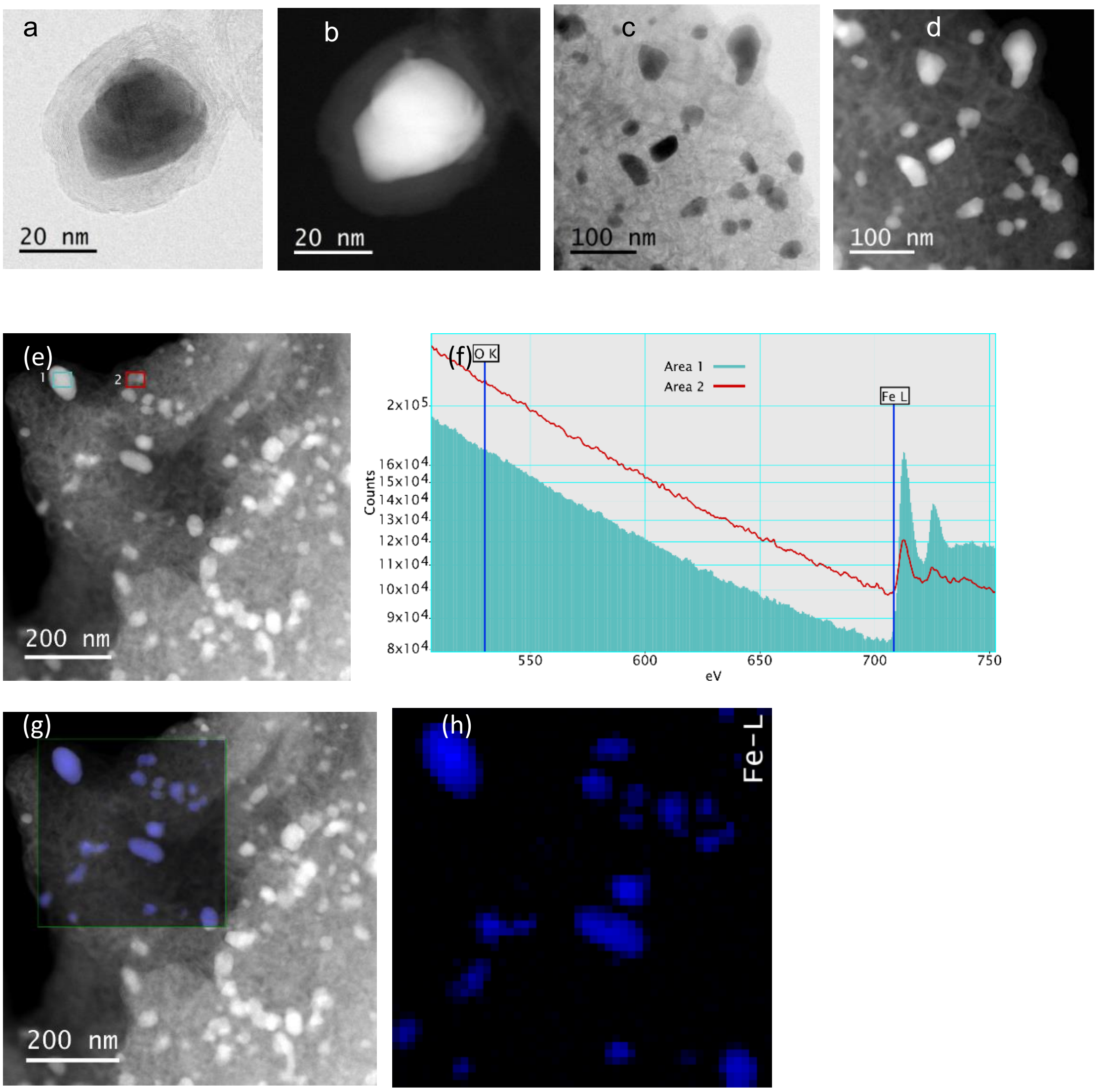

Supplementary Fig. 7 A pair of high resolution ABF-STEM (a) and HAADF-STEM (b) images of an Fe particle of Fe-Cellulose-1000 showing coating of its surface by graphene layers and the corresponding pair of ABF-STEM (c) and HAADF-STEM (d) images showing an overview of the sample. STEM-ADF image (e) with marked areas used for extraction of the EELS spectra (f). The same data set was then used to calculate the elemental map for iron, overlaid onto the ADF image (g) and independently (h). Within the detection limit, no oxygen could be observed at the particles or within the support.

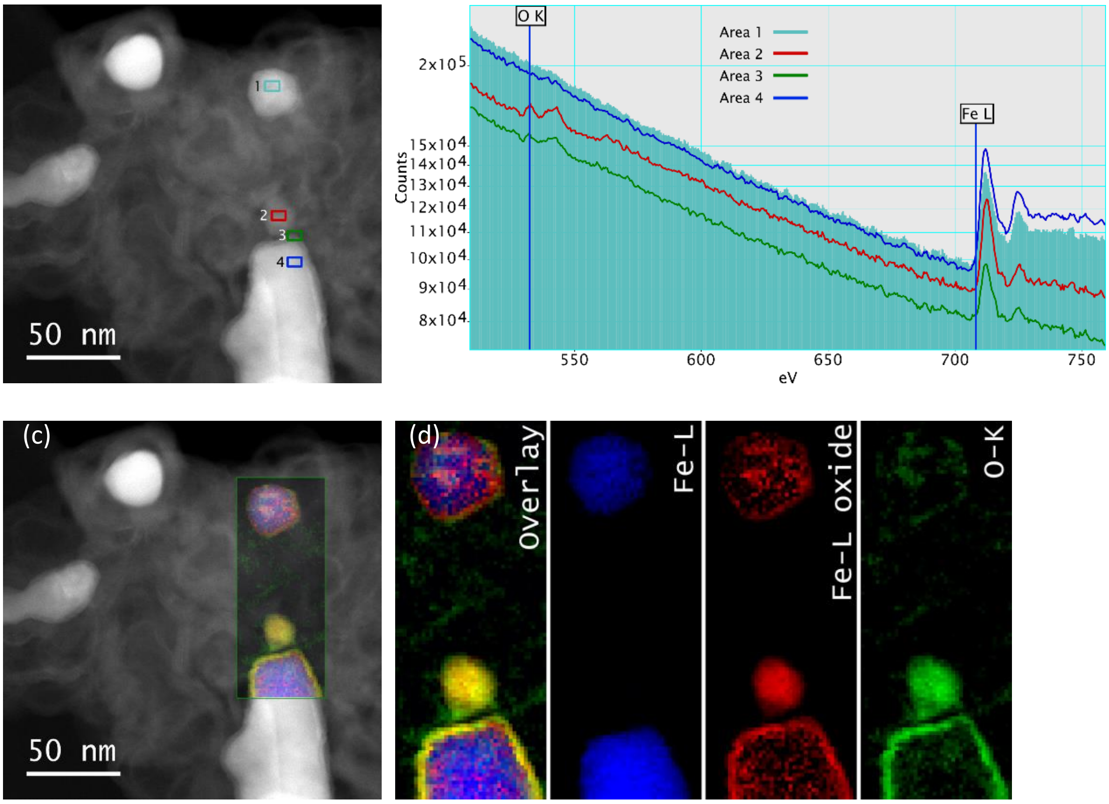

Supplementary Fig. 8 STEM-ADF image (a) with marked areas used for extraction of the EELS spectra (b) of the sample used for 48 h in reaction 1. ADF image (c) with marked area for elemental mapping (d).

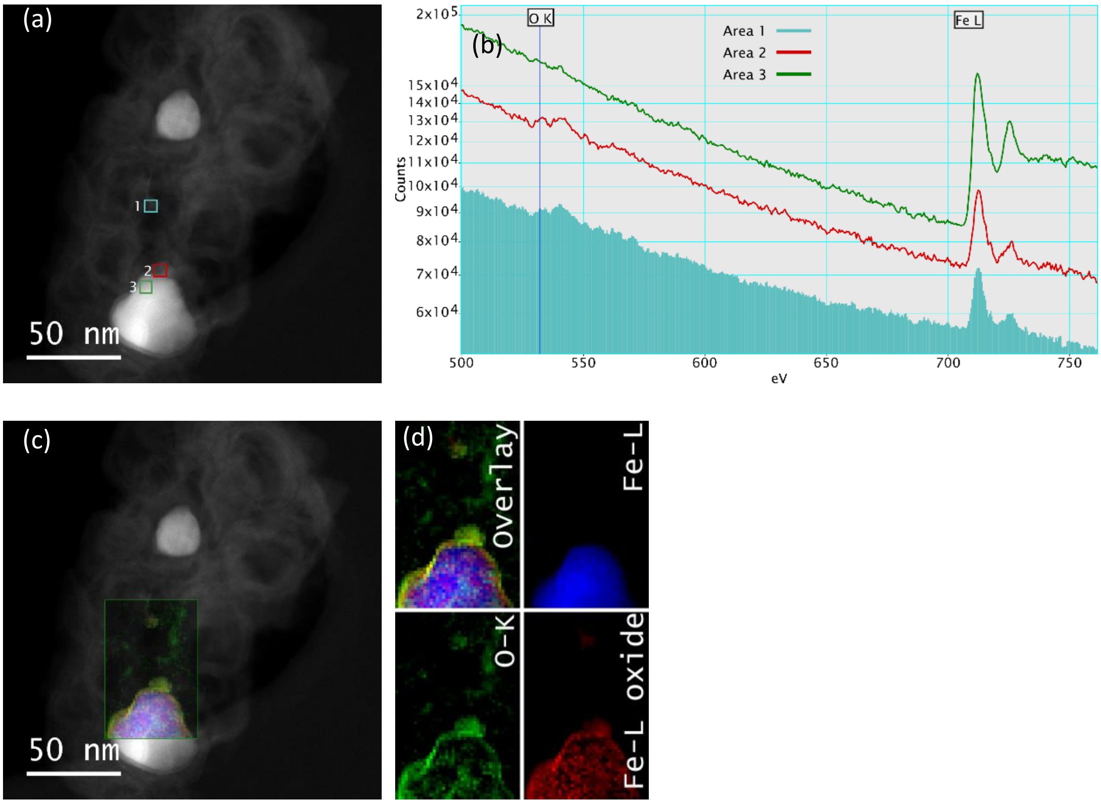

Supplementary Fig. 9 STEM-ADF image (a) with marked areas used for extraction of the EELS spectra (b) of the sample used for 72 h in reaction 2. ADF image (c) with marked area for elemental mapping (d).

To obtain additional information on the local structure of Fe XANES and EXAFS measurements have been performed on the Fe K absorption edge (Supplementary Fig. 10). The main edge of the XANES spectra corresponds to a 1s to 4p electron excitation. The shoulder feature at around 7112 eV is due to Fe-Fe 3d-4p hybridization. The first maximum in the first-derivative of the XANES spectrum (inset in Supplementary Fig. 10a) was applied to determine the edge energy. The fresh Fe-Cellulose-1000 catalyst and the Fe foil reference show the same edge energy (7112.0 eV) confirming that the fresh catalyst contains zerovalent Fe. Compared with Fe foil, the maximum absorption peak of the catalyst is broader and very similar to that reported for theta-Fe_3C standards[9]. For EXAFS fitting, both theta-Fe_3C and Fe foil structures[10] were used as modelling structures (Supplementary Fig. 10b, c and d and Supplementary Table 4). The estimated ratio of Fe:Fe_3C from EXAFS fitting is around 15:85. The Fe-Fe bond distances were shrinked compared with the Fe-Fe bond distances in reported theta-Fe_3C and Fe foil crystal structures[11]. These results clearly confirm those of PXRD and XPS described above.

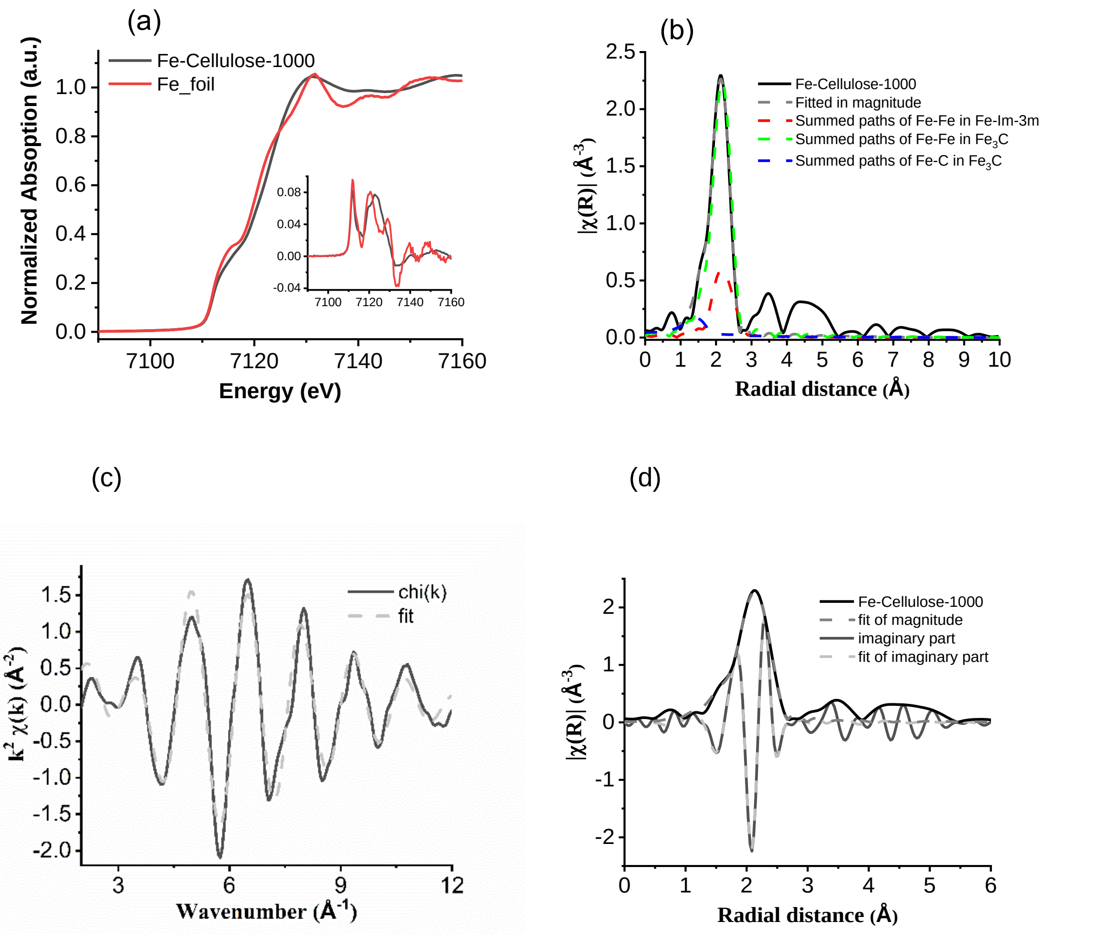

Supplementary Fig. 10 (a) XANES spectra of Fe-Cellulose-1000 and Fe foil (inset: 1st

derivative); (b) R-space EXAFS spectra of Fe-Cellulose-1000 and fitting (dashed line), summed fitted paths of Fe-Fe from Fe_Im-3m (red), Fe-Fe from theta-Fe_3C (green) and Fe-C from theta-Fe_3C (in blue). (c) k-space EXAFS data and fitting. (d) R-space EXAFS and fitting.

Supplementary Table 4. Fitting results of the fresh Fe-Cellulose-1000 catalyst based on theta-Fe_3C and Fe_Im-3m crystal structure model

Crystal Model	Path	Ratio	CN	Amp	Debye-Waller factor	Enot	R-fitted	R in crystal model
Theta-Fe_3C	Fe-C	0.85	0.7	0.684	0.01182	4.153	1.9512	2.0071
Theta-Fe_3C	Fe-C	0.85	0.7	0.684	0.01198	4.153	1.9861	2.0430
Theta-Fe_3C	Fe-C	0.85	0.7	0.684	0.01336	4.153	2.3277	2.3944
Theta-Fe_3C	Fe-Fe	0.85	0.7	0.684	0.00468	4.153	2.3736	2.4416
Theta-Fe_3C	Fe-Fe	0.85	2.7	0.684	0.00485	4.153	2.4800	2.5511
Theta-Fe_3C	Fe-Fe	0.85	0.7	0.684	0.00488	4.153	2.5019	2.5736
Theta-Fe_3C	Fe-Fe	0.85	2.7	0.684	0.00498	4.153	2.5738	2.6475
Theta-Fe_3C	Fe-Fe	0.85	0.7	0.684	0.00506	4.153	2.6297	2.7050
Theta-Fe_3C	Fe-C	0.85	0.7	0.684	0.01172	4.153	1.9273	1.9825
Theta-Fe_3C	Fe-Fe	0.85	1.3	0.684	0.00488	4.153	2.4940	2.5655
Theta-Fe_3C	Fe-Fe	0.85	1.3	0.684	0.00498	4.153	2.5634	2.6368
Theta-Fe_3C	Fe-Fe	0.85	1.3	0.684	0.00505	4.153	2.6172	2.6922
Fe_Im3m	Fe-Fe	0.15	8.0	0.684	0.00423	4.153	2.4368	2.4838
Fe_Im3m	Fe-Fe	0.15	6.0	0.684	0.00483	4.153	2.8137	2.8680

(CN: coordination number was set as calculated value from the crystal structure. Ratio: a fraction of how much Fe_Im-3m and how much Fe_3C were mixed was used as guessing parameter with the value between 0 and 1. Debye-Waller factor: a Debye function was used to define the disorder level. Enot: energy shift was one guessing parameter set as the same between different paths. R: Bond distances were fitted by guessing two coefficients times the origin bond distances in theta-Fe_3C and Fe_Im-3m, respectively, to measure the lattice expansion or contraction).

Conclusions on the catalyst structure

Based on the presented characterization data it can be concluded that the freshly pyrolyzed catalyst consists of Fe/Fe_3C particles of 20-50 nm size which are covered by a multiple graphene layer. The latter is removed during the early stages of the catalytic reaction, thus enabling contact the contact of the Fe surface with reactants. During reaction, Fe_3C is partly converted to metallic Fe which is considered as active phase in the reaction.

7. Mechanistic studies

7.1. Kinetic isotope effect investigations

All the experiments below used the same batch of iron catalyst and D_2O from the same bottle. We performed kinetic studies with aniline as substrate because of its good solubility in water. We found that the reaction order with respect to aniline is a first-order reaction (see below Supplementary Fig. 12).

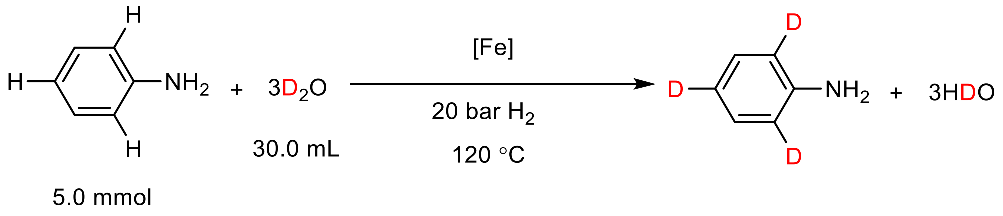

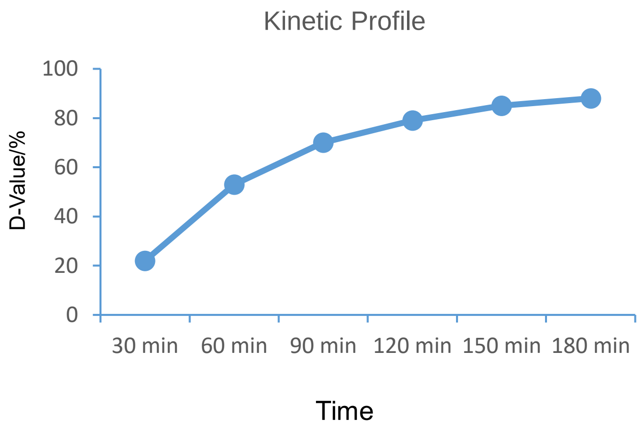

Supplementary Fig. 11 Kinetic profile

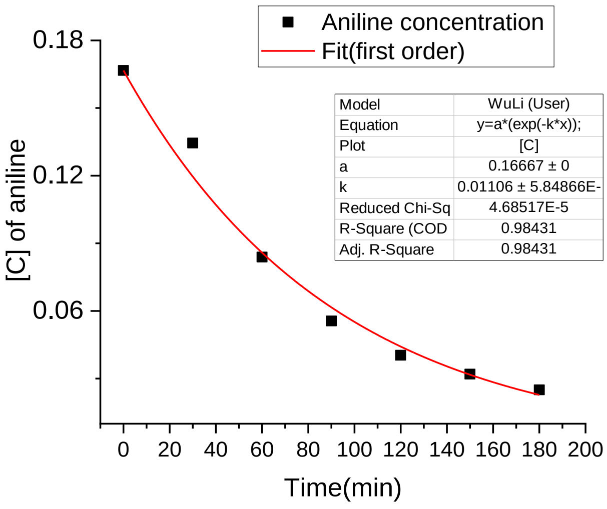

Supplementary Fig. 12 First-order reaction

In a 50 mL steel Parr autoclave (see below) loaded with Fe-Cellulose-1000 catalyst (300 mg, 0.21 mmol), aniline (465 mg, 5.0 mmol) and deuterium oxide 30.0 mL (1650 mmol) were added. The autoclave was flushed with hydrogen 6 times at 10 bar and finally pressurized to the desired value (20 bar). Then, it was it placed into an equipment for the autoclave and heated to 120 °C (about 10 min). The first sample was taken 20 min after heating. Then, the other 5 samples were taken in each 30 min. The 6 samples were measured directly in D_2O by ^1H NMR. The D-incorporation is determined by ^1H NMR.

Comparison experiments (both reactions are performed under identical conditions)

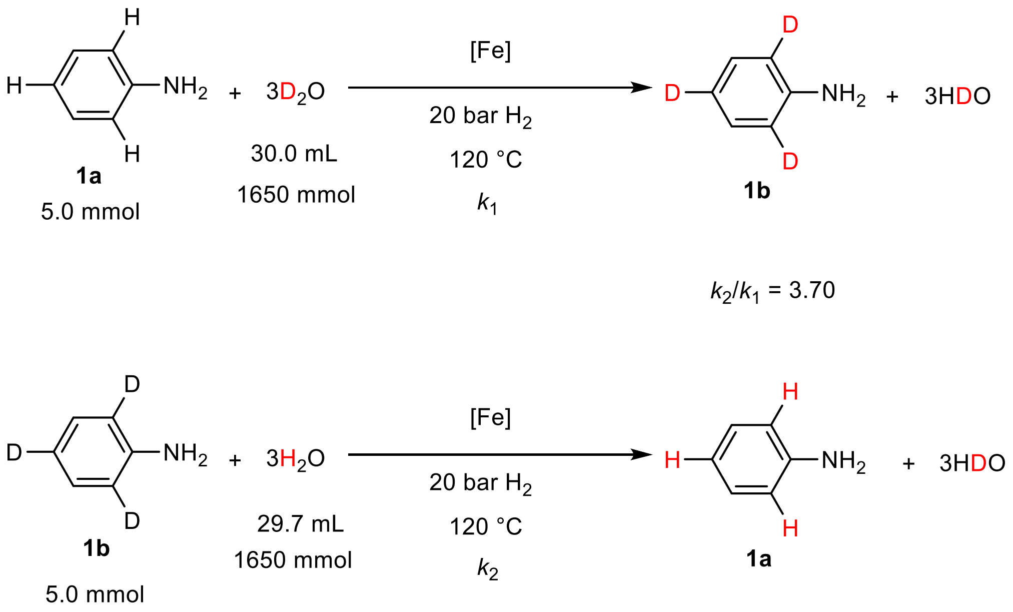

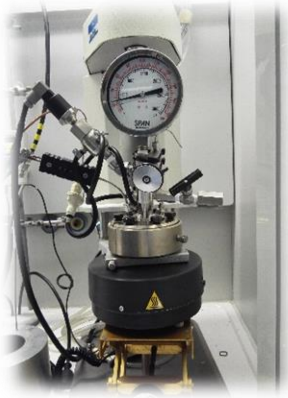

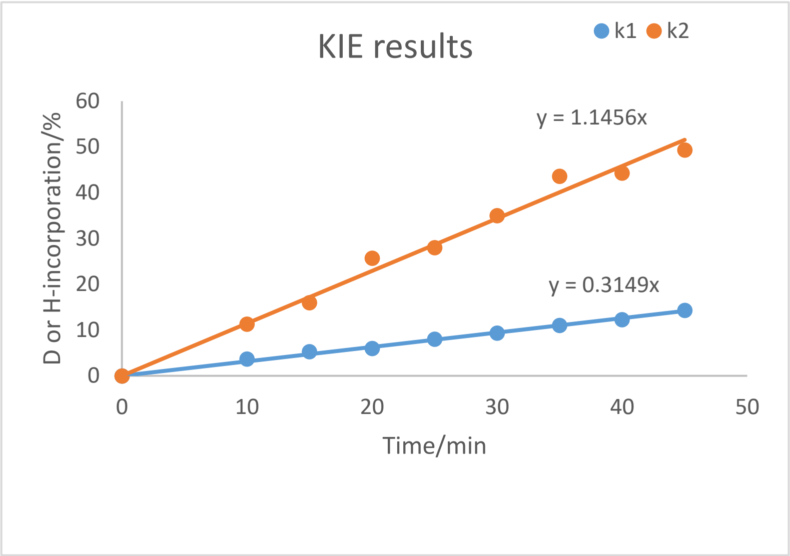

Supplementary Fig. 13 Comparison experiments

^{1}H NMR for the substrate of aniline:

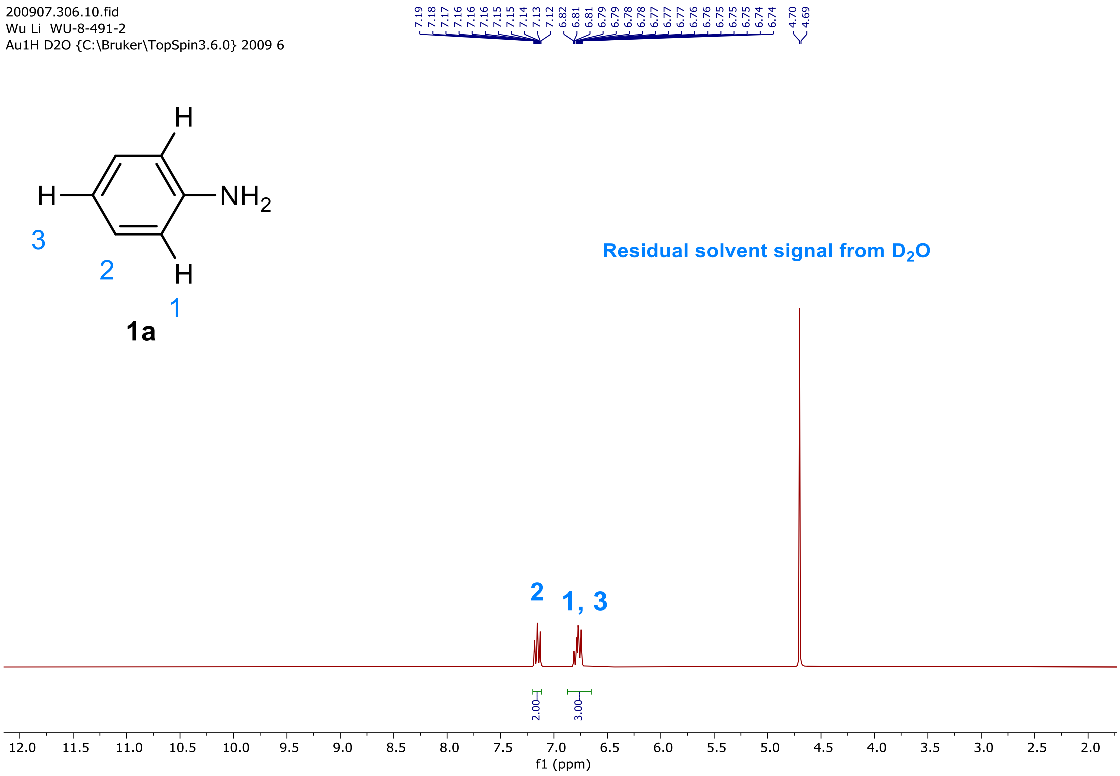

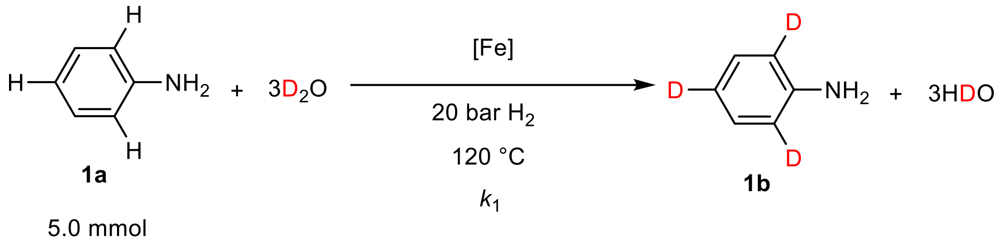

In a 50 mL steel Parr autoclave loaded with Fe-Cellulose-1000 catalyst (300 mg, 0.21 mmol), aniline (465 mg, 5.0 mmol) and deuterium oxide 30.0 mL (1650 mmol) were added. The autoclave was flushed with hydrogen 6 times at 10 bar and finally pressurized to the desired value (20 bar). Then, it was it placed into an equipment for the autoclave and heated to 120 °C (about 10 min). The first sample was taken 10 min after heating. Then, the other 7 samples were taken in each 5 min. The 8 samples were measured directly in D_2O by ^1H NMR (see below).

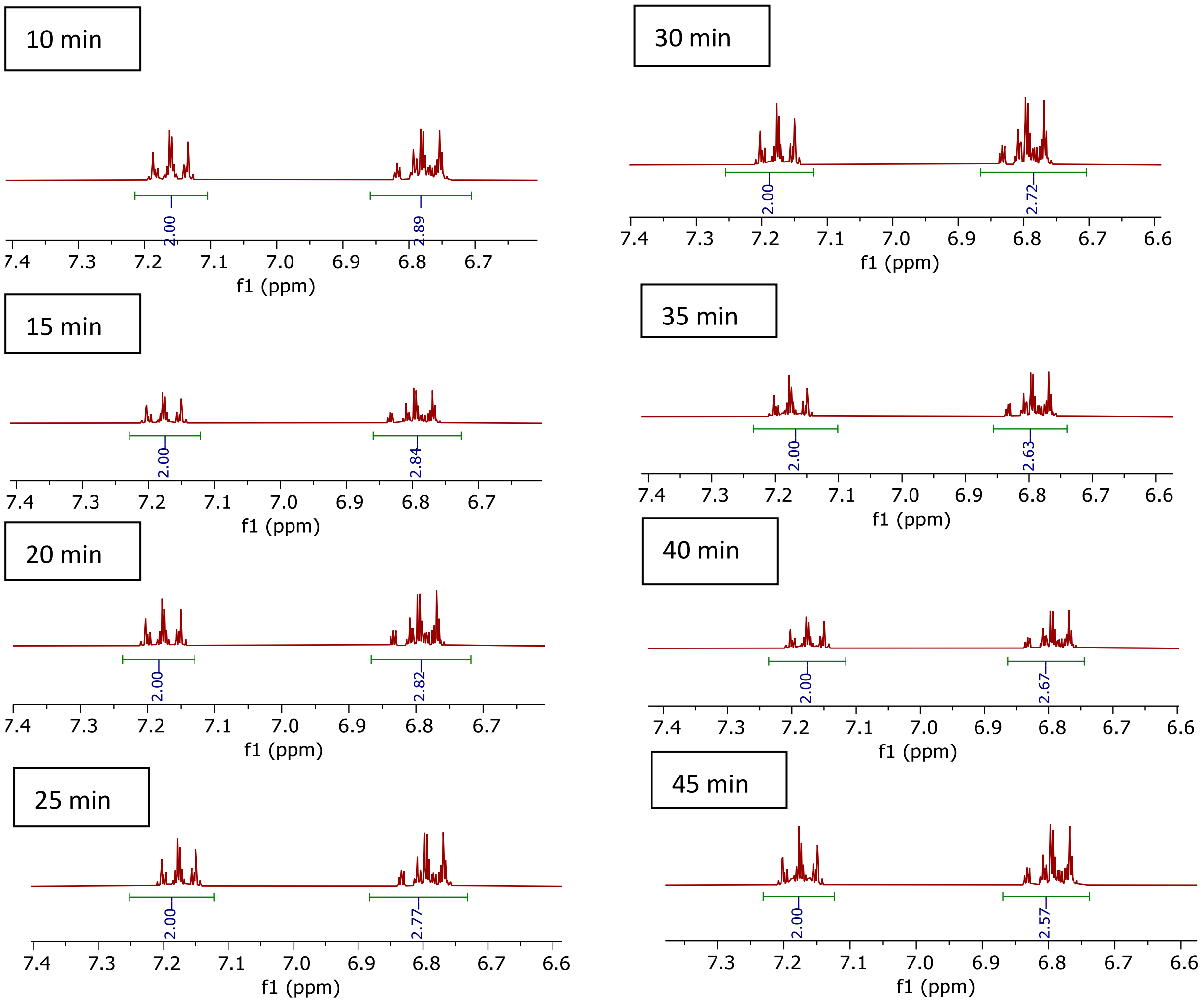

^{1}H NMR for aniline-2, 4, 6-*d*3:

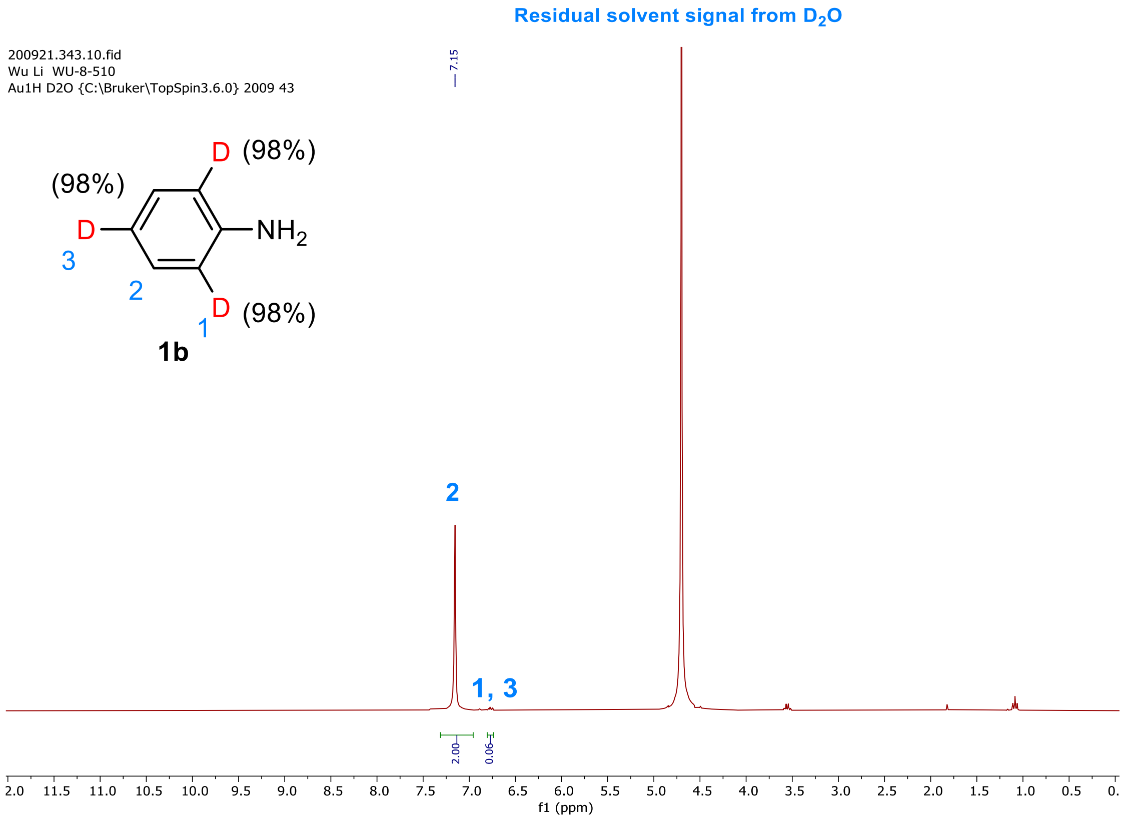

HRMS (ESI-TOF) for aniline-2, 4, 6-*d*3:

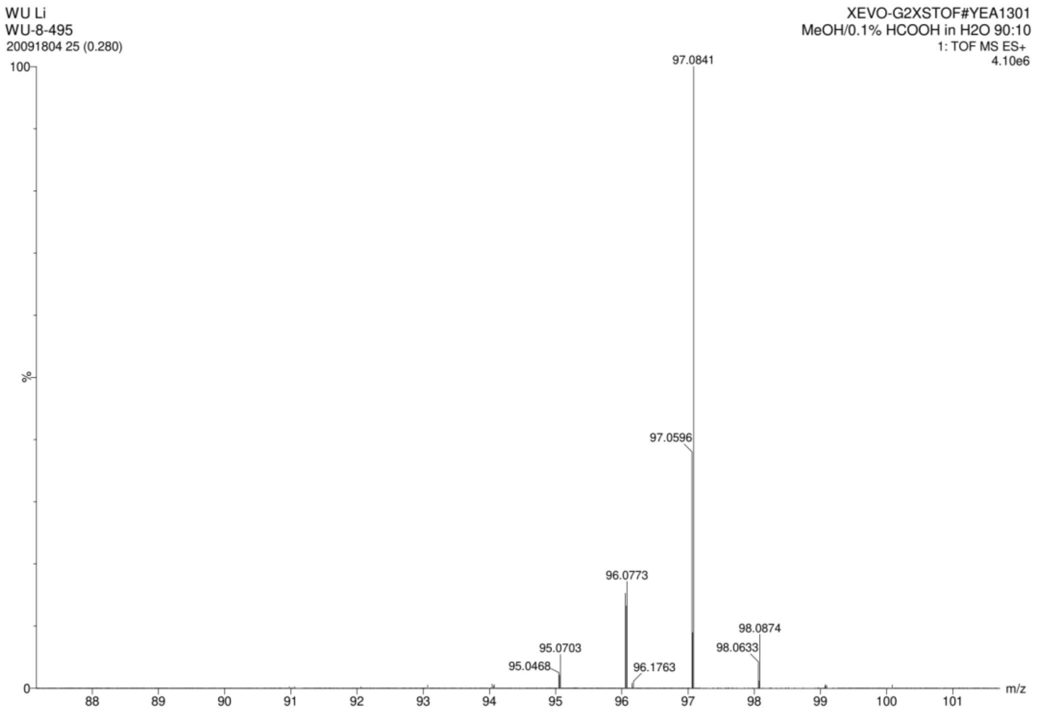

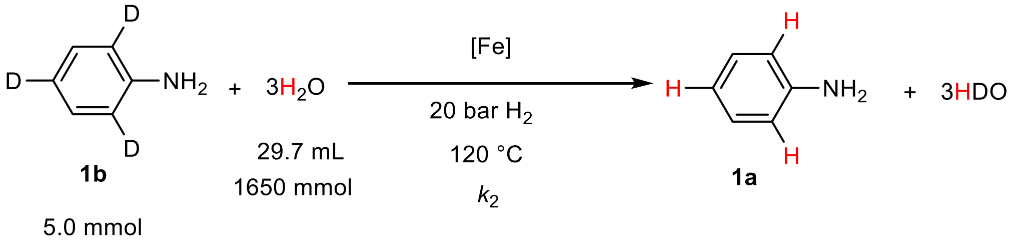

In a 50 mL steel Parr autoclave loaded with Fe-Cellulose-1000 catalyst (300 mg, 0.21 mmol), aniline-2,4,6-*d*3 (480 mg, 5.0 mmol) and H_2O 29.7 mL (1650 mmol) were added. The autoclave was flushed with hydrogen 6 times at 10 bar and finally pressurized to the desired value (20 bar). Then, it was it placed into an equipment for the autoclave and heated to 120 °C (about 10 min). The first sample was taken after 10 min from heating. Then, the other 7 samples were taken in each 5 min. The 8 samples were measured after adding 0.5 mL D_2O in by ^{1}H NMR (see below).

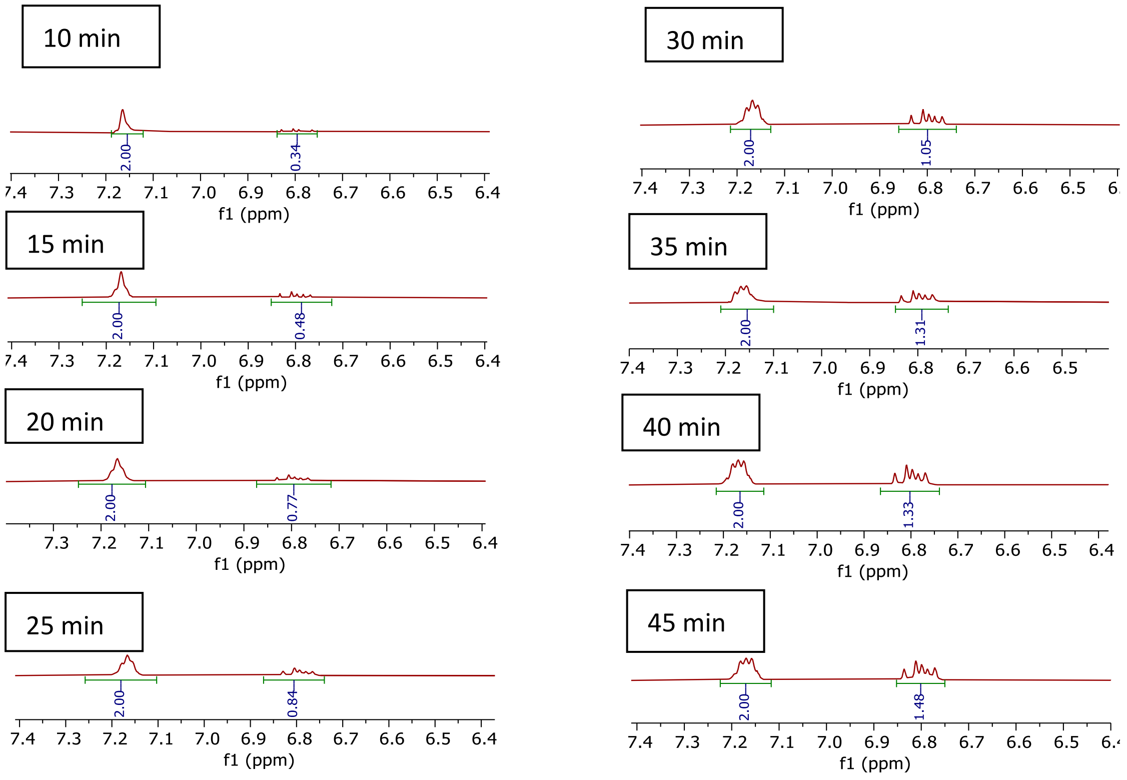

Secondary kinetic isotope effects (both reactions are performed under identical conditions)

Evaluation of the kinetic isotope effect was also performed in vials following a modified version of the deuteration procedure:
In a 4 mL vial fitted with magnetic stirring bar and septum cap, iron catalyst (30 mg, 10 mol% Fe) and aniline or aniline-3, 5-*d*2 (0.25 mmol) were added. Then, a needle was inserted in the septum which allows gaseous reagents to enter. After adding the solvent deuterium oxide (1.5 mL), the vials were set in an alloy plate and then placed into a 300 mL steel Parr autoclave. The autoclave was flushed with hydrogen 6 times at 10 bar and finally pressurized to the desired value (20 bar). Then, it was placed into an aluminum block and heated to 120 °C. After 5 hours, the autoclave was quickly cooled down to room temperature with an ice bath and vented. Finally, the samples were removed from the autoclave, the heterogeneous catalyst was filtered, and the reaction mixtures were subject to NMR analyses to determine the deuterium content.

This procedure was followed twice with aniline and aniline-3, 5-*d*2 in the same autoclave to ensure the reproducibility of the results.
With aniline (10 mol% Fe, 5 h), [96]D and repeat one [96]D
With aniline-3, 5-*d*2 (10 mol% Fe, 5 h), [64]D and repeat one [71]D
A secondary KIE of 1.42 if we take the average of the two last values.

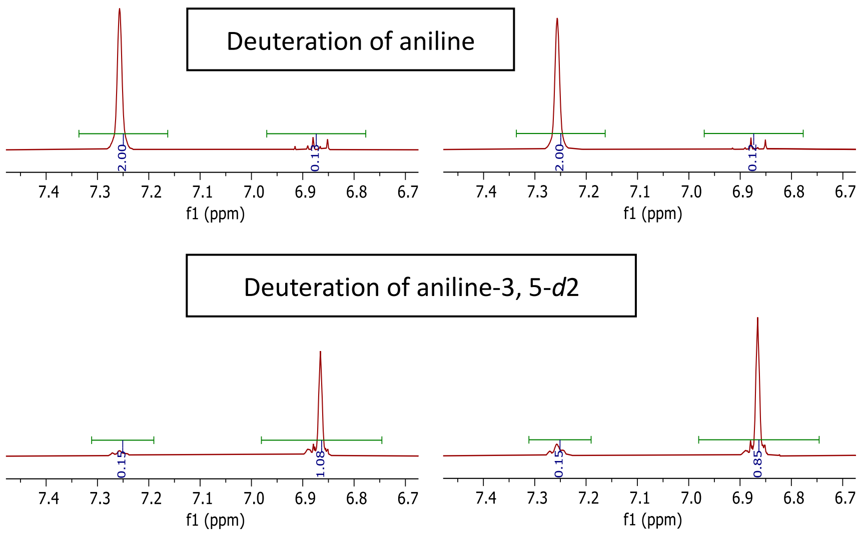

Supplementary Fig. 14 Secondary kinetic isotope effect

7.2. EPR studies

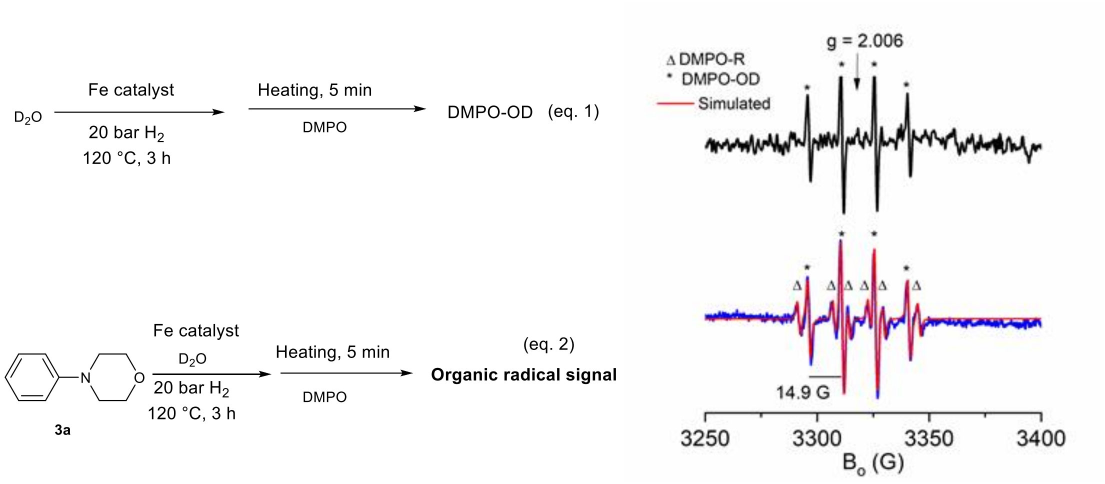

Supplementary Fig. 15 EPR spin trapping studies with DMPO

In a 4 mL vial fitted with magnetic stirring bar and septum cap, iron catalyst (40 mg) was added. A needle was inserted in the septum which allows gaseous reagents to enter. After adding the solvent deuterium oxide (1.5 mL), the vial was set in an alloy plate and then placed into a 300 mL steel Parr autoclave. The autoclave was flushed with hydrogen 6 times at 10 bar and finally pressurized to the desired value (20 bar). Then, it was placed into an aluminium block and heated to the desired temperature (120 °C) and at 120 °C for 3 h. At the end of the reaction, the autoclave was quickly cooled down to room temperature with an ice bath and vented. Finally, the sample was removed from the autoclave immediately and sealed under argon. The sample was heated again at 140 °C for 5 min and 15 µl of 5,5-Dimethyl-1-pyrroline-*N*-oxide (DMPO) was added (Supplementary eq. 1). Then a glass microcapillary tube (Hirschmann) was filled under N_2 with about 50 µL of the reaction mixture and sealed with capillary sealing wax. EPR spectra were immediately recorded at room temperature. Spectral simulation was performed using the Easyspin program[11].

A similar experiment was performed in the presence of 4-phenylmorpholine (40 mg) according to Supplementary eq. 2. The EPR spectrum of the reaction mixture described in eq. S1 exhibited a four-lines signal with approximately 1:2:2:1 intensity at g = 2.006 with A_N = 15.05 and A_H = 14.85 G due to the formation of DMPO-OD spin adduct indicating the formation of *OD radical. In the presence of 4-phenylmorpholine (Supplementary eq. 2), a new six-lines signal appeared at g = 2.006 with A_N = 15.71 and A_H = 22.24 G due to the formation of DMPO-R spin adducts[12] indicating the in situ formation of *R radicals during the catalytic reaction and supporting the proposed reaction mechanism (Fig. 2d in the main manuscript). Interestingly, no *R radicals were detected when the Fe catalyst and the 4-phenylmorpholine were heated in toluene (both the reactions in D_2O and toluene are performed under identical conditions). This indicates without doubt that formation of radical intermediates is a consequence of homolytic D-OD scission initiated by the Fe catalyst.

8. Control experiments

8.1. Control experiments without catalyst

For comparison several control experiments were performed in the absence of the iron catalyst in D_2O under H_2, 120 °C for 24 h. As shown in Supplementary Fig. 16, 4-phenylmorpholine (**3a**), 4-methoxyaniline (**4a**), 1*H*-indazol-5-amine (**44a**), 1,2,3,4-tetrahydroquinoline (**52a**) and melatonin (**67a**) gave no D-incorporation products. 3,4-Dimethylphenol (**38a**) gave very low D-incorporation without catalyst.

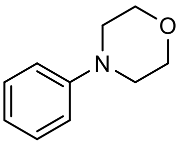

3a

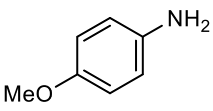

4a

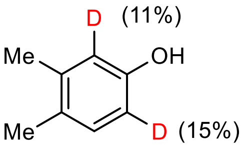

from **38a**

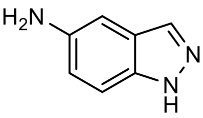

44a

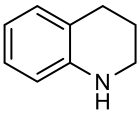

52a

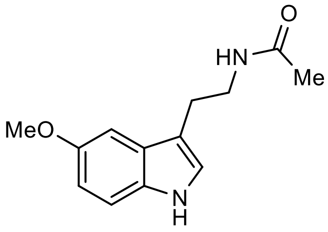

Melatonin

67a

Supplementary Fig. 16 Control experiments

8.2. Control the quality

Supplementary Table 5 Different batches of catalyst

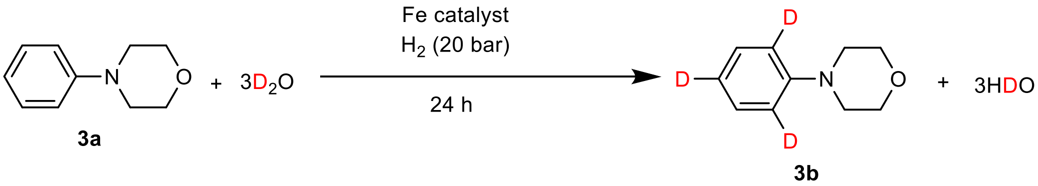

Different batches of catalyst			
Entry	*ortho*-D (%)[a]	*para*-D (%)[a]	Total-D (%)[a]
1	98.83	99.85	99.17
2	93.95	99.14	95.68
3	99.08	99.79	99.32
4	99.16	99.42	99.25
5	97.87	97.76	97.83
6	99.35	99.98	99.56

Reaction conditions: 4-phenylmorpholine (41 mg, 0.25 mmol), Fe-Cellulose-1000 (61 mg, 20 mol% Fe), D_2O (3.0 mL), H_2 (20 bar), 24 h.

[a]Deuterium content determined by quantitative ^1H NMR in acetone-d_6: decrease of the signal corresponding to the aromatic protons, using the morpholine signals as internal calibration.

Supplementary Table 6 The same batch of catalyst

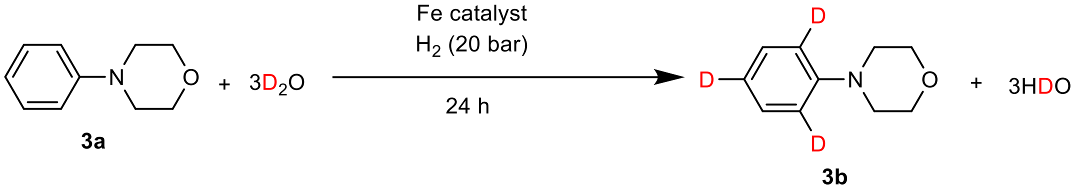

The same batch of catalyst			
Entry	*ortho*-D (%)[a]	*para*-D (%)[a]	Total-D (%)[a]
1	99.11	99.60	99.27
2	97.87	97.76	97.83
3	98.93	99.54	99.13
4	98.74	99.67	99.05

Reaction conditions: 4-phenylmorpholine (41 mg, 0.25 mmol), Fe-Cellulose-1000 (61 mg, 20 mol% Fe), D_2O (3.0 mL), H_2 (20 bar), 24 h.

[a]Deuterium content determined by quantitative ^1H NMR in acetone-d_6: decrease of the signal corresponding to the aromatic protons, using the morpholine signals as internal calibration.

8.3. Dehalogenation measurements

Here, we provide the LC-MS results of chloro-, bromo- and iodo-containing products. As shown below, for most of the performed reactions the analysis of the crude reaction mixtures revealed no dehalogenation products at all (Supplementary Table 7, entries 3, 5-12). However, in the case of iodo-substituted anilines (Supplementary Table 7, entries 1-2) some amounts of 4-5% of deiodination were observed. Notably, in these cases the product can be relatively simple purified (because of the different physical properties).

Supplementary Table 7 Dehalogenation test

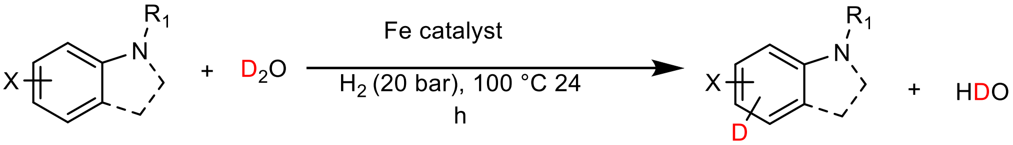

Entry	Substrate	Product	Dehalogenated product (%)
1	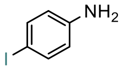 **7a**	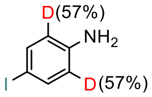 **7b**	5
2	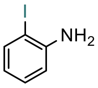 **13a**	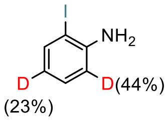 **13b**	4

3	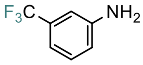 **10a**	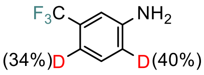 **10b**	n.d.
4	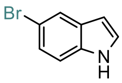 **62a**	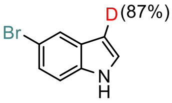 **62b**	5
5	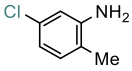 **16a**	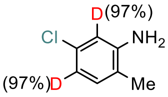 **16b**	n.d.
6	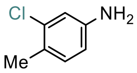 **17a**	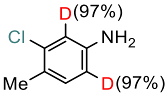 **17b**	n.d.
7	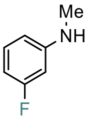 **28a**	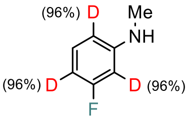 **28b**	n.d.<br>loss of Me (3%)
8	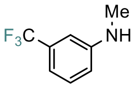 **30a**	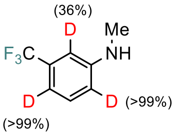 **30b**	n.d.<br>loss of Me (10%)
9	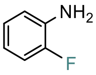 **12a**	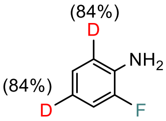 **12b**	n.d.
10	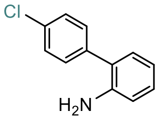 **14a**	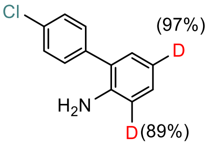 **14b**	n.d.
11	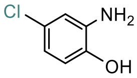 **19a**	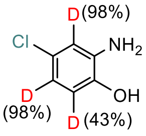 **19b**	n.d.
12	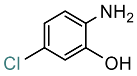 **18a**	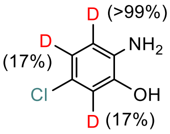 **18b**	n.d.
13	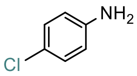 **8a**	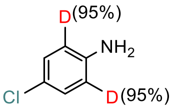 **8b**	20

n.d. = not detected

9. Scale up reactions

9.1. 20 g and 50 g scale in 300 mL autoclave

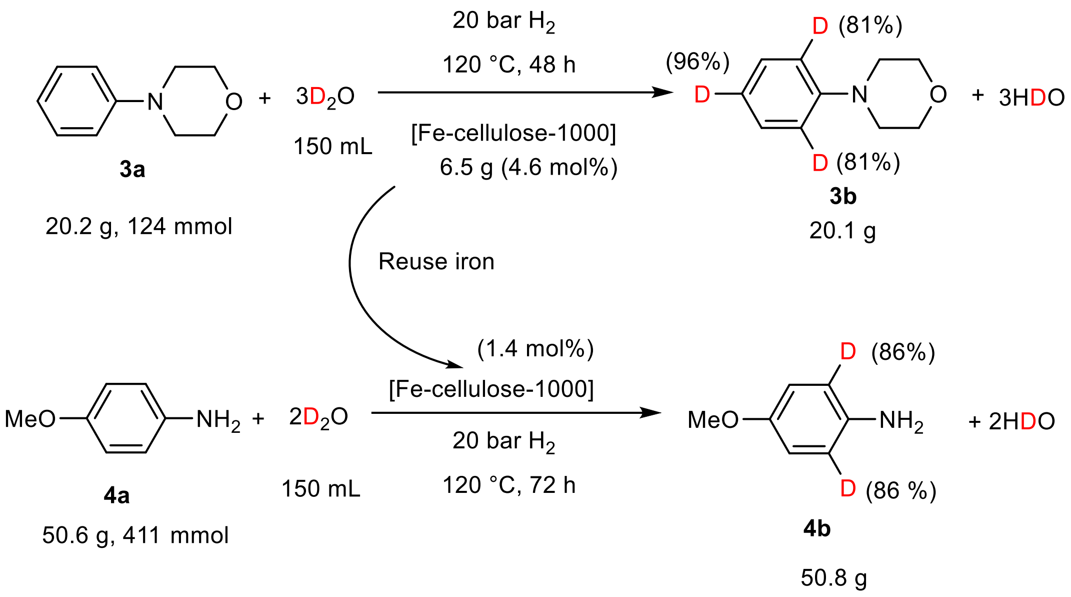

Control reaction (no deuteration):

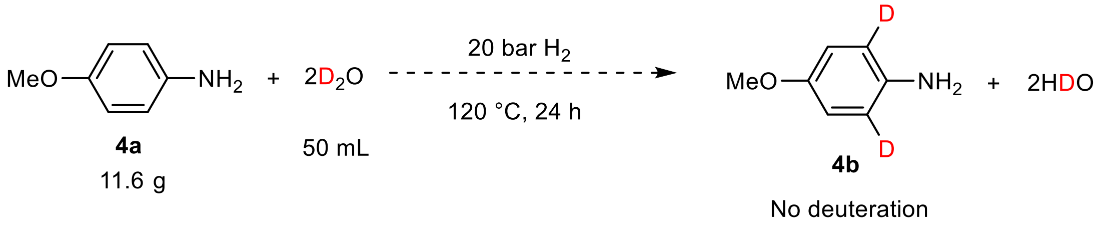

In a 300 mL steel Parr autoclave fitted with magnetic stirring bar and substrate (11.6 g, 94.3 mmol). Then, deuterium oxide (50 mL, 2750 mmol) was added. The autoclave was flushed with hydrogen 6 times at 10 bar and finally pressurized to the desired value (20 bar). Then, it was placed into an aluminum block and heated to 120 °C, and at 120 °C for 24 h. At the end of the reaction, the autoclave was quickly cooled down at room temperature with an ice bath and vented. Finally, ethyl acetate (300 mL) was added to the crude mixture. This mixture was filtered by filter paper and the water layer was removed from the mixture and washed with ethyl acetate (300 mL, 3 times). No deuterated product was observed.

20 g Scale:

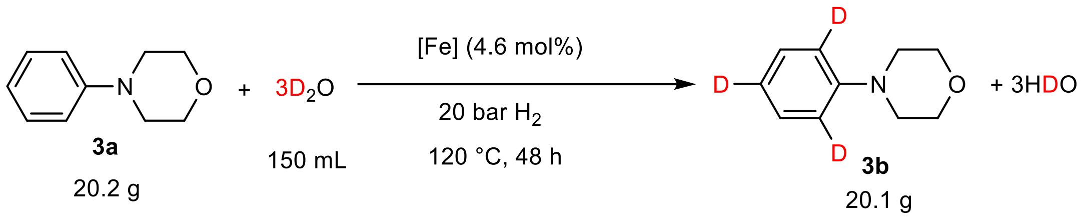

In a 300 mL steel Parr autoclave fitted with magnetic stirring bar the iron catalyst (6.5 g, 5.7 mmol) and substrate (20.2 g, 124 mmol) were added. Then, deuterium oxide (150 mL, 8250 mmol) was introduced. The autoclave was flushed with hydrogen 6 times at 10 bar and finally pressurized to the desired value (20 bar). Then, it was placed into an aluminium block and heated to the desired temperature. At the end of the reaction, the autoclave was quickly cooled down at room temperature with an ice bath and vented. Finally, ethyl acetate (400 mL) was added to the crude mixture. This mixture was filtered by filter paper. The water layer was removed from the mixture and washed with ethyl acetate (400 mL, 3 times). After removal of all volatiles from the organic mixture in vacuo, the desired product was obtained (see below for characterisation details). The catalyst was reused for the deuteration of 50.6 g *p*-anisidine (see below).

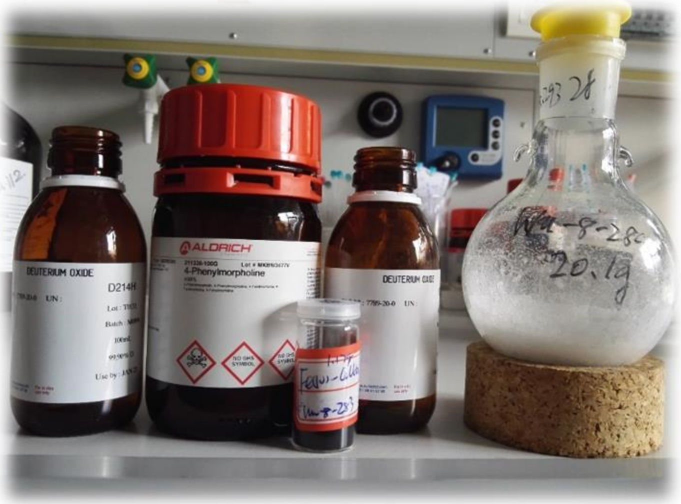

^{1}H NMR for the substrate of **3a**:

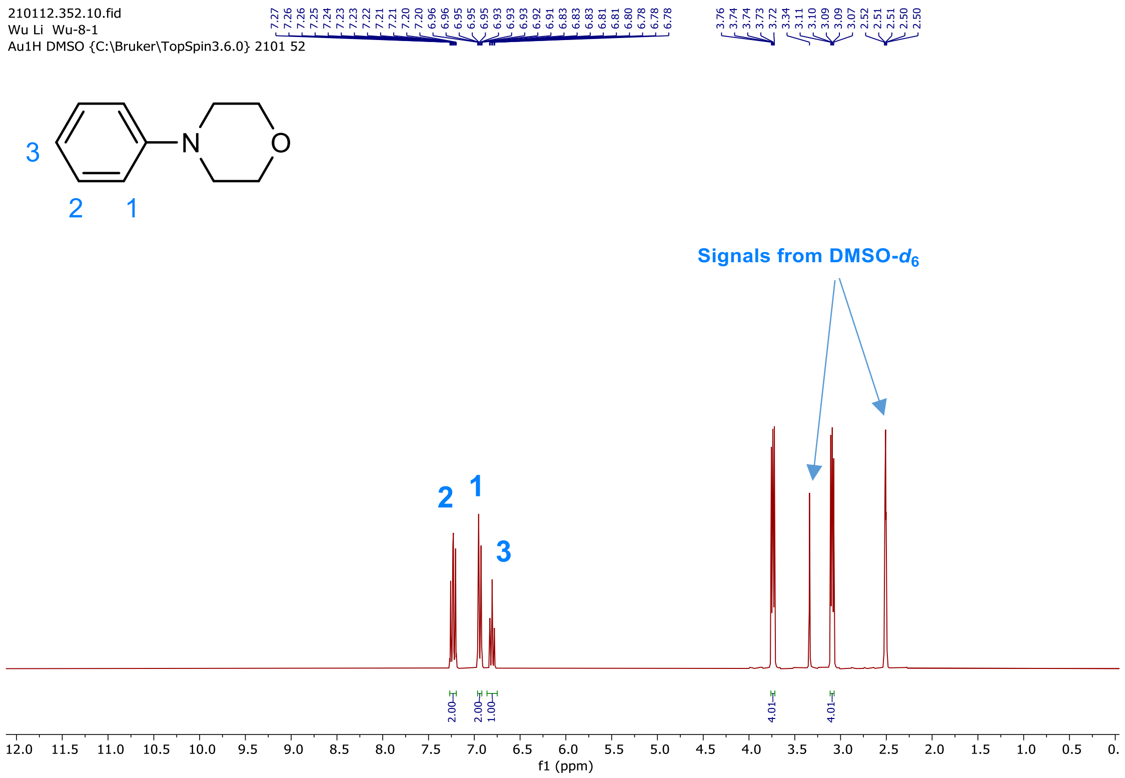

Original spectra for the product:

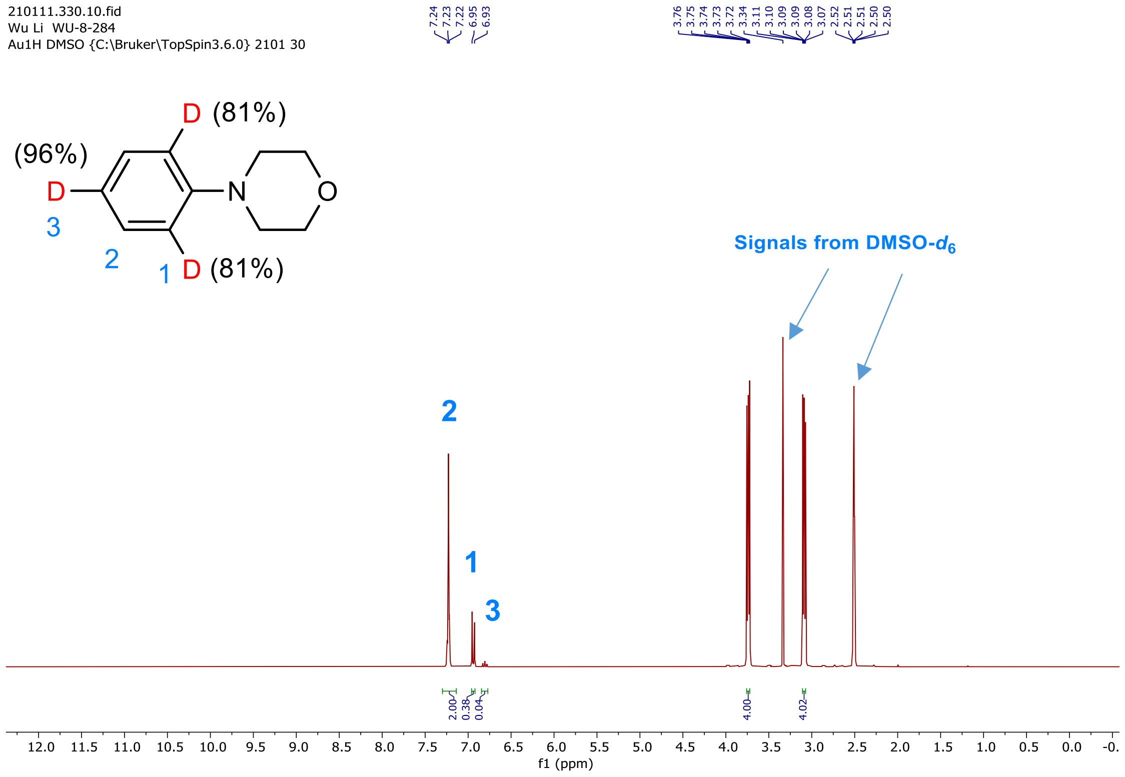

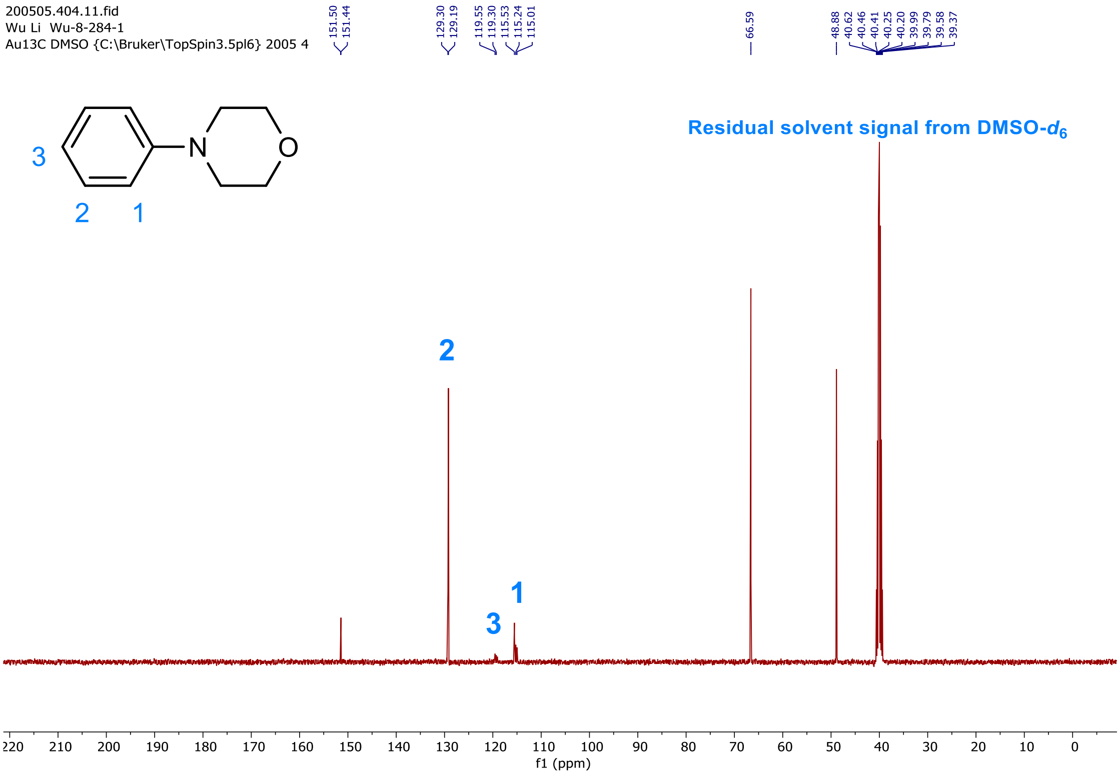

HRMS (ESI-TOF) of 3a:

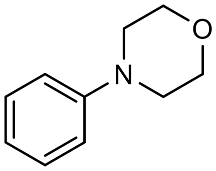

Chemical Formula: $C_{10}H_{13}NO$
Exact Mass: 163.0997

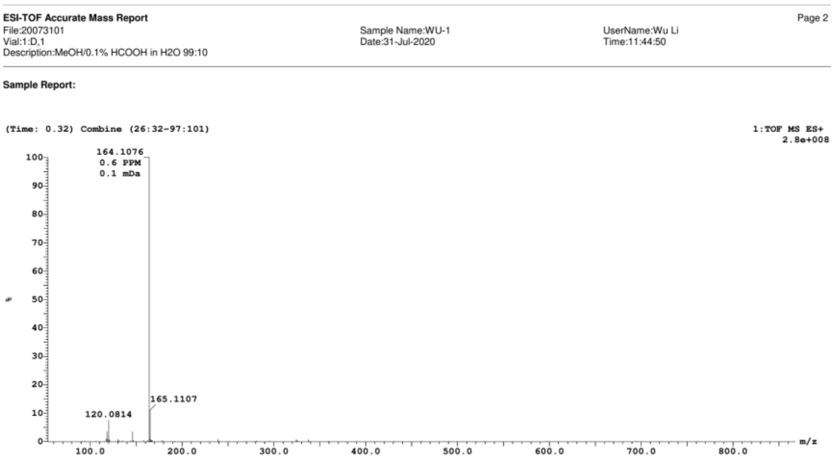

HRMS (ESI-TOF) of 3b [M+H]$^+$:

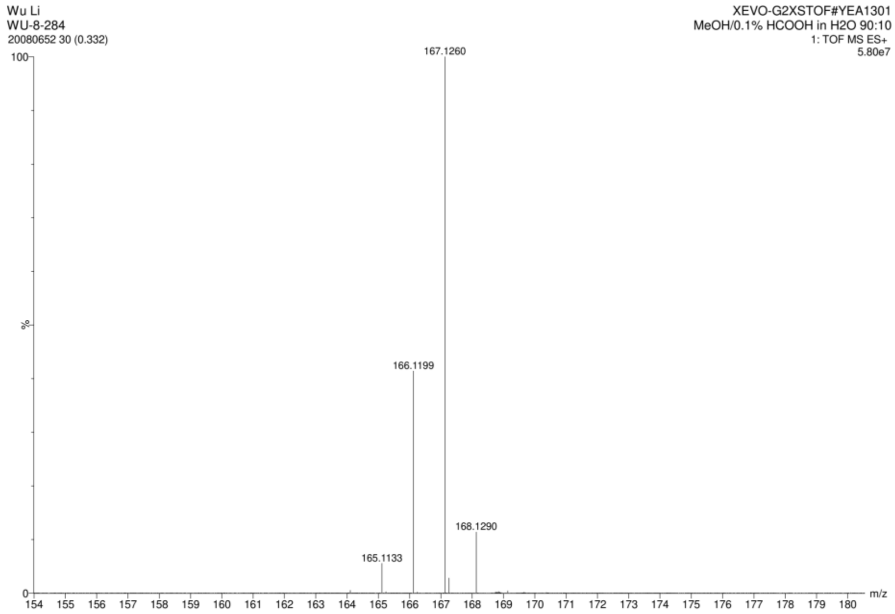

50 g Scale:

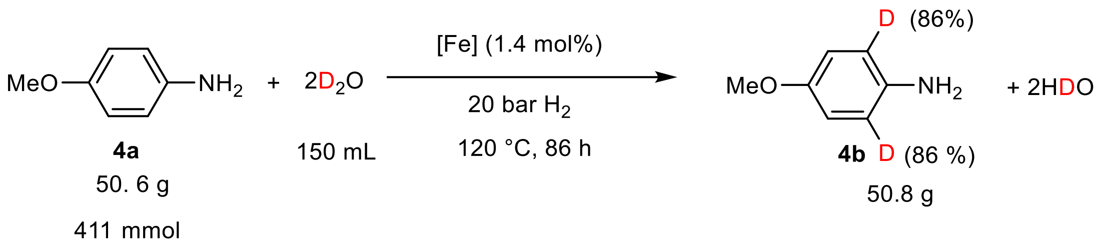

In a 300 mL steel Parr autoclave fitted with magnetic stirring bar, the iron catalyst (reuse from the catalyst above) and 50.6 g *p*-anisidine (411 mmol) were added. Then, the solvent deuterium oxide (150 mL, 8250 mmol) was added. The autoclave was flushed with hydrogen 6 times at 10 bar and finally pressurized to the desired value (20 bar). Then, it was placed into an aluminum block and heated to the desired temperature. At the end of the reaction, the autoclave was quickly cooled down at room temperature with an ice bath and vented. Finally, ethyl acetate (500 mL) was added to the crude reaction. The reaction mixture was filtered by filter paper and then 200 mL H_2O was added. The water layer was removed from the mixture and washed with ethyl acetate (500 mL, 3 times). After removal of all volatiles in vacuo, the desired product was obtained (see below for characterisation details).

Original spectra for 4a:

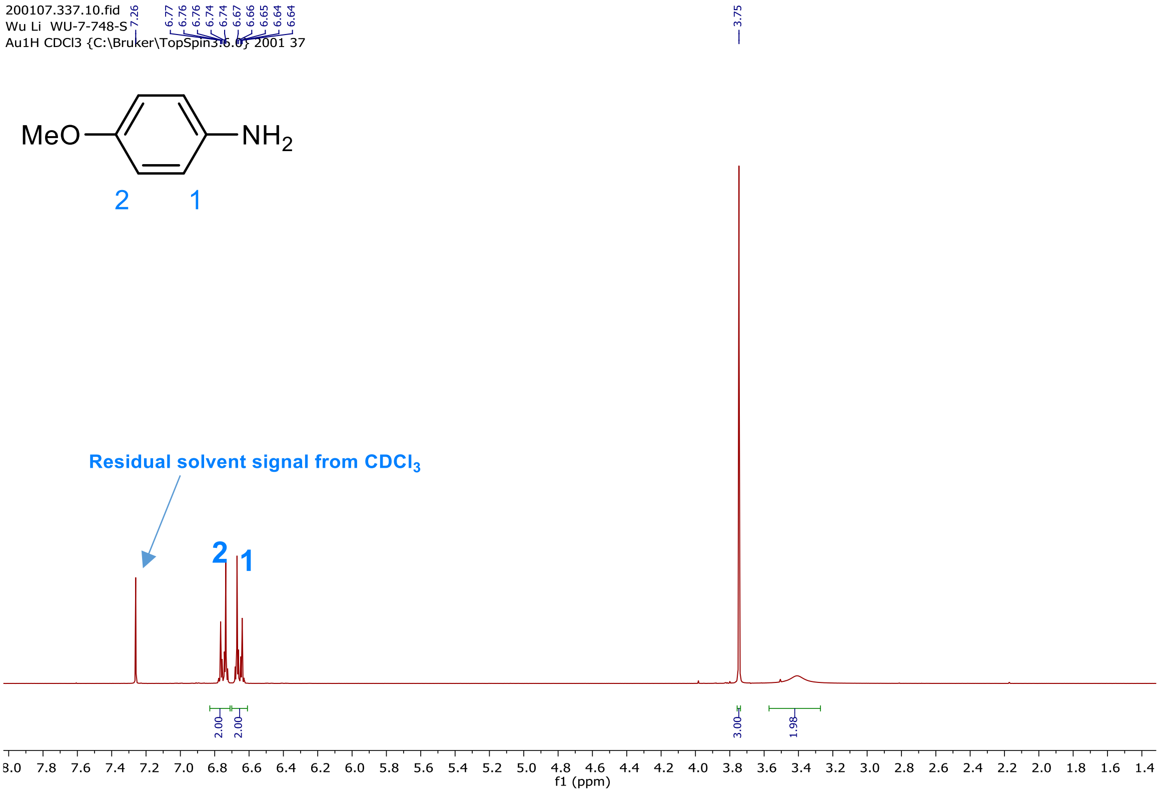

Original spectra for the product 4b:

In DMSO-d_6:

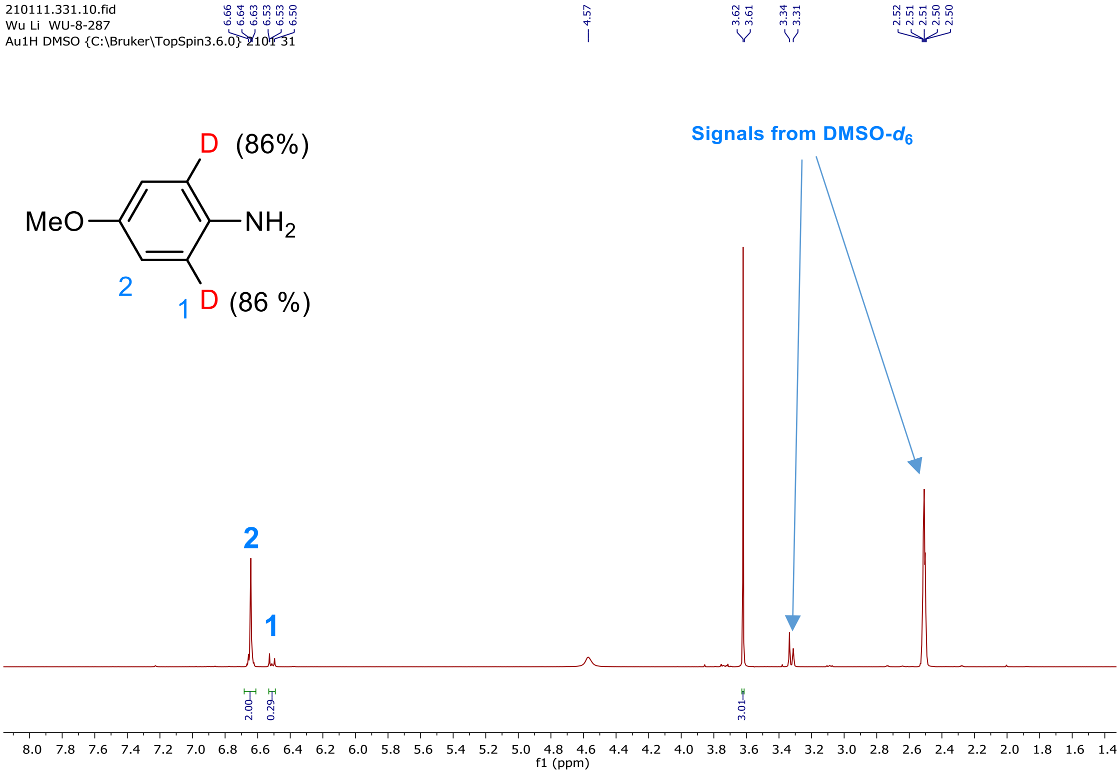

In $CDCl_3$:

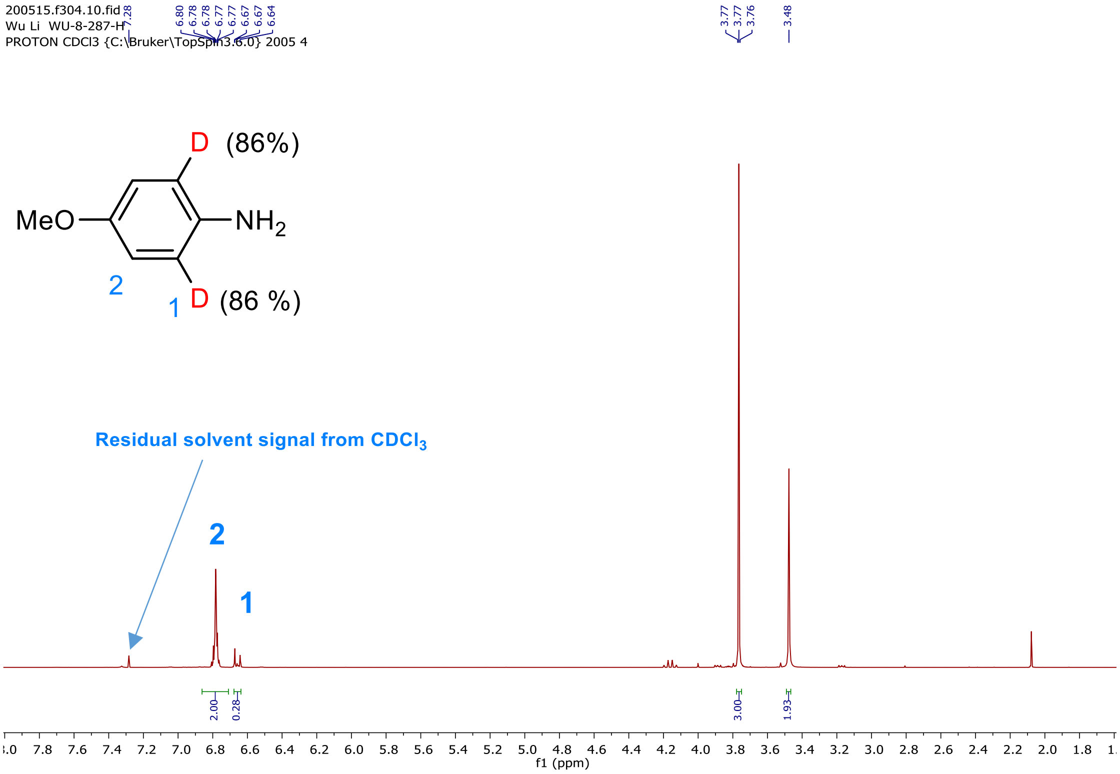

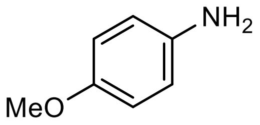

Chemical Formula: C_7H_9NO
Exact Mass: 123.0684

HRMS (ESI-TOF) of 4a:

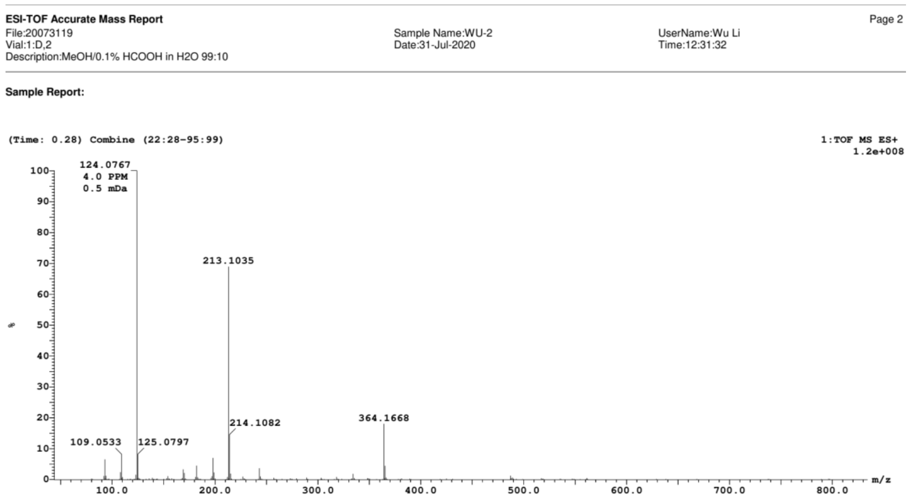

HRMS (ESI-TOF) of 4b [M+H]$^+$:

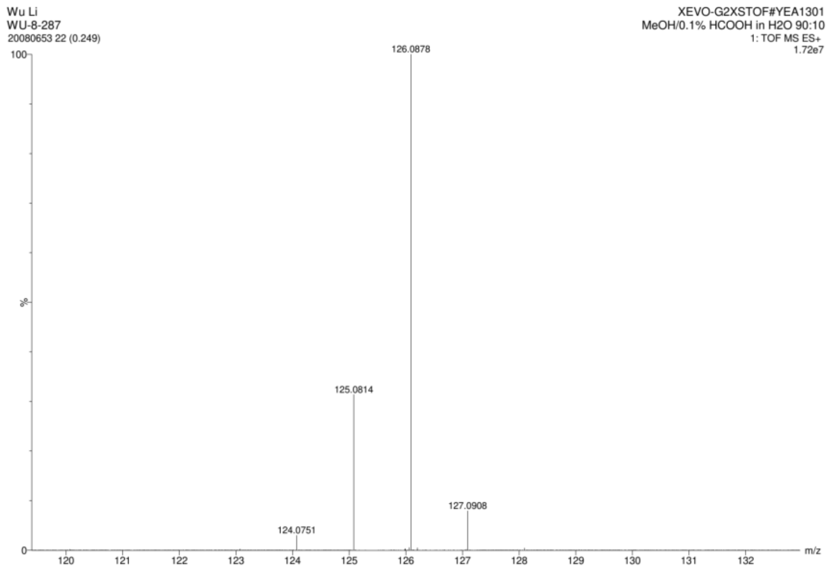

9.2. >300 g-scale reactions in 2 L autoclave

Control reaction (no deuteration):

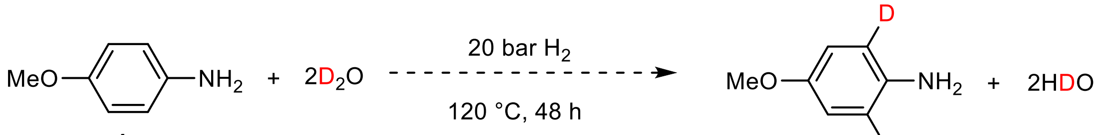

4a 50.0 g; 200 mL; **4b** No deuteration

a) Reuse of the catalyst for the preparation of 1 kg deuterated products

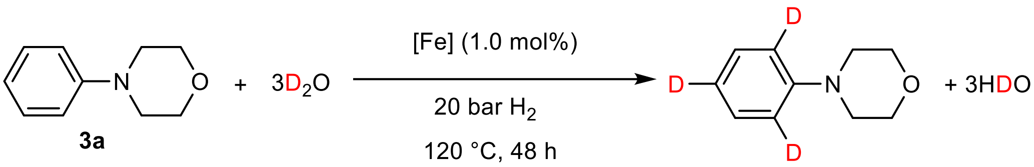

3a 2.0 mol; **3b**

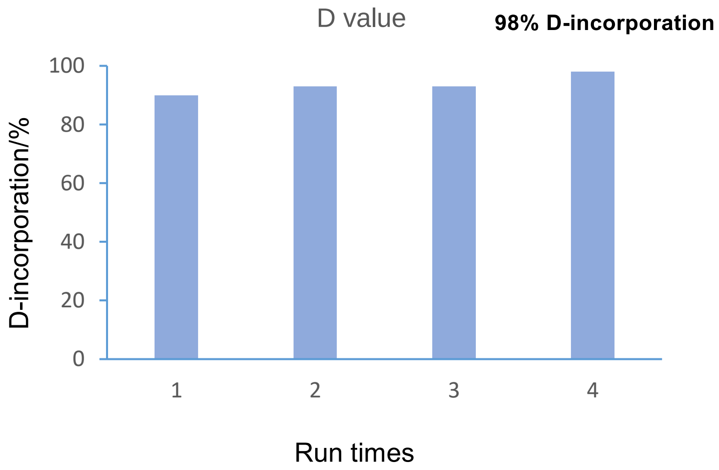

Supplementary Fig. 17 Reuse of the Fe-Cellulose-1000 catalyst for 2.0 mol scale

1. 340 g, 2.09 mol, 120 °C, 120 h. (90% D-incorporation)
2. 334 g, 2.06 mol, 140 °C, 48 h. (93% D-incorporation)
3. 331 g, 2.03 mol, 140 °C, 48 h. (93% D-incorporation)
4. 334 g from 2, 140 °C, 48 h. (98% D-incorporation, *commercial application*: 95-98% deuteration.)

Overall, 1.005 kg 4-phenylmorpholine (6.17 mol) were deuterated with 20 mmol iron catalyst.

b) Use of the recycled catalyst is possible for different substrates

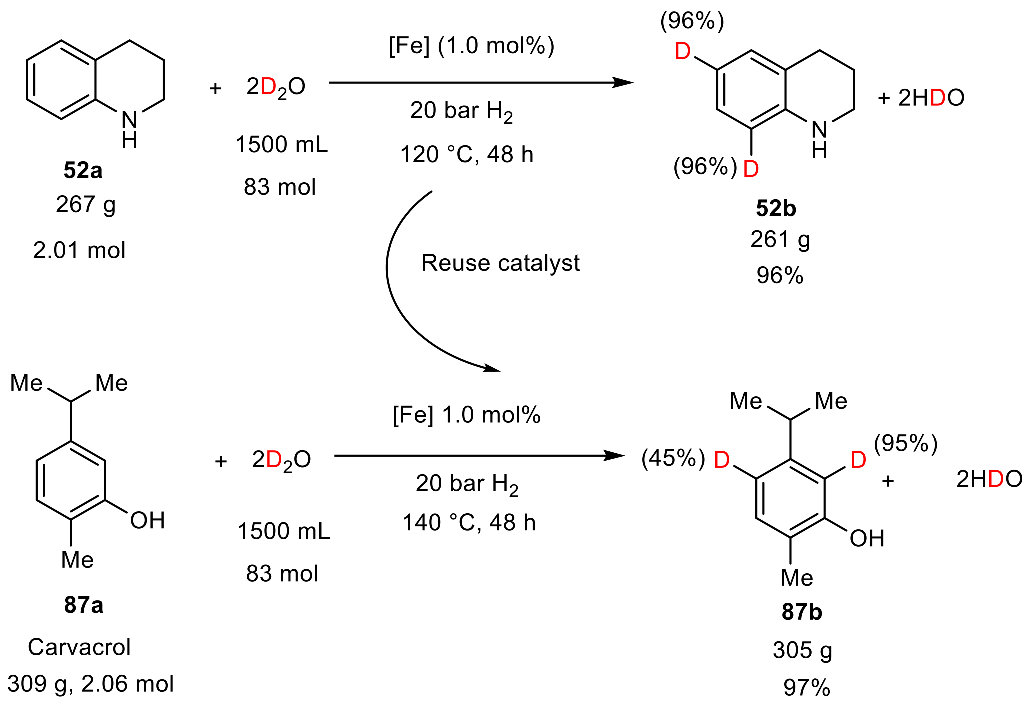

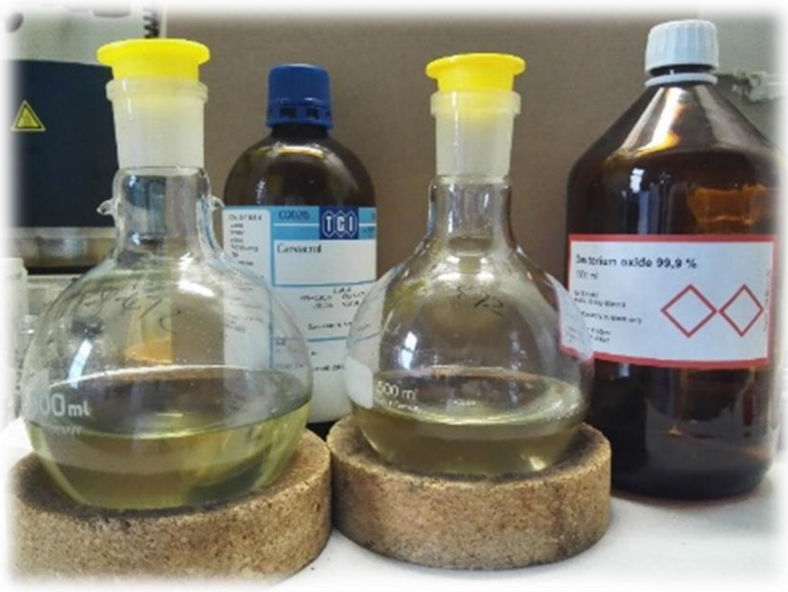

Control reaction (no deuteration):

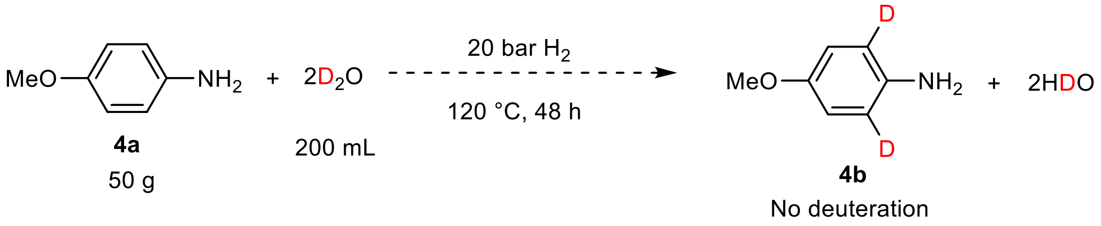

In a 2.0 L steel Parr autoclave (see below) fitted with 50.0 g *p*-anisidine and deuterium oxide 200 mL. The autoclave was flushed with hydrogen 6 times at 10 bar and finally pressurized to the desired value (20 bar). Then, it was it placed into an equipment (see below) and heated to 120 °C. At the end of the reaction, the autoclave was quickly cooled down at room temperature with an ice bath and vented. Finally, ethyl acetate (500 mL) was added to the crude reaction. The reaction mixture was filtered by filter paper. The D_2O layer was removed from the mixture and washed with ethyl acetate (500 mL, 3 times). After removal of all volatiles in vacuo, *p*-anisidine was obtained without D-incorporation.

a) Reuse the catalyst for preparation of 1 kg of 4-phenylmorpholine

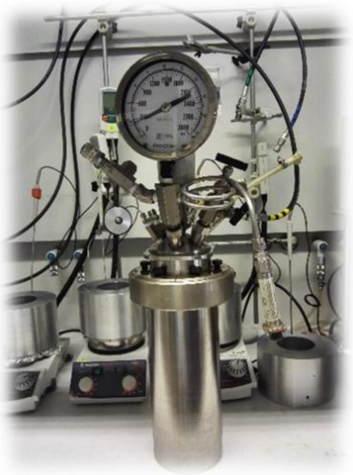

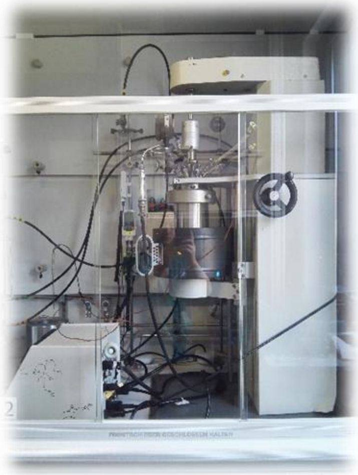

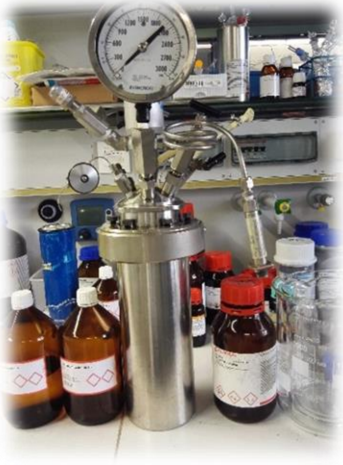

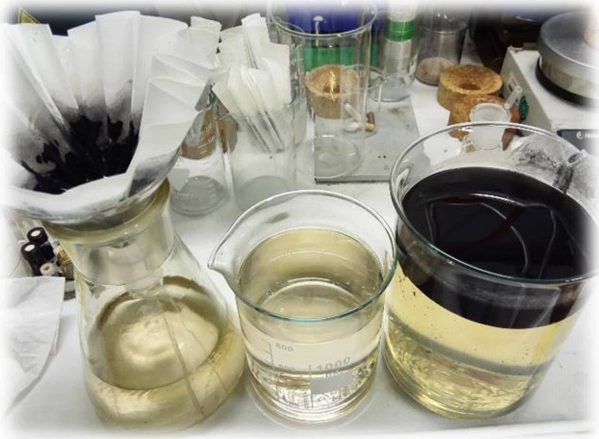

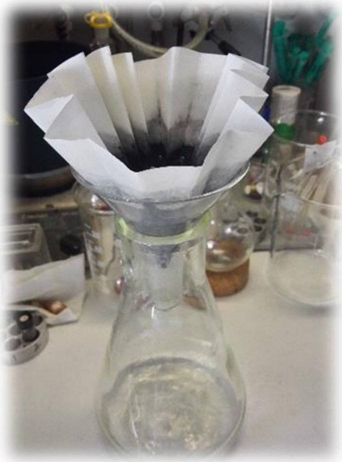

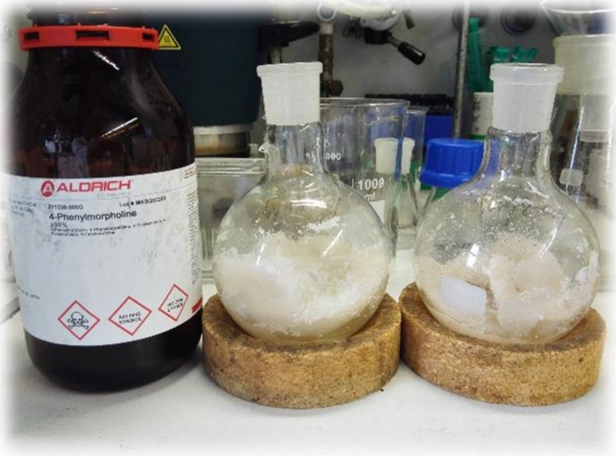

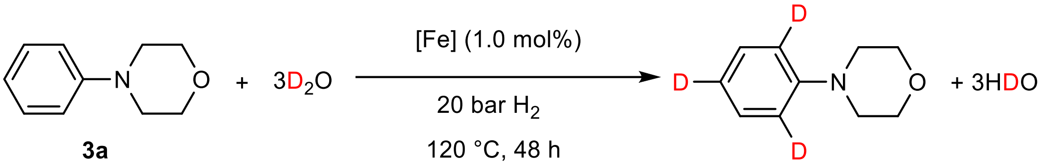

3a
2.0 mol

3b

(1) First run: 120 °C, 120 h.

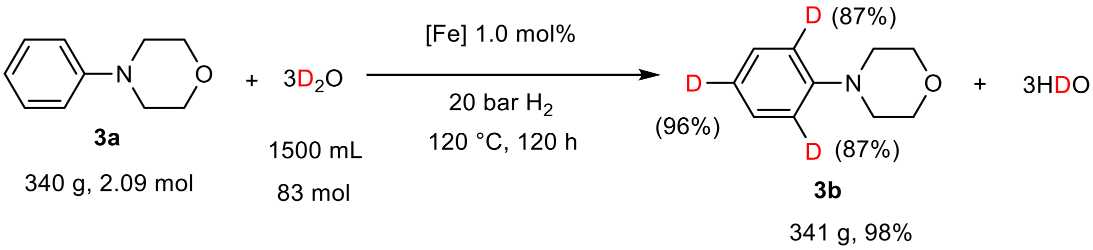

3a
340 g, 2.09 mol

1500 mL
83 mol

3b
341 g, 98%

In a 2 L steel Parr autoclave fitted with 28.0 g Fe-Cellulose-1000 catalyst, 340 g 4-phenylmorpholine (2.09 mol) and deuterium oxide 1500 mL (83 mol) were added. The

autoclave was flushed with hydrogen 6 times at 10 bar and finally pressurized to the desired value (20 bar). Then, it was it placed into an equipment for the autoclave and heated to 120 °C, and then at 120 °C for 120 h. At the end of the reaction, the autoclave was quickly cooled down at room temperature with an ice bath and vented. Finally, the crude reaction was added to ethyl acetate (1.5 L). The reaction mixture was filtered by filter paper (see above). The D_2O layer was removed from the mixture and washed with ethyl acetate (1.5 L, 3 times). After removal of all volatiles in vacuo, 341 g D-product was obtained (see below for characterisation details). Attention: During the cool down, trace amounts of gas could stay in the solid phase (product and catalyst). Thus, the solid phase should be dissolved slowly in ethyl acetate.

Original spectra for the product:

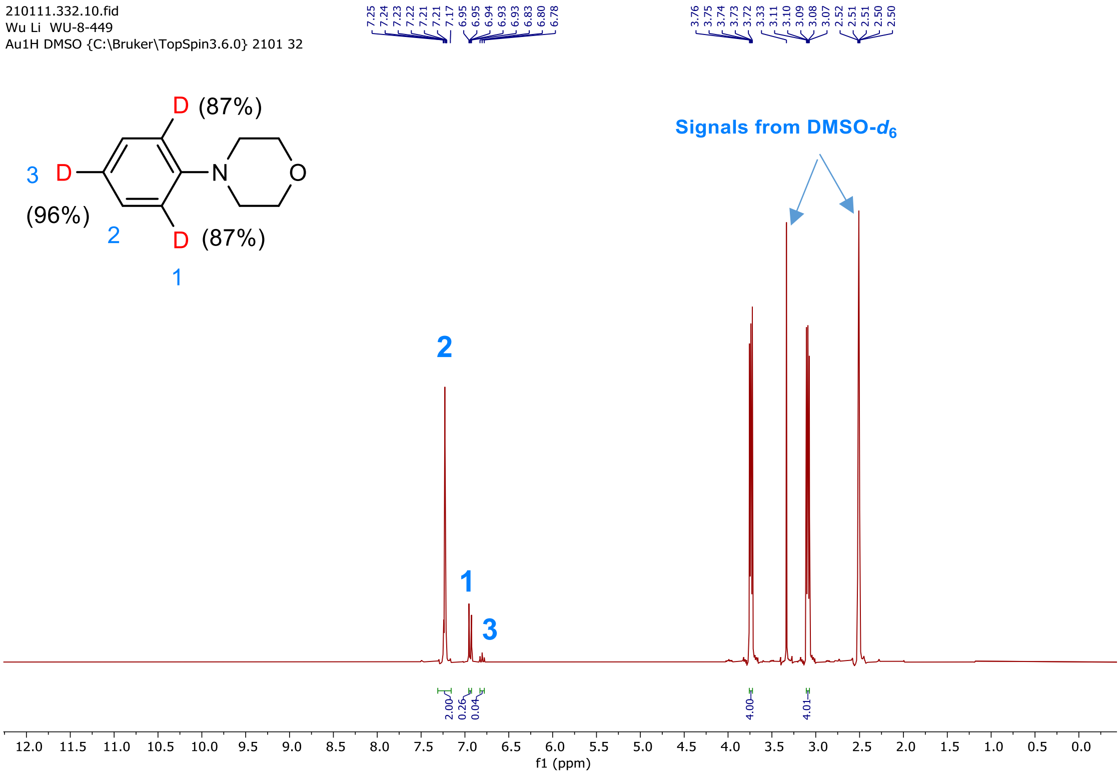

HRMS (ESI-TOF) of 3a:

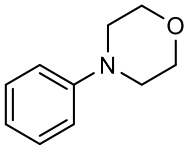

Chemical Formula: $C_{10}H_{13}NO$
Exact Mass: 163.0997

ESI-TOF Accurate Mass Report
File:20073101
Vial:1:D,1
Description:MeOH/0.1% HCOOH in H2O 99:10

Sample Name:WU-1
Date:31-Jul-2020

UserName:Wu Li
Time:11:44:50

Page 2

Sample Report:

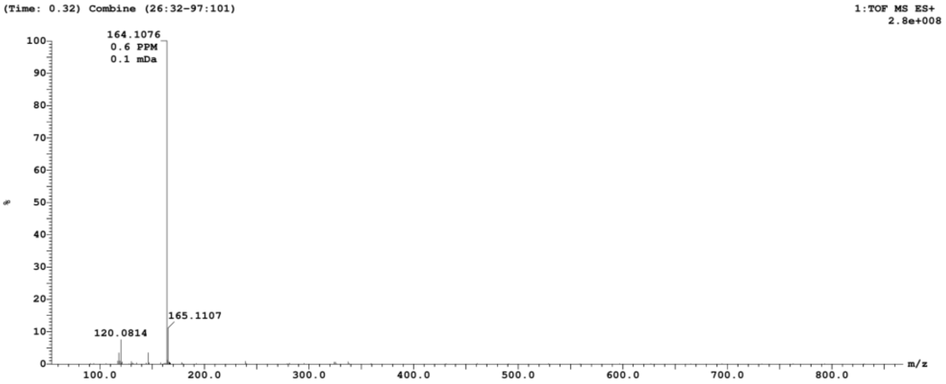

HRMS (ESI-TOF) of 3b:

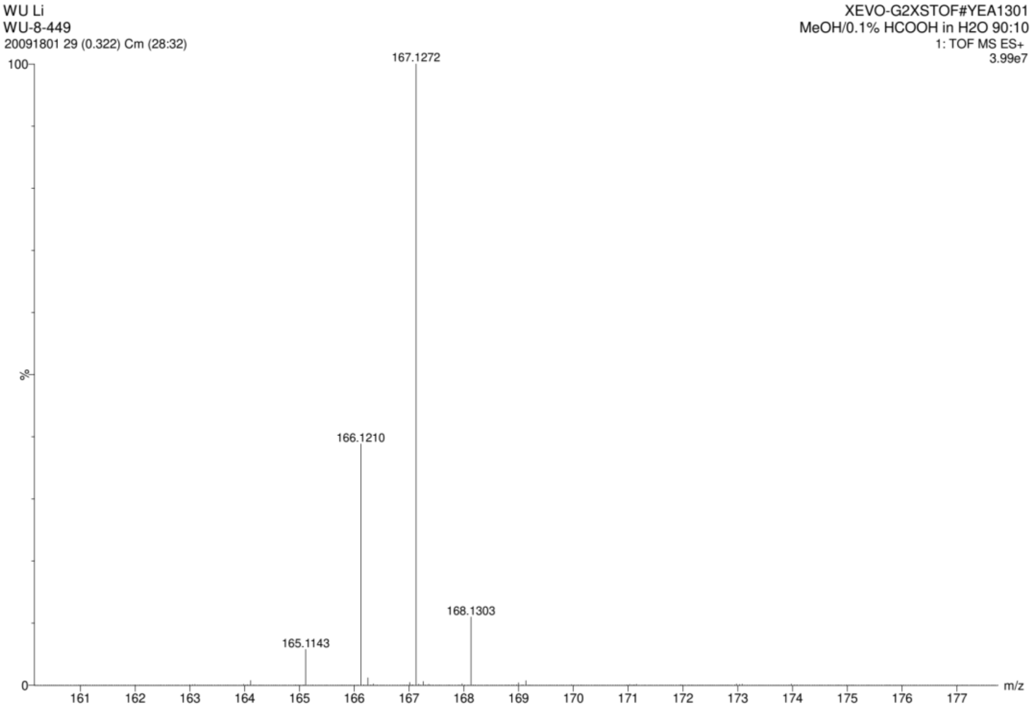

(2) Second run: 140 °C, 48 h.

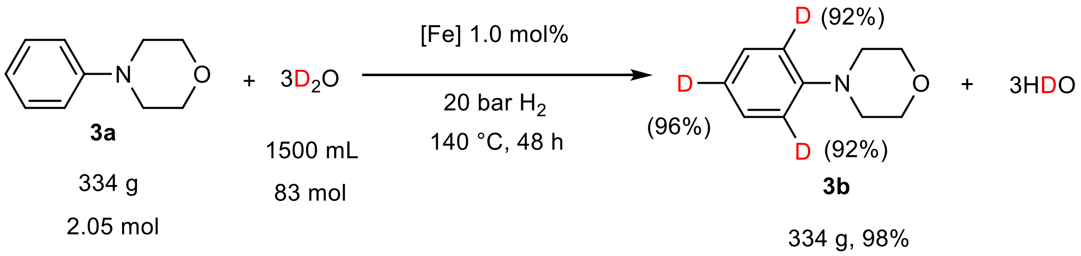

In a 2 L steel Parr autoclave loaded with Fe-Cellulose-1000 catalyst from first run, 334 g 4-phenylmorpholine (2.05 mol) and deuterium oxide 1500 mL (83 mol) were added. The autoclave was flushed with hydrogen 6 times at 10 bar and finally pressurized to the desired value (20 bar). Then, it was placed into an equipment for the autoclave and heated to 140 °C and then at 140 °C for 48 h. At the end of the reaction, the autoclave was quickly cooled down to room temperature with an ice bath and vented. Finally, the crude reaction was added to ethyl acetate (1.5 L). The reaction mixture was filtered by filter paper. The D_2O layer was removed from the mixture and washed with ethyl acetate (1.5 L, 3 times). After removal of all volatiles in vacuo, 334 g D-product was obtained (see below for characterisation details). Attention: During the cool down trace amount of gas can stay in the solid phase (product and catalyst). Thus, the solid phase should be dissolved slowly in ethyl acetate.

Original spectra for the product:

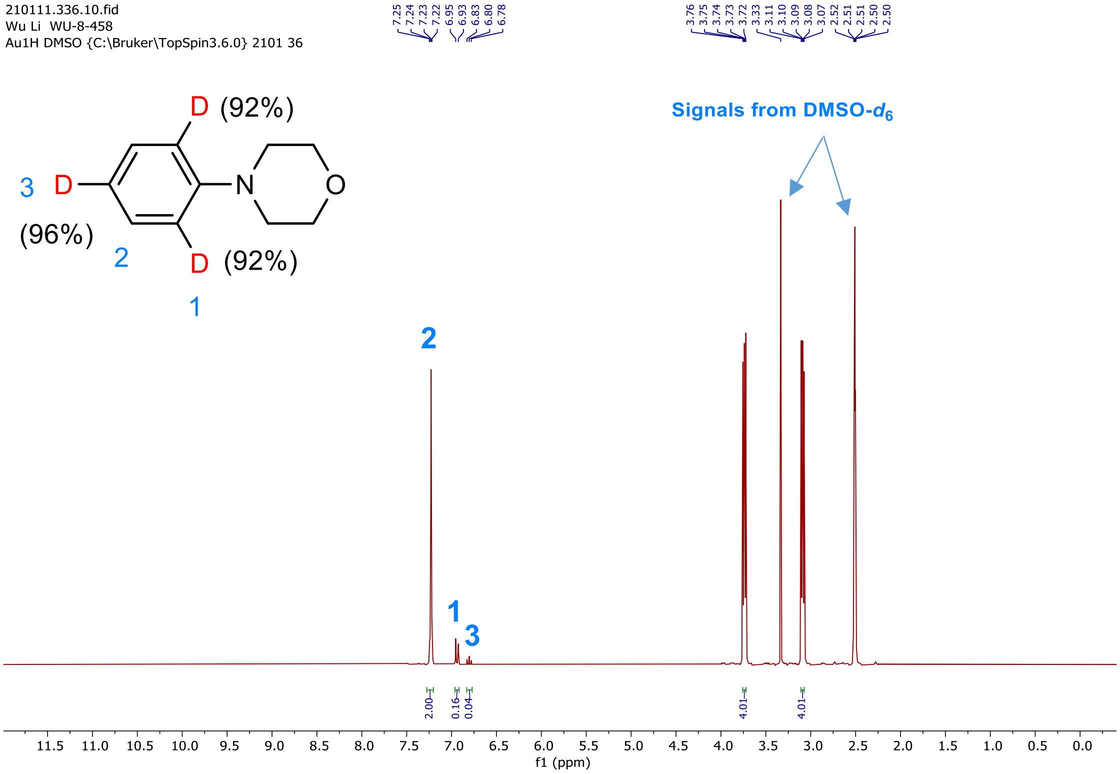

HRMS (ESI-TOF) of the product:

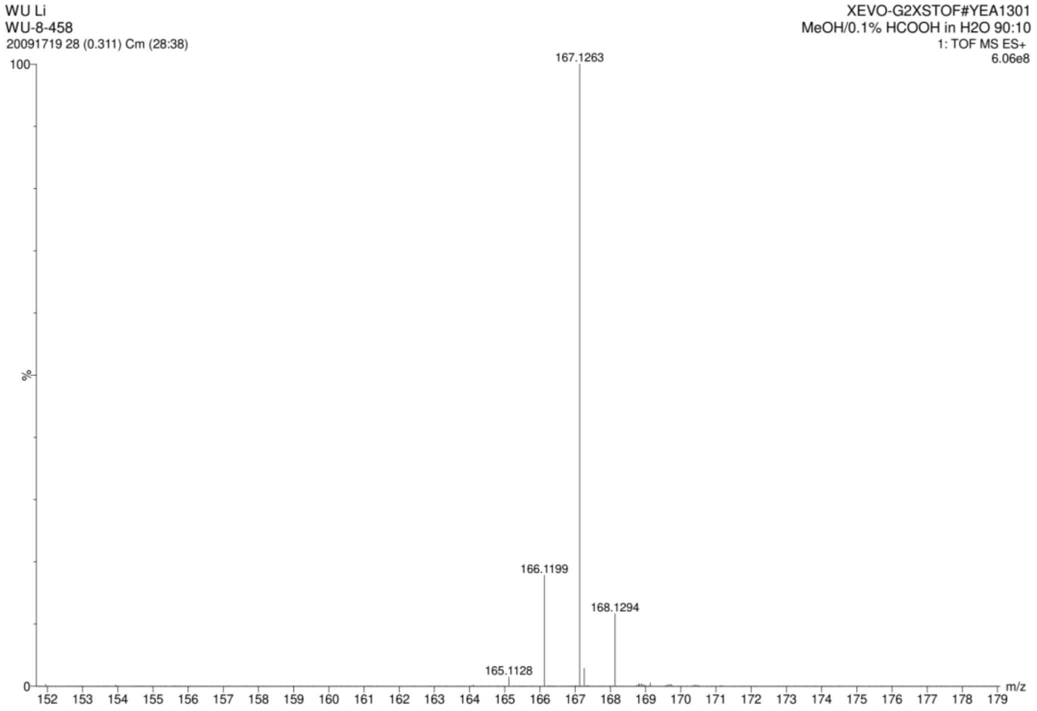

(3) Third run: 140 °C, 48 h.

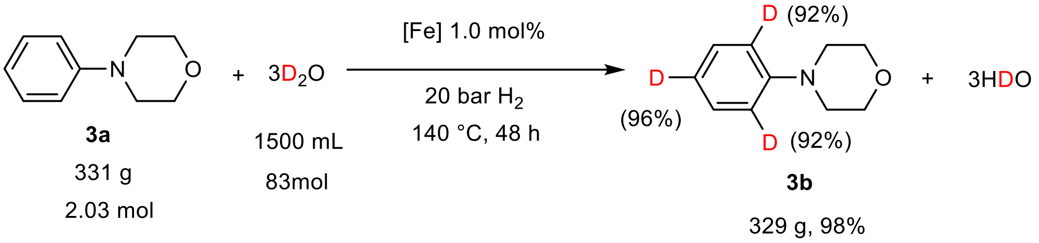

In a 2 L steel Parr autoclave loaded with Fe-Cellulose-1000 catalyst from second run, 331 g 4-phenylmorpholine (2.03 mol) and deuterium oxide 1500 mL (83 mol) were added. The autoclave was flushed with hydrogen 6 times at 10 bar and finally pressurized to the desired value (20 bar). Then, it was placed into an equipment for the autoclave and heated to 140 °C and then at 140 °C for 48 h. At the end of the reaction, the autoclave was quickly cooled down to room temperature with an ice bath and vented. Finally, the crude reaction was added to ethyl acetate. The reaction mixture was filtered by filter paper. The D_2O layer was removed from the mixture and washed with ethyl acetate (1.5 L, 3 times). After removal of all volatiles in vacuo, 329 g D-product was obtained (see below for characterisation details). Attention: During the cool down trace amounts of gas can stay in the solid phase (product and catalyst). Thus, the solid phase should be dissolved slowly in ethyl acetate.

Original spectra for the product:

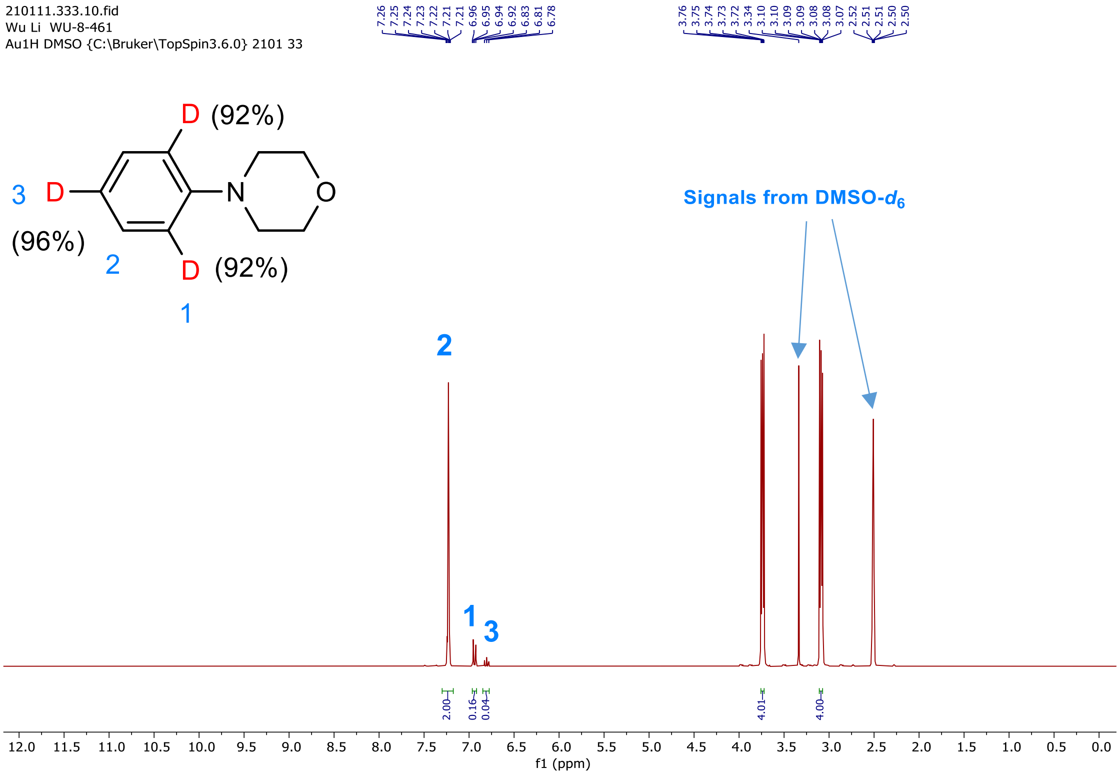

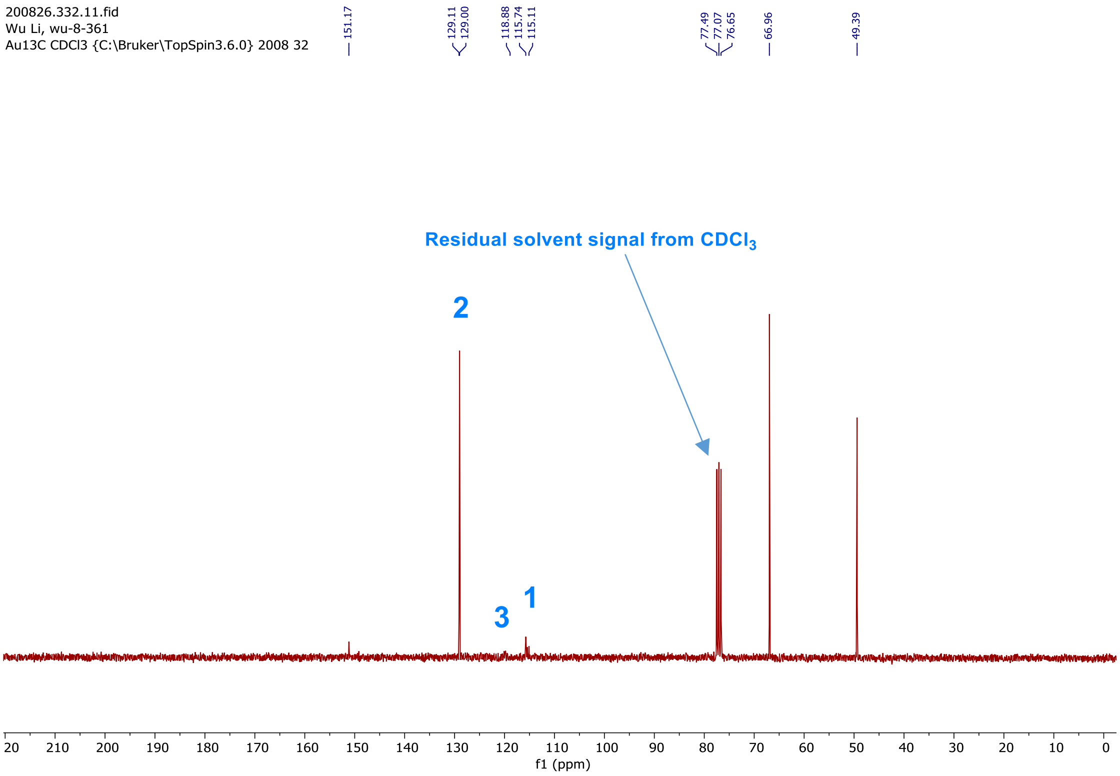

HRMS (ESI-TOF) of the product:

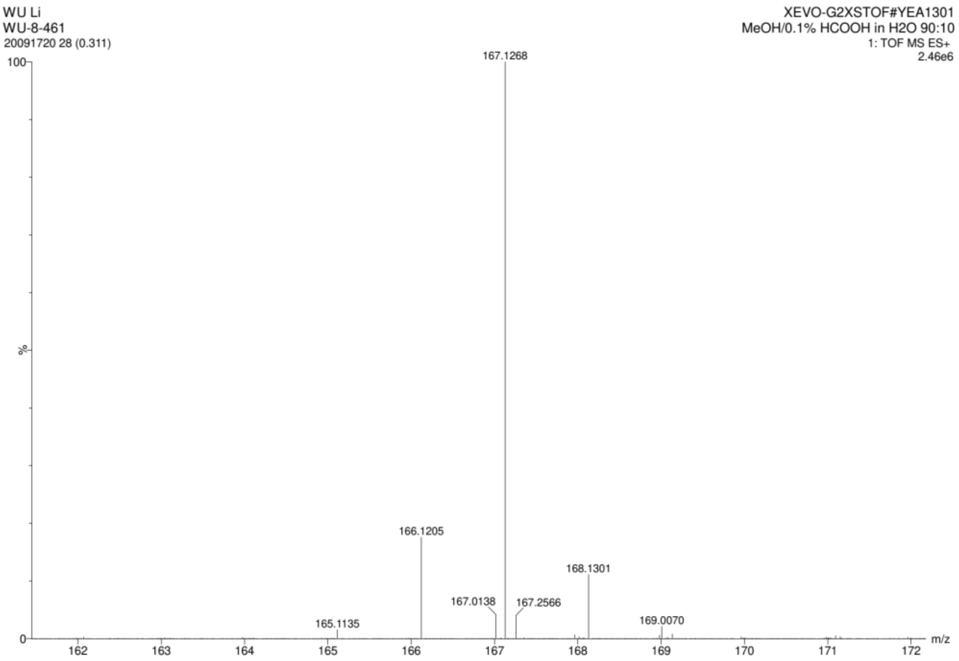

(4) Fourth run: 140 °C, 48 h.

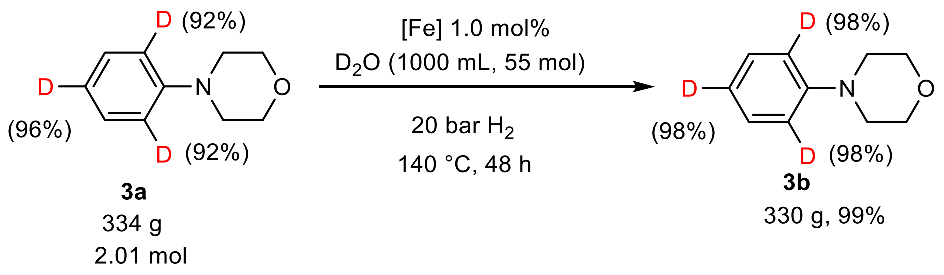

In a 2 L steel Parr autoclave loaded with Fe-Cellulose-1000 catalyst from third run, 334 g 4-phenylmorpholine-D (2.01 mol) and deuterium oxide 1000 mL (55 mol) were added. The autoclave was flushed with hydrogen 6 times at 10 bar and finally pressurized to the desired value (20 bar). Then, it was it placed into an equipment for the autoclave and heated to 140 °C and then at 140 °C for 48 h. At the end of the reaction, the autoclave was quickly cooled down at room temperature with an ice bath and vented. Finally, the crude reaction was added to ethyl acetate (1.5 L). The reaction mixture was filtered by filter paper. The D_2O layer was removed from the mixture and washed with ethyl acetate (1.5 L, 3 times). After removal of all volatiles in vacuo, 330 g D-product was obtained (see below for characterisation details). (Attention: During the cool down, trace amount of gas can stay in the solid phase (product and catalyst). The solid phase should be dissolved in ethyl acetate slowly.)

Original spectra for the product:

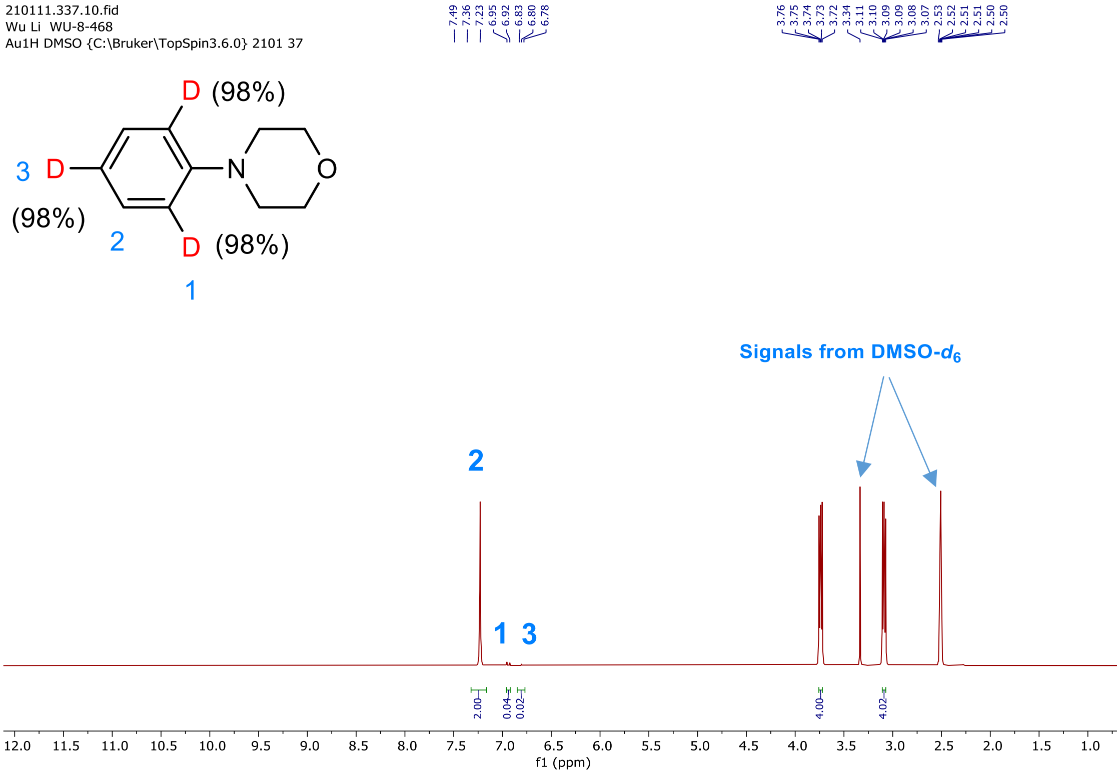

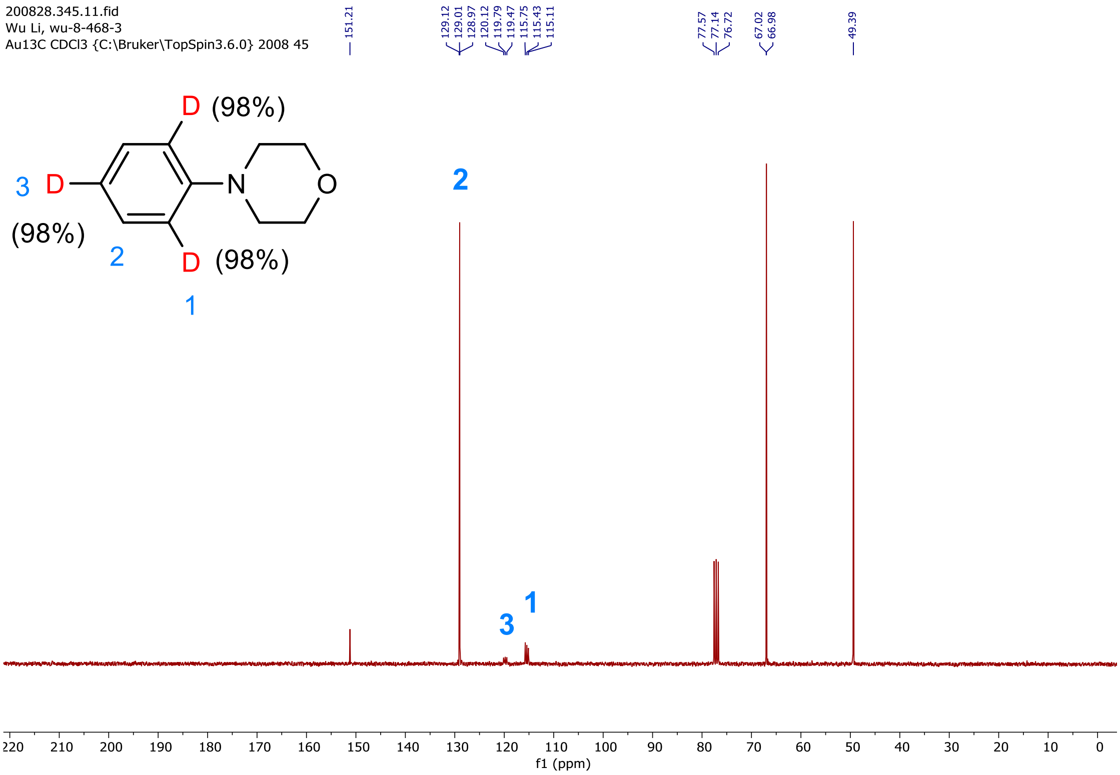

HRMS (ESI-TOF) of the product:

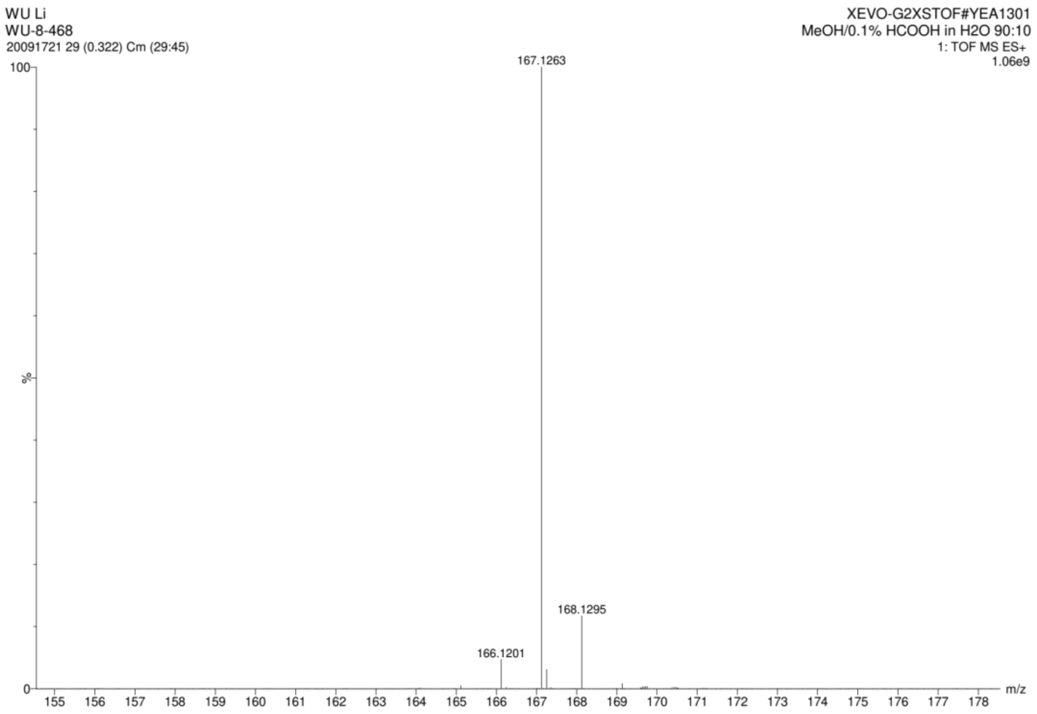

b) The same catalyst, different substrates

(1) First run for 1, 2, 3, 4-tetrahydroquinoline

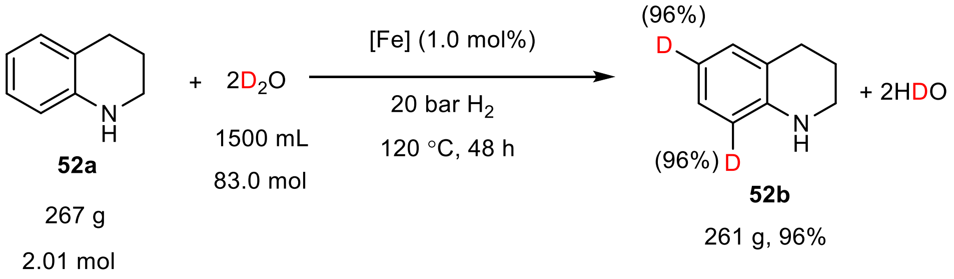

In a 2 L steel Parr autoclave loaded with 28.0 g Fe-Cellulose-1000 catalyst, 267 g 1, 2, 3, 4-tetrahydroquinoline (2.01 mol) and deuterium oxide 1500 mL (83 mol) were added. The autoclave was flushed with hydrogen 6 times at 10 bar and finally pressurized to the desired value (20 bar). Then, it was it placed into an equipment for the autoclave and heated to 140 °C and then at 140 °C for 48 h. At the end of the reaction, the autoclave was quickly cooled down at room temperature with an ice bath and vented. Finally, the crude reaction was added to ethyl acetate (1.5 L). The reaction mixture was filtered by filter paper (see above). The D_2O layer was removed from the mixture and washed with ethyl acetate (1.5 L, 3 times). After removal of all volatiles in vacuo, 261 g D-product was obtained (see below for characterisation details).

Original spectra for the product:

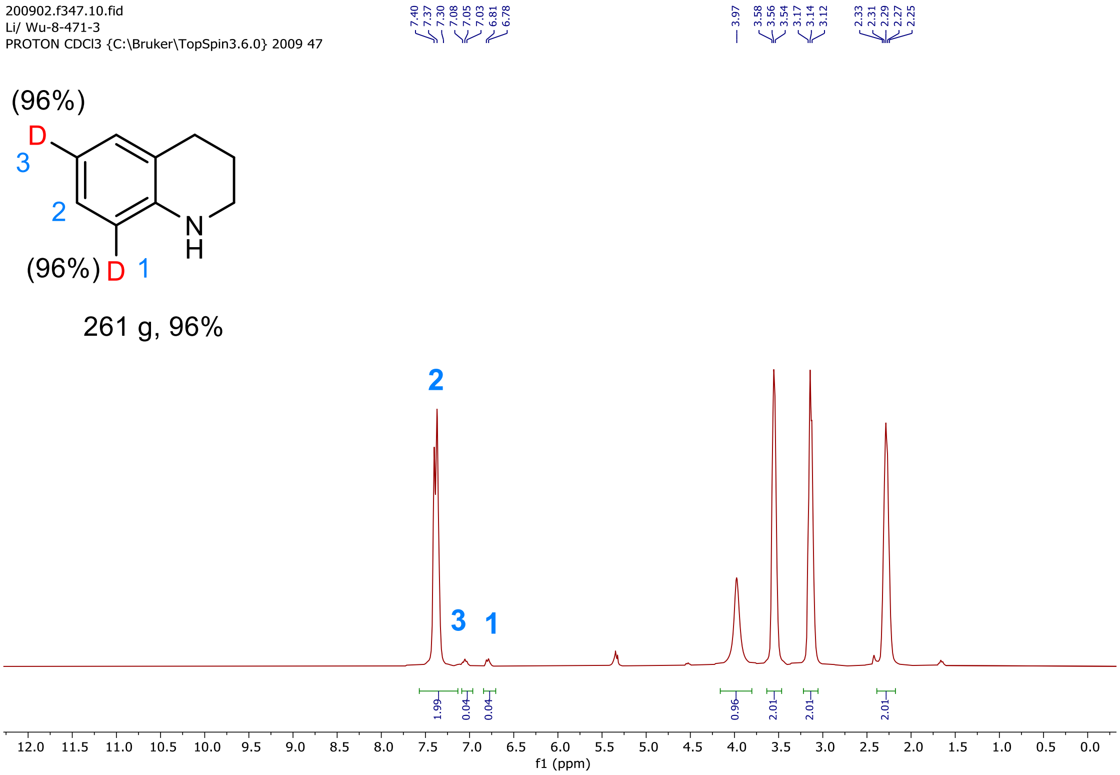

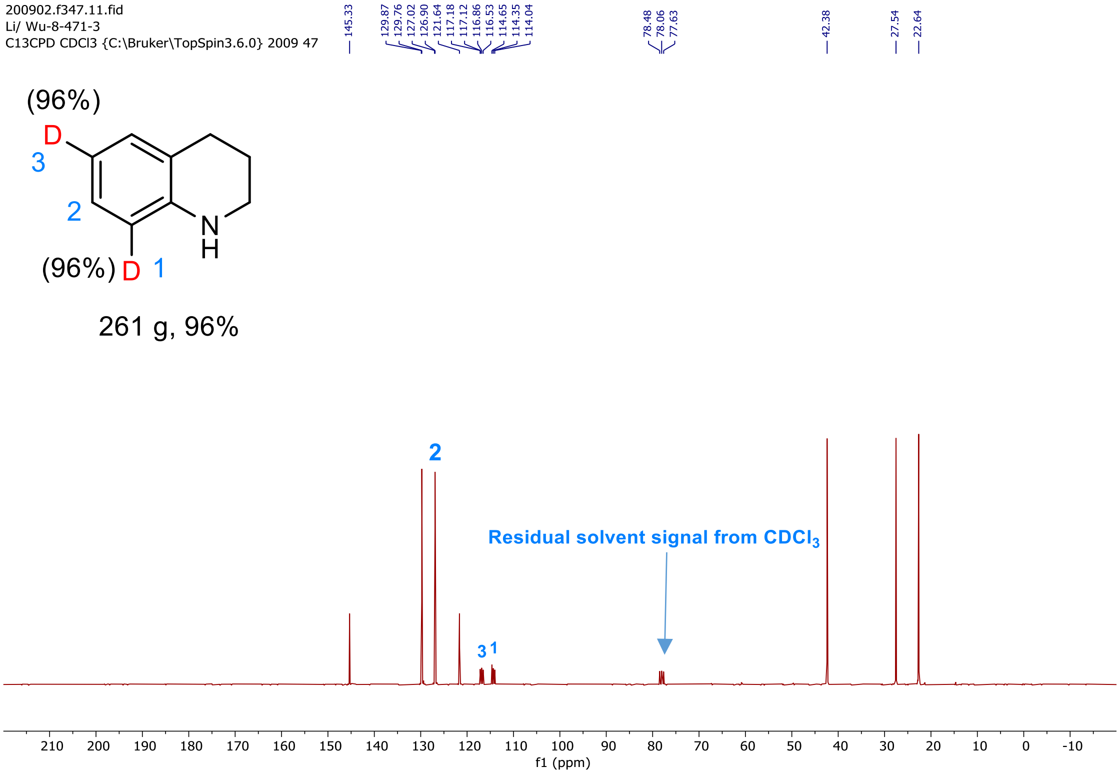

HRMS (ESI-TOF) of the unlabelled substrate:

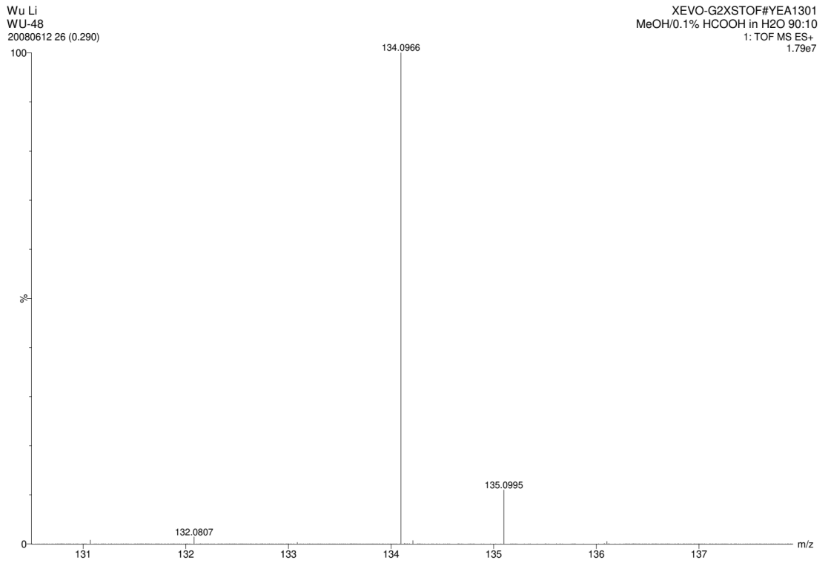

HRMS (ESI-TOF) of the product:

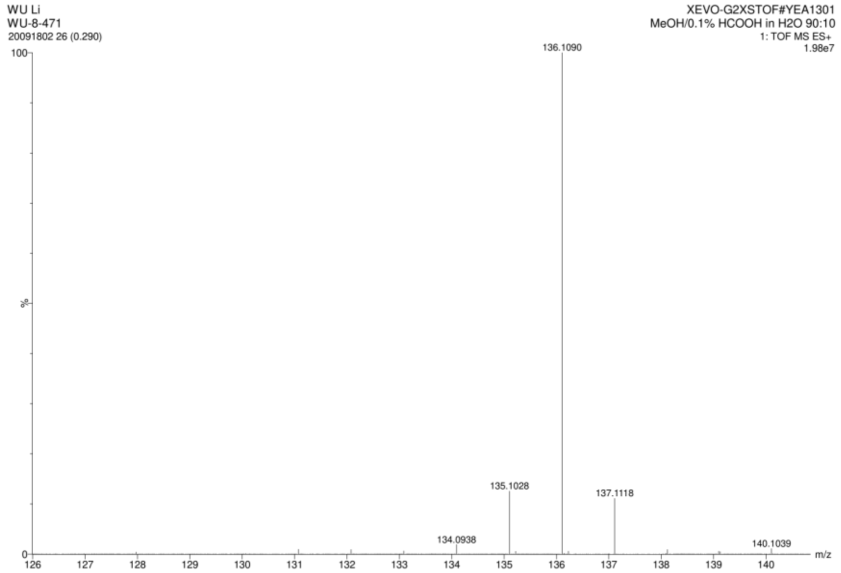

(2) Second run for Carvacrol

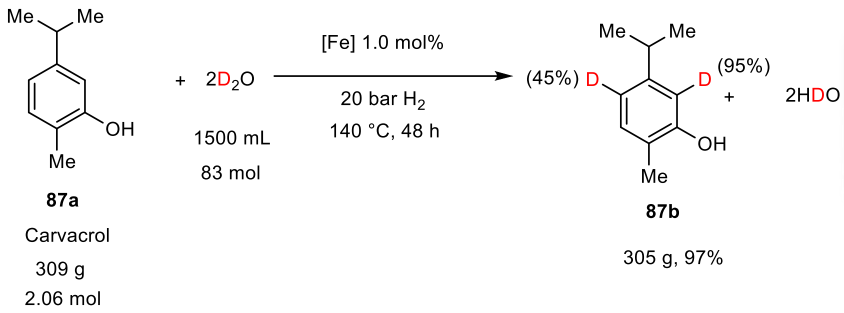

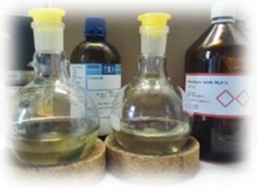

In a 2 L steel Parr autoclave loaded with Fe-Cellulose-1000 catalyst from the first run, 309 g Carvacrol (2.06 mol) and deuterium oxide 1500 mL (83 mol) were added. The autoclave was flushed with hydrogen 6 times at 10 bar and finally pressurized to the desired value (20 bar). Then, it was it placed into an equipment for the autoclave and heated to 140 °C and then at 140 °C for 48 h. At the end of the reaction, the autoclave was quickly cooled down at room temperature with an ice bath and vented. Finally, the crude mixture was added to ethyl acetate (1.5 L). The reaction mixture was filtered by filter paper. The D_2O layer was removed from the mixture and washed with ethyl acetate (1.5 L, 3 times). After removal of all volatiles in vacuo, 305 g D-product was obtained (see below for characterisation details).

Original spectra for the product:

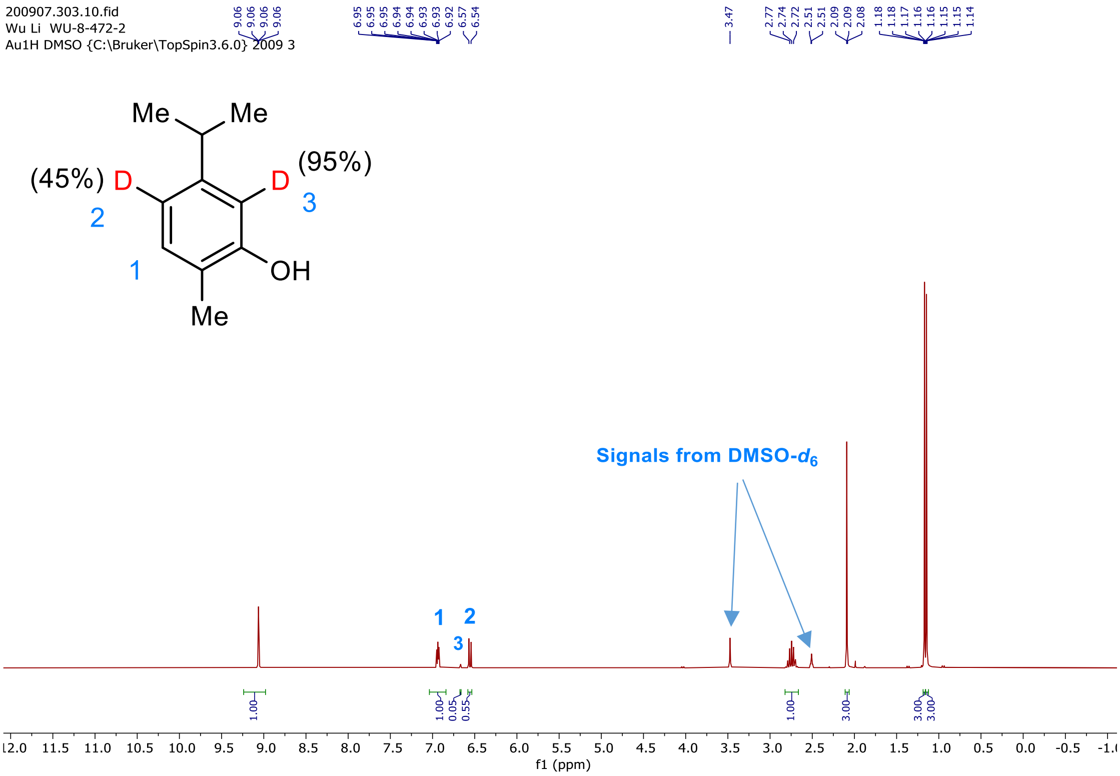

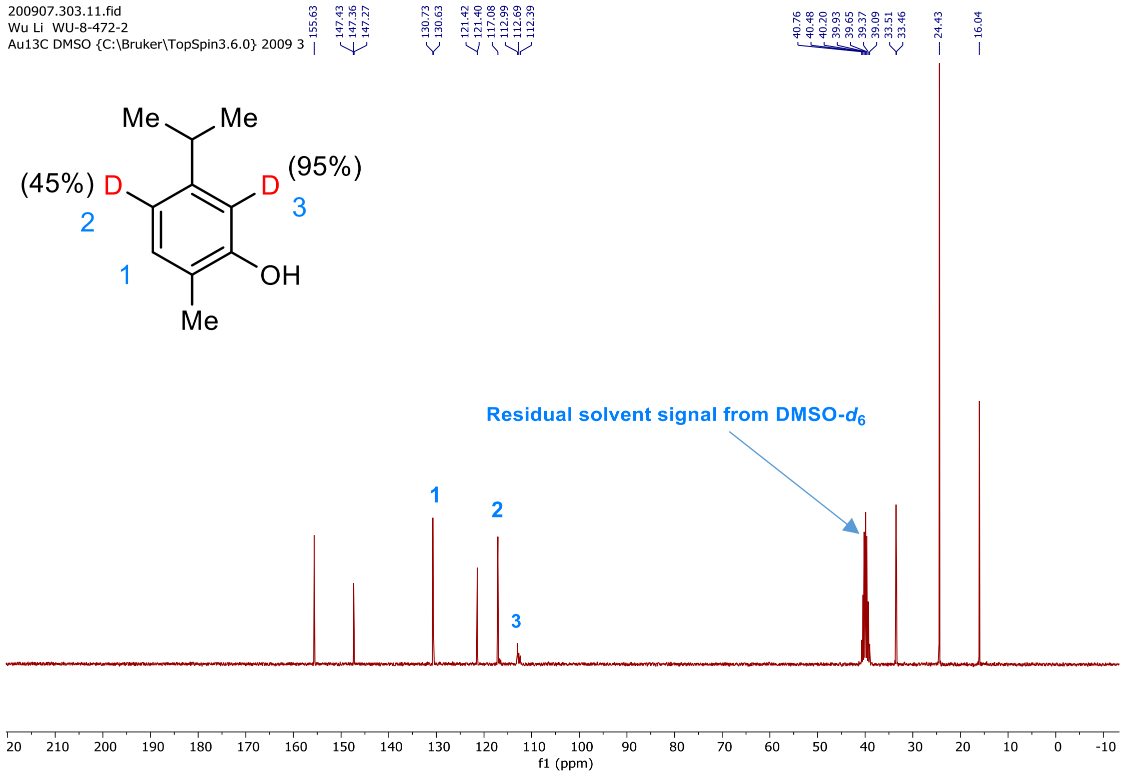
200907.303.11.fid
Wu Li WU-8-472-2
Au13C DMSO {C:\Bruker\TopSpin3.6.0} 2009 3
155.63
147.43
147.36
147.27
130.73
130.63
121.42
121.40
117.08
112.99
112.69
112.39
40.76
40.48
40.20
39.93
39.65
39.37
39.09
33.51
33.46
24.43
16.04
Me
Me
(45%) D
D (95%)
2
3
1
OH
Me
Residual solvent signal from DMSO-d_6
1
2
3
f1 (ppm)

10. Procedure for catalyst recycling

In a 4 mL vial fitted with magnetic stirring bar and septum cap, iron catalyst (60 mg) and 4-phenylmorpholine were added, independently. Then, a needle was inserted in the septum which allows gaseous reagents to enter. Solvent D_2O (1.5 mL) was added. The vial (up to eight) was set in an alloy plate and then placed into a 300 mL steel Parr autoclave. The autoclave was flushed with hydrogen 6 times at 10 bar and finally pressurized to the desired value (20 bar). Then, it was placed into an aluminum block and heated to the desired temperature (120 °C). The reaction was stirred for 24 hours at 120 °C. At the end of the reaction, the autoclave was quickly cooled down to room temperature with an ice bath and vented. Finally, the samples were removed from the autoclave, and ethyl acetate was added to the crude mixture. The reaction mixture was centrifuged, and the organic layer was removed from the vials (3 times). After removal of all volatiles in vacuo the desired products were obtained. The catalyst was washed by 3.0 mL EtOH for three times by centrifugation. Finally, the iron catalyst was evacuated and then dried under an oil pump vacuum for 4 hours. The ICP-MS results are summarized in Supplementary Table 8 and no iron leaching into the liquid phase was observed with this heterogeneous iron catalyst.

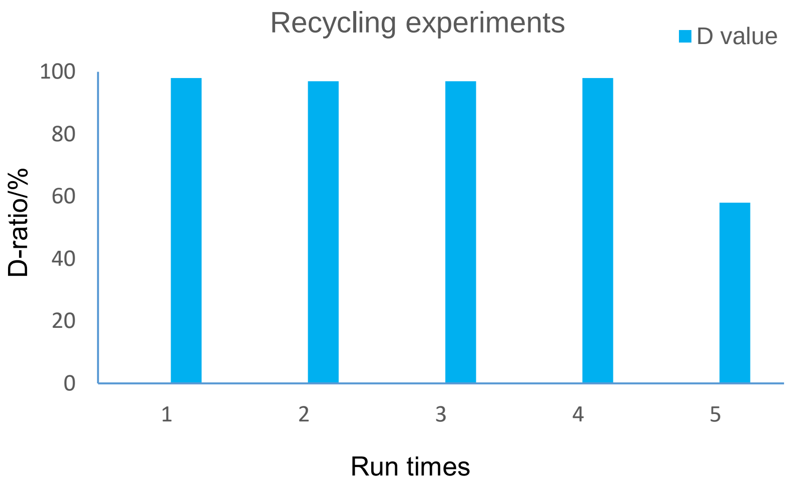

Supplementary Fig. 18 Recycling of the Fe-Cellulose-1000

11. ICP-OES analysis

ICP-OES (inductively coupled plasma optical emission spectrometry) for quantification analysis: Varian/Agilent 715-ES

Supplementary Table 8. ICP-OES results of the solution above the iron catalyst during several recycling experiments

ICP-OES

ANALYSENERGEBNIS

FILTER: *Wu, Li* *9873* *9877* *

ICP-Nr	Probe			Ergebnis *Element*	*erw.*	*gef(a)*	*gef(b)*
9873	WU-7-746-1	10.02.2020	g/l	Fe	-	nn	nn
9874	WU-7-746-2	10.02.2020	g/l	Fe	-	nn	nn
9875	WU-7-746-3	10.02.2020	g/l	Fe	-	nn	nn
9876	WU-7-746-4	10.02.2020	g/l	Fe	-	nn	nn
9877	WU-7-746-5	10.02.2020	g/l	Fe	-	nn	nn

erw.= erwarteter Wert, gef(1) / (2) = gefundener Wert (Einfach-, Doppelbestimmung) - = keine Angabe

Datum 10.02.2020

NWG: 0,01w% Fe

12. Characterisation Data for Substrates and Products

Positions and percentage of deuterium incorporation were determined by ^{1}H NMR. The equation below was used to determine the degree of D-incorporation; peaks were calibrated against a signal corresponding to a unlabelled position.

$$\% \text{ Deuteration} = 100 - \left[\left(\frac{\text{Residual integral}}{\text{no. labelling sites}}\right) \times 100\right]$$

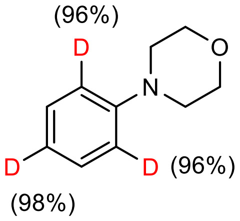

3b

According to GP, Fe-Cellulose-1000 (60 mg, 0.05 mol), substrate (41 mg, 0.25 mmol), D_2O (1.5 mL), H_2 (20 bar), room temperature to 120 °C and then at 120 °C for 24 h. The product **3b** (40 mg, 0.24 mmol, 96%) was obtained.

^{1}H NMR (300 MHz, DMSO-d_6) δ 7.23 (s, 2H), 6.95 – 6.92 (m, 0.07H), 6.83 – 6.78 (m, 0.02H), 3.76 – 3.72 (m, 4H), 3.11 – 3.07 (m, 4H).

^{13}C NMR (101 MHz, DMSO-d_6) δ 151.50, 129.30, 119.55, 119.30, 115.53, 115.24, 115.01, 66.59, 48.88.

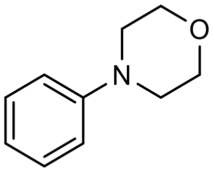

Chemical Formula: $C_{10}H_{13}NO$
Exact Mass: 163.0997

HRMS (ESI-TOF) of 3a:

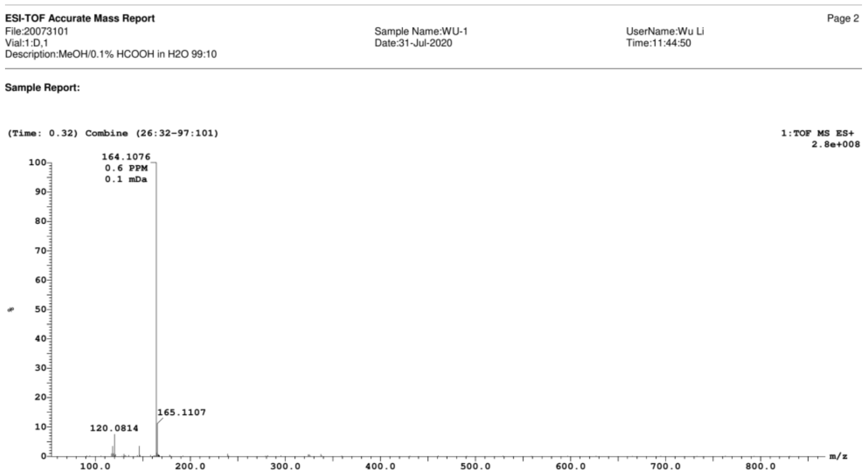

HRMS (ESI-TOF) of 3b [M+H]⁺:

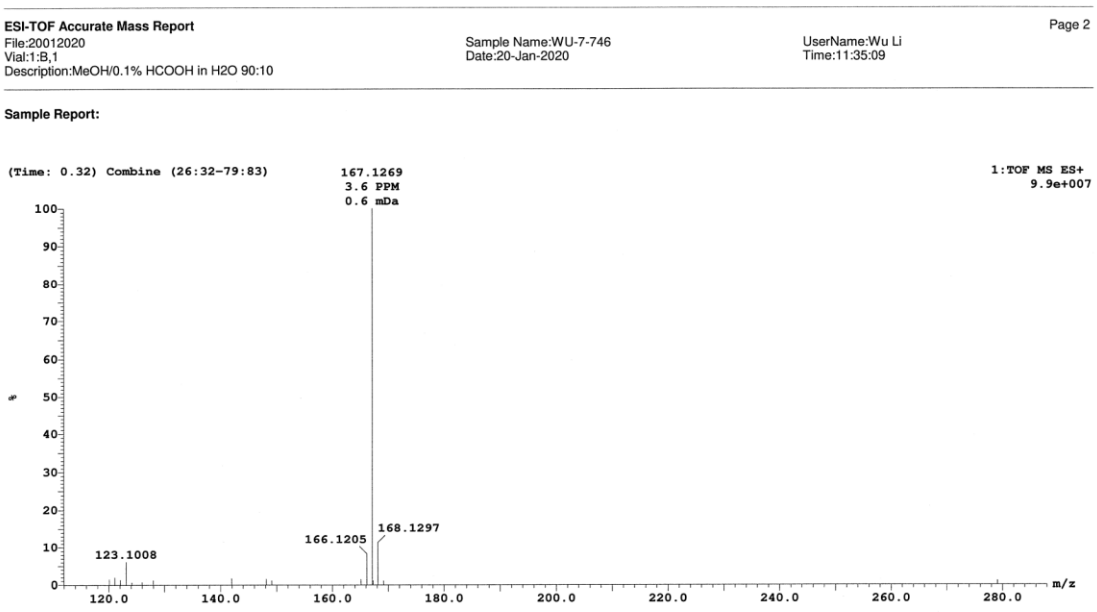

Comparison of the measured deuterium content using NMR integrals obtained from "routine" and quantitative NMR experiments

	Position	Starting material	Product	D-content
**Routine NMR**	*ortho*	1.9	1.78	6%
	meta	1.95	0.03	98%
	para	0.91	0.01	99%
**Quantitative NMR**	*ortho*	1.99	2	0%
	meta	1.97	0.03	98%
	para	0.99	0.01	99%

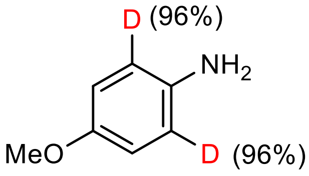

4b

According to GP, Fe-Cellulose-1000 (60 mg, 0.05 mol), substrate (33 mg, 0.27 mmol), D_2O (1.5 mL), H_2 (20 bar), room temperature to 120 °C and then at 120 °C for 24 h. The product **4b** (32 mg, 0.26 mmol, 96%) was obtained.

^{1}H NMR (300 MHz, $CDCl_3$) δ 6.75 (s, 2H), 6.67 (0.08), 3.75 (s, 3H), 3.31 (s, 2H).

^{13}C NMR (75 MHz, $CDCl_3$) δ 152.93, 139.88, 116.58, 116.27, 115.96, 114.81, 55.85.

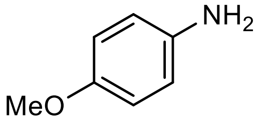

Chemical Formula: C_7H_9NO
Exact Mass: 123.0684

HRMS (ESI-TOF) of 4a:

ESI-TOF Accurate Mass Report Page 2
File:20073119 Sample Name:WU-2 UserName:Wu Li
Vial:1:D,2 Date:31-Jul-2020 Time:12:31:32
Description:MeOH/0.1% HCOOH in H2O 99:10

Sample Report:

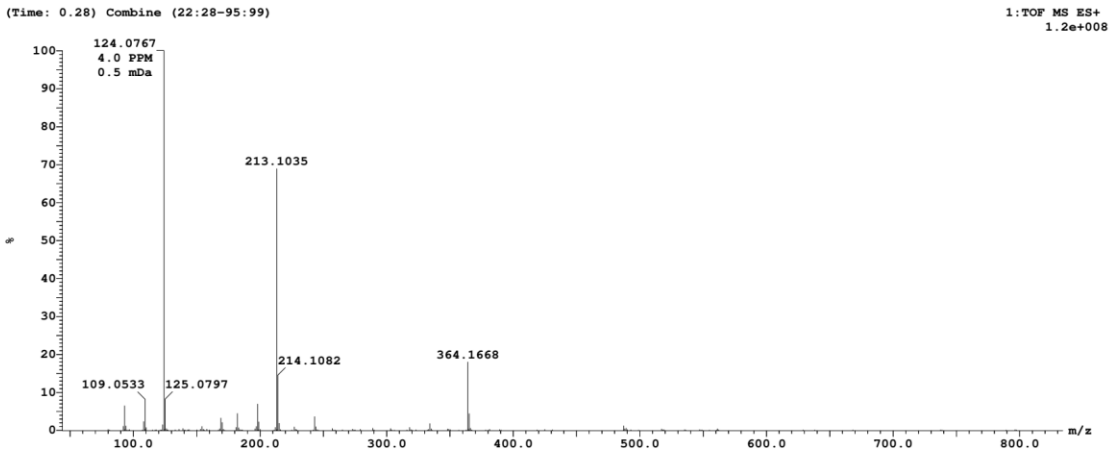

HRMS (ESI-TOF) of 4b [M+H]$^+$:

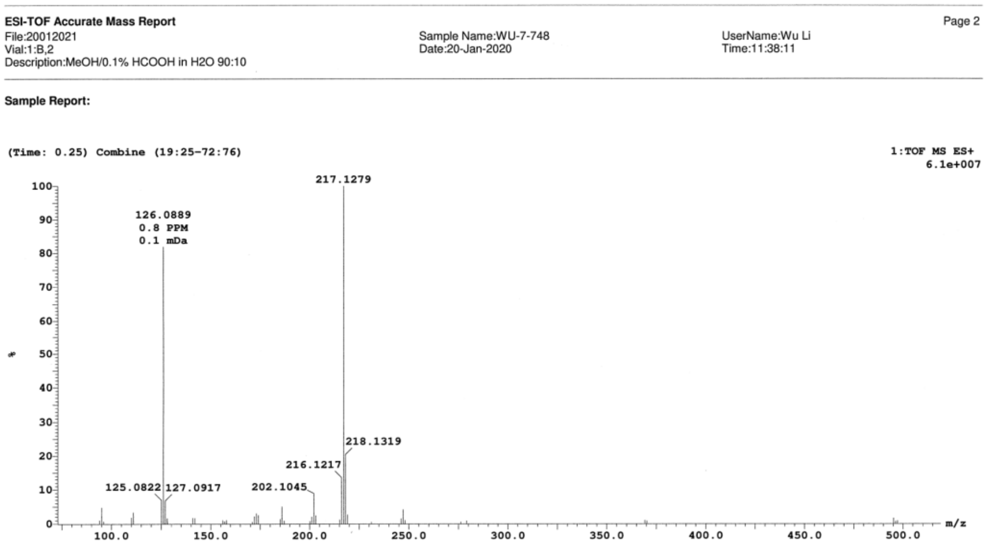

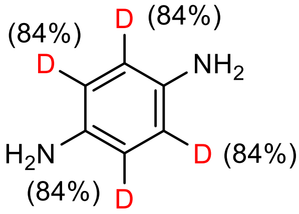

5b

According to GP, Fe-Cellulose-1000 (60 mg, 0.05 mol), substrate (29 mg, 0.27 mmol), D_2O (1.5 mL), H_2 (20 bar), room temperature to 120 °C and then at 120 °C for 24 h. The product **5b** (30 mg, 0.27 mmol, >99%) was obtained. Internal standard trimethoxybenzene was also used to confirm the deuterium content.

^{1}H NMR (300 MHz, $CDCl_3$) δ 6.57 (s, 0.65H), 3.31 (s, 4H).

^{13}C NMR (101 MHz, DMSO-d_6) δ 138.74, 115.28, 115.02, 114.79.

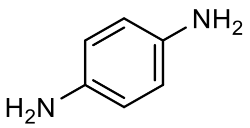

Chemical Formula: $C_6H_8N_2$
Exact Mass: 108.0687

HRMS (ESI-TOF) of 5a:

ESI-TOF Accurate Mass Report Page 2

File:20073120 Sample Name:WU-3 UserName:Wu Li
Vial:1:D,3 Date:31-Jul-2020 Time:12:34:03
Description:MeOH/0.1% HCOOH in H2O 99:10

Sample Report:

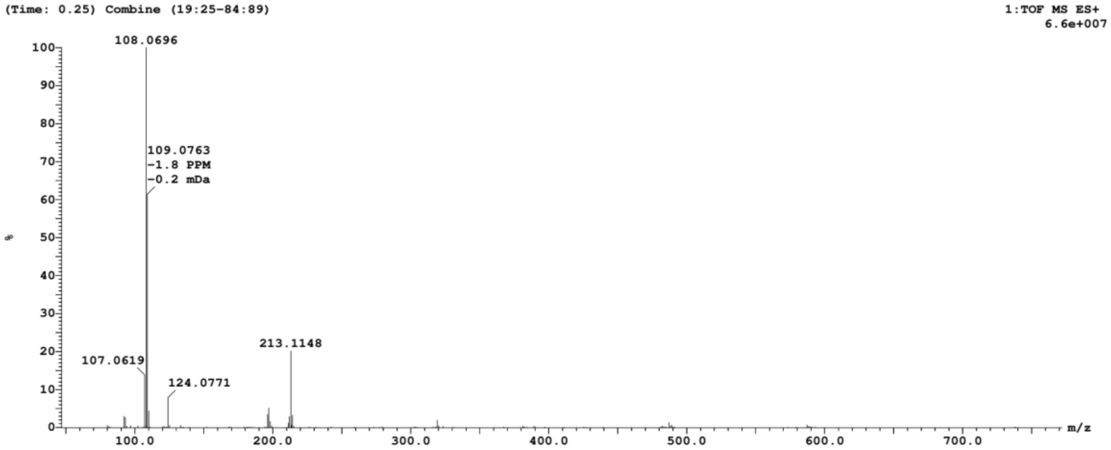

HRMS (ESI-TOF) of 5b $[M+H]^+$:

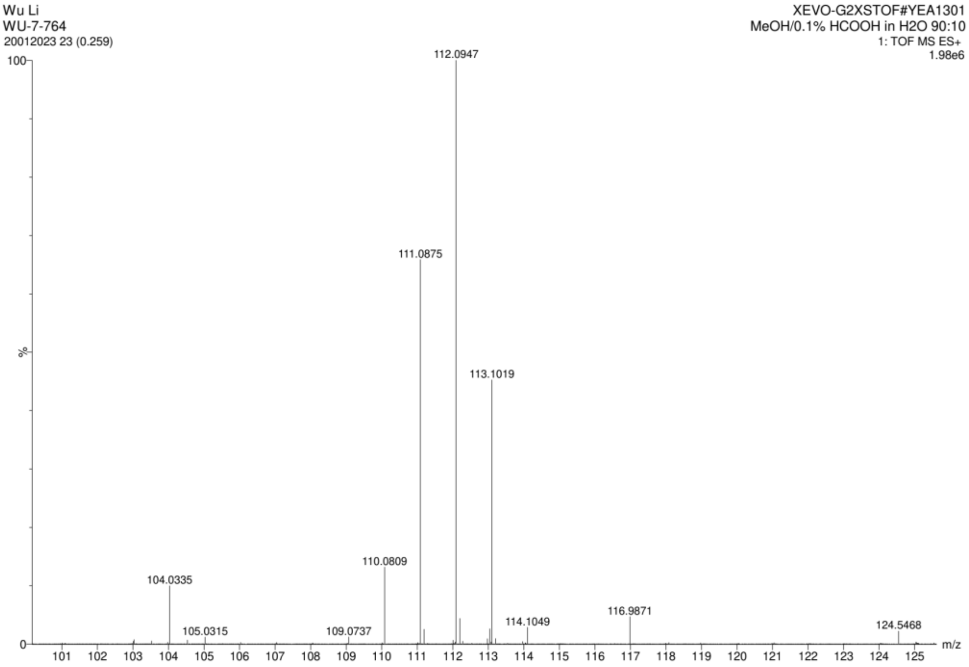

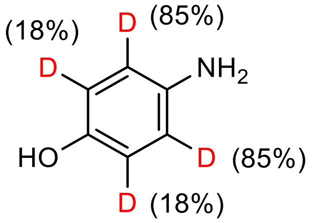

6b

According to GP, Fe-Cellulose-1000 (60 mg, 0.05 mol), substrate (35 mg, 0.32 mmol), D_2O (1.5 mL), H_2 (20 bar), room temperature to 120 °C and then at 120 °C for 24 h. The product **6b** (27 mg, 0.24 mmol, 77%) was obtained.

^1H NMR (300 MHz, DMSO-d_6) δ 8.33 (s, 1H), 6.47 (s, 1.64H), 6.43 – 6.40 (m, 0.30H), 4.34 (s, 2H).

^{13}C NMR (75 MHz, DMSO-d_6) δ 148.14, 140.47, 115.38, 115.25, 114.67.

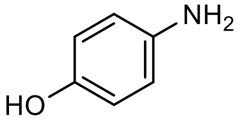

Chemical Formula: C_6H_7NO
Exact Mass: 109.0528

HRMS (ESI-TOF) of 6a:

ESI-TOF Accurate Mass Report Page 2
File:20073121
Vial:1:D,4
Description:MeOH/0.1% HCOOH in H2O 99:10
Sample Name:WU-4
Date:31-Jul-2020
UserName:Wu Li
Time:12:36:39

Sample Report:

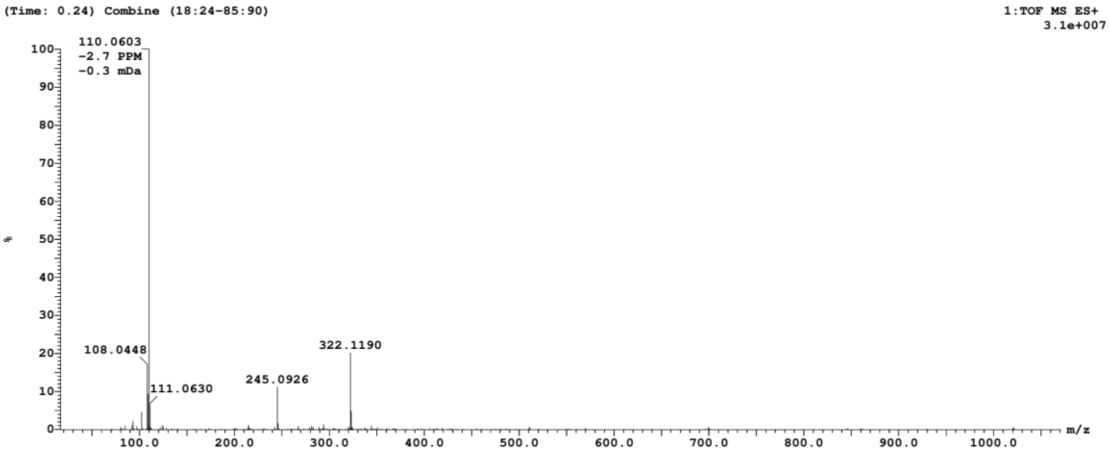

HRMS (ESI-TOF) of 6b [M+H]+:

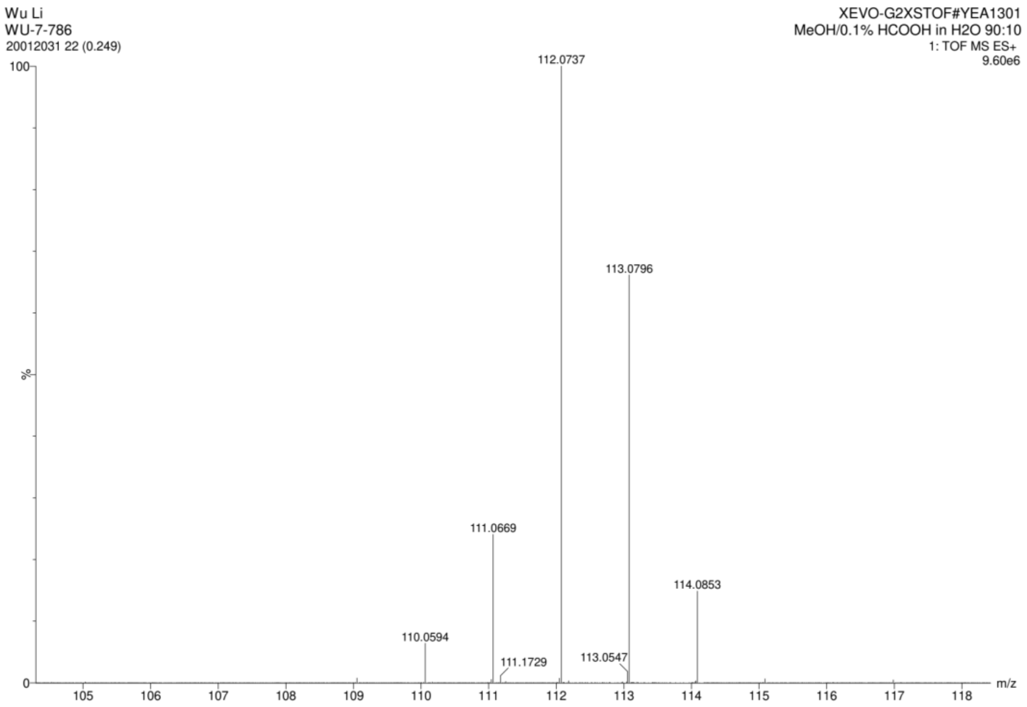

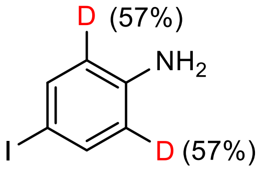

7b

According to GP, Fe-Cellulose-1000 (61 mg, 0.05 mol), substrate (57 mg, 0.26 mmol), D_2O (1.5 mL), H_2 (20 bar), room temperature to 120 °C and then at 120 °C for 24 h. The product **7b** (51 mg, 0.23 mmol, 88%) was obtained.

^{1}H NMR (300 MHz, $CDCl_3$) δ 7.41 (ddd, J = 4.1, 2.8, 1.0 Hz, 2H), 6.47 (d, J = 8.8 Hz, 0.87H), 3.50 (s, 2H).

^{13}C NMR (75 MHz, $CDCl_3$) δ 145.16, 139.00, 138.03, 137.94, 129.20, 117.42.

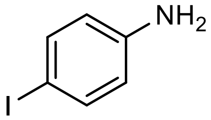

Chemical Formula: C_6H_6IN
Exact Mass: 218.9545

HRMS (ESI-TOF) of 7a:

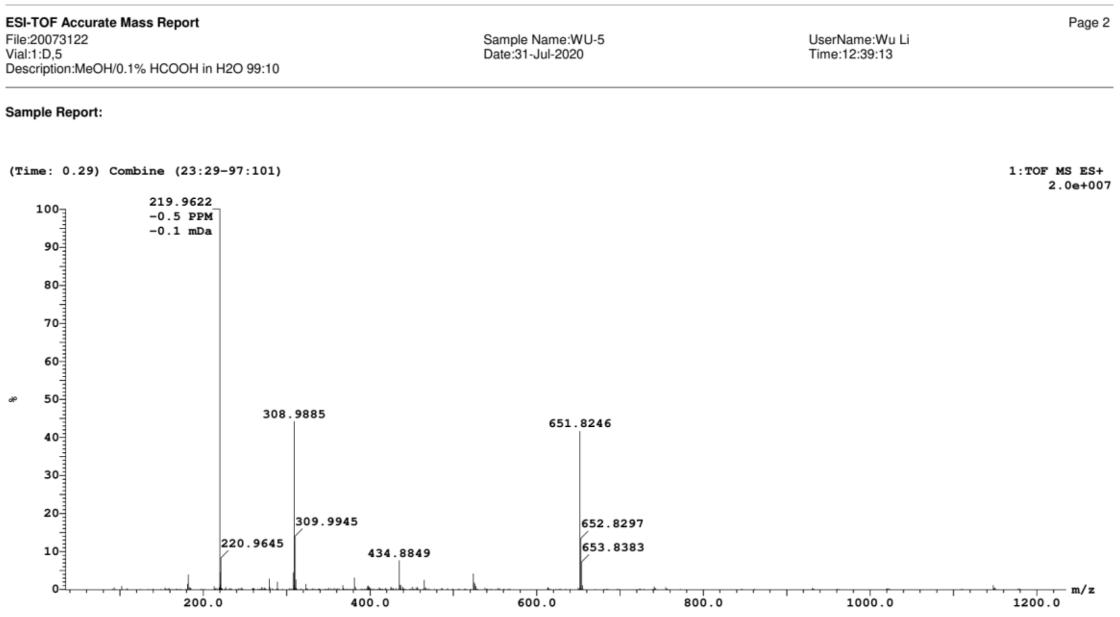

HRMS (ESI-TOF) of 7b $[M+H]^+$:

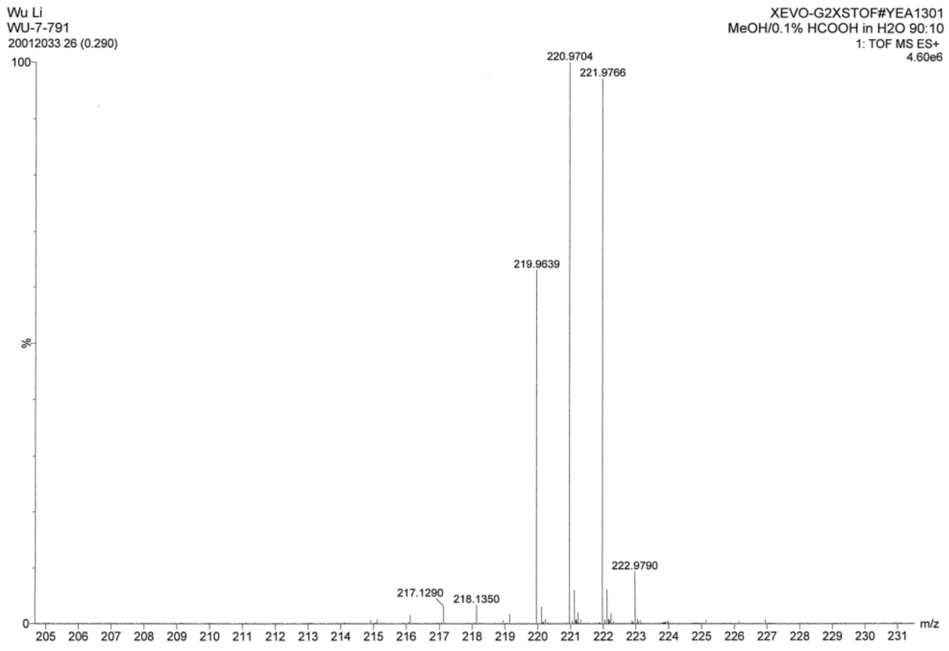

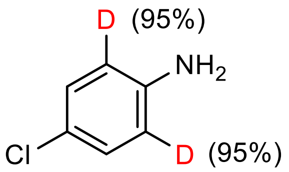

8b

According to GP, Fe-Cellulose-1000 (62 mg, 0.05 mol), substrate (40 mg, 0.31 mmol), D_2O (1.5 mL), H_2 (20 bar), room temperature to 120 °C and then at 120 °C for 24 h. The product **8b** (35 mg, 0.27 mmol, 87%) was obtained.

^{1}H NMR (400 MHz, DMSO-d_6) δ 7.00 (s, 2H), 6.53 – 6.56 (m, 0.11H), 5.20 (s, 2H).

^{13}C NMR (101 MHz, DMSO-d_6) δ 147.57, 147.52, 128.48, 128.38, 118.67, 115.16, 114.91, 114.67.

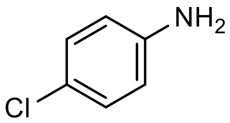

Chemical Formula: C_6H_6ClN
Exact Mass: 127.0189

HRMS (ESI-TOF) of 8a:

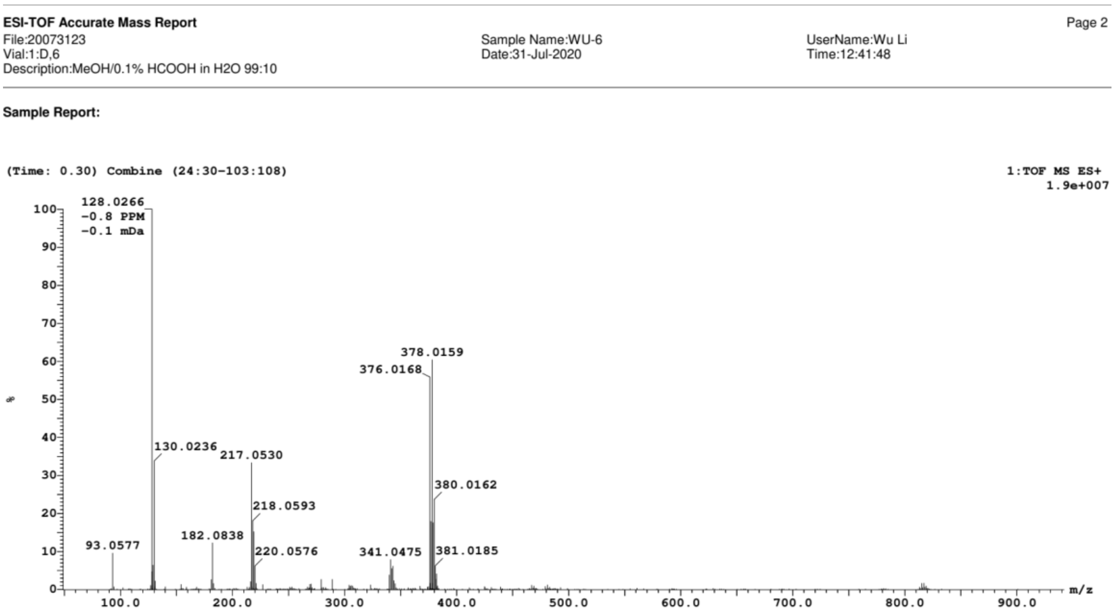

HRMS (ESI-TOF) of 8b $[M+H]^+$:

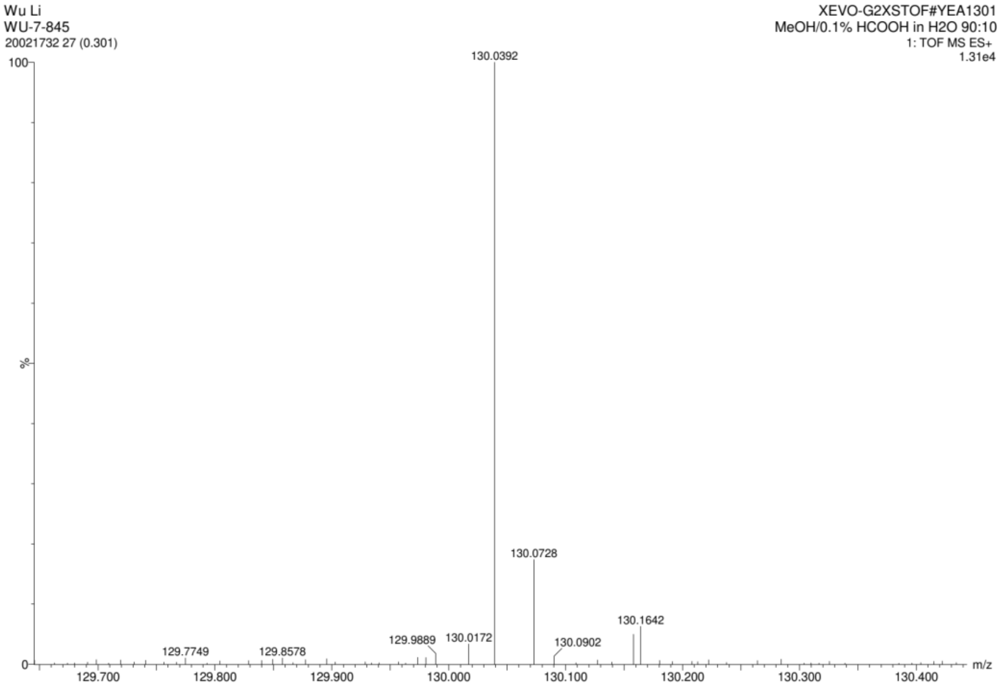

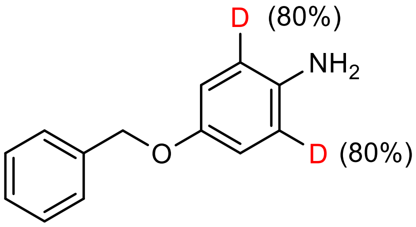

9b

According to GP, Fe-Cellulose-1000 (62 mg, 0.05 mol), substrate (57 mg, 0.29 mmol), D_2O (1.5 mL), H_2 (20 bar), room temperature to 120 °C and then at 120 °C for 24 h. The product **9b** (52 mg, 0.26 mmol, 90%) was obtained.

^{1}H NMR (300 MHz, DMSO-*d*$_6$) δ 7.58 – 7.17 (m, 5H), 6.73 (s, 2H), 6.53 – 6.50 (m, 0.40 H), 4.94 (s, 2H), 4.64 (s, 2H).

^{13}C NMR (75 MHz, DMSO-*d*$_6$) δ 149.69, 142.54, 142.48, 137.82, 128.31, 127.56, 115.70, 115.61, 114.90, 69.80.

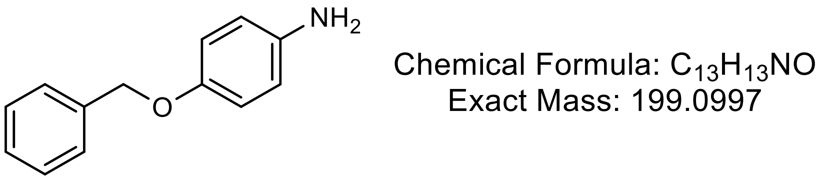

HRMS (ESI-TOF) of 9a:

ESI-TOF Accurate Mass Report Page 2
File:20073124 Sample Name:WU-7 UserName:Wu Li
Vial:1:D,7 Date:31-Jul-2020 Time:12:44:23
Description:MeOH/0.1% HCOOH in H2O 99:10

Sample Report:

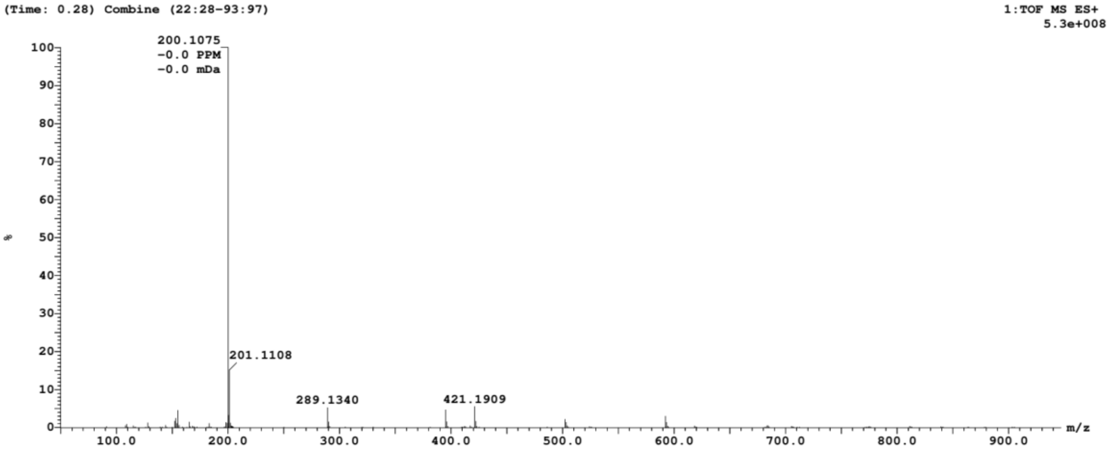

HRMS (ESI-TOF) of 9b $[M+H]^+$:

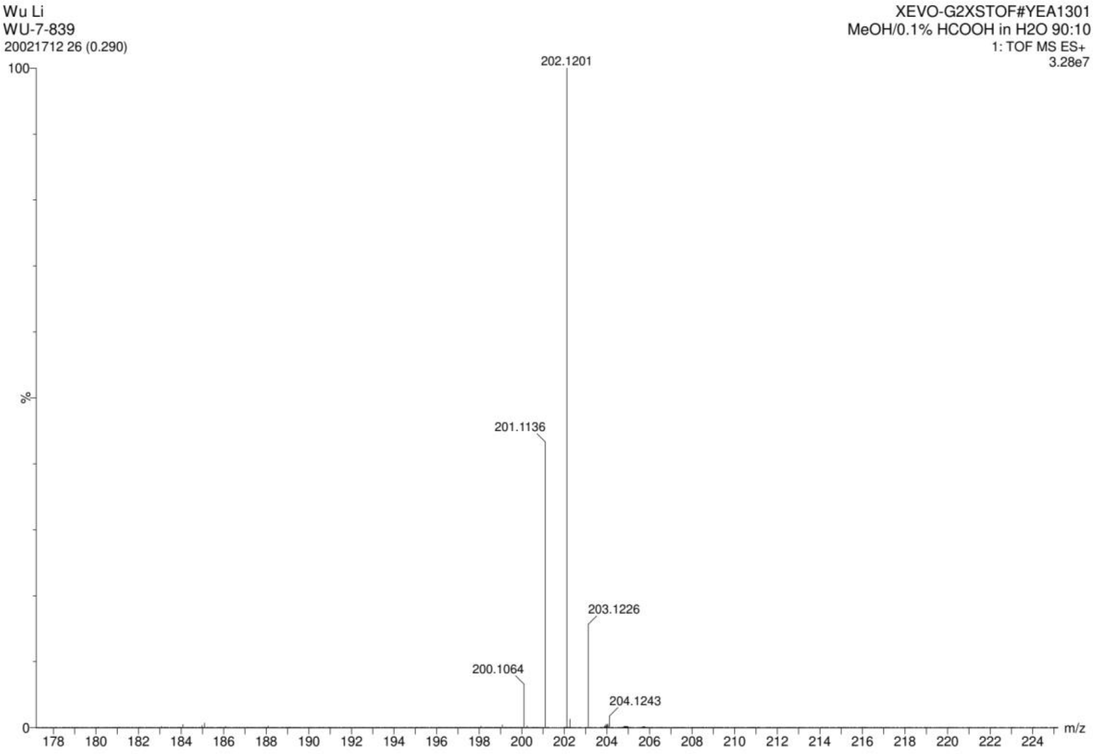

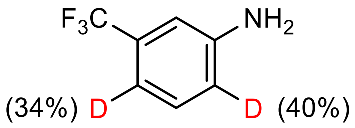

10b

According to GP, Fe-Cellulose-1000 (60 mg, 0.05 mol), substrate (42 mg, 0.26 mmol), D_2O (1.5 mL), H_2 (20 bar), room temperature to 120 °C and then at 120 °C for 24 h. The product **10b** (41 mg, 0.25 mmol, 96%) was obtained.

^{1}H NMR (400 MHz, DMSO-d_6) δ 7.17 (s, 1H), 6.84 (d, J = 2.1 Hz, 0.66H), 6.80 (dd, J = 7.9, 1.3 Hz, 1H), 6.75 (dd, J = 7.7, 1.3 Hz, 0.60H), 5.38 (s, 2H).

^{13}C NMR (101 MHz, DMSO-d_6) δ 149.69, 149.62, 130.74, 130.66, 130.43, 130.35, 130.31, 130.20, 130.12, 129.20, 126.49, 123.79, 121.09, 118.18, 112.68, 112.64, 112.60, 112.56, 110.38, 110.34, 110.30.

^{19}F NMR (282 MHz, DMSO-d_6) δ -61.71 (d, J = 2.6 Hz).

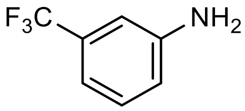

Chemical Formula: $C_7H_6F_3N$
Exact Mass: 161.0452

HRMS (ESI-TOF) of 10a:

ESI-TOF Accurate Mass Report Page 2
File:20073125 Sample Name:WU-8 UserName:Wu Li
Vial:1:D,8 Date:31-Jul-2020 Time:12:46:58
Description:MeOH/0.1% HCOOH in H2O 99:10

Sample Report:

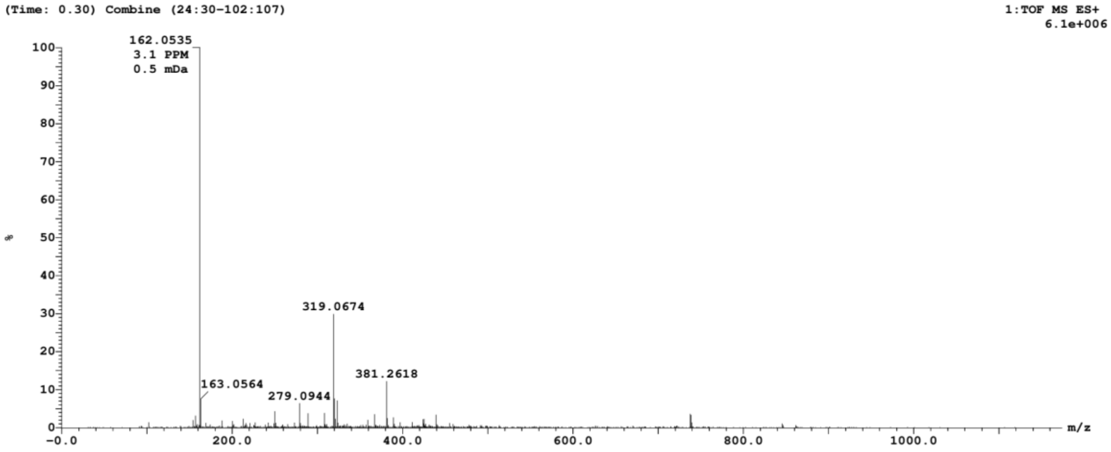

HRMS (ESI-TOF) of 10b [M+H]+:

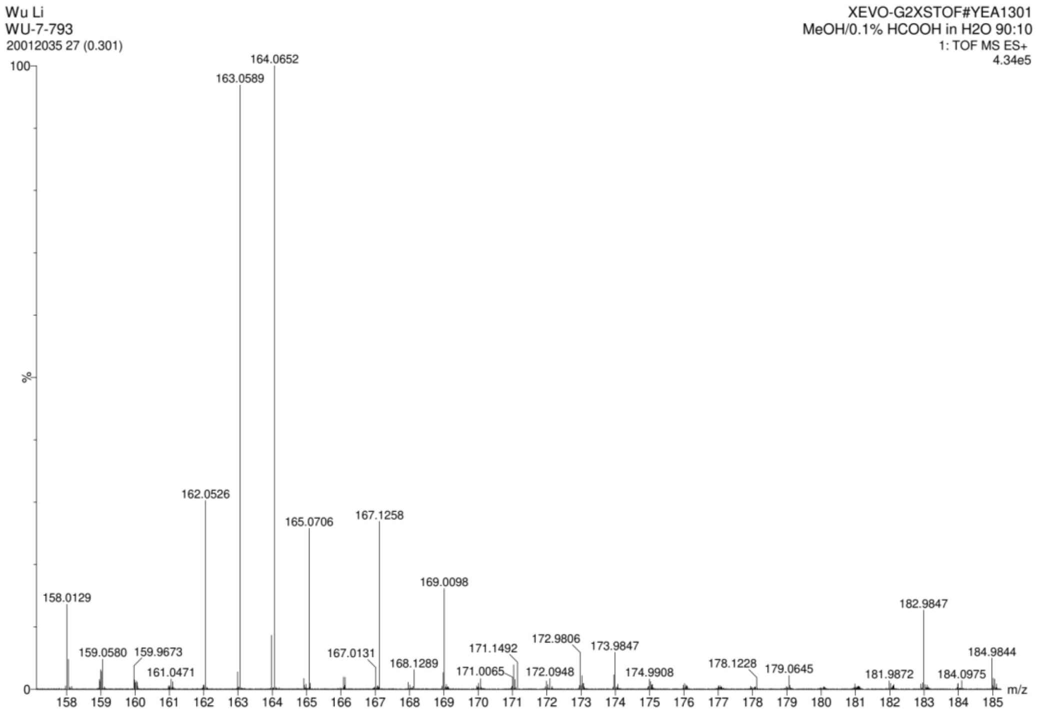

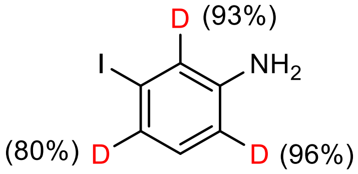

11b

According to GP, Fe-Cellulose-1000 (61 mg, 0.05 mol), substrate (60 mg, 0.28 mmol), D_2O (1.5 mL), H_2 (20 bar), room temperature to 120 °C and then at 120 °C for 24 h. The product **11b** (55 mg, 0.26 mmol, 93%) was obtained.

^{1}H NMR (300 MHz, $CDCl_3$) δ 7.17 – 7.16 (m, 0.20 H), 7.09 – 7.05 (m, 0.07H), 6.87 (q, J = 1.1 Hz, 1H), 6.64-6.61 (m, 0.04H), 3.47 (s, 2H).

^{13}C NMR (75 MHz, $CDCl_3$) δ 147.49, 130.63, 129.19,127.08, 124.40, 94.76.

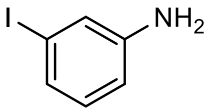

Chemical Formula: C_6H_6IN
Exact Mass: 218.9545

HRMS (ESI-TOF) of 11a:

ESI-TOF Accurate Mass Report Page 2

File:20073126 Sample Name:WU-9 UserName:Wu Li

Vial:1:E,1 Date:31-Jul-2020 Time:12:59:53

Description:MeOH/0.1% HCOOH in H2O 99:10

Sample Report:

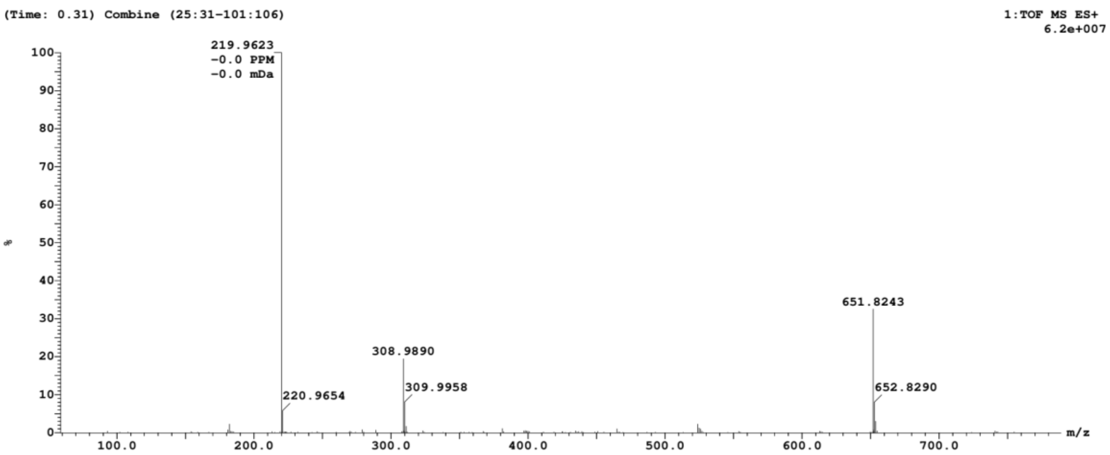

HRMS (ESI-TOF) of 11b $[M+H]^+$:

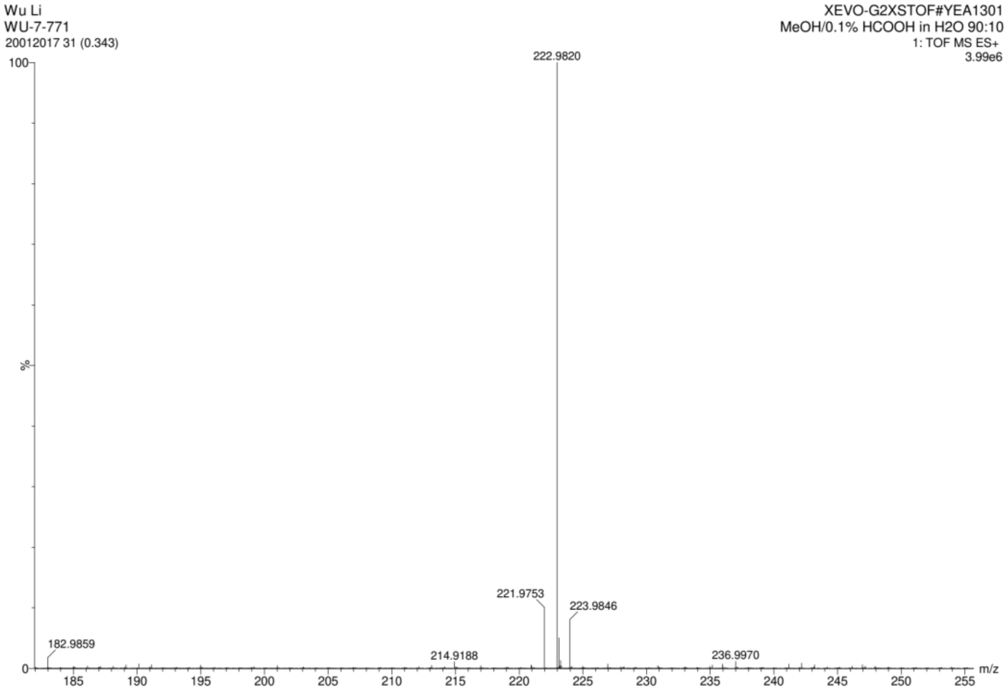

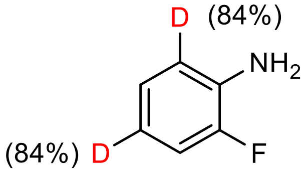

12b

According to GP, Fe-Cellulose-1000 (60 mg, 0.05 mol), substrate (45 mg, 0.41 mmol), D_2O (1.5 mL), H_2 (20 bar), room temperature to 120 °C and then at 120 °C for 24 h. The product **12a** (42 mg, 0.37 mmol, 90%) was obtained.

^{1}H NMR (300 MHz, $CDCl_3$) δ 7.11 – 6.87 (m, 2H), 6.81 – 6.75 (m, 0.32H), 3.32 (s, 2H).

^{13}C NMR (101 MHz, DMSO-d_6) δ 152.77, 150.43, 136.65, 136.59, 136.53, 136.46, 125.27, 125.24, 125.16, 125.13, 117.55, 117.49, 117.39, 117.35, 117.25, 117.08, 115.60, 115.52, 115.42.

^{19}F NMR (282 MHz, DMSO-d_6) δ -135.38.

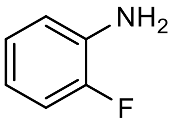

Chemical Formula: C_6H_6FN
Exact Mass: 111.0484

HRMS (ESI-TOF) of 12a:

ESI-TOF Accurate Mass Report Page 2
File:20073127 Sample Name:WU-10 UserName:Wu Li
Vial:1:E,2 Date:31-Jul-2020 Time:13:02:24
Description:MeOH/0.1% HCOOH in H2O 99:10

Sample Report:

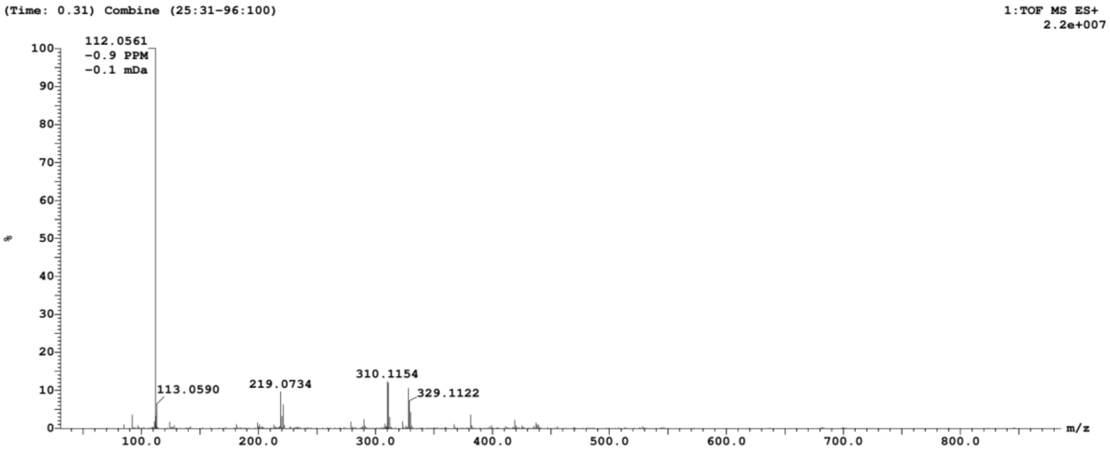

HRMS (ESI-TOF) of 12b [M+H]+:

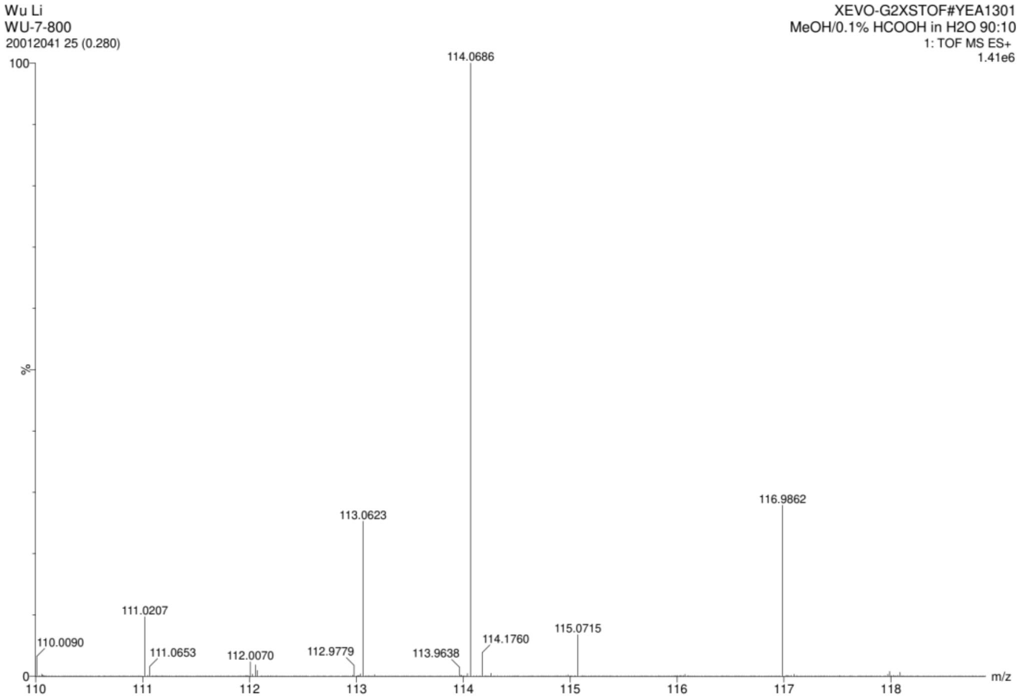

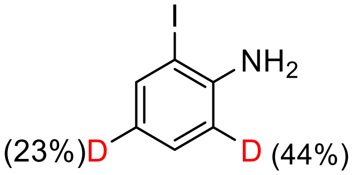

13b

According to GP, Fe-Cellulose-1000 (60 mg, 0.05 mol), substrate (53 mg, 0.24 mmol), D_2O (1.5 mL), H_2 (20 bar), room temperature to 120 °C and then at 120 °C for 24 h. The product **13b** (47 mg, 0.21 mmol, 88%) was obtained.

^{1}H NMR (300 MHz, $CDCl_3$) δ 7.70 – 7.59 (m, 1H), 7.21 – 7.09 (m, 1H), 6.75 (dt, J = 8.0, 0.8 Hz, 0.76H), 6.53 – 6.42 (m, 0.56H), 4.03 (s, 2H).

^{13}C NMR (75 MHz, $CDCl_3$) δ 146.86, 139.11, 139.01, 129.45, 129.35, 129.24, 120.09, 114.85, 84.29.

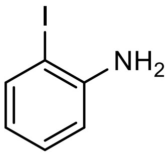

Chemical Formula: C_6H_6IN
Exact Mass: 218.9545

HRMS (ESI-TOF) of 13a:

ESI-TOF Accurate Mass Report Page 2

File:20073128 Sample Name:WU-11 UserName:Wu Li

Vial:1:E,3 Date:31-Jul-2020 Time:13:04:58

Description:MeOH/0.1% HCOOH in H2O 99:10

Sample Report:

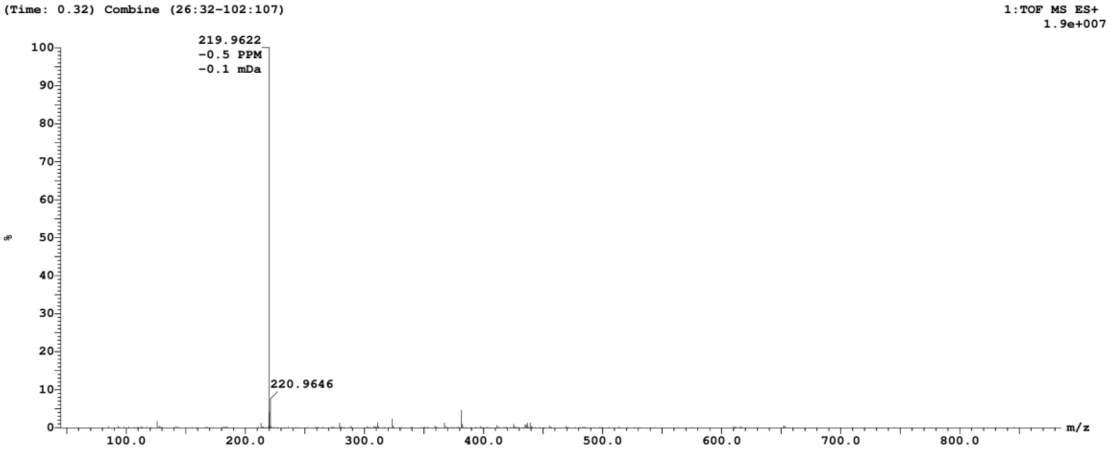

HRMS (EI) of 13b $[M]^+$:

WU-7-766 HR (EI)

File : D:\Xcalibur\data\2001\20012405aeihr-av3.RAW

Full ms [200.500 - 235.500] - Range: 219.000 - 230.000

Scan No. 1 of 1

Mass	Absolute Intensity	Relative Intensity	Theoretical Mass	Delta [ppm]	Delta [mmu]	RDB	Composition
219.96045	8203779	82.7	219.96022	1.0	0.2	4.0	$C_6 H_5 {}^2H_1 N_1 I_1$
220.96568	2501553	25.2	220.96650	-3.7	-0.8	4.0	$C_6 H_4 {}^2H_2 N_1 I_1$

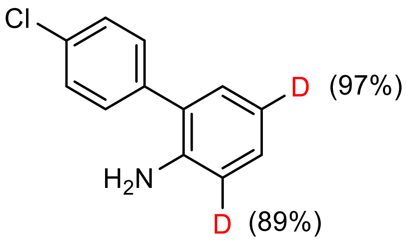

14b

According to GP, Fe-Cellulose-1000 (62 mg, 0.05 mol), substrate (52 mg, 0.26 mmol), D_2O (1.5 mL), H_2 (20 bar), room temperature to 120 °C and then at 120 °C for 24 h. The product **14b** (51 mg, 0.25 mmol, 88%) was obtained.

^{1}H NMR (400 MHz, DMSO-d_6) δ 7.46 (q, J = 8.2 Hz, 4H), 7.01 (d, J = 32.3 Hz, 2H), 6.78 – 7.76 (m, 0.11H), 6.65 – 6.63 (m, 0.03H), 4.82 (s, 2H).

^{13}C NMR (101 MHz, DMSO-d_6) δ 145.03, 138.56, 131.36, 130.52, 129.85, 128.67, 128.40, 128.29, 124.37, 116.75, 116.51, 116.26, 115.40, 115.13.

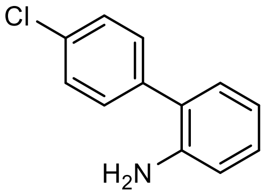

Chemical Formula: $C_{12}H_{10}ClN$
Exact Mass: 203.0502

HRMS (ESI-TOF) of 14a:

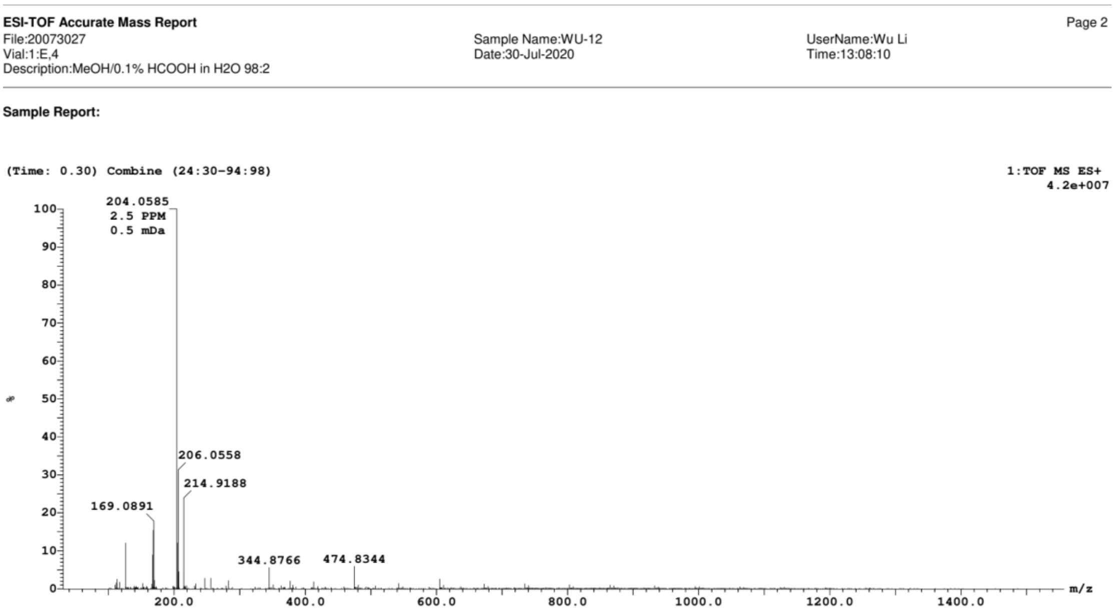

HRMS (ESI-TOF) of 14b $[M+H]^+$:

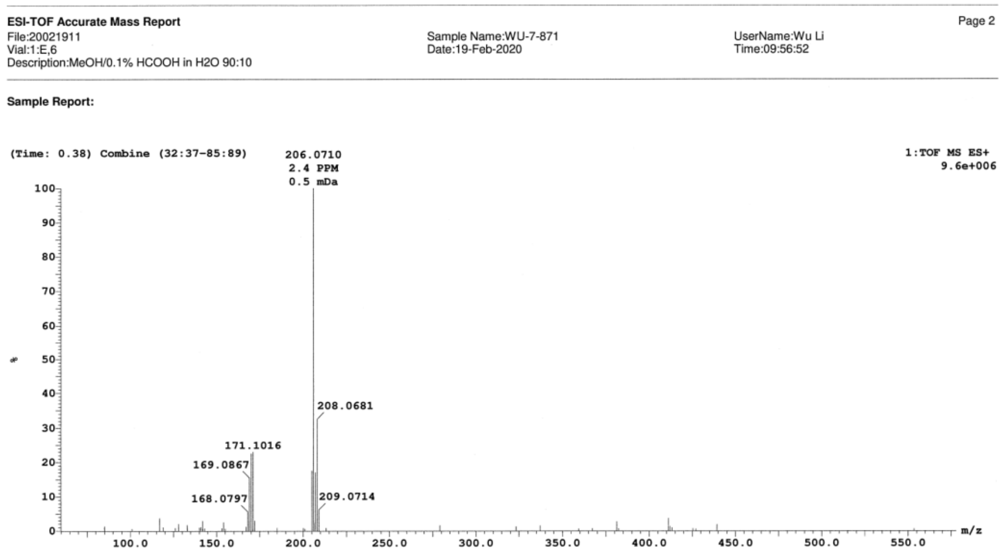

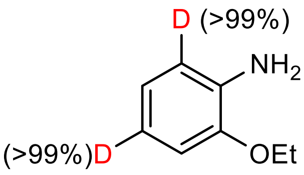

15b

According to GP, Fe-Cellulose-1000 (62 mg, 0.05 mol), substrate (42 mg, 0.31 mmol), D_2O (1.5 mL), H_2 (20 bar), room temperature to 120 °C and then at 120 °C for 24 h. The product **15b** (40 mg, 0.29 mmol, 94%) was obtained.

^{1}H NMR (300 MHz, $CDCl_3$) δ 6.79 (s, 2H), 4.07 (q, J = 7.0 Hz, 2H), 3.68 (s, 2H), 1.45 (t, J = 7.0 Hz, 3H).

^{13}C NMR (75 MHz, $CDCl_3$) δ 146.76, 136.29, 120.86, 118.74, 117.19, 115.22, 111.49, 63.84, 15.12.

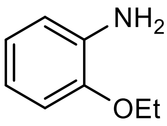

Chemical Formula: $C_8H_{11}NO$
Exact Mass: 137.0841

HRMS (ESI-TOF) of 15a:

ESI-TOF Accurate Mass Report
File:20073132
Vial:1:E,5
Description:MeOH/0.1% HCOOH in H2O 99:10
Sample Name:WU-13
Date:31-Jul-2020
UserName:Wu Li
Time:13:21:15
Page 2

Sample Report:

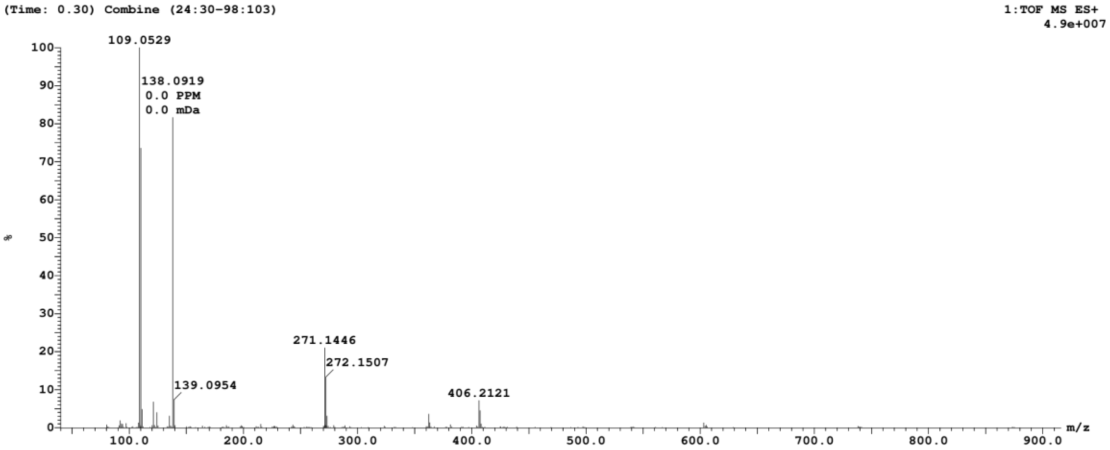

HRMS (ESI-TOF) of 15b $[M+H]^+$:

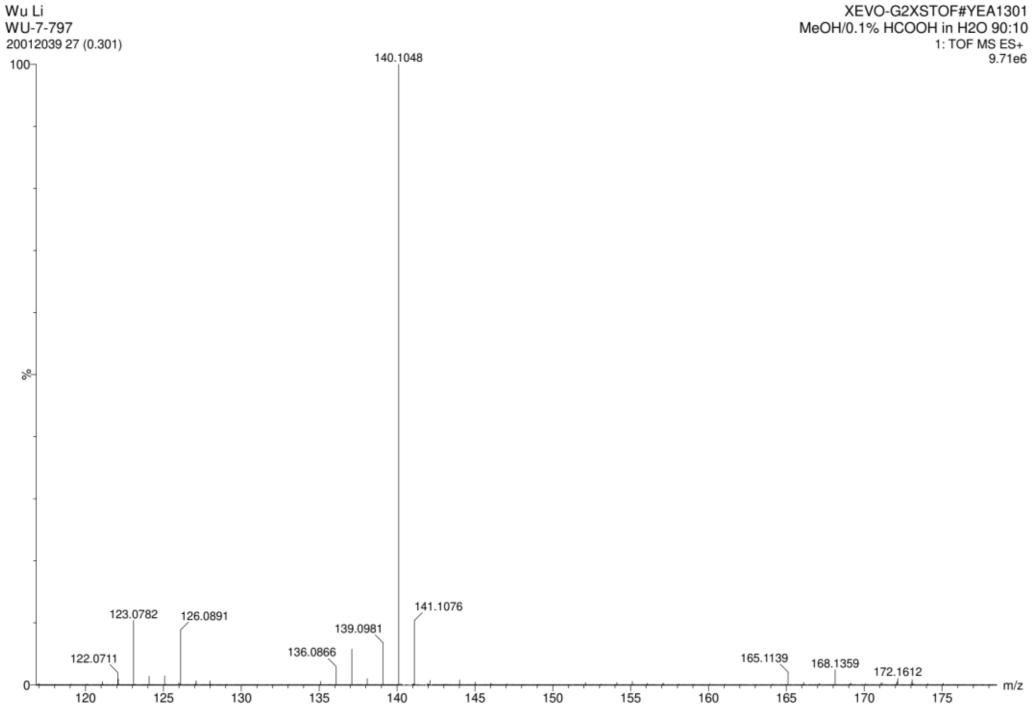

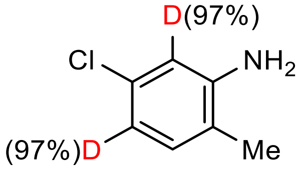

16b

According to GP, Fe-Cellulose-1000 (61 mg, 0.05 mol), substrate (35 mg, 0.25 mmol), D_2O (1.5 mL), H_2 (20 bar), room temperature to 120 °C and then at 120 °C for 24 h. The product **16b** (30 mg, 0.21 mmol, 84%) was obtained.

^{1}H NMR (300 MHz, $CDCl_3$) δ 7.04 – 6.88 (m, 1H), 6.68 – 6.65 (m, 0.07H), 3.64 (s, 2H), 2.12 (d, *J* = 0.8 Hz, 3H).

^{13}C NMR (75 MHz, DMSO-d_6) δ 148.56, 148.51, 148.46, 131.46, 130.86, 120.29, 113.31, 113.02, 112.70, 17.31.

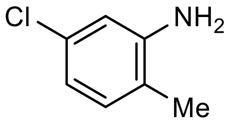

Chemical Formula: C_7H_8ClN
Exact Mass: 141.0345

HRMS (ESI-TOF) of 16b [M+H]$^+$:

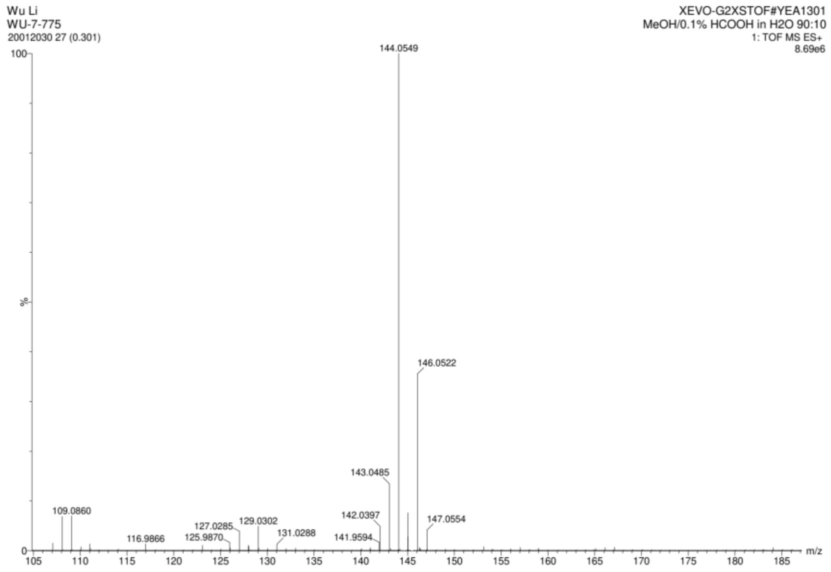

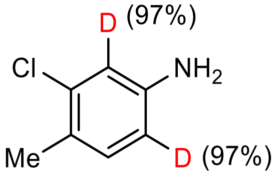

17b

According to GP, Fe-Cellulose-1000 (61 mg, 0.05 mol), substrate (40 mg, 0.28 mmol), D_2O (1.5 mL), H_2 (20 bar), room temperature to 120 °C and then at 120 °C for 24 h. The product **17b** (38 mg, 0.26 mmol, 92%) was obtained.

^{1}H NMR (400 MHz, DMSO-d_6) δ 6.93 (s, 1H), 6.93 (s, 1H), 6.60 (s, 0.03H), 6.44 – 6.42 (m, 0.03H), 2.13 (s, 3H).

^{13}C NMR (101 MHz, DMSO-d_6) δ 148.01, 133.15, 131.16, 121.18, 113.65, 113.40, 113.16, 112.92, 112.67, 112.44, 18.43.

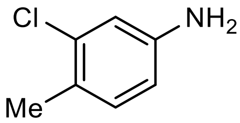

Chemical Formula: C_7H_8ClN
Exact Mass: 141.0345

HRMS (ESI-TOF) of 17a:

ESI-TOF Accurate Mass Report Page 2
File:20073030 Sample Name:WU-15 UserName:Wu Li
Vial:1:E,7 Date:30-Jul-2020 Time:13:22:03
Description:MeOH/0.1% HCOOH in H2O 98:2

Sample Report:

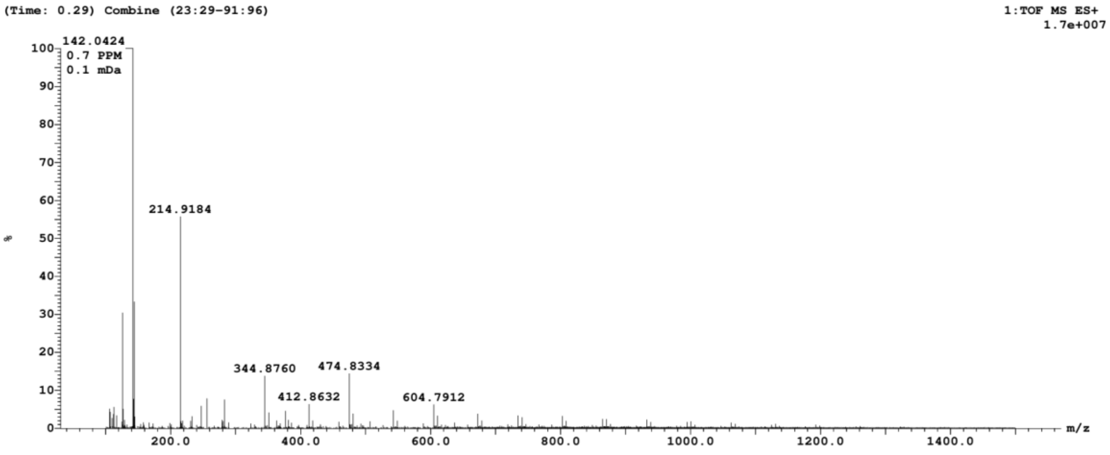

HRMS (ESI-TOF) of 17b [M+H]⁺:

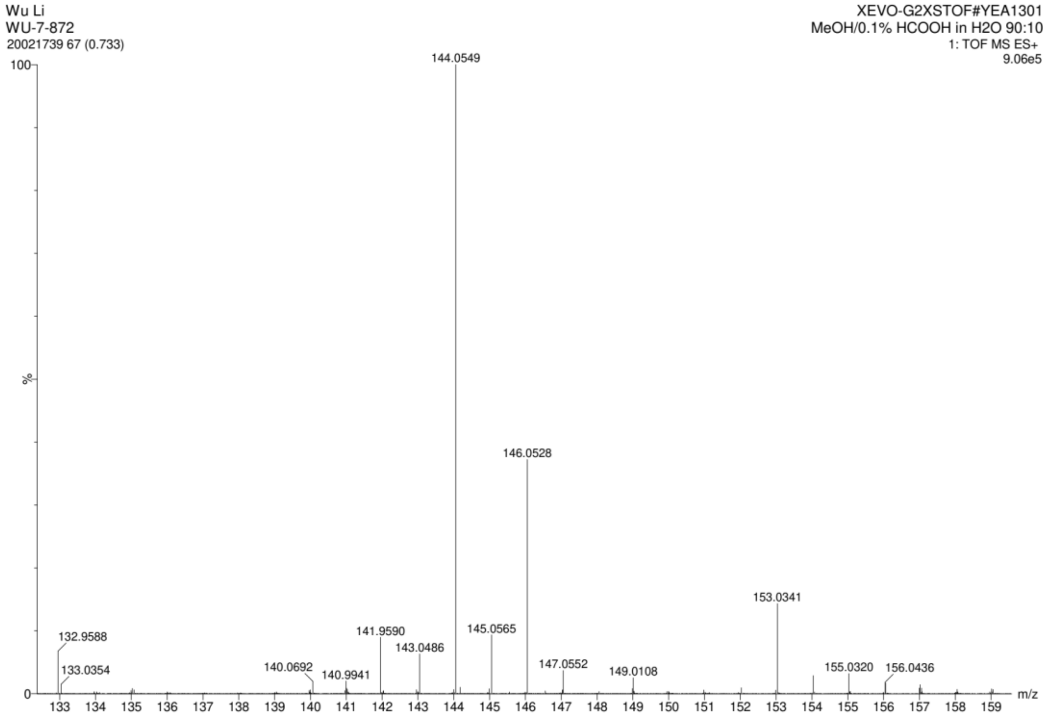

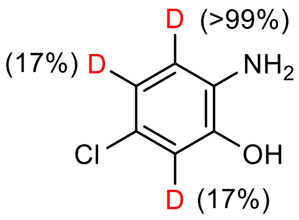

18b

According to GP, Fe-Cellulose-1000 (60 mg, 0.05 mol), substrate (39 mg, 0.27 mmol), D_2O (1.5 mL), H_2 (20 bar), room temperature to 120 °C and then at 120 °C for 24 h. The product **18b** (40 mg, 0.27 mmol, >99%) was obtained. Internal standard trimethoxybenzene was also used to confirm the deuterium content.

^{1}H NMR (300 MHz, DMSO-d_6) δ 9.47 (s, 1H), 6.56 (s, 1.67H), 4.62 (s, 2H).

^{13}C NMR (101 MHz, DMSO-d_6) δ 145.15, 136.26, 136.19, 119.40, 119.23, 119.14, 115.20, 115.09, 114.40, 114.15.

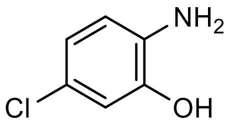

Chemical Formula: C_6H_6ClNO
Exact Mass: 143.0138

HRMS (ESI-TOF) of 18a:

ESI-TOF Accurate Mass Report
File:20073116
Vial:1:E,8
Description:MeOH/0.1% HCOOH in H2O 99:10

Sample Name:WU-16
Date:31-Jul-2020

UserName:Wu Li
Time:12:23:45

Page 2

Sample Report:

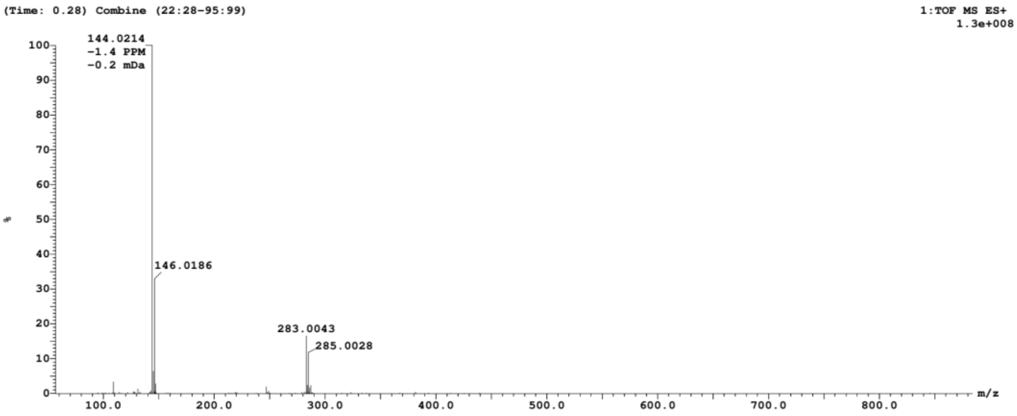

HRMS (EI) of 18b [M]$^+$

Wu-7-768

File : D:\Xcalibur\data\2001\20012406eihr-av3.RAW
Full ms [127.500 - 158.500] - Range: 145.000 - 148.000
Scan No. 1 of 1

Mass	Absolute Intensity	Relative Intensity	Theoretical Mass	Delta [ppm]	Delta [mmu]	RDB	Composition
145.02532	21383680	100.0	145.02580	-3.3	-0.5	4.0	$C_6 H_4 {}^2H_2 O_1 N_1 Cl_1$
146.03115	14798592	69.2					
147.02342	7171108	33.5	147.02285	3.9	0.6	4.0	$C_6 H_4 {}^2H_2 O_1 N_1 {}^{37}Cl_1$

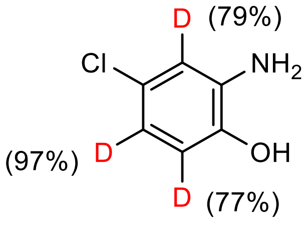

19b

According to GP, Fe-Cellulose-1000 (61 mg, 0.05 mol), substrate (45 mg, 0.31 mmol), D_2O (1.5 mL), H_2 (20 bar), room temperature to 120 °C and then at 120 °C for 24 h. The product **19b** (46 mg, 0.31 mmol, >99%) was obtained.

^{1}H NMR (300 MHz, DMSO-d_6) δ 9.23 (s, 1H), 6.60 – 6.58 (d, J = 4.3 Hz, 0.43H), 6.37 (s, 0.03H), 4.79 (s, 2H).

^{13}C NMR (101 MHz, DMSO-d_6) δ 143.25, 143.11, 138.73, 123.17, 115.53, 115.33, 115.04, 113.69, 113.42.

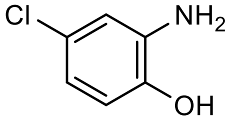

Chemical Formula: C_6H_6ClNO
Exact Mass: 143.0138

HRMS (ESI-TOF) of 19a:

ESI-TOF Accurate Mass Report Page 2
File:20073117 Sample Name:WU-17 UserName:Wu Li
Vial:1:F,1 Date:31-Jul-2020 Time:12:26:20
Description:MeOH/0.1% HCOOH in H2O 99:10

Sample Report:

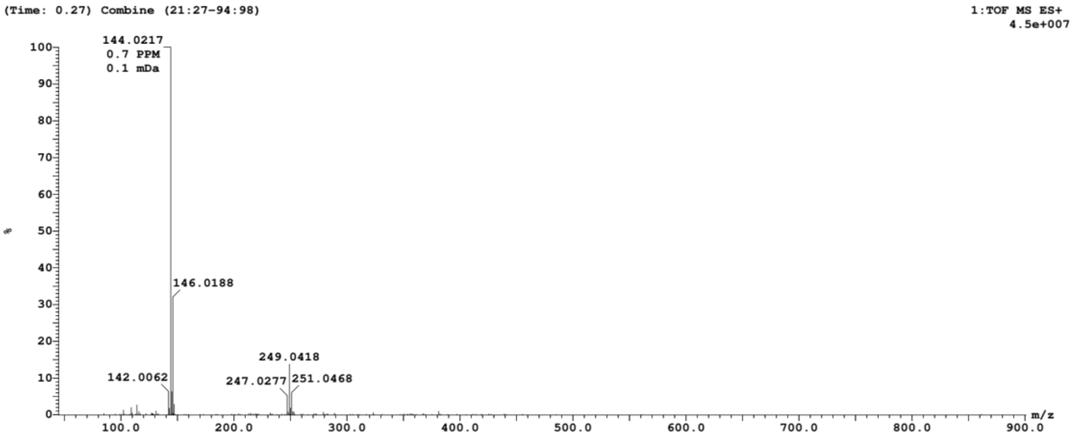

HRMS (EI) of 19b [M]$^+$:

wu-7-765

File : D:\Xcalibur\data\2001\20012404eihr-av3.RAW
Full ms [138.500 - 158.500] - Range: 146.000 - 149.000
Scan No. 1 of 1

Mass	Absolute Intensity	Relative Intensity	Theoretical Mass	Delta [ppm]	Delta [mmu]	RDB	Composition
146.03151	31206144	100.0	146.03207	-3.9	-0.6	4.0	$C_6 H_3 {}^2H_3 O_1 N_1 Cl_1$
147.03541	12429056	39.8					
148.02951	9450240	30.3	148.02912	2.6	0.4	4.0	$C_6 H_3 {}^2H_3 O_1 N_1 {}^{37}Cl_1$

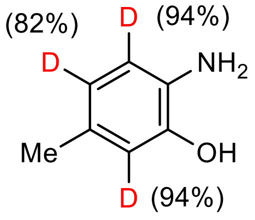

20b

According to GP, Fe-Cellulose-1000 (60 mg, 0.05 mol), substrate (28 mg, 0.23 mmol), D_2O (1.5 mL), H_2 (20 bar), room temperature to 120 °C and then at 120 °C for 24 h. The product **20b** (29 mg, 0.23 mmol, 96%) was obtained.

^{1}H NMR (300 MHz, DMSO-d_6) δ 8.81 (s, 1H), 6.49 – 6.45 (m, 0.18H), 6.36 – 6.35 (m, 0.08 H), 4.24 (s, 1H), 2.09 (s, 3H).

^{13}C NMR (101 MHz, DMSO-d_6) δ 144.36, 134.08, 125.44, 125.35, 120.07, 115.64, 115.36, 114.96, 114.86, 114.60, 20.72.

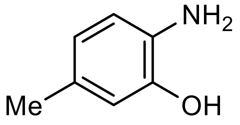

Chemical Formula: C_7H_9NO
Exact Mass: 123.0684

HRMS (ESI-TOF) of 20a:

ESI-TOF Accurate Mass Report
File:20073129
Vial:1:F,2
Description:MeOH/0.1% HCOOH in H2O 99:10

Sample Name:WU-18
Date:31-Jul-2020

UserName:Wu Li
Time:12:57:20

Page 2

Sample Report:

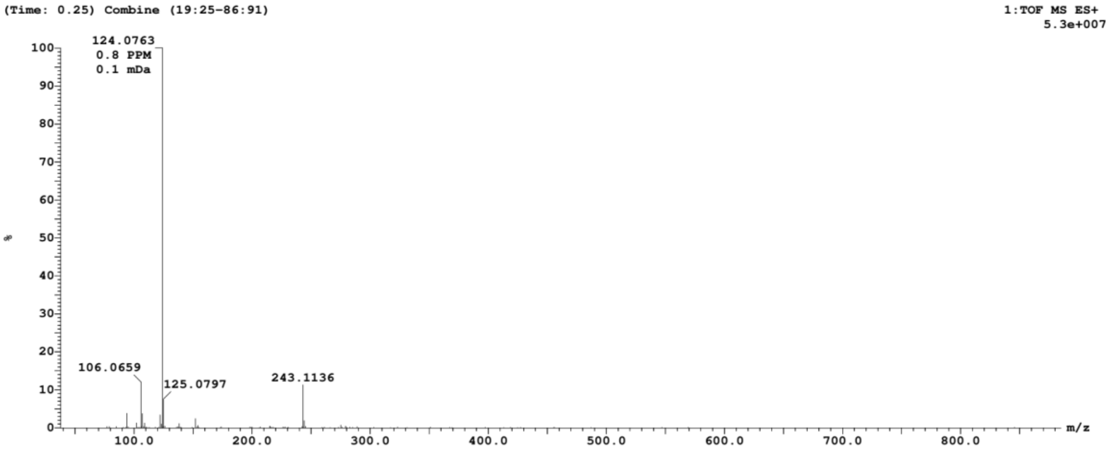

HRMS (ESI-TOF) of 20b [M+H]⁺:

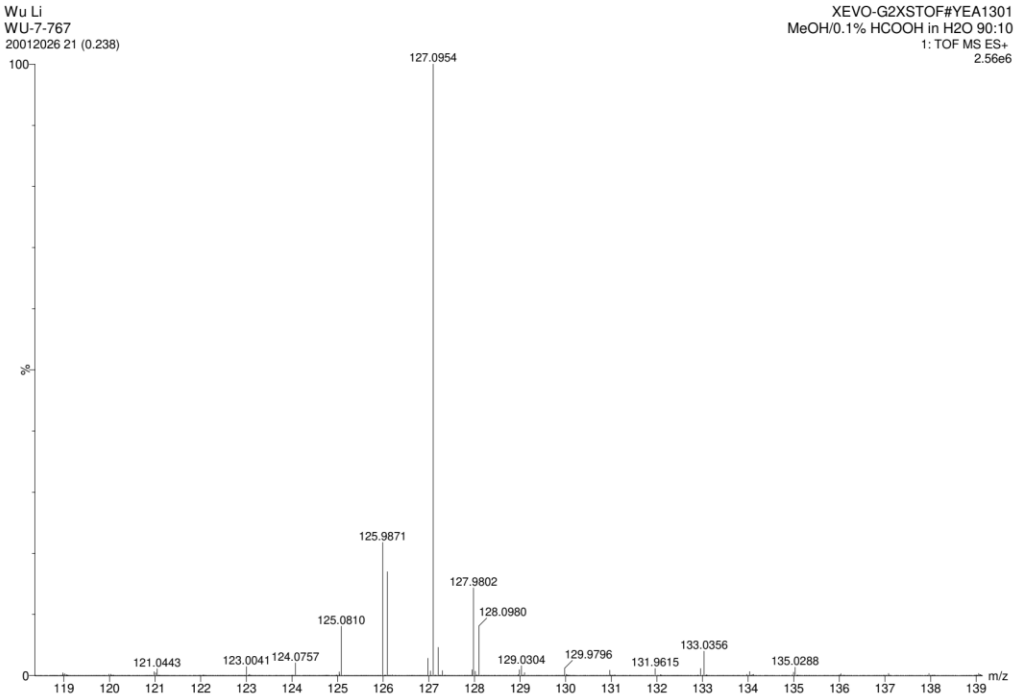

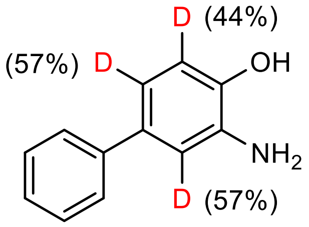

21b

According to GP, Fe-Cellulose-1000 (60 mg, 0.05 mol), substrate (45 mg, 0.24 mmol), D_2O (1.5 mL), H_2 (20 bar), room temperature to 120 °C and then at 120 °C for 24 h. The product **21b** (41 mg, 0.22 mmol, 96%) was obtained.

^{1}H NMR (300 MHz, DMSO-d_6) δ 9.15 (s, 0.31H), 7.86 – 7.13 (m, 5H), 6.92 (s, 0.56H), 6.73 (s, 0.86H), 4.60 (s, 0.7H).

^{13}C NMR (75 MHz, DMSO-d_6) δ 143.88, 141.05, 136.80, 131.67, 128.65, 126.07, 125.86, 114.88, 114.60, 112.62.

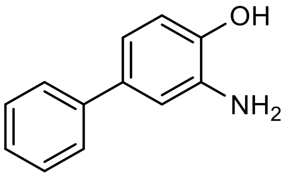

Chemical Formula: $C_{12}H_{11}NO$
Exact Mass: 185.0841

HRMS (ESI-TOF) of 21a:

ESI-TOF Accurate Mass Report Page 2

File:20073133 Sample Name:WU-19 UserName:Wu Li
Vial:1:D,1 Date:31-Jul-2020 Time:13:39:38
Description:MeOH/0.1% HCOOH in H2O 99:10

Sample Report:

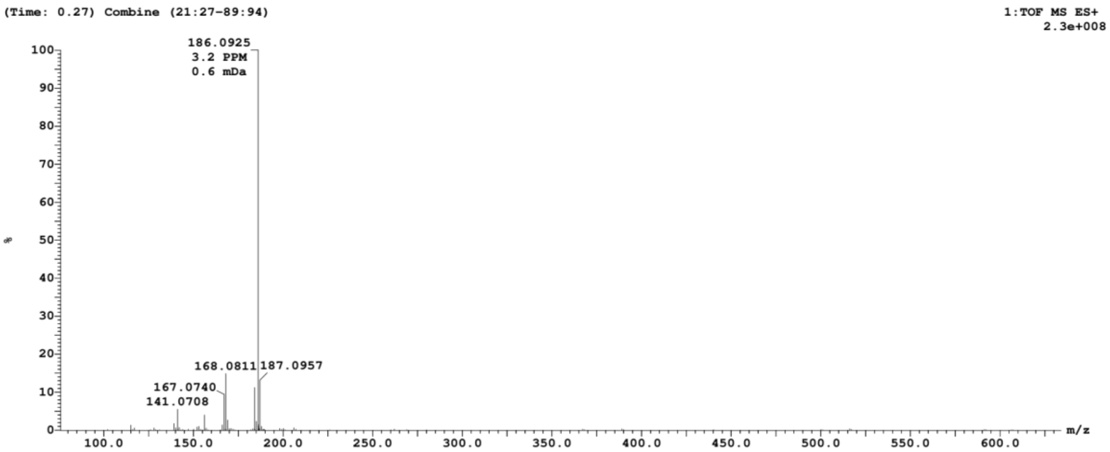

HRMS (ESI-TOF) of 21b $[M+H]^+$:

ESI-TOF Accurate Mass Report Page 2

File:20012037 Sample Name:WU-7-795 UserName:Wu Li
Vial:1:E,5 Date:20-Jan-2020 Time:12:31:12
Description:MeOH/0.1% HCOOH in H2O 90:10

Sample Report:

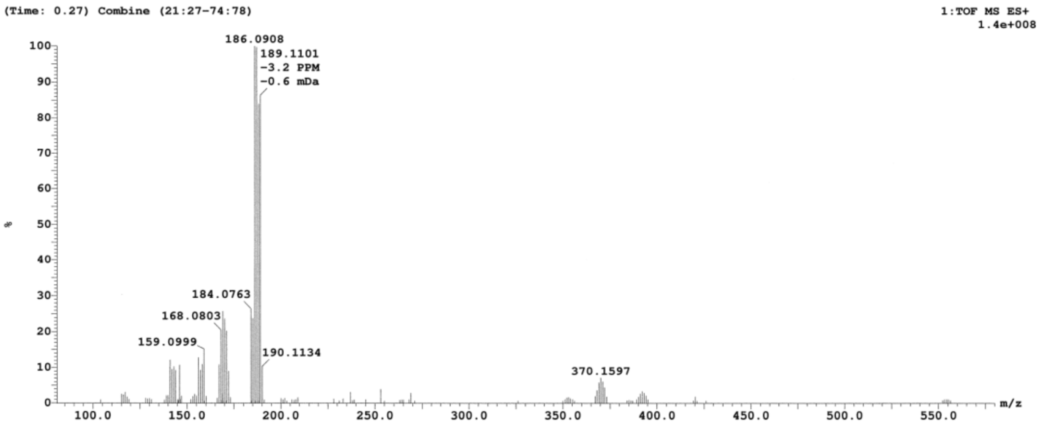

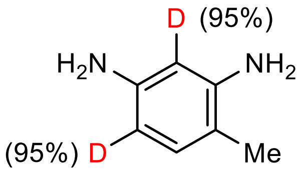

22b

According to GP, Fe-Cellulose-1000 (60 mg, 0.05 mol), substrate (37 mg, 0.30 mmol), D_2O (1.5 mL), H_2 (20 bar), room temperature to 120 °C and then at 120 °C for 24 h. The product **22b** (36 mg, 0.29 mmol, 97%) was obtained.

^1H NMR (300 MHz, DMSO-d_6) δ 6.55 (s, 1H), 5.89 (s, 0.05H), 5.78 – 5.75 (m, 0.05H), 4.41 (d, J = 7.8 Hz, 2H), 1.90 (s, 3H).

^{13}C NMR (75 MHz, DMSO-d_6) δ 146.88, 146.54, 130.02, 109.35, 103.41, 102.74, 100.87, 100.20, 16.60.

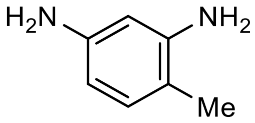

Chemical Formula: $C_7H_{10}N_2$
Exact Mass: 122.0844

HRMS (ESI-TOF) of 22a:

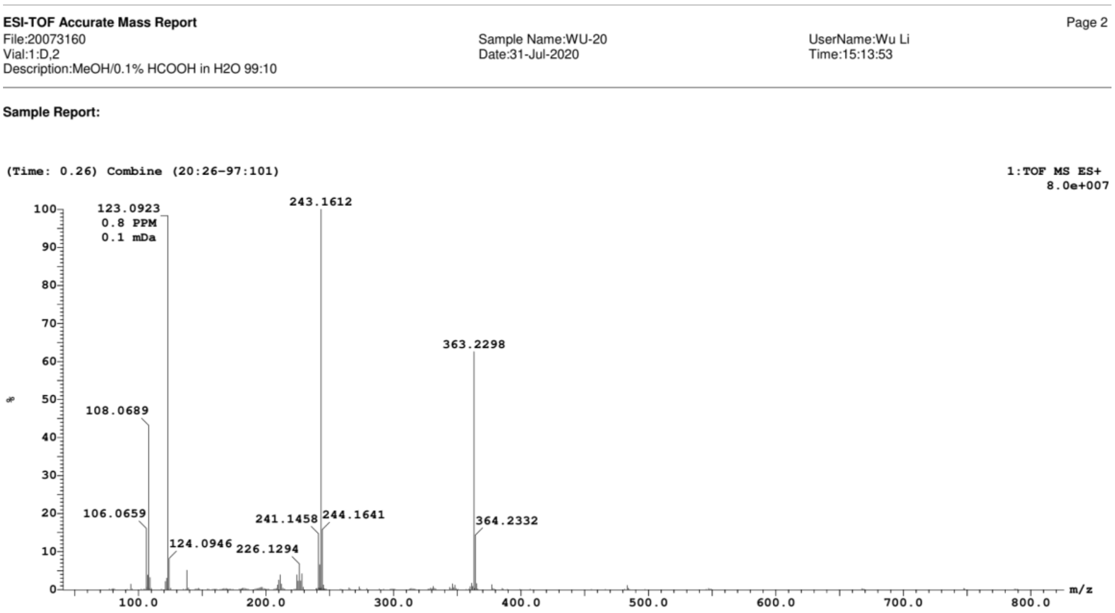

HRMS (ESI-TOF) of 22b [M+H]$^+$

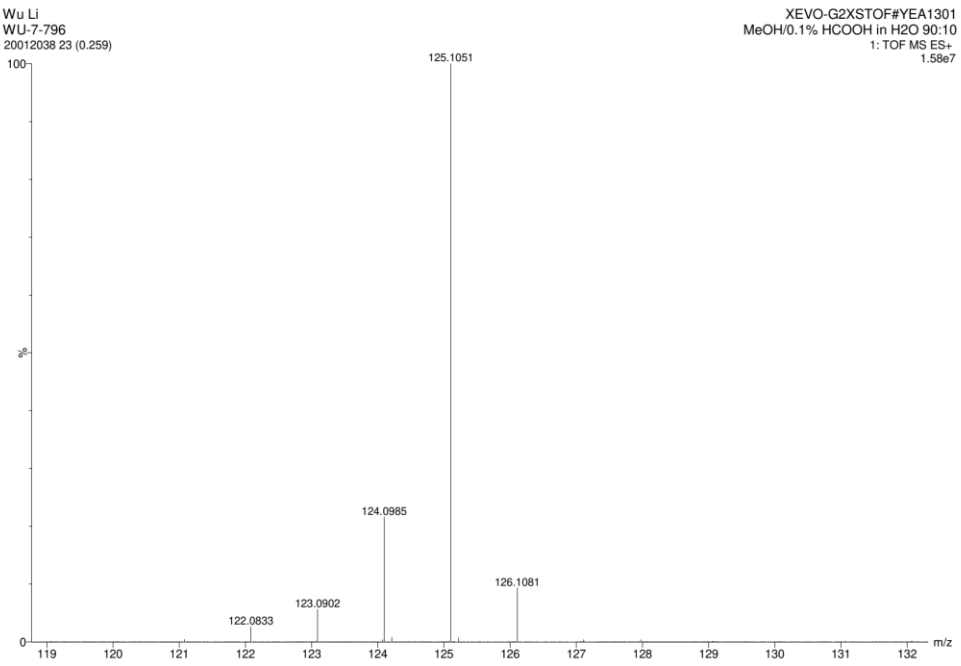

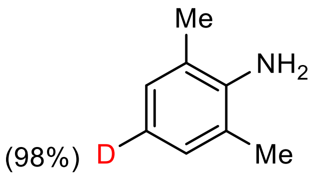

23b

According to GP, Fe-Cellulose-1000 (60 mg, 0.05 mol), substrate (45 mg, 0.37 mmol), D_2O (1.5 mL), H_2 (20 bar), room temperature to 120 °C and then at 120 °C for 24 h. The product **23b** (41 mg, 0.34 mmol, 96%) was obtained.

^{1}H NMR (300 MHz, DMSO-d_6) δ 6.79 (s, 2H), 6.43 (m, 0.02 H), 4.47 (s, 2H), 2.07 (d, J = 0.8 Hz, 6H).

^{13}C NMR (75 MHz, DMSO-d_6) δ 144.06, 144.02, 127.61, 120.50, 115.53, 17.78.

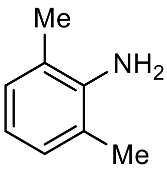

Chemical Formula: $C_8H_{11}N$
Exact Mass: 121.0891

HRMS (ESI-TOF) of 23a:

ESI-TOF Accurate Mass Report
File:20073152
Vial:1:D,3
Description:MeOH/0.1% HCOOH in H2O 99:10
Sample Name:WU-21
Date:31-Jul-2020
UserName:Wu Li
Time:14:47:21
Page 2

Sample Report:

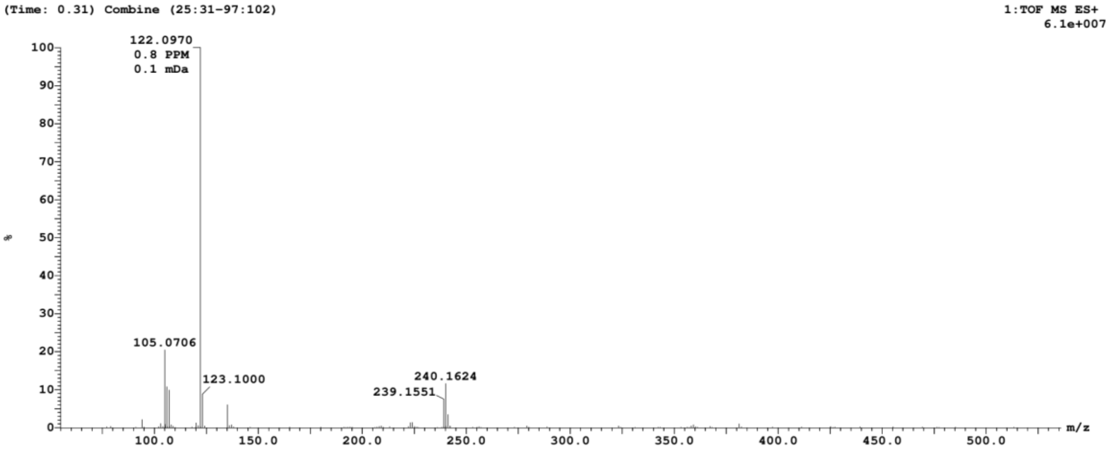

HRMS (ESI-TOF) of 23b $[M+H]^+$

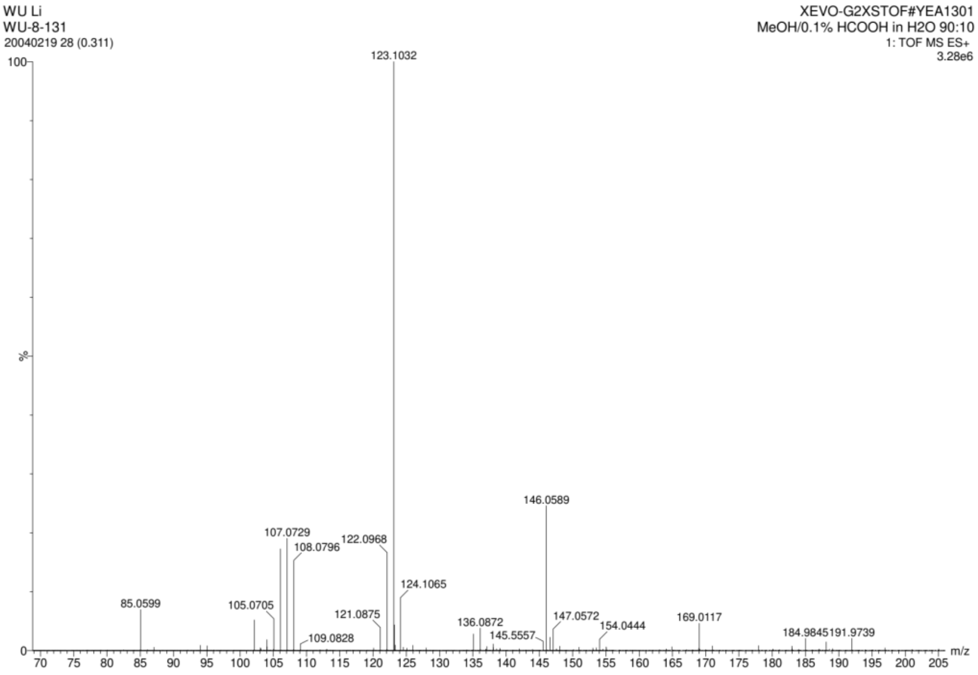

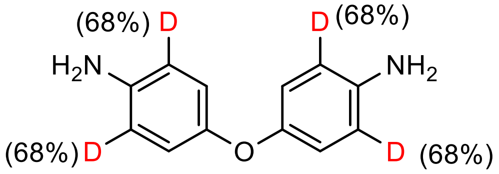

24b

According to GP, Fe-Cellulose-1000 (60 mg, 0.05 mol), substrate (53 mg, 0.27 mmol), D_2O (1.5 mL), H_2 (20 bar), room temperature to 120 °C and then at 120 °C for 24 h. The product **24b** (53 mg, 0.26 mmol, 96%) was obtained.

^{1}H NMR (300 MHz, DMSO-d_6) δ 6.63 (d, J = 3.4 Hz, 4H), 6.53 (d, J = 8.8 Hz, 1.28H), 4.76 (s, 1.15H).

^{13}C NMR (75 MHz, DMSO-d_6) δ 148.47, 144.00, 143.93, 143.87, 118.96, 118.85, 114.82, 114.57, 114.25.

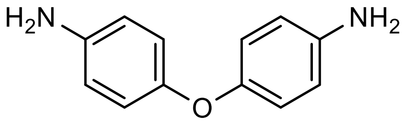

Chemical Formula: $C_{12}H_{12}N_2O$
Exact Mass: 200.0950

HRMS (ESI-TOF) of 24a:

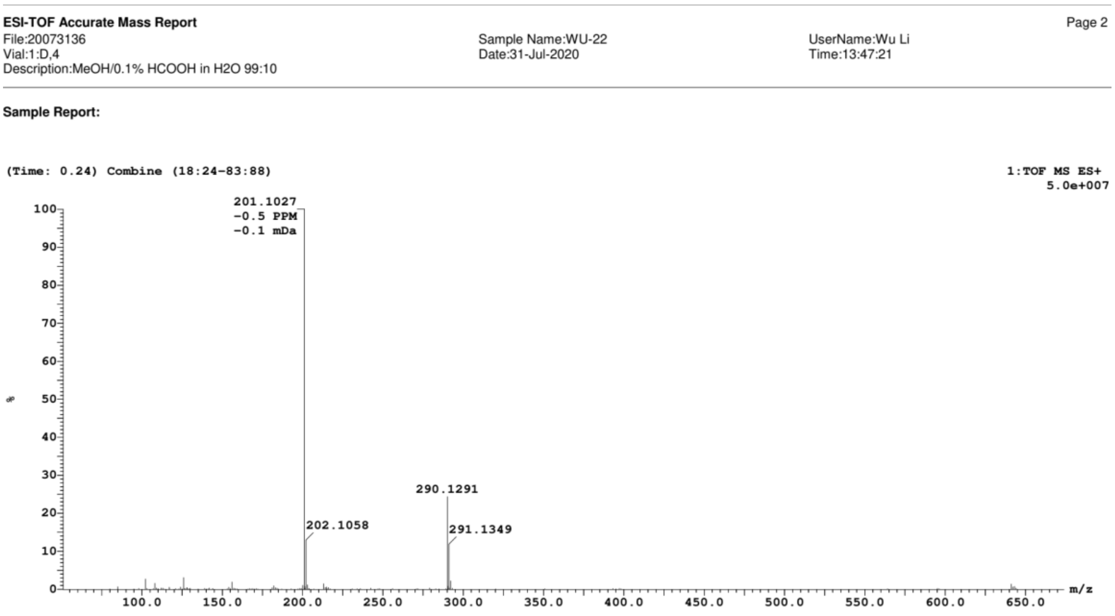

HRMS (ESI-TOF) of 24b [M+H]$^+$:

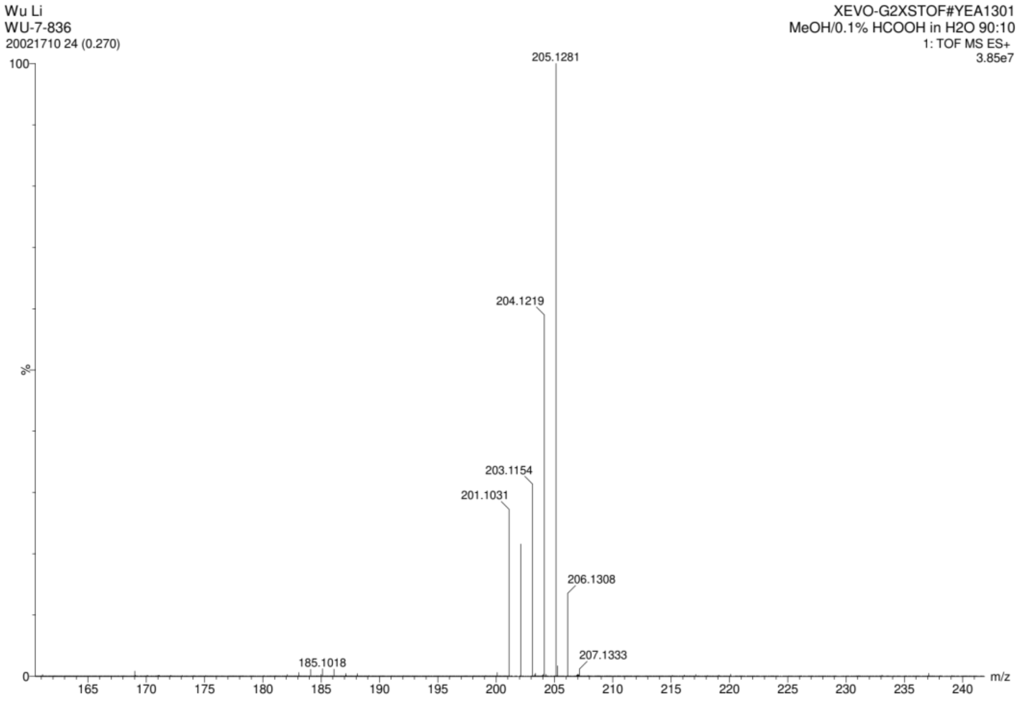

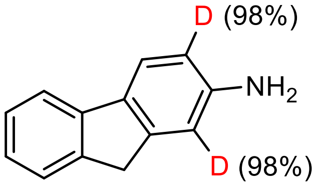

25b

According to GP, Fe-Cellulose-1000 (60 mg, 0.05 mol), substrate (61 mg, 0.34 mmol), D_2O (1.5 mL), H_2 (20 bar), room temperature to 120 °C and then at 120 °C for 24 h. The product **25b** (60 mg, 0.33 mmol, 97%) was obtained.

^{1}H NMR (300 MHz, $CDCl_3$) δ 7.67 (d, *J* = 7.5 Hz, 1H), 7.59 (s, 1H), 7.50 (d, *J* = 7.3 Hz, 1H), 7.36 (t, *J* = 7.4 Hz, 1H), 7.29 – 7.17 (m, 1H), 6.88 (s, 0.02H), 6.74 – 6.71 (m, 0.02H), 3.83 (s, 2H), 3.76 – 3.53 (m, 1H).

^{13}C NMR (75 MHz, $CDCl_3$) δ 145.66, 145.13, 142.37, 142.24, 133.07, 126.72, 126.61, 125.16, 124.84, 124.73, 120.61, 118.67, 114.09, 113.77, 111.61, 36.86.

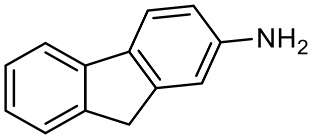

Chemical Formula: $C_{13}H_{11}N$
Exact Mass: 181.0891

HRMS (ESI-TOF) of 25a:

ESI-TOF Accurate Mass Report Page 2

File:20073137 Sample Name:WU-23 UserName:Wu Li

Vial:1:D,5 Date:31-Jul-2020 Time:13:49:57

Description:MeOH/0.1% HCOOH in H2O 99:10

Sample Report:

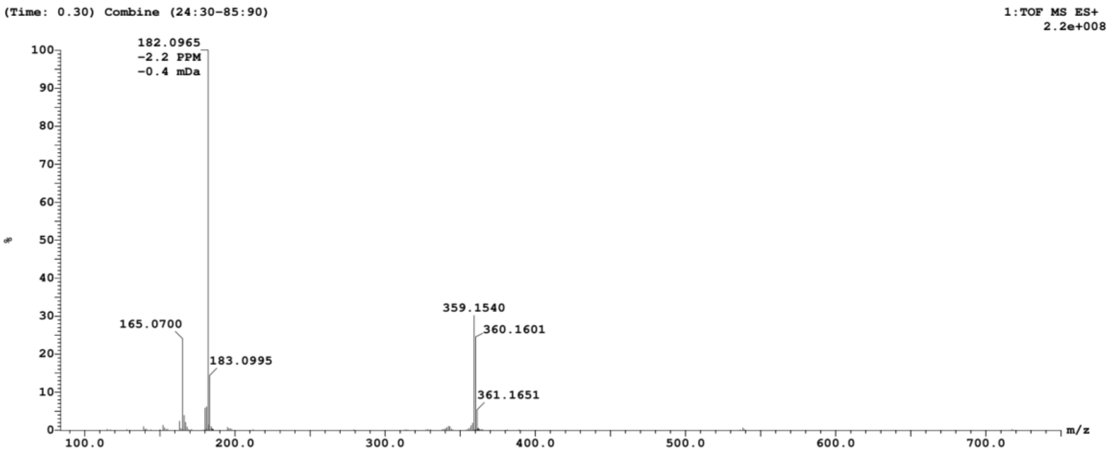

HRMS (ESI-TOF) of 25b $[M+H]^+$

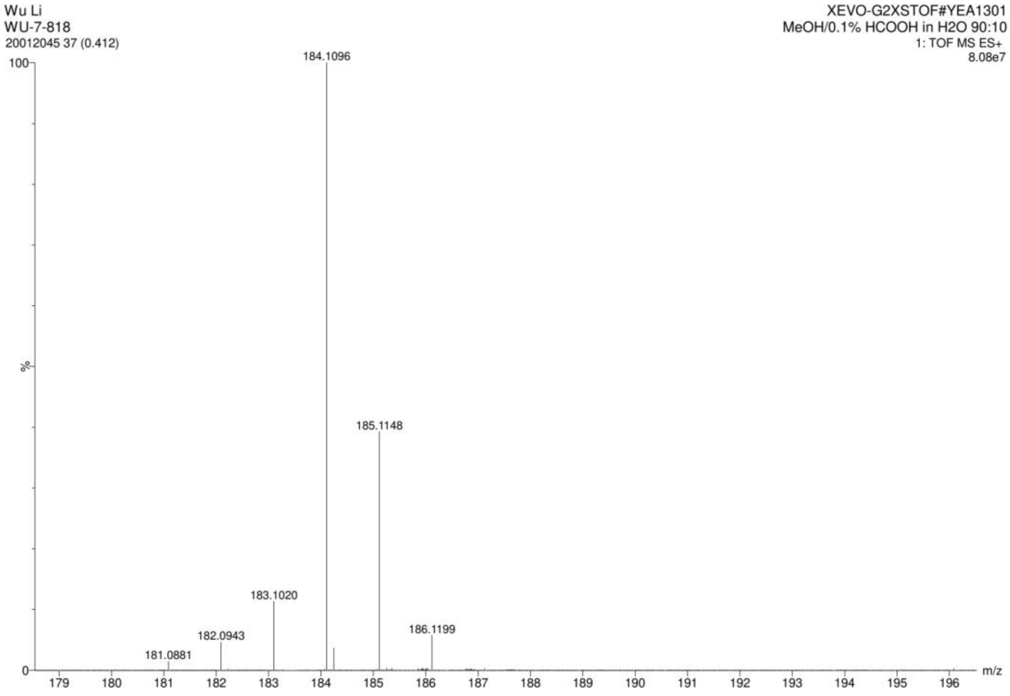

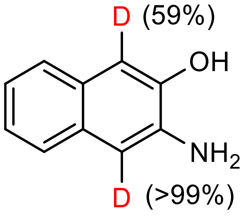

26b

According to GP, Fe-Cellulose-1000 (60 mg, 0.05 mol), substrate (37 mg, 0.23 mmol), D_2O (1.5 mL), H_2 (20 bar), room temperature to 120 °C and then at 120 °C for 24 h. The product **26b** (36 mg, 0.23 mmol, >99%) was obtained.

^{1}H NMR (300 MHz, DMSO-d_6) δ 9.82 (s, 1H), 7.46 (dd, J = 11.6, 7.8 Hz, 2H), 7.08 (dt, J = 19.6, 7.2 Hz, 2H), 6.88 (s, 0.41H), 5.01 (s, 1H).

^{13}C NMR (75 MHz, DMSO-d_6) δ 146.36, 138.79, 130.02, 129.94, 127.85, 127.77, 125.89, 125.84, 125.02, 124.98, 123.17, 121.68, 108.43, 107.21.

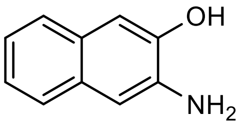

Chemical Formula: $C_{10}H_9NO$
Exact Mass: 159.0684

HRMS (ESI-TOF) of 26a:

ESI-TOF Accurate Mass Report Page 2
File:20073138 Sample Name:WU-24 UserName:Wu Li
Vial:1:D,6 Date:31-Jul-2020 Time:13:52:32
Description:MeOH/0.1% HCOOH in H2O 99:10

Sample Report:

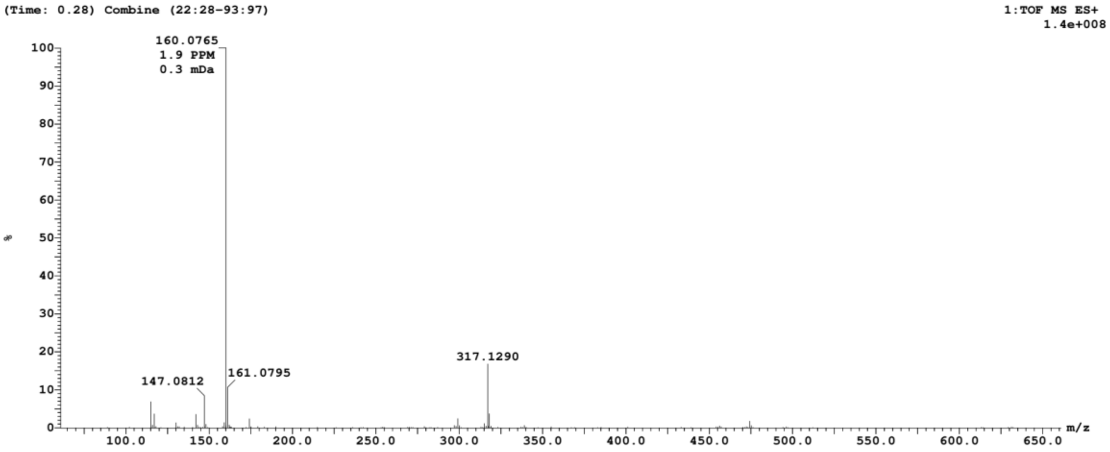

HRMS (ESI-TOF) of 26b $[M+H]^+$

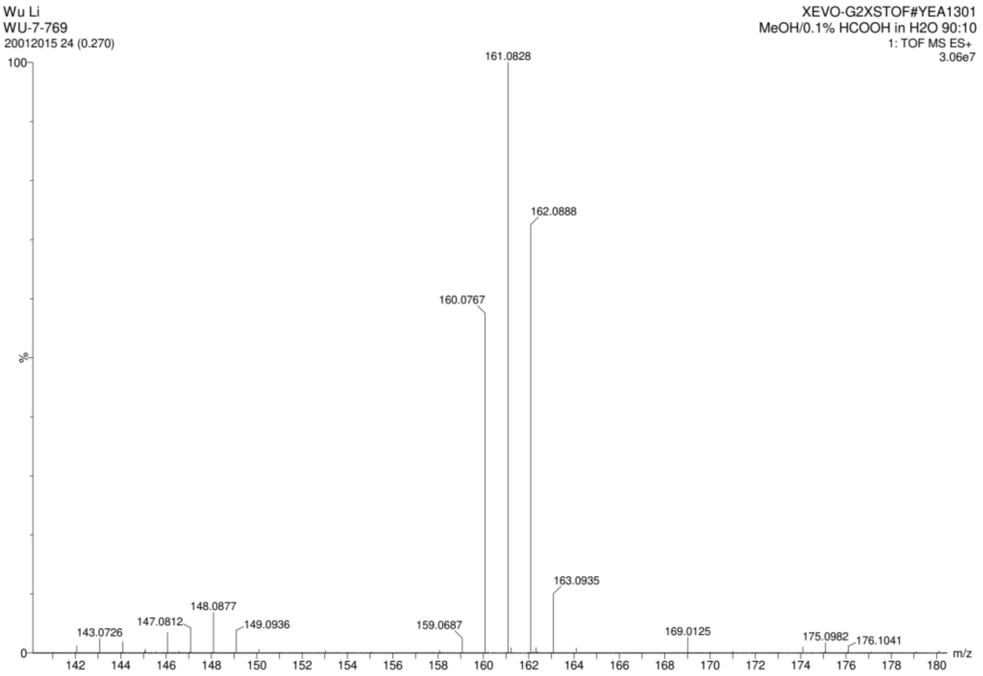

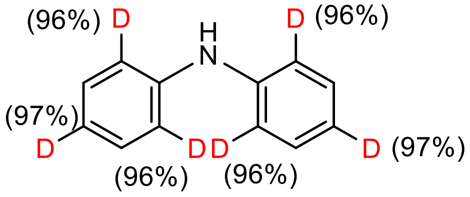

27b

According to GP, Fe-Cellulose-1000 (60 mg, 0.05 mol), substrate (44 mg, 0.26 mmol), D_2O (1.5 mL), H_2 (20 bar), room temperature to 120 °C and then at 120 °C for 24 h. The product **27b** (43 mg, 0.25 mmol, 96%) was obtained.

^{1}H NMR (300 MHz, DMSO-d_6) δ 8.14 (s, 1H), 7.22 (s, 4H), 7.08 (m, 0.18H), 6.80 (m, 0.06H).

^{13}C NMR (75 MHz, DMSO-d_6) δ 143.29, 129.03, 128.92, 119.58, 119.31, 119.01, 116.66, 116.35, 116.04.

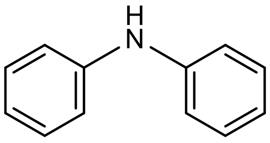

Chemical Formula: $C_{12}H_{11}N$
Exact Mass: 169.0891

HRMS (ESI-TOF) of 27a:

ESI-TOF Accurate Mass Report
File:20073139
Vial:1:D,7
Description:MeOH/0.1% HCOOH in H2O 99:10

Sample Name:WU-25
Date:31-Jul-2020

UserName:Wu Li
Time:13:55:07

Page 2

Sample Report:

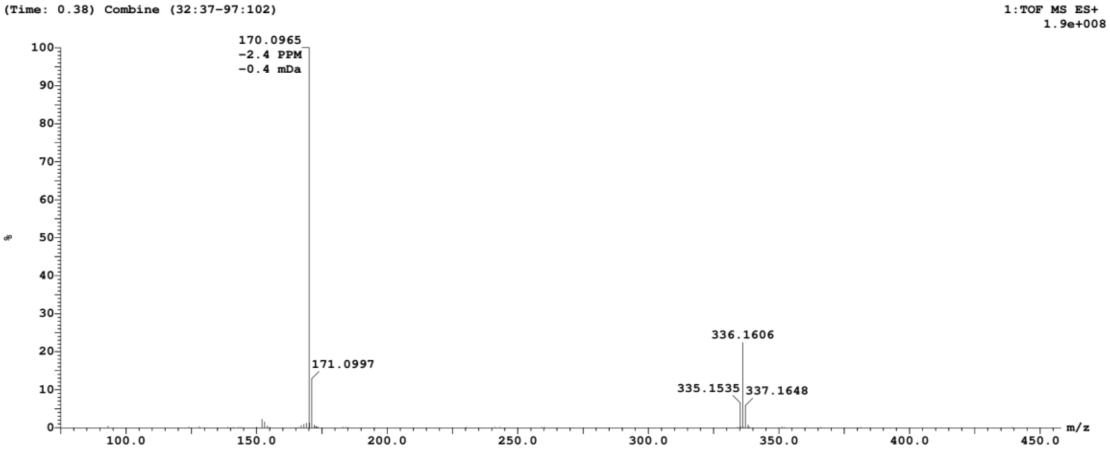

HRMS (ESI-TOF) of 27b $[M+H]^+$

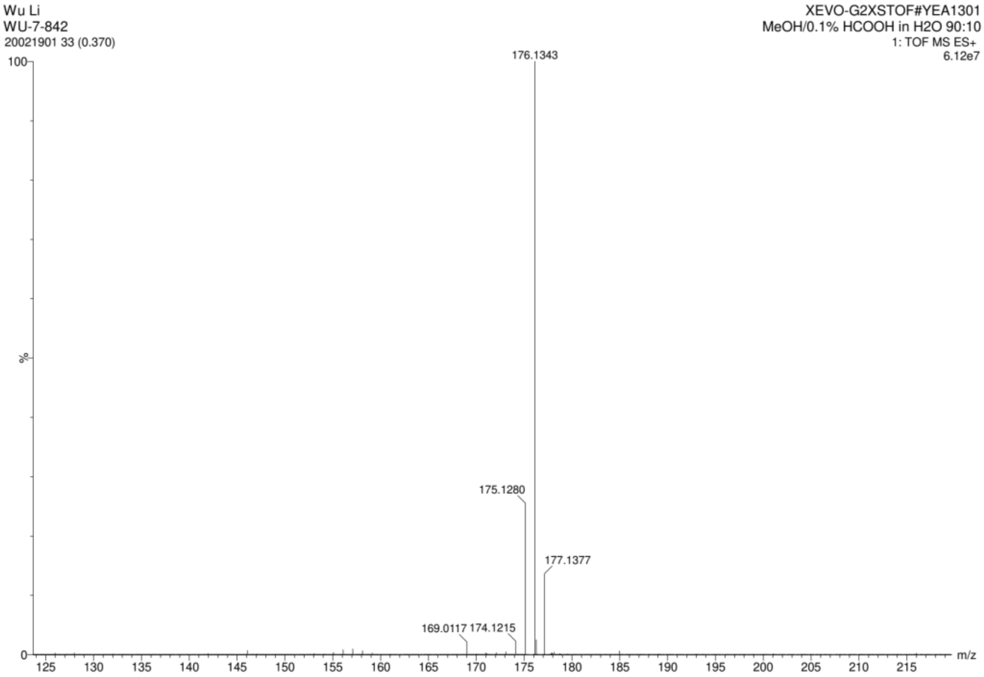

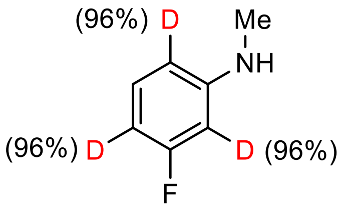

28b

According to GP, Fe-Cellulose-1000 (61 mg, 0.05 mol), substrate (38 mg, 0.30 mmol), D_2O (1.5 mL), H_2 (20 bar), room temperature to 120 °C and then at 120 °C for 24 h. The product **28b** (32 mg, 0.25 mmol, 83%) was obtained.

^{1}H NMR (400 MHz, DMSO-d_6) δ 7.06 (d, J = 7.1 Hz, 1H), 6.37 (m, 0.04 H), 6.26 (m, 0.09H), 6.08 – 5.83 (m, 1H), 2.66 (d, J = 5.0 Hz, 3H).

^{13}C NMR (101 MHz, DMSO-d_6) δ 165.19, 162.81, 152.35, 152.24, 130.39, 130.29, 108.44, 108.18, 107.95, 101.88, 101.66, 101.43, 98.16, 97.91, 97.68, 97.43, 30.02.

^{19}F NMR (282 MHz, $CDCl_3$) δ -113.68.

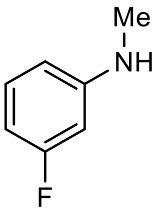

Chemical Formula: C_7H_8FN
Exact Mass: 125.0641

HRMS (ESI-TOF) of 28a:

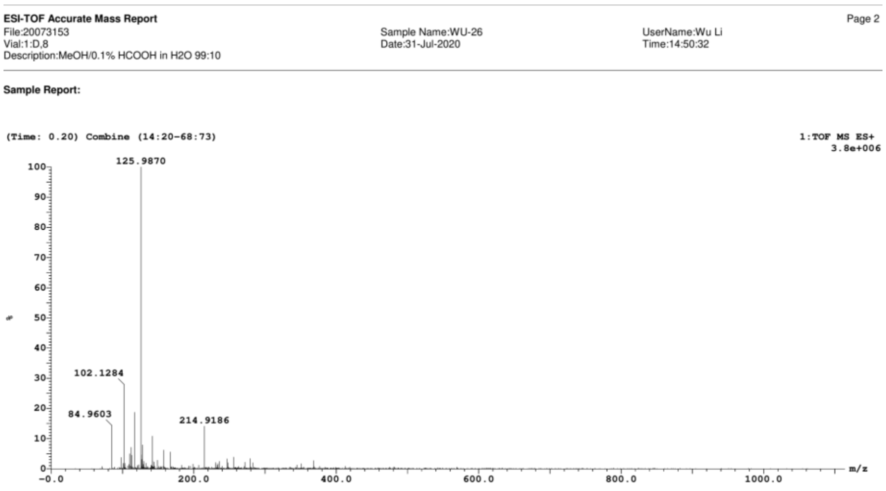

HRMS (ESI-TOF) of 28b [M+H]$^+$:

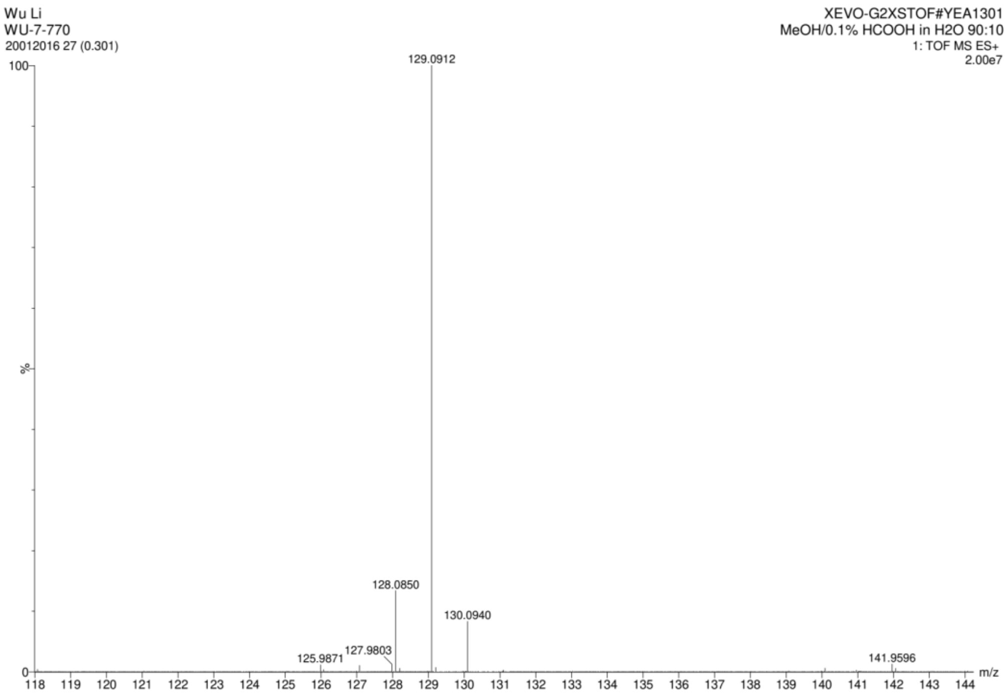

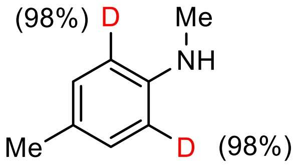

29b

According to GP, Fe-Cellulose-1000 (60 mg, 0.05 mol), substrate (37 mg, 0.31 mmol), D_2O (1.5 mL), H_2 (20 bar), room temperature to 120 °C and then at 120 °C for 24 h. The product **29b** (32 mg, 0.26 mmol, 84%) was obtained.

^{1}H NMR (300 MHz, $CDCl_3$) δ 7.03 (s, 2H), 6.58 (m, 0.05H), 3.35 (d, *J* = 20.9 Hz, 1H), 2.83 (s, 3H), 2.27 (s, 3H).

^{13}C NMR (75 MHz, $CDCl_3$) δ 147.13, 129.70, 126.60, 112.79, 112.48, 31.24, 20.51.

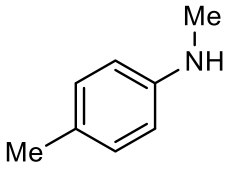

Chemical Formula: $C_8H_{11}N$
Exact Mass: 121.0891

HRMS (ESI-TOF) of 29a:

ESI-TOF Accurate Mass Report

File:20073141 | Sample Name:WU-27 | UserName:Wu Li | Page 2

Vial:1:E,1 | Date:31-Jul-2020 | Time:14:00:18

Description:MeOH/0.1% HCOOH in H2O 99:10

Sample Report:

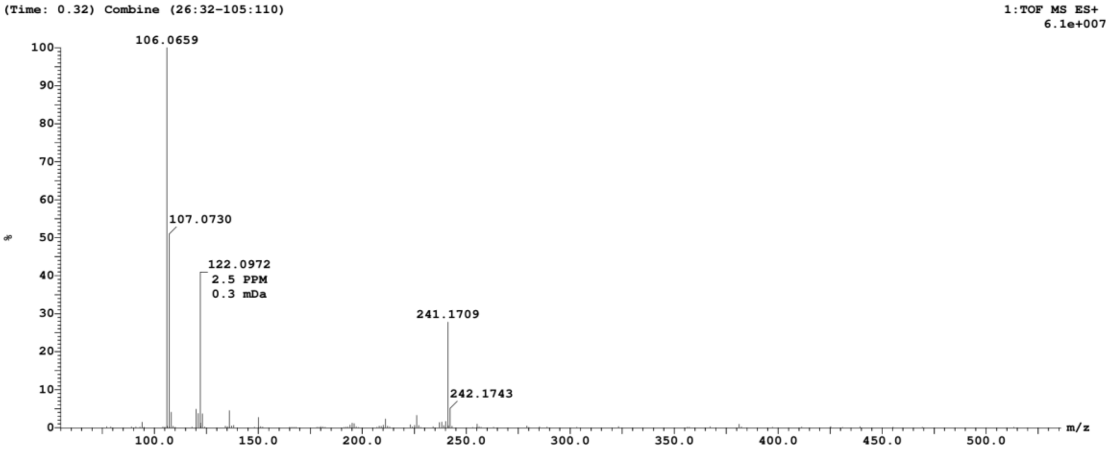

HRMS (ESI-TOF) of 29b $[M+H]^+$:

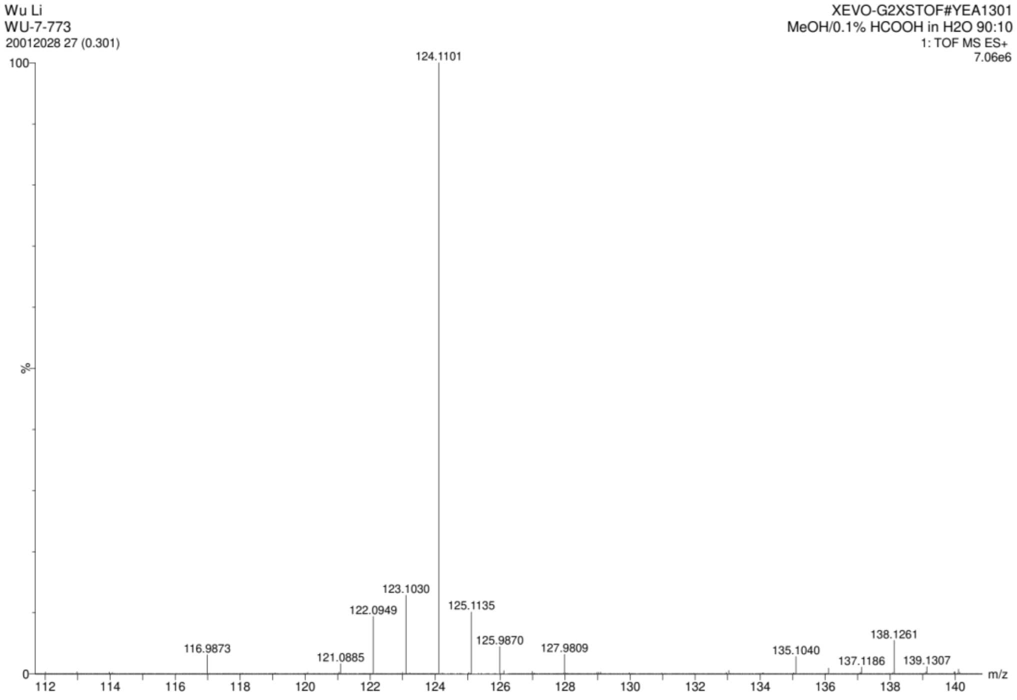

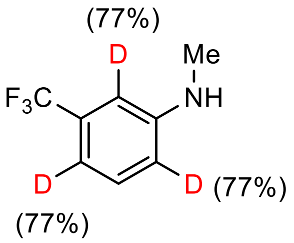

30b

According to GP, Fe-Cellulose-1000 (61 mg, 0.05 mol), substrate (48 mg, 0.27 mmol), D_2O (1.5 mL), H_2 (20 bar), room temperature to 120 °C and then at 120 °C for 24 h. The product **30b** (46 mg, 0.26 mmol, 96%) was obtained.

^1H NMR (400 MHz, DMSO-d_6) δ 7.21 (t, J = 4.2 Hz, 1H), 6.74 (d, J = 8.1 Hz, 0.68H), 5.89 (s, 1H), 2.65 (d, J = 3.5 Hz, 3H).

^{13}C NMR (101 MHz, DMSO-d_6) δ 150.49, 150.44, 130.69, 130.38, 130.08, 129.97, 129.87, 129.77, 129.00, 126.29, 123.59, 120.88, 115.54, 115.25, 115.01, 111.82, 111.58, 111.34, 107.52, 107.26, 107.02, 29.71.

^{19}F NMR (282 MHz, DMSO-d_6) δ -61.60.

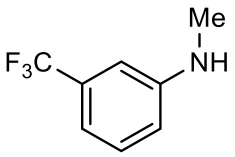

Chemical Formula: $C_8H_8F_3N$
Exact Mass: 175.0609

HRMS (ESI-TOF) of 30a:

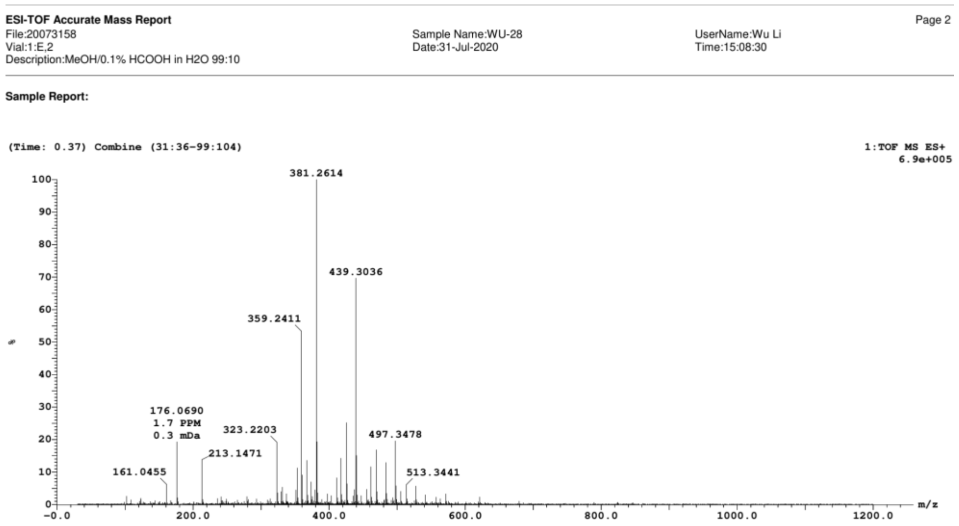

HRMS (EI) of 30b $[M]^+$

File : D:\Xcalibur\data\2001\20012308eihr-av2.RAW
Full ms [164.500 - 196.500] - Range: 178.000 - 178.100
Scan No. 1 of 1

Mass	Absolute Intensity	Relative Intensity	Theoretical Mass	Delta [ppm]	Delta [mmu]	RDB	Composition
178.07744	132783	4.5	178.07762	-1.0	-0.2	4.5	$C_8 H_3 {}^2H_4 N_1 F_3$

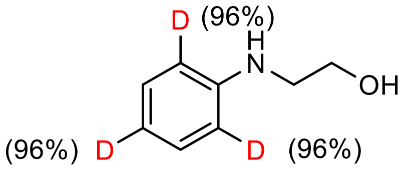

31b (>99%)

According to GP, Fe-Cellulose-1000 (60 mg, 0.05 mol), substrate (37 mg, 0.27 mmol), D_2O (1.5 mL), H_2 (20 bar), room temperature to 120 °C and then at 120 °C for 24 h. The product **31b** (38 mg, 0.27 mmol, >99%) was obtained.

^{1}H NMR (300 MHz, DMSO-d_6) δ 7.07 (s, 2H), 6.56 (m, 0.11H), 5.43 (s, 1H), 4.69 (m, 1H), 3.57 (q, J = 5.9 Hz, 2H), 3.09 (dd, J = 8.1, 3.7 Hz, 2H).

^{13}C NMR (75 MHz, $CDCl_3$) δ 148.08, 129.31, 129.20, 118.16, 117.84, 113.41, 112.78, 61.33, 46.24.

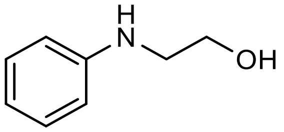

Chemical Formula: $C_8H_{11}NO$
Exact Mass: 137.0841

HRMS (ESI-TOF) of 31a:

ESI-TOF Accurate Mass Report
File:20073143
Vial:1:E,3
Description:MeOH/0.1% HCOOH in H2O 99:10

Sample Name:WU-29
Date:31-Jul-2020

UserName:Wu Li
Time:14:05:28

Page 2

Sample Report:

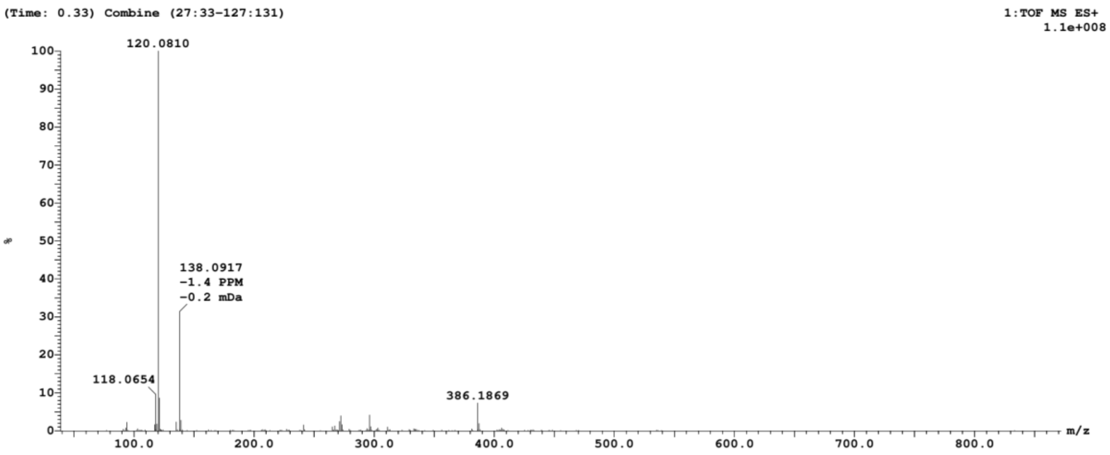

HRMS (ESI-TOF) of 31b $[M+H]^+$

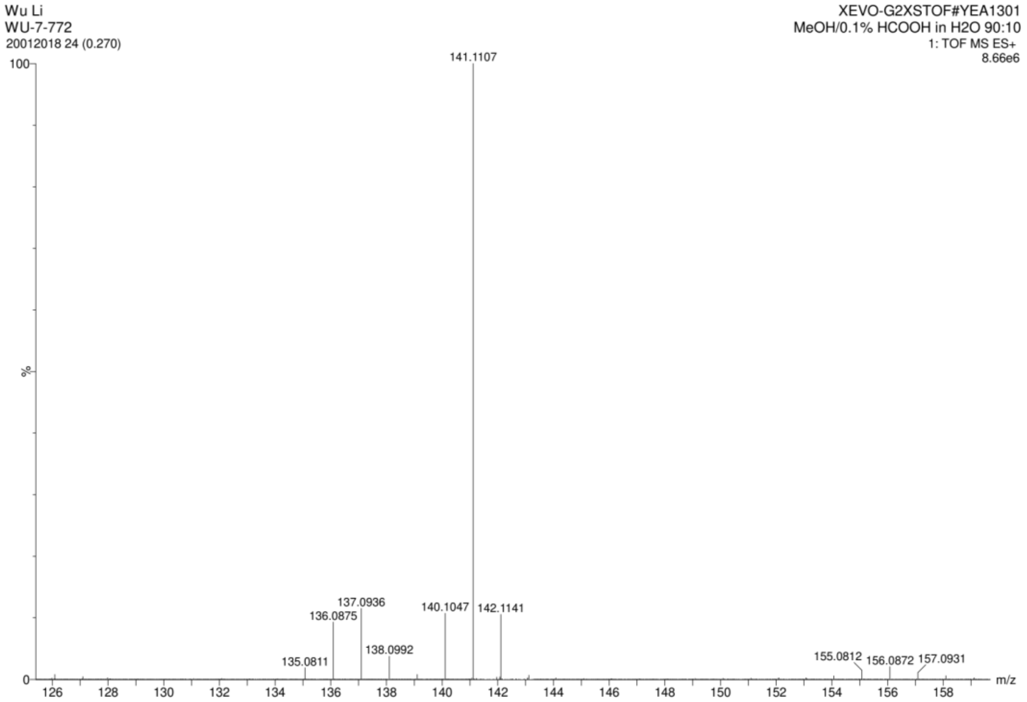

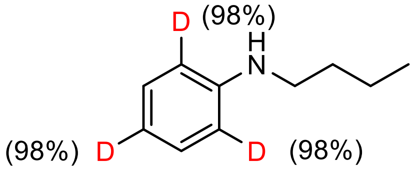

32b

According to GP, Fe-Cellulose-1000 (61 mg, 0.05 mol), substrate (39 mg, 0.26 mmol), D_2O (1.5 mL), H_2 (20 bar), room temperature to 120 °C and then at 120 °C for 24 h. The product **32b** (38 mg, 0.25 mmol, 96%) was obtained.

^1H NMR (300 MHz, DMSO-d_6) δ 7.05 (s, 2H), 6.58 – 6.46 (m, 0.05H), 5.47 (m, 1H), 3.02 – 2.95 (m, 2H), 1.57 – 1.49 (m, 2H), 1.39 (ddd, J = 10.0, 7.7, 5.9 Hz, 2H), 0.92 (t, J = 7.3 Hz, 3H).

^{13}C NMR (101 MHz, DMSO-d_6) δ 149.46, 149.41, 129.14, 129.03, 115.68, 115.43, 115.20, 112.31, 112.26, 112.02, 111.79, 43.01, 42.92, 31.38, 31.35, 20.37, 14.28.

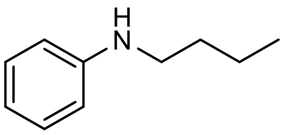

Chemical Formula: $C_{10}H_{15}N$
Exact Mass: 149.1204

HRMS (ESI-TOF) of 32a:

ESI-TOF Accurate Mass Report Page 2
File:20073144 Sample Name:WU-30 UserName:Wu Li
Vial:1:E,4 Date:31-Jul-2020 Time:14:08:02
Description:MeOH/0.1% HCOOH in H2O 99:10

Sample Report:

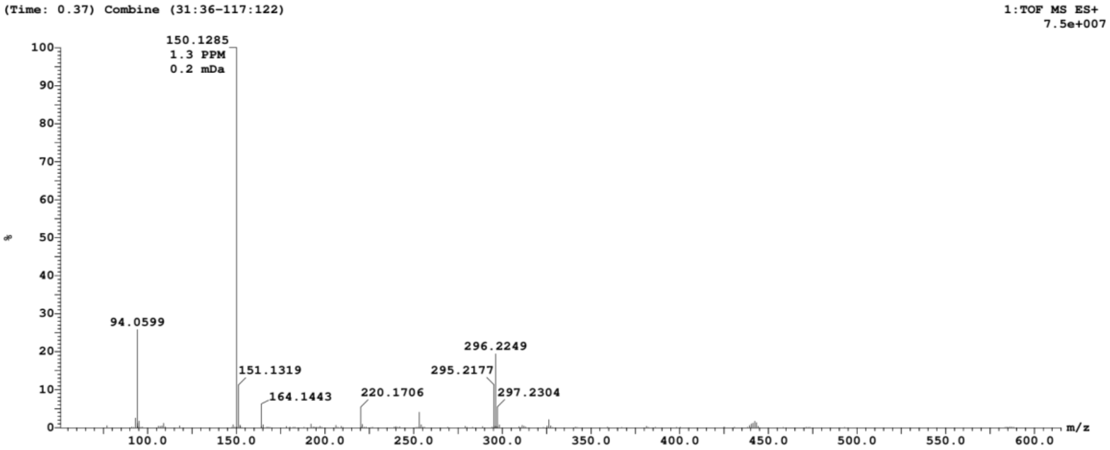

HRMS (ESI-TOF) of 32b $[M+H]^+$:

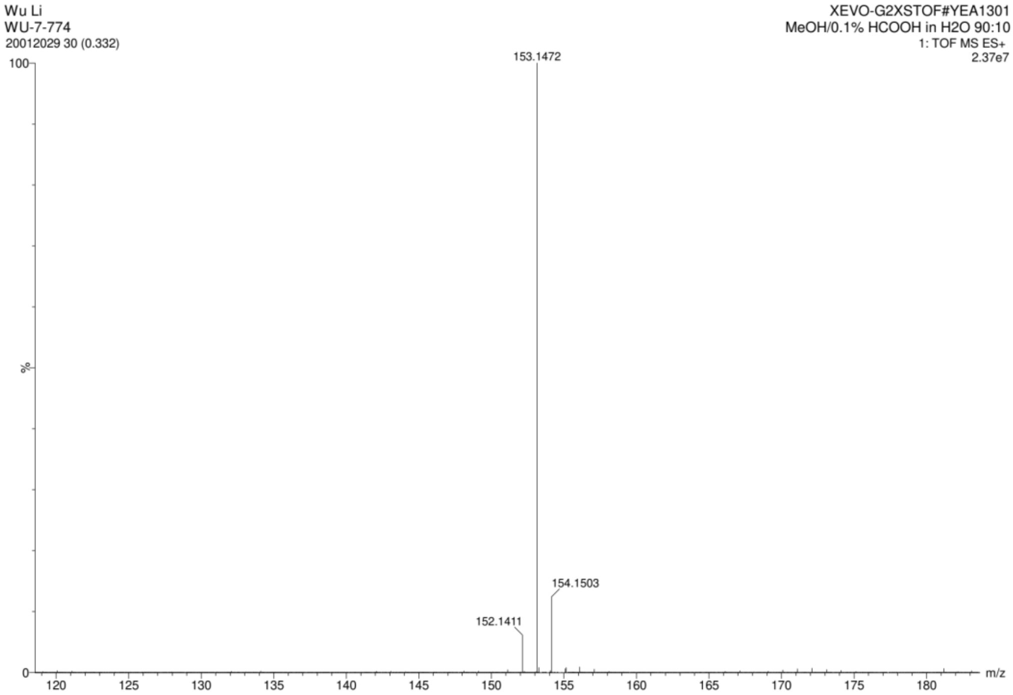

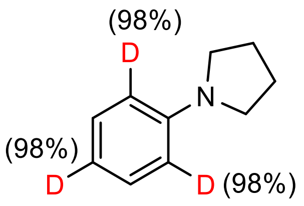

33b

According to GP, Fe-Cellulose-1000 (60 mg, 0.05 mol), substrate (49 mg, 0.33 mmol), D_2O (1.5 mL), H_2 (20 bar), room temperature to 120 °C and then at 120 °C for 24 h. The product **33b** (41 mg, 0.27 mmol, 82%) was obtained.

^{1}H NMR (400 MHz, DMSO-d_6) δ 7.14 (s, 2H), 6.52 (m, 0.07H), 3.18 (d, J = 5.8 Hz, 4H), 1.93 (q, J = 6.2 Hz, 4H).

^{13}C NMR (101 MHz, DMSO-d_6) δ 147.61, 128.77, 128.66, 114.96, 114.71, 114.48, 111.52, 111.28, 111.04, 47.19, 24.91.

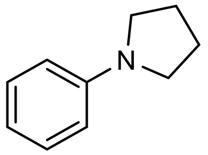

Chemical Formula: $C_{10}H_{13}N$
Exact Mass: 147.1048

HRMS (ESI-TOF) of 33a:

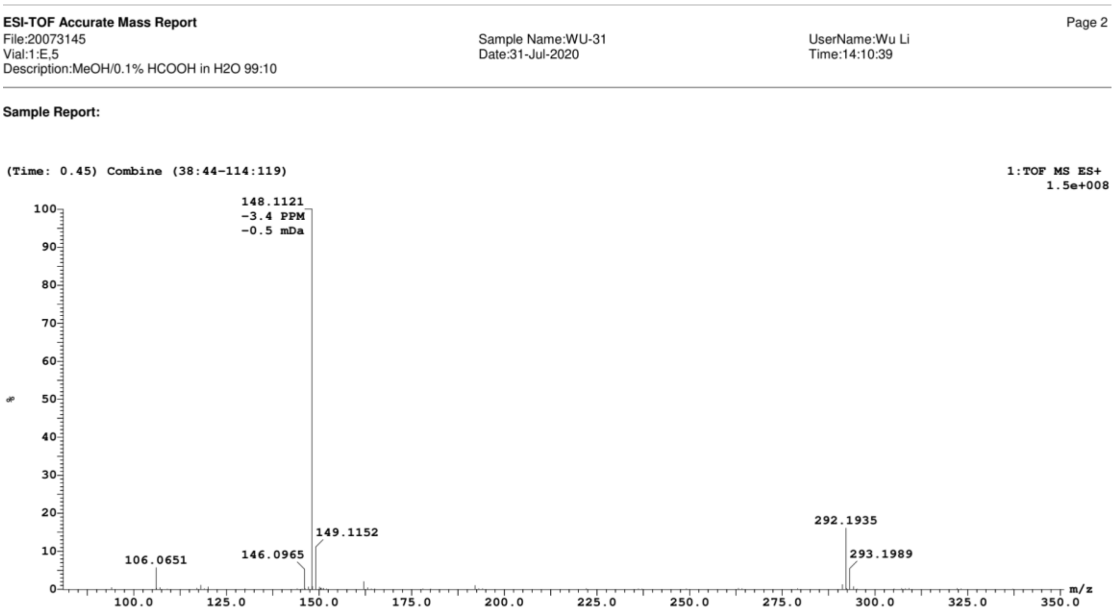

HRMS (ESI-TOF) of 33b [M+H]⁺:

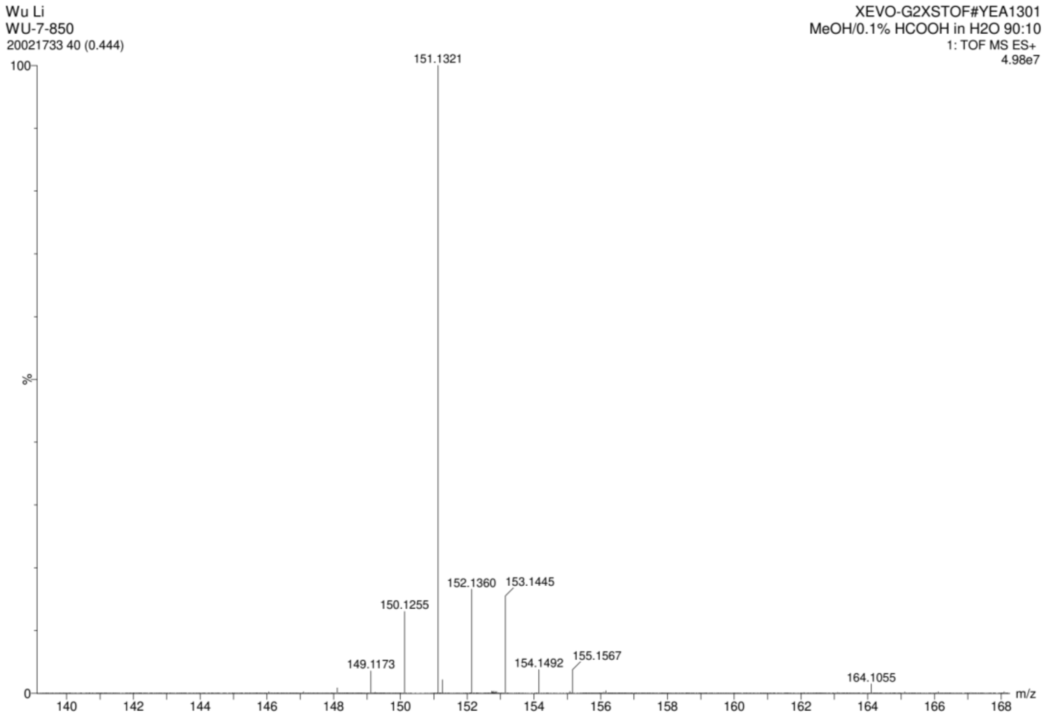

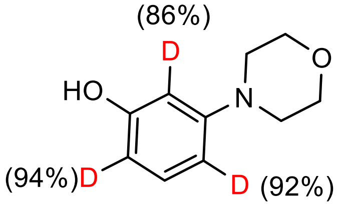

34b

According to GP, Fe-Cellulose-1000 (60 mg, 0.05 mol), substrate (48 mg, 0.27 mmol), D_2O (1.5 mL), H_2 (20 bar), room temperature to 120 °C and then at 120 °C for 24 h. The product **34b** (47 mg, 0.26 mmol, 96%) was obtained.

^{1}H NMR (300 MHz, $CDCl_3$) δ 7.15 (d, J = 4.2 Hz, 1H), 6.53 (m, 0.06H), 6.39 (m, 0.14H), 6.36 (s, 0.08H), 5.90 (s, 1H), 3.99 – 3.78 (m, 4H), 3.26 – 3.06 (m, 4H).

^{13}C NMR (75 MHz, $CDCl_3$) δ 156.73, 152.70, 130.12, 130.02, 108.36, 107.93, 107.25, 102.99, 102.83, 102.73, 66.91, 49.33.

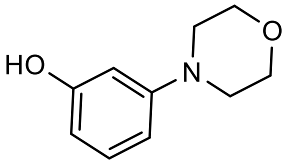

Chemical Formula: $C_{10}H_{13}NO_2$
Exact Mass: 179.0946

HRMS (ESI-TOF) of 34a:

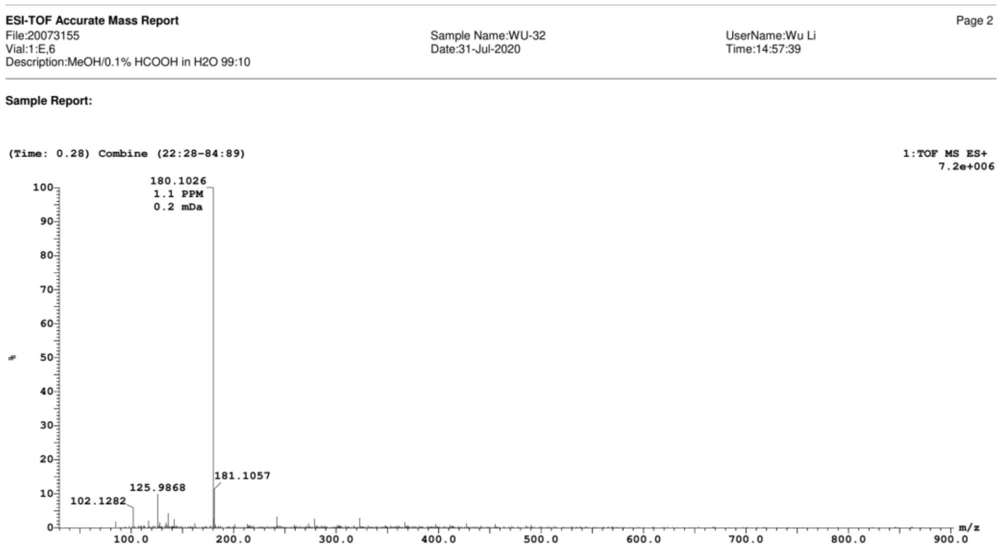

HRMS (ESI-TOF) of 34b [M+H]+:

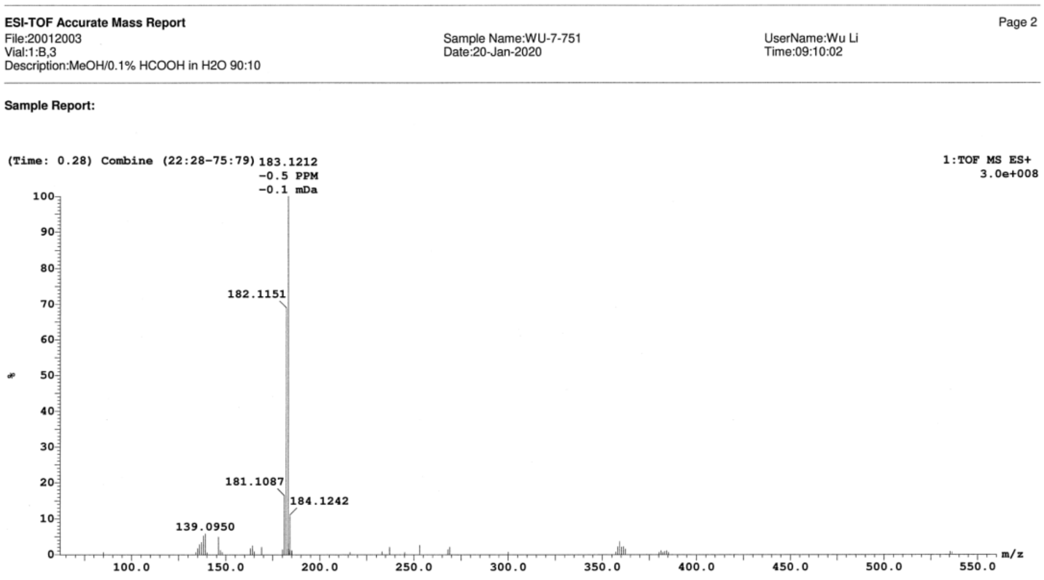

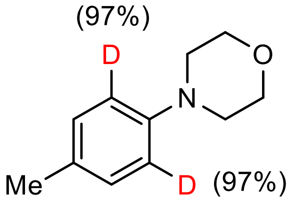

According to GP, Fe-Cellulose-1000 (61 mg, 0.05 mol), substrate (43 mg, 0.24 mmol), D_2O (1.5 mL), H_2 (20 bar), room temperature to 120 °C and then at 120 °C for 24 h. The product **35b** (43 mg, 0.24 mmol, >99%) was obtained.

^{1}H NMR (300 MHz, $CDCl_3$) δ 7.10 (s, 2H), 6.86 (m, 0.06H), 3.98 – 3.76 (m, 4H), 3.23 – 3.01 (m, 4H), 2.29 (d, J = 0.7 Hz, 3H).

^{13}C NMR (75 MHz, $CDCl_3$) δ 149.17, 129.72, 116.16, 115.84, 115.52, 67.09, 50.03, 20.55.

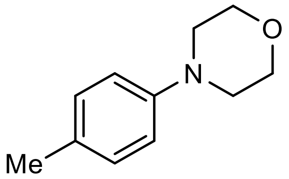

Chemical Formula: $C_{11}H_{15}NO$
Exact Mass: 177.1154

HRMS (ESI-TOF) of 35a:

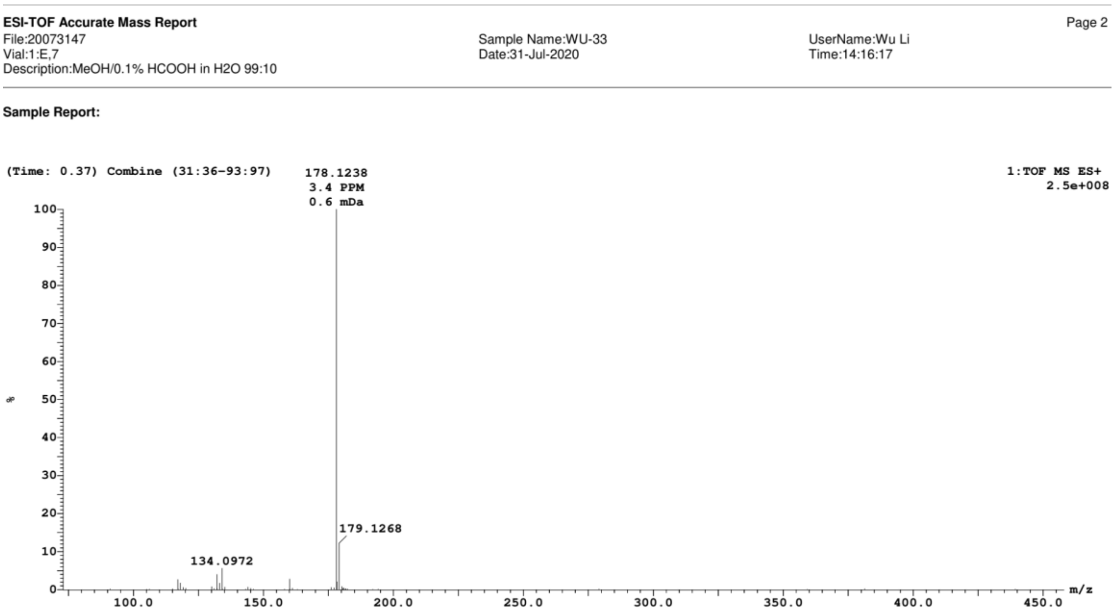

HRMS (ESI-TOF) of 35b $[M+H]^+$:

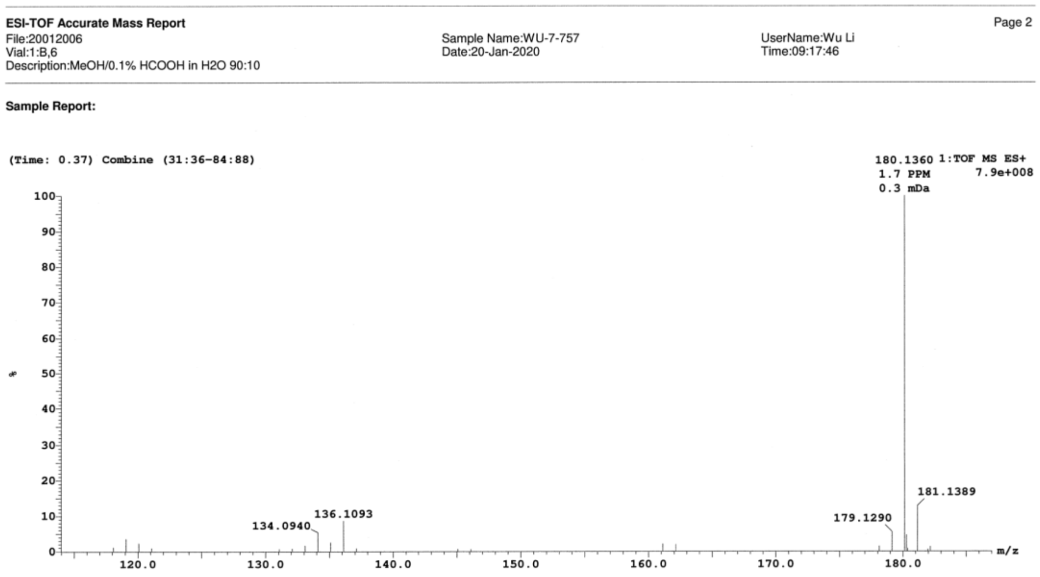

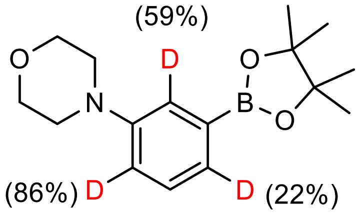

36b

According to GP, Fe-Cellulose-1000 (61 mg, 0.05 mol), substrate (78 mg, 0.27 mmol), D_2O (1.5 mL), H_2 (20 bar), room temperature to 120 °C and then at 120 °C for 24 h. The product **36b** (75 mg, 0.26 mmol, 96%) was obtained.

^{1}H NMR (300 MHz, DMSO-d_6) δ 7.30 – 7.22 (m, 1H), 7.19 (d, J = 2.4 Hz, 0.41H), 7.16 (s, 0.14H), 7.09 (dt, J = 8.4, 1.4 Hz, 0.78H), 3.77 – 3.70 (m, 4H), 3.13 – 3.03 (m, 4H), 1.29 (s, 12H).

^{13}C NMR (75 MHz, $CDCl_3$) δ 150.79, 150.73, 128.61, 128.50, 126.67, 121.92, 118.91, 83.85, 67.09, 49.59, 24.97.

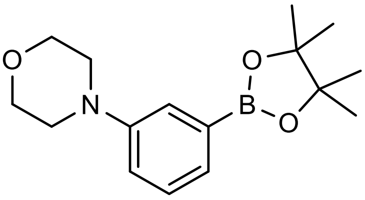

Chemical Formula: $C_{16}H_{24}BNO_3$
Exact Mass: 289.1849

HRMS (ESI-TOF) of 36a:

ESI-TOF Accurate Mass Report Page 2
File:20073148
Vial:1:E,8
Description:MeOH/0.1% HCOOH in H2O 99:10
Sample Name:WU-34
Date:31-Jul-2020
UserName:Wu Li
Time:14:18:53

Sample Report:

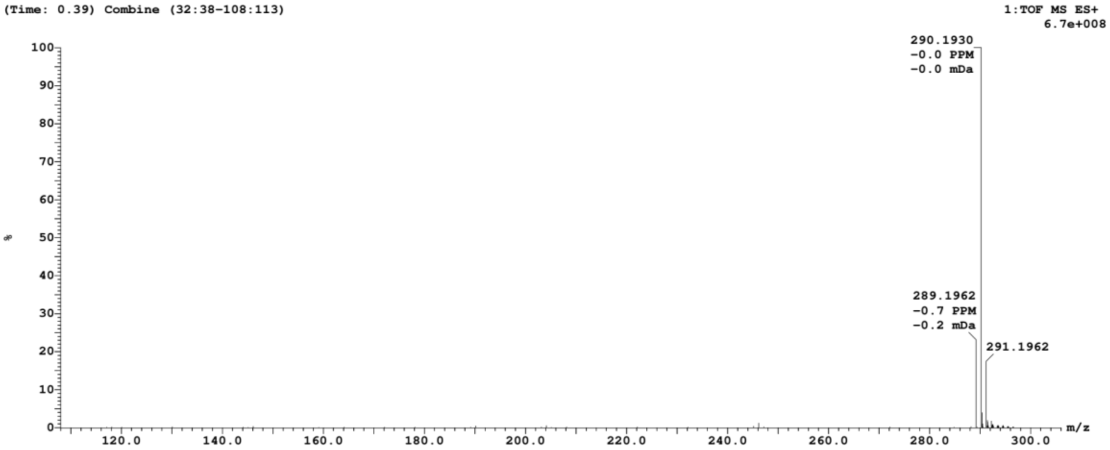

HRMS (ESI-TOF) of 36b $[M+H]^+$:

ESI-TOF Accurate Mass Report Page 2
File:20012022
Vial:1:B,8
Description:MeOH/0.1% HCOOH in H2O 90:10
Sample Name:WU-7-760
Date:20-Jan-2020
UserName:Wu Li
Time:11:40:46

Sample Report:

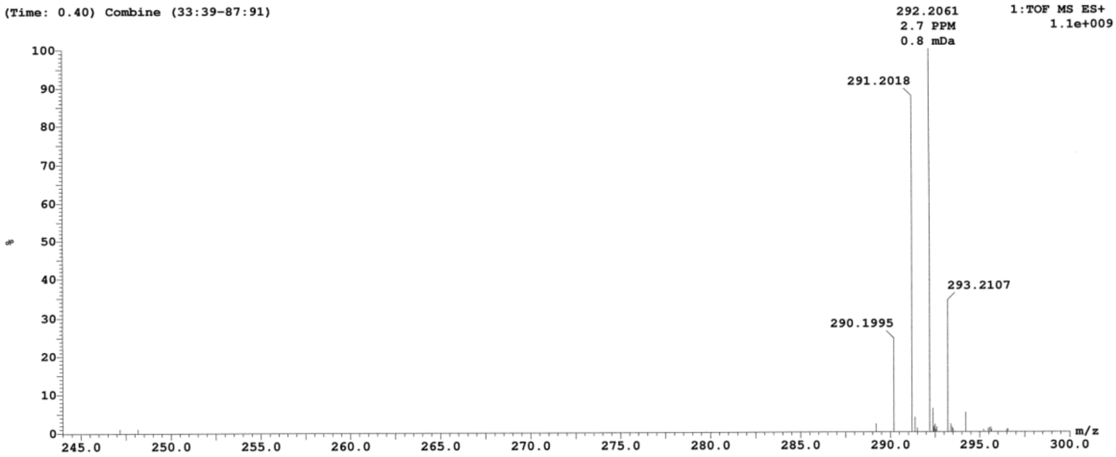

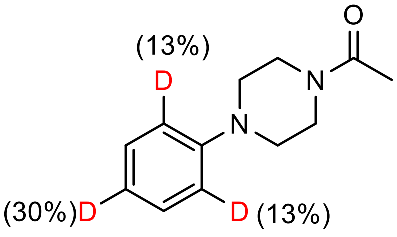

37b

According to GP, Fe-Cellulose-1000 (61 mg, 0.05 mol), substrate (51 mg, 0.25 mmol), D_2O (1.5 mL), H_2 (20 bar), room temperature to 120 °C and then at 120 °C for 24 h. The product **37b** (50 mg, 0.24 mmol, 96%) was obtained.

1H NMR (300 MHz, DMSO-d_6) δ 7.29 – 7.18 (m, 2H), 6.98 – 6.93 (m, 1.74H), 6.81 (tt, J = 7.3, 1.1 Hz, 0.70), 3.57 (q, J = 4.7 Hz, 4H), 3.14 (t, J = 5.2 Hz, 2H), 3.08 (t, J = 5.3 Hz, 2H), 2.04 (s, 3H).

^{13}C NMR (75 MHz, $CDCl_3$) δ 169.04, 150.99, 129.31, 129.20, 120.63, 116.73, 77.36, 49.79, 49.45, 46.31, 41.42, 21.44.

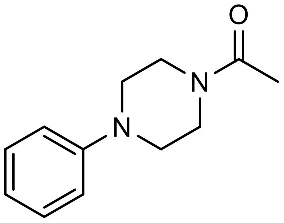

Chemical Formula: $C_{12}H_{16}N_2O$
Exact Mass: 204.1263

HRMS (ESI-TOF) of 37a:

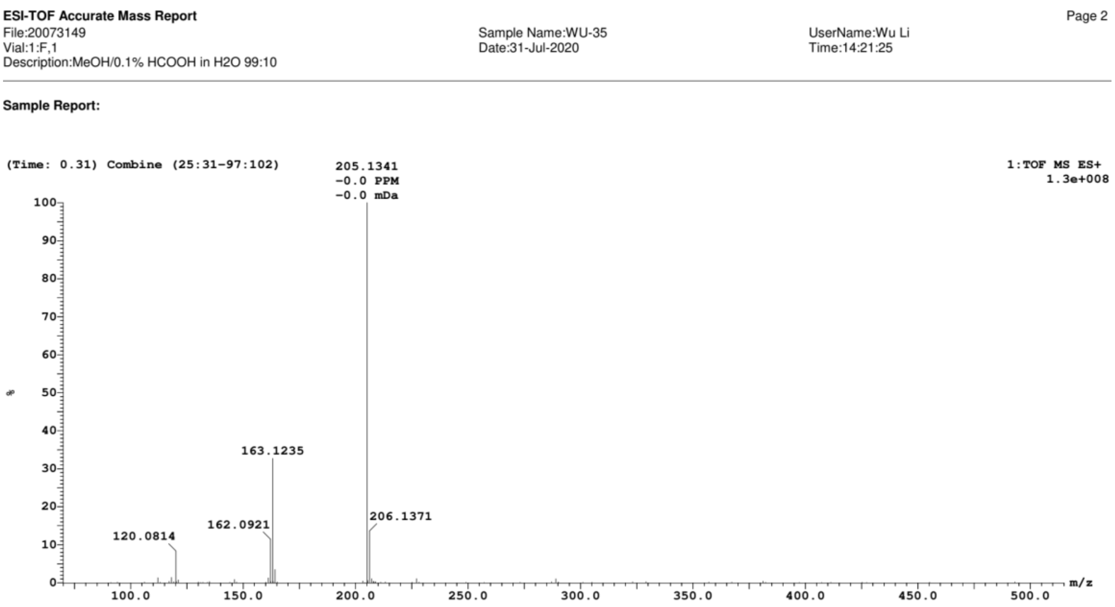

HRMS (ESI-TOF) of 37b [M+H]$^+$:

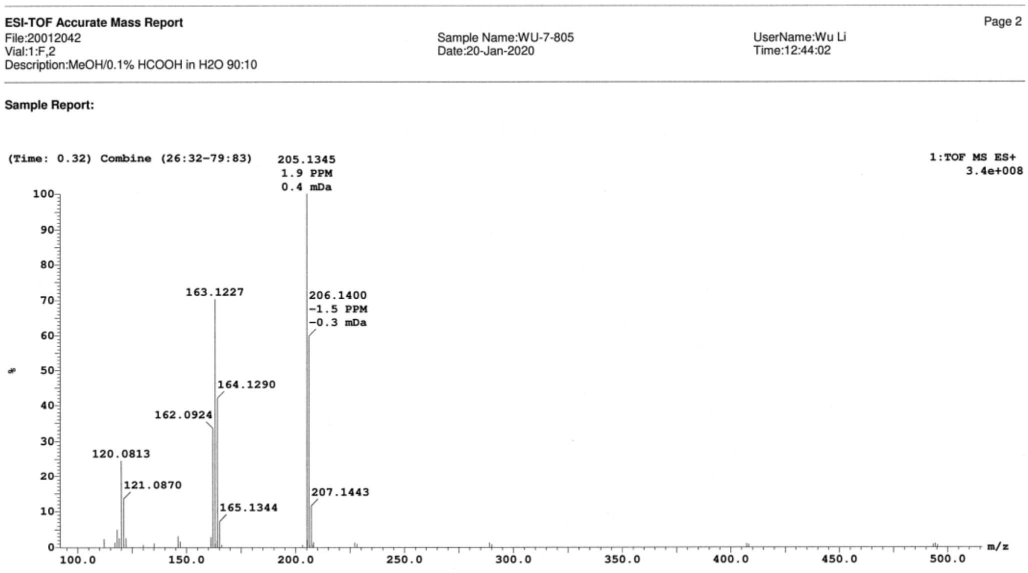

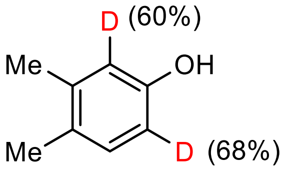

38b

According to GP, Fe-Cellulose-1000 (63 mg, 0.05 mol), substrate (36 mg, 0.30 mmol), D_2O (1.5 mL), H_2 (20 bar), room temperature to 120 °C and then at 120 °C for 72 h. The product **38b** (36 mg, 0.29 mmol, 97%) was obtained.

^{1}H NMR (300 MHz, DMSO-d_6) δ 8.98 (s, 1H), 7.01 – 6.84 (m, 1H), 6.56 (m, 0.40H), 6.46 (m, 0.32H), 2.10 (m, 6H).

^{13}C NMR (75 MHz, DMSO-d_6) δ 155.67, 155.63, 155.58, 137.35, 137.27, 130.56, 130.47, 126.35, 116.92, 112.80, 20.02, 19.96, 18.84.

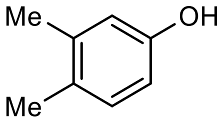

Chemical Formula: $C_8H_{10}O$
Exact Mass: 122.0732

HRMS (ESI-TOF) of 38a:

ESI-TOF Accurate Mass Report Page 2

File:20073159 Sample Name:WU-36 UserName:Wu Li

Vial:1:F,2 Date:31-Jul-2020 Time:15:11:07

Description:MeOH/0.1% HCOOH in H2O 99:10

Sample Report:

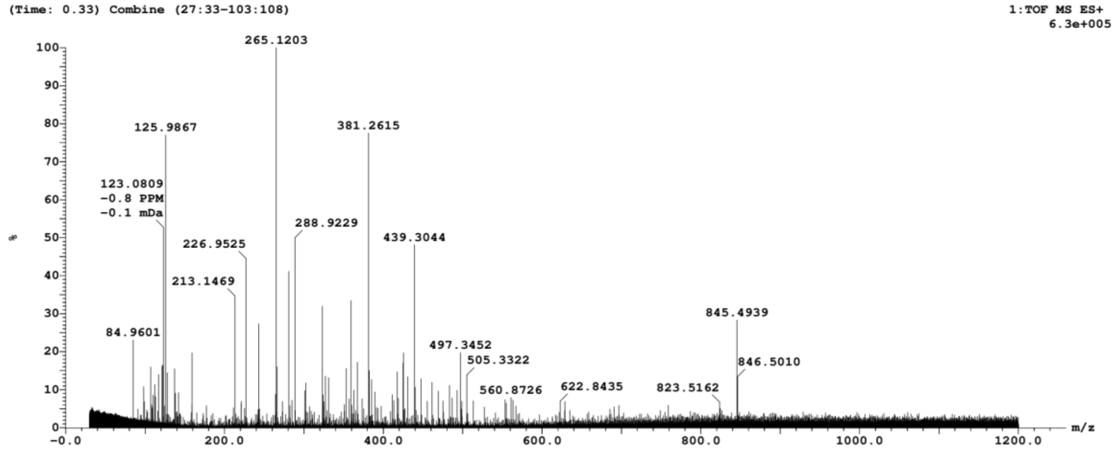

HRMS (EI) of 38b [M]$^+$:

Wu Li 7-897 HR (EI)

File : D:\Xcalibur\data\2005\20050602ahrei-av2.RAW
Full ms [115.500 - 135.500] - Range: 115.500 - 135.500
Scan No. 1 of 1

Mass	Absolute Intensity	Relative Intensity	Theoretical Mass	Delta [ppm]	Delta [mmu]	RDB	Composition
118.99147	17262080	86.5					
123.07856	19947264	100.0	123.07889	-2.7	-0.3	4.0	$C_8 H_9 {}^2H_1 O_1$
124.08516	13523200	67.8	124.08517	-0.1	-0.0	4.0	$C_8 H_8 {}^2H_2 O_1$
130.99147	14906880	74.7					

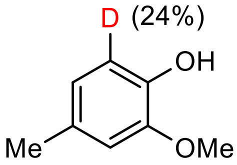

39b

According to GP, Fe-Cellulose-1000 (62 mg, 0.05 mol), substrate (40 mg, 0.29 mmol), D_2O (1.5 mL), H_2 (20 bar), room temperature to 120 °C and then at 120 °C for 72 h. The product **39b** (32 mg, 0.23 mmol, 80%) was obtained.

^{1}H NMR (300 MHz, DMSO-d_6) δ 8.66 (s, 1H), 6.77 (dd, J = 2.0, 0.7 Hz, 1H), 6.70 (d, J = 7.9 Hz, 0.76H), 6.62 – 6.50 (m, 1H), 3.78 (s, 3H), 2.25 (s, 3H).

^{13}C NMR (75 MHz, DMSO-d_6) δ 147.81, 144.62, 128.34, 121.41, 121.31, 115.75, 113.60, 55.94, 21.03.

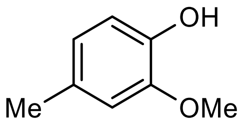

Chemical Formula: $C_8H_{10}O_2$
Exact Mass: 138.0681

HRMS (ESI-TOF) of 39a:

Wu - 37 HR (EI)

File : D:\Xcalibur\data\2009\20090106hrei-av2.RAW
Full ms [127.500 - 159.500] - Range: 138.000 - 138.500
Scan No. 1 of 1

Mass	Absolute Intensity	Relative Intensity	Theoretical Mass	Delta [ppm]	Delta [mmu]	RDB	Composition
138.06788	2640790	100.0	138.06753	2.5	0.4	4.0	$C_8 H_{10} O_2$

HRMS (EI) of 39b [M]$^+$

Wu - 8 - 82 HR (EI)

File : D:\Xcalibur\data\2004\20040705eihr-av2.RAW
Full ms [127.500 - 146.500] - Range: 138.000 - 146.500
Scan No. 1 of 1

Mass	Absolute Intensity	Relative Intensity	Theoretical Mass	Delta [ppm]	Delta [mmu]	RDB	Composition
138.06742	16352768	65.2	138.06753	-0.8	-0.1	4.0	$C_8 H_{10} O_2$
139.07325	6480777	25.8	139.07381	-4.0	-0.6	4.0	$C_8 H_9 {}^2H_1 O_2$

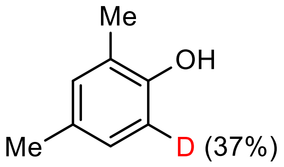

40b

According to GP, Fe-Cellulose-1000 (60 mg, 0.05 mol), substrate (35 mg, 0.29 mmol), D_2O (1.5 mL), H_2 (20 bar), room temperature to 120 °C and then at 120 °C for 72 h. The product **40b** (25 mg, 0.20 mmol, 71%) was obtained.

^{1}H NMR (400 MHz, DMSO-d_6) δ 8.93 (s, 1H), 6.84 (d, J = 2.3 Hz, 1H), 6.76 (dt, J = 5.7, 2.5 Hz, 1H), 6.64 (d, J = 8.0 Hz, 0.63H), 2.14 (s, 3H), 2.07 (s, 3H).

^{13}C NMR (101 MHz, DMSO-d_6) δ 153.48, 131.58, 127.44, 127.30, 123.87, 114.86, 20.56, 16.39.

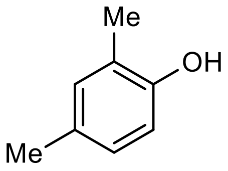

Chemical Formula: $C_8H_{10}O$
Exact Mass: 122.0732

HRMS (ESI-TOF) of 40a:

Wu Li Wu - 38 HR (EI)

File : D:\Xcalibur\data\2009\20090104hrei-av3.RAW
Full ms [115.500 - 135.500] - Range: 122.000 - 122.500
Scan No. 1 of 1

Mass	Absolute Intensity	Relative Intensity	Theoretical Mass	Delta [ppm]	Delta [mmu]	RDB	Composition
122.07285	1861764	100.0	122.07262	1.9	0.2	4.0	$C_8 H_{10} O_1$

HRMS (EI) of 40b [M]$^+$:

Wu Li 8-90 HR (EI)

File : D:\Xcalibur\data\2005\20051104hrei-av2.RAW
Full ms [115.500 - 135.500] - Range: 123.000 - 123.500
Scan No. 1 of 1

Mass	Absolute Intensity	Relative Intensity	Theoretical Mass	Delta [ppm]	Delta [mmu]	RDB	Composition
123.03775	830380	6.8					
123.07868	4553310	37.5	123.07889	-1.8	-0.2	4.0	$C_8 H_9 {}^2H_1 O_1$
123.11656	176515	1.5					

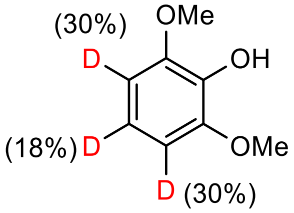

41b

According to GP, Fe-Cellulose-1000 (61 mg, 0.05 mol), substrate (42 mg, 0.27 mmol), D_2O (1.5 mL), H_2 (20 bar), room temperature to 120 °C and then at 120 °C for 72 h. The product **41b** (36 mg, 0.23 mmol, 85%) was obtained.

^{1}H NMR (400 MHz, DMSO-*d*$_6$) δ 8.26 (s, 1H), 6.72 – 6.68 (m, 0.82H), 6.60 (dd, *J* = 8.2, 0.9 Hz, 1.40H), 3.75 (s, 6H).

^{13}C NMR (101 MHz, DMSO-*d*$_6$) δ 148.66, 148.62, 136.12, 136.10, 118.60, 118.49, 106.16, 106.13, 56.39.

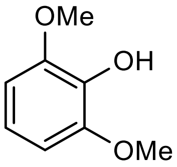

Chemical Formula: $C_8H_{10}O_3$
Exact Mass: 154.0630

HRMS (ESI-TOF) of 41a:

Wu – 39 HR (EI)

File : D:\Xcalibur\data\2009\20090107ahrei-av2.RAW
Full ms [139.500 - 173.500] - Range: 154.000 - 154.500
Scan No. 1 of 1

Mass	Absolute Intensity	Relative Intensity	Theoretical Mass	Delta [ppm]	Delta [mmu]	RDB	Composition
154.06267	405333	19.8	154.06245	1.4	0.2	4.0	$C_8\ H_{10}\ O_3$

HRMS (EI) of 41b [M]$^+$:

Wu-8-84 HR (EI)

File : D:\Xcalibur\data\2004\20040706eihr-av2.RAW
Full ms [138.500 - 172.500] - Range: 154.000 - 156.000
Scan No. 1 of 1

Mass	Absolute Intensity	Relative Intensity	Theoretical Mass	Delta [ppm]	Delta [mmu]	RDB	Composition
154.06221	81927168	100.0	154.06245	-1.5	-0.2	4.0	$C_8\ H_{10}\ O_3$
155.06823	71752192	87.6	155.06872	-3.2	-0.5	4.0	$C_8\ H_9\ {}^2H_1\ O_3$

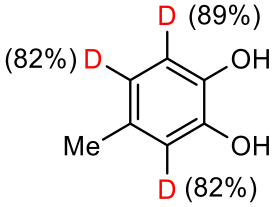

42b

According to GP, Fe-Cellulose-1000 (60 mg, 0.05 mol), substrate (33 mg, 0.27 mmol), D_2O (1.5 mL), H_2 (20 bar), room temperature to 120 °C and then at 120 °C for 72 h. The product **42b** (31 mg, 0.24 mmol, 89%) was obtained.

^{1}H NMR (400 MHz, DMSO-d_6) δ 9.22 – 8.22 (m, 2H), 6.58 (s, 0.37H), 6.38 (s, 0.11H), 2.10 (s, 3H).

^{13}C NMR (101 MHz, DMSO-d_6) δ 145.37, 143.29, 143.25, 128.15, 119.82, 116.56, 115.70, 20.69.

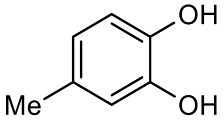

Chemical Formula: $C_7H_8O_2$
Exact Mass: 124.0524

HRMS (ESI-TOF) of 42a unlabelled:

Wu 40 HR (EI)

File : D:\Xcalibur\data\2009\20090105hrei-av2.RAW
Full ms [115.500 - 135.500] - Range: 124.000 - 124.500
Scan No. 1 of 1

Mass	Absolute Intensity	Relative Intensity	Theoretical Mass	Delta [ppm]	Delta [mmu]	RDB	Composition
124.05203	7679550	100.0	124.05188	1.2	0.2	4.0	$C_7 H_8 O_2$

HRMS (EI) of 42b [M]$^+$:

Wu Li 8-88 HR (EI)

File : D:\Xcalibur\data\2005\20051105hrei-av2.RAW
Full ms [115.500 - 135.500] - Range: 126.000 - 126.500
Scan No. 1 of 1

Mass	Absolute Intensity	Relative Intensity	Theoretical Mass	Delta [ppm]	Delta [mmu]	RDB	Composition
126.06406	54727424	94.0	126.06443	-3.0	-0.4	4.0	$C_7 H_6 {}^2H_2 O_2$

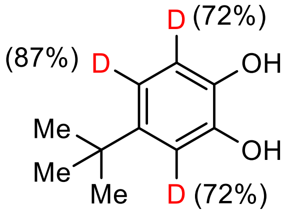

43b

According to GP, Fe-Cellulose-1000 (62 mg, 0.05 mol), substrate (55 mg, 0.33 mmol), D_2O (1.5 mL), H_2 (20 bar), room temperature to 120 °C and then at 120 °C for 72 h. The product **43b** (52 mg, 0.31 mmol, 94%) was obtained.

^{1}H NMR (400 MHz, DMSO-d_6) δ 8.58 (s, 2H), 6.74 (m, 0.13H), 6.62 (m, 0.56H), 1.18 (s, 9H).

^{13}C NMR (101 MHz, DMSO-d_6) δ 144.95, 143.22, 143.17, 142.09, 142.01, 116.03, 115.93, 115.50, 115.39, 113.36, 113.11, 34.01, 31.90.

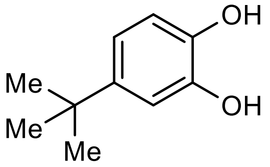

Chemical Formula: $C_{10}H_{14}O_2$
Exact Mass: 166.0994

HRMS (ESI-TOF) of 43a:

Wu – 41 HR (EI)

File : D:\Xcalibur\data\2009\20090108hrei-av3.RAW
Full ms [151.500 - 185.500] - Range: 166.000 - 166.500
Scan No. 1 of 1

Mass	Absolute Intensity	Relative Intensity	Theoretical Mass	Delta [ppm]	Delta [mmu]	RDB	Composition
166.09940	1889354	74.8	166.09883	3.4	0.6	4.0	$C_{10}H_{14}O_2$

HRMS (EI) of 43b [M]$^+$:

Wu – 8 – 86 HR (EI)

File : D:\Xcalibur\data\2004\20040707eihr-av5.RAW
Full ms [150.500 - 185.500] - Range: 168.000 - 170.000
Scan No. 1 of 1

Mass	Absolute Intensity	Relative Intensity	Theoretical Mass	Delta [ppm]	Delta [mmu]	RDB	Composition
168.11107	140191488	86.4	168.11138	-1.9	-0.3	4.0	$C_{10}H_{12}{}^2H_2O_2$
169.11731	144687360	89.2	169.11766	-2.1	-0.4	4.0	$C_{10}H_{11}{}^2H_3O_2$

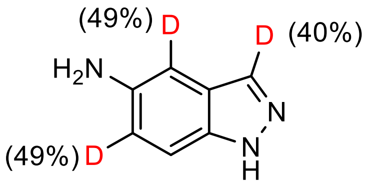

44b

According to GP, Fe-Cellulose-1000 (62 mg, 0.05 mol), substrate (33 mg, 0.25 mmol), D_2O (1.5 mL), H_2 (20 bar), room temperature to 120 °C and then at 120 °C for 24 h. The product **44b** (30 mg, 0.22 mmol, 88%) was obtained.

^{1}H NMR (300 MHz, DMSO-d_6) δ 7.84 – 7.56 (m, 0.60H), 7.25 (d, J = 8.7 Hz, 1H), 6.78 (d, J = 8.8 Hz, 1.02H), 4.73 (s, 1H).

^{13}C NMR (75 MHz, DMSO-d_6) δ 142.12, 134.40, 131.39, 123.84, 118.08, 110.18, 101.57, 100.37.

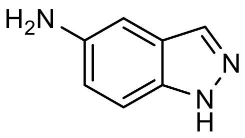

Chemical Formula: $C_7H_7N_3$
Exact Mass: 133.0640

HRMS (ESI-TOF) of 44a:

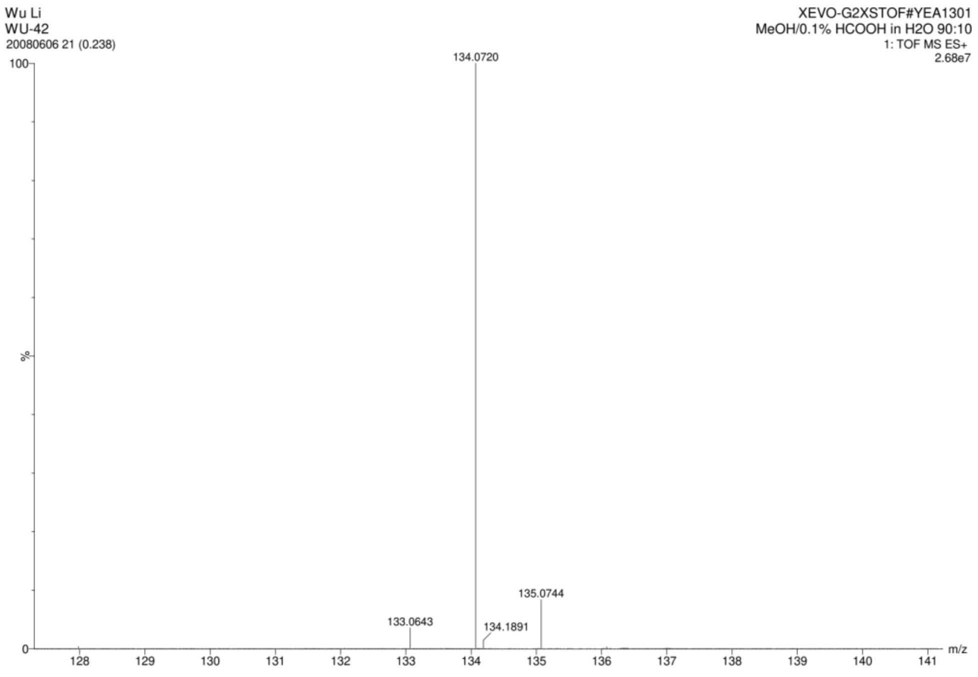

HRMS (ESI-TOF) of 44b [M+H]$^+$:

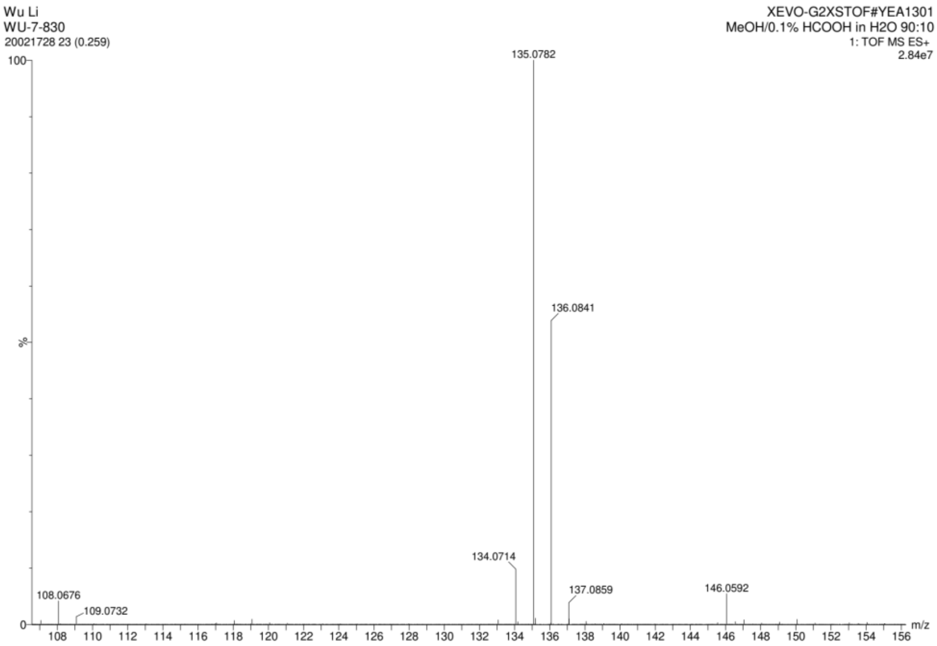

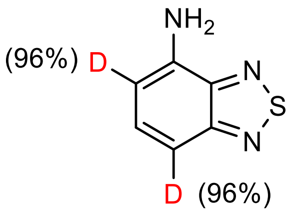

45b

According to GP, Fe-Cellulose-1000 (63 mg, 0.05 mol), substrate (40 mg, 0.26 mmol), D_2O (1.5 mL), H_2 (20 bar), room temperature to 120 °C and then at 120 °C for 24 h. The product **45b** (38 mg, 0.25 mmol, 96%) was obtained.

^{1}H NMR (300 MHz, DMSO-d_6) δ 7.40 (s, 1H), 7.15 (m, 0.04H), 6.59 (m, 0.04H), 6.18 (d, J = 4.8 Hz, 2H).

^{13}C NMR (101 MHz, DMSO-d_6) δ 155.98, 147.74, 147.71, 141.38, 141.32, 141.25, 132.35, 107.11, 106.86, 106.61, 105.26, 105.02, 104.79.

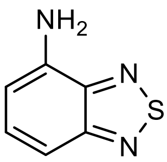

Chemical Formula: $C_6H_5N_3S$
Exact Mass: 151.0204

HRMS (ESI-TOF) of 45a:

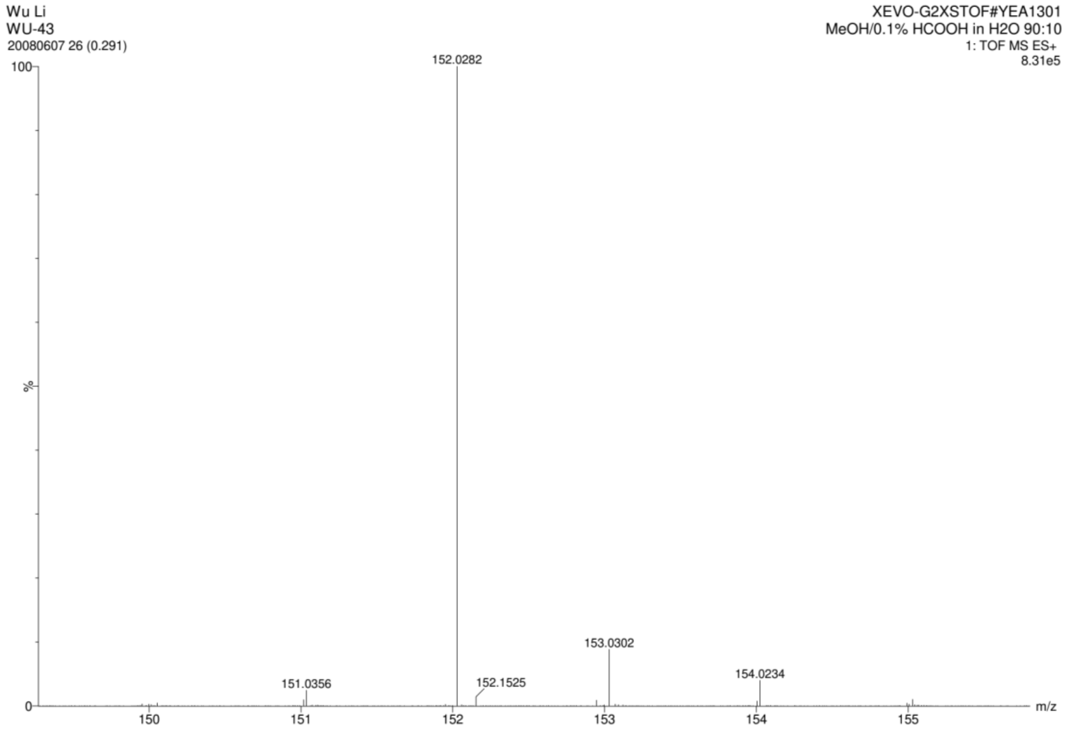

HRMS (ESI-TOF) of 45b [M+H]$^+$:

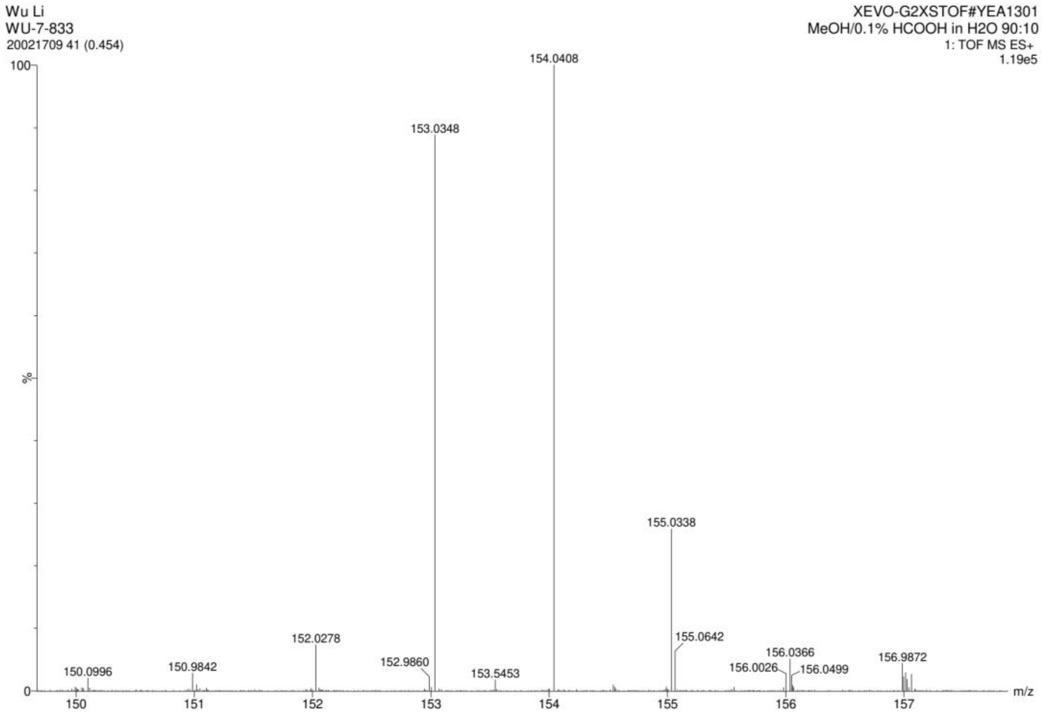

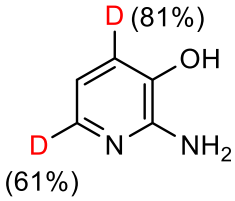

46b

According to GP, Fe-Cellulose-1000 (59 mg, 0.05 mol), substrate (28 mg, 0.25 mmol), D_2O (1.5 mL), H_2 (20 bar), room temperature to 120 °C and then at 120 °C for 72 h. The product **46b** (26 mg, 0.23 mmol, 92%) was obtained.

^{1}H NMR (300 MHz, DMSO-d_6) δ 9.42 (s, 1H), 7.42 (m, 0.39H), 6.83 (m, 0.19H), 6.38 (q, J = 2.4, 1.7 Hz, 1H), 5.37 (s, 2H).

^{13}C NMR (75 MHz, DMSO-d_6) δ 150.85, 139.52, 137.82, 118.95, 112.77, 112.62.

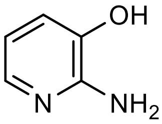

Chemical Formula: $C_5H_6N_2O$
Exact Mass: 110.0480

HRMS (ESI-TOF) of 46a:

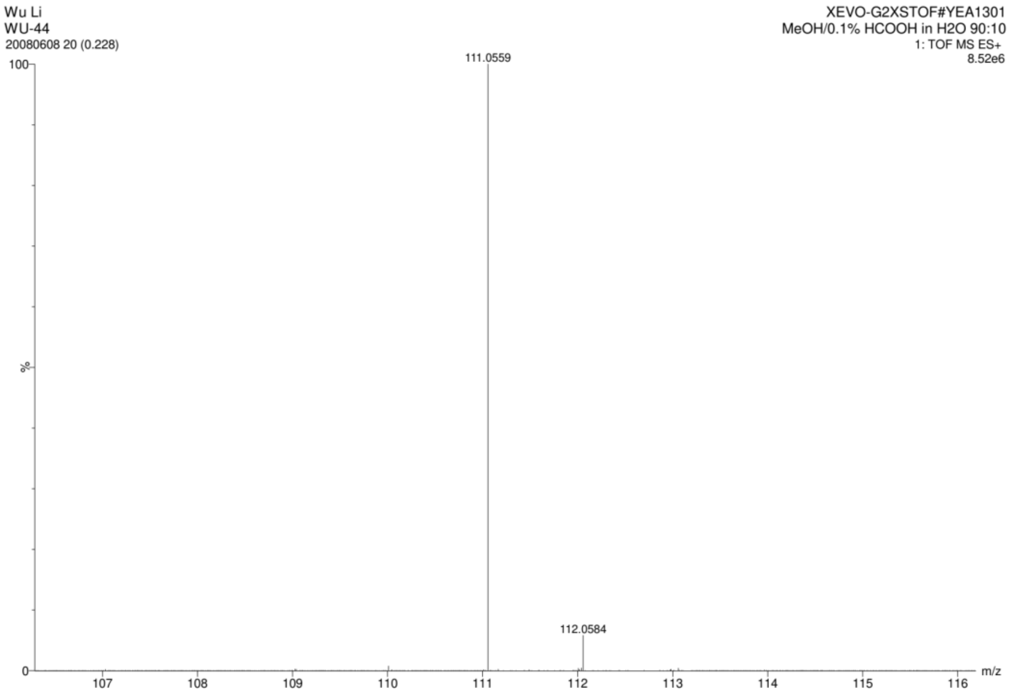

HRMS (ESI-TOF) of 46b [M+H]$^+$:

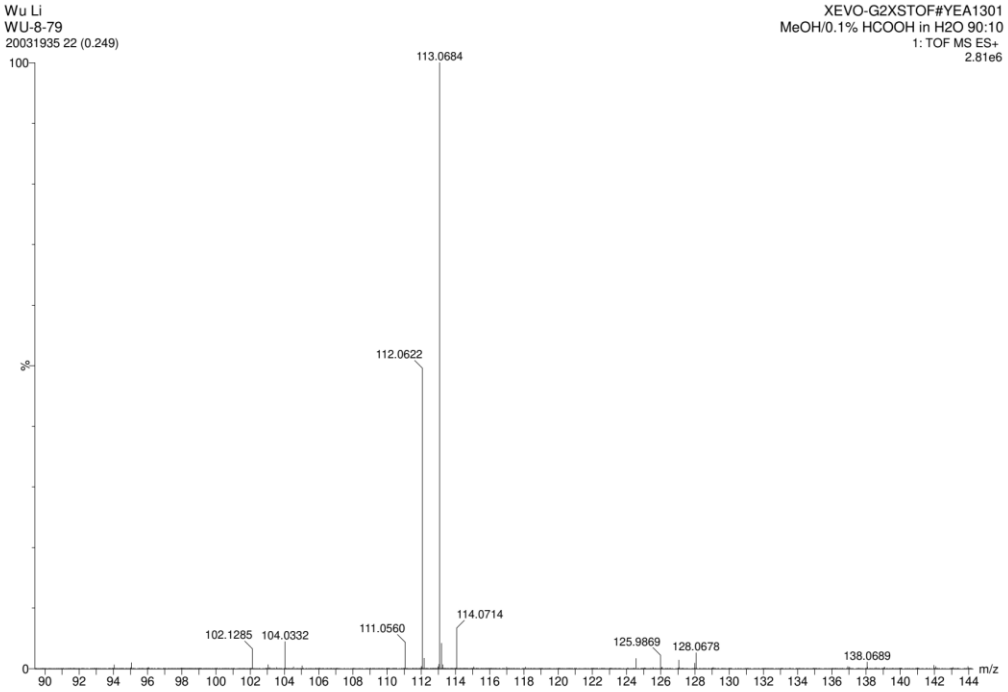

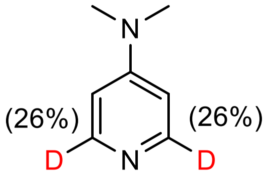

47b

According to GP, Fe-Cellulose-1000 (60 mg, 0.05 mol), substrate (30 mg, 0.25 mmol), D_2O (1.5 mL), H_2 (20 bar), room temperature to 120 °C and then at 120 °C for 72 h. The product **47b** (26 mg, 0.21 mmol, 84%) was obtained.

^{1}H NMR (300 MHz, DMSO-d_6) δ 7.70 – 7.61 (m, 1.49H), 6.22 – 6.06 (m, 2H), 2.50 (s, 6H).

^{13}C NMR (75 MHz, DMSO-d_6) δ 153.93, 149.36, 106.70, 106.57, 38.58.

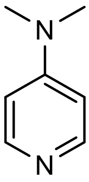

Chemical Formula: $C_7H_{10}N_2$
Exact Mass: 122.0844

HRMS (ESI-TOF) of 47a:

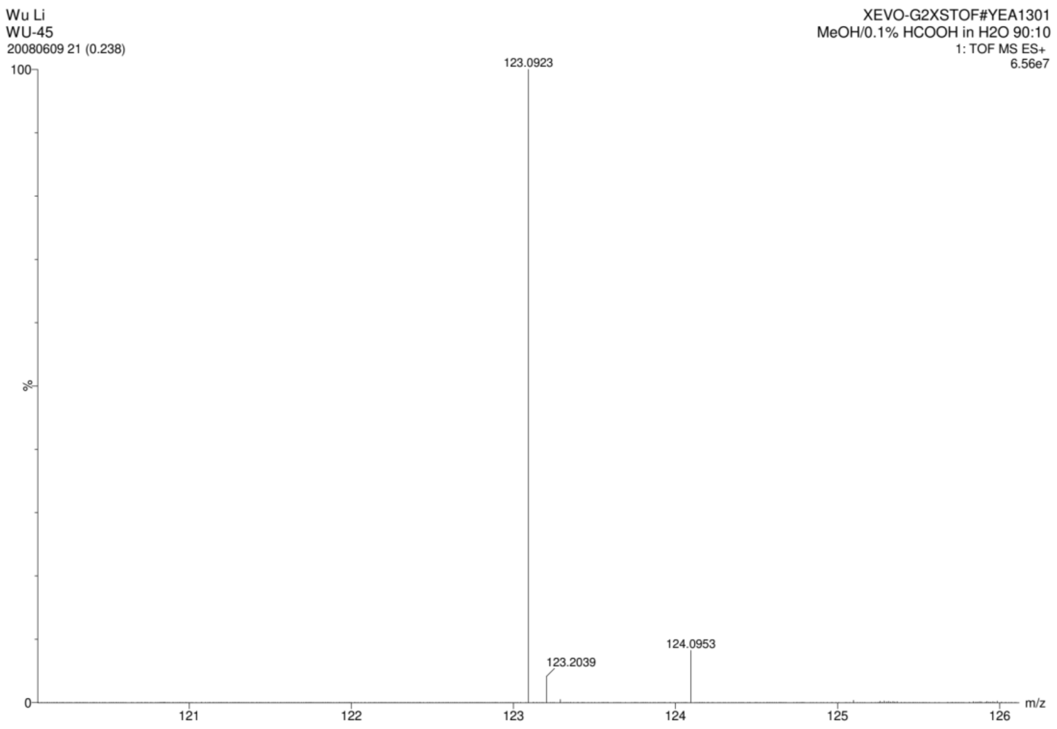

HRMS (ESI-TOF) of 47b $[M+H]^+$:

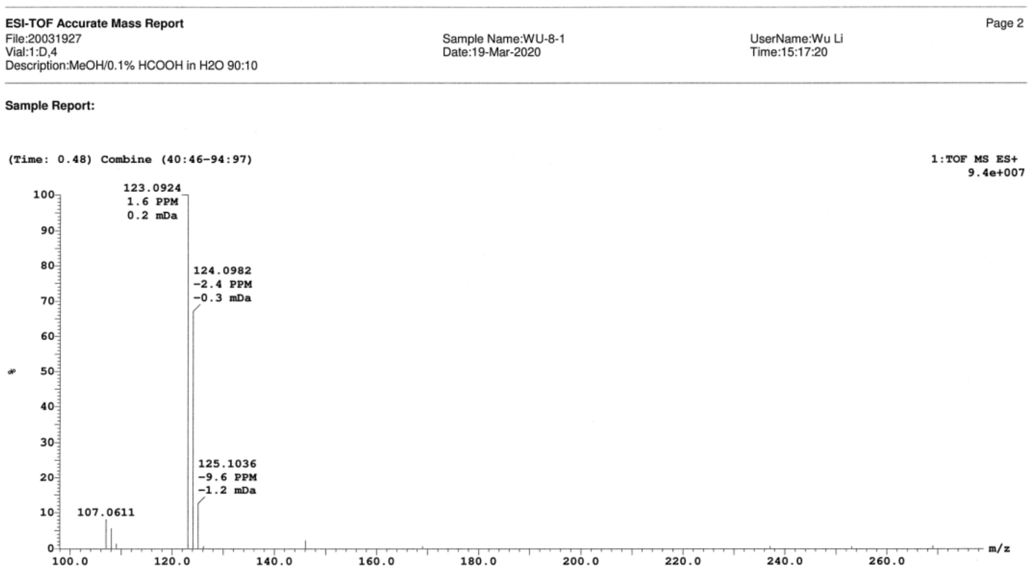

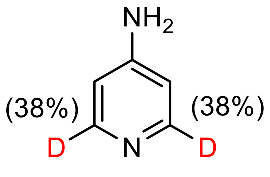

48b

According to GP, Fe-Cellulose-1000 (60 mg, 0.05 mol), substrate (35 mg, 0.37 mmol), D_2O (1.5 mL), H_2 (20 bar), room temperature to 140 °C and then at 140 °C for 24 h. The product **48b** (29 mg, 0.31 mmol, 84%) was obtained.
^{1}H NMR (300 MHz, DMSO-d_6) δ 8.11 – 7.88 (m, 1.24H), 6.65 – 6.29 (m, 2H), 5.98 (s, 2H).
^{13}C NMR (75 MHz, DMSO-d_6) δ 154.68, 149.92, 109.31, 109.26, 109.17.

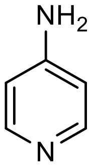

Chemical Formula: $C_5H_6N_2$
Exact Mass: 94.0531

HRMS (ESI-TOF) of 48a:

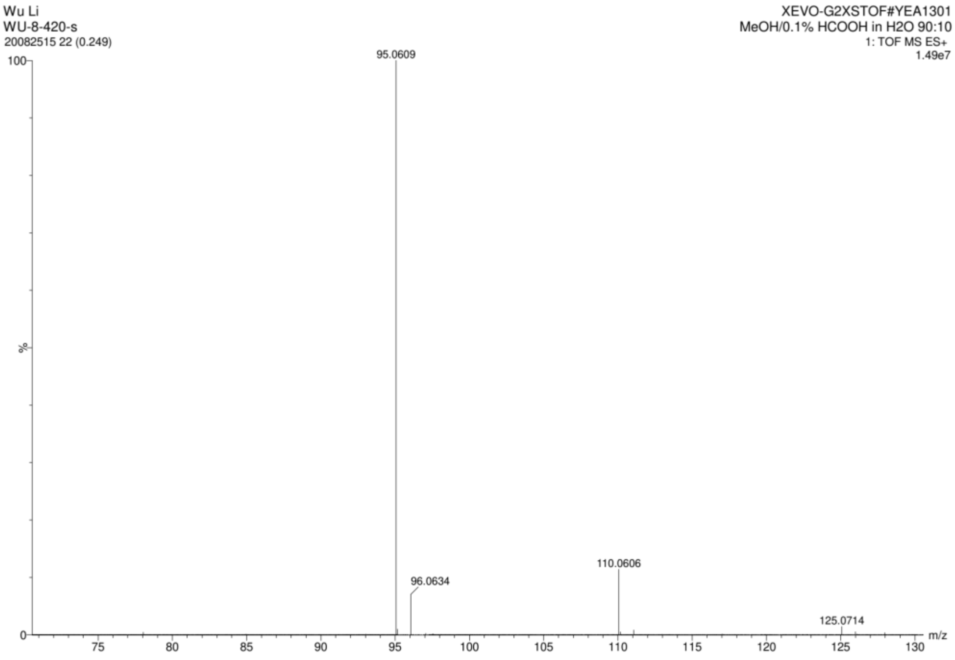

HRMS (ESI-TOF) of 48b [M+H]$^+$:

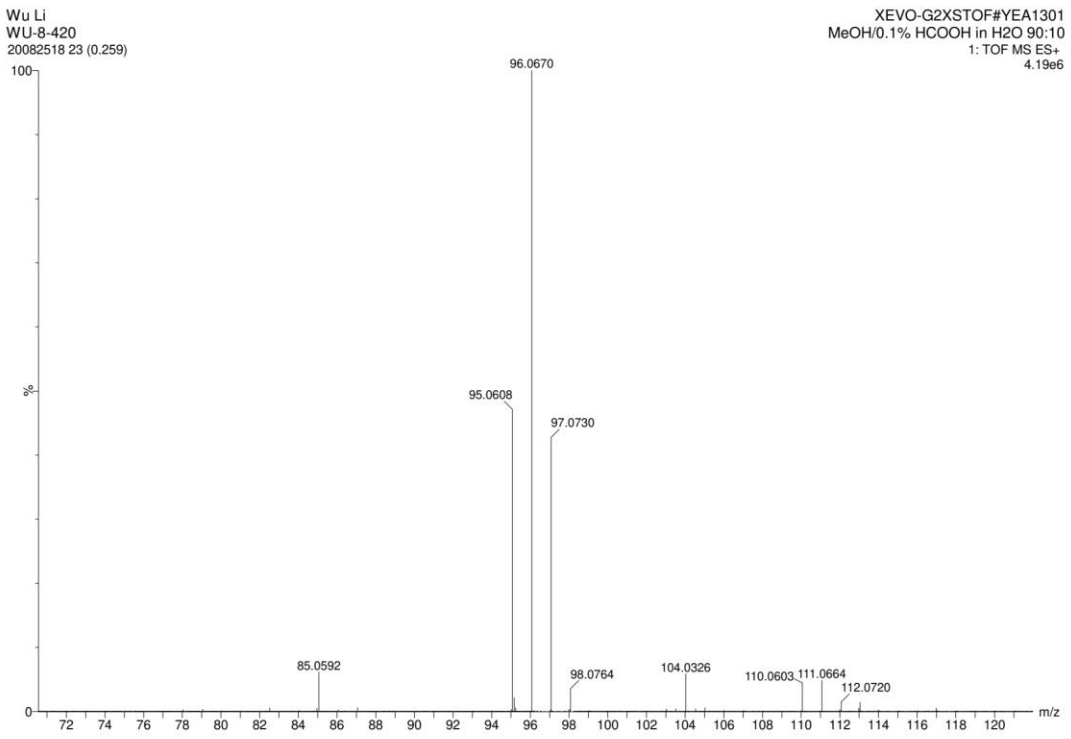

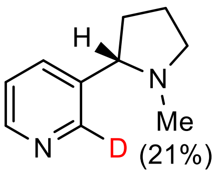

49b

According to GP, Fe-Cellulose-1000 (60 mg, 0.05 mol), substrate (45 mg, 0.28 mmol), D_2O (1.5 mL), H_2 (20 bar), room temperature to 140 °C and then at 140 °C for 24 h. The product **49b** (41 mg, 0.25 mmol, 89%) was obtained.

^{1}H NMR (300 MHz, DMSO-d_6) δ 8.50 (dd, J = 2.3, 0.9 Hz, 0.79H), 8.45 (dd, J = 4.8, 1.7 Hz, 1H), 7.72 (dt, J = 7.8, 2.0 Hz, 1H), 7.35 (ddd, J = 7.9, 4.8, 0.9 Hz, 1H), 3.20 – 3.03 (m, 2H), 2.29 – 2.13 (m, 2H), 2.07 (s, 3H), 1.95 – 1.67 (m, 2H), 1.65 – 1.53 (m, 1H).

^{13}C NMR (75 MHz, DMSO-d_6) δ 149.42, 148.82, 139.23, 135.27, 124.14, 68.43, 56.81, 35.46, 22.68.

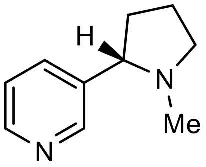

Chemical Formula: $C_{10}H_{14}N_2$
Exact Mass: 162.1157

HRMS (ESI-TOF) of 49a unlabelled:

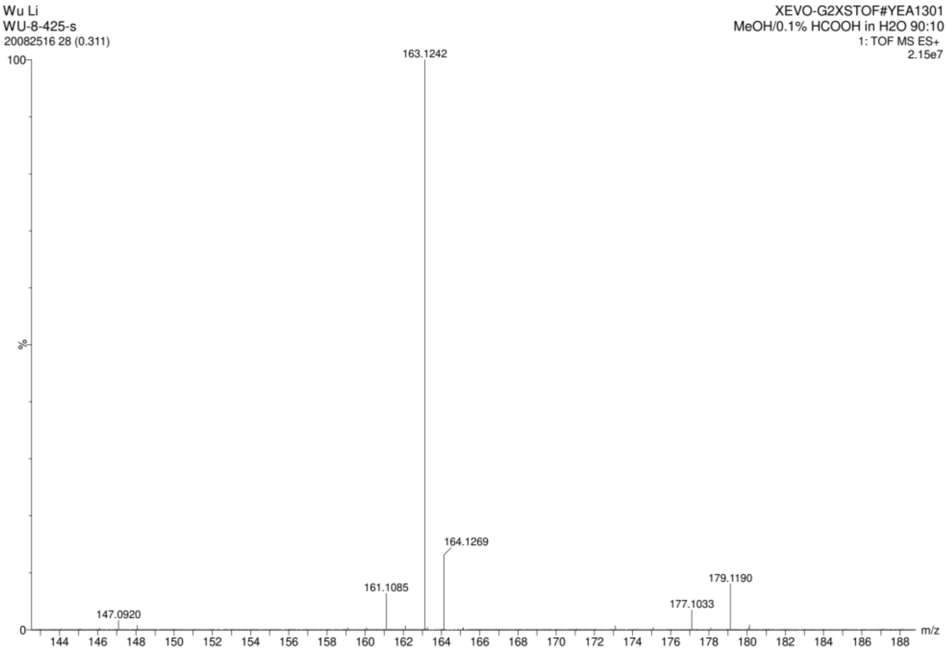

HRMS (ESI-TOF) of 49b [M+H]$^+$:

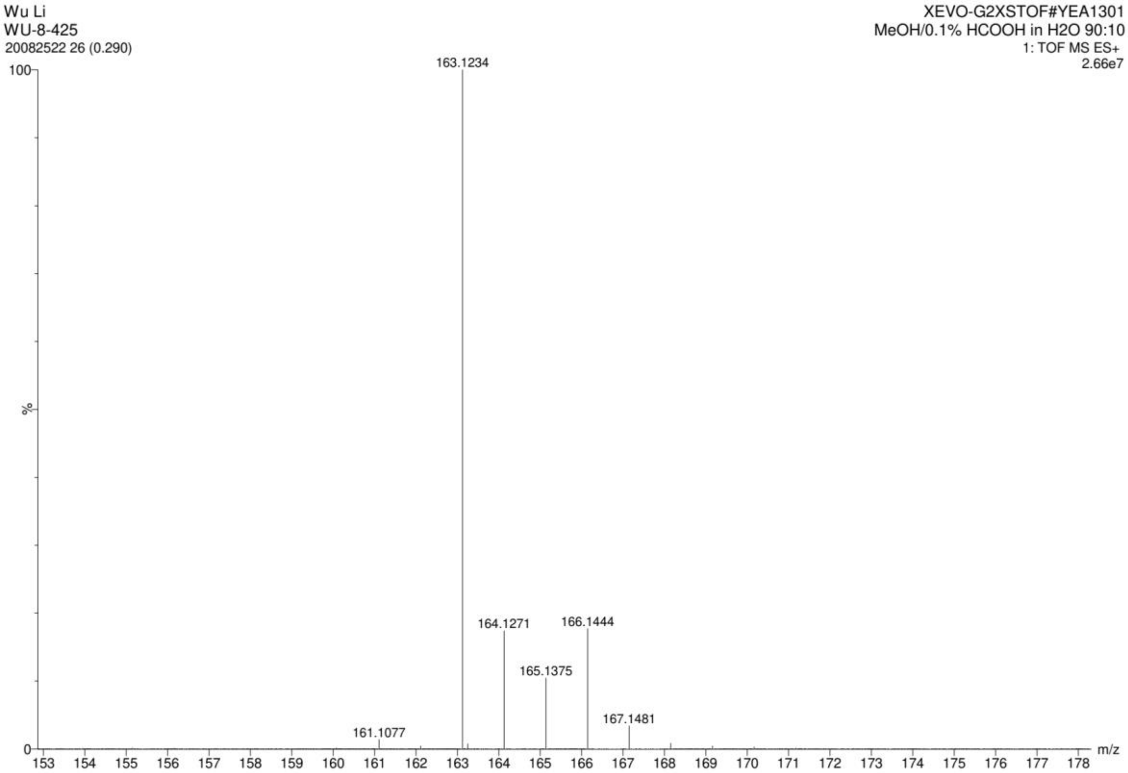

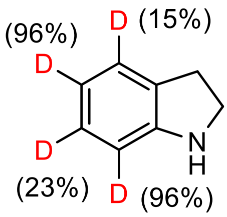

50b

According to GP, Fe-Cellulose-1000 (59 mg, 0.05 mol), substrate (37 mg, 0.31 mmol), D_2O (1.5 mL), H_2 (20 bar), room temperature to 120 °C and then at 120 °C for 24 h. The product **50b** (37 mg, 0.30 mmol, 97%) was obtained.

^{1}H NMR (400 MHz, DMSO-d_6) δ 7.02 (s, 0.85H), 6.90 (s, 0.77H), 6.52 (m, 0.09H), 5.41 (s, 1H), 3.39 (t, J = 8.5 Hz, 2H), 2.89 (t, J = 8.5 Hz, 2H).

^{13}C NMR (101 MHz, DMSO-d_6) δ 152.92, 129.15, 127.24, 127.13, 125.52, 124.63, 124.52, 121.32, 121.22, 120.43, 120.31, 119.21, 117.28, 117.03, 116.79, 108.83, 108.58, 108.34, 46.89, 29.70.

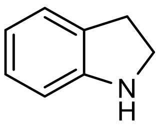

Chemical Formula: C_8H_9N
Exact Mass: 119.0735

HRMS (ESI-TOF) of 50a:

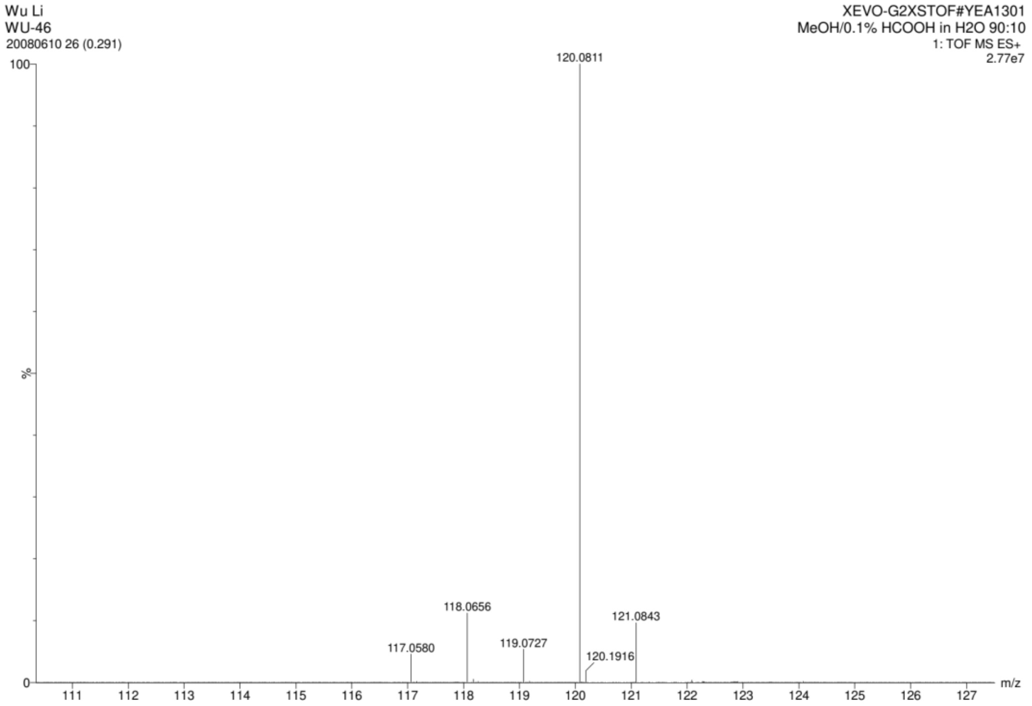

HRMS (ESI-TOF) of 50b [M+H]$^+$:

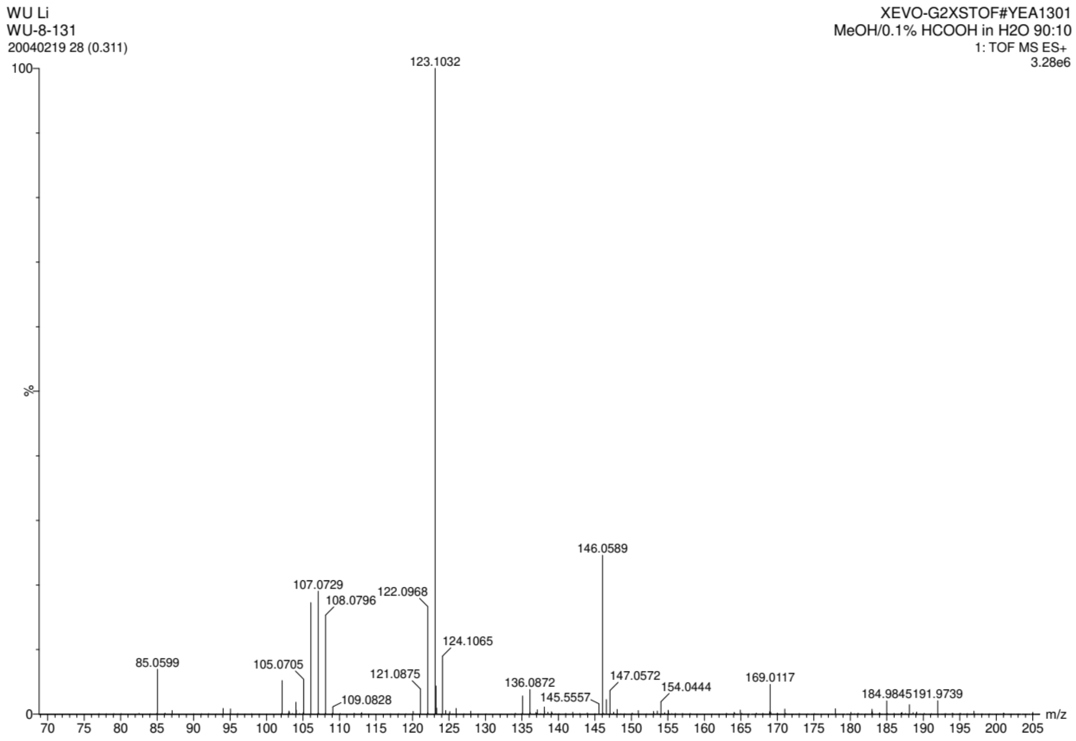

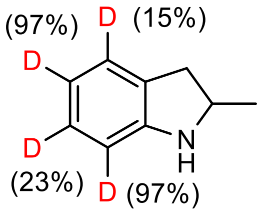

51b

According to GP, Fe-Cellulose-1000 (60 mg, 0.05 mol), substrate (36 mg, 0.27 mmol), D_2O (1.5 mL), H_2 (20 bar), room temperature to 120 °C and then at 120 °C for 24 h. The product **51b** (35 mg, 0.26 mmol, 96%) was obtained.

^{1}H NMR (300 MHz, DMSO-d_6) δ 7.02 – 6.93 (m, 0.85H), 6.87 (s, 0.77H), 6.48 (m, 0.06H), 3.83 (dtt, J = 8.2, 6.1, 1.8 Hz, 1H), 3.02 (dd, J = 15.5, 8.6 Hz, 1H), 2.48 – 2.49 (m, 1H), 1.17 (d, J = 6.1 Hz, 3H).

^{13}C NMR (101 MHz, DMSO-d_6) δ 152.23, 128.57, 127.27, 127.16, 124.69, 124.58, 117.10, 116.85, 116.60, 108.39, 108.14, 107.90, 54.85, 37.76, 22.59.

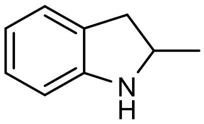

Chemical Formula: $C_9H_{11}N$
Exact Mass: 133.0891

HRMS (ESI-TOF) of 51a:

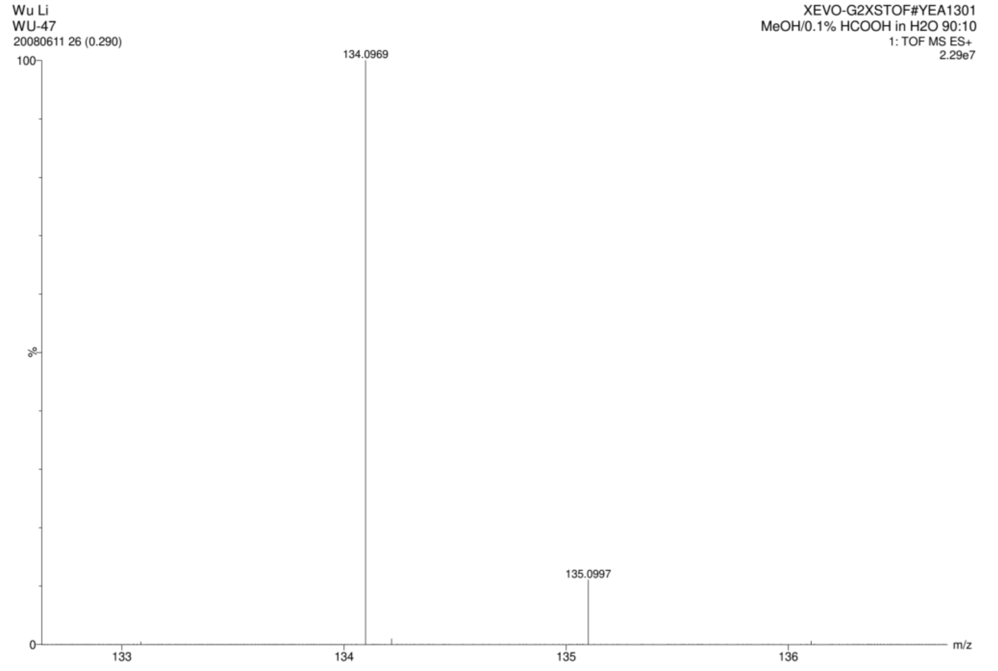

HRMS (ESI-TOF) of 51b [M+H]⁺:

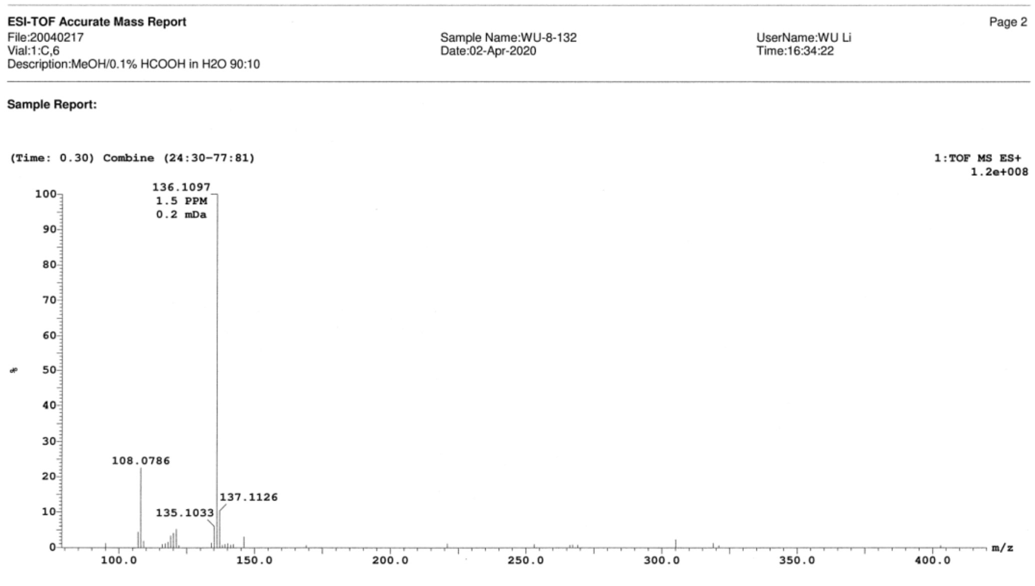

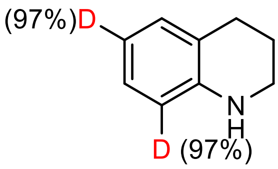

52b

According to GP, Fe-Cellulose-1000 (60 mg, 0.05 mol), substrate (43 mg, 0.32 mmol), D_2O (1.5 mL), H_2 (20 bar), room temperature to 120 °C and then at 120 °C for 24 h. The product **52b** (40 mg, 0.30 mmol, 94%) was obtained.

^{1}H NMR (300 MHz, $CDCl_3$) δ 7.12 – 6.83 (m, 2H), 6.65 (m, 0.03H), 6.50 (m, 0.03H), 3.62 (s, 1H), 3.38 – 3.15 (m, 2H), 2.79 (t, *J* = 6.4 Hz, 2H), 2.10 – 1.86 (m, 2H).

^{13}C NMR (101 MHz, DMSO-d_6) δ 145.77, 145.73, 129.37, 129.26, 126.70, 126.58, 120.27, 115.47, 115.22, 114.98, 113.75, 113.51, 113.28, 41.30, 41.17, 27.21, 27.19, 22.08, 22.06.

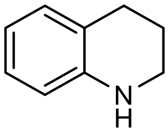

Chemical Formula: $C_9H_{11}N$
Exact Mass: 133.0891

HRMS (ESI-TOF) of 52a:

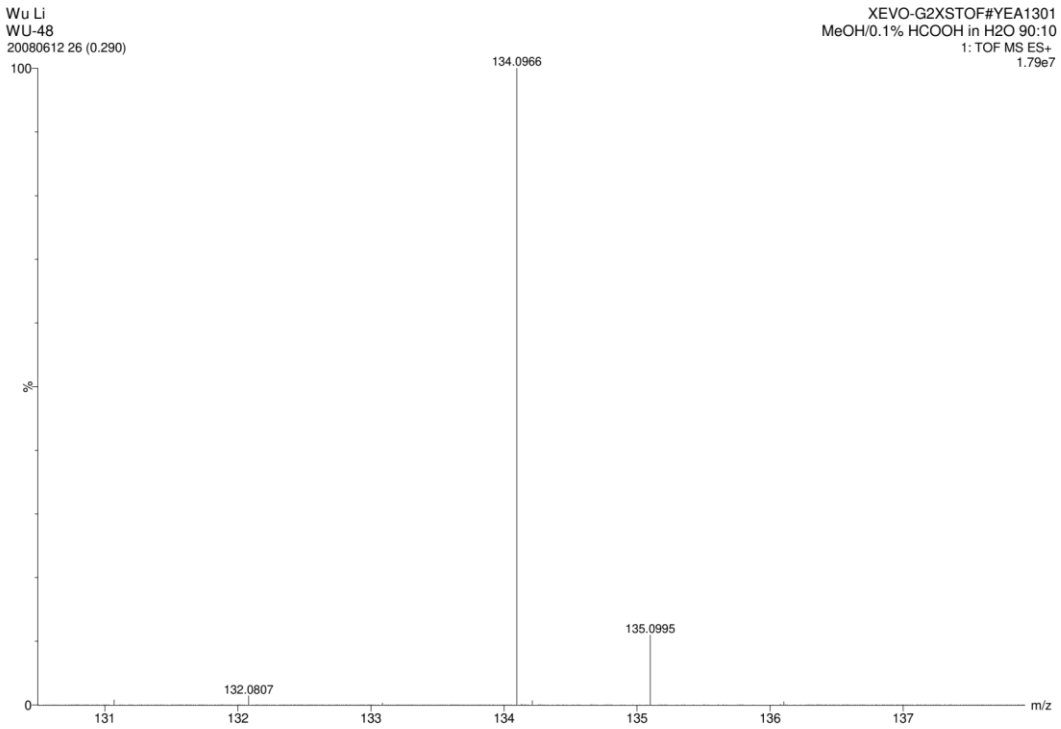

HRMS (ESI-TOF) of 52b [M+H]+:

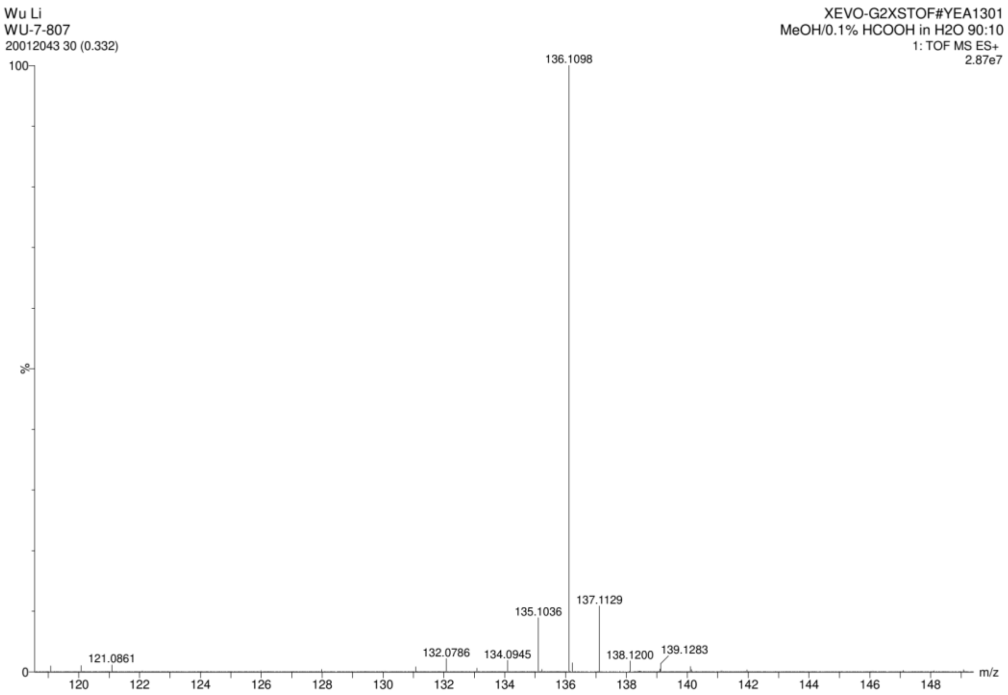

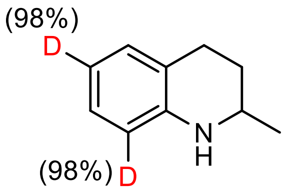

53b

According to GP, Fe-Cellulose-1000 (61 mg, 0.05 mol), substrate (37 mg, 0.25 mmol), D_2O (1.5 mL), H_2 (20 bar), room temperature to 120 °C and then at 120 °C for 24 h. The product **53b** (34 mg, 0.23 mmol, 92%) was obtained.

^{1}H NMR (300 MHz, $CDCl_3$) δ 6.97 (s, 2H), 6.71 (m, 0.02H), 6.50 (m, 0.02H), 3.60 (s, 1H), 3.41 (ddt, *J* = 9.8, 6.4, 3.2 Hz, 1H), 3.01 – 2.56 (m, 2H), 1.95 (ddt, *J* = 12.4, 6.0, 3.2 Hz, 1H), 1.61 (dddd, *J* = 12.9, 11.3, 9.9, 5.5 Hz, 1H), 1.23 (d, *J* = 6.3 Hz, 3H).

^{13}C NMR (101 MHz, DMSO-d_6) δ 145.80, 145.75, 129.16, 129.06, 126.63, 126.52, 120.01, 115.61, 115.36, 113.72, 113.47, 113.24, 46.64, 46.51, 46.31, 46.18, 30.19, 26.56, 26.54, 26.35, 22.64, 22.60.

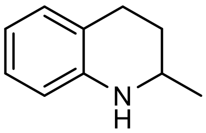

Chemical Formula: $C_{10}H_{13}N$
Exact Mass: 147.1048

HRMS (ESI-TOF) of 53a:

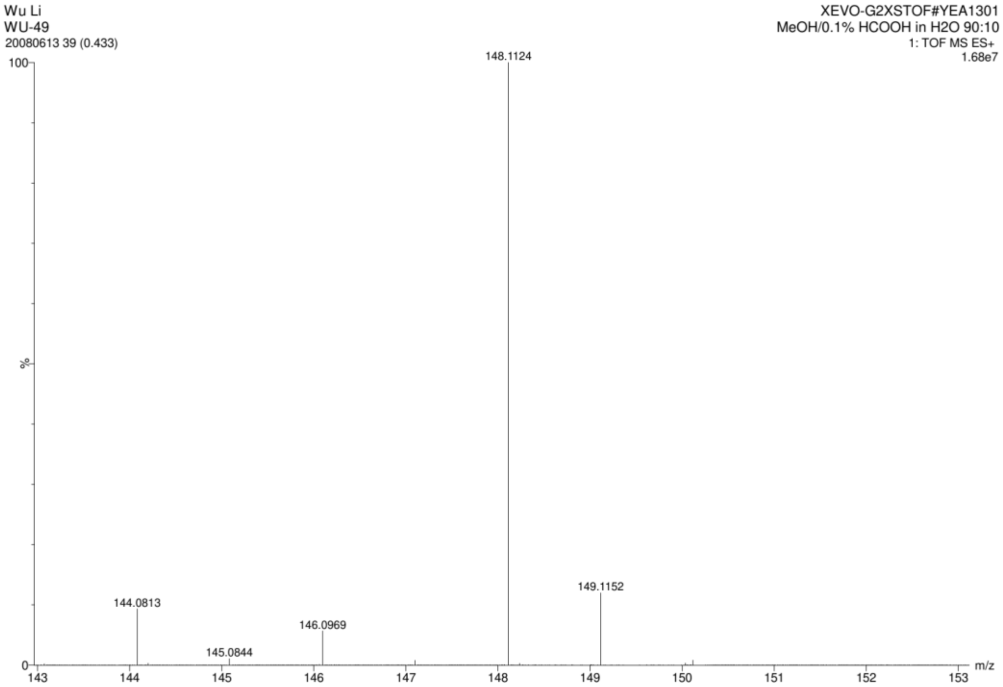

HRMS (ESI-TOF) of 53b $[M+H]^+$:

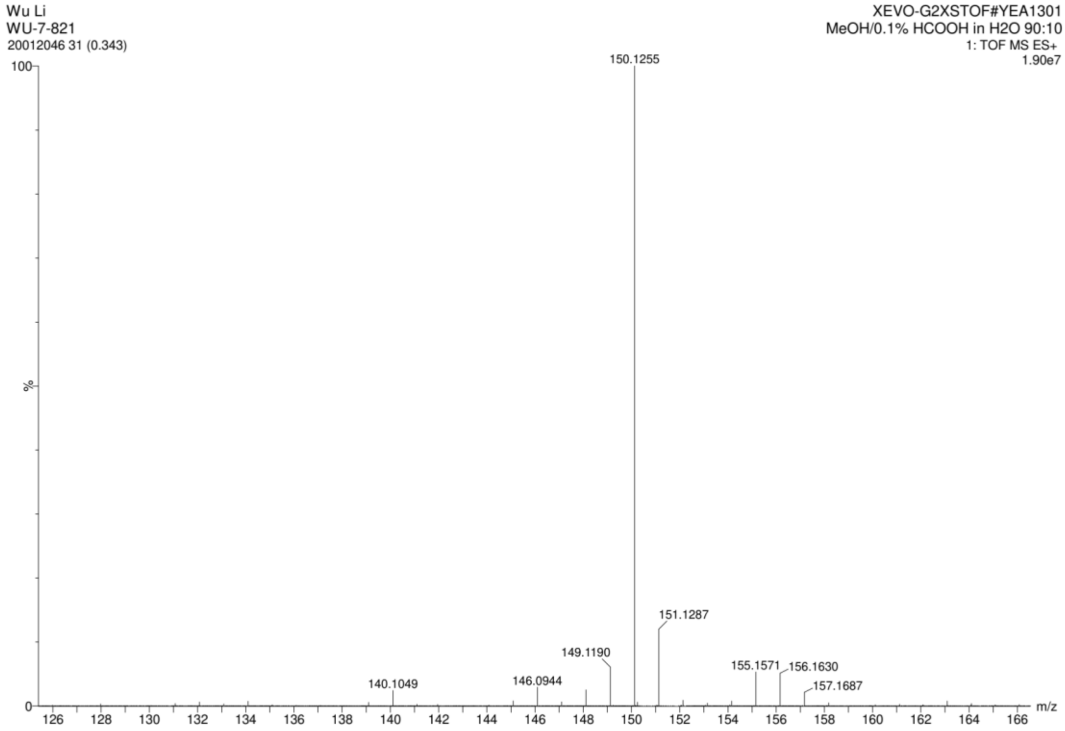

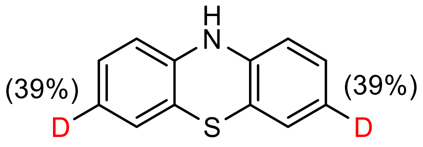

54b

According to GP, Fe-Cellulose-1000 (59 mg, 0.05 mol), substrate (50 mg, 0.25 mmol), D_2O (1.5 mL), H_2 (20 bar), room temperature to 120 °C and then at 120 °C for 24 h. The product **54b** (49 mg, 0.25 mmol, 99%) was obtained.

^{1}H NMR (300 MHz, DMSO-d_6) δ 7.06 – 6.95 (m, 2H), 6.95 – 6.87 (m, 2H), 6.77 (dt, J = 7.6, 1.2 Hz, 1.23H), 6.74 – 6.65 (m, 2H).

^{13}C NMR (75 MHz, DMSO-d_6) δ 142.09, 142.03, 127.51, 127.40, 127.30, 126.22, 126.12, 121.75, 116.32, 114.41, 114.35.

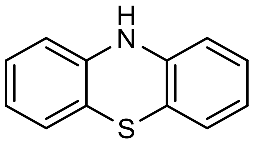

Chemical Formula: $C_{12}H_9NS$
Exact Mass: 199.0456

HRMS (ESI-TOF) of 54a:

File : D:\Xcalibur\data\2009\20090110hrei-av2.RAW
Full ms [189.500 - 223.500] - Range: 199.000 - 199.500
Scan No. 1 of 1

Mass	Absolute Intensity	Relative Intensity	Theoretical Mass	Delta [ppm]	Delta [mmu]	RDB	Composition
199.04508	7902745	100.0	199.04502	0.3	0.1	9.0	$C_{12}H_9N_1S_1$

HRMS (ESI-TOF) of 54b [M+H]$^+$:

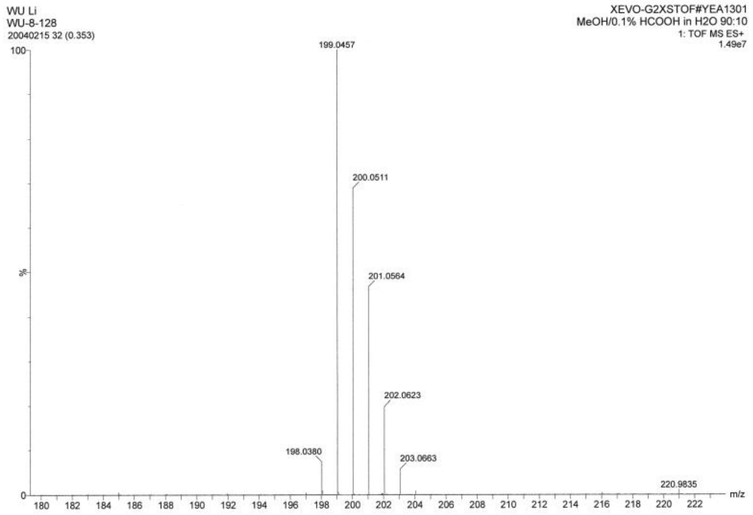

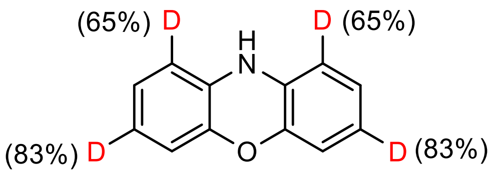

55b

According to GP, Fe-Cellulose-1000 (59 mg, 0.05 mol), substrate (47 mg, 0.26 mmol), D_2O (1.5 mL), H_2 (20 bar), room temperature to 120 °C and then at 120 °C for 24 h. The product **55b** (40 mg, 0.25 mmol, 96%) was obtained.

^{1}H NMR (300 MHz, DMSO-d_6) δ 8.16 (s, 1H), 6.71 (dq, J = 3.5, 1.6 Hz, 2H), 6.64 – 6.52 (m, 2.34H), 6.45 (dd, J = 7.8, 1.2 Hz, 0.71H).

^{13}C NMR (75 MHz, DMSO-d_6) δ 142.75, 132.41, 132.34, 132.27, 123.89, 123.77, 123.67, 120.31, 115.04, 114.94, 113.26, 113.21.

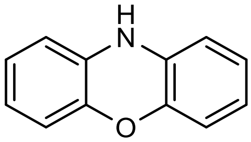

Chemical Formula: $C_{12}H_9NO$
Exact Mass: 183.0684

HRMS (ESI-TOF) of 55a:

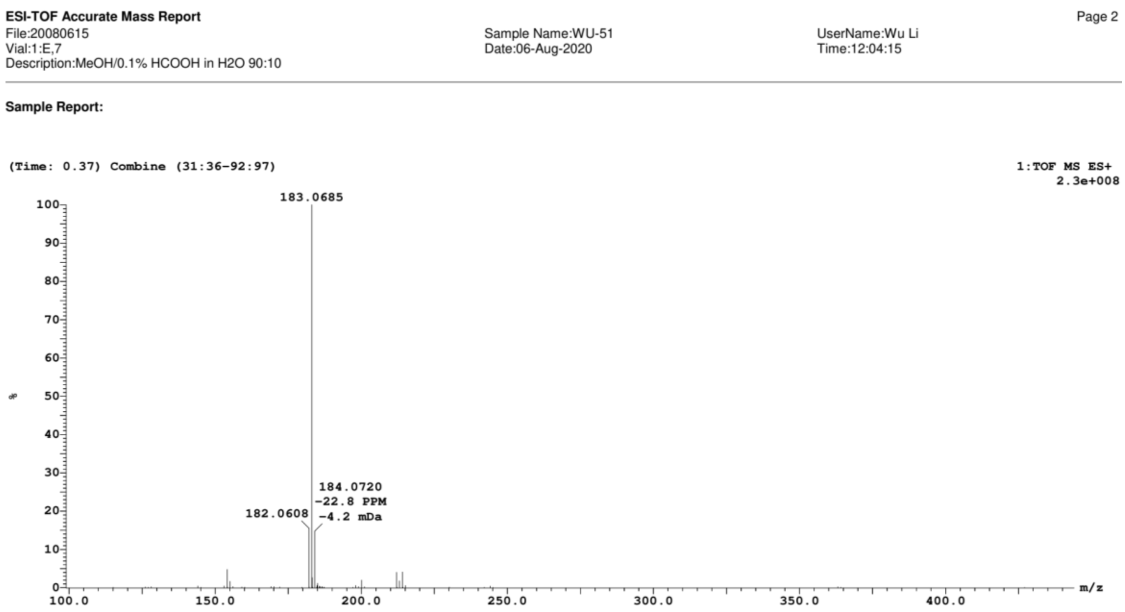

HRMS (ESI-TOF) of 55b [M+H]$^+$:

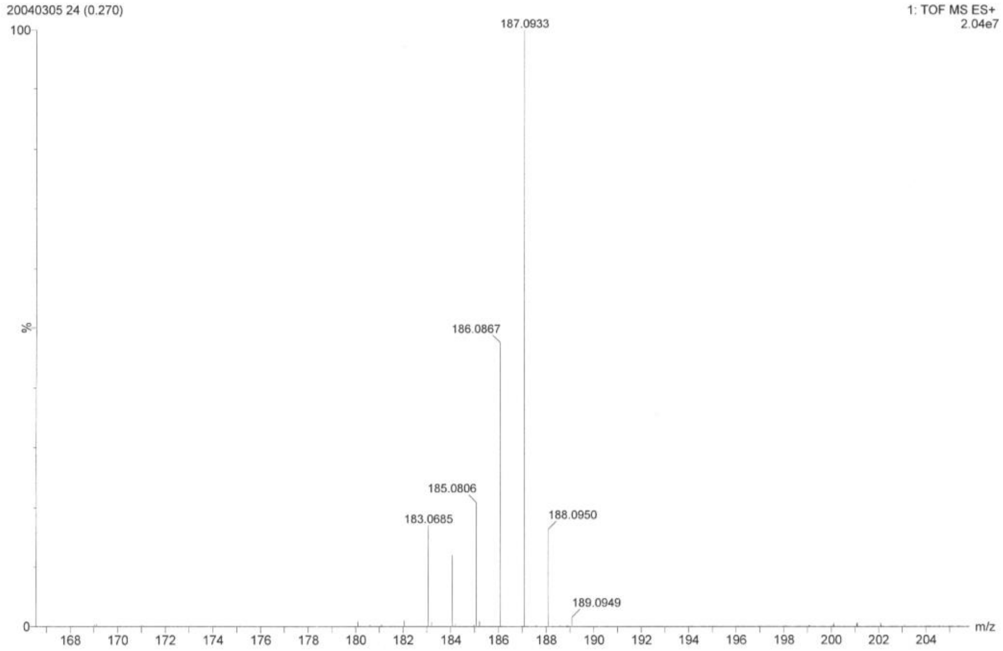

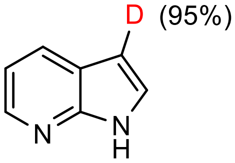

56b

According to GP, Fe-Cellulose-1000 (60 mg, 0.05 mol), substrate (35 mg, 0.30 mmol), D_2O (1.5 mL), H_2 (20 bar), room temperature to 120 °C and then at 120 °C for 24 h. The product **56b** (32 mg, 0.27 mmol, 90%) was obtained.

^1H NMR (300 MHz, $CDCl_3$) δ 11.70 (s, 1H), 8.37 (dd, *J* = 4.8, 1.6 Hz, 1H), 7.99 (dd, *J* = 7.8, 1.5 Hz, 1H), 7.41 (s, 1H), 7.11 (dd, *J* = 7.8, 4.8 Hz, 1H), 6.53 (m, 0.05H).

^{13}C NMR (101 MHz, DMSO-d_6) δ 148.90, 148.78, 142.89, 128.51, 126.34, 126.19, 120.04, 115.88, 100.33, 100.27, 100.07, 99.81.

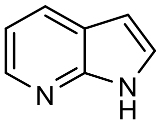

Chemical Formula: $C_7H_6N_2$
Exact Mass: 118.0531

HRMS (ESI-TOF) of 56a unlabelled:

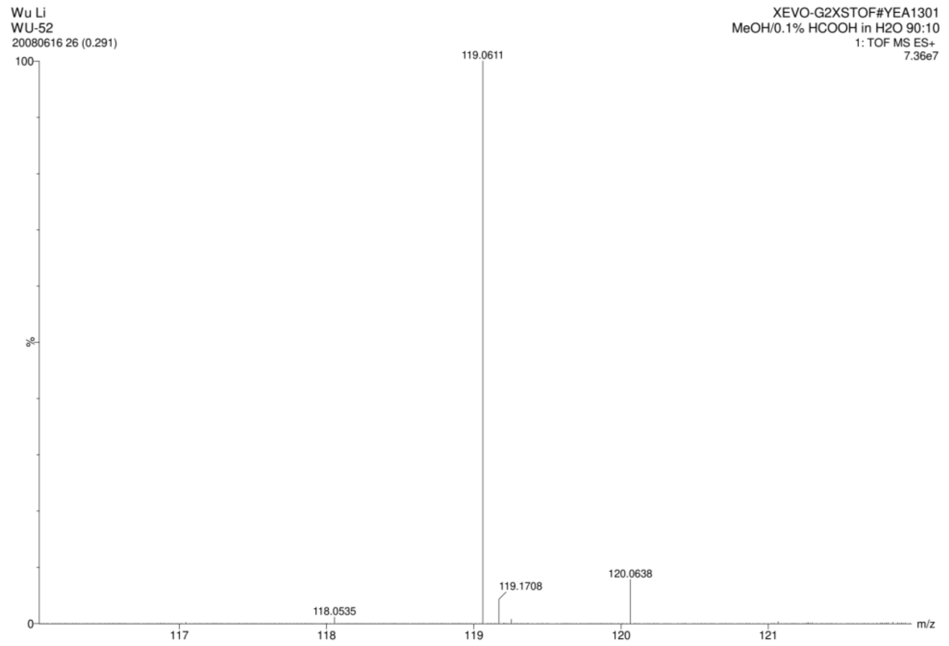

HRMS (ESI-TOF) of 56b [M+H]$^+$:

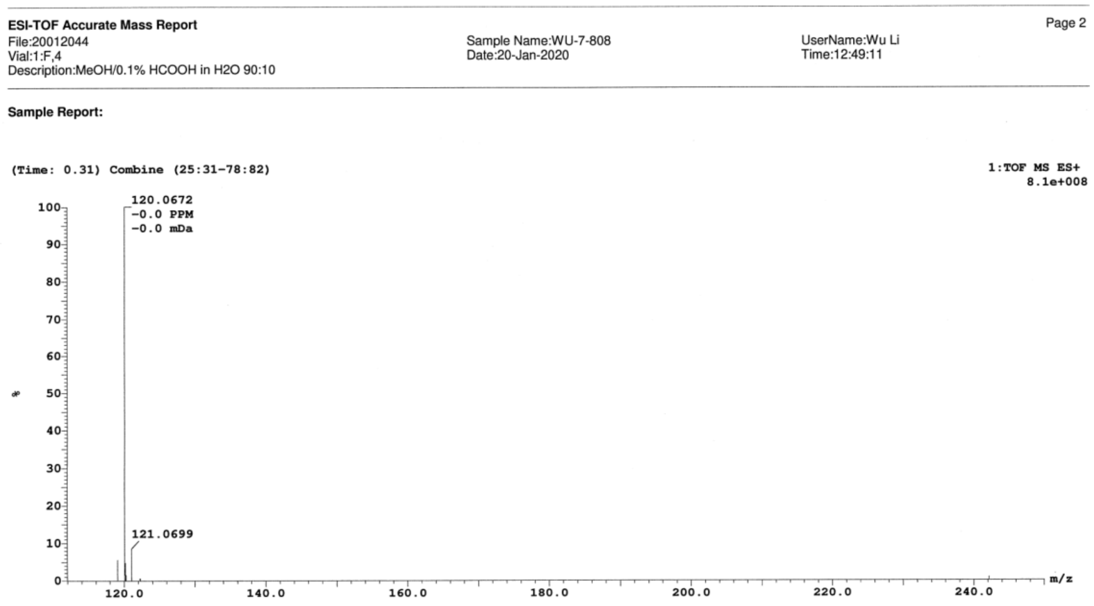

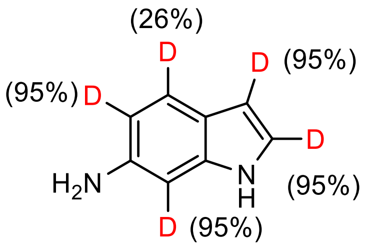

57b

According to GP, Fe-Cellulose-1000 (63 mg, 0.05 mol), substrate (32 mg, 0.24 mmol), D_2O (1.5 mL), H_2 (20 bar), room temperature to 120 °C and then at 120 °C for 24 h. The product **57b** (30 mg, 0.22 mmol, 92%) was obtained.

^{1}H NMR (400 MHz, DMSO-d_6) δ 10.43 (s, 1H), 7.17 (d, J = 0.9 Hz, 0.74H), 6.96 (m, 0.05H), 6.66 (m, 0.05H), 6.56 (m, 0.05H), 4.66 (s, 2H).

^{13}C NMR (75 MHz, DMSO-d_6) δ 143.96, 137.86, 120.31, 119.90.

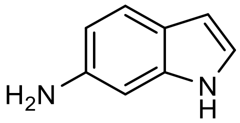

Chemical Formula: $C_8H_8N_2$
Exact Mass: 132.0687

HRMS (ESI-TOF) of 57a:

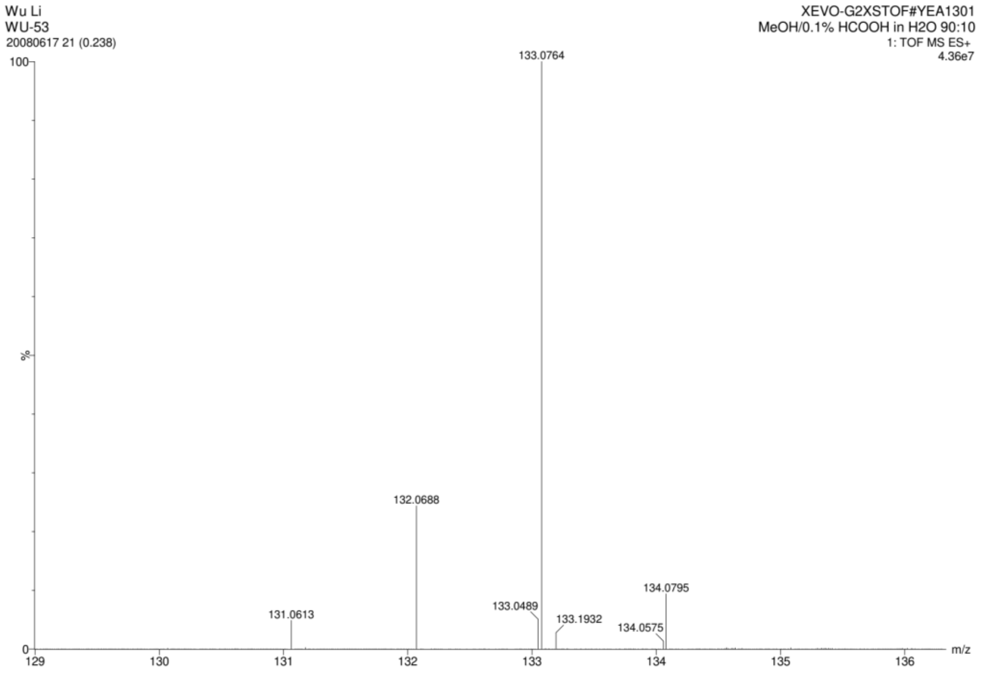

HRMS (ESI-TOF) of 57b [M+H]+:

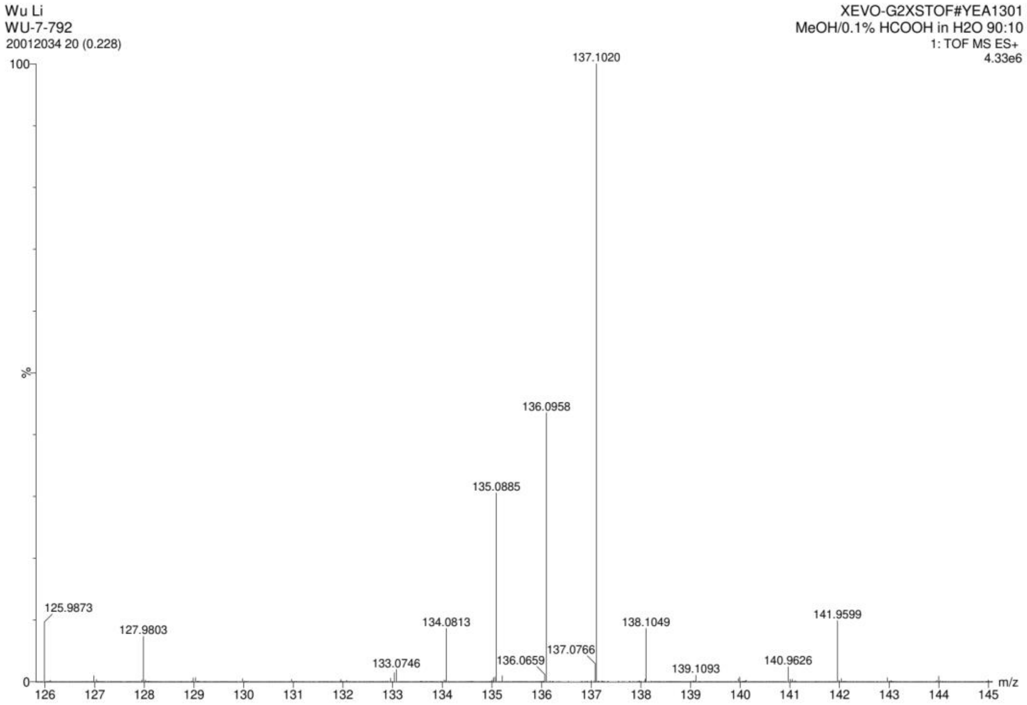

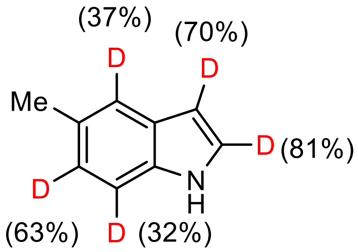

58b

According to GP, Fe-Cellulose-1000 (60 mg, 0.05 mol), substrate (33 mg, 0.25 mmol), D_2O (1.5 mL), H_2 (20 bar), room temperature to 120 °C and then at 120 °C for 24 h. The product **58b** (29 mg, 0.21 mmol, 84%) was obtained.

^{1}H NMR (300 MHz, $CDCl_3$) δ 8.01 (s, 1H), 7.47 (dd, J = 1.8, 1.0 Hz, 0.63H), 7.30 (m, 0.37H), 7.06 (d, J = 3.8 Hz, 0.68H), 6.50 (m, 0.30H), 2.48 (s, 3H).

^{13}C NMR (101 MHz, DMSO-d_6) δ 134.65, 128.29, 127.54, 127.46, 122.93, 122.82, 120.03, 111.52, 111.41, 100.74, 21.81, 21.67, 21.61.

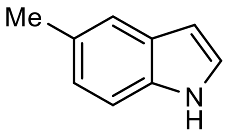

Chemical Formula: C_9H_9N
Exact Mass: 131.0735

HRMS (ESI-TOF) of 58a:

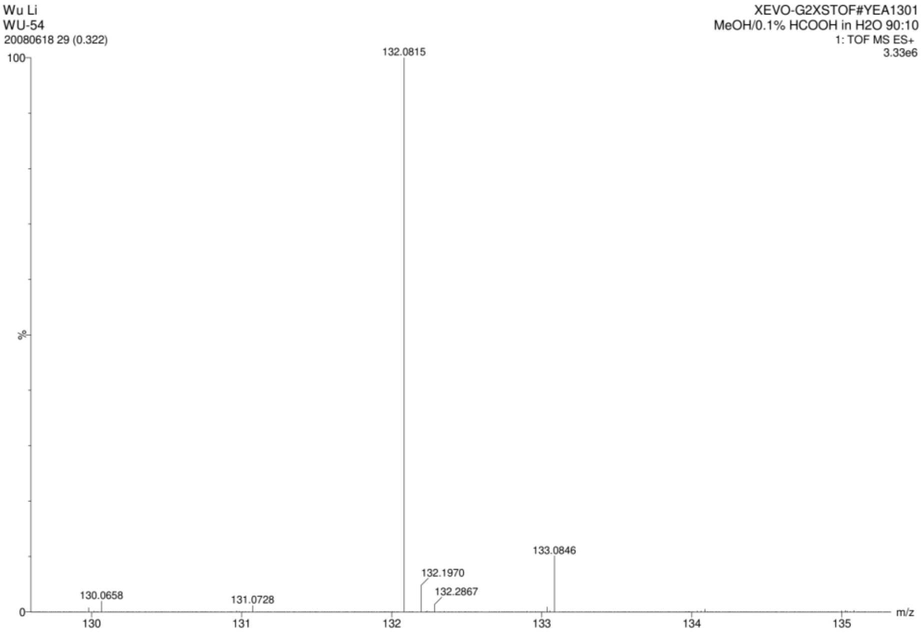

HRMS (EI) of 58b [M]⁺:

WU-7-790 HR (EI)

File : D:\Xcalibur\data\2001\20012305eihr-av2.RAW
Full ms [114.500 - 146.500] - Range: 131.000 - 146.500
Scan No. 1 of 1

Mass	Absolute Intensity	Relative Intensity	Theoretical Mass	Delta [ppm]	Delta [mmu]	RDB	Composition
131.07102	1709345	11.4	131.07140	-2.9	-0.4	6.5	$C_9 H_7 {}^2H_1 N_1$
132.07754	4374824	29.1	132.07768	-1.1	-0.1	6.5	$C_9 H_6 {}^2H_2 N_1$
133.08415	5101913	33.9	133.08396	1.4	0.2	6.5	$C_9 H_5 {}^2H_3 N_1$
134.09059	3217706	21.4	134.09023	2.6	0.4	6.5	$C_9 H_4 {}^2H_4 N_1$

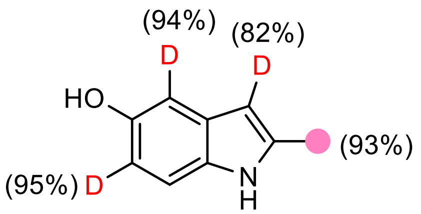

59b

According to GP, Fe-Cellulose-1000 (62 mg, 0.05 mol), substrate (38 mg, 0.26 mmol), D_2O (1.5 mL), H_2 (20 bar), room temperature to 120 °C and then at 120 °C for 24 h. The product **59b** (38 mg, 0.26 mmol, >99%) was obtained.

^{1}H NMR (400 MHz, DMSO-*d*$_6$) δ 10.50 (s, 1H), 8.62 (s, 1H), 6.76 (s, 0.06H), 6.54 (m, 0.05H), 5.95 (m, 0.18H), 2.29 (s, 0.20H), 7.07 (s, 1H).

^{13}C NMR (101 MHz, DMSO-*d*$_6$) δ 150.67, 150.62, 136.25, 136.16, 131.04, 129.79, 129.71, 111.13, 111.03, 110.23, 109.97, 109.74, 103.74, 103.49, 103.26, 98.93, 98.70, 98.45, 13.32, 13.09, 12.90.

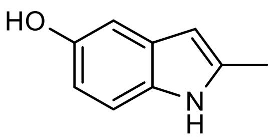

Chemical Formula: C_9H_9NO
Exact Mass: 147.0684

HRMS (ESI-TOF) of 59a:

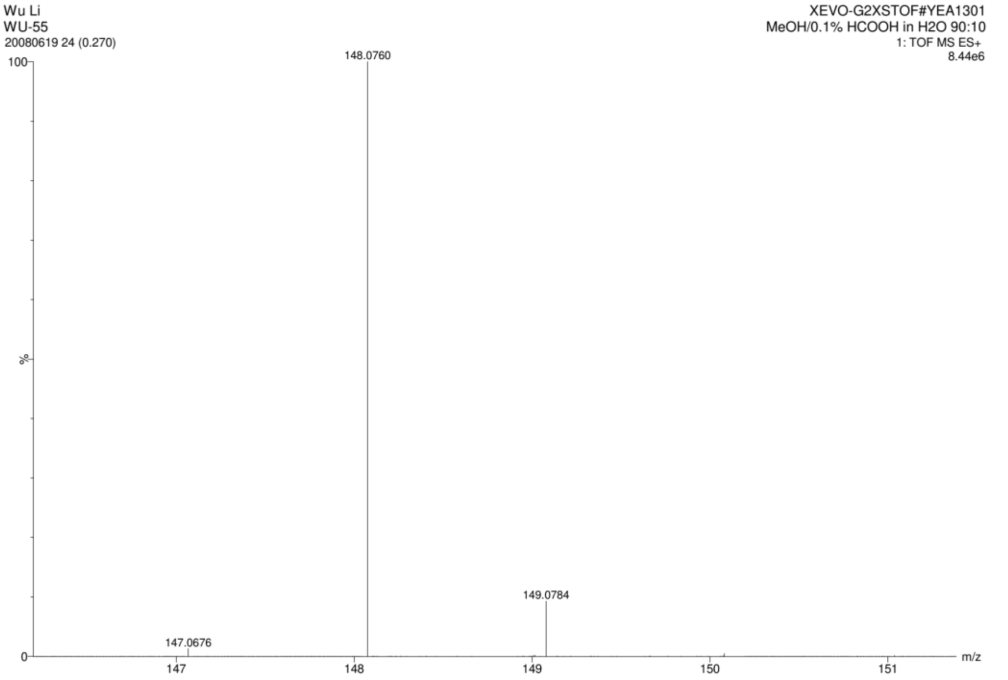

HRMS (ESI-TOF) of 59b [M+H]$^+$:

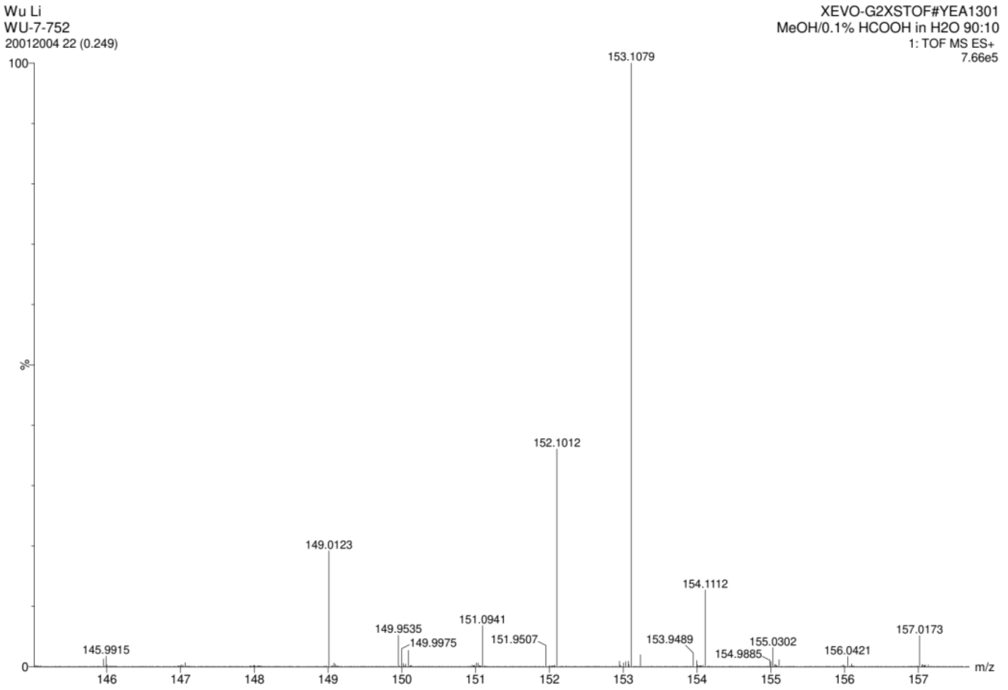

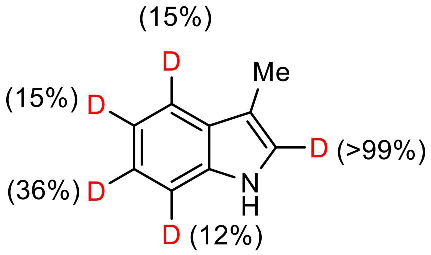

60b

According to GP, Fe-Cellulose-1000 (61 mg, 0.05 mol), substrate (37 mg, 0.28 mmol), D_2O (1.5 mL), H_2 (20 bar), room temperature to 120 °C and then at 120 °C for 24 h. The product **60b** (37 mg, 0.28 mmol, 96%) was obtained.

^{1}H NMR (400 MHz, DMSO-d_6) δ 10.75 (s, 1H), 7.62 – 7.45 (m, 0.85H), 7.43 – 7.34 (m, 0.88H), 7.21 – 7.07 (m, 0.85H), 7.07 – 6.98 (m, 0.64H), 2.30 (s, 3H).

^{13}C NMR (101 MHz, DMSO-d_6) δ 136.72, 136.56, 128.44, 128.40, 123.15, 122.88, 122.72, 122.61, 121.32, 121.22, 118.69, 118.58, 118.47, 118.31, 118.07, 111.72, 111.67, 111.61, 111.56, 109.55, 109.52, 10.01.

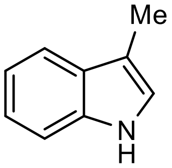

Chemical Formula: C_9H_9N
Exact Mass: 131.0735

HRMS (ESI-TOF) of 60a:

Wu 56 HR (EI)

File : D:\Xcalibur\data\2008\20083112hrei-av2.RAW
Full ms [115.500 - 147.500] - Range: 130.000 - 130.500
Scan No. 1 of 1

Mass	Absolute Intensity	Relative Intensity	Theoretical Mass	Delta [ppm]	Delta [mmu]	RDB	Composition
130.06527	556897	9.1	130.06513	1.1	0.1	6.5	$C_9\ H_8\ N_1$

HRMS (EI) of 60b [M]$^+$:

Wu-7-794

File : D:\Xcalibur\data\2001\20012306eihr-av2.RAW
Full ms [114.500 - 146.500] - Range: 131.000 - 146.500
Scan No. 1 of 1

Mass	Absolute Intensity	Relative Intensity	Theoretical Mass	Delta [ppm]	Delta [mmu]	RDB	Composition
131.07101	8118502	59.4	131.07140	-3.0	-0.4	6.5	$C_9\ H_7\ {}^2H_1\ N_1$
132.07767	13655808	100.0	132.07768	-0.1	-0.0	6.5	$C_9\ H_6\ {}^2H_2\ N_1$
133.08408	9971968	73.0	133.08396	0.9	0.1	6.5	$C_9\ H_5\ {}^2H_3\ N_1$
134.09039	3882042	28.4	134.09023	1.2	0.2	6.5	$C_9\ H_4\ {}^2H_4\ N_1$

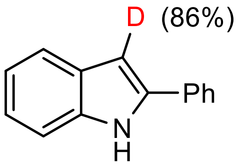

61b

According to GP, Fe-Cellulose-1000 (59 mg, 0.05 mol), substrate (47 mg, 0.24 mmol), D_2O (1.5 mL), H_2 (20 bar), room temperature to 120 °C and then at 120 °C for 24 h. The product **61b** (48 mg, 0.24 mmol, >99%) was obtained.

^1H NMR (300 MHz, DMSO-d_6) δ 11.54 (s, 1H), 7.93 – 7.82 (m, 2H), 7.58 – 7.39 (m, 4H), 7.36 – 7.27 (m, 1H), 7.11 (ddd, J = 8.1, 7.0, 1.2 Hz, 1H), 7.05 – 6.95 (m, 1H), 6.90 (s, 0.14H).

^{13}C NMR (75 MHz, DMSO-d_6) δ 137.54, 137.39, 137.13, 136.97, 132.22, 132.17, 128.89, 128.52, 127.37, 124.97, 121.56, 120.05, 120.02, 119.36, 119.25, 111.30, 111.25, 98.64.

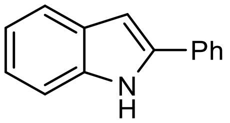

Chemical Formula: $C_{14}H_{11}N$
Exact Mass: 193.0891

HRMS (ESI-TOF) of 61a:

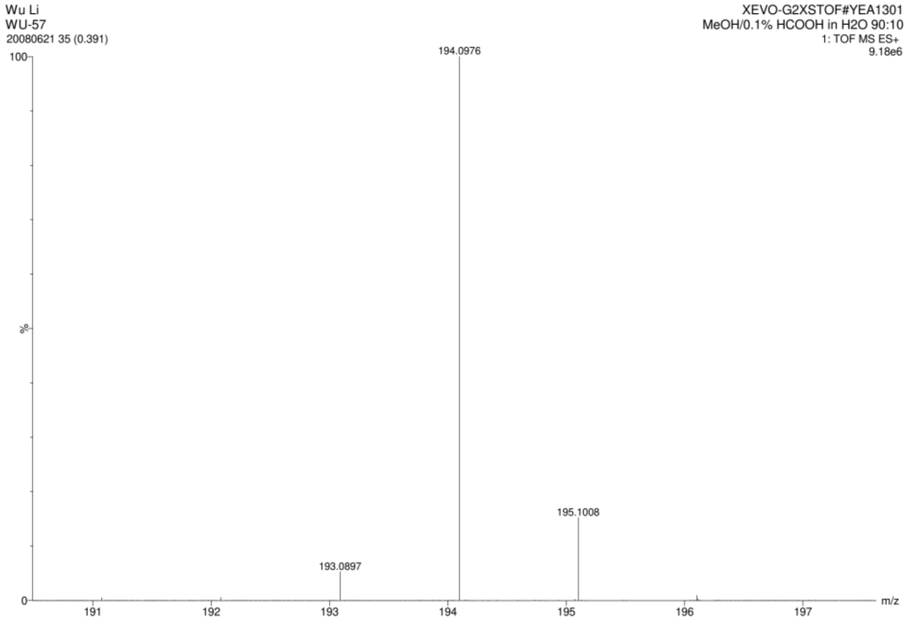

HRMS (ESI-TOF) of 61b [M+H]$^+$:

ESI-TOF Accurate Mass Report | Page 2
File:20040214 | Sample Name:WU-8-127 | UserName:WU Li
Vial:1:C,3 | Date:02-Apr-2020 | Time:16:26:35
Description:MeOH/0.1% HCOOH in H2O 90:10

Sample Report:

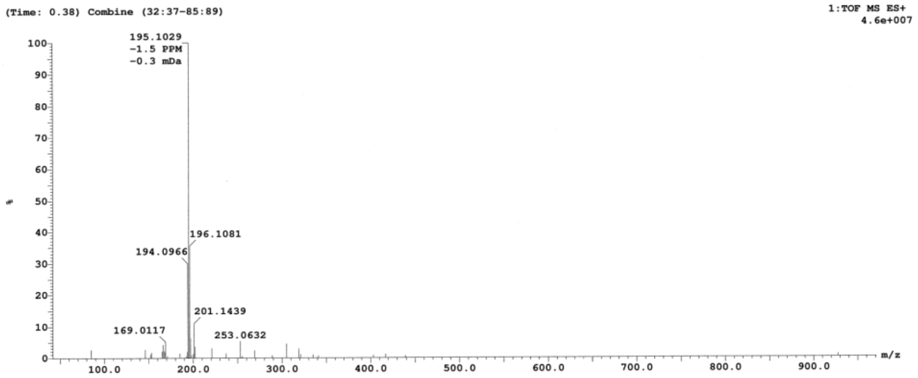

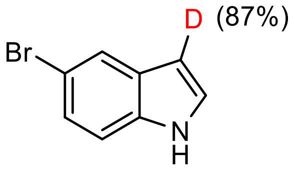

62b

According to GP, Fe-Cellulose-1000 (60 mg, 0.05 mol), substrate (48 mg, 0.24 mmol), D_2O (1.5 mL), H_2 (20 bar), room temperature to 120 °C and then at 120 °C for 24 h. The product **62b** (45 mg, 0.23 mmol, 96%) was obtained.

^{1}H NMR (300 MHz, $CDCl_3$) δ 8.18 (s, 1H), 7.81 (dd, *J* = 1.7, 0.8 Hz, 1H), 7.32 – 7.27 (m, 2H), 7.22 (d, *J* = 2.4 Hz, 1H), 6.45 (m, 0.13H).

^{13}C NMR (75 MHz, $CDCl_3$) δ 134.51, 129.67, 125.41, 125.07, 124.95, 123.30, 113.13, 112.56, 102.35.

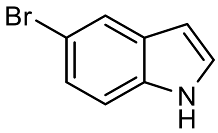

Chemical Formula: C_8H_6BrN
Exact Mass: 194.9684

HRMS (ESI-TOF) of 62a:

Wu-58

File : D:\Xcalibur\data\2008\20083113hrei-av3.RAW
Full ms [177.500 - 209.500] - Range: 177.500 - 209.500
Scan No. 1 of 1

Mass	Absolute Intensity	Relative Intensity	Theoretical Mass	Delta [ppm]	Delta [mmu]	RDB	Composition
194.96837	2342464	100.0	194.96781	2.8	0.6	6.0	$C_8 H_6 N_1 Br_1$
196.96598	2304179	98.4	196.96577	1.1	0.2	6.0	$C_8 H_6 N_1 {}^{81}Br_1$

HRMS (EI) of 62b [M]$^+$:

Wu-7-758

File : D:\Xcalibur\data\2001\20012401eihr-av3.RAW
Full ms [188.500 - 208.500] - Range: 194.000 - 200.000
Scan No. 1 of 1

Mass	Absolute Intensity	Relative Intensity	Theoretical Mass	Delta [ppm]	Delta [mmu]	RDB	Composition
194.96756	4183165	62.5	194.96781	-1.3	-0.3	6.0	$C_8 H_6 N_1 Br_1$
195.97361	6627847	99.0	195.97409	-2.5	-0.5	6.0	$C_8 H_5 {}^2H_1 N_1 Br_1$
196.96945	6520092	97.4					
197.97235	6696025	100.0	197.97204	1.6	0.3	6.0	$C_8 H_5 {}^2H_1 N_1 {}^{81}Br_1$
198.97789	2654037	39.6	198.97832	-2.2	-0.4	6.0	$C_8 H_4 {}^2H_2 N_1 {}^{81}Br_1$
199.98524	1036039	15.5	199.98460	3.2	0.6	6.0	$C_8 H_3 {}^2H_3 N_1 {}^{81}Br_1$

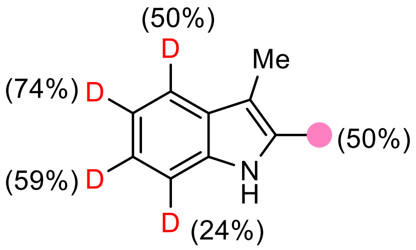

63b

According to GP, Fe-Cellulose-1000 (60 mg, 0.05 mol), substrate (36 mg, 0.25 mmol), D_2O (1.5 mL), H_2 (20 bar), room temperature to 120 °C and then at 120 °C for 24 h. The product **63b** (39 mg, 0.26 mmol, >99%) was obtained.

^{1}H NMR (400 MHz, DMSO-d_6) δ 10.63 (s, 1H), 7.38 – 7.36 (m, 0.50H), 7.26 – 7.23 (m, 0.76H), 6.98 – 6.95 (m, 0.26H), 6.94 – 6.93 (m, 0.41H), 2.43 – 2.27 (m, 1.50H), 2.17 (s, 3H).

^{13}C NMR (101 MHz, DMSO-d_6) δ 135.63, 135.48, 131.66, 131.51, 129.40, 129.37, 129.34, 129.29, 120.33, 120.23, 118.38, 118.27, 118.16, 117.73, 117.62, 110.66, 110.61, 110.55, 110.50, 105.42, 105.39, 105.36, 11.65, 11.60, 11.55, 11.40, 11.35, 11.21, 11.15, 8.79.

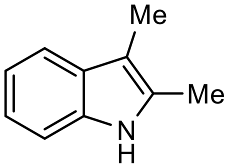

Chemical Formula: $C_{10}H_{11}N$
Exact Mass: 145.0891

HRMS (ESI-TOF) of 63a:

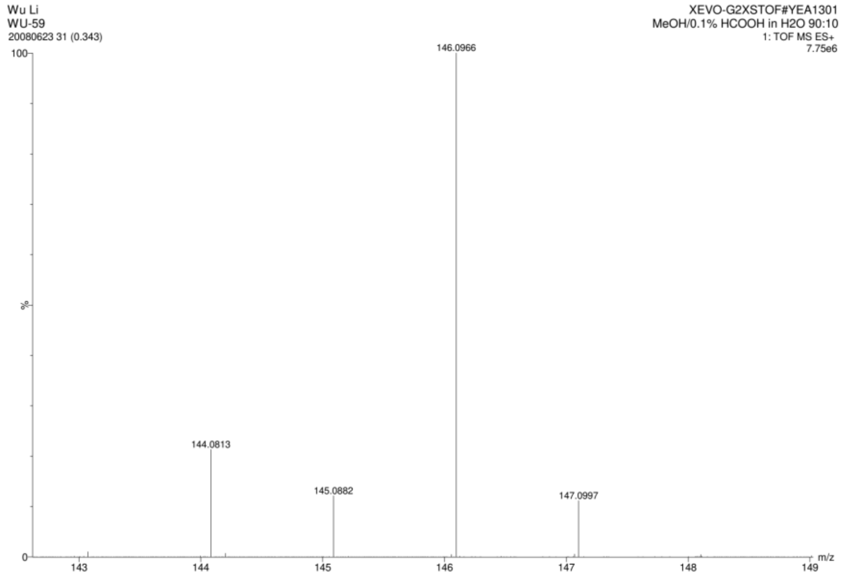

HRMS (EI) of 63b [M]$^+$:

WU-7-762 HR (EI)

File : D:\Xcalibur\data\2001\20012403eihr-av2.RAW
Full ms [138.500 - 158.500] - Range: 138.500 - 150.000
Scan No. 1 of 1

Mass	Absolute Intensity	Relative Intensity	Theoretical Mass	Delta [ppm]	Delta [mmu]	RDB	Composition
146.09287	12742912	23.5	146.09333	-3.1	-0.5	6.5	$C_{10}H_8{}^2H_2N_1$
147.09948	26590976	49.0	147.09961	-0.8	-0.1	6.5	$C_{10}H_7{}^2H_3N_1$
148.10589	44964864	82.9	148.10588	0.1	0.0	6.5	$C_{10}H_6{}^2H_4N_1$
149.11250	54251008	100.0	149.11216	2.3	0.3	6.5	$C_{10}H_5{}^2H_5N_1$

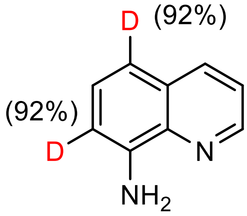

64b

According to GP, Fe-Cellulose-1000 (60 mg, 0.05 mol), substrate (41 mg, 0.28 mmol), D_2O (1.5 mL), H_2 (20 bar), room temperature to 140 °C and then at 140 °C for 24 h. The product **64b** (38 mg, 0.26 mmol, 93%) was obtained.

^{1}H NMR (300 MHz, DMSO-d_6) δ 8.73 (dd, J = 4.2, 1.8 Hz, 1H), 8.18 (dd, J = 8.3, 1.7 Hz, 1H), 7.45 (dd, J = 8.3, 4.1 Hz, 1H), 7.30 (s, 1H), 7.08 –7.05 (m, 0,08 H), 6.89 –6.87 (m, 0,08 H), 5.92 (s, 2H), 4.30 (s, 2H).

^{13}C NMR (75 MHz, DMSO-d_6) δ 147.43, 145.58, 136.26, 134.91, 127.82, 121.88. 113.64, 106.59.

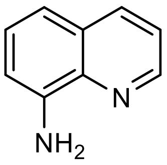

Chemical Formula: $C_9H_8N_2$
Exact Mass: 144.0687

HRMS (ESI-TOF) of 64a:

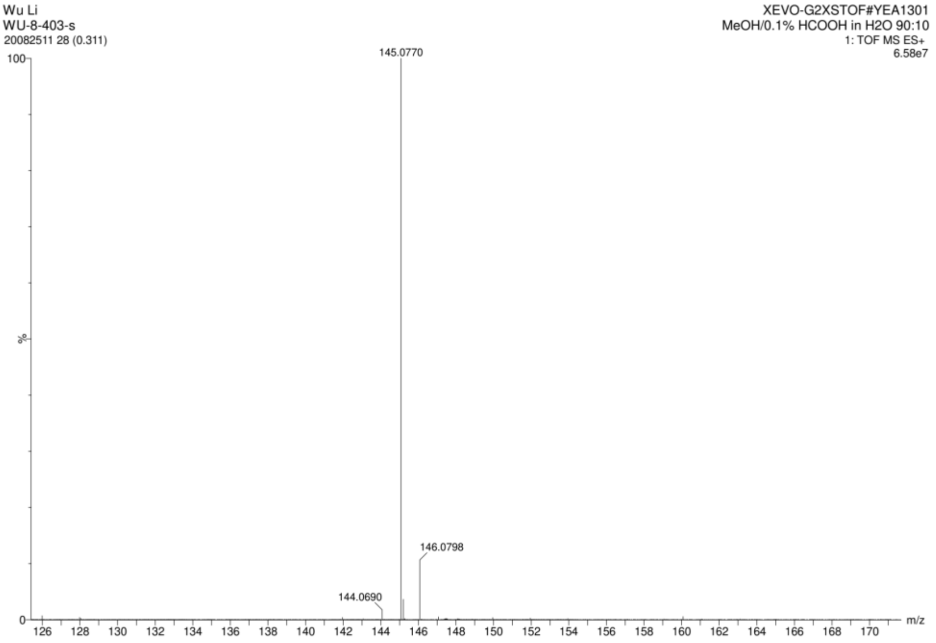

HRMS (ESI-TOF) of 64b $[M+H]^+$:

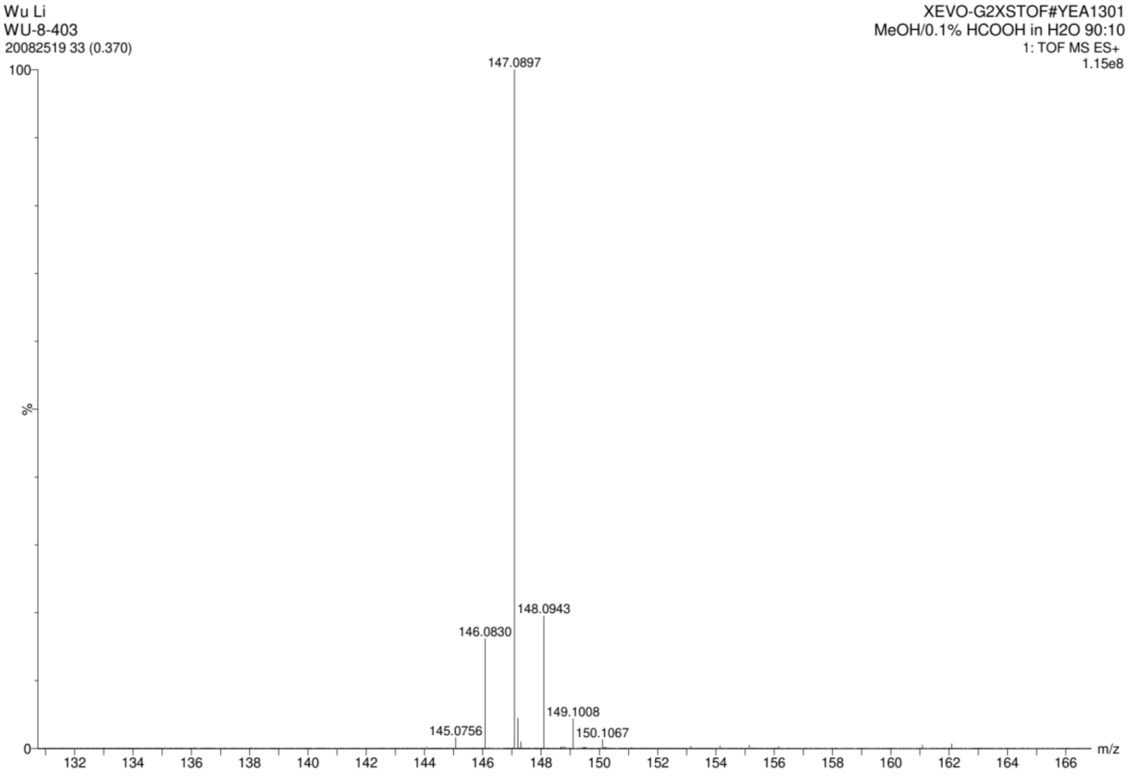

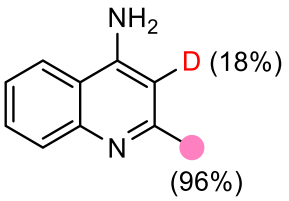

65b

According to GP, Fe-Cellulose-1000 (60 mg, 0.05 mol), substrate (39 mg, 0.25 mmol), D_2O (1.5 mL), H_2 (20 bar), room temperature to 140 °C and then at 140 °C for 24 h. The product **65b** (39 mg, 0.24 mmol, 96%) was obtained.

^1H NMR (300 MHz, DMSO-d_6) δ 8.23 – 8.00 (m, 1H), 7.81 – 7.61 (m, 1H), 7.54 (ddd, J = 8.3, 6.7, 1.4 Hz, 1H), 7.31 (ddd, J = 8.2, 6.7, 1.3 Hz, 1H), 6.64 (s, 2H), 6.44 (s, 0.82H).

^{13}C NMR (75 MHz, DMSO-d_6) δ 158.69, 151.94, 151.88, 148.97, 129.24, 128.61, 123.20, 122.54, 117.75, 117.71, 102.49.

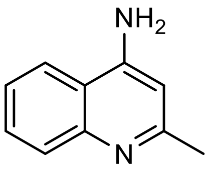

Chemical Formula: $C_{10}H_{10}N_2$
Exact Mass: 158.0844

HRMS (ESI-TOF) of 65a:

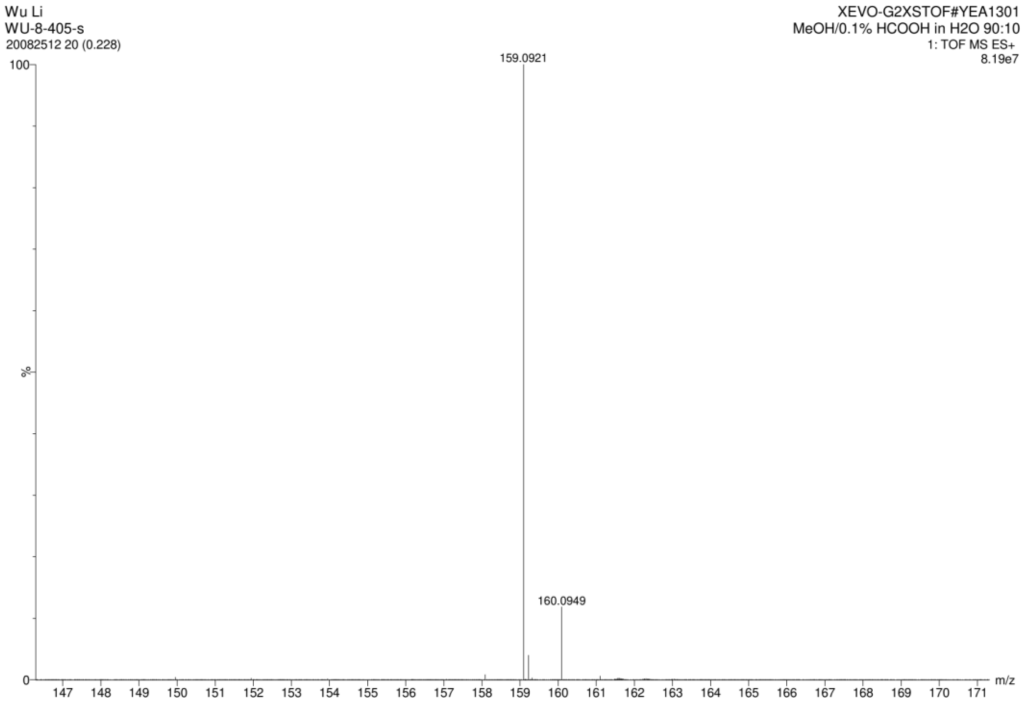

HRMS (ESI-TOF) of 65b [M+H]⁺:

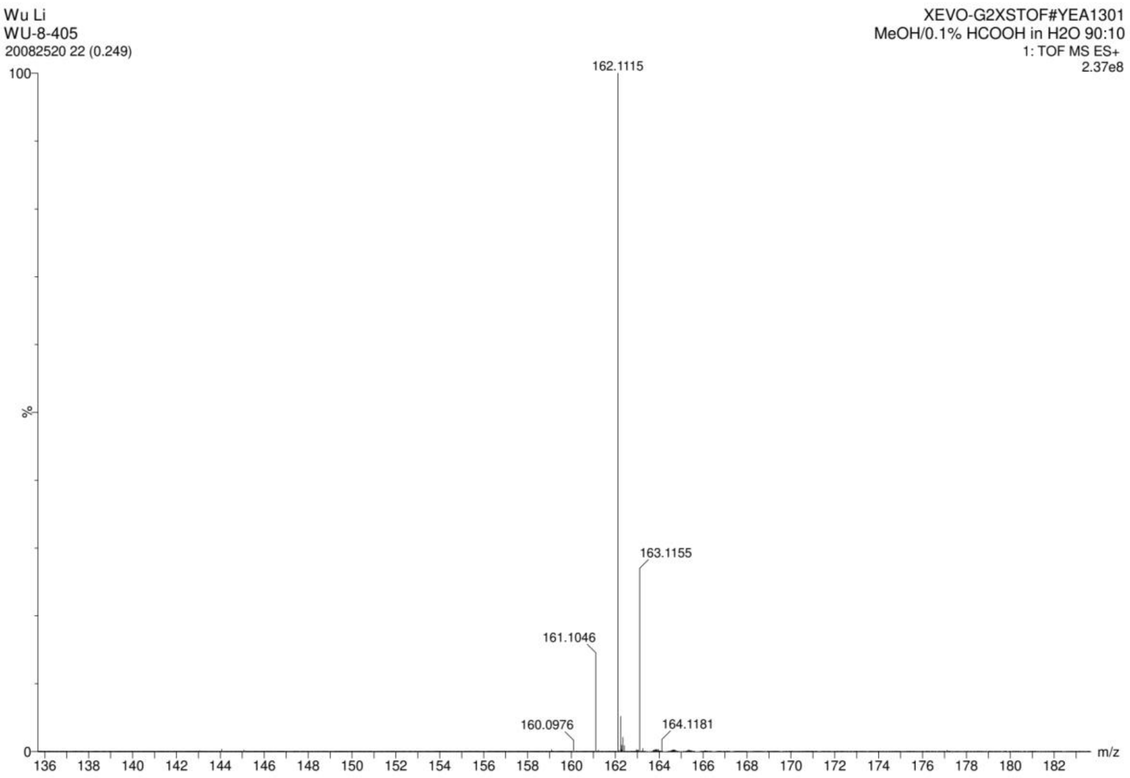

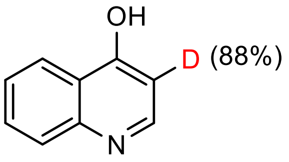

66b

According to GP, Fe-Cellulose-1000 (60 mg, 0.05 mol), substrate (30 mg, 0.21 mmol), D_2O (1.5 mL), H_2 (20 bar), room temperature to 140 °C and then at 140 °C for 24 h. The product **66b** (28 mg, 0.19 mmol, 92%) was obtained.

^{1}H NMR (300 MHz, DMSO-d_6) δ 11.76 (s, 1H), 8.09 (dd, J = 8.1, 1.5 Hz, 1H), 7.90 (s, 1H), 7.64 (ddd, J = 8.4, 6.8, 1.6 Hz, 1H), 7.59 – 7.49 (m, 1H), 7.31 (ddd, J = 8.2, 6.7, 1.3 Hz, 1H).

^{13}C NMR (75 MHz, DMSO-d_6) δ 177.36, 140.48, 139.78, 132.10, 125.40, 123.53, 118.72. 108.92

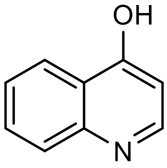

Chemical Formula: C_9H_7NO
Exact Mass: 145.0528

HRMS (ESI-TOF) of 66a unlabelled:

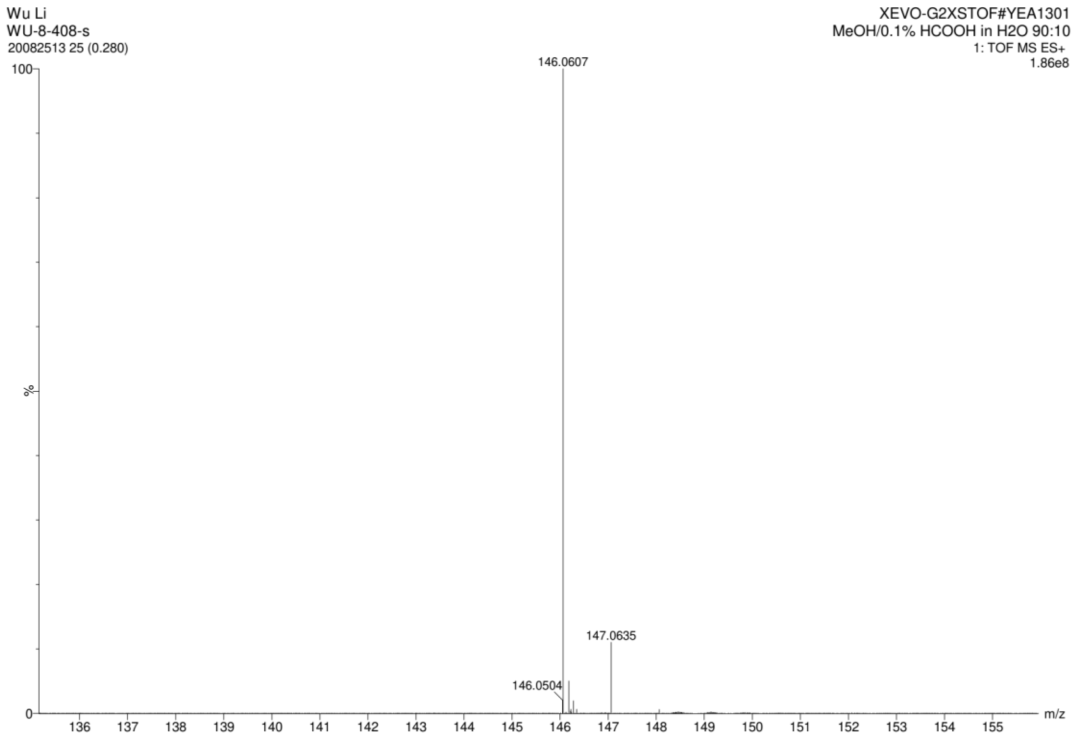

HRMS (ESI-TOF) of 66b $[M+H]^+$:

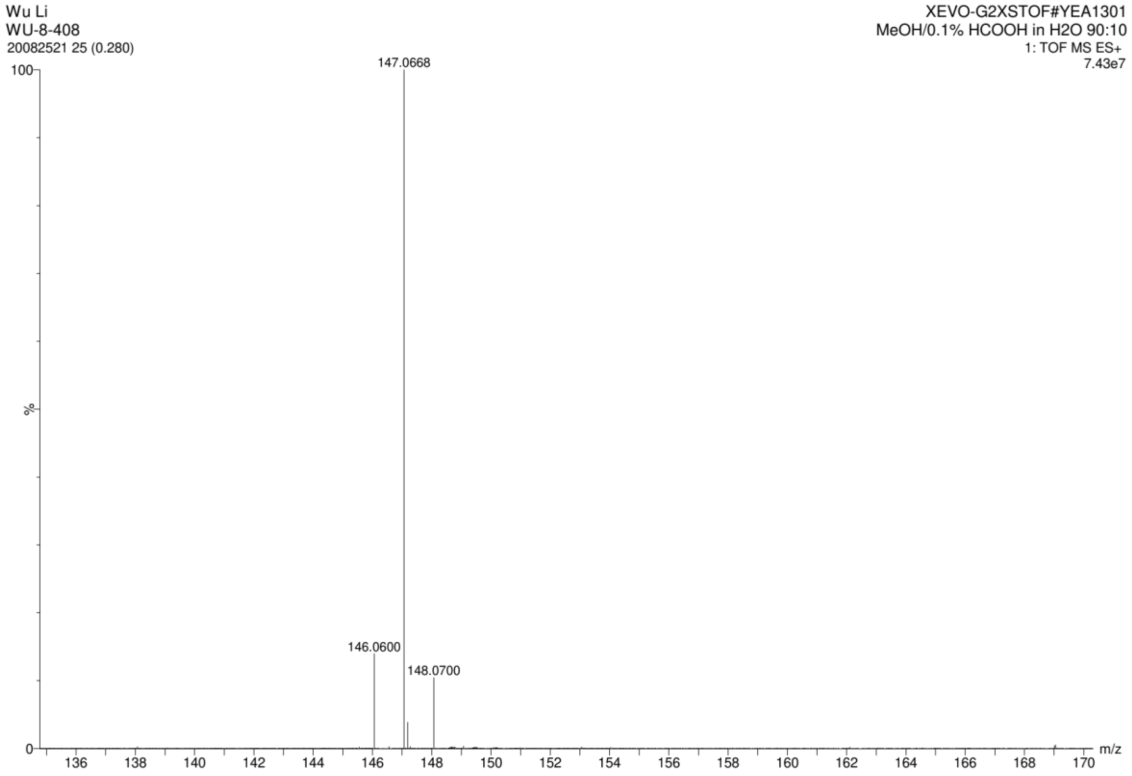

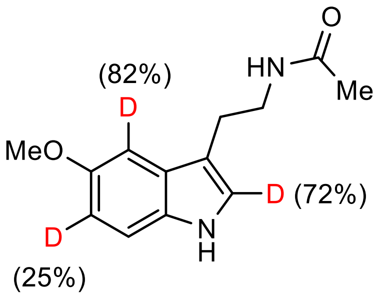

67b
[D] Melatonin

According to GP, Fe-Cellulose-1000 (60 mg, 0.05 mol), substrate (56 mg, 0.24 mmol), D_2O (1.5 mL), H_2 (20 bar), room temperature to 120 °C and then at 120 °C for 24 h. The product **67b** (56 mg, 0.24 mmol, >99%) was obtained.

^{1}H NMR (300 MHz, $CDCl_3$) δ 8.55 – 8.32 (m, 1H), 7.27 – 7.21 (m, 1H), 7.03 (s, 0.18H), 6.98 (s, 0.28H), 6.91 – 6.81 (m, 0.75H), 5.75 (s, 1H), 3.84 (s, 3H), 3.56 (td, J = 6.7, 2.1 Hz, 2H), 2.92 (t, J = 6.8 Hz, 2H), 1.91 (s, 3H).

^{13}C NMR (75 MHz, $CDCl_3$) δ 170.39, 170.30, 154.00, 131.66, 131.52, 127.73, 123.00, 122.84, 112.38, 112.17, 112.12, 100.54, 77.36, 56.03, 39.90, 39.78, 25.34, 23.44, 23.39.

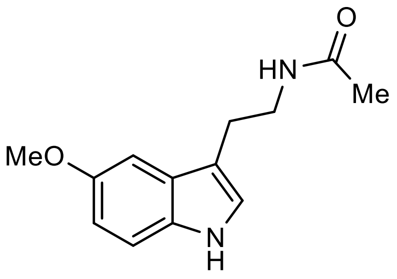

Melatonin

Chemical Formula: $C_{13}H_{16}N_2O_2$
Exact Mass: 232.1212

HRMS (ESI-TOF) of 67a:

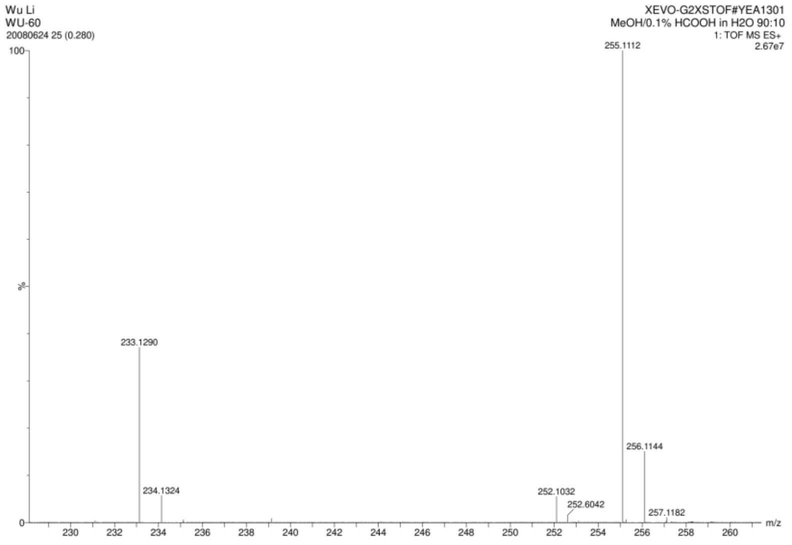

HRMS (ESI-TOF) of 67b $[M+Na]^+$:

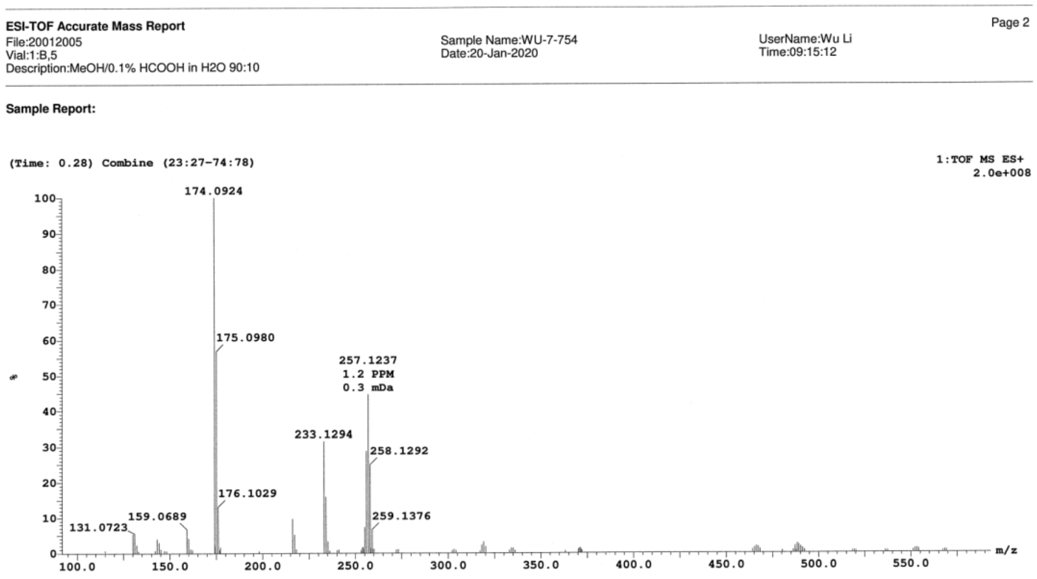

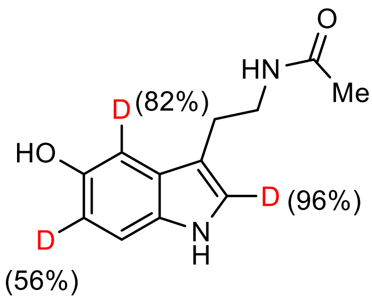

68b

[D] *N*-Acetylserotonin

According to GP, Fe-Cellulose-1000 (62 mg, 0.05 mol), substrate (53 mg, 0.24 mmol), D_2O (1.5 mL), H_2 (20 bar), room temperature to 120 °C and then at 120 °C for 24 h. The product **68b** (53 mg, 0.24 mmol, >99%) was obtained.

^{1}H NMR (300 MHz, DMSO-d_6) δ 10.36 (s, 1H), 8.93 (s, 1H), 8.02 (t, *J* = 5.6 Hz, 1H), 7.15 (t, *J* = 4.3 Hz, 1H), 7.03 (s, 0.18H), 6.83 (s, 0.04H), 6.62 – 6.59 (m, 0.44H), 3.28 (td, *J* = 7.5, 5.6 Hz, 2H), 2.83 – 2.61 (m, 2H), 1.82 (s, 3H).

^{13}C NMR (75 MHz, DMSO-d_6) δ 171.30, 150.49, 150.45, 131.53, 128.51, 123.95, 112.60, 112.50, 112.02, 111.66, 111.50, 40.31, 25.62, 23.18.

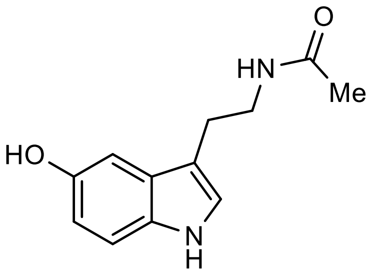

N-Acetylserotonin

Chemical Formula: $C_{12}H_{14}N_2O_2$
Exact Mass: 218.1055

HRMS (ESI-TOF) of 68a:

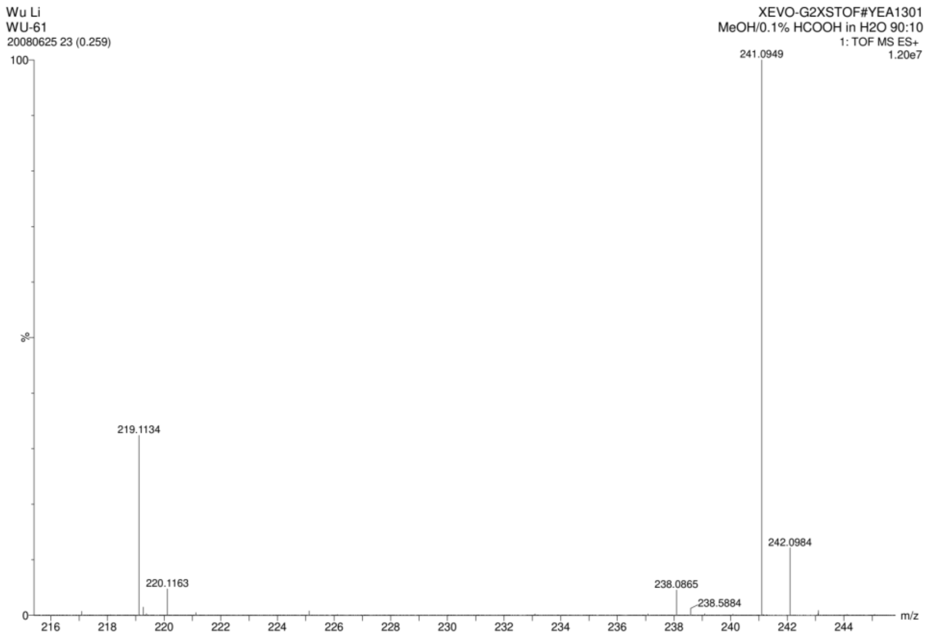

HRMS (ESI-TOF) of 68b [M+Na]$^+$:

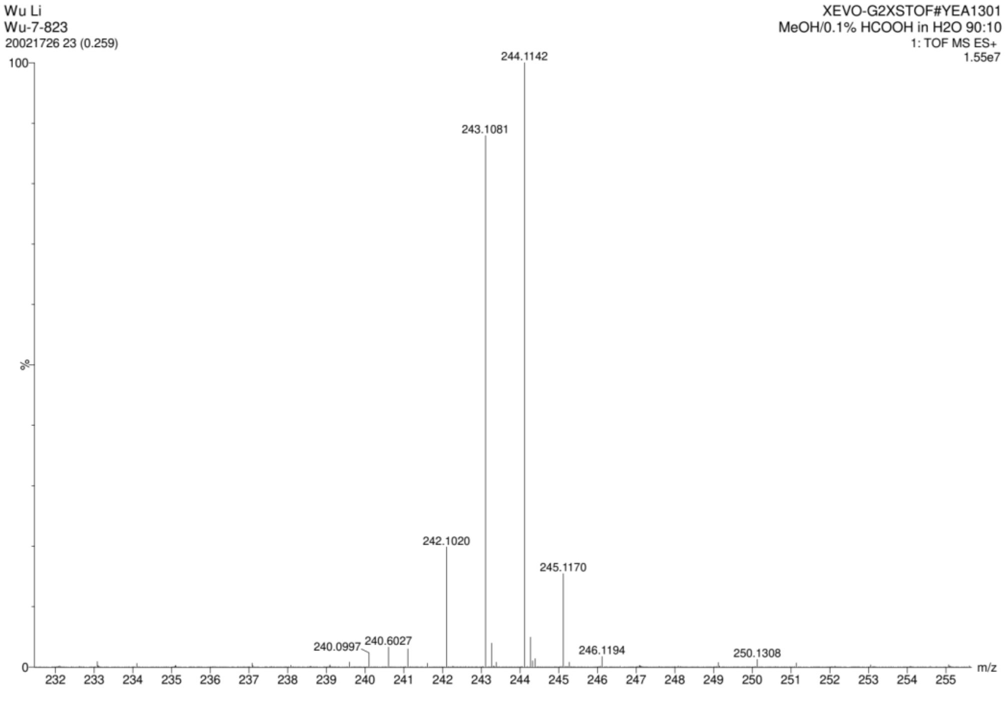

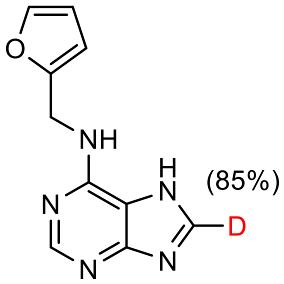

69b
[D] Kinetin

According to GP, Fe-Cellulose-1000 (60 mg, 0.05 mol), substrate (60 mg, 0.28 mmol), D_2O (1.5 mL), H_2 (20 bar), room temperature to 120 °C and then at 120 °C for 24 h. The product **69b** (56 mg, 0.26 mmol, 93%) was obtained.

^{1}H NMR (300 MHz, DMSO-d_6) δ 12.90 (s, 1H), 8.22 (s, 1H), 8.13 (s, 0.15H), 8.02 (1H), 7.64 – 7.42 (m, 1H), 6.37 (t, J = 2.5 Hz, 1H), 6.25 (d, J = 3.3 Hz, 1H), 4.71 (s, 2H).

^{13}C NMR (101 MHz, DMSO-d_6) δ 153.52, 152.69, 142.30, 138.91, 137.96, 110.91, 107.13.

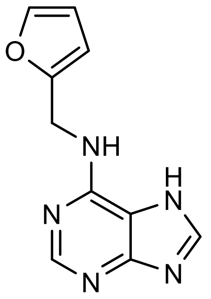

Chemical Formula: $C_{10}H_9N_5O$
Exact Mass: 215.0807

Kinetin

HRMS (ESI-TOF) of 69a:

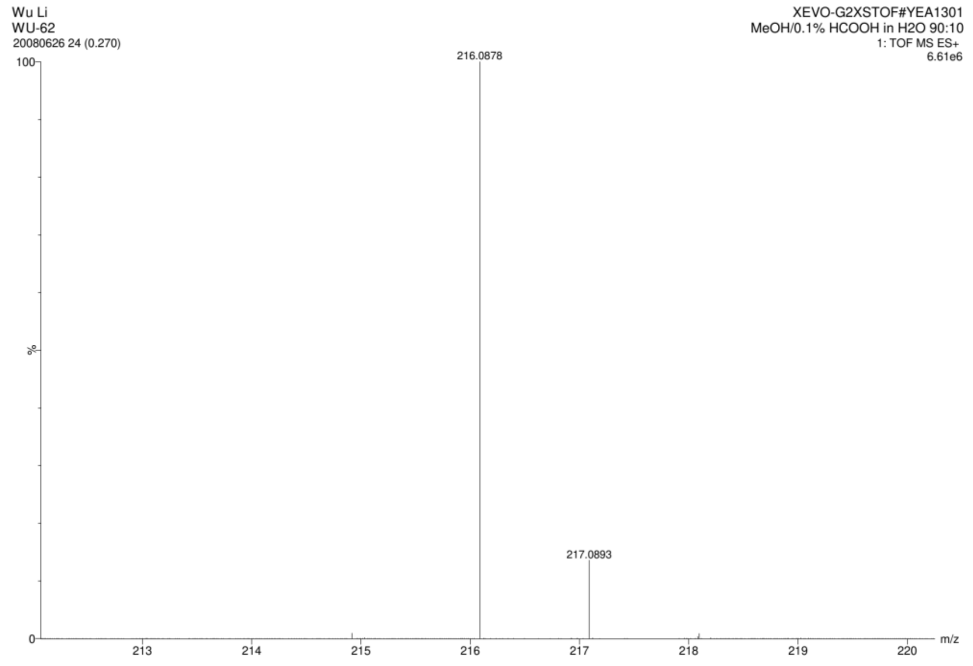

HRMS (ESI-TOF) of 69b [M+H]+:

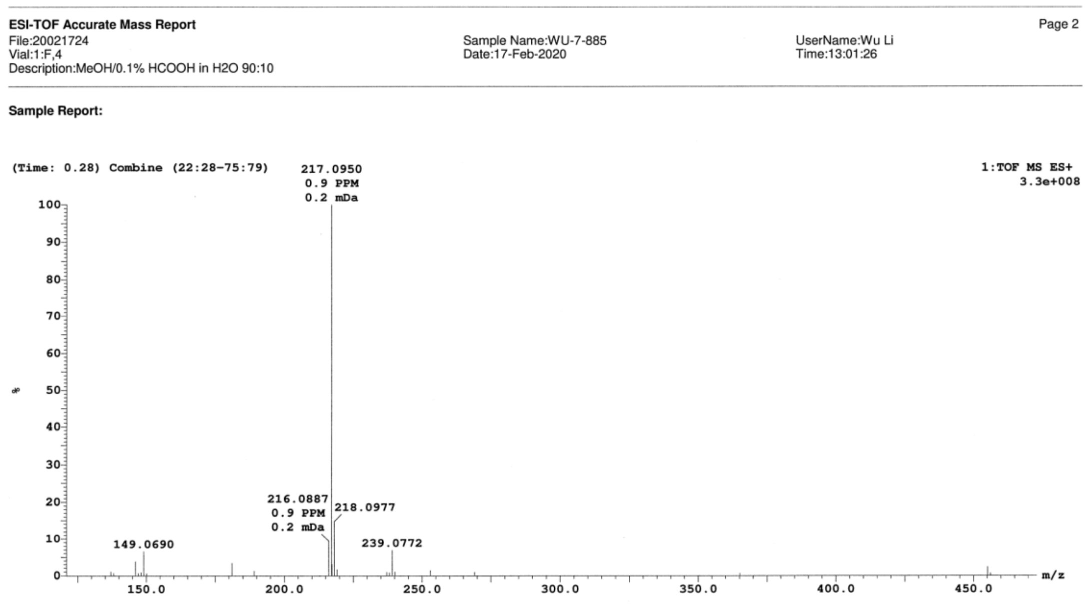

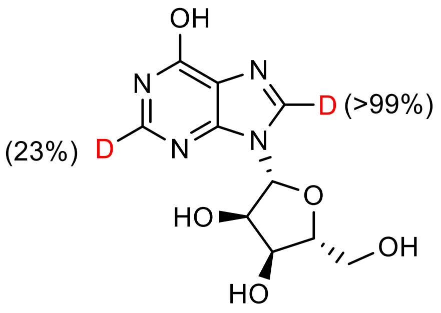

70b
[D] Inosine

According to GP, Fe-Cellulose-1000 (60 mg, 0.05 mol), substrate (68 mg, 0.25 mmol), D_2O (1.5 mL), H_2 (20 bar), room temperature to 120 °C and then at 120 °C for 24 h. The product **70b** (63 mg, 0.23 mmol, 92%) was obtained.

^1H NMR (400 MHz, D_2O) δ 8.13 (s, 0.77H), 6.03 (d, *J* = 5.6 Hz, 1H), 4.70 (t, *J* = 5.4 Hz, 1H), 4.39 (t, *J* = 4.6 Hz, 1H), 4.23 (q, *J* = 3.6 Hz, 1H), 3.94 – 3.65 (m, 2H).

^{13}C NMR (75 MHz, D_2O) δ 158.39, 146.19, 142.99, 109.14, 93.65, 89.38, 85.45, 74.04, 70.85, 70.26, 67.06, 62.80, 61.18.

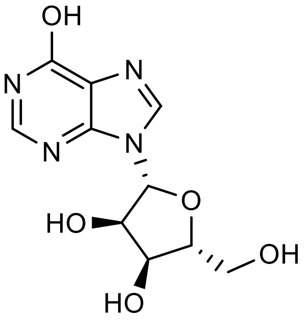

Inosine

Chemical Formula: $C_{10}H_{12}N_4O_5$
Exact Mass: 268.0808

HRMS (EI) of 70b [M]$^+$:

Wu Li 7- 893 HR (EI)

File : D:\Xcalibur\data\2005\20050603ahrei-av3.RAW
Full ms [251.500 - 285.500] - Range: 269.000 - 271.000
Scan No. 1 of 1

Mass	Absolute Intensity	Relative Intensity	Theoretical Mass	Delta [ppm]	Delta [mmu]	RDB	Composition
269.08724	311888	100.0	269.08650	2.8	0.7	7.0	$C_{10} H_{11} {}^2H_1 O_5 N_4$
270.09320	232176	74.4	270.09277	1.6	0.4	7.0	$C_{10} H_{10} {}^2H_2 O_5 N_4$

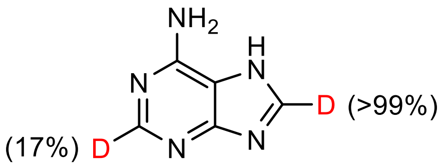

71b
[D] Adenine

According to GP, Fe-Cellulose-1000 (60 mg, 0.05 mol), substrate (32 mg, 0.24 mmol), D_2O (1.5 mL), H_2 (20 bar), room temperature to 120 °C and then at 120 °C for 24 h. The product **71b** (30 mg, 0.22 mmol, 91%) was obtained.

^{1}H NMR (300 MHz, DMSO-d_6) δ 8.11 (s, 0.83H), 7.08 (s, 2H).

^{13}C NMR (101 MHz, DMSO-d_6) δ 155.78, 152.87, 151.03, 139.51, 119.83.

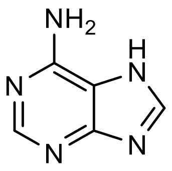

Chemical Formula: $C_5H_5N_5$
Exact Mass: 135.0545

Adenine

HRMS (ESI-TOF) of 71a:

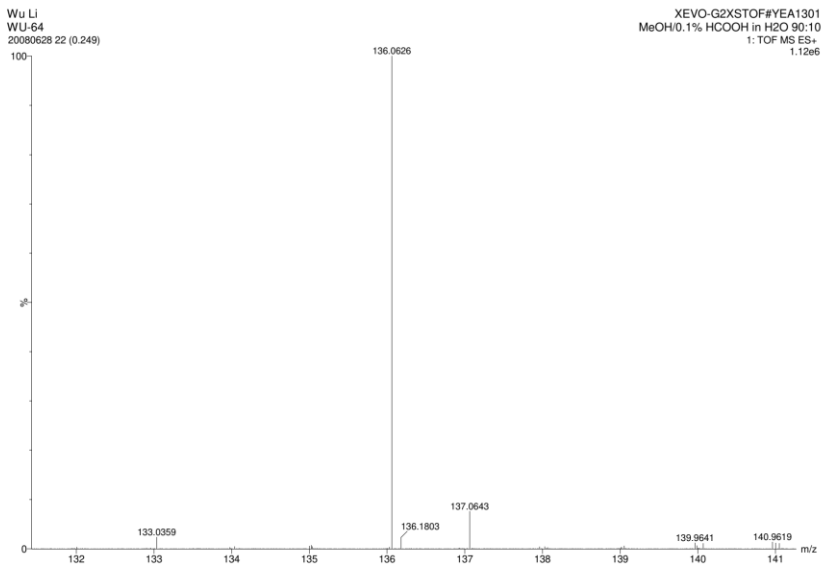

HRMS (ESI-TOF) of 71b [M+H]+:

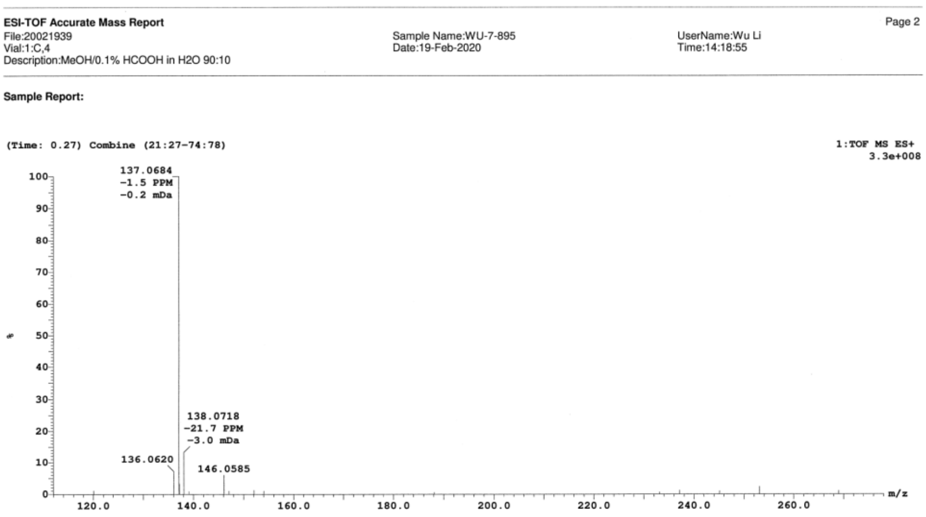

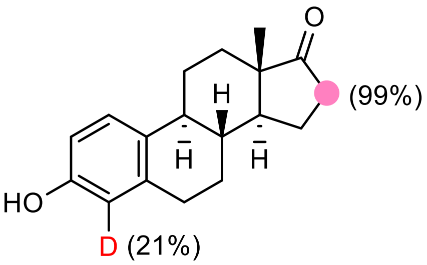

72b
[D] Estrone

According to GP, Fe-Cellulose-1000 (60 mg, 0.05 mol), substrate (71 mg, 0.26 mmol), D_2O (1.5 mL), H_2 (20 bar), room temperature to 120 °C and then at 120 °C for 72 h. The product **72b** (66 mg, 0.24 mmol, 93%) was obtained.

^{1}H NMR (300 MHz, DMSO-d_6) δ 9.19 (s, 1H), 7.04 (d, J = 8.4 Hz, 1H), 6.51 (dd, J = 8.5, 2.6 Hz, 1H), 6.45 (d, J = 2.6 Hz, 0.79H), 2.73 (dd, J = 7.6, 2.9 Hz, 2H), 2.38 (d, J = 7.9 Hz, 1H), 2.35 – 2.23 (m, 1H), 2.18 – 1.82 (m, 3H), 1.80 – 0.98 (m, 6H), 0.80 (s, 3H).

^{13}C NMR (75 MHz, DMSO-d_6) δ 155.23, 137.64, 130.51, 126.53, 115.37, 113.20, 49.93, 47.81, 43.77, 38.37, 35.82, 31.68, 29.41, 26.47, 25.93, 21.50, 13.88.

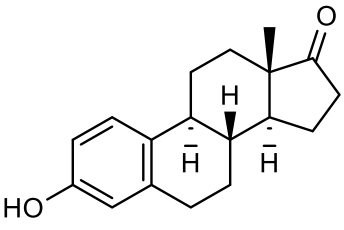

Estrone

Chemical Formula: $C_{18}H_{22}O_2$
Exact Mass: 270.1620

HRMS (ESI-TOF) of 72a unlabelled:

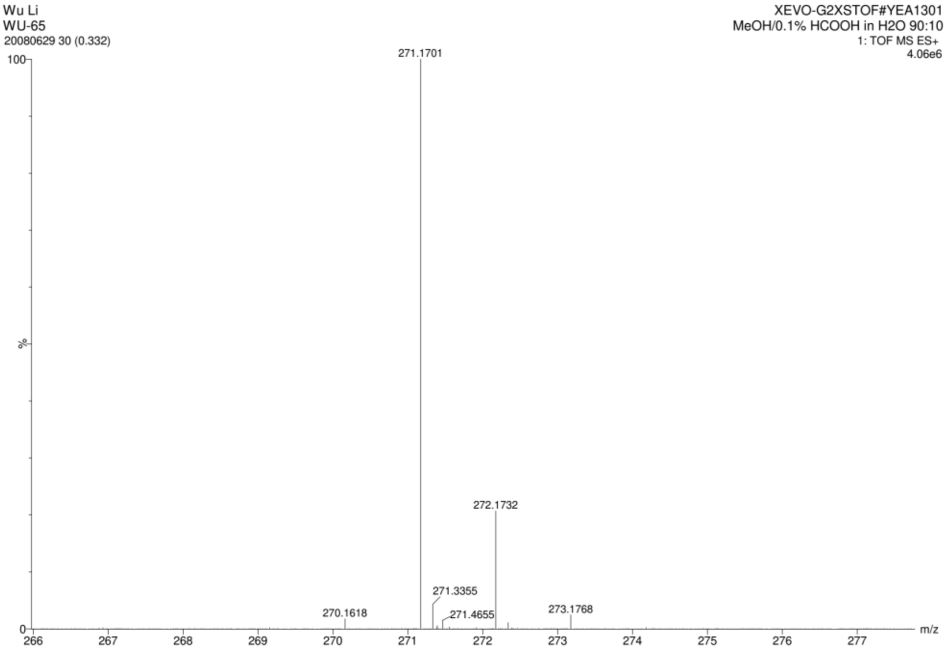

HRMS (ESI-TOF) of 72b [M+H]$^+$:

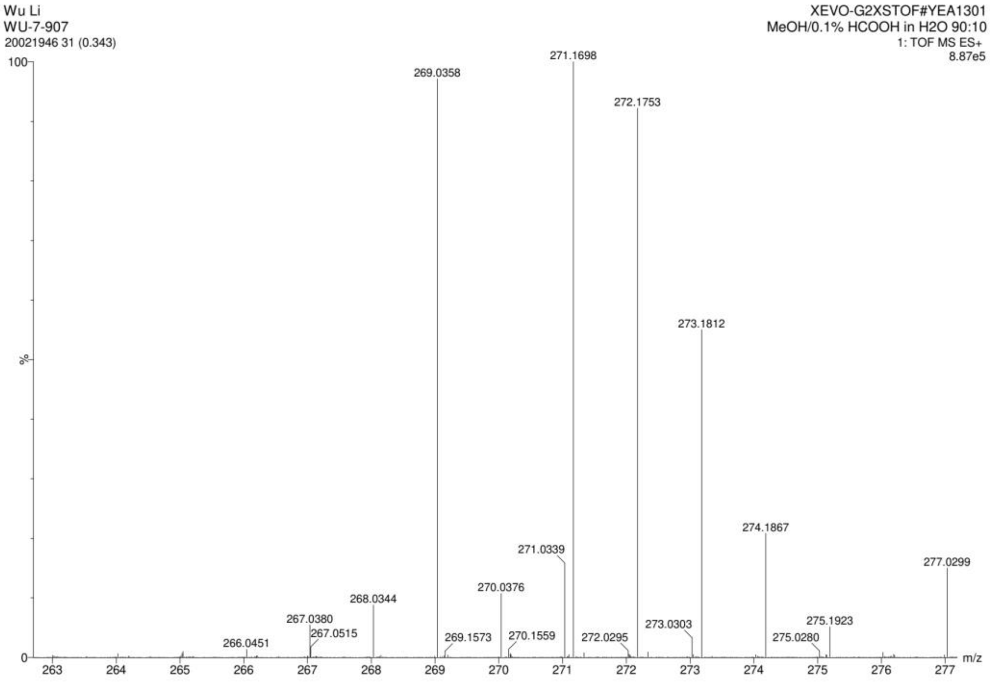

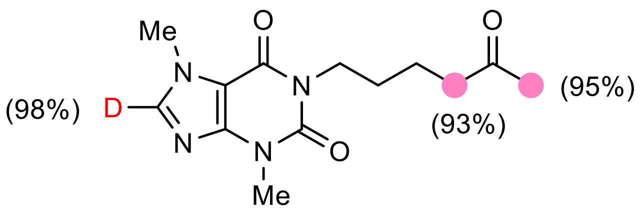

73b

[D] Pentoxifylline

According to GP, Fe-Cellulose-1000 (60 mg, 0.05 mol), substrate (70 mg, 0.25 mmol), D_2O (1.5 mL), H_2 (20 bar), room temperature to 120 °C and then at 120 °C for 24 h. The product **73b** (60 mg, 0.21 mmol, 84%) was obtained.

^{1}H NMR (400 MHz, DMSO-d_6) δ 7.47 (s, 0.02H), 3.93 (s, 5H), 3.50 (s, 3H), 2.46 (m, 0.15H), 2.06 (m, 0.15H), 1.58 (d, J = 5.0 Hz, 4H).

^{13}C NMR (101 MHz, DMSO-d_6) δ 209.06, 155.25, 151.46, 148.75, 141.59, 141.27, 140.96, 107.60, 77.48, 77.36, 77.16, 76.84, 42.83, 42.65, 42.47, 42.28, 40.81, 33.58, 29.69, 27.41, 20.91, 20.86.

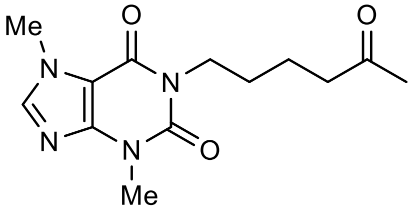

Chemical Formula: $C_{13}H_{18}N_4O_3$
Exact Mass: 278.1379

Pentoxifylline

HRMS (ESI-TOF) of 73a:

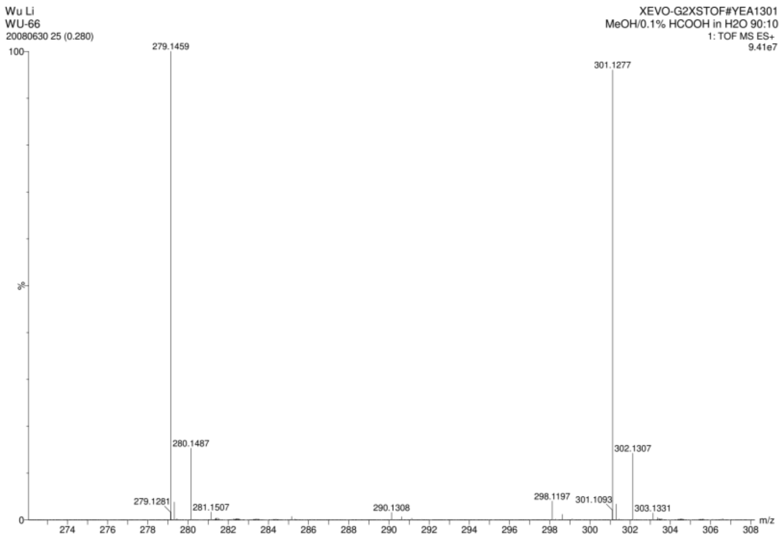

HRMS (ESI-TOF) of 73b $[M+H]^+$:

ESI-TOF Accurate Mass Report

Page 1

Results file: E:\Projects\2005.PRO\SampleDB\2005.rpt
Last modified: Thursday, May 07, 2020 13:35:44

Sample Summary:

Sample	File	Sample Name	User	Target	Formula	Expected Mass	Observed Mass	Error PPM	Error mDa	
121	20050722	WU-8-263	Wu Li	284.1755	C13H12N4O3[2H6]	285.1833	285.1826	-2.5	-0.7	$[M+H]^+$
						307.1647	307.1649	0.7	0.2	$[M+Na]^+$

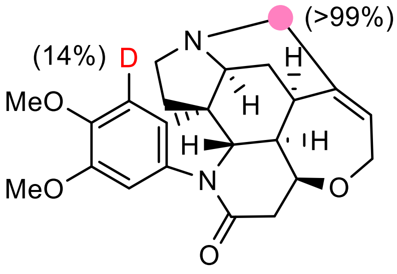

74b
[D] Brucine

According to GP, Fe-Cellulose-1000 (60 mg, 0.05 mol), substrate (82 mg, 0.21 mmol), D_2O (1.5 mL), H_2 (20 bar), room temperature to 120 °C and then at 120 °C for 24 h. The product **74b** (77 mg, 0.19 mmol, 90%) was obtained.

^{1}H NMR (400 MHz, $CDCl_3$) δ 7.74 (s, 0.86H), 6.60 (s, 1H), 5.91 – 5.79 (m, 1H), 4.21 (d, J = 3.4 Hz, 1H), 4.14 – 3.92 (m, 2H), 3.86 – 3.73 (m, 8H), 3.70 – 3.59 (m, 1H), 3.21 – 3.04 (m, 2H), 2.78 (ddd, J = 12.2, 10.1, 6.8 Hz, 1H), 2.66 (d, J = 14.8 Hz, 1H), 2.28 (dt, J = 14.4, 4.4 Hz, 1H), 1.96 – 1.69 (m, 2H), 1.40 (dt, J = 14.5, 2.2 Hz, 1H), 1.28 – 0.99 (m, 1H).

^{13}C NMR (101 MHz, $CDCl_3$) δ 168.96, 149.20, 146.22, 140.17, 135.90, 127.68, 123.25, 105.48, 100.97, 77.61, 77.30, 64.56, 60.32, 59.93, 56.44, 56.19, 52.64, 51.92, 50.16, 48.20, 42.34, 31.48, 26.73.

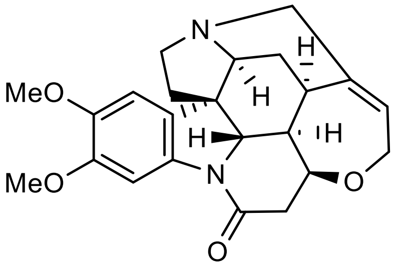

Chemical Formula: $C_{23}H_{26}N_2O_4$
Exact Mass: 394.1893

Brucine

HRMS (ESI-TOF) of 74a:

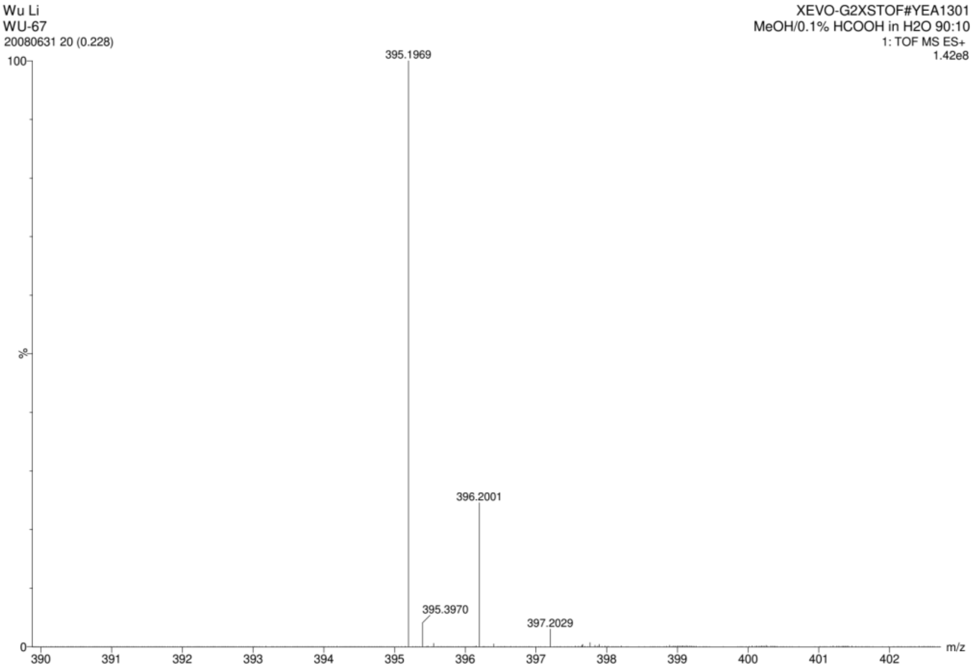

HRMS (ESI-TOF) of 74b $[M+H]^+$:

ESI-TOF Accurate Mass Report

File:20032019
Vial:1:B,2
Description:MeOH/0.1% HCOOH in H2O 90:10

Sample Name:WU-8-70
Date:20-Mar-2020

UserName:Wu Li
Time:15:42:45

Page 2

Sample Report:

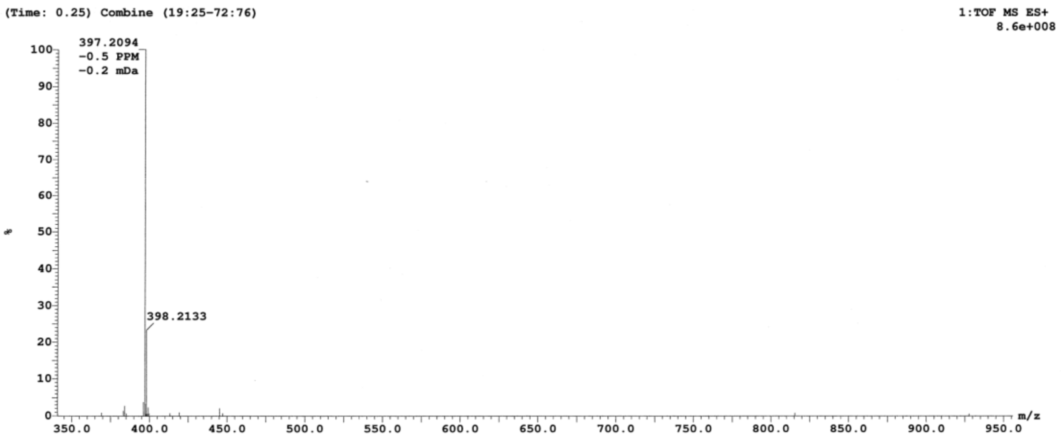

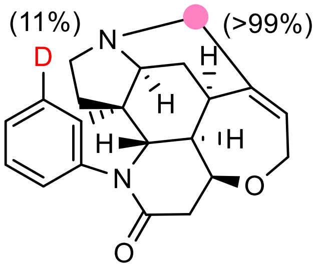

75b
[D] Strychnine

According to GP, Fe-Cellulose-1000 (60 mg, 0.05 mol), substrate (84 mg, 0.25 mmol), D_2O (1.5 mL), H_2 (20 bar), room temperature to 120 °C and then at 120 °C for 24 h. The product **75b** (77 mg, 0.23 mmol, 92%) was obtained.

^1H NMR (300 MHz, $CDCl_3$) δ 8.09 (d, J = 8.0 Hz, 0.89H), 7.24 (d, J = 7.3 Hz, 1H), 7.11 (dt, J = 14.6, 7.3 Hz, 2H), 5.89 (s, 1H), 4.27 (d, J = 3.5 Hz, 1H), 4.10 (qd, J = 13.9, 6.6 Hz, 2H), 3.93 (d, J = 3.6 Hz, 1H), 3.85 (d, J = 10.5 Hz, 1H), 3.70 (d, J = 14.8 Hz, 1H), 3.27 – 3.07 (m, 2H), 2.86 (q, J = 9.6 Hz, 1H), 2.69 (t, J = 16.0 Hz, 1H), 2.35 (dt, J = 14.5, 4.5 Hz, 1H), 2.20 (s, 1H), 2.00 – 1.72 (m, 2H), 1.45 (d, J = 14.3 Hz, 1H), 1.26 (d, J = 9.6 Hz, 1H).

^{13}C NMR (75 MHz, $CDCl_3$) δ 169.48, 142.32, 140.61, 132.85, 128.70, 127.45, 124.35, 122.40, 116.37, 77.36, 64.74, 60.34, 60.24, 52.79, 52.08, 50.48, 48.36, 42.98, 31.72, 26.97.

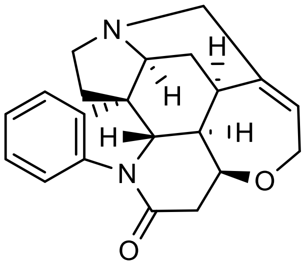

Chemical Formula: $C_{21}H_{22}N_2O_2$
Exact Mass: 334.1681

Strychnine

HRMS (ESI-TOF) of 75a:

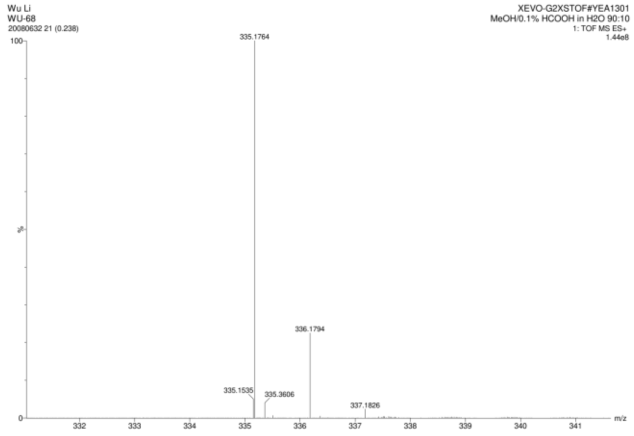

HRMS (ESI-TOF) of 75b $[M+H]^+$:

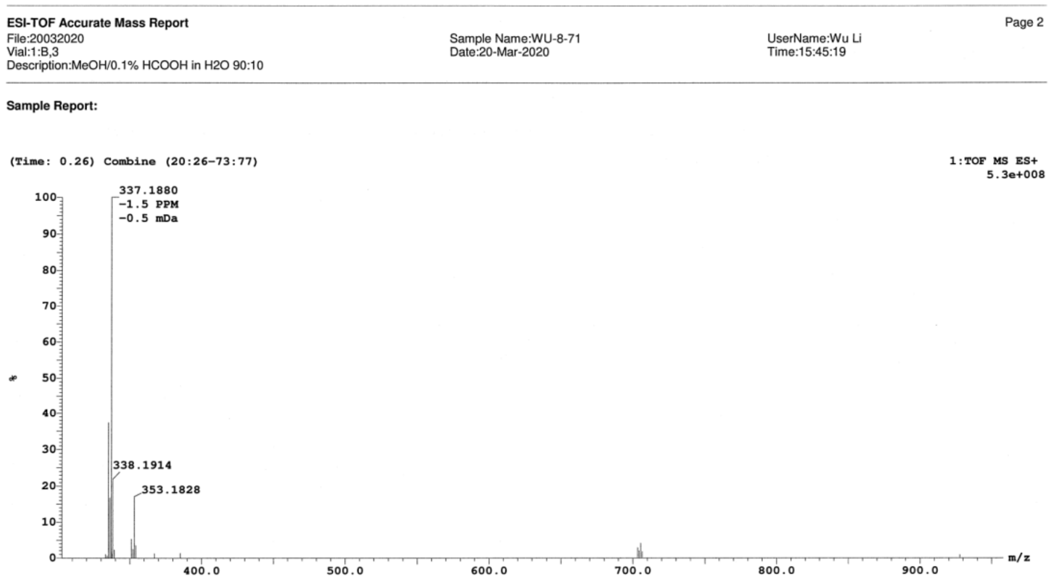

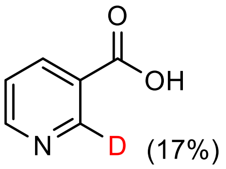

76b
[D] Niacin

According to GP, Fe-Cellulose-1000 (60 mg, 0.05 mol), substrate (34 mg, 0.28 mmol), D_2O (1.5 mL), H_2 (20 bar), room temperature to 120 °C and then at 120 °C for 72 h. The product **76b** (34 mg, 0.27 mmol, 96%) was obtained.

^{1}H NMR (300 MHz, DMSO-d_6) δ 9.06 (s, 0.83H), 8.77 (d, J = 4.6 Hz, 1H), 8.26 (d, J = 7.8 Hz, 1H), 7.53 (t, J = 6.4 Hz, 1H).

^{13}C NMR (101 MHz, DMSO-d_6) δ 166.81, 153.69, 150.71, 137.42, 126.88, 124.27.

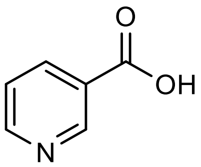

Chemical Formula: $C_6H_5NO_2$
Exact Mass: 123.0320

Niacin

HRMS (ESI-TOF) of 76a:

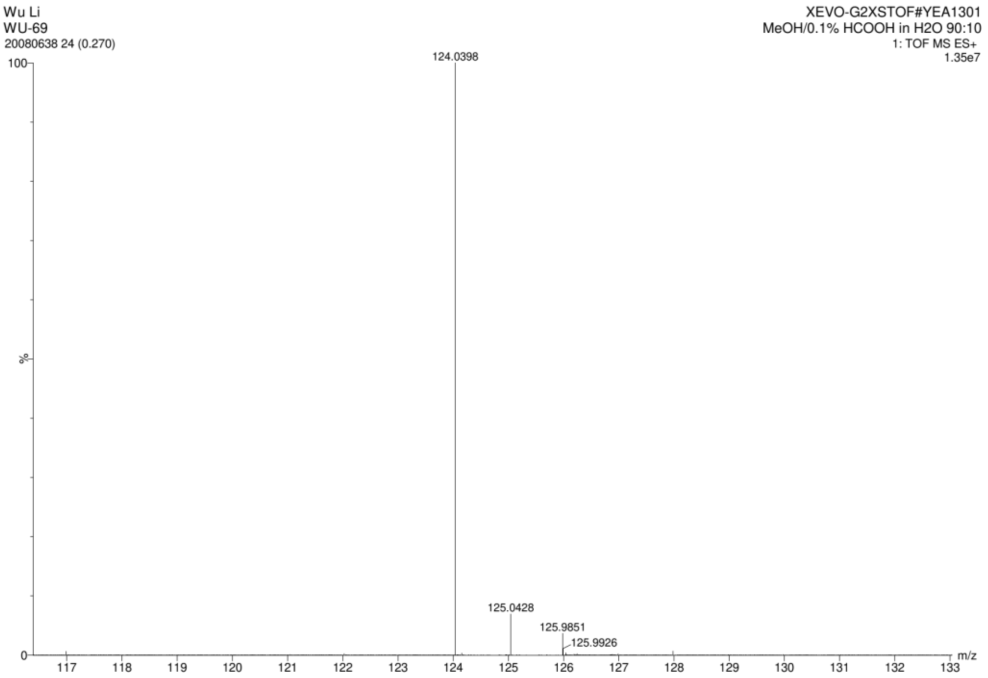

HRMS (ESI-TOF) of 76b [M+H]⁺:

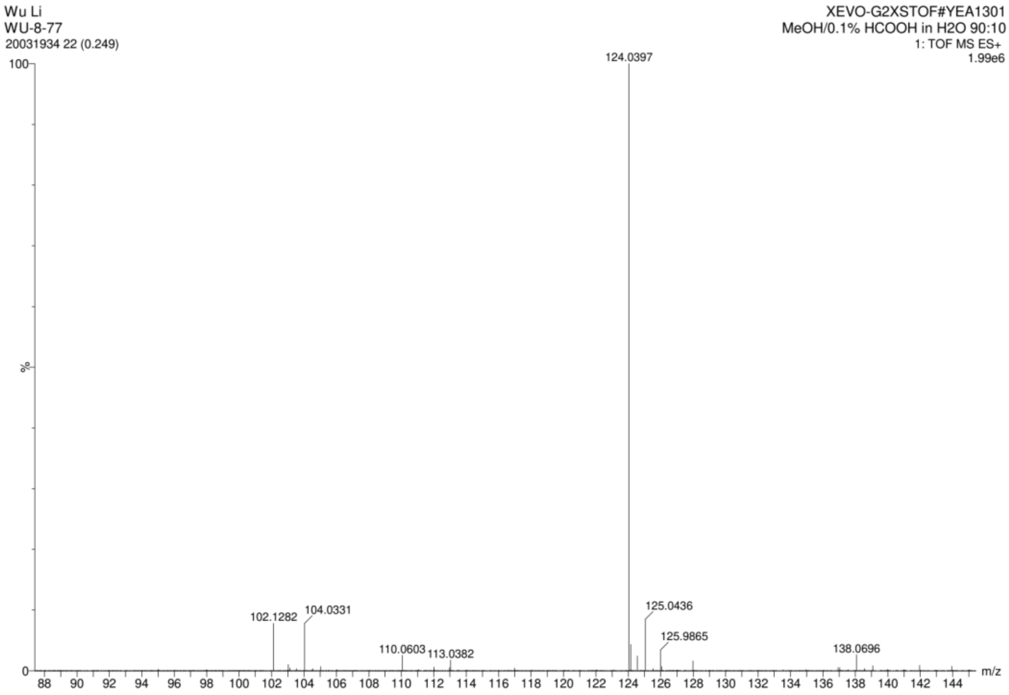

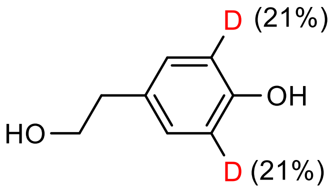

77b
[D] Tyrosol

According to GP, Fe-Cellulose-1000 (61 mg, 0.05 mol), substrate (37 mg, 0.27 mmol), D_2O (1.5 mL), H_2 (20 bar), room temperature to 120 °C and then at 120 °C for 72 h. The product **77b** (37 mg, 0.27 mmol, >99%) was obtained.

^{1}H NMR (300 MHz, DMSO-d_6) δ 9.12 (s, 1H), 7.10 – 6.87 (m, 2H), 6.72 – 6.63 (m, 1.59H), 4.57 (t, J = 5.2 Hz, 1H), 3.53 (td, J = 7.2, 3.7 Hz, 2H), 2.60 (t, J = 7.3 Hz, 2H).

^{13}C NMR (75 MHz, DMSO-d_6) δ 155.91, 155.87, 130.14, 130.04, 129.91, 115.39, 63.07, 38.74.

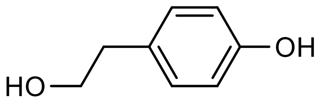

Chemical Formula: $C_8H_{10}O_2$
Exact Mass: 138.0681

Tyrosol

HRMS (EI) of 77b [M]$^+$:

Wu Li 8-6 HR (EI)

File : D:\Xcalibur\data\2005\20051106hrei-av2.RAW
Full ms [115.500 - 147.500] - Range: 115.500 - 147.500
Scan No. 1 of 1

Mass	Absolute Intensity	Relative Intensity	Theoretical Mass	Delta [ppm]	Delta [mmu]	RDB	Composition
138.06788	10048512	14.2	138.06753	2.5	0.3	4.0	$C_8 H_{10} O_2$
138.07461	70602240	100.0					
139.07375	5727182	8.1	139.07381	-0.4	-0.1	4.0	$C_8 H_9 {}^2H_1 O_2$
139.08021	39861248	56.5					
140.08391	8580352	12.2					

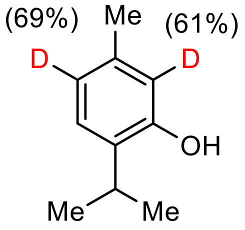

78b
[D] Thymol

According to GP, Fe-Cellulose-1000 (60 mg, 0.05 mol), substrate (39 mg, 0.26 mmol), D_2O (1.5 mL), H_2 (20 bar), room temperature to 120 °C and then at 120 °C for 72 h. The product **78b** (32 mg, 0.21 mmol, 81%) was obtained.

^{1}H NMR (300 MHz, DMSO-d_6) δ 9.06 (s, 1H), 7.12 – 6.89 (m, 1H), 6.58 (s, 0.39H), 6.53 (m, 0.31H), 3.23 – 3.01 (m, 1H), 2.17 (s, 3H), 1.13 (d, J = 6.9 Hz, 6H).

^{13}C NMR (75 MHz, DMSO-d_6) δ 154.61, 154.57, 135.64, 135.55, 131.61, 126.08, 125.96, 120.08, 115.99, 26.46, 23.08, 21.14, 21.08, 21.02.

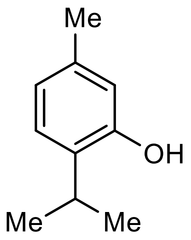

Chemical Formula: $C_{10}H_{14}O$
Exact Mass: 150.1045

Thymol

HRMS (EI) of 78b [M]$^+$:

Wu Li 8-4 HR(EI)

File : D:\Xcalibur\data\2005\20051107hrei-av2.RAW
Full ms [139.500 - 159.500] - Range: 139.500 - 159.500
Scan No. 1 of 1

Mass	Absolute Intensity	Relative Intensity	Theoretical Mass	Delta [ppm]	Delta [mmu]	RDB	Composition
151.11099	1507480	90.9	151.11174	-5.0	-0.8	3.5	$C_{10} H_{15} O_1$
152.11730	1657489	100.0	152.11802	-4.7	-0.7	3.5	$C_{10} H_{14} {}^2H_1 O_1$

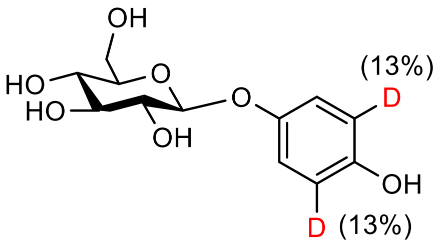

79b
[D] Arbutin

According to GP, Fe-Cellulose-1000 (61 mg, 0.05 mol), substrate (76 mg, 0.28 mmol), D_2O (1.5 mL), H_2 (20 bar), room temperature to 120 °C and then at 120 °C for 72 h. The product **79b** (70 mg, 0.26 mmol, 93%) was obtained.

^{1}H NMR (300 MHz, DMSO-d_6) δ 9.02 (s, 1H), 6.95 – 6.80 (m, 2H), 6.73 – 6.62 (m, 1.75H), 6.55 (s, 1H), 5.26 (d, J = 4.5 Hz, 1H), 5.03 (dd, J = 16.8, 4.8 Hz, 2H), 4.60 (dd, J = 22.5, 6.5 Hz, 2H), 3.76 – 3.61 (m, 1H), 3.30 – 3.07 (m, 4H).

^{13}C NMR (75 MHz, DMSO-d_6) δ 152.67, 150.83, 118.13, 116.12, 115.95, 102.27, 77.40, 77.07, 73.76, 70.26, 61.25.

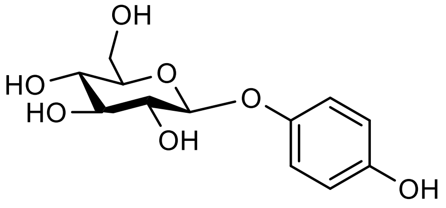

Chemical Formula: $C_{12}H_{16}O_7$
Exact Mass: 272.0896

Arbutin

HRMS (ESI-TOF) of 79b [M+Na]$^+$:

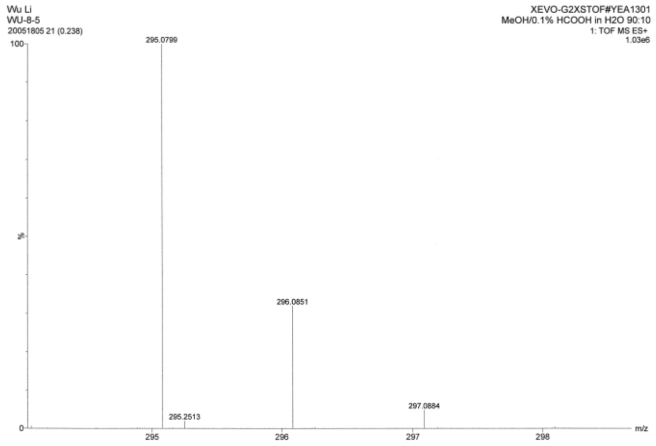

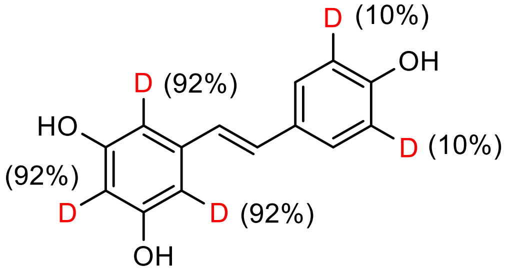

80b
[D] Resveratrol

According to GP, Fe-Cellulose-1000 (60 mg, 0.05 mol), substrate (52 mg, 0.23 mmol), D_2O (1.5 mL), H_2 (20 bar), room temperature to 120 °C and then at 120 °C for 72 h. The product **80b** (51 mg, 0.22 mmol, 96%) was obtained.

^{1}H NMR (300 MHz, DMSO-d_6) δ 9.30 (s, 3H), 7.50 – 7.29 (m, 2H), 6.94 (d, J = 16.4 Hz, 1H), 6.81 (d, J = 16.4 Hz, 1H), 6.78 – 6.70 (m, 1.80H), 6.40 (s, 0.17H), 6.12 (s, 0.08H).

^{13}C NMR (75 MHz, DMSO-d_6) δ 158.88, 158.84, 157.64, 139.57, 129.60, 128.54, 128.35, 128.31, 126.03, 115.98, 115.43, 104.76.

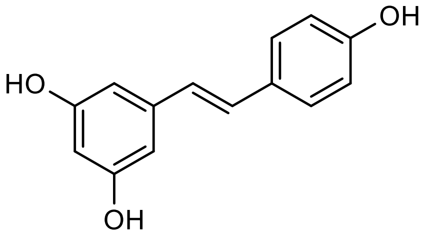

Chemical Formula: $C_{14}H_{12}O_3$
Exact Mass: 228.0786

Resveratrol

HRMS (ESI-TOF) of 80a:

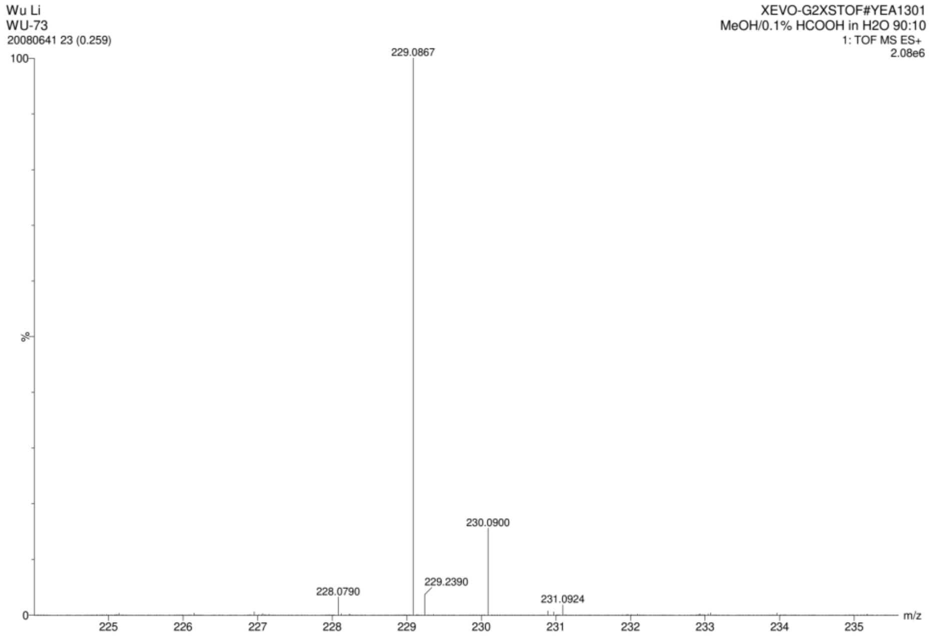

HRMS (EI) of 80b [M]⁺:

File : D:\Xcalibur\data\2005\20051109hrei-av3.RAW
Full ms [215.500 - 247.500] - Range: 215.500 - 247.500
Scan No. 1 of 1

Mass	Absolute Intensity	Relative Intensity	Theoretical Mass	Delta [ppm]	Delta [mmu]	RDB	Composition
218.75913	982750	2.6					
218.98453	1780825	4.8					
226.92047	463855	1.2					
227.93754	2617409	7.0					
228.95383	8993536	24.1					
228.97186	4622711	12.4					
228.98511	425357	1.1					
229.08436	554118	1.5	229.08437	-0.0	-0.0	9.0	$C_{14} H_{11} {}^2H_1 O_3$
229.96961	18778624	50.4					
229.97793	10706688	28.7					
229.98415	628138	1.7					
230.09040	887717	2.4	230.09065	-1.1	-0.3	9.0	$C_{14} H_{10} {}^2H_2 O_3$
230.87232	1079256	2.9					
230.98508	37246464	100.0					
231.09693	929045	2.5	231.09693	0.0	0.0	9.0	$C_{14} H_9 {}^2H_3 O_3$
231.99070	11200256	30.1					
232.00099	17067520	45.8					
232.10310	630581	1.7	232.10320	-0.5	-0.1	9.0	$C_{14} H_8 {}^2H_4 O_3$
232.99736	5168374	13.9					
233.01694	7619781	20.5					
234.03277	2571306	6.9					
235.04937	986601	2.6					
236.06631	486291	1.3					
242.98508	978908	2.6					

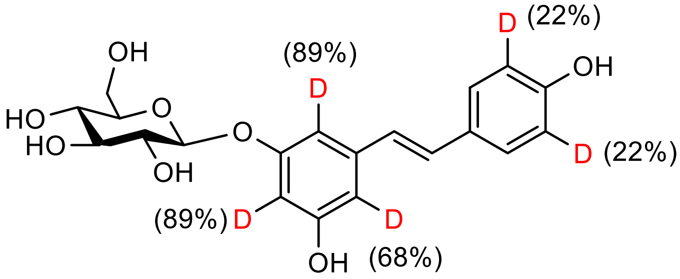

81b
[D] Piceid

According to GP, Fe-Cellulose-1000 (60 mg, 0.05 mol), substrate (88 mg, 0.23 mmol), D_2O (1.5 mL), H_2 (20 bar), room temperature to 120 °C and then at 120 °C for 72 h. The product **81b** (87 mg, 0.22 mmol, 96%) was obtained.

^{1}H NMR (300 MHz, DMSO-d_6) δ 9.53 (d, J = 40.1 Hz, 2H), 7.45 – 7.34 (m, 2H), 7.07 – 6.97 (m, 1H), 6.87 (d, J = 16.4 Hz, 1H), 6.80 – 6.72 (m, 1.33H), 6.67 (m, 0.32H), 6.33 (s, 0.11H), 5.29 (d, J = 4.9 Hz, 1H), 5.06 (dd, J = 17.4, 4.9 Hz, 2H), 4.85 – 4.57 (m, 2H), 3.79 – 3.67 (m, 1H), 3.35 – 3.12 (m, 5H).

^{13}C NMR (101 MHz, DMSO-d_6) δ 159.25, 158.71, 157.36, 139.65, 129.01, 128.45, 128.40, 125.62, 115.98, 101.16, 77.60, 77.16, 73.75, 70.23, 61.18, 60.24, 21.24, 14.56.

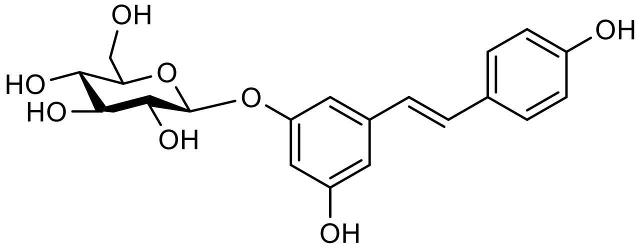

Piceid

Chemical Formula: $C_{20}H_{22}O_8$
Exact Mass: 390.1315

HRMS (ESI-TOF) of 81a:

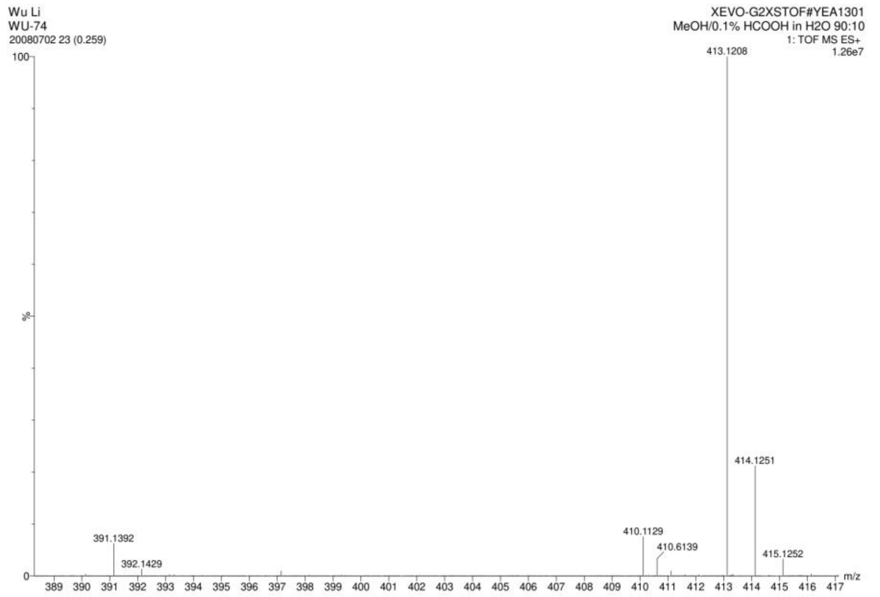

HRMS (ESI-TOF) of 81b [M+Na]$^+$:

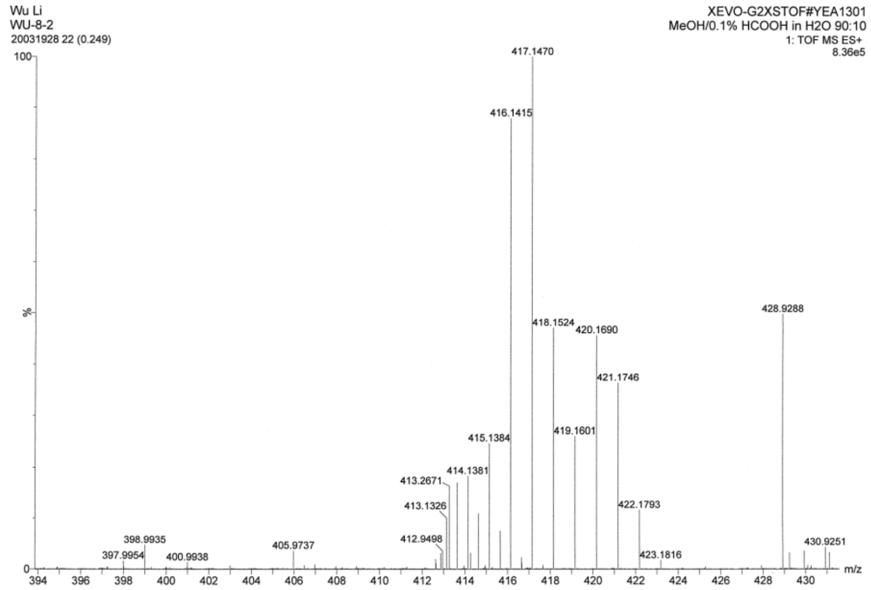

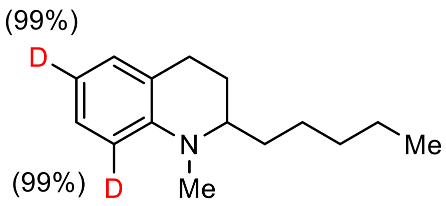

82b

[D] (±)Augustureine

According to GP, Fe-Cellulose-1000 (61 mg, 0.05 mol), substrate (57 mg, 0.26 mmol), D_2O (1.5 mL), H_2 (20 bar), room temperature to 120 °C and then at 120 °C for 24 h. The product **82b** (51 mg, 0.23 mmol, 88%) was obtained.

^{1}H NMR (300 MHz, DMSO-d_6) δ 7.02 – 6.91 (m, 1H), 6.88 (dd, J = 1.8, 1.0 Hz, 1H), 6.47 (m, 0.02H) 3.23 (d, J = 4.3 Hz, 1H), 2.84 (s, 3H), 2.76 – 2.52 (m, 2H), 1.91 – 1.64 (m, 2H), 1.59 – 1.45 (m, 1H), 1.40 – 1.14 (m, 7H), 0.94 – 0.80 (m, 3H).

^{13}C NMR (75 MHz, DMSO-d_6) δ 145.53, 128.62, 127.11, 121.65, 114.15, 110.74, 110.43, 58.34, 37.95, 31.95, 30.90, 25.52, 24.45, 23.40, 22.60, 14.35.

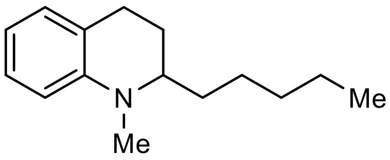

(±)Augustureine

Chemical Formula: $C_{15}H_{23}N$
Exact Mass: 217.1830

HRMS (ESI-TOF) of 82a:

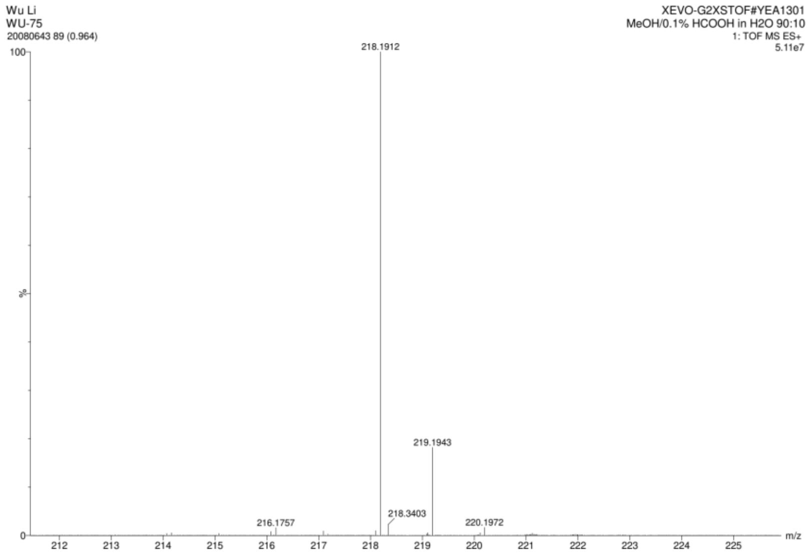

HRMS (ESI-TOF) of 82b [M+H]$^+$:

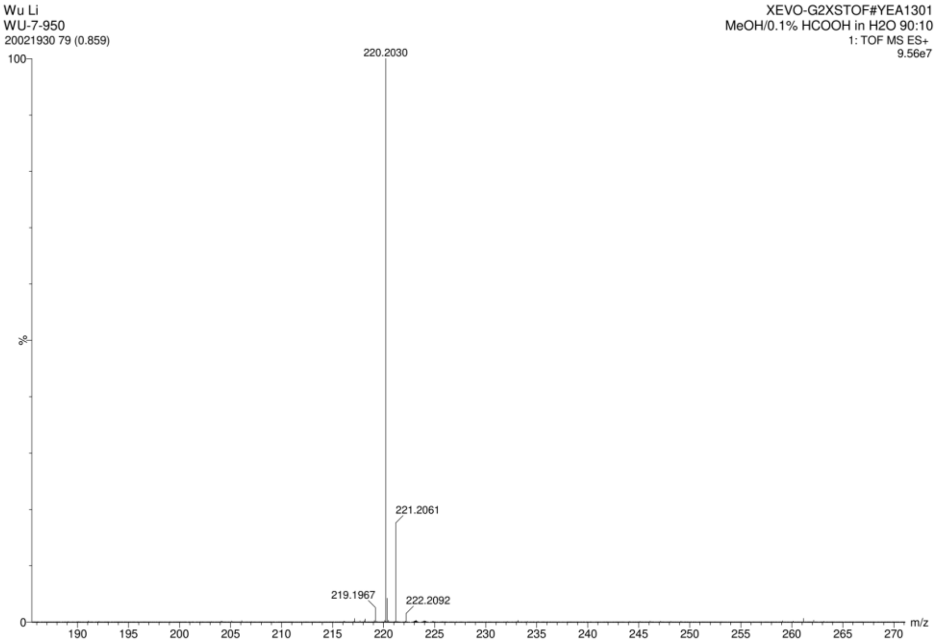

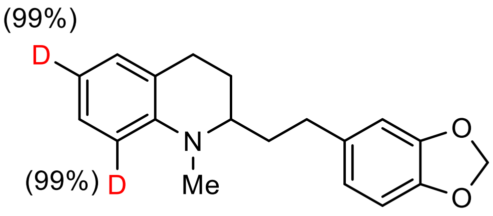

83b
[D] Galipinine

According to GP, Fe-Cellulose-1000 (62 mg, 0.05 mol), substrate (72 mg, 0.24 mmol), D_2O (1.5 mL), H_2 (20 bar), room temperature to 120 °C and then at 120 °C for 24 h. The product **83b** (70 mg, 0.24 mmol, 99%) was obtained.

^{1}H NMR (300 MHz, DMSO-d_6) δ 6.97 (d, J = 1.7 Hz, 1H), 6.93 – 6.86 (m, 1H), 6.84 – 6.77 (m, 2H), 6.72 – 6.63 (m, 1H), 6.47 (m, 0.02H), 5.95 (s, 2H), 3.24 (dq, J = 8.4, 4.2 Hz, 1H), 2.82 (s, 3H), 2.78 – 2.53 (m, 3H), 2.49 – 2.39 (m, 1H), 1.93 (ddt, J = 13.0, 5.5, 3.2 Hz, 1H), 1.85 – 1.69 (m, 2H), 1.56 (dddd, J = 13.4, 10.0, 9.2, 5.5 Hz, 1H).

^{13}C NMR (75 MHz, DMSO-d_6) δ 147.21, 145.16, 145.05, 135.78, 128.19, 126.66, 121.27, 120.89, 108.67, 108.03, 100.57, 57.42, 37.44, 32.55, 31.20, 23.80, 22.92.

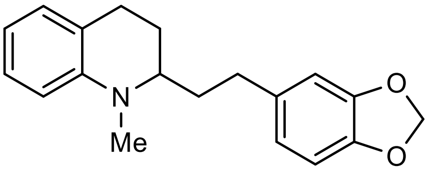

Chemical Formula: $C_{19}H_{21}NO_2$
Exact Mass: 295.1572

Galipinine

HRMS (ESI-TOF) of 83a:

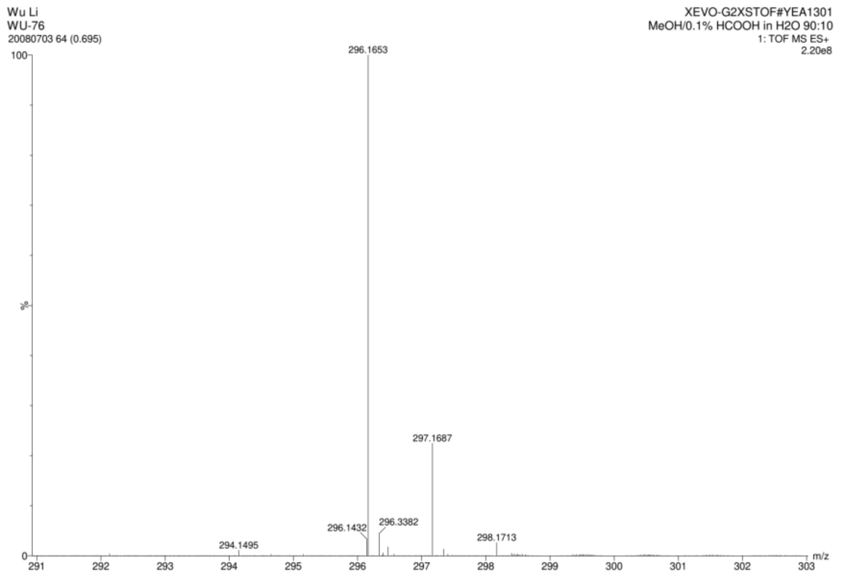

HRMS (ESI-TOF) of 83b [M+H]⁺:

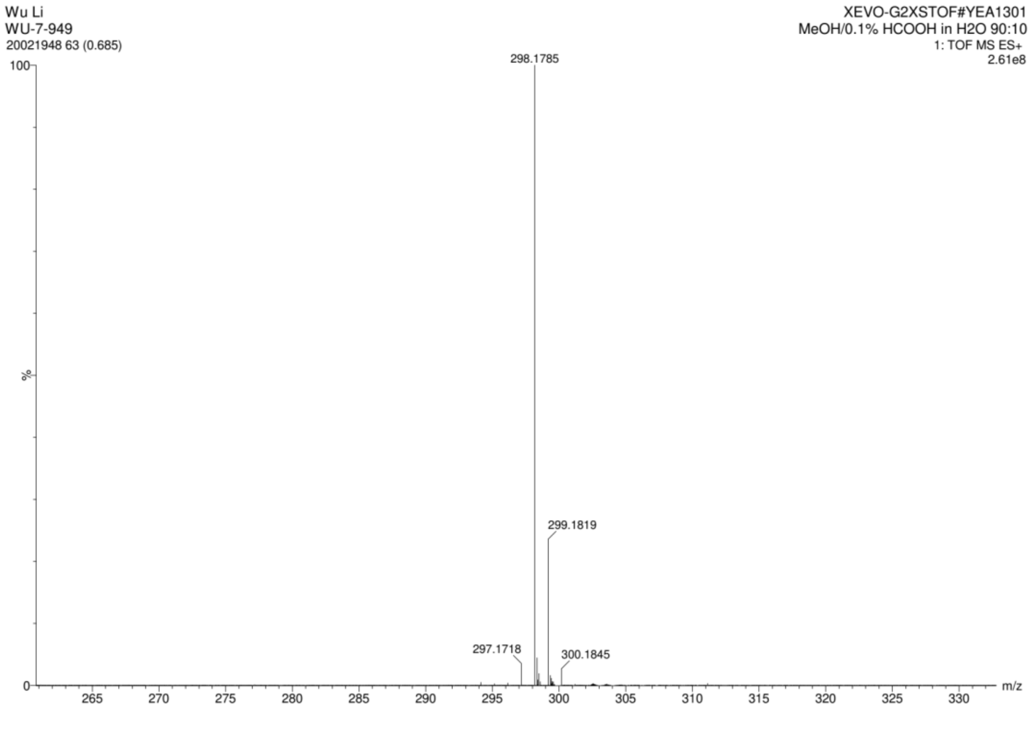

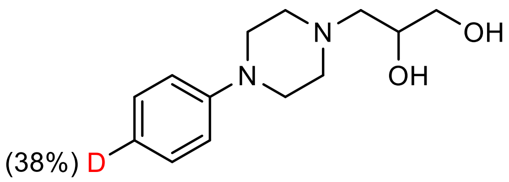

84b

[D] Dropropizine

According to GP, Fe-Cellulose-1000 (60 mg, 0.05 mol), substrate (55 mg, 0.23 mmol), D_2O (1.5 mL), H_2 (20 bar), room temperature to 120 °C and then at 120 °C for 24 h. The product **84b** (51 mg, 0.21 mmol, 91%) was obtained.

^{1}H NMR (300 MHz, DMSO-d_6) δ 7.21 (dt, J = 8.5, 3.5 Hz, 2H), 6.92 (d, J = 8.5 Hz, 2H), 6.77 (t, J = 7.2 Hz, 0.62H), 4.44 (s, 1H), 3.66 (t, J = 5.9 Hz, 1H), 3.11 (t, J = 5.0 Hz, 4H), 2.57 (q, J = 4.2 Hz, 4H), 2.44 (dd, J = 12.6, 5.0 Hz, 1H), 2.29 (dd, J = 12.6, 7.0 Hz, 1H).

^{13}C NMR (75 MHz, DMSO-d_6) δ 151.08, 128.90, 128.79, 118.72, 115.29, 68.76, 64.77, 61.52, 53.49, 48.25.

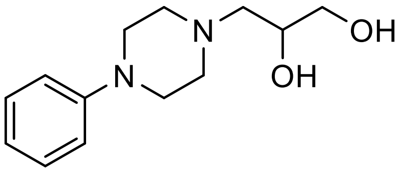

Chemical Formula: $C_{13}H_{20}N_2O_2$

Exact Mass: 236.1525

Dropropizine

HRMS (ESI-TOF) of 84a:

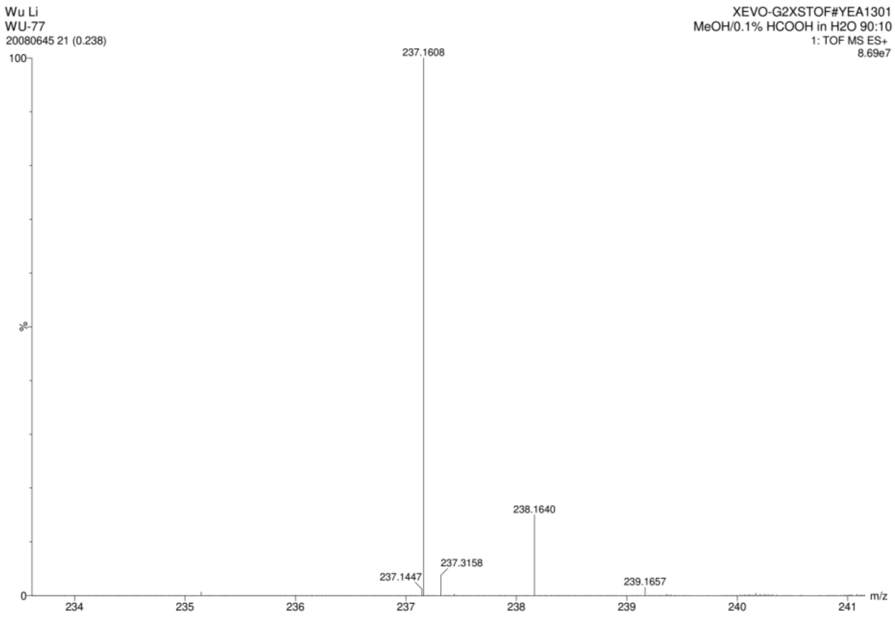

HRMS (ESI-TOF) of 84b [M+H]+:

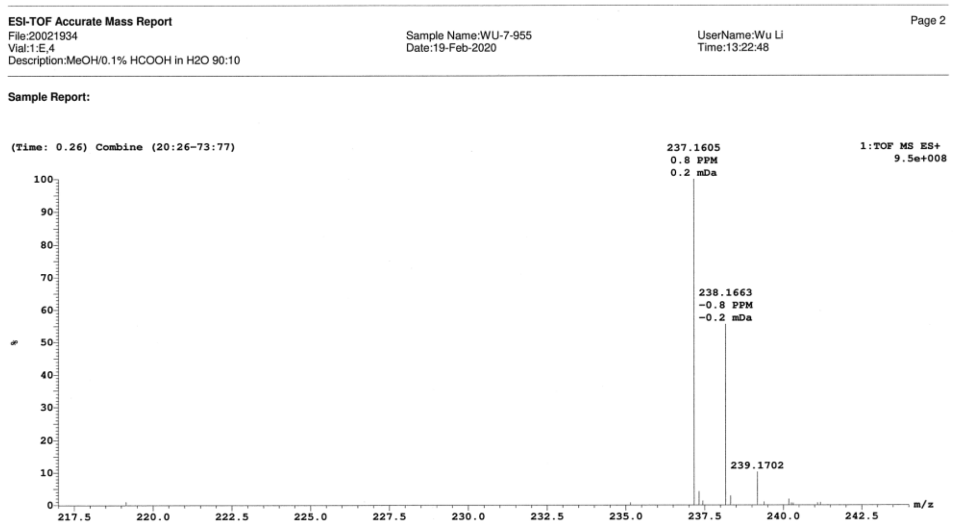

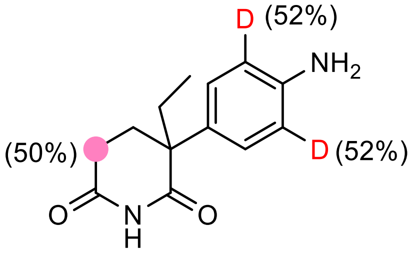

85b

[D] DL-Aminoglutethimide

According to GP, Fe-Cellulose-1000 (61 mg, 0.05 mol), substrate (66 mg, 0.28 mmol), D_2O (1.5 mL), H_2 (20 bar), room temperature to 120 °C and then at 120 °C for 72 h. The product **85b** (60 mg, 0.26 mmol, 91%) was obtained.

^{1}H NMR (300 MHz, DMSO-d_6) δ 10.65 (s, 1H), 7.00 – 6.80 (m, 2H), 6.56 (d, J = 8.8 Hz, 0.96H), 2.39 – 1.81 (m, 3.00H), 1.80 – 1.59 (m, 2H), 0.70 (td, J = 7.4, 2.9 Hz, 3H).

^{13}C NMR (75 MHz, DMSO-d_6) δ 177.15, 174.24, 147.61, 147.55, 127.16, 127.06, 126.92, 114.82, 49.73, 32.67, 26.10, 9.23.

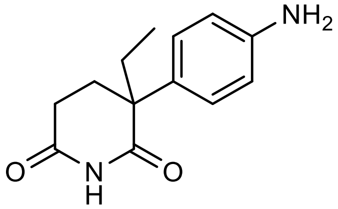

Chemical Formula: $C_{13}H_{16}N_2O_2$
Exact Mass: 232.1212

DL-Aminoglutethimide

HRMS (ESI-TOF) of 85a:

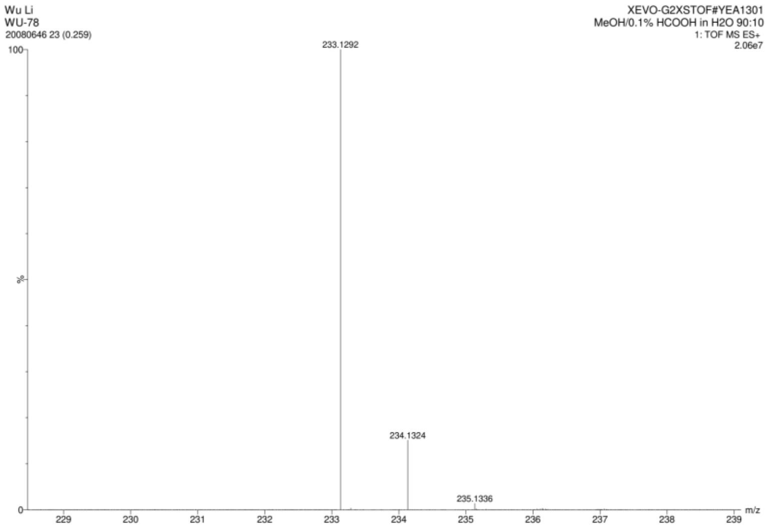

HRMS (EI) of 85b $[M]^+$:

Wu Li Wu-7-869 HR(EI)

File : D:\Xcalibur\data\2002\20022601hrei-av2.RAW
Full ms [215.500 - 247.500] - Range: 234.000 - 234.500
Scan No. 1 of 1

Mass	Absolute Intensity	Relative Intensity	Theoretical Mass	Delta [ppm]	Delta [mmu]	RDB	Composition
234.13435	4821496	100.0	234.13473	-1.6	-0.4	6.5	$C_{13}H_{16}{}^{2}H_{1}O_{2}N_{2}$

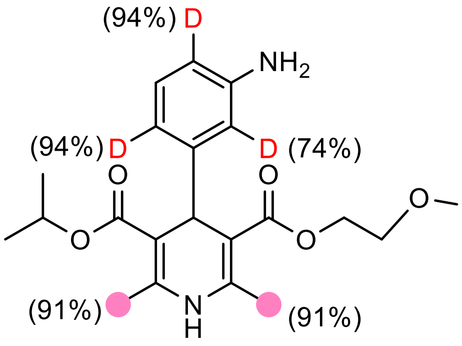

86b
[D] Nimodipine-NH_2

According to GP, Fe-Cellulose-1000 (62 mg, 0.05 mol), substrate (75 mg, 0.19 mmol), D_2O (1.5 mL), H_2 (20 bar), room temperature to 120 °C and then at 120 °C for 24 h. The product **86b** (62 mg, 0.16 mmol, 96%) was obtained.

^{1}H NMR (300 MHz, DMSO-d_6) δ 8.68 (s, 1H), 6.81 (s, 1H), 4.92 – 4.72 (m, 4H), 6.38 (s, 0.26H), 6.30 (m, 0.13H), 4.16 – 3.96 (m, 2H), 3.50 (dd, J = 5.5, 4.1 Hz, 2H), 3.27 (s, 3H), 2.30 – 2.15 (m, 0.54H), 1.21 – 1.15 (m, 3H), 1.10 (d, J = 6.2 Hz, 3H).

^{13}C NMR (75 MHz, DMSO-d_6) δ 167.14, 166.69, 148.40, 147.95, 145.11, 144.36, 127.84, 102.44, 101.65, 70.02, 65.96, 62.28, 58.14, 21.92, 21.66.

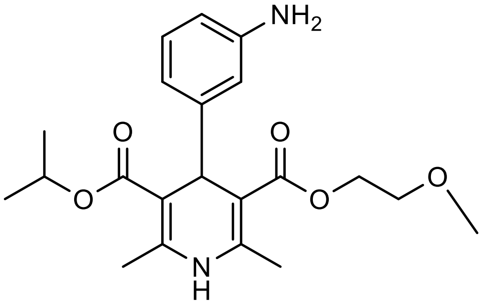

Nimodipine-NH_2

Chemical Formula: $C_{21}H_{28}N_2O_5$
Exact Mass: 388.1998

HRMS (ESI-TOF) of 86a:

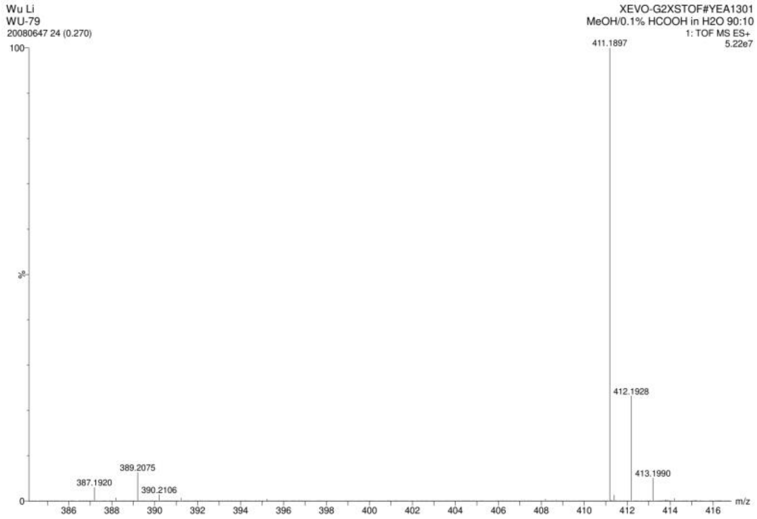

HRMS (ESI-TOF) of 86b [M+H]$^+$:

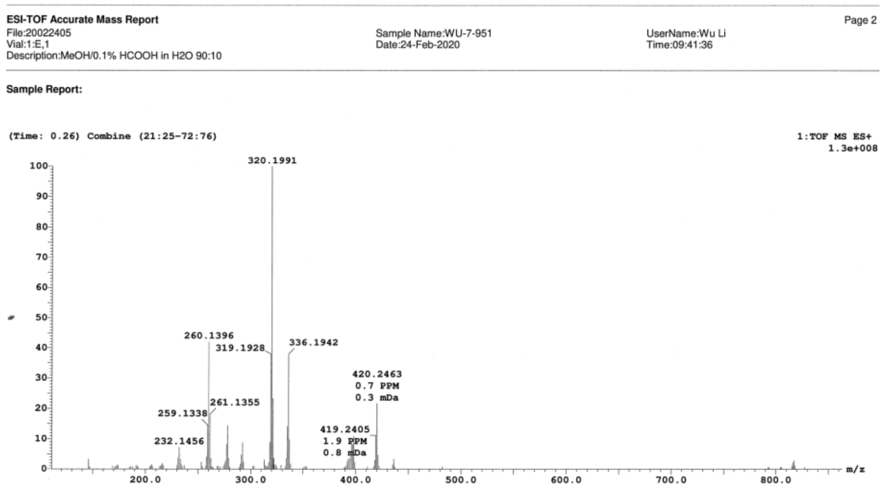

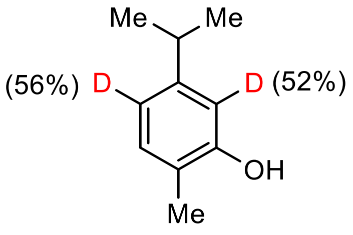

87b
[D] Carvacrol

According to GP, Fe-Cellulose-1000 (60 mg, 0.05 mol), substrate (42 mg, 0.28 mmol), D_2O (1.5 mL), H_2 (20 bar), room temperature to 120 °C and then at 120 °C for 72 h. The product **87b** (37 mg, 0.24 mmol, 86%) was obtained.

^{1}H NMR (300 MHz, DMSO-*d*$_6$) δ 9.05 (s, 1H), 6.93 (td, *J* = 3.8, 3.3, 0.8 Hz, 1H), 6.64 (s, 0.48H), 6.56 (m, 0.44H), 2.82 – 2.64 (m, 1H), 2.06 (d, *J* = 0.7 Hz, 3H), 1.14 (d, *J* = 6.9 Hz, 6H).

^{13}C NMR (75 MHz, DMSO-*d*$_6$) δ 155.19, 155.15, 147.00, 146.92, 146.84, 130.31, 130.20, 120.96, 116.66, 112.52, 33.07, 33.02, 32.97, 24.01, 15.61.

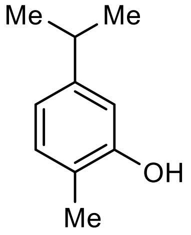

Chemical Formula: $C_{10}H_{14}O$
Exact Mass: 150.1045

Carvacrol

HRMS (EI) of 87b [M]$^+$:

Wu Li 8-8 HR(EI)

File : D:\Xcalibur\data\2005\20051108hrei-av2.RAW
Full ms [139.500 - 173.500] - Range: 151.000 - 151.500
Scan No. 1 of 1

Mass	Absolute Intensity	Relative Intensity	Theoretical Mass	Delta [ppm]	Delta [mmu]	RDB	Composition
151.11065	1276777	14.0	151.11019	3.0	0.5	4.0	$C_{10}H_{13}{}^2H_1O_1$
151.14879	228516	2.5					

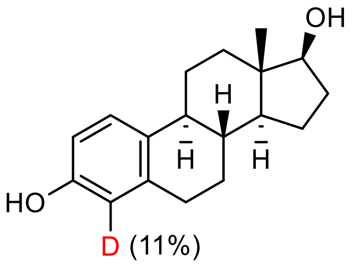

88b
[D] Estradiol

According to GP, Fe-Cellulose-1000 (60 mg, 0.05 mol), substrate (65 mg, 0.24 mmol), D_2O (1.5 mL), H_2 (20 bar), room temperature to 120 °C and then at 120 °C for 72 h. The product **88b** (65 mg, 0.24 mmol, 99%) was obtained.

^{1}H NMR (300 MHz, DMSO-d_6) δ 9.17 (s, 1H), 7.00 (d, *J* = 8.4 Hz, 1H), 6.49 (dd, *J* = 8.3, 2.5 Hz, 1H), 6.42 (d, *J* = 2.6 Hz, 1H), 4.74 (d, *J* = 4.7 Hz, 1H), 3.49 (td, *J* = 8.4, 4.3 Hz, 1H), 2.65 (q, *J* = 4.5, 4.0 Hz, 2H), 2.29 – 2.08 (m, 1H), 2.08 – 1.93 (m, 1H), 1.93 – 1.66 (m, 3H), 1.52 (dd, *J* = 12.3, 6.5 Hz, 1H), 1.42 – 0.94 (m, 7H), 0.62 (s, 3H).

^{13}C NMR (75 MHz, DMSO-d_6) δ 155.17, 137.74, 131.14, 126.56, 115.45, 113.22, 80.68, 49.94, 43.97, 43.25, 39.17, 36.97, 30.19, 29.62, 27.40, 26.56, 23.23, 11.70.

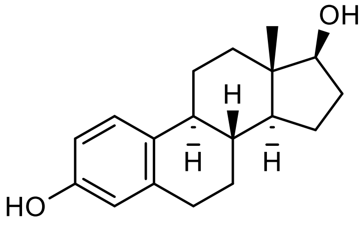

Estradiol

Chemical Formula: $C_{18}H_{24}O_2$
Exact Mass: 272.1776

HRMS (ESI-TOF) of 88b [M+H]$^+$:

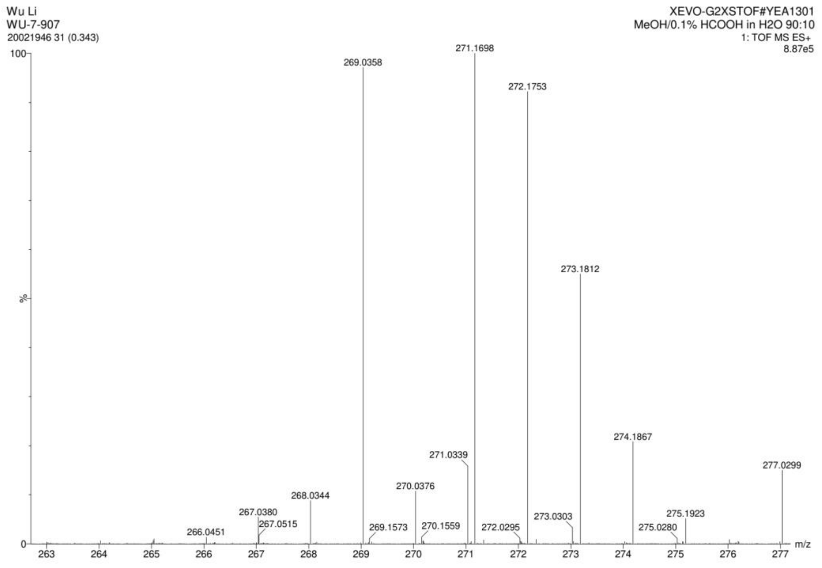

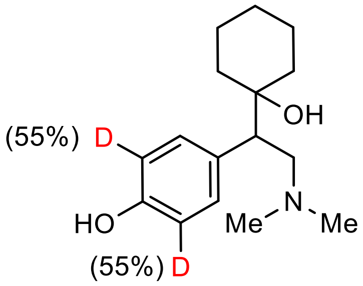

89b

[D] O-Desmethylvenlafaxine

According to GP, Fe-Cellulose-1000 (60 mg, 0.05 mol), substrate (65 mg, 0.25 mmol), D_2O (1.5 mL), H_2 (20 bar), room temperature to 120 °C and then at 120 °C for 24 h. The product **89b** (57 mg, 0.22 mmol, 86%) was obtained.

^{1}H NMR (300 MHz, DMSO-d_6) δ 9.14 (s, 1H), 6.96 (s, 2H), 6.63 (d, *J* = 8.0 Hz, 0.91H), 5.42 (s, 1H), 2.99 (t, *J* = 10.4 Hz, 1H), 2.71 (t, *J* = 7.5 Hz, 1H), 2.34 (dd, *J* = 12.7, 6.1 Hz, 1H), 2.14 (s, 6H), 1.66 – 0.75 (m, 10H).

^{13}C NMR (75 MHz, DMSO-d_6) δ 155.99, 132.17, 130.54, 130.43, 114.85, 73.01, 60.89, 52.08, 45.77, 37.61, 32.84, 26.16, 21.70.

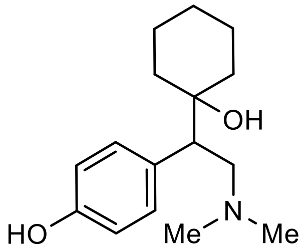

Chemical Formula: $C_{16}H_{25}NO_2$
Exact Mass: 263.1885

O-Desmethylvenlafaxine

HRMS (ESI-TOF) of 89a:

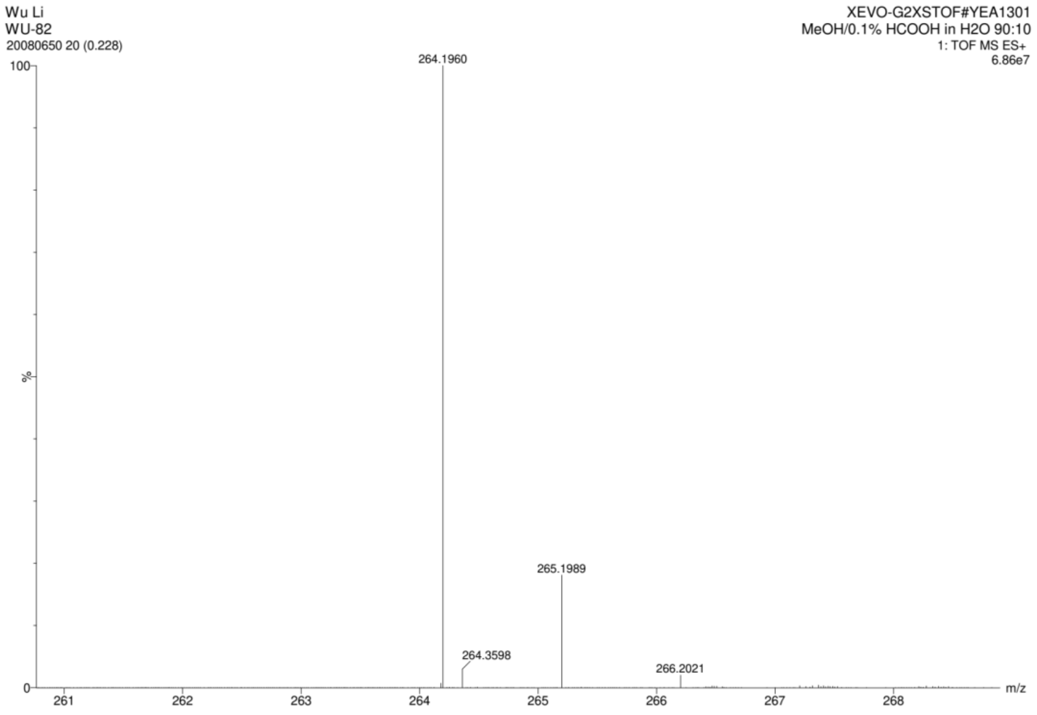

HRMS (ESI-TOF) of 89b [M+H]$^+$:

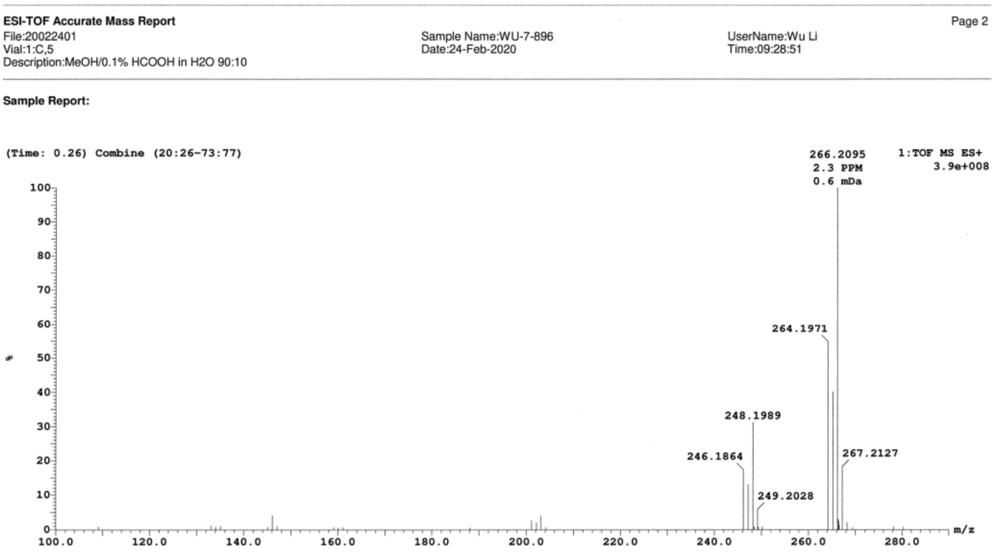

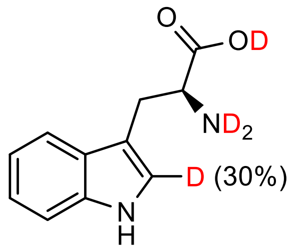

90b

[D] L-Tryptophan

According to GP, Fe-Cellulose-1000 (60 mg, 0.05 mol), substrate (52 mg, 0.25 mmol), D_2O (1.5 mL), H_2 (20 bar), room temperature to 120 °C and then at 120 °C for 24 h. The product **90b** (52 mg, 0.24 mmol, >99%) was obtained.

^{1}H NMR (300 MHz, D_2O) δ 7.64 (d, *J* = 7.9 Hz, 1H), 7.44 (d, *J* = 8.1 Hz, 1H), 7.22 (s, 0.70H), 7.21 – 7.06 (m, 2H), 3.95 (dd, *J* = 8.1, 4.8 Hz, 1H), 3.36 (d, *J* = 4.8 Hz, 1H), 3.23 (dd, *J* = 15.7, 7.6 Hz, 1H).

^{13}C NMR (75 MHz, D_2O) δ 174.54, 136.29, 126.62, 124.98, 122.09, 119.42, 118.41, 111.91, 107.48, 55.04, 26.38.

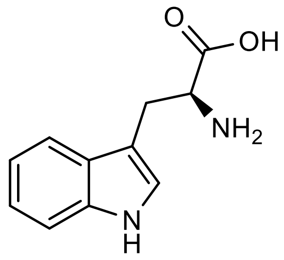

L-Tryptophan

Chemical Formula: $C_{11}H_{12}N_2O_2$

Exact Mass: 204.0899

HRMS (ESI-TOF) for *L-Tryptophan:*

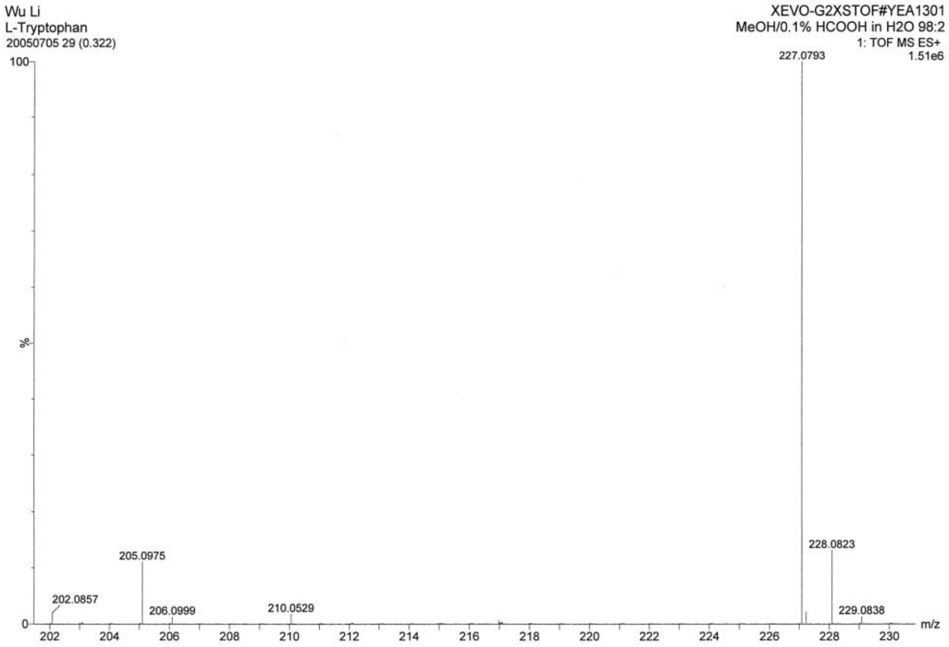

HRMS (ESI-TOF) [D] L-Tryptophan:

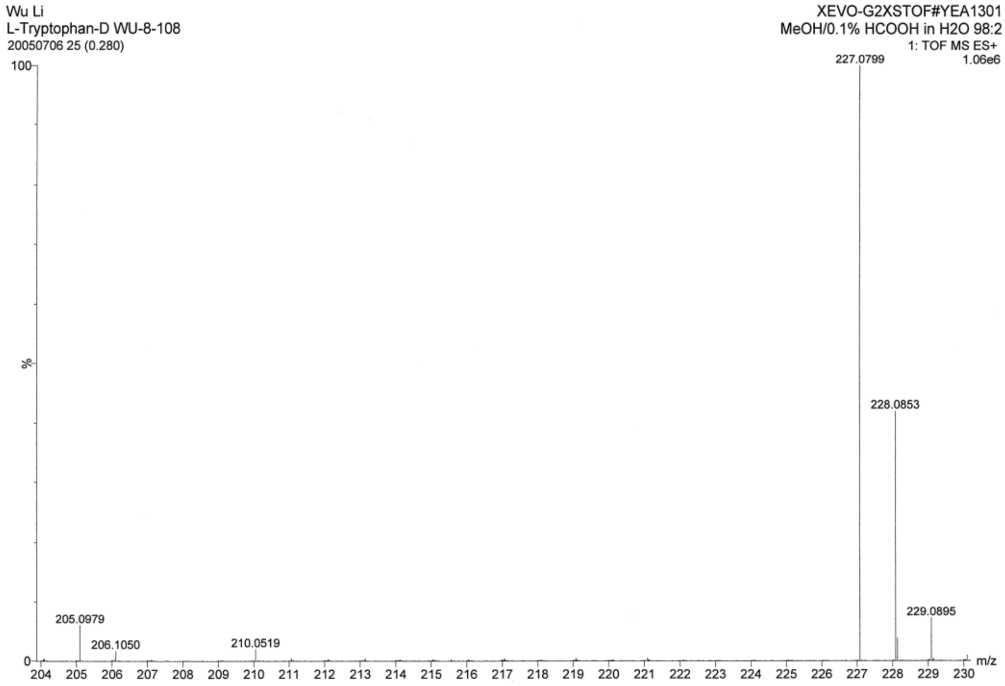

HPLC for *L-Tryptophan:*

```
Data File D:\CHEM32\1\DATA\2005\20050000701.D
Sample Name: L-Tryptophan

=====================================================================
Acq. Operator   : SYSTEM                          Seq. Line :   2
Acq. Instrument : LC4                              Location : Vial 2
Injection Date  : 5/7/2020 10:18:54 AM                  Inj :   1
                                                 Inj Volume : 1.000 µl
Acq. Method     : C:\CHEM32\1\METHODS\RUI-HCOOH-KAL-PROPSR-21.04.2020.M
Last changed    : 4/24/2020 3:48:28 PM by SYSTEM
Analysis Method : C:\CHEM32\1\METHODS\METHOD 4-FA.M
Last changed    : 5/7/2020 12:26:22 PM by SYSTEM
                  (modified after loading)
Additional Info : Peak(s) manually integrated
```

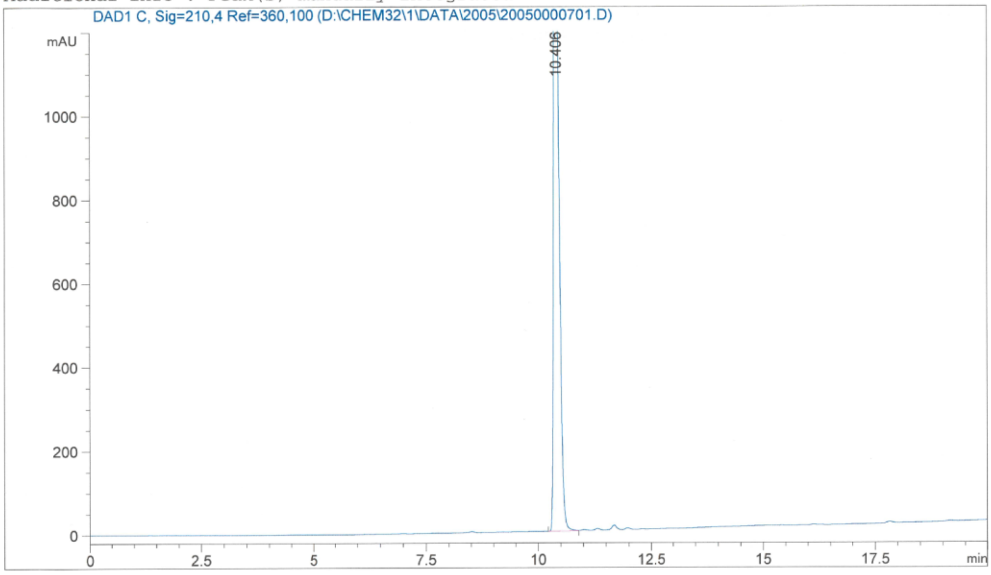


```
=====================================================================
                    Internal Standard Report
=====================================================================

Sorted By             :      Signal
Multiplier            :      1.0000
Dilution              :      1.0000
Do not use Multiplier & Dilution Factor with ISTDs

Signal 1: DAD1 C, Sig=210,4 Ref=360,100

=====================================================================
=====================================================================
                      Area Percent Report
=====================================================================

Sorted By             :      Signal
Multiplier            :      1.0000
Dilution              :      1.0000
Do not use Multiplier & Dilution Factor with ISTDs
```

HPLC for [D] L-Tryptophan:

```
File D:\CHEM32\1\DATA\2005\20050000700.D
Sample Name: Wu-8-108

=====================================================================
Acq. Operator   : SYSTEM                        Seq. Line :   1
Acq. Instrument : LC4                            Location : Vial 1
Injection Date  : 5/7/2020 9:18:02 AM                 Inj :   1
                                               Inj Volume : 1.000 µl
Acq. Method     : C:\CHEM32\1\METHODS\RUI-HCOOH-KAL-PROPSR-21.04.2020.M
Last changed    : 4/24/2020 3:48:28 PM by SYSTEM
Analysis Method : C:\CHEM32\1\METHODS\METHOD 4-FA.M
Last changed    : 5/7/2020 12:26:22 PM by SYSTEM
                  (modified after loading)
Additional Info : Peak(s) manually integrated
```

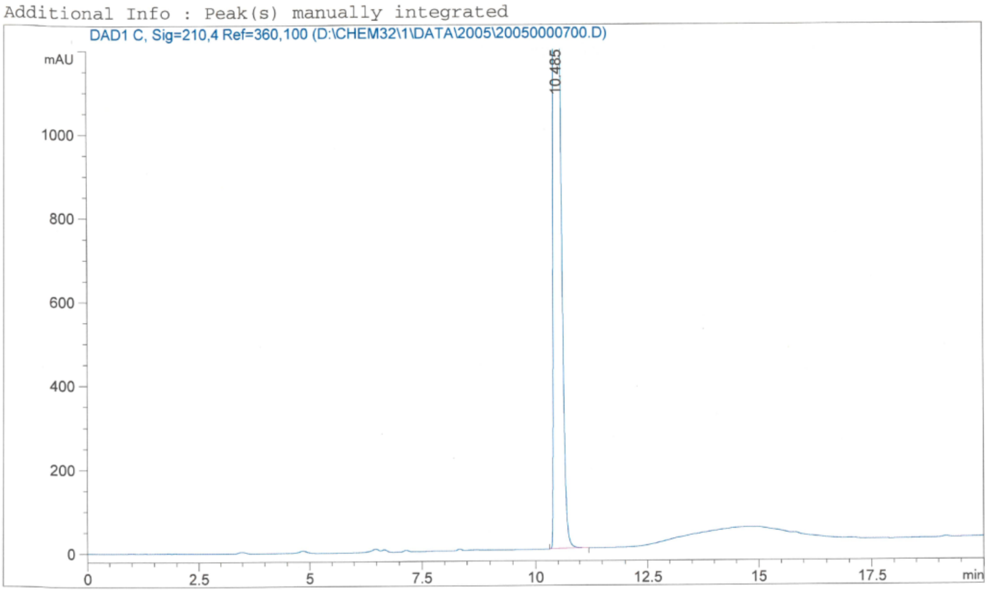


```
=====================================================================
                     Internal Standard Report
=====================================================================

Sorted By             :      Signal
Multiplier            :      1.0000
Dilution              :      1.0000
Do not use Multiplier & Dilution Factor with ISTDs

Signal 1: DAD1 C, Sig=210,4 Ref=360,100

=====================================================================
=====================================================================
                        Area Percent Report
=====================================================================

Sorted By             :      Signal
Multiplier            :      1.0000
Dilution              :      1.0000
Do not use Multiplier & Dilution Factor with ISTDs
```

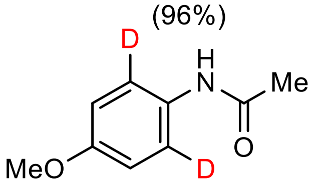

91b

Substrate **4b** (63 mg, 0.5 mmol), acetic anhydride 108 mg (1.0 mmol) in ethyl acetate at room temperature for 1 h. After removal of all volatiles in vacuum the desired product **91b** were obtained.

^{1}H NMR (300 MHz, DMSO-d_6) δ 9.77 (s, 1H), 7.49 (m, 0.09H), 6.86 (s, 2H), 3.71 (s, 3H), 2.00 (s, 3H).

^{13}C NMR (75 MHz, DMSO-d_6) δ 168.16, 155.46, 132.87, 120.97, 120.67, 114.12, 55.58, 24.26.

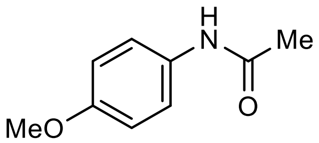

Chemical Formula: $C_9H_{11}NO_2$
Exact Mass: 165.0790

HRMS (ESI-TOF) of 91b [M+H]$^+$:

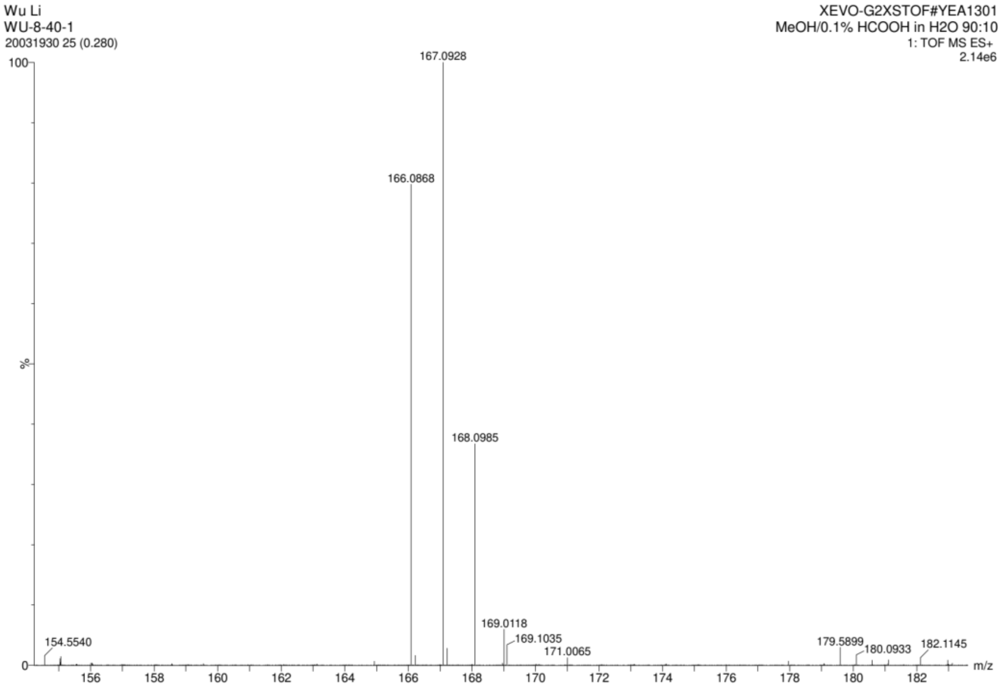

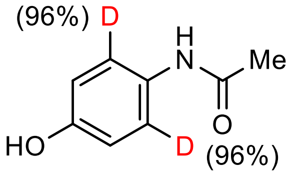

92b (71%)
[D] Paracetamol

A 10 mL round bottom flask was charged with **91b** (0.5 mmol, 84 mg), added dry dichloromethane (2 mL) and the reaction mixture was cooled to 0 °C followed by the slow addition of BBr_3 (0.56 mmol, 140 mg) under argon. The reaction mixture was stirred at 0 °C for 1.5 h. After completion of the reaction (progress was monitored by TLC; SiO_2, hexane/ethyl acetate=1:1 the reaction mixture was quenched with NH_4Cl solution (15 mL) and extracted with ethyl acetate (3x10 mL). The combined organic layer was washed with brine (3x10 mL) and dried over anhydrous Na_2SO_4. Solvent was removed under reduced pressure and the remaining residue was purified by column chromatography to obtain the desired product **92b** in 71%[13] .

^{1}H NMR (300 MHz, DMSO-d_6) δ 9.65 (s, 1H), 9.13 (s, 1H), 7.36 (m, 0.09H), 6.68 (s, 2H), 1.99 (s, 3H).

^{13}C NMR (75 MHz, DMSO-d_6) δ 167.55, 153.15, 130.93, 120.86, 120.55, 120.23, 114.92, 23.77.

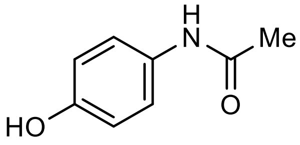

Chemical Formula: $C_8H_9NO_2$
Exact Mass: 151.0633

Paracetamol

HRMS (ESI-TOF) of 92b [M+H]$^+$:

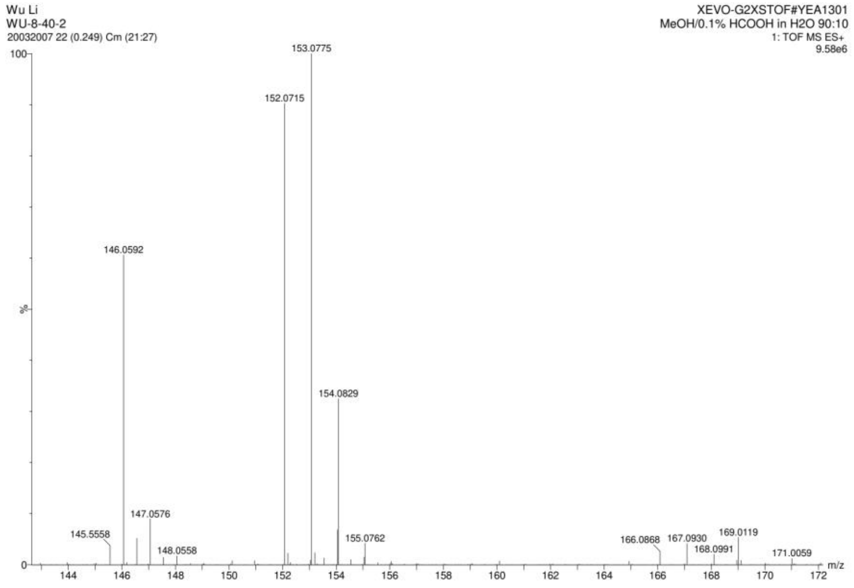

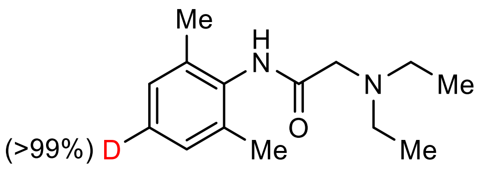

93b

[D] Lidocaine

[D] Lidocaine **93b** was obtained according to the procedure in[14].

^{1}H NMR (400 MHz, DMSO-d_6) δ 9.16 (s, 1H), 7.14 – 6.99 (m, 2H), 3.14 (s, 2H), 2.62 (q, *J* = 7.1 Hz, 4H), 2.14 (d, *J* = 0.7 Hz, 6H), 1.08 (t, *J* = 7.1 Hz, 6H).

^{13}C NMR (101 MHz, DMSO-d_6) δ 170.02, 135.68, 135.58, 127.97, 126.19, 57.33, 48.52, 18.65, 12.63.

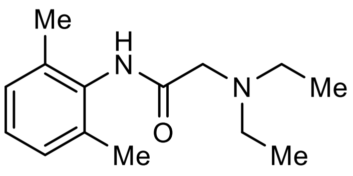

Chemical Formula: $C_{14}H_{22}N_2O$
Exact Mass: 234.1732

Lidocaine

HRMS (ESI-TOF) of 93b [M+H]$^+$:

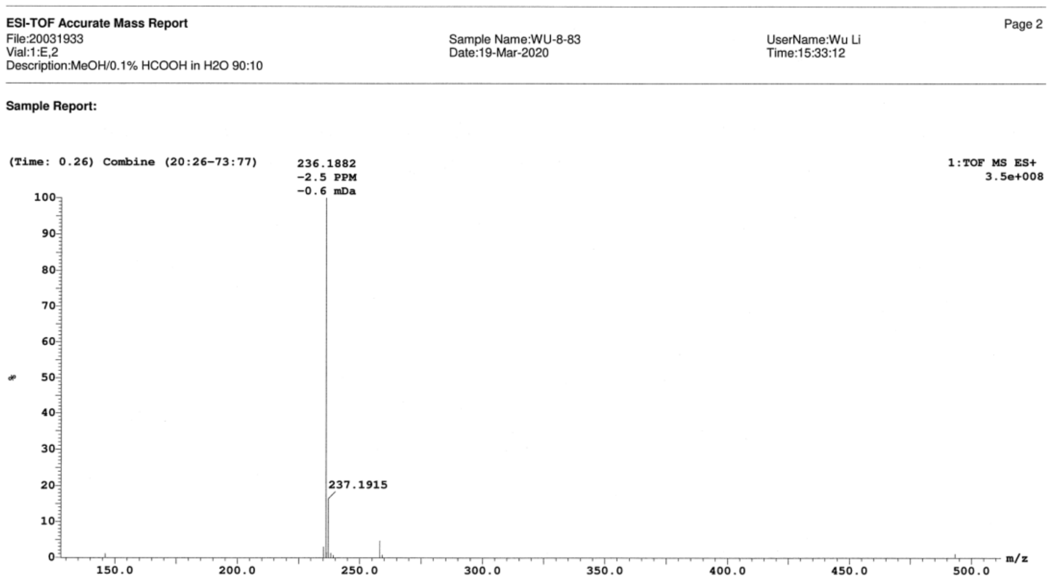

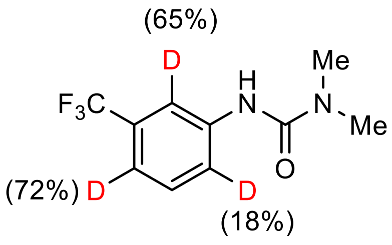

94b
[D] Fluometuron

[D] Fluometuron **94b** is obtained from **10b**.

^{1}H NMR (300 MHz, DMSO-d_6) δ 8.61 (s, 1H), 7.94 (m, 0.35), 7.76 (dp, J = 8.4, 0.7 Hz, 0.82H), 7.51 – 7.35 (m, 1H), 7.26 (m, 0.28), 2.94 (s, 6H).

^{13}C NMR (75 MHz, DMSO-d_6) δ 155.90, 145.83, 142.02, 129.80, 129.69, 129.57, 126.64, 123.26, 36.65.

^{19}F NMR (282 MHz, DMSO-d_6) δ -61.24.

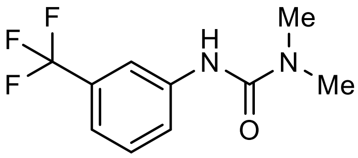

Fluometuron

Chemical Formula: $C_{10}H_{11}F_3N_2O$
Exact Mass: 232.0823

HRMS (ESI-TOF) of 94b [M+H]$^{+}$:

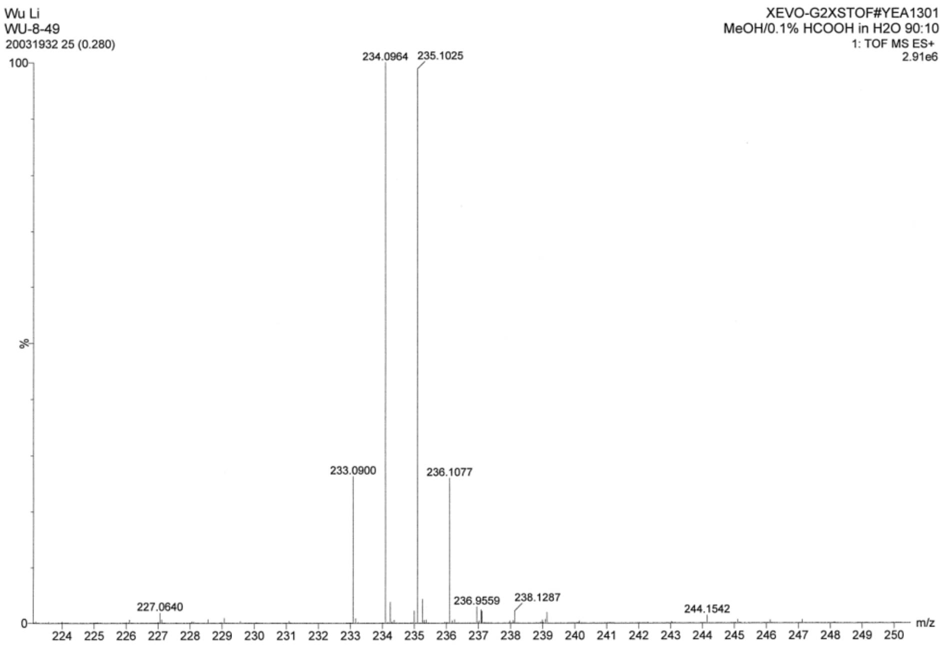

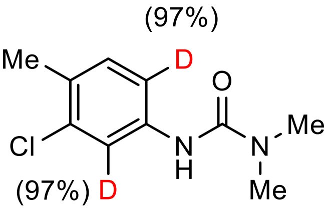

95b
[D] Clortoluron

[D] Clortoluron **95b** is obtained from **17b**.

^{1}H NMR (300 MHz, DMSO-d_6) δ 8.35 (s, 1H), 7.64 (s, 0.03H), 7.34 (m, 0.03H), 7.18 (d, J = 0.8 Hz, 1H), 2.92 (s, 6H), 2.24 (d, J = 0.7 Hz, 3H).

^{13}C NMR (75 MHz, DMSO-d_6) δ 155.93, 140.30, 132.96, 130.98, 128.23, 36.64, 19.21.

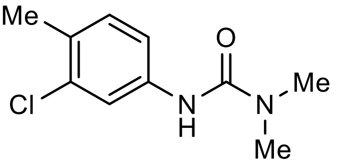

Chemical Formula: $C_{10}H_{13}ClN_2O$
Exact Mass: 212.0716

Clortoluron

HRMS (ESI-TOF) of 95b [M+H]$^+$:

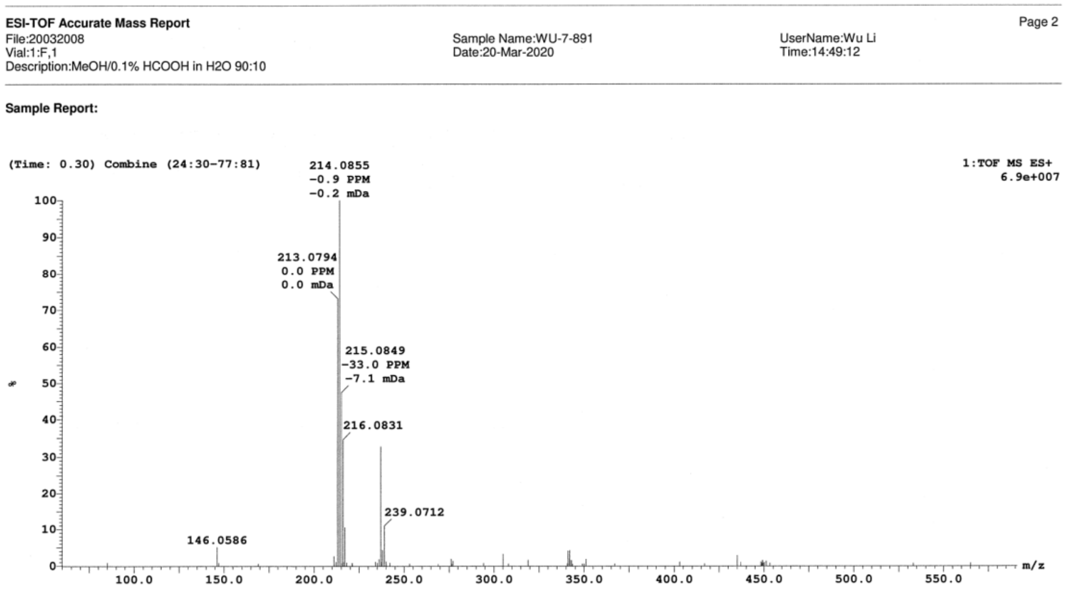

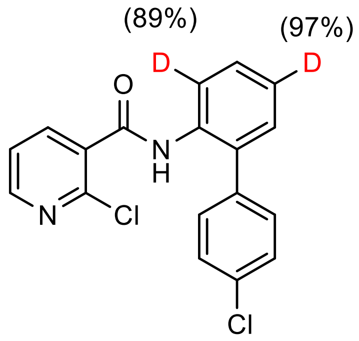

96b

[D] Boscalid

[D] Boscalid **96b** is obtained from **14b**.

^{1}H NMR (300 MHz, DMSO-d_6) δ 10.18 (s, 1H), 8.49 (dd, J = 4.8, 1.9 Hz, 1H), 7.89 (dd, J = 7.5, 1.9 Hz, 1H), 7.57 – 7.44 (m, 6H), 7.41 (d, J = 1.6 Hz, 1.09H).

^{13}C NMR (75 MHz, DMSO-d_6) δ 164.69, 150.79, 146.98, 138.30, 138.17, 136.81, 134.37, 133.45, 132.67, 131.21, 130.65, 128.79, 128.56, 123.49.

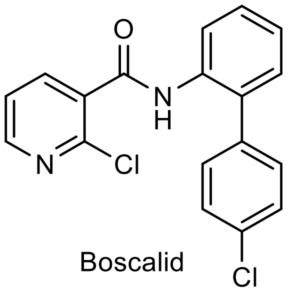

Boscalid

Chemical Formula: $C_{18}H_{12}Cl_2N_2O$
Exact Mass: 342.0327

HRMS (ESI-TOF) of 96b [M+H]$^+$:

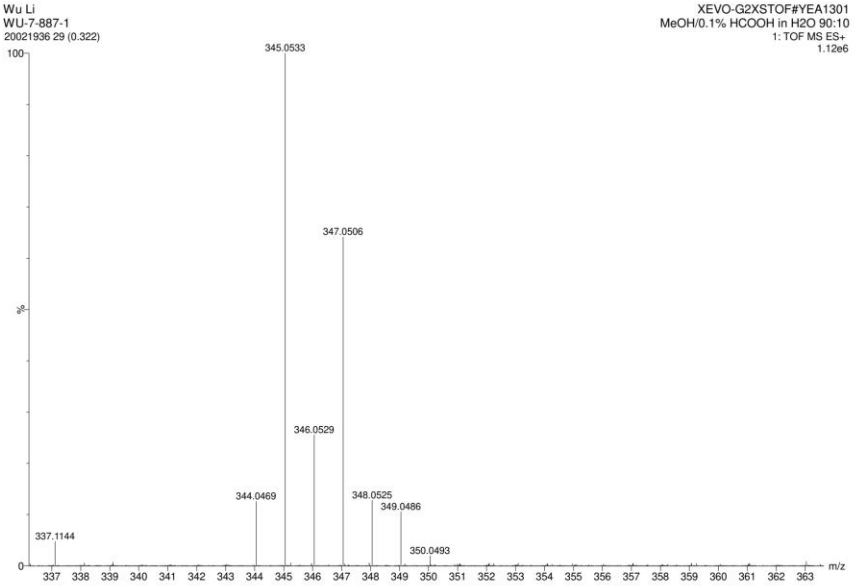

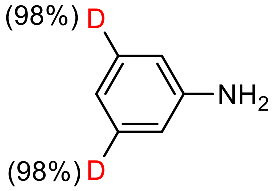

2a

According to GP, Fe-cellulose-1000 (60 mg, 0.05 mol), H_2O 1.5 mL, substrate aniline-2,3,4,5,6-*d*$_5$ (98 atom% D, 45 mg, 0.46 mmol), H_2 (20 bar), room temperature to 120 °C and then at 120 °C for 24 h. Ethyl acetate was added to the crude reaction. The reaction mixture was centrifuged, and the organic layer was removed from the vials (3 times). After removal of all volatiles in vacuo the desired product **2a** was obtained.

^{1}H NMR (400 MHz, DMSO-*d*$_6$) δ 6.57 (s, 2H), 6.49 (s, 1H), 4.96 (s, 2H).

^{13}C NMR (101 MHz, DMSO-*d*$_6$) δ 170.84, 149.00, 129.18, 128.95, 128.71, 115.92, 114.25.

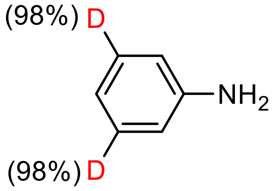

2a

HRMS (EI) of 2a [M]$^+$:

ESI-TOF Accurate Mass Report Page 1

Results file: E:\Projects\2005.PRO\SampleDB\2005.rpt
Last modified: Thursday, May 07, 2020 16:22:17

Sample Summary:

Sample	File	Sample Name	User	Target	Formula	Expected Mass	Observed Mass	Error PPM	Error mDa
122	20050723	WU-8-289	Wu Li	95.0704<br>220.9670	C6H5N[2H2]<br>C6H4IN[2H2]				

13. ^{1}H NMR, ^{13}C NMR and ^{19}F NMR spectra for substrates and products

^{1}H NMR for 3a:

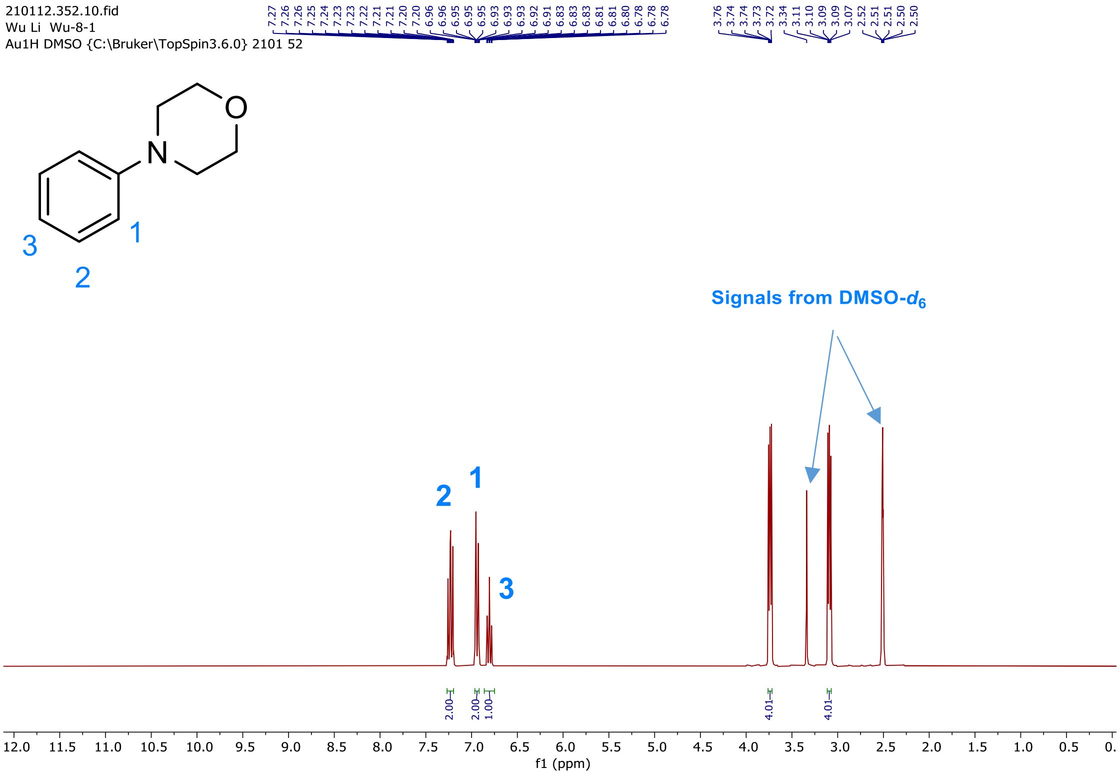

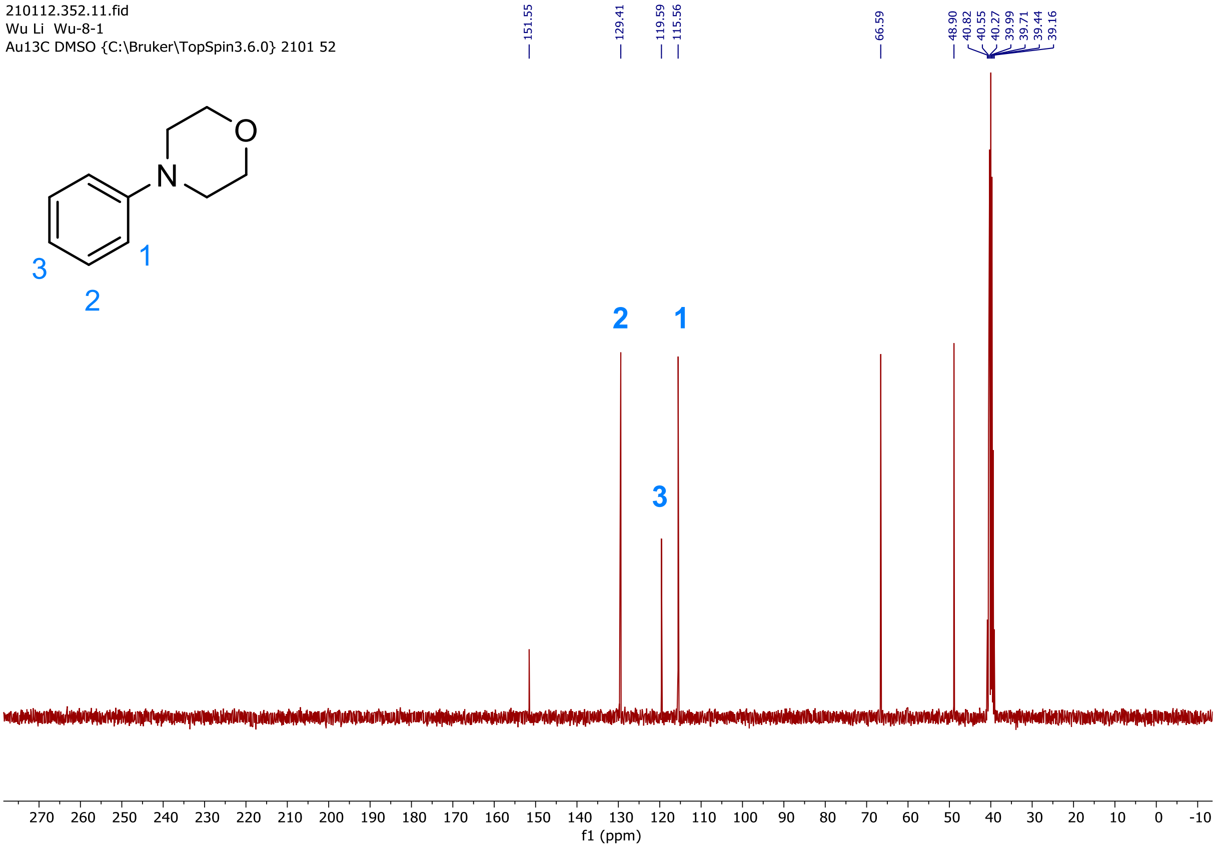

Original spectra for 3b:

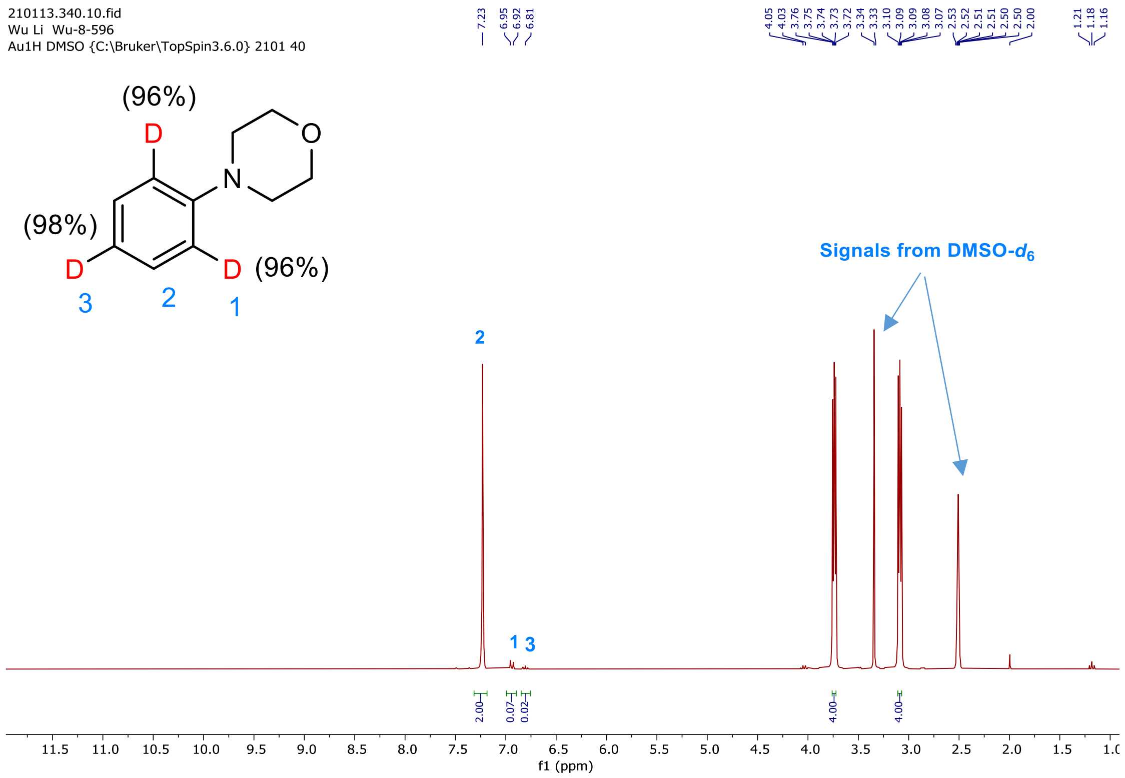

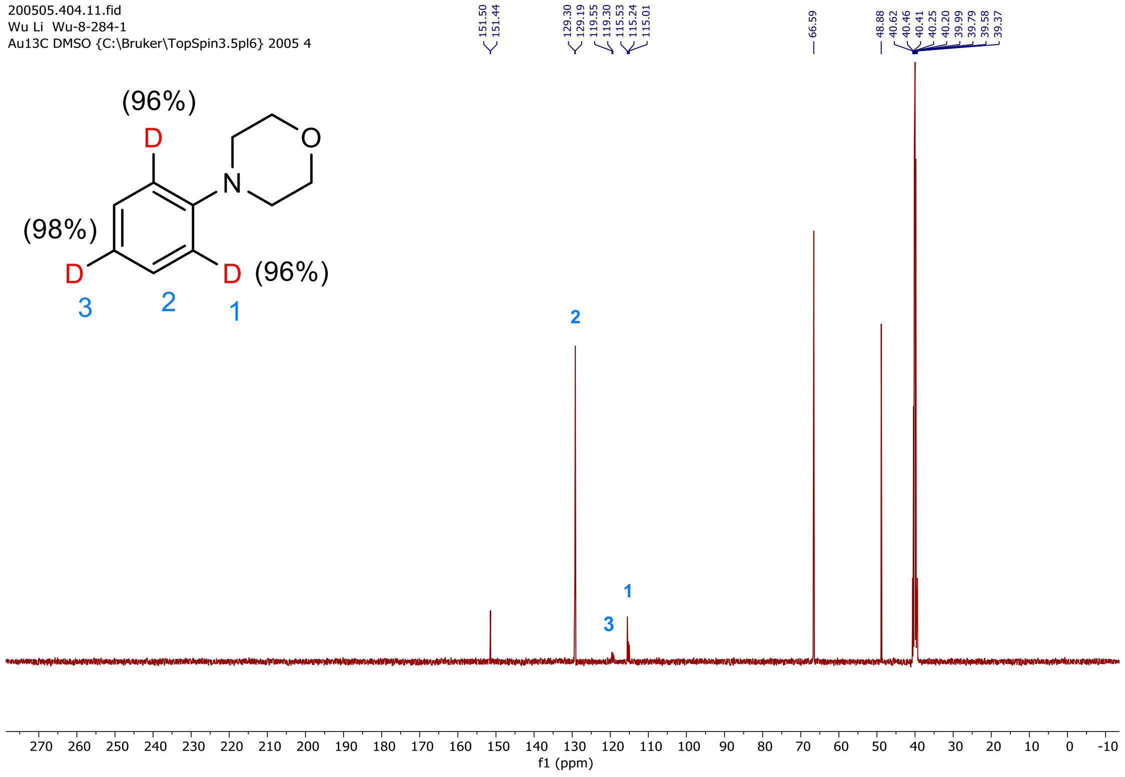

^{1}H NMR for 4a:

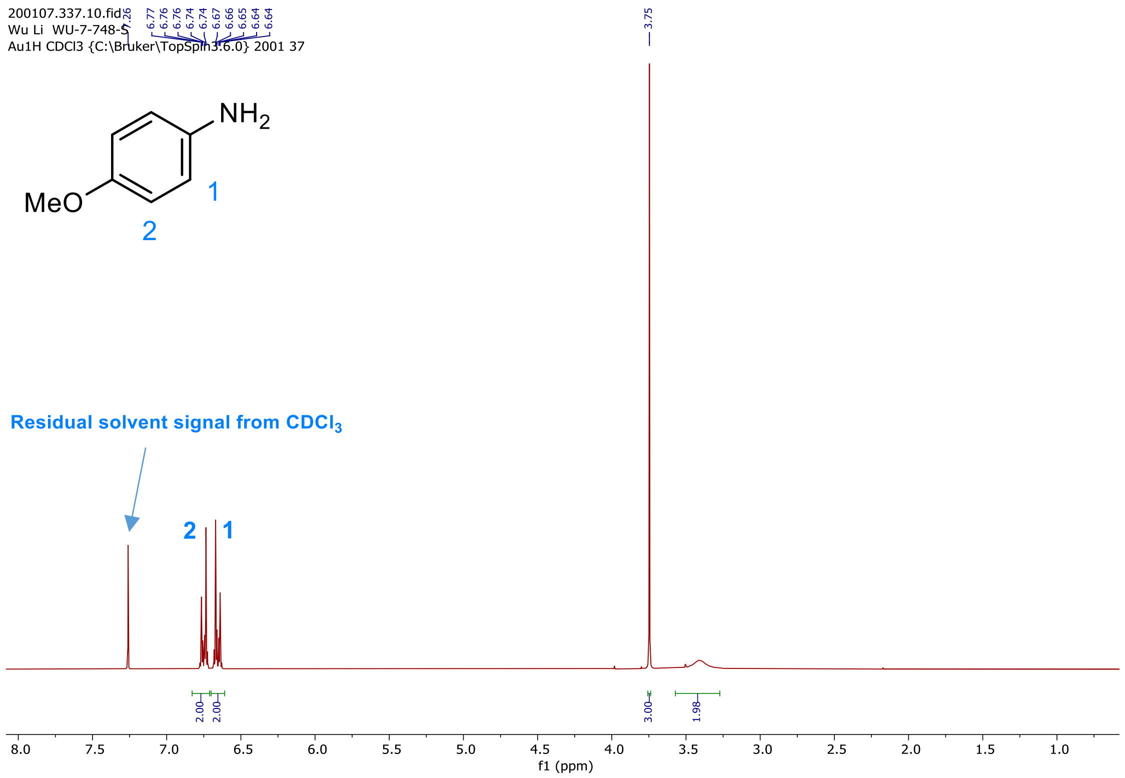

Original spectra for 4b:

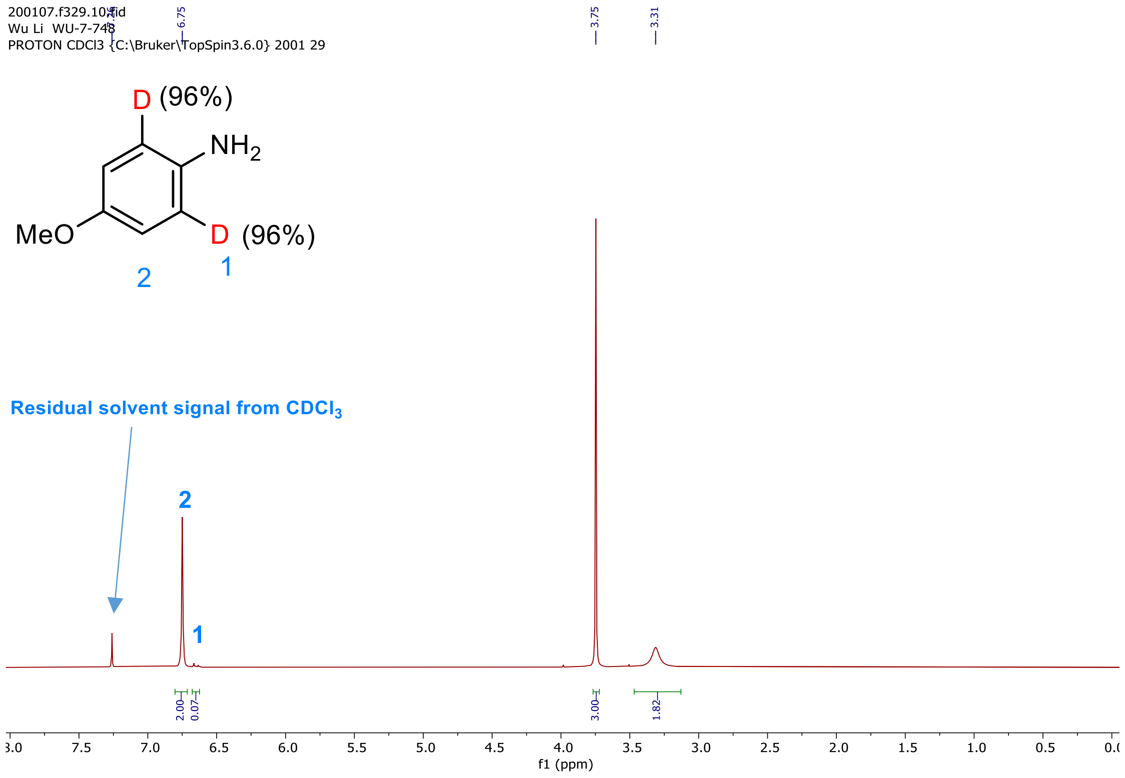

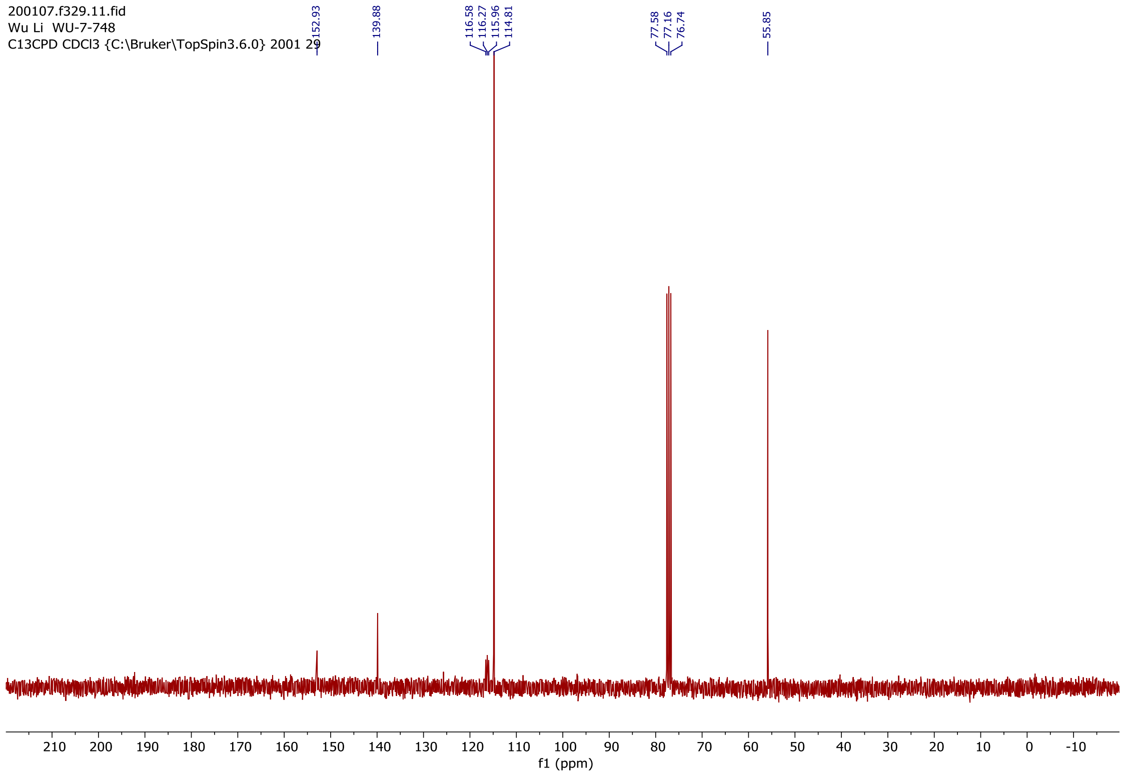
200107.f329.11.fid
Wu Li WU-7-748
C13CPD CDCl3 {C:\Bruker\TopSpin3.6.0} 2001 29
152.93
139.88
116.58
116.27
115.96
114.81
77.58
77.16
76.74
55.85
f1 (ppm)

^{1}H NMR for 5a:

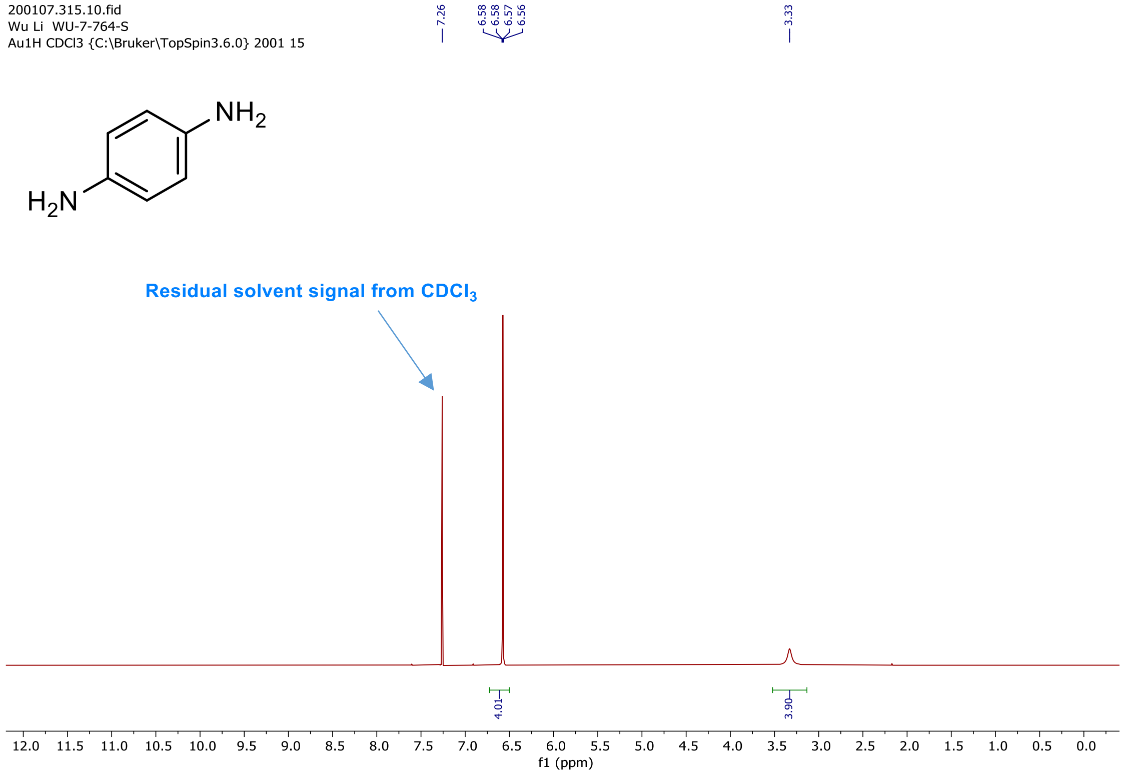
200107.315.10.fid
Wu Li WU-7-764-S
Au1H CDCl3 {C:\Bruker\TopSpin3.6.0} 2001 15
7.26
6.58
6.58
6.57
6.56
3.33
NH2
H2N
Residual solvent signal from CDCl3
4.01
3.90
f1 (ppm)

Original spectra for 5b:

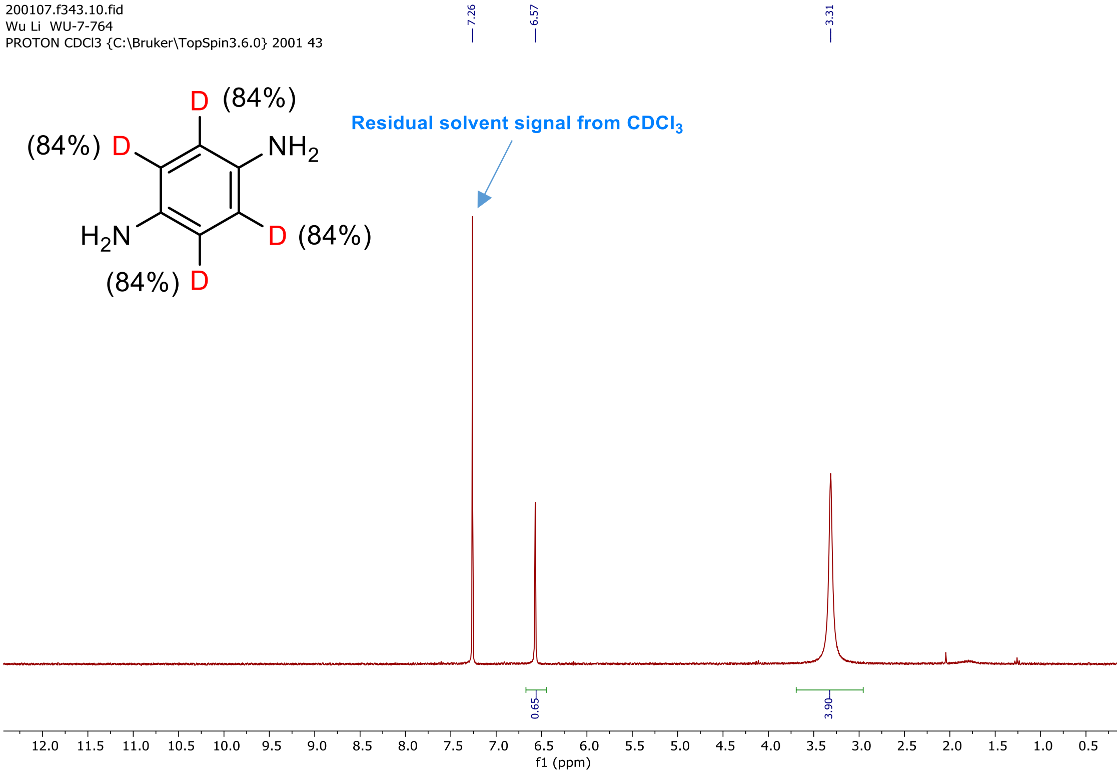

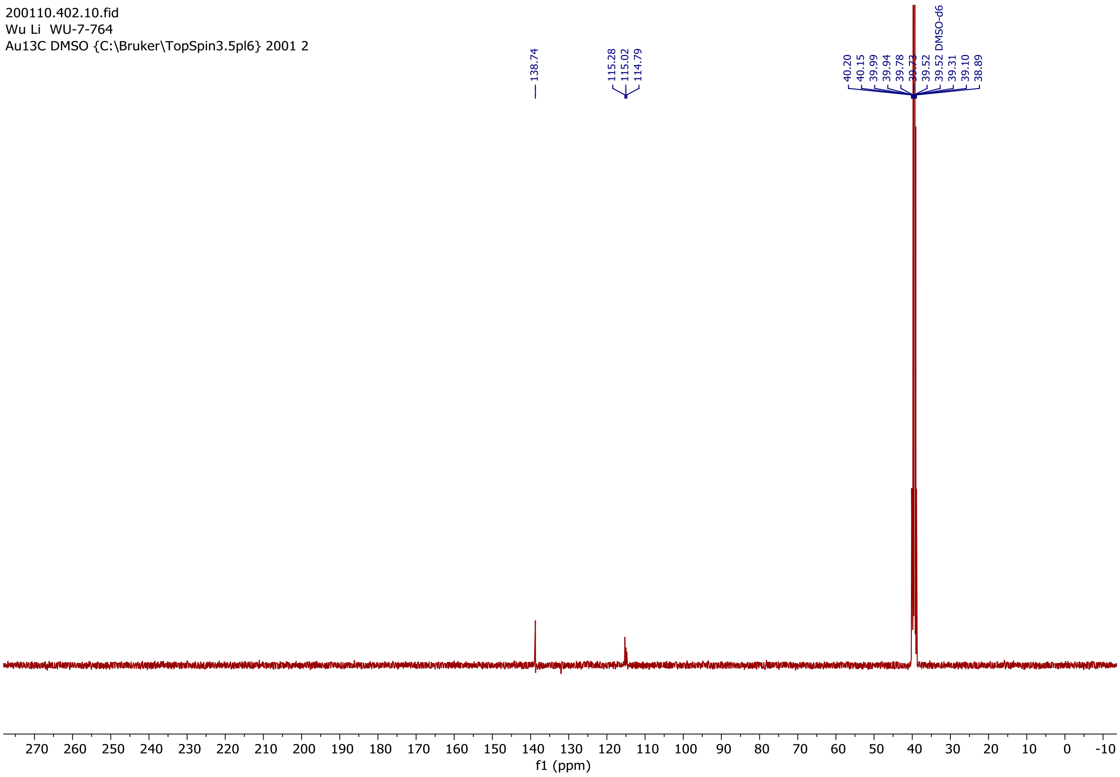

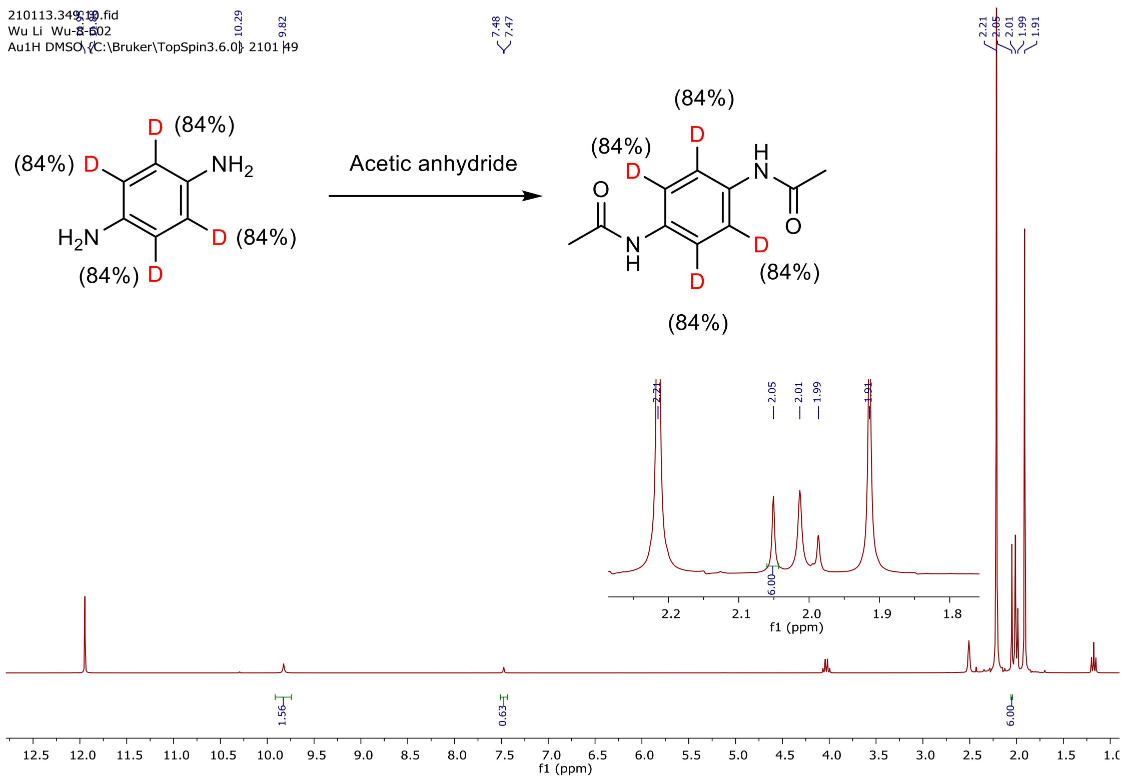
210113.349.10.fid
Wu Li Wu-8-602
Au1H DMSO {C:\Bruker\TopSpin3.6.0} 2101 49
Acetic anhydride
(84%)
D
NH2
H2N
O
H
N
f1 (ppm)
1.56
0.63
6.00

^{1}H NMR for 6a:

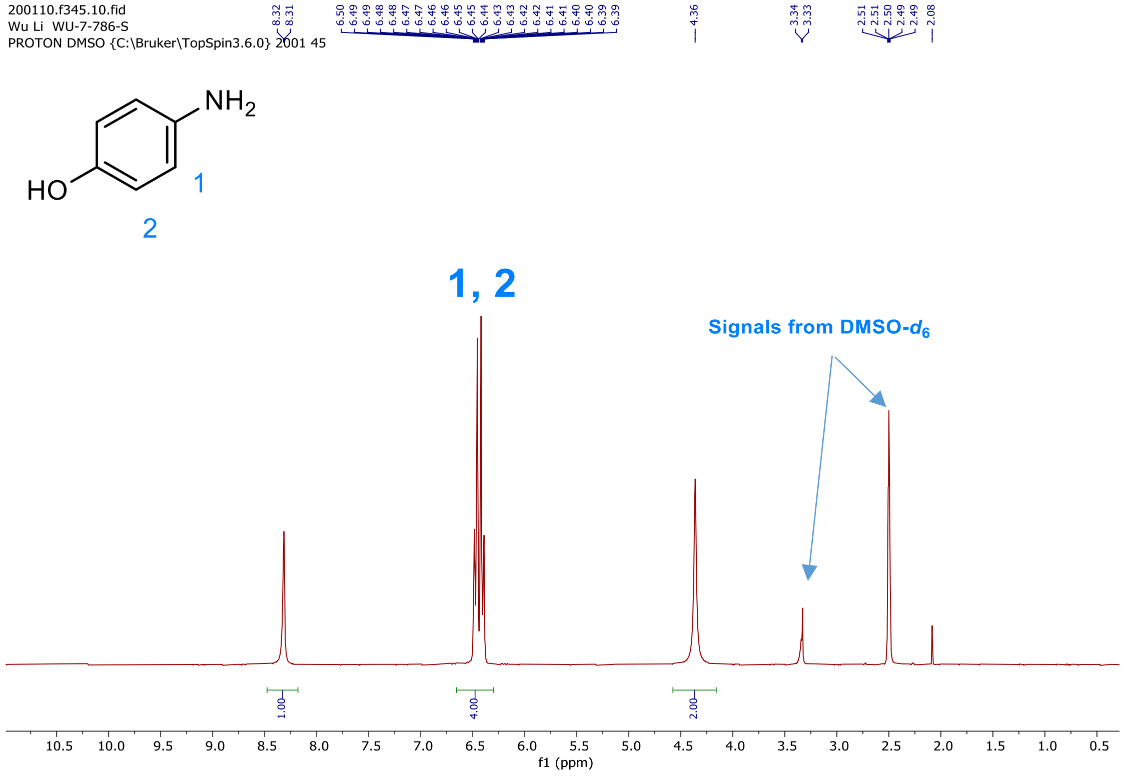
200110.f345.10.fid
Wu Li WU-7-786-S
PROTON DMSO {C:\Bruker\TopSpin3.6.0} 2001 45
NH2
HO
1
2
1, 2
Signals from DMSO-d6
1.00
4.00
2.00
f1 (ppm)

Original spectra for 6b:

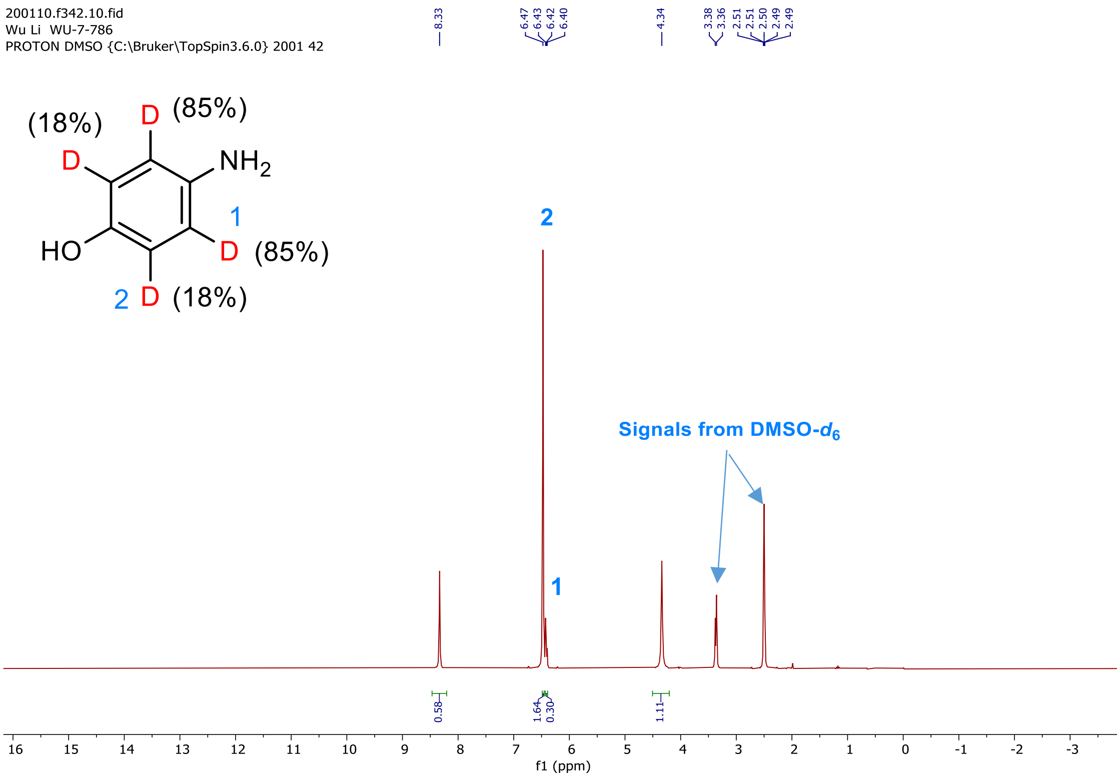

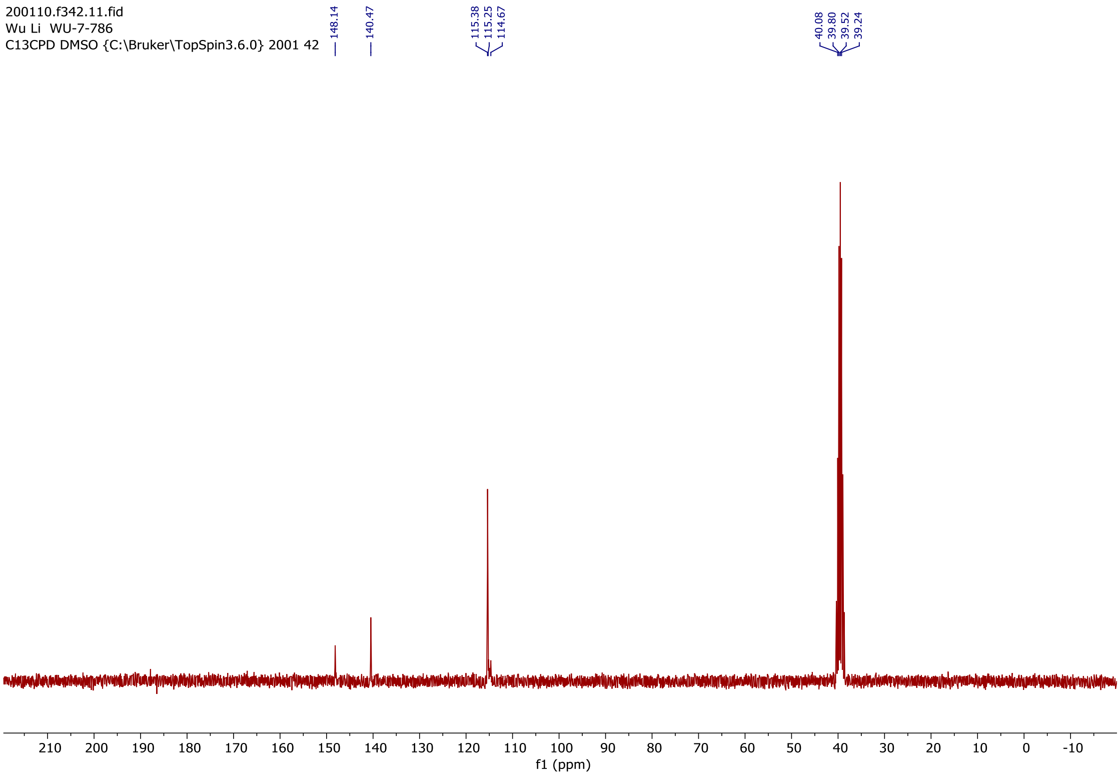

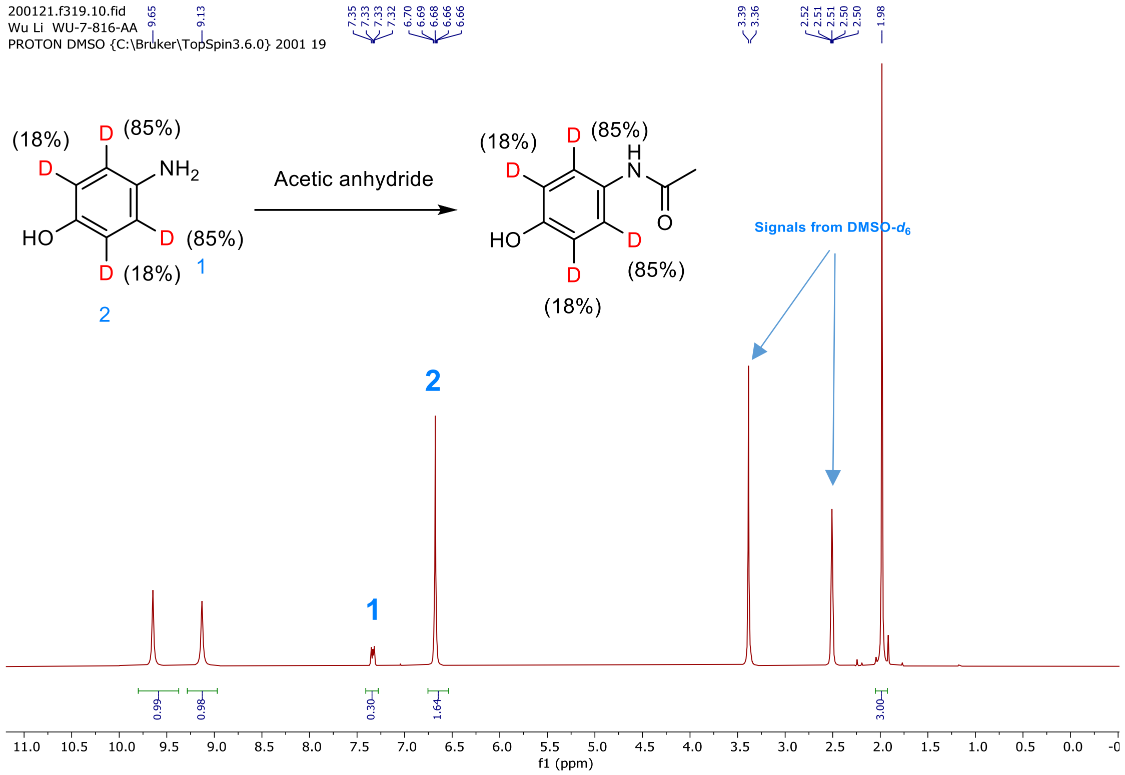
200121.f319.10.fid
Wu Li WU-7-816-AA
PROTON DMSO {C:\Bruker\TopSpin3.6.0} 2001 19
9.65
9.13
7.35
7.33
7.33
7.32
6.70
6.69
6.68
6.66
6.66
3.39
3.36
2.52
2.51
2.51
2.50
2.50
1.98
(18%)
(85%)
NH2
HO
(85%)
(18%)
1
2
Acetic anhydride
(18%)
(85%)
HO
(85%)
(18%)
Signals from DMSO-d6
2
1
0.99
0.98
0.30
1.64
3.00
f1 (ppm)

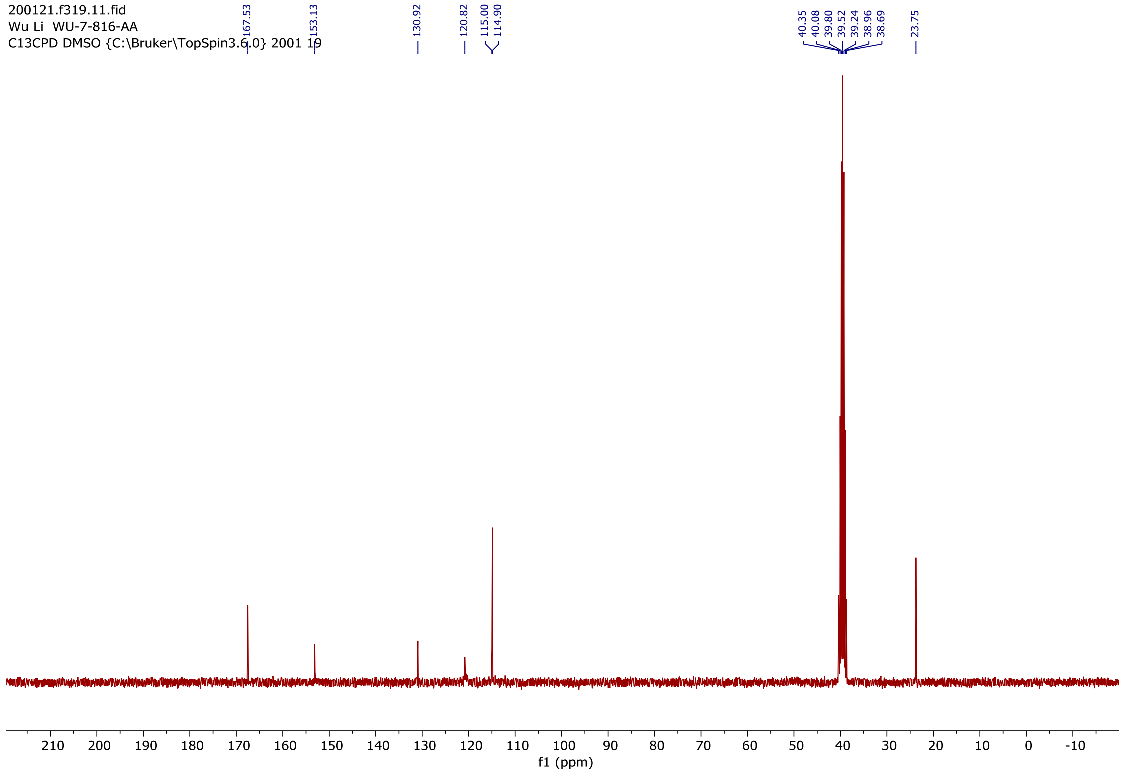
200121.f319.11.fid
Wu Li WU-7-816-AA
C13CPD DMSO {C:\Bruker\TopSpin3.6.0} 2001 19
167.53
153.13
130.92
120.82
115.00
114.90
40.35
40.08
39.80
39.52
39.24
38.96
38.69
23.75
f1 (ppm)

^{1}H NMR for 7a:

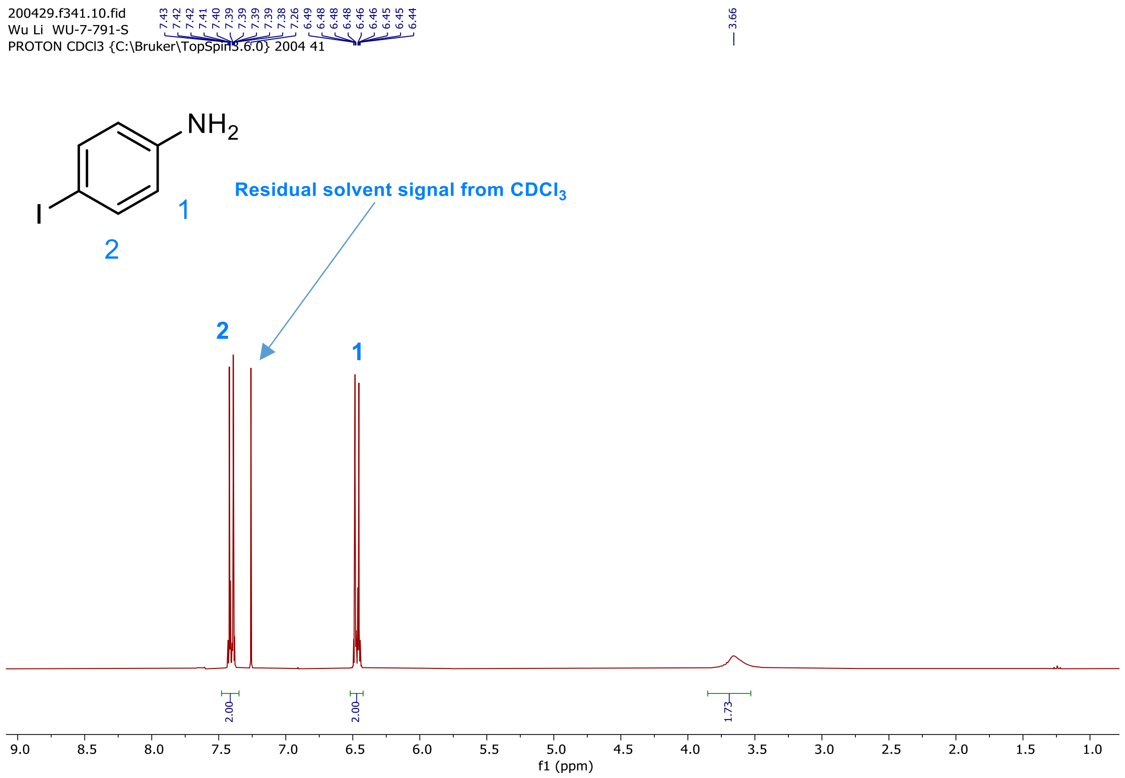

Original spectra for 7b:

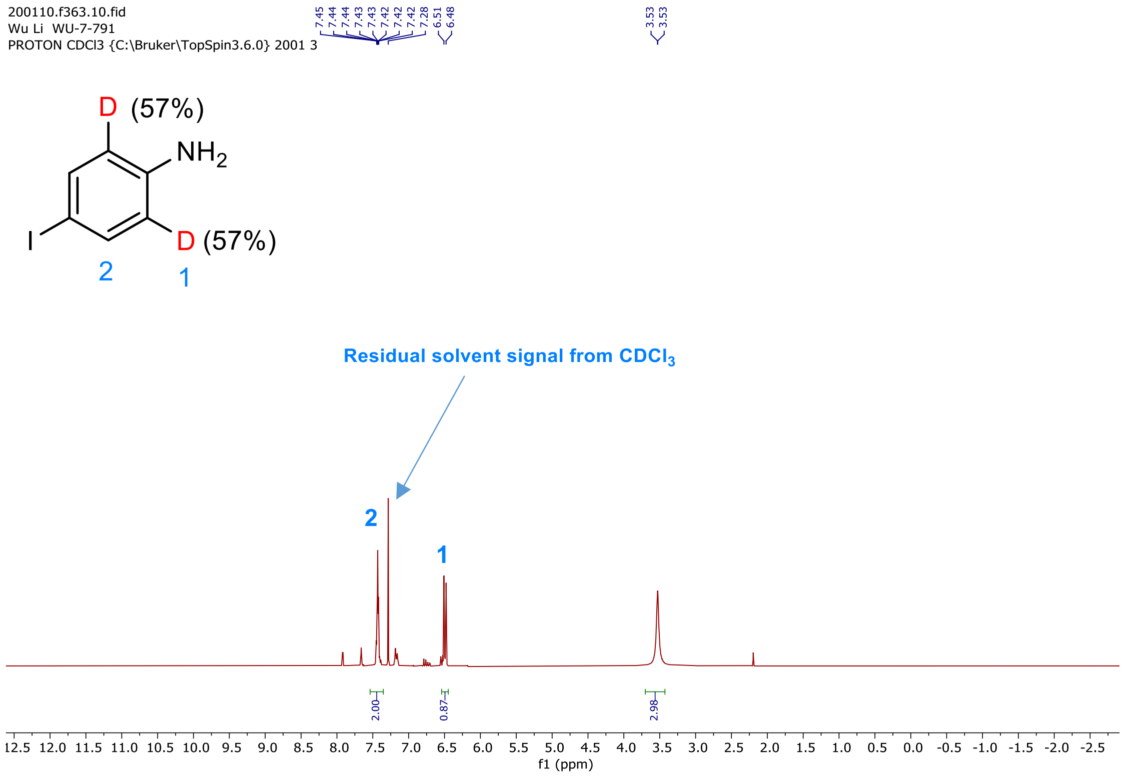

200110.f363.11.fid
Wu Li WU-7-791
C13CPD CDCl3 {C:\Bruker\TopSpin3.6.0} 2001 3

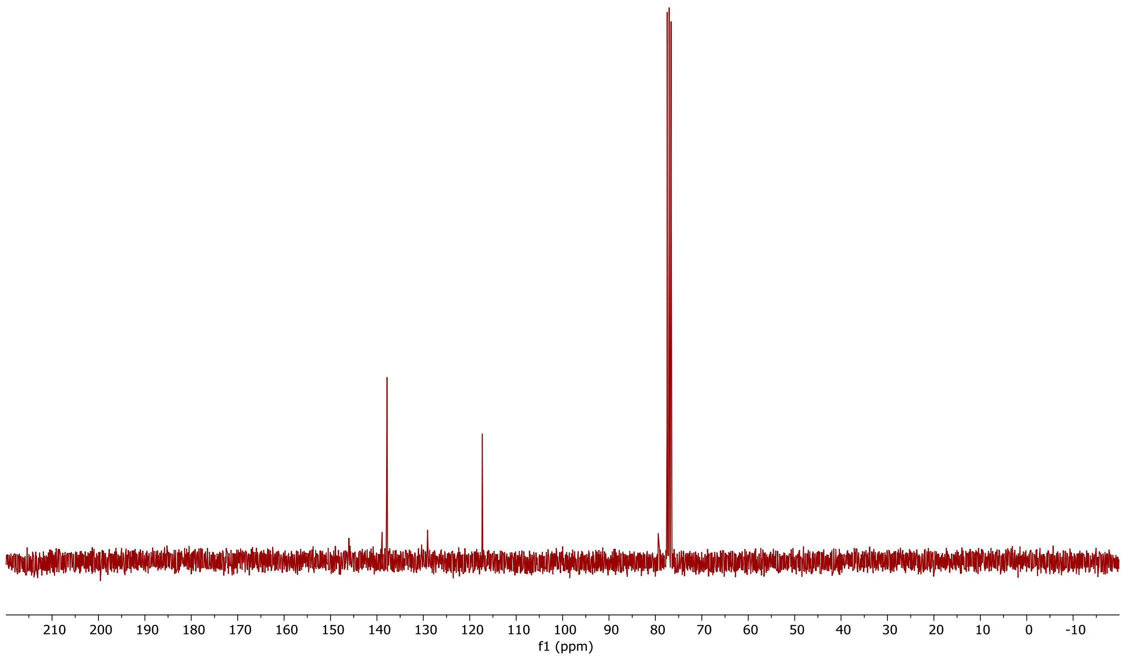

^{1}H NMR for 8a:

200120.332.10.fid
Wu Li, wu-7-845-S
Au1H CDCl3 {C:\Bruker\TopSpin3.6.0} 2001 32

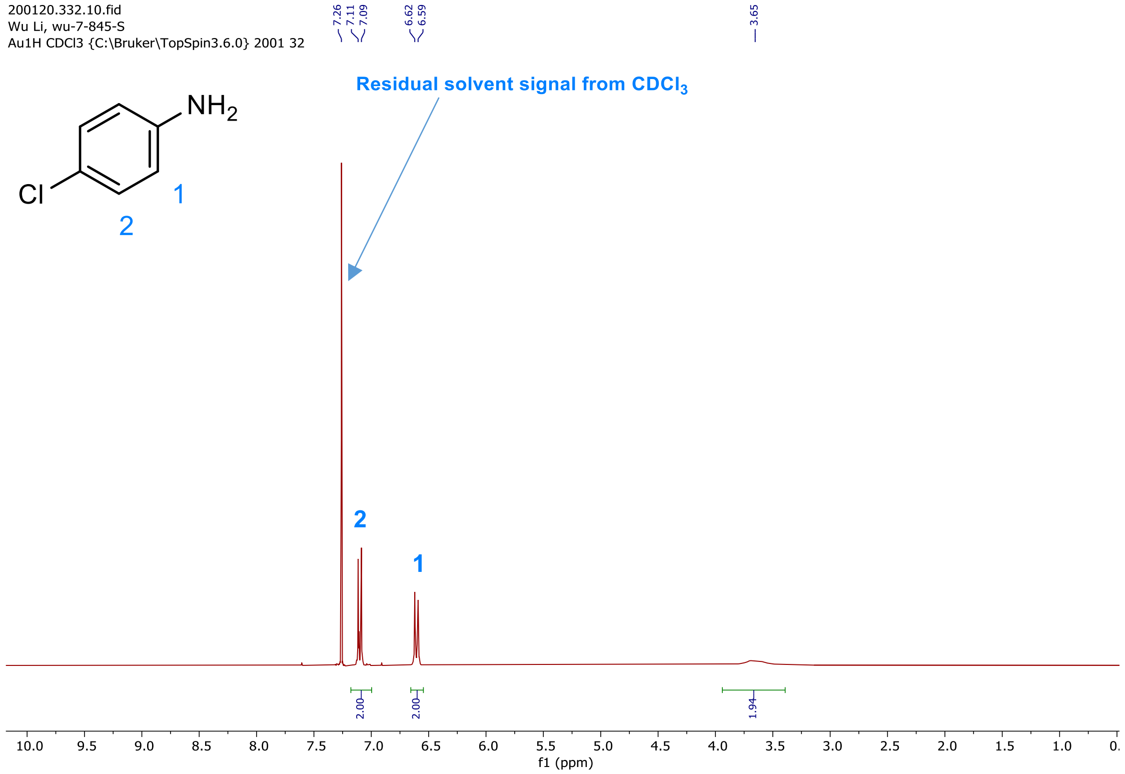

Original spectra for 8b:

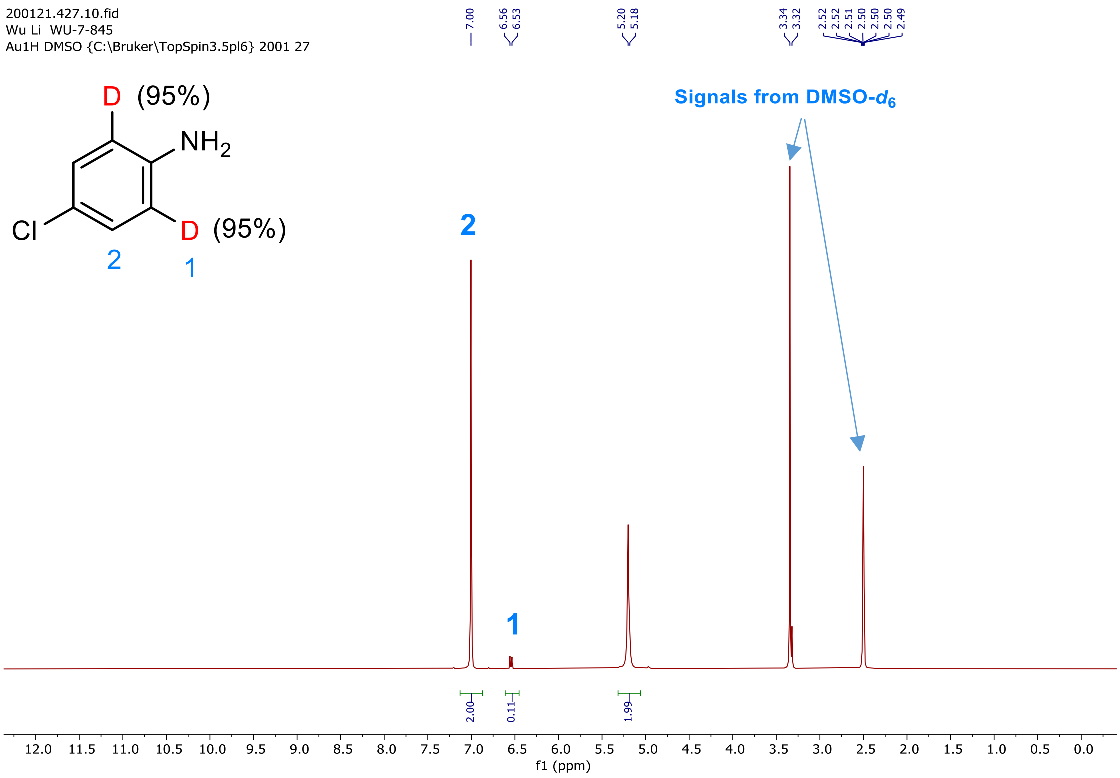

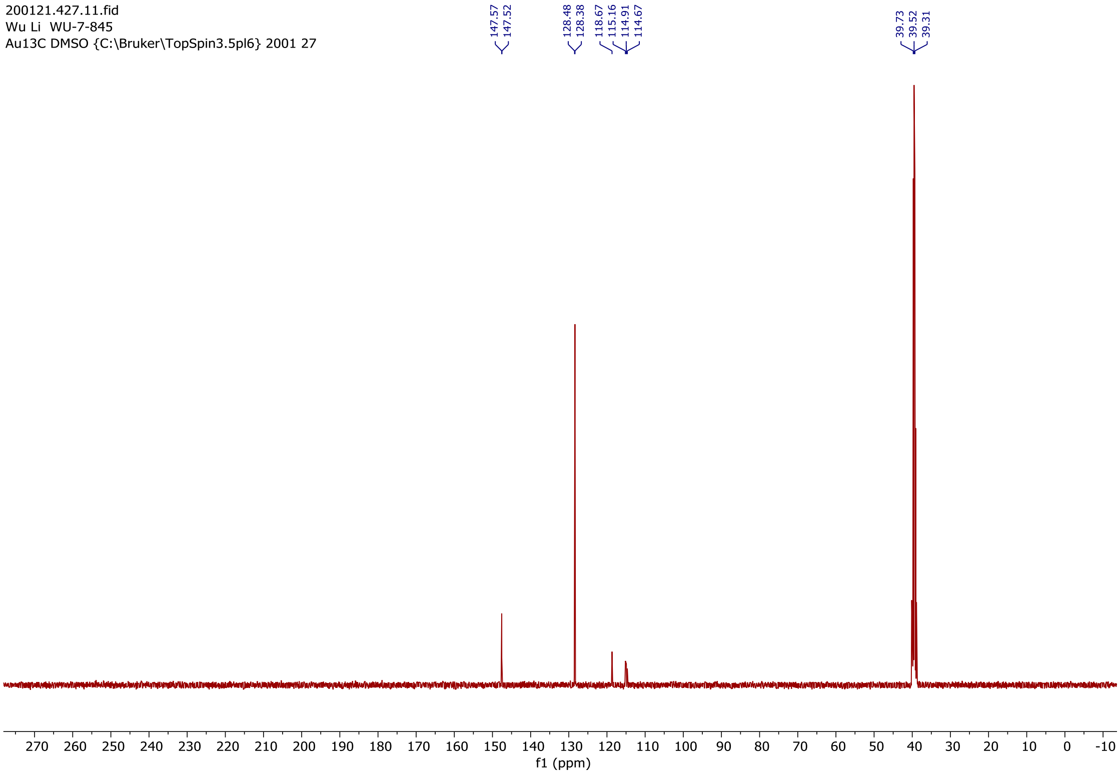

^{1}H NMR for 9a:

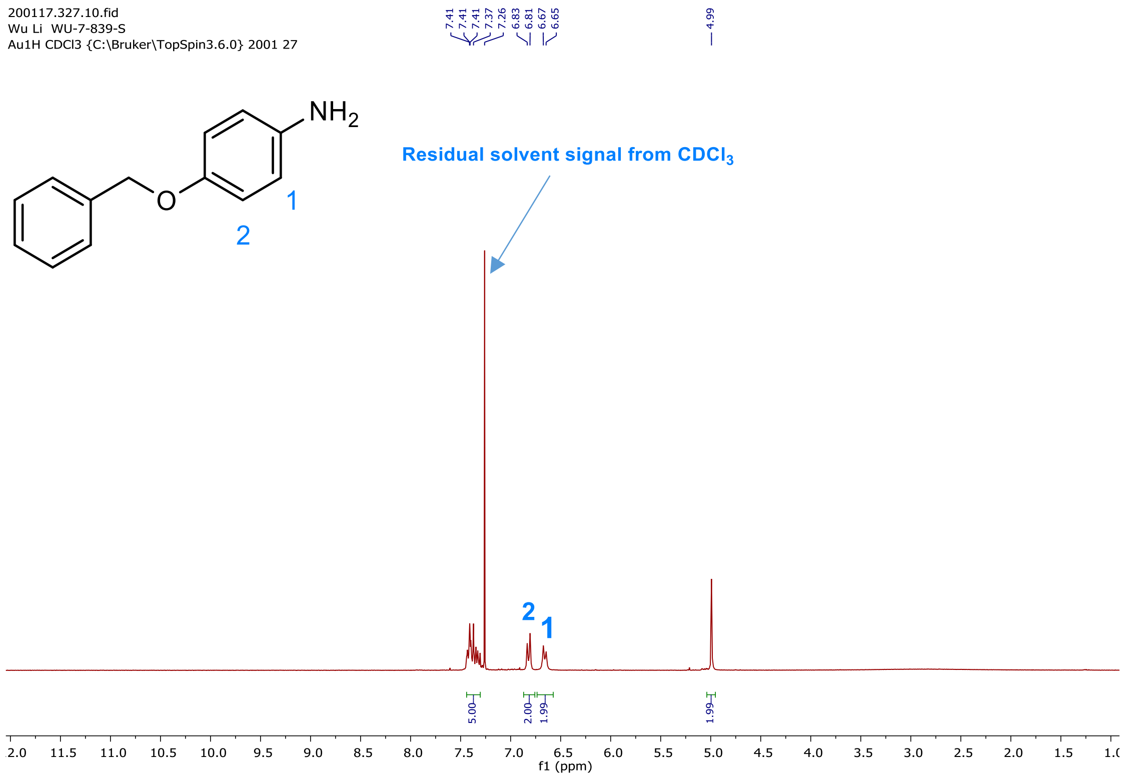

Original spectra for 9b:

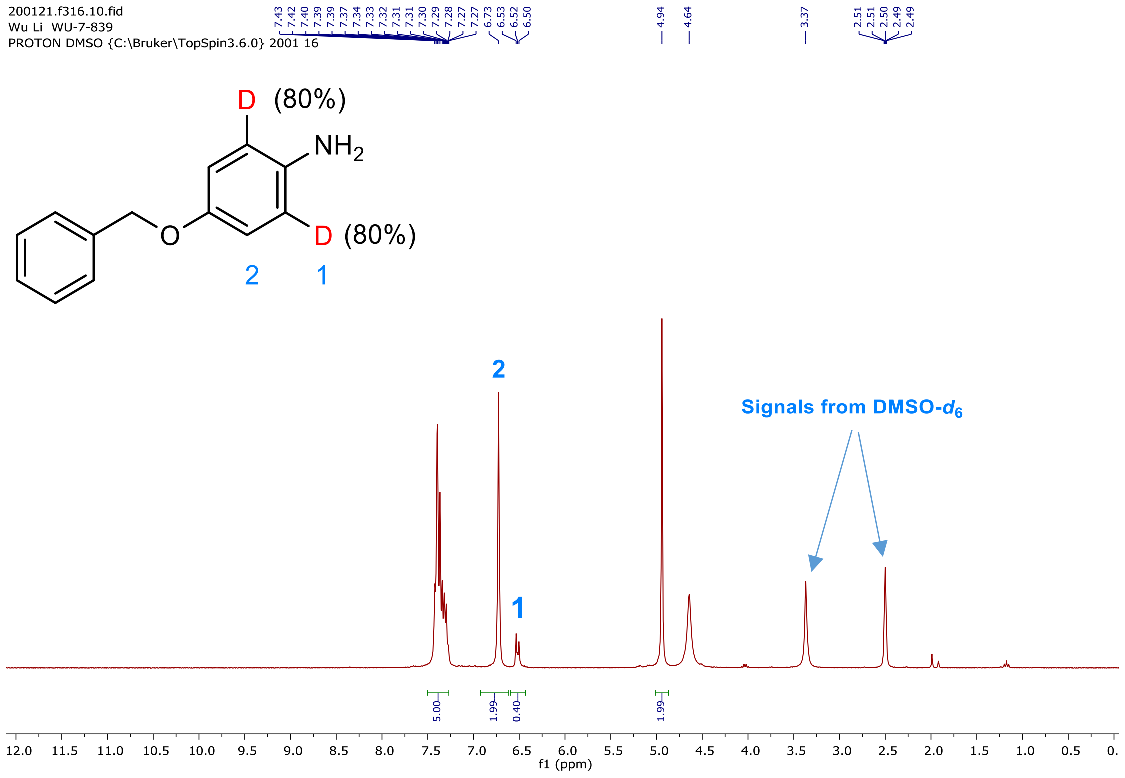

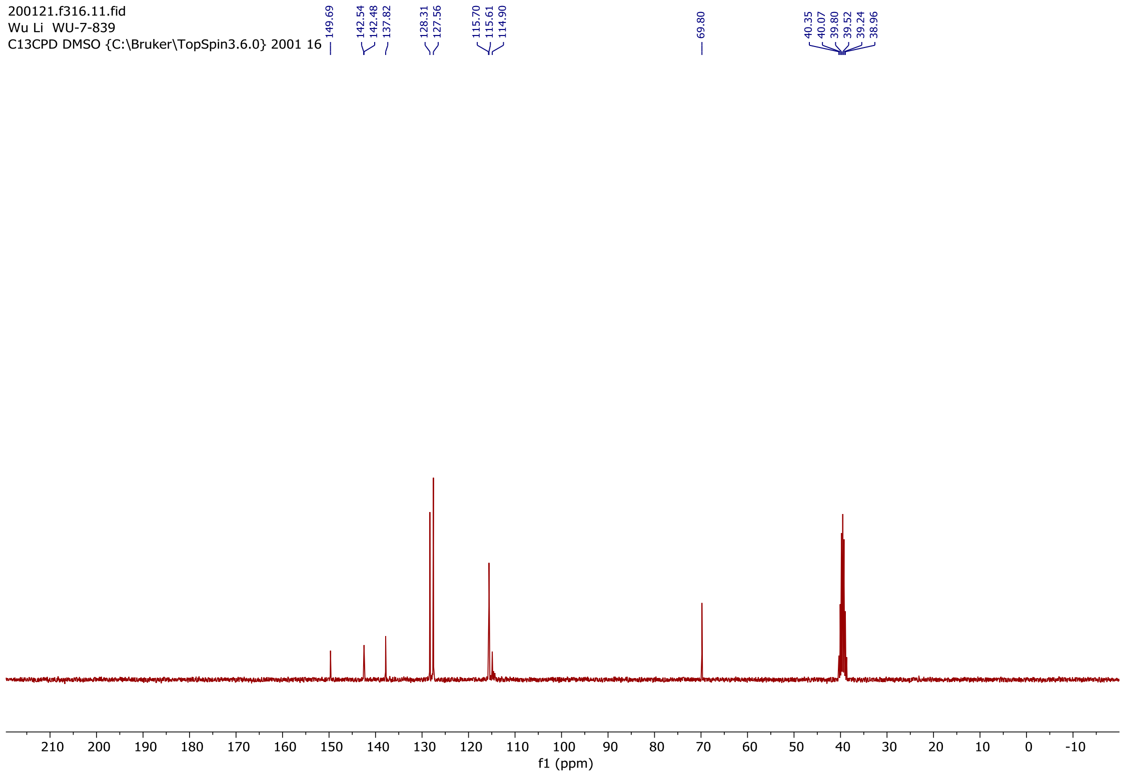

^{1}H NMR for 10a:

In $CDCl_3$

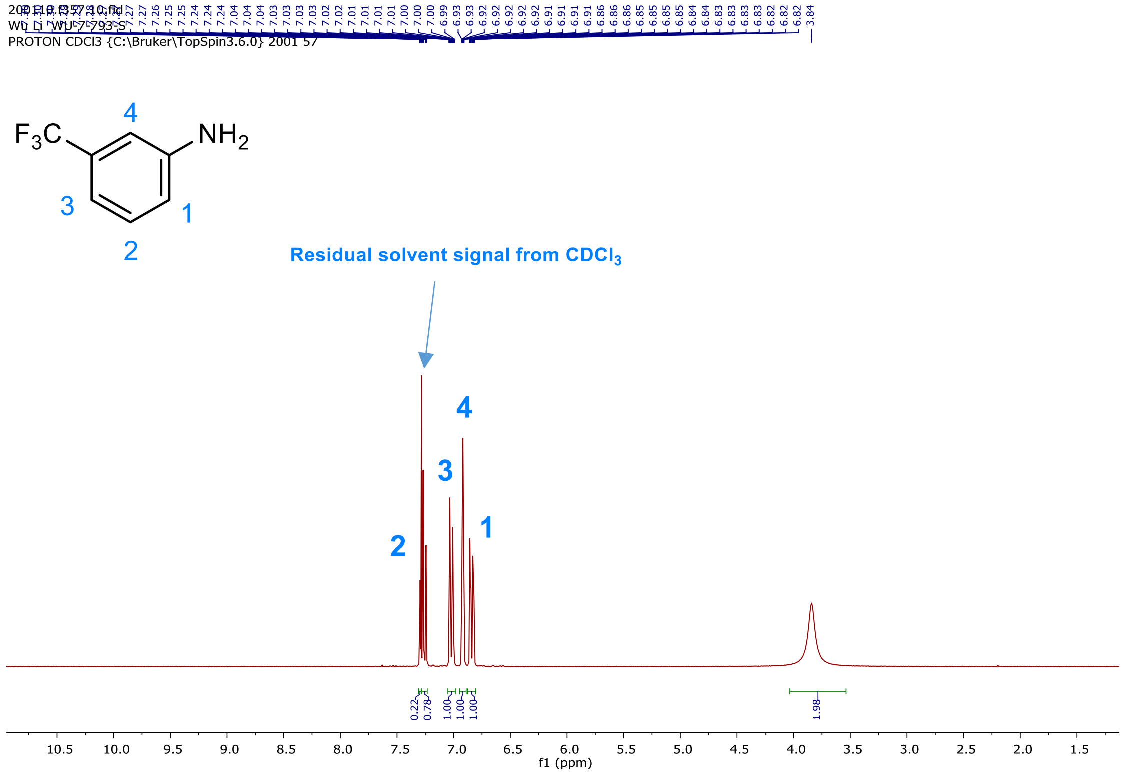

In DMSO-d_6:

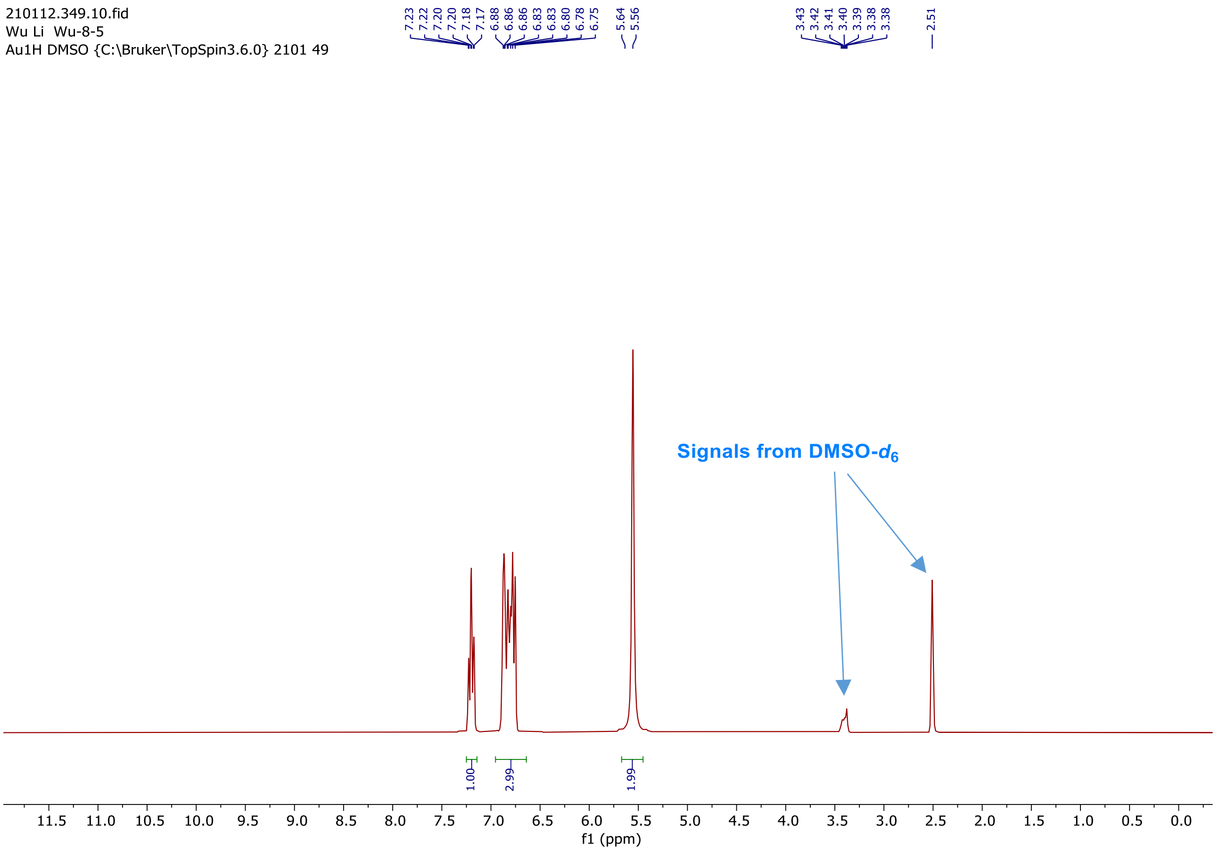

Original spectra for 10b:

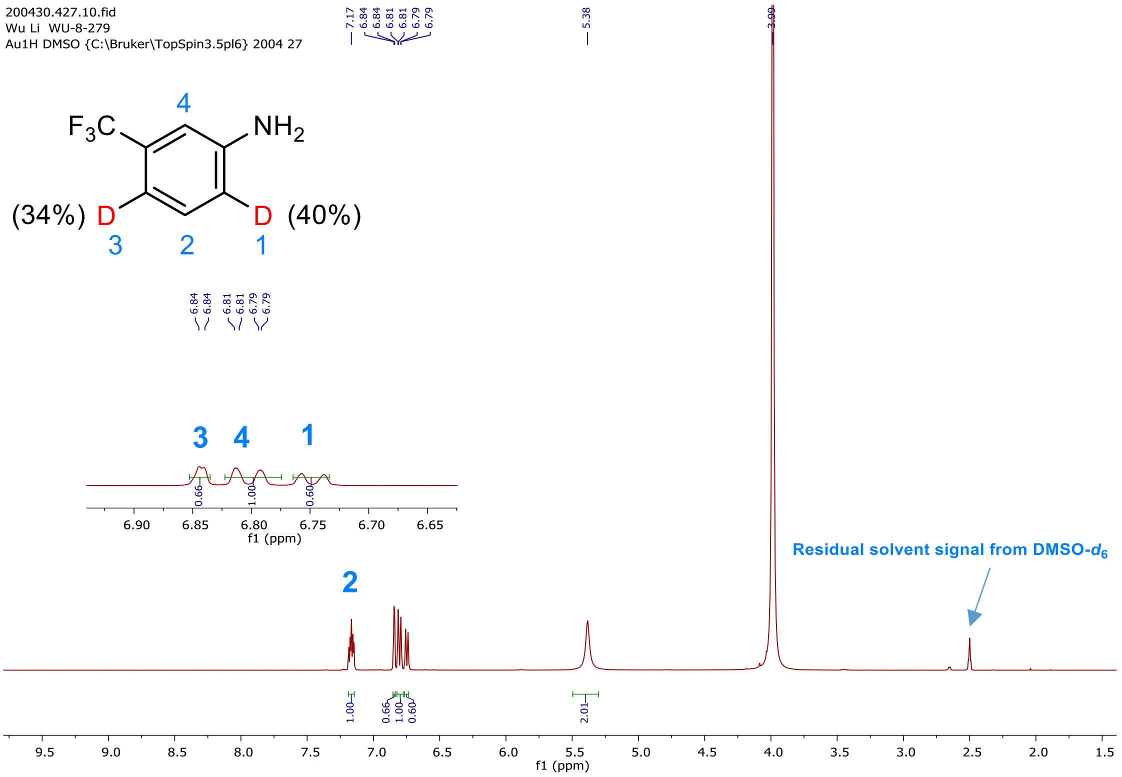

200430.427.11.fid
Wu Li WU-8-279
Au13C DMSO {C:\Bruker\TopSpin3.5pl6} 2004 27

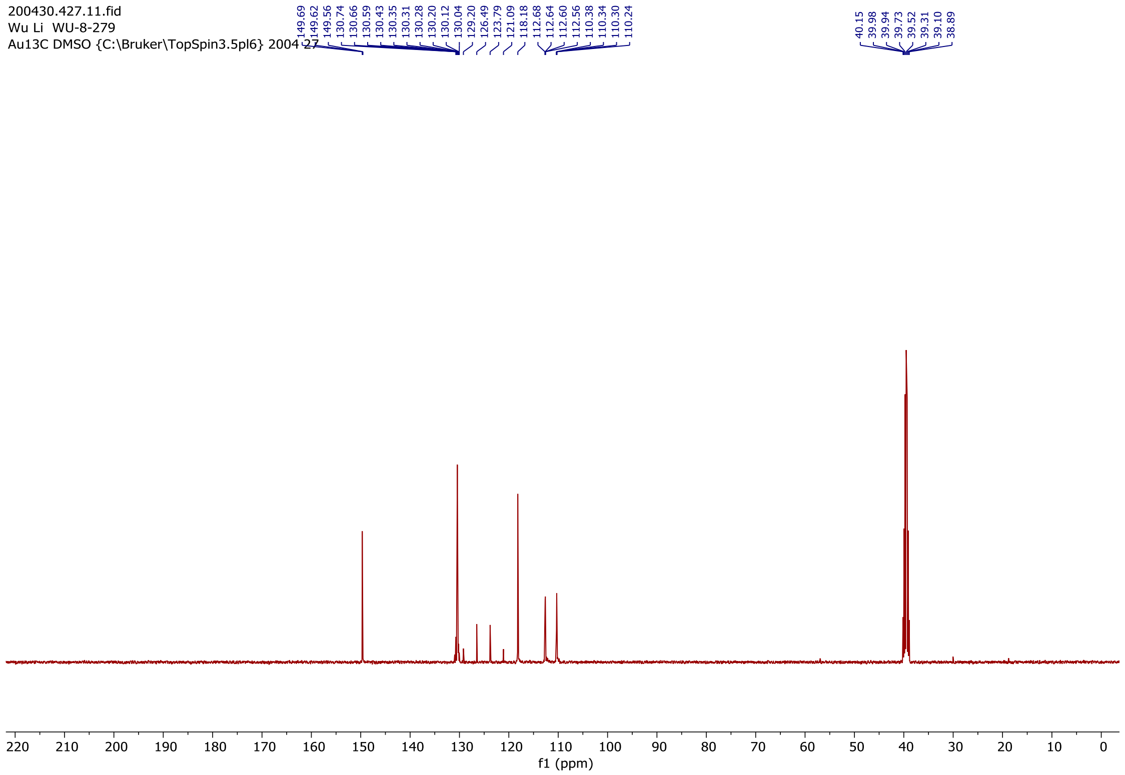

200428.f332.12.fid
Wu Li WU-8-279
19F(H-entk) DMSO {C:\Bruker\TopSpin3.6.0} 2004 32

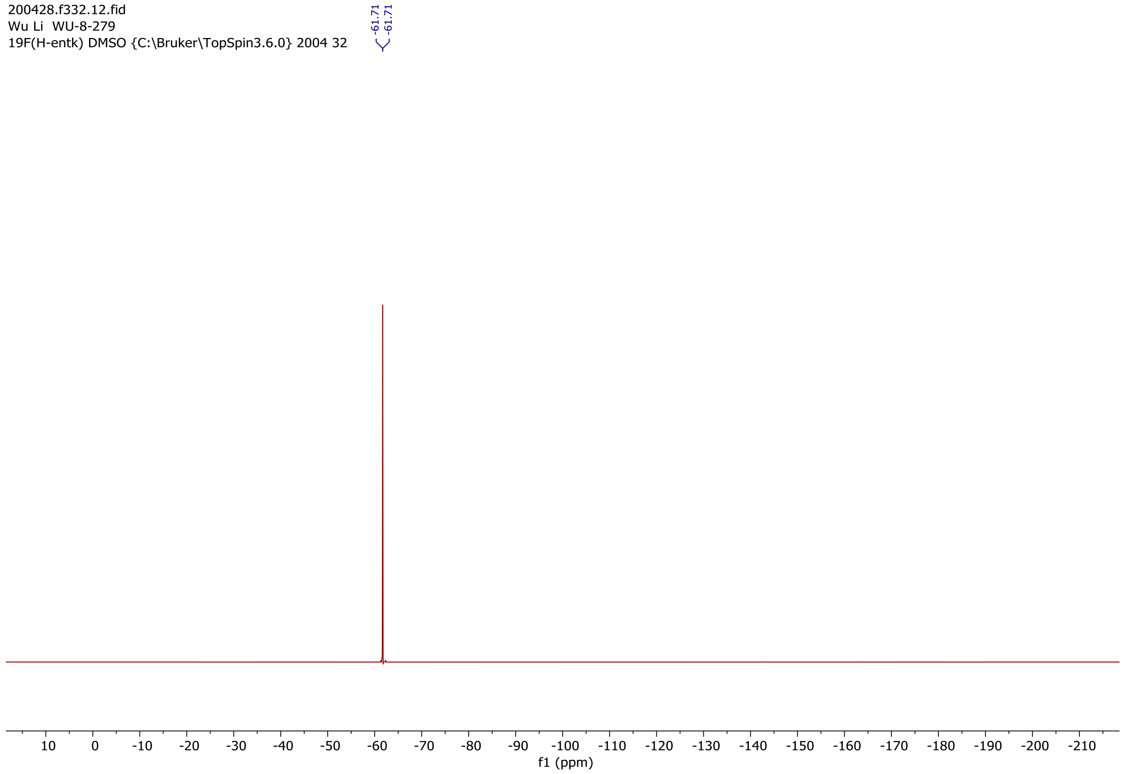

^{1}H NMR for 11a:

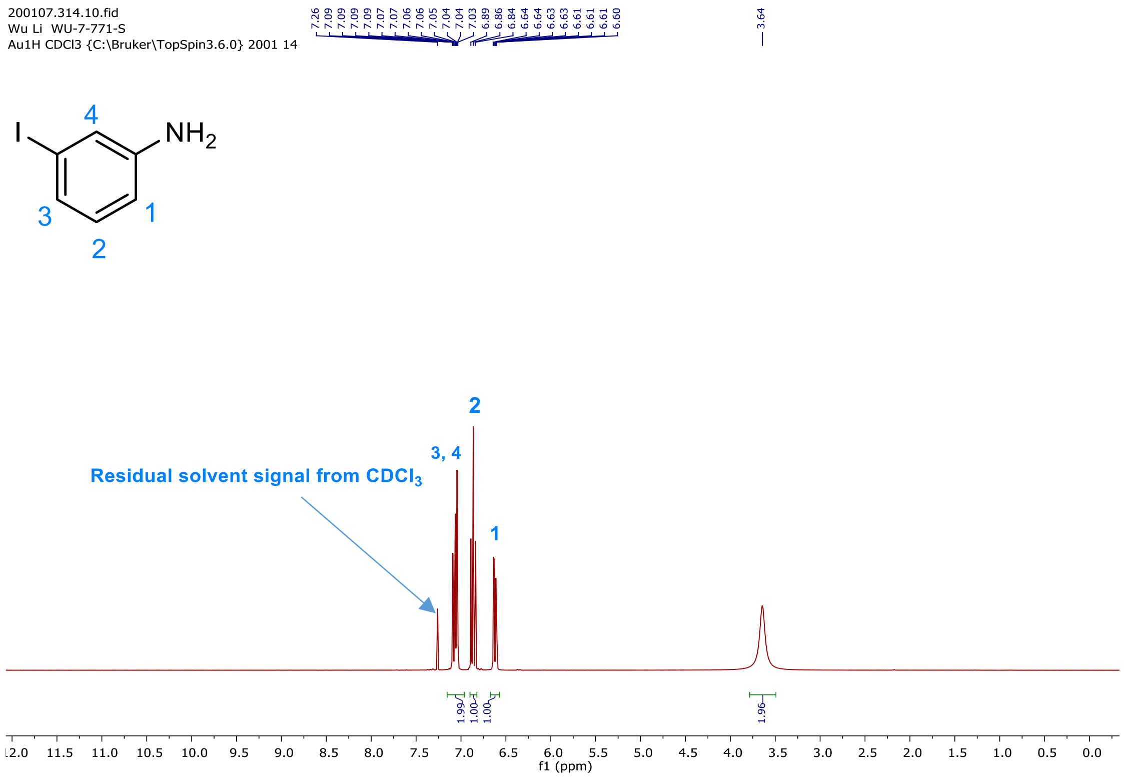

Original spectra for 11b:

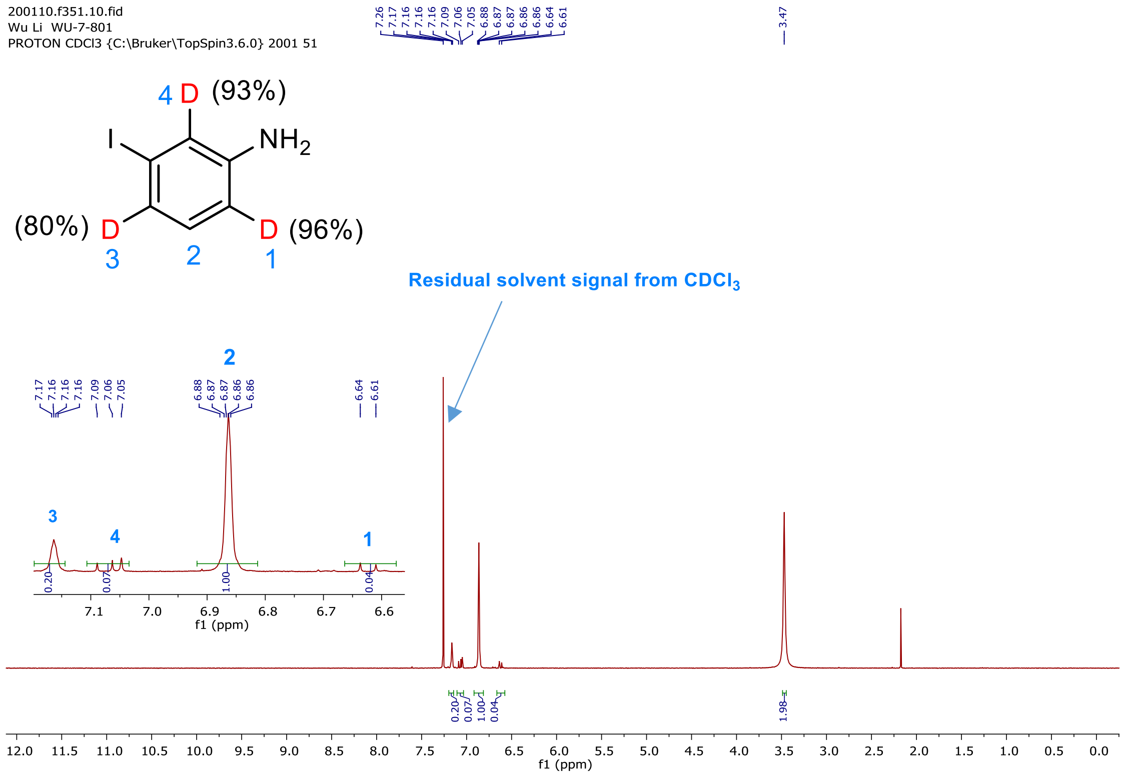

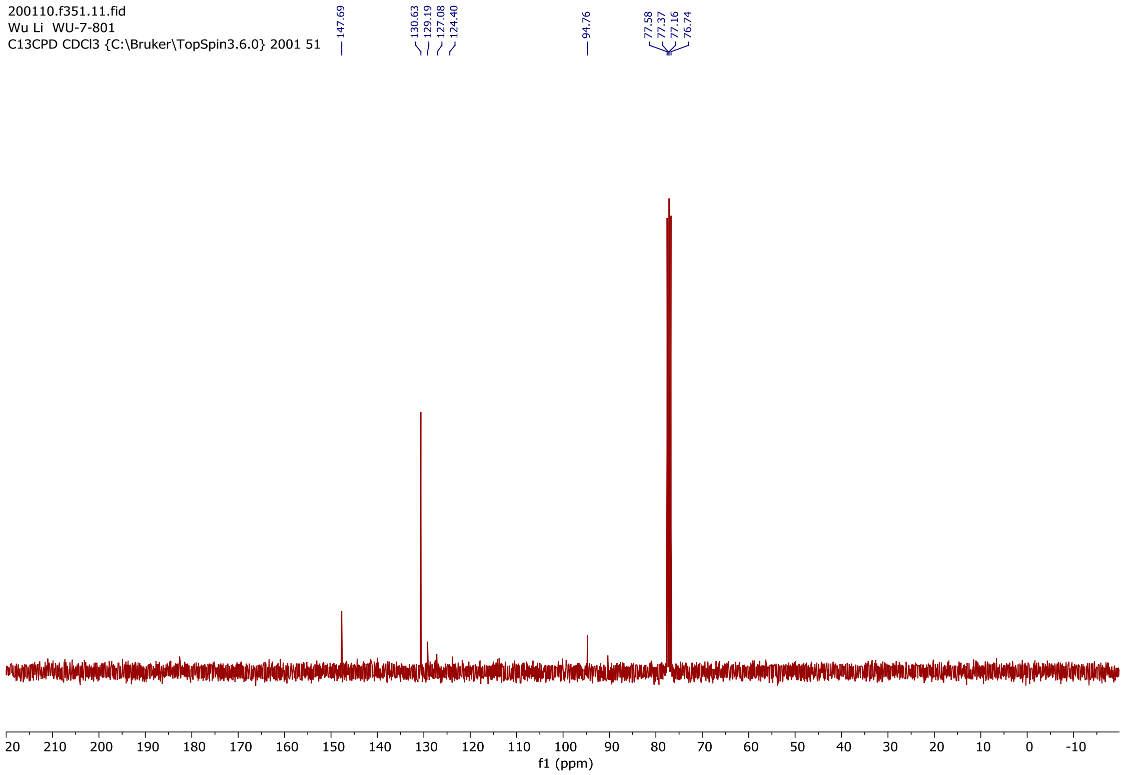

^{1}H NMR for 12a:

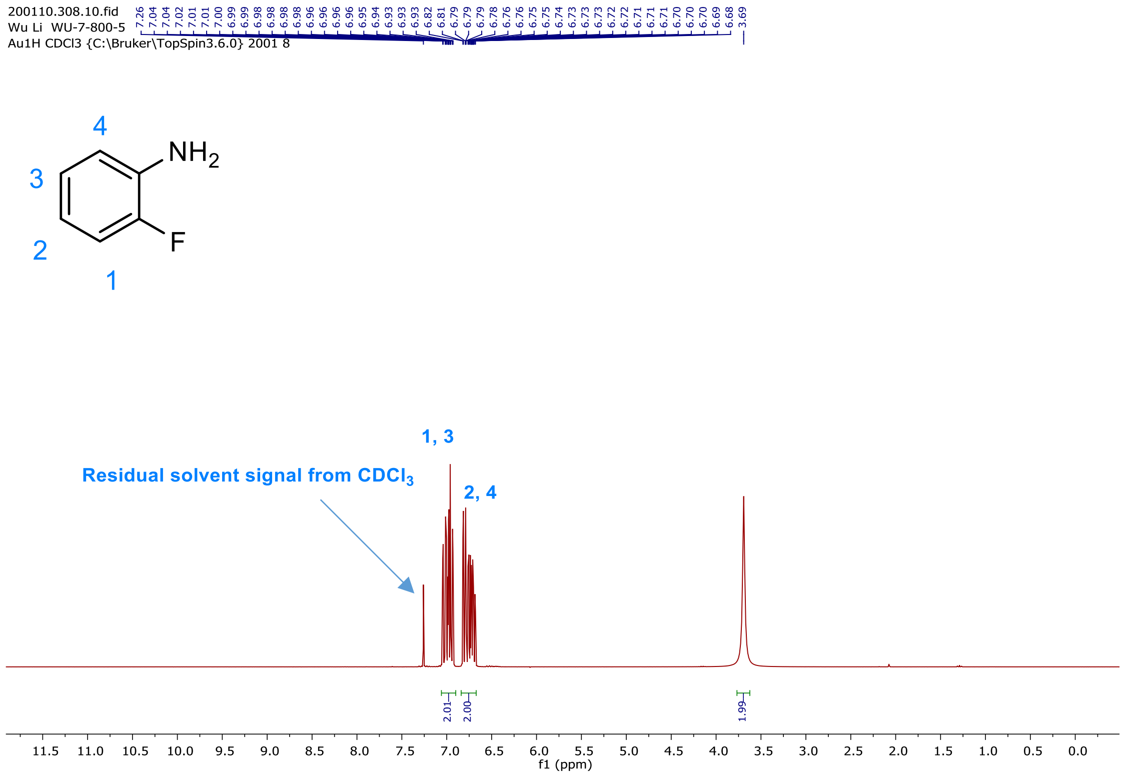

Original spectra for 12b:

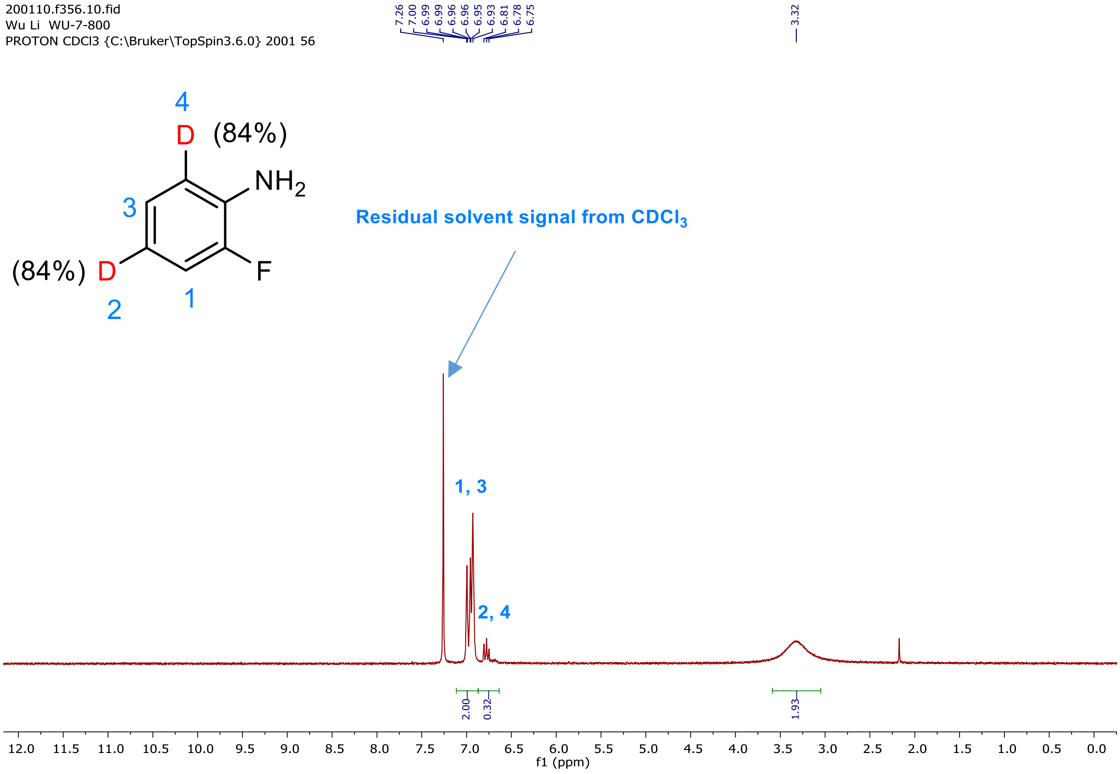

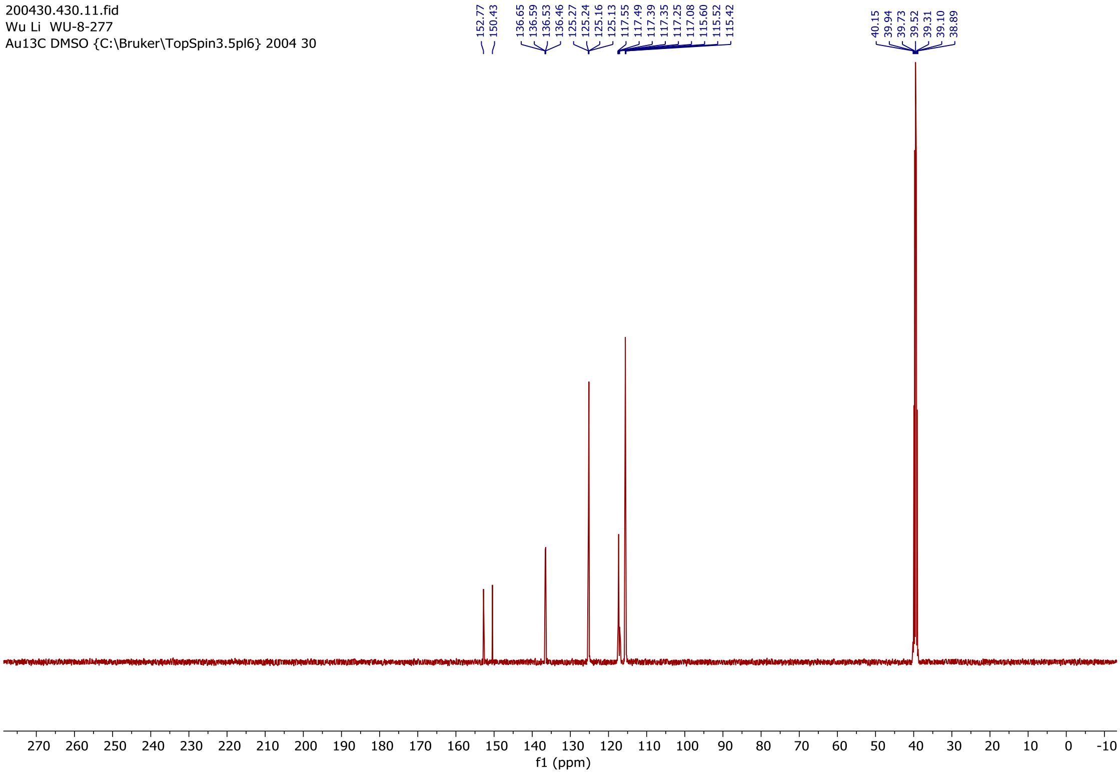

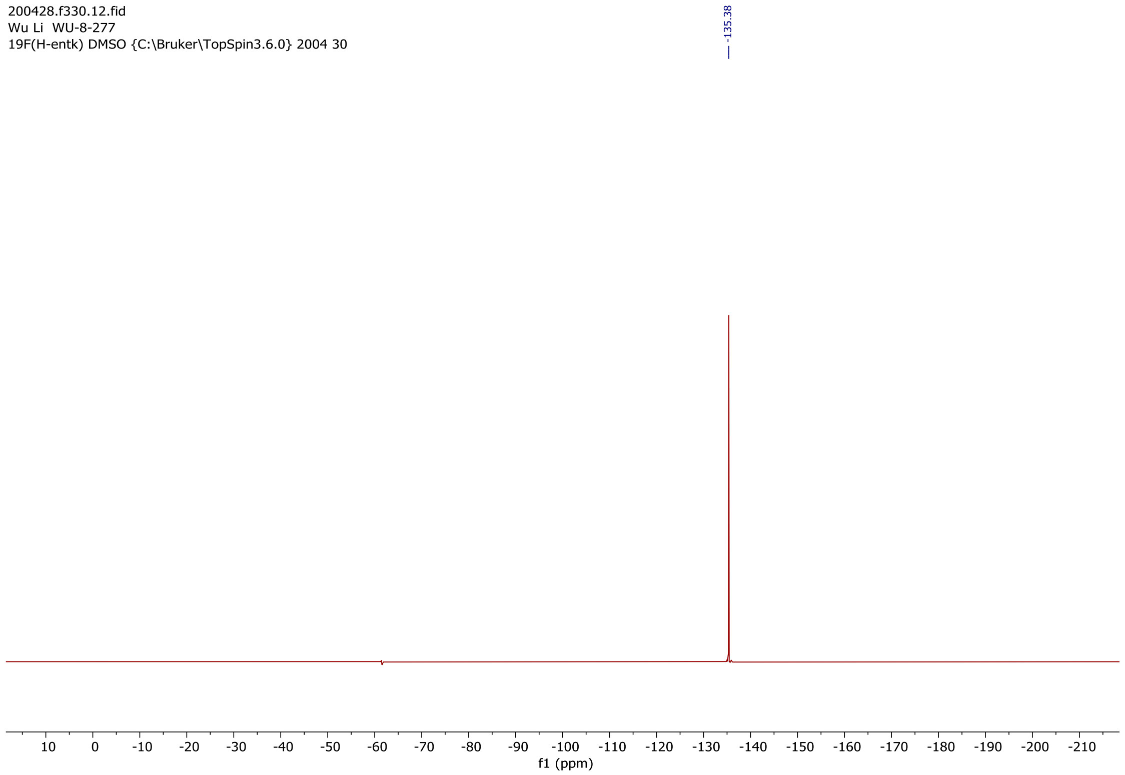
200428.f330.12.fid
Wu Li WU-8-277
19F(H-entk) DMSO {C:\Bruker\TopSpin3.6.0} 2004 30
-135.38
f1 (ppm)

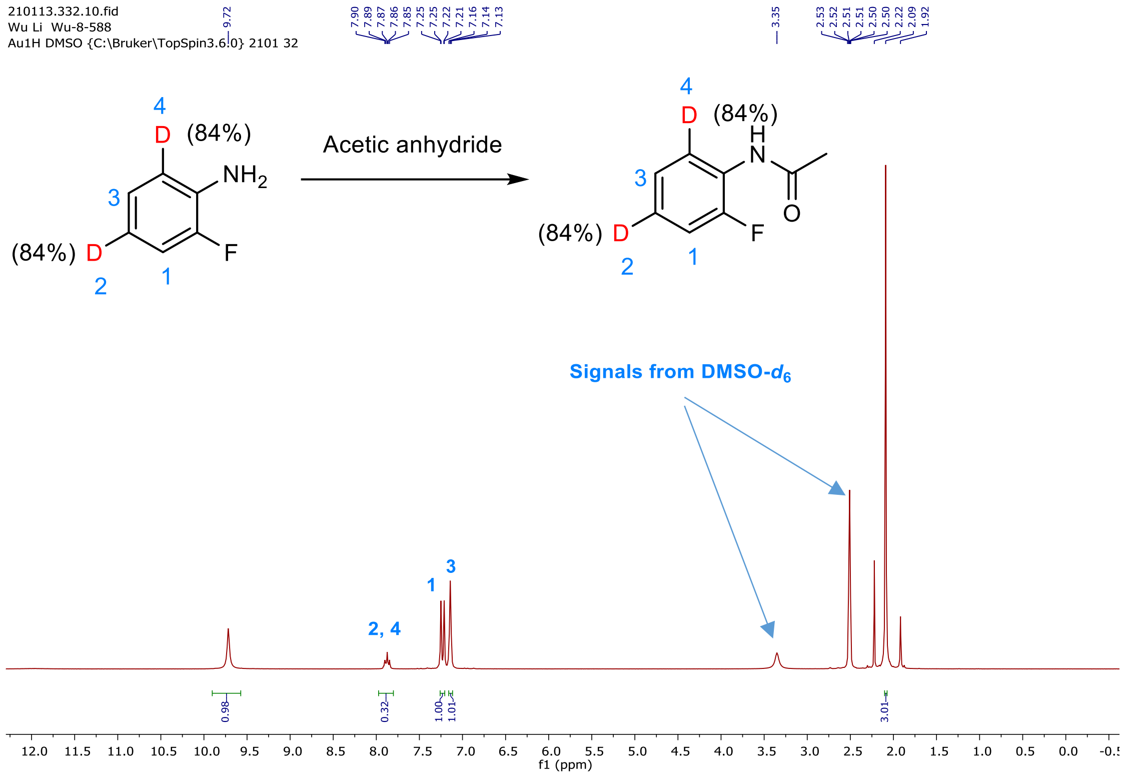
210113.332.10.fid
Wu Li Wu-8-588
Au1H DMSO {C:\Bruker\TopSpin3.6.0} 2101 32
Acetic anhydride
(84%)
NH2
F
Signals from DMSO-d6
f1 (ppm)

^{1}H NMR for 13a:

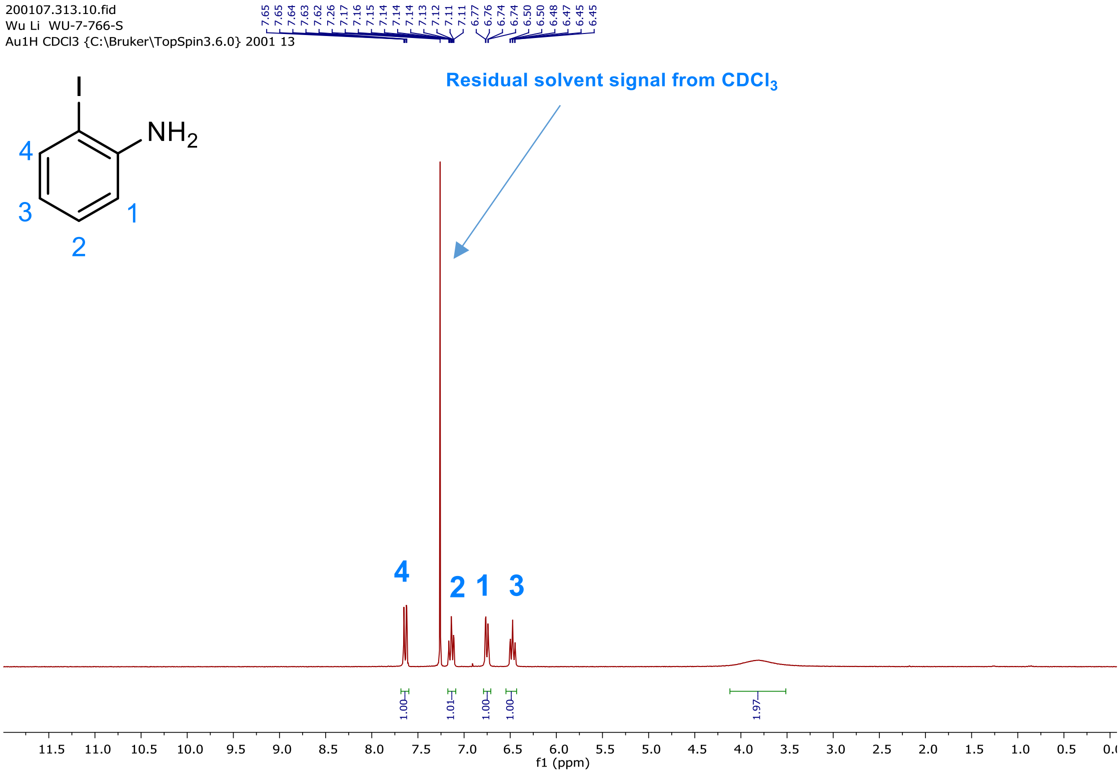

Original spectra for 13b:

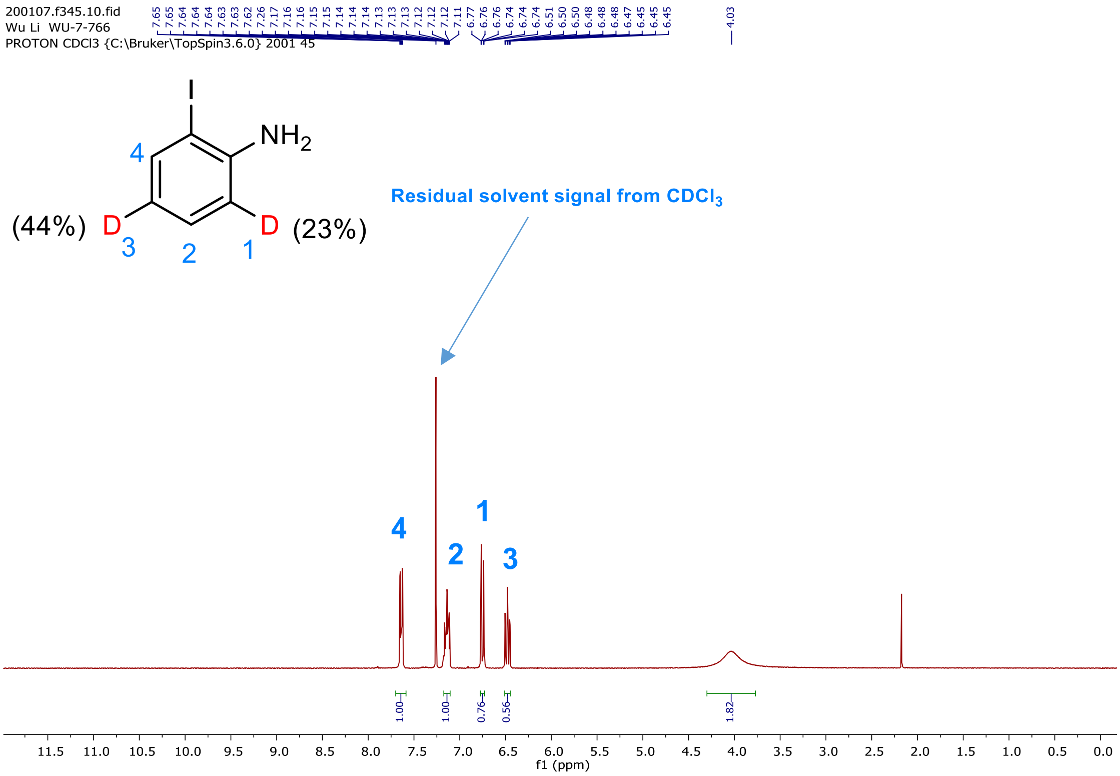

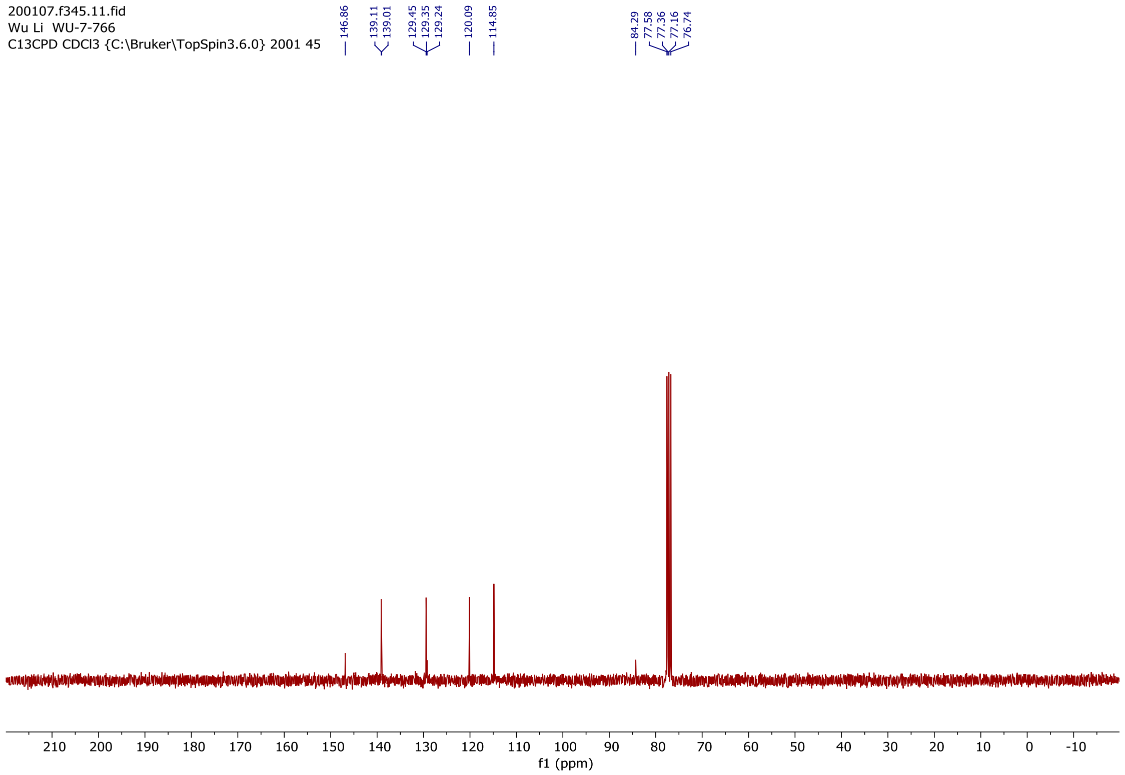

^{1}H NMR for 14a:

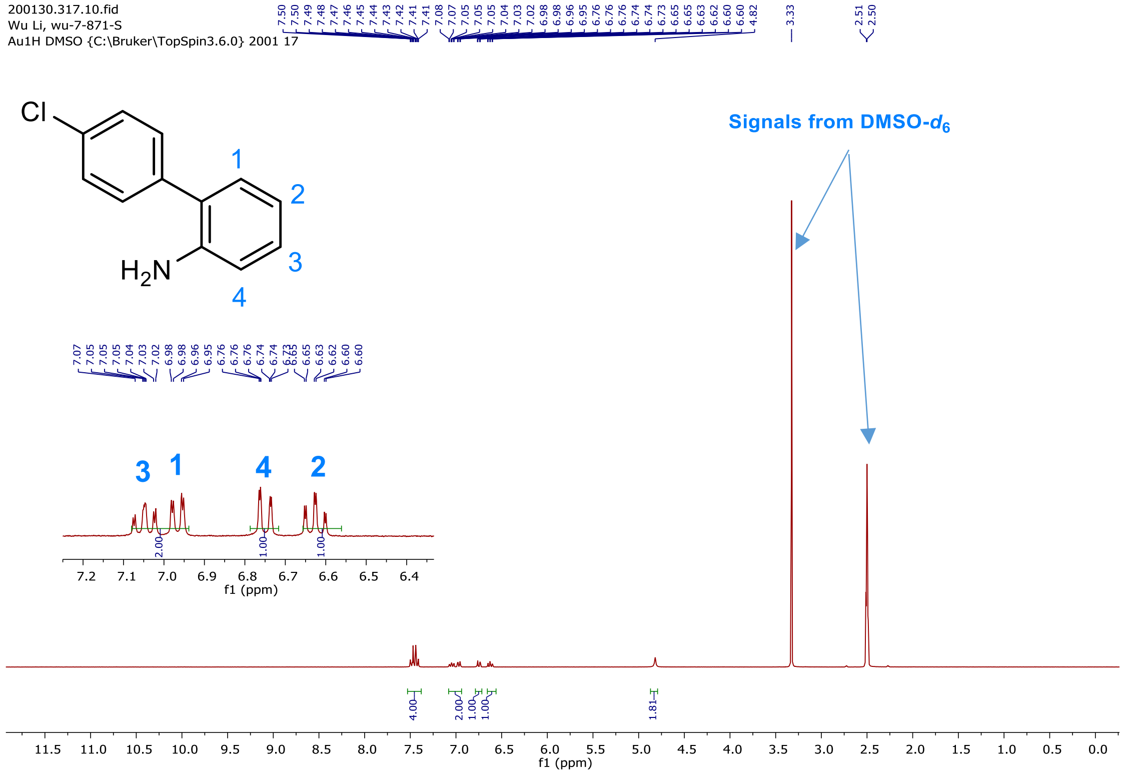

Original spectra for 14b:

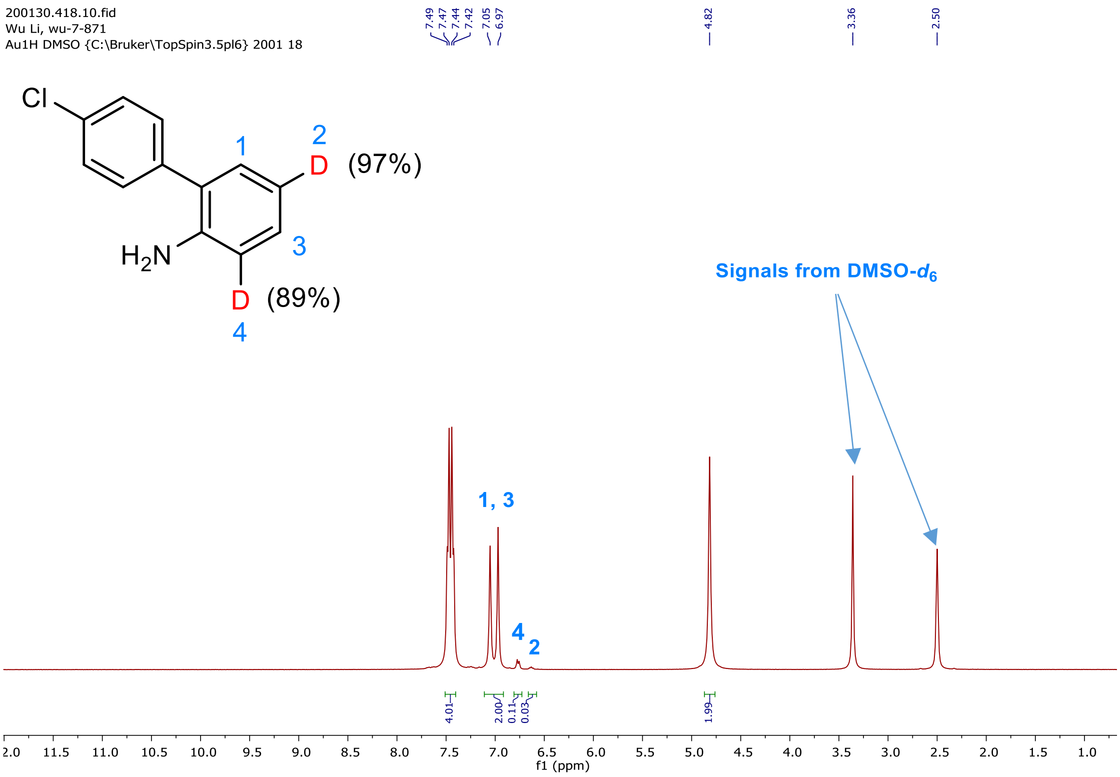

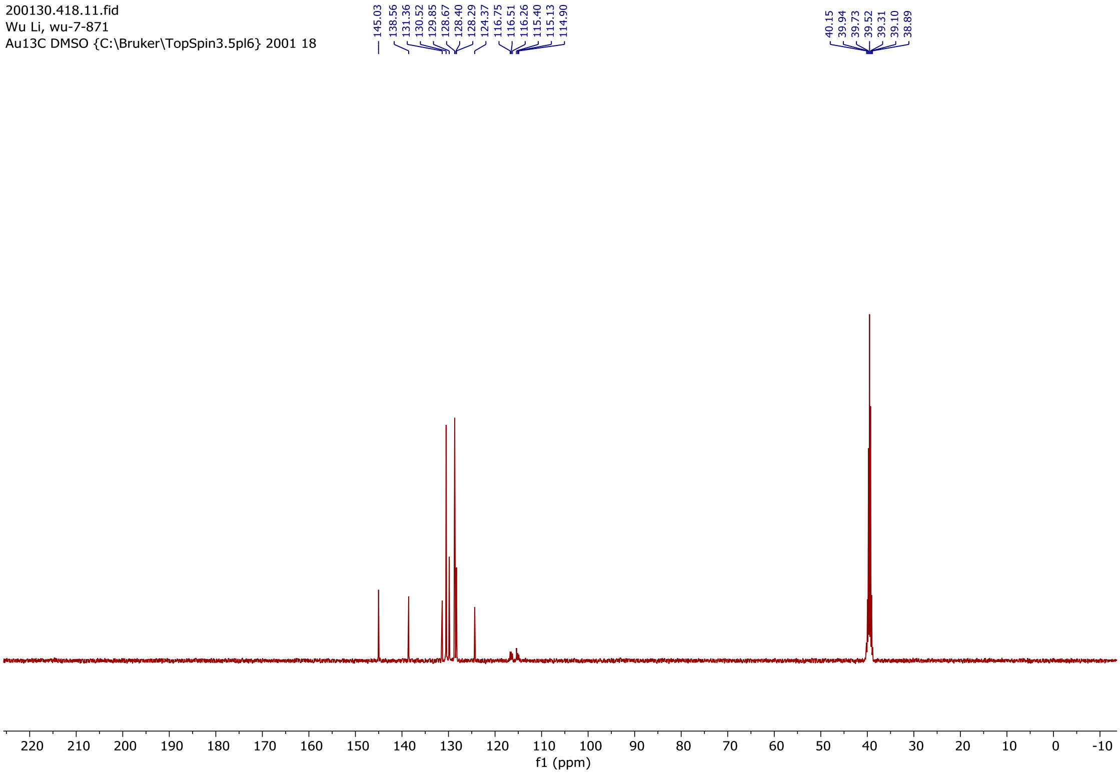

1H NMR for 15a:

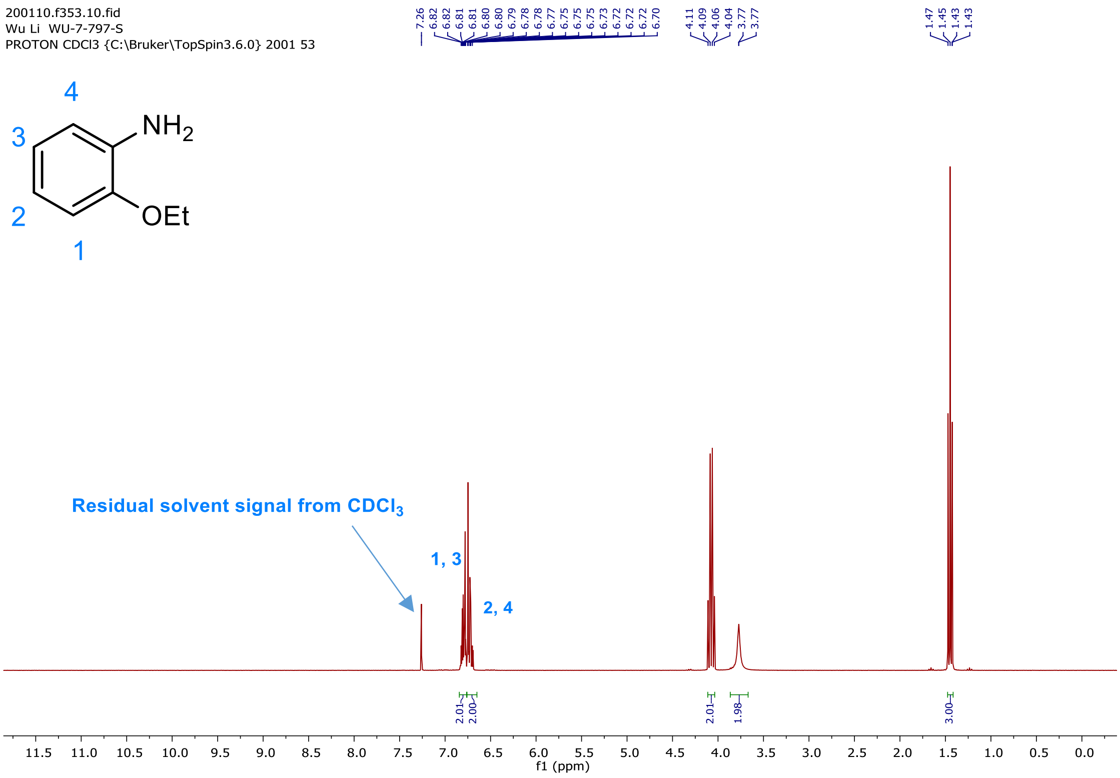

Original spectra for 15b:

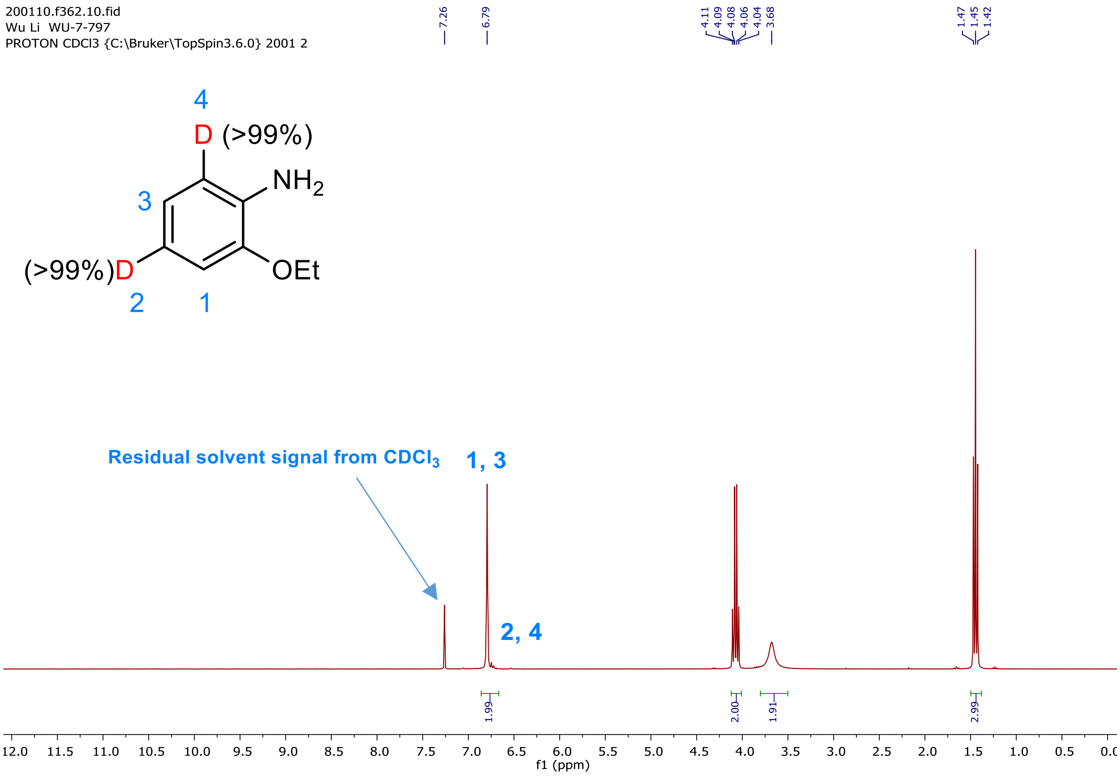

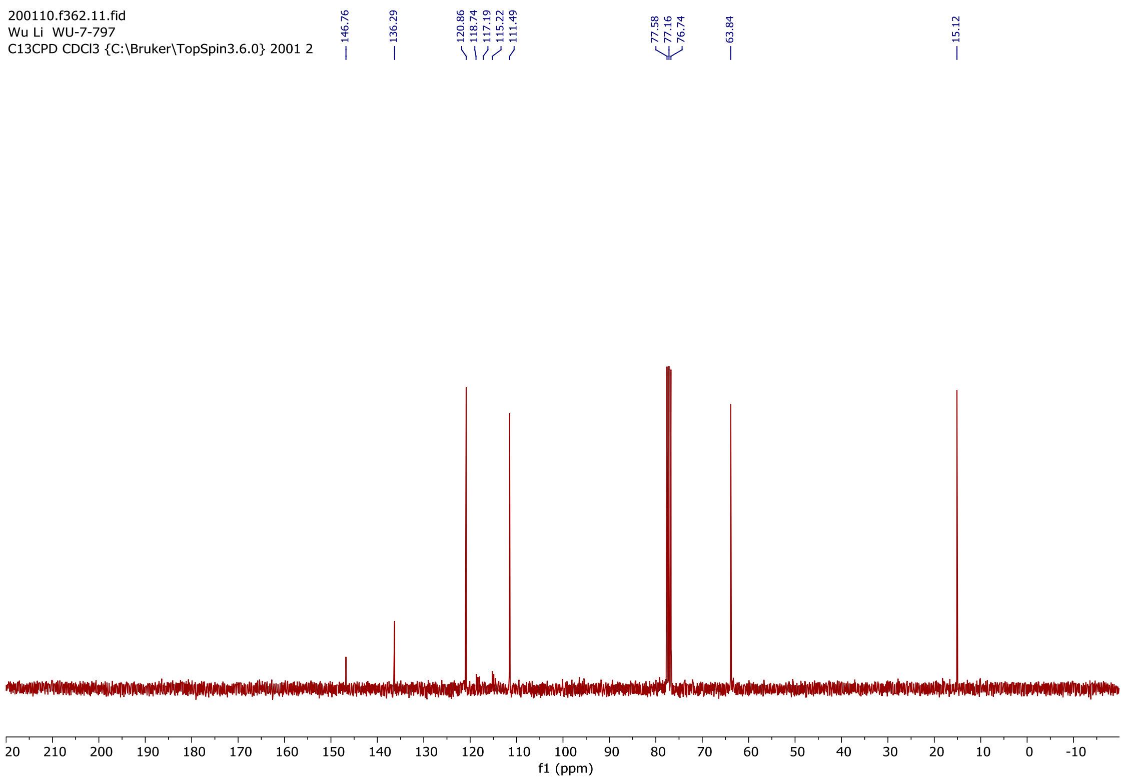

^{1}H NMR for 16a:

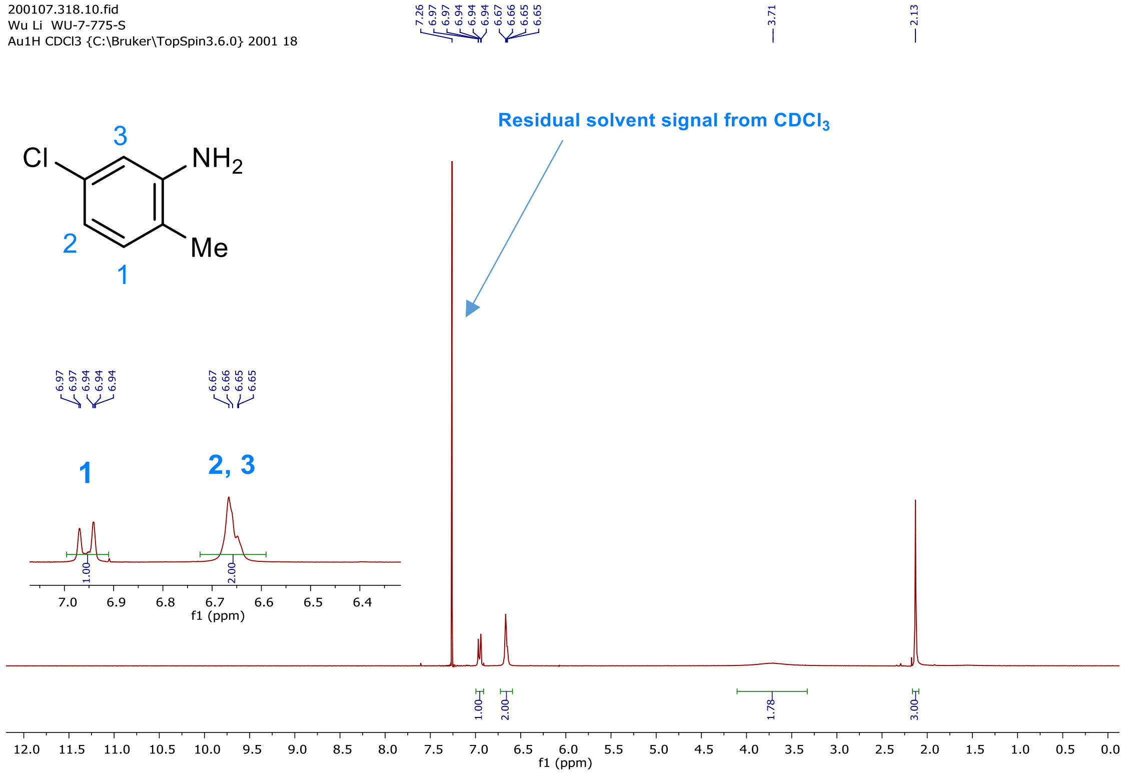

Original spectra for 16b:

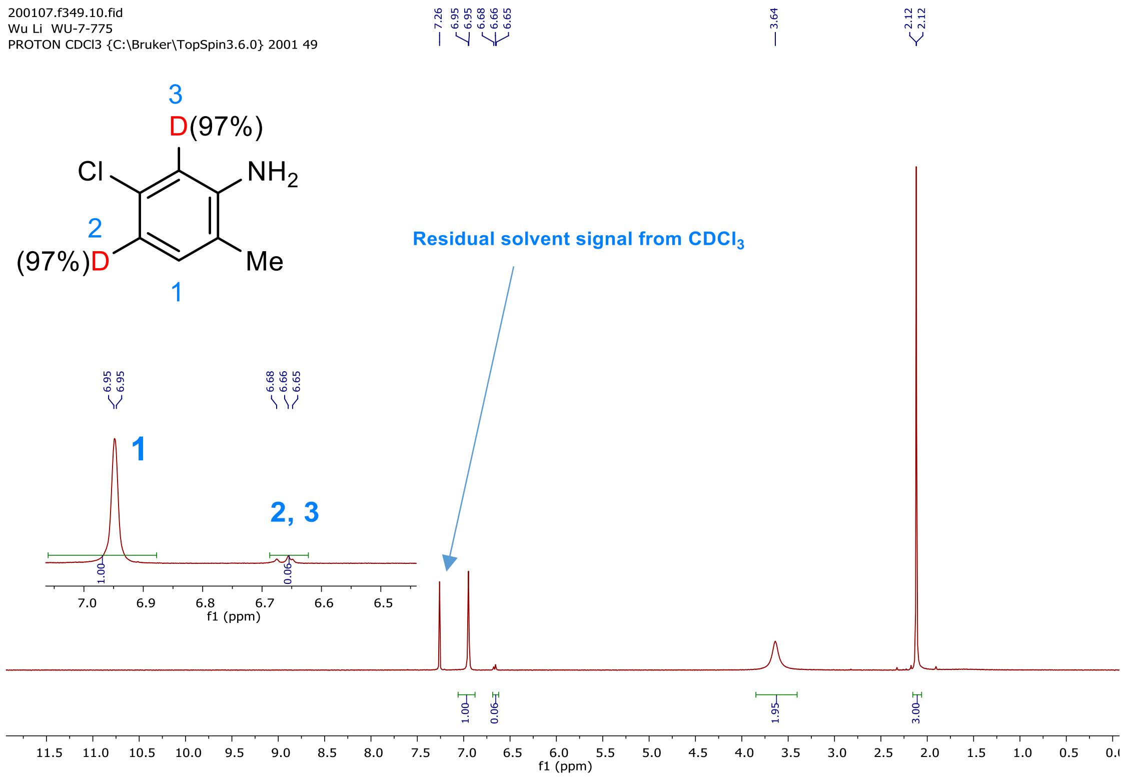

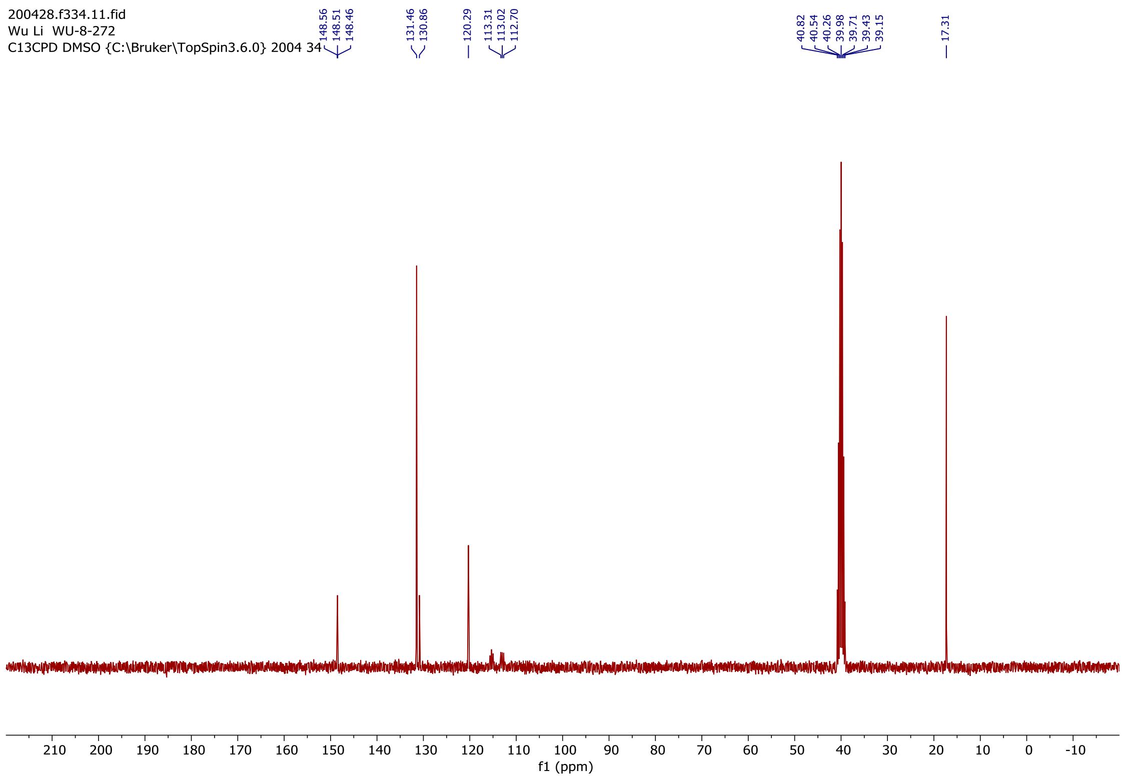

^{1}H NMR for 17a:

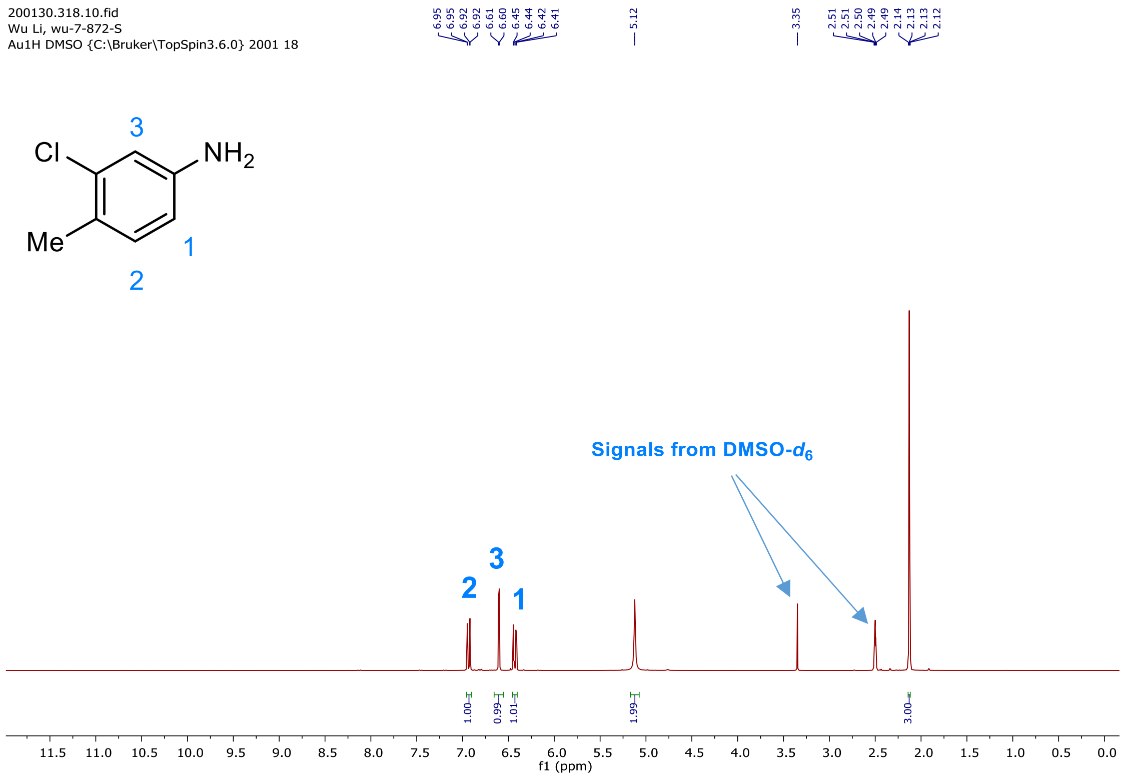

Original spectra for 17b:

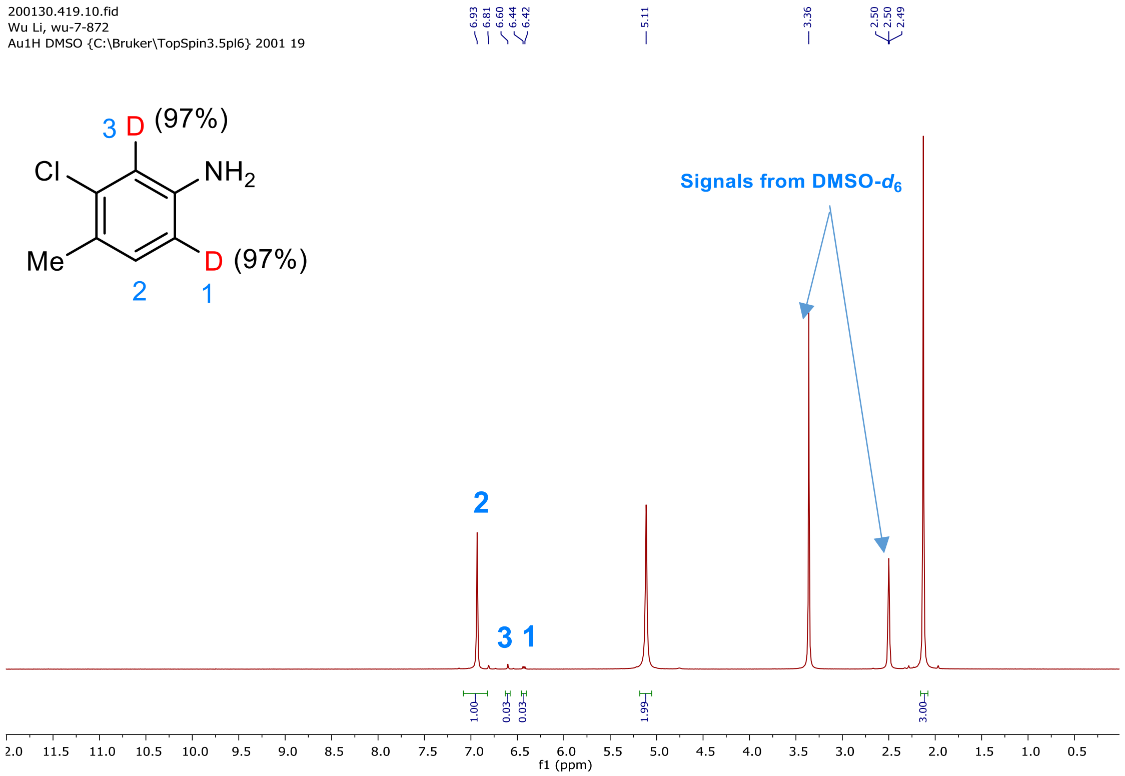

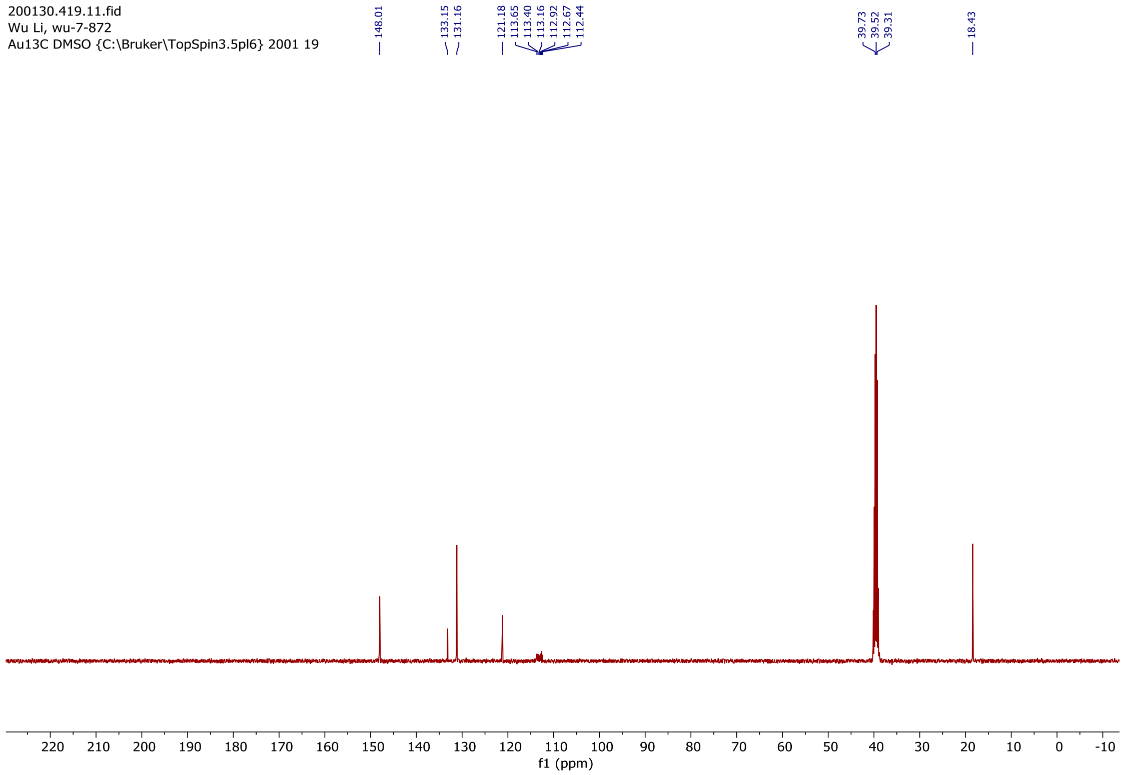

^{1}H NMR for 18a:

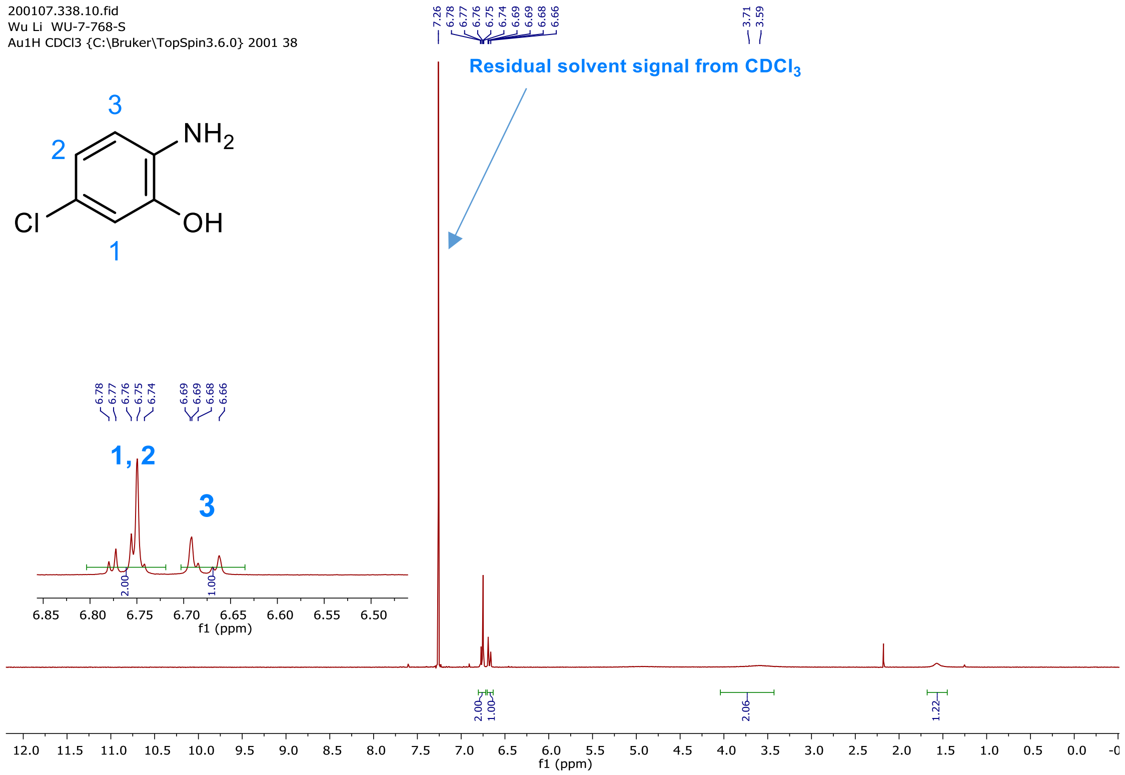

Original spectra for 18b:

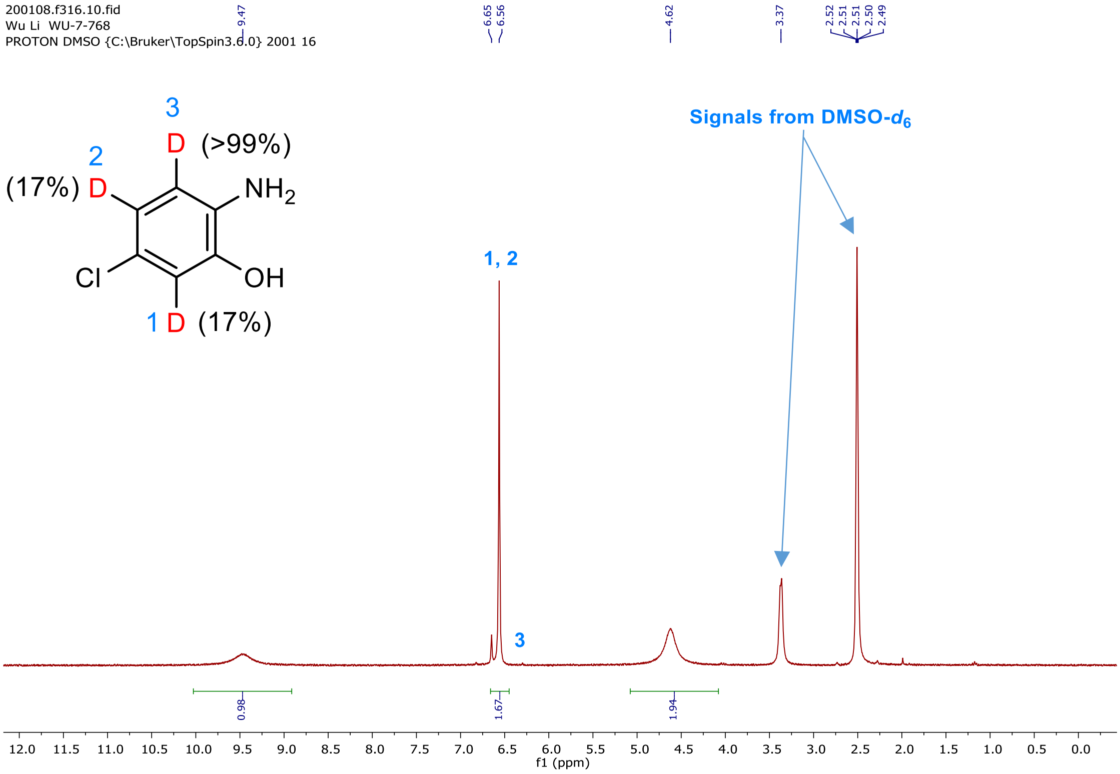

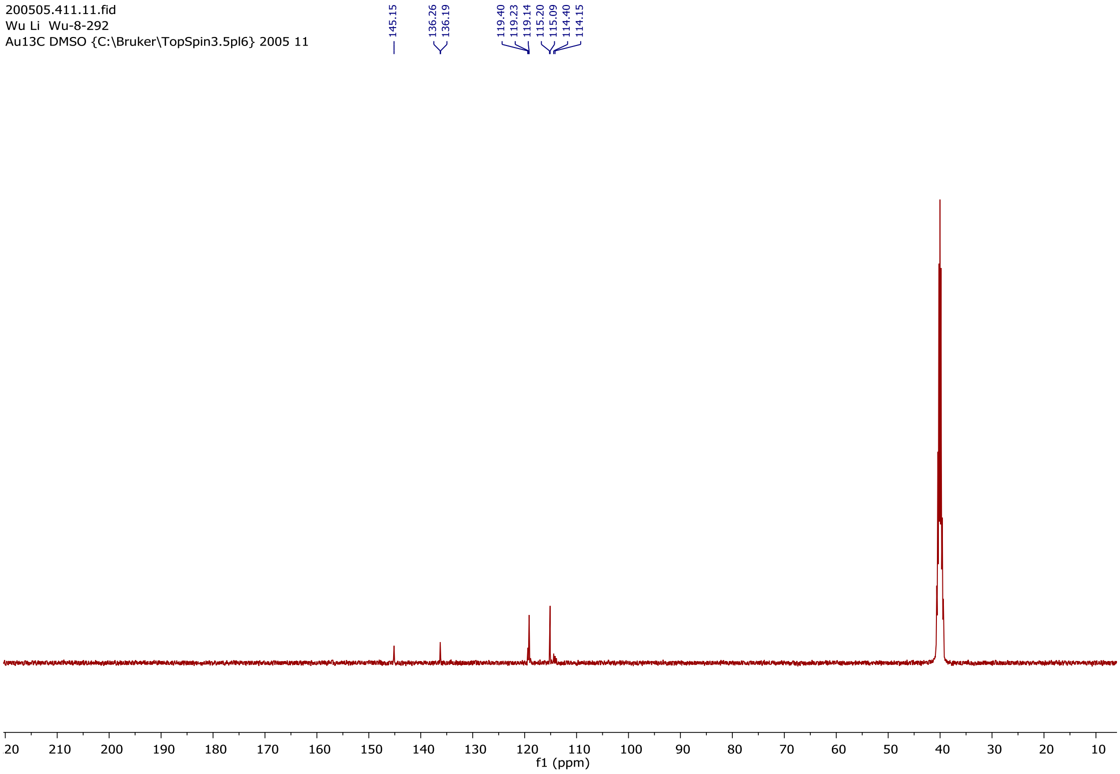

^{1}H NMR for 19a:

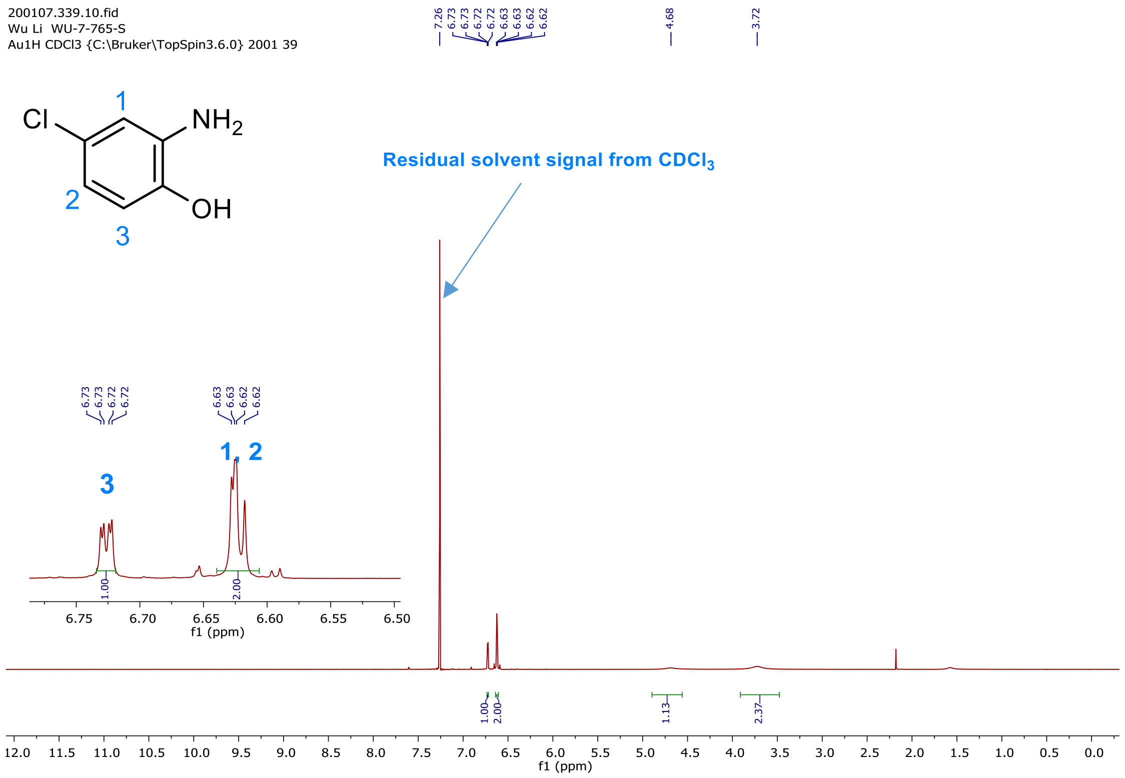

Original spectra for 19b:

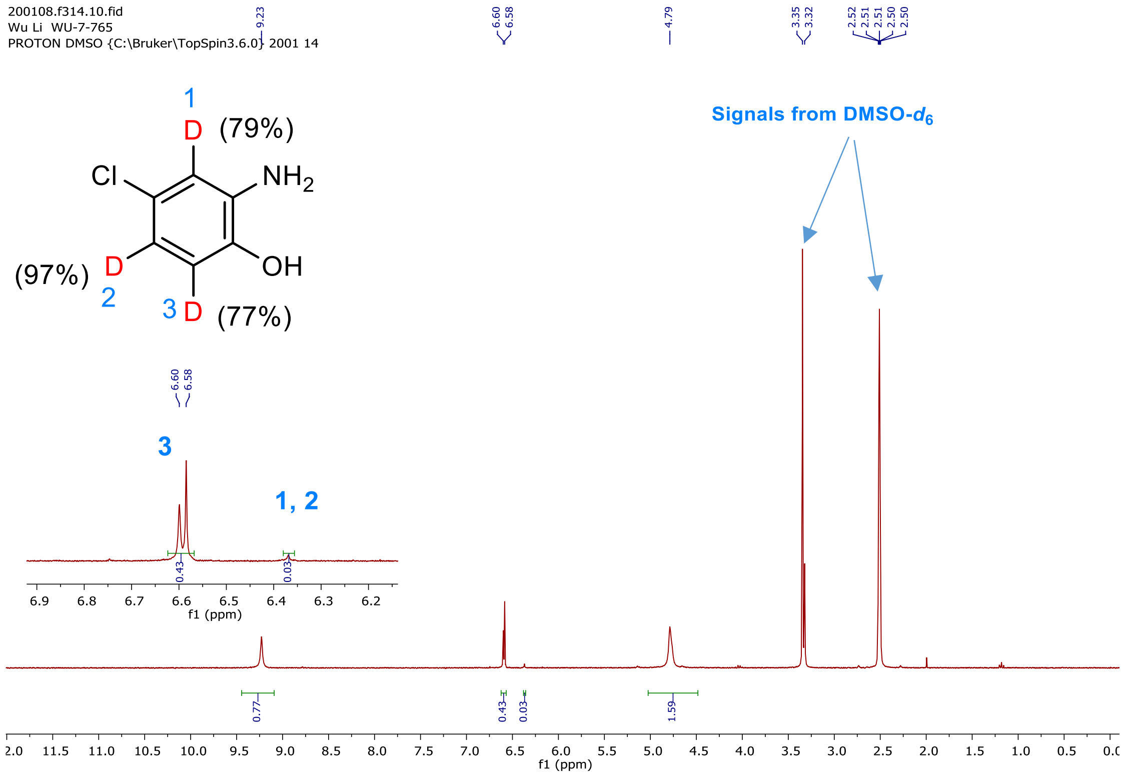

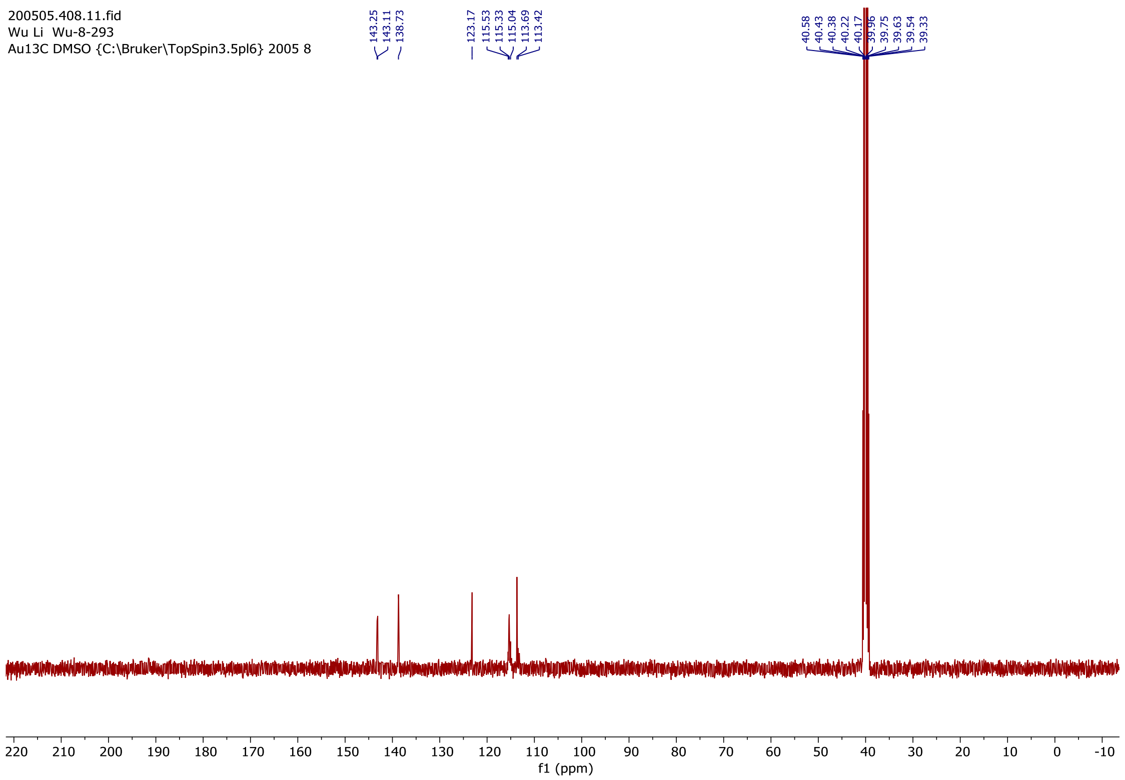
200505.408.11.fid
Wu Li Wu-8-293
Au13C DMSO {C:\Bruker\TopSpin3.5pl6} 2005 8
143.25
143.11
138.73
123.17
115.53
115.33
115.04
113.69
113.42
40.58
40.43
40.38
40.22
40.17
39.96
39.75
39.63
39.54
39.33
f1 (ppm)

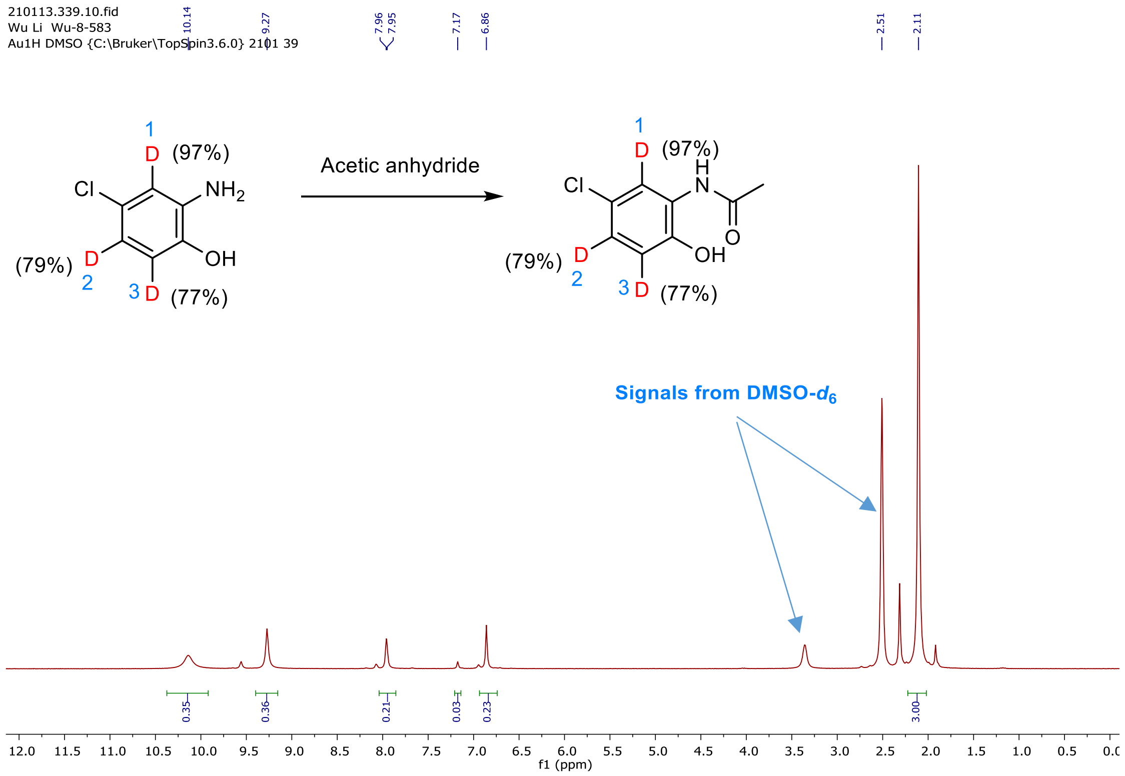
210113.339.10.fid
Wu Li Wu-8-583
Au1H DMSO {C:\Bruker\TopSpin3.6.0} 2101 39
10.14
9.27
7.96
7.95
7.17
6.86
2.51
2.11
Acetic anhydride
Cl
NH2
OH
D (97%)
(79%) D
D (77%)
1
2
3
Signals from DMSO-d6
0.35
0.36
0.21
0.03
0.23
3.00
f1 (ppm)

^{1}H NMR for 20a:

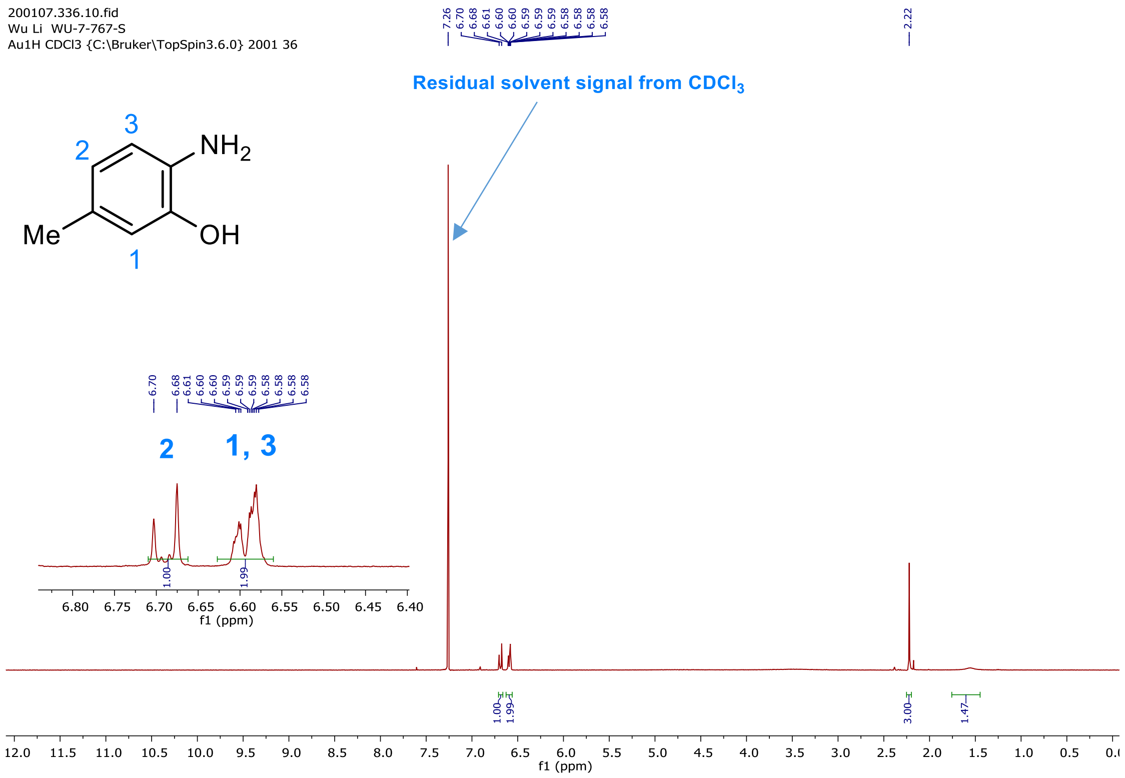

Original spectra for 20b:

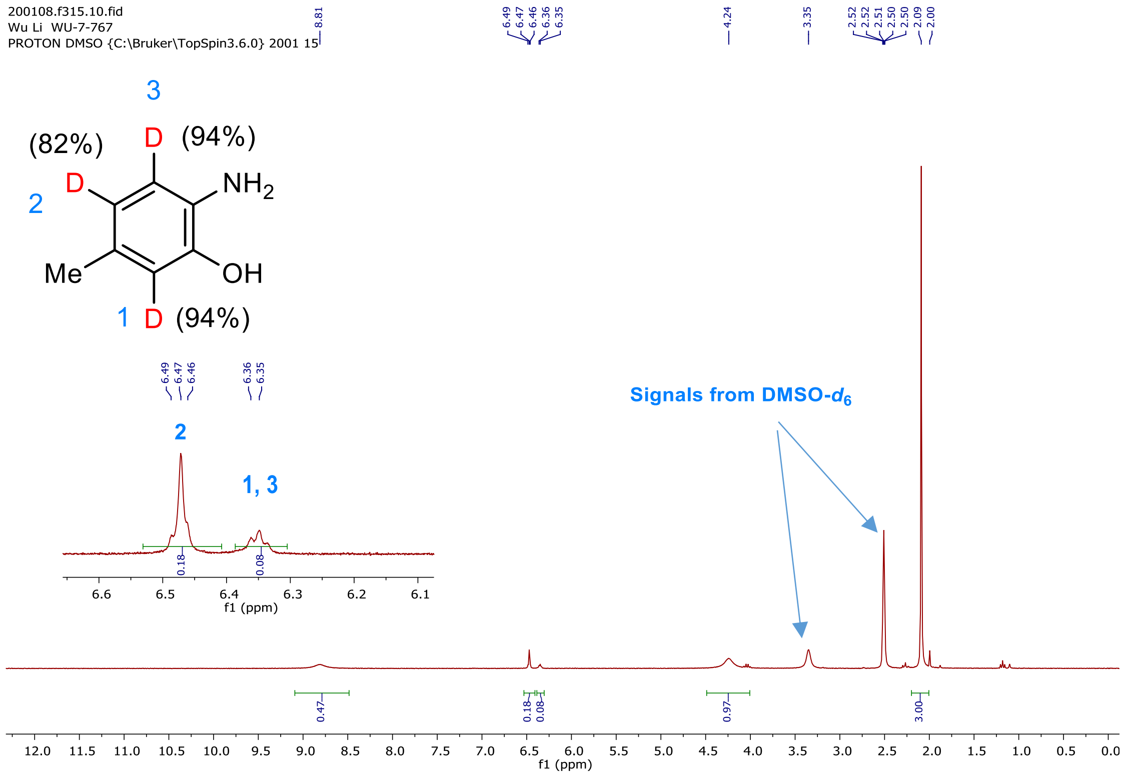

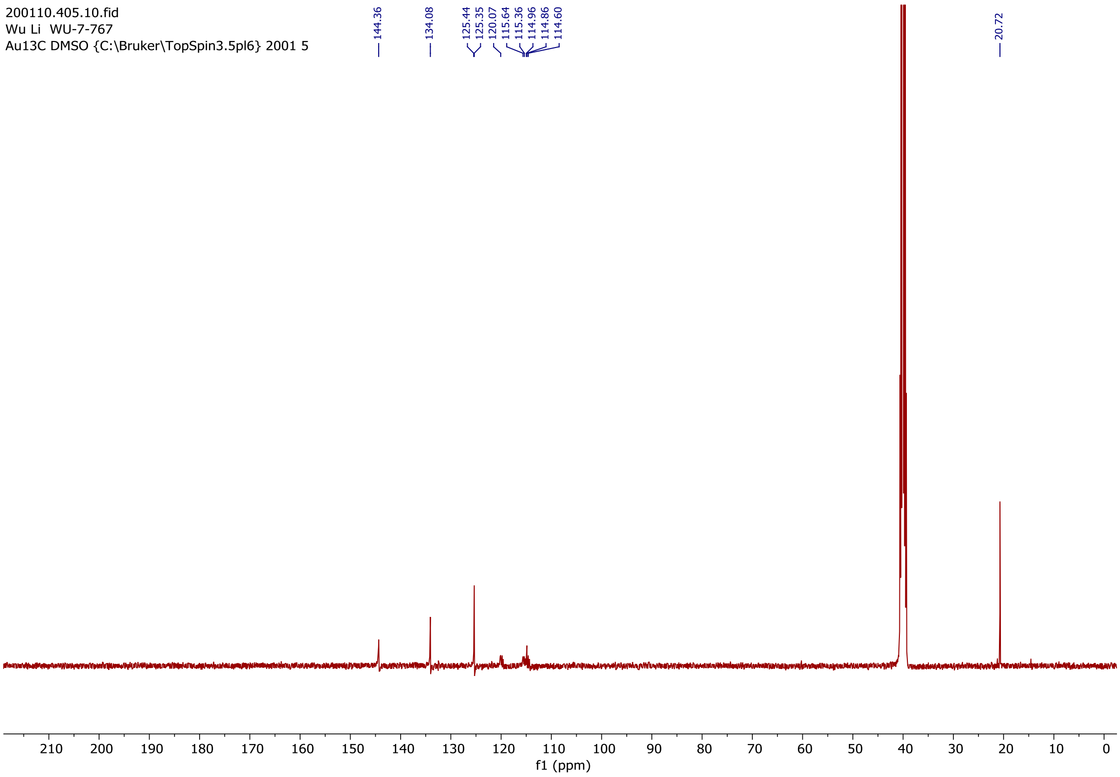

^{1}H NMR for 21a:

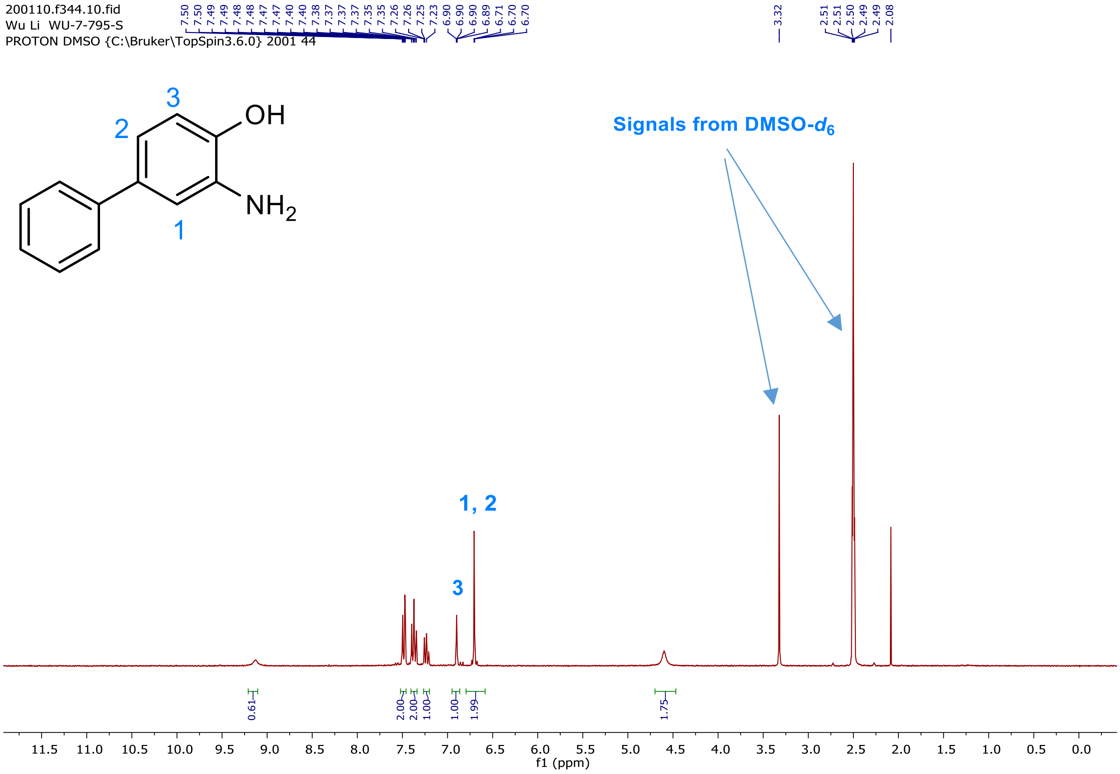

Original spectra for 21b:

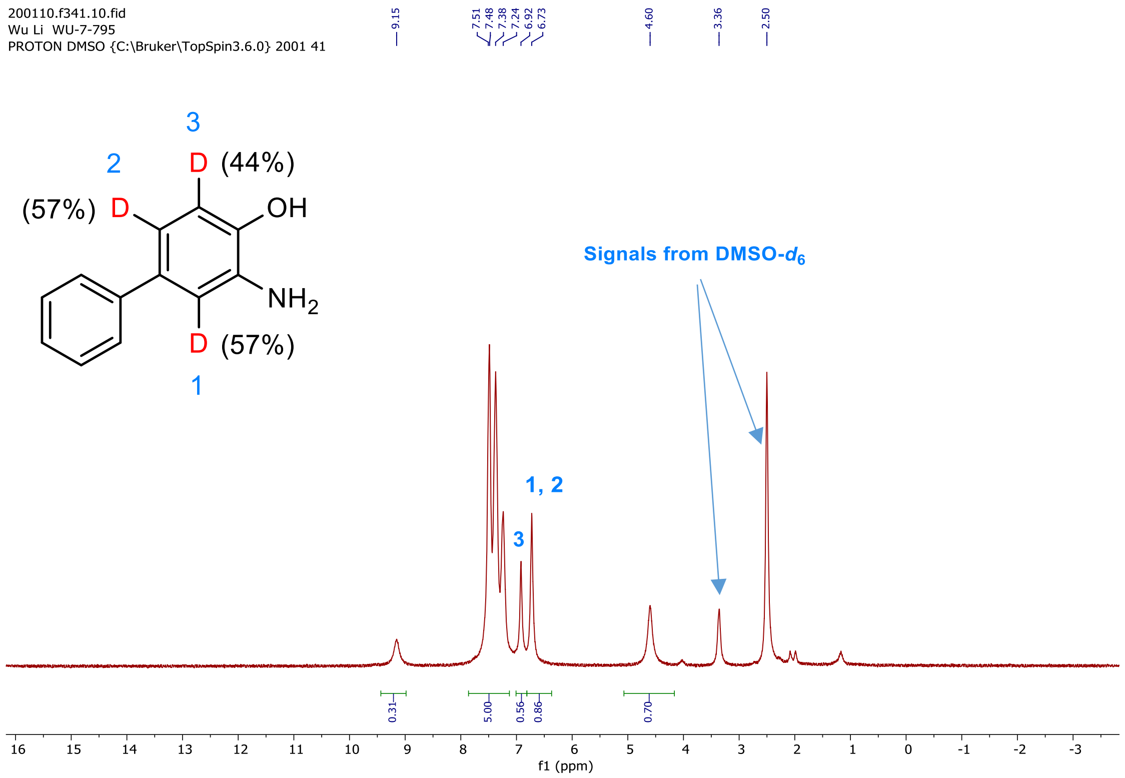

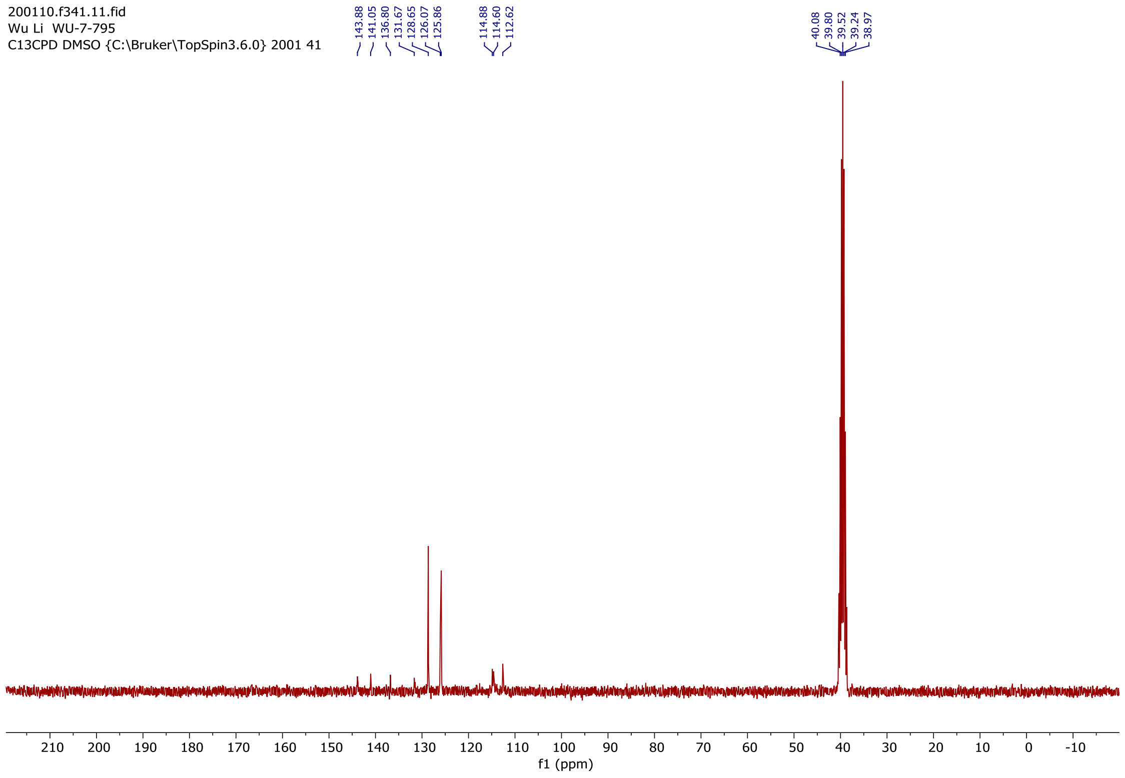

^{1}H NMR for 22a:

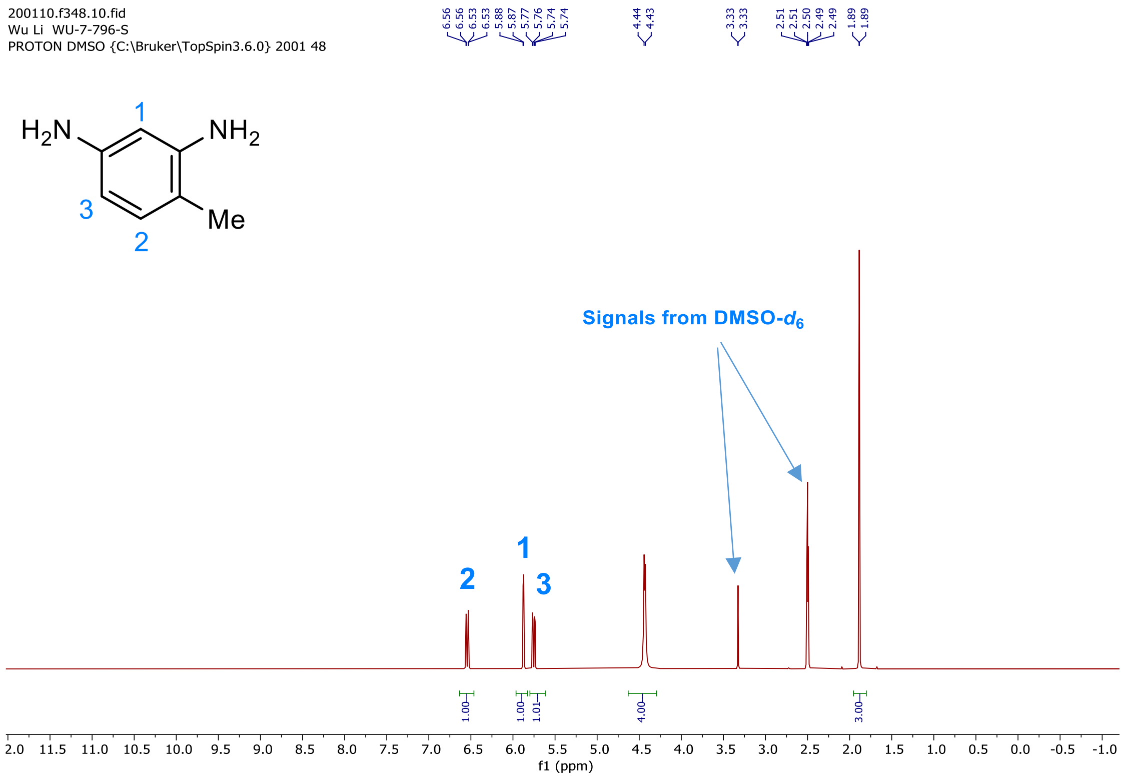

Original spectra for 22b:

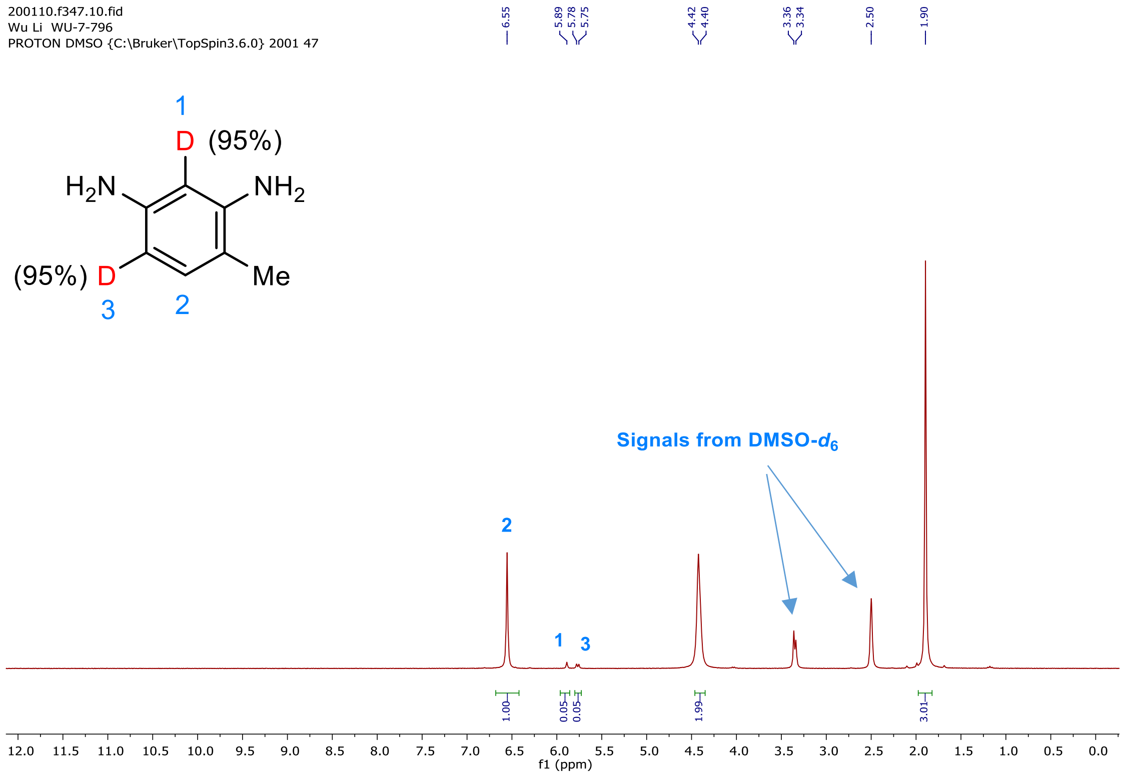

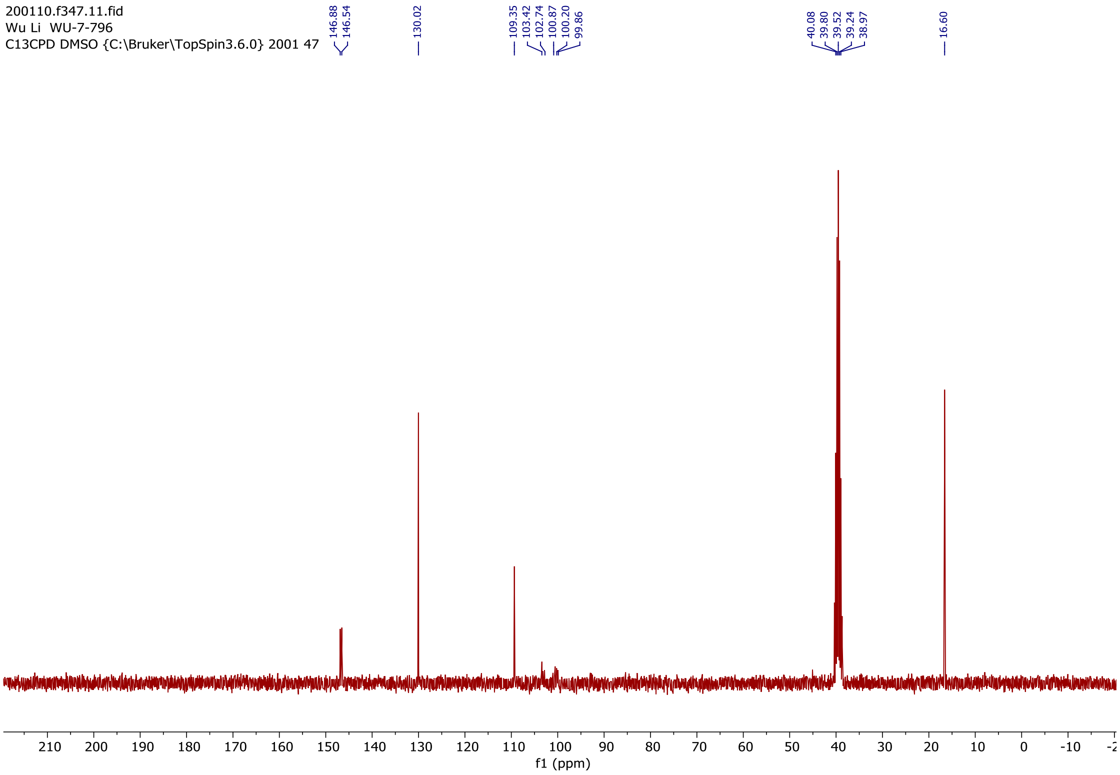

^{1}H NMR for 23a:

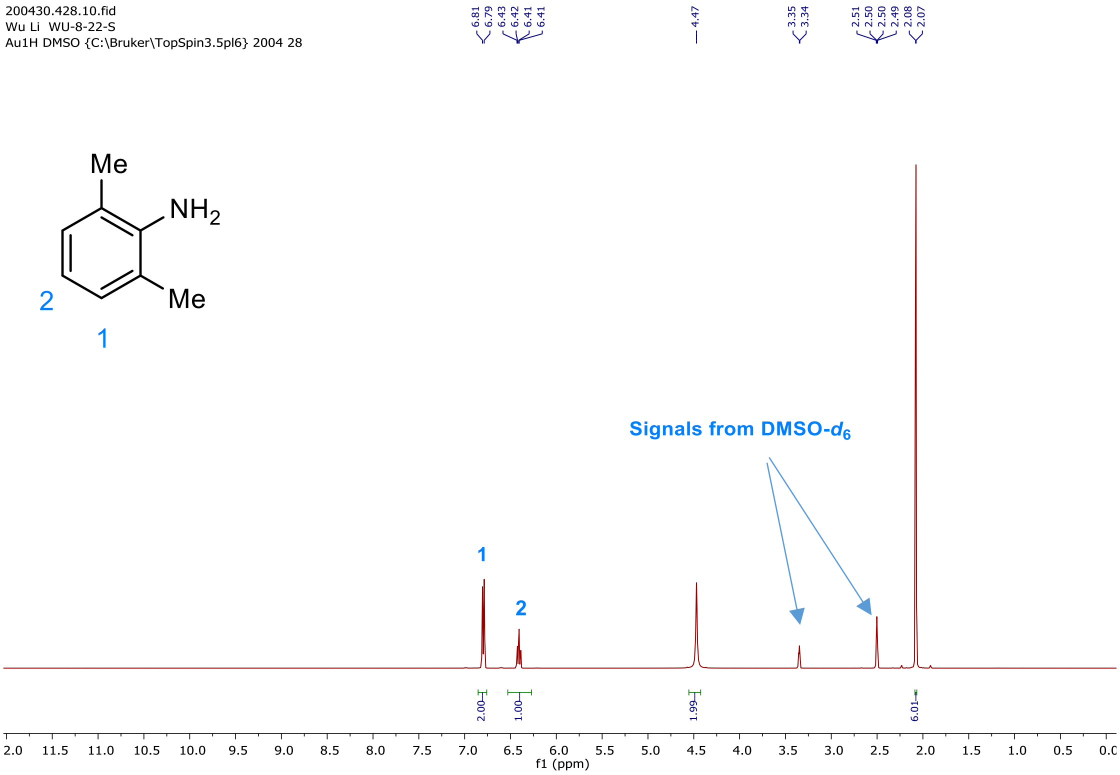

Original spectra for 23b:

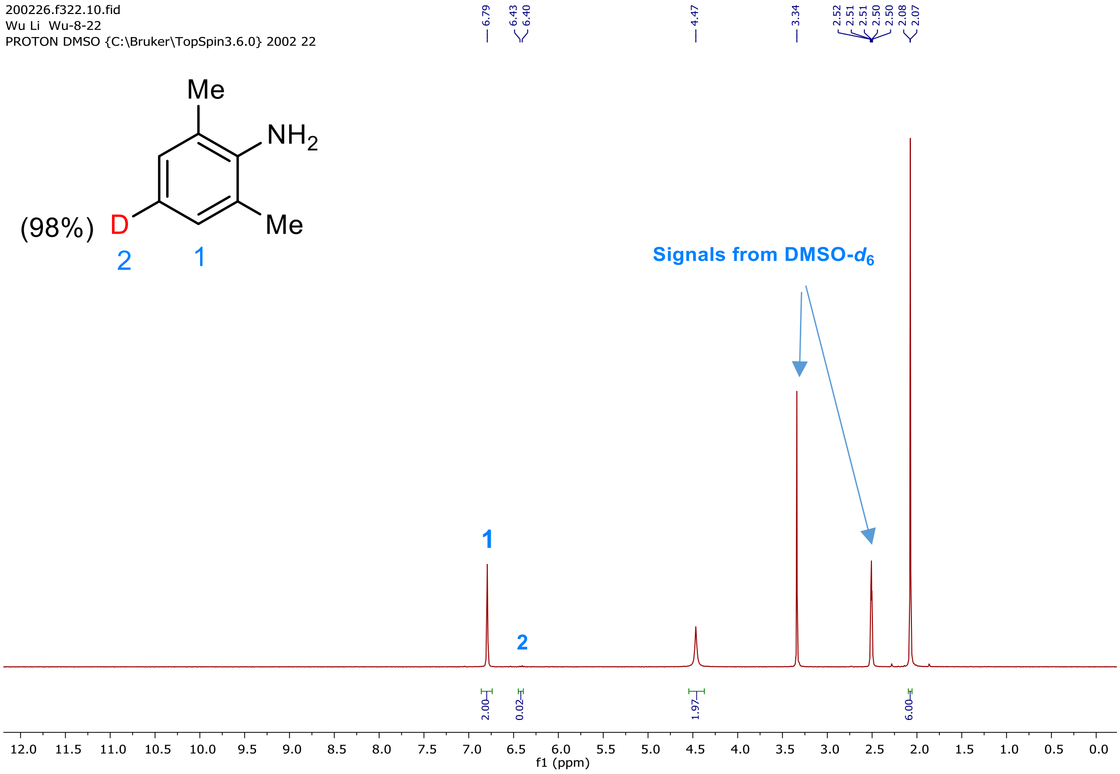

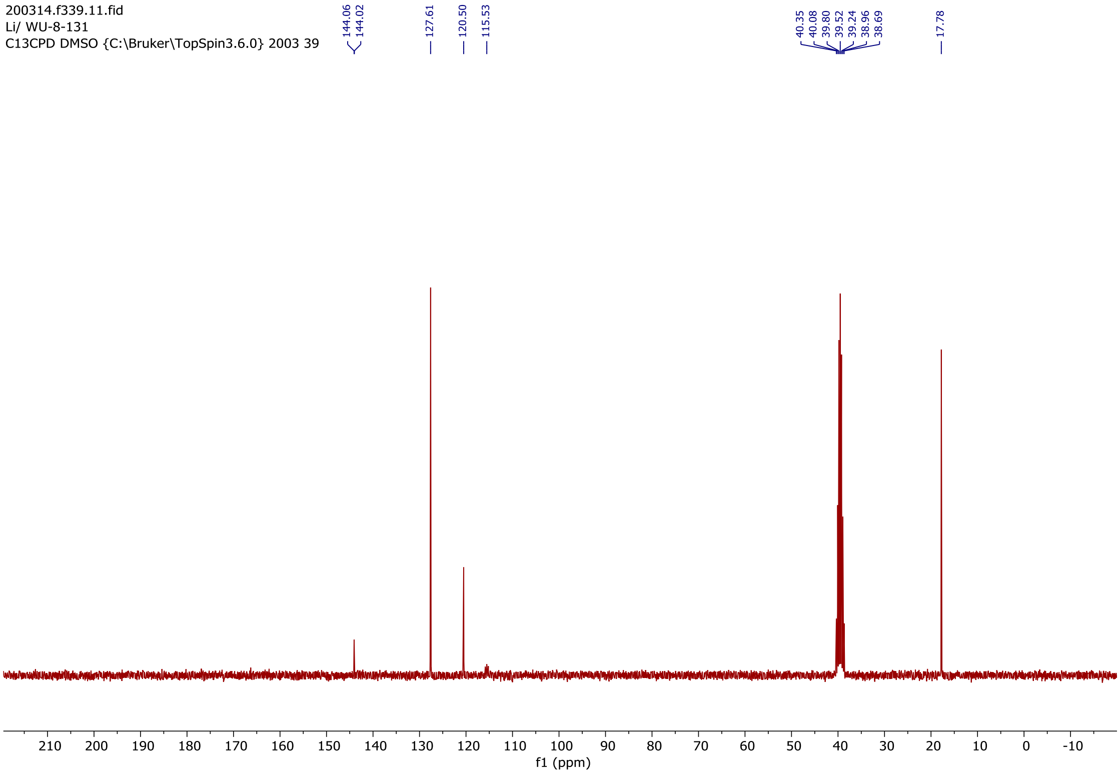

^{1}H NMR for 24a:

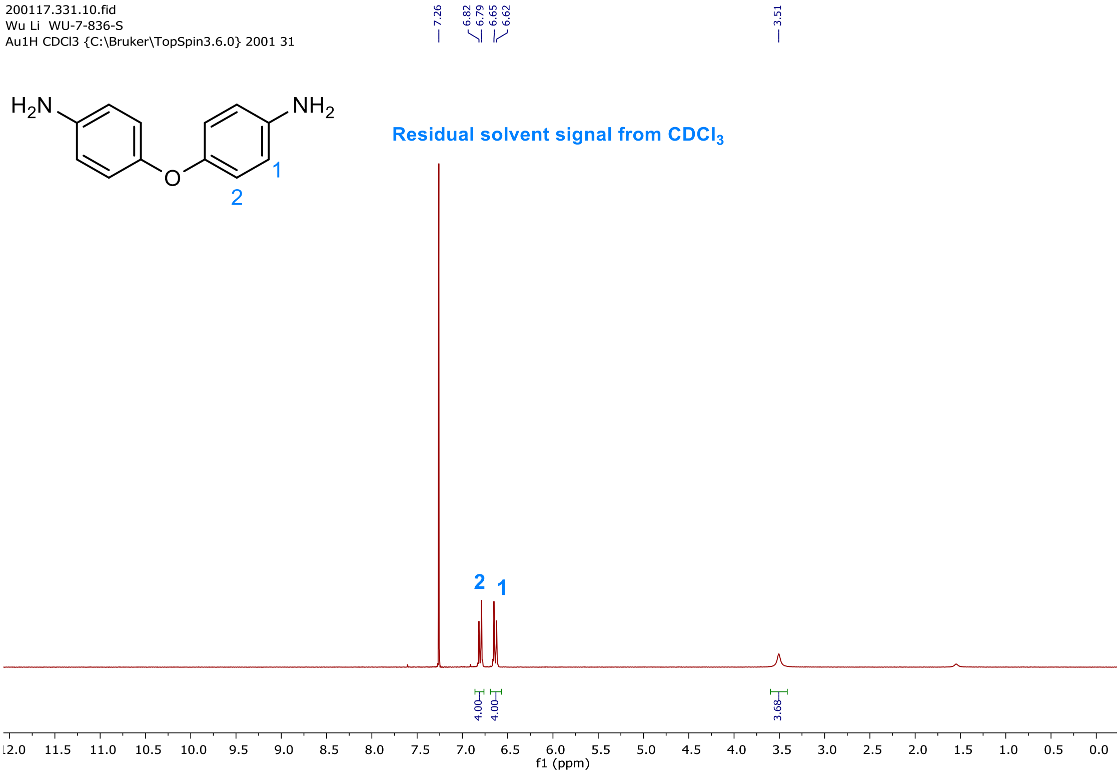

Original spectra for 24b:

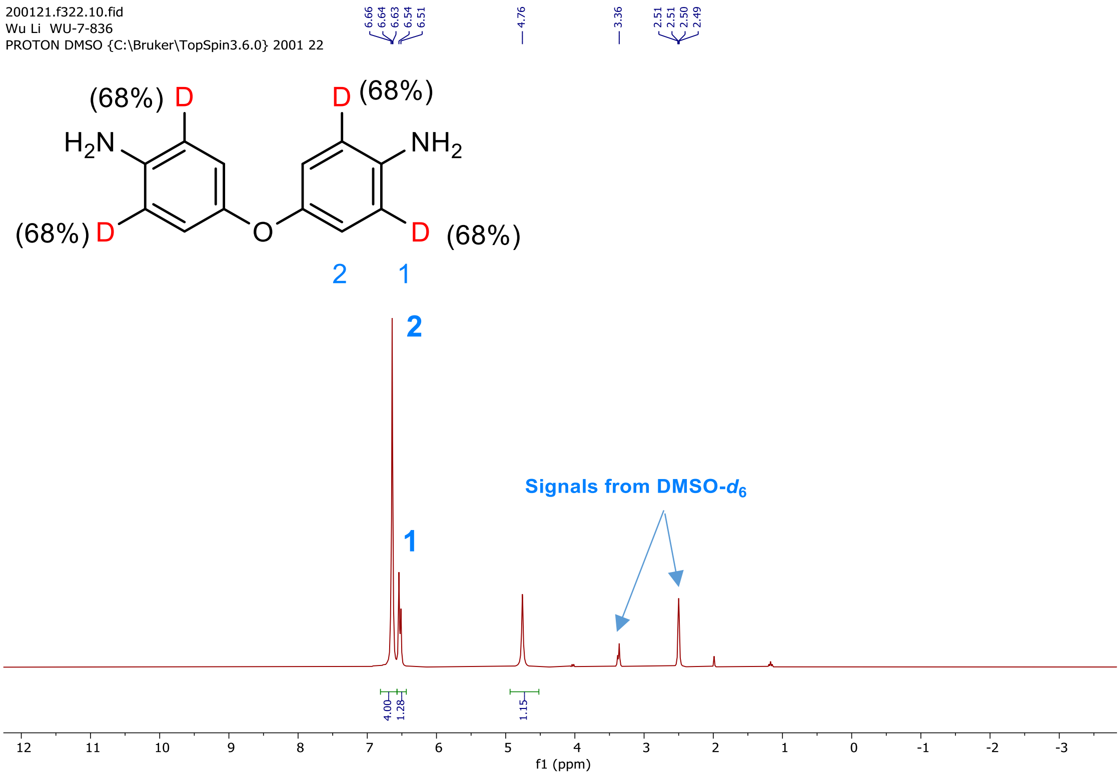

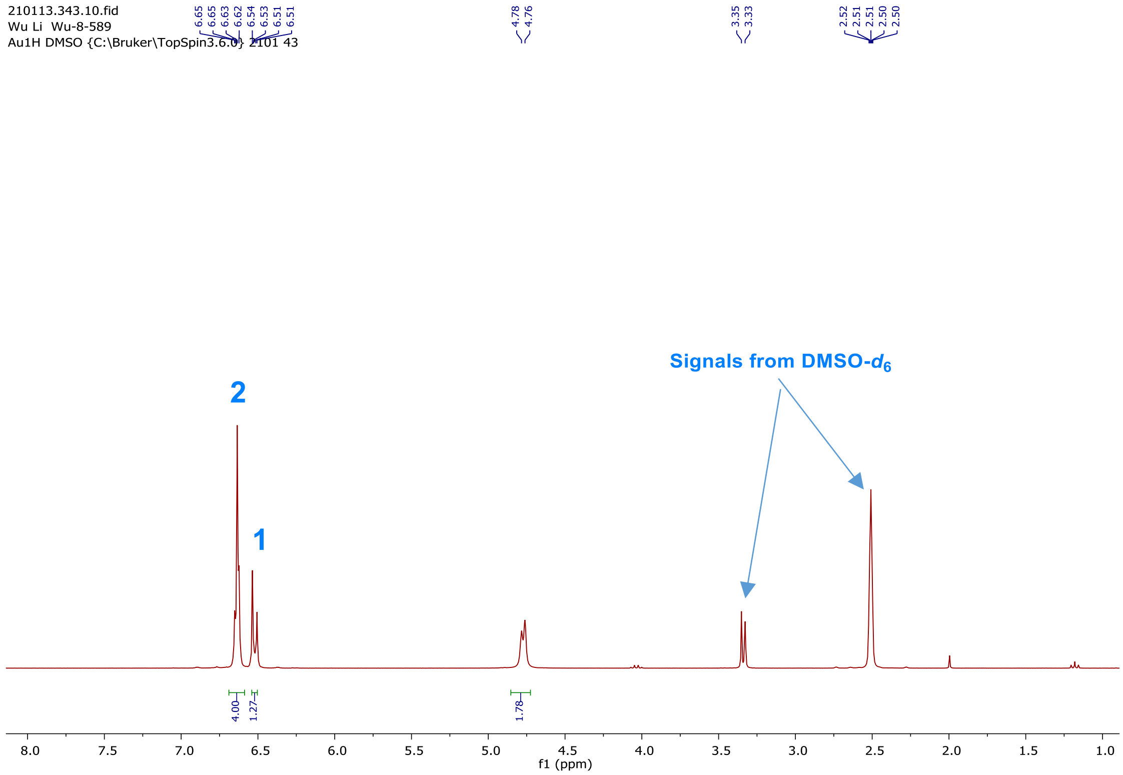

210113.343.10.fid
Wu Li Wu-8-589
Au1H DMSO {C:\Bruker\TopSpin3.6.0} 2101 43
6.65 6.65 6.63 6.62 6.54 6.53 6.51 6.51
4.78 4.76
3.35 3.33
2.52 2.51 2.51 2.50 2.50
2
1
Signals from DMSO-d_6
4.00
1.27
1.78
f1 (ppm)

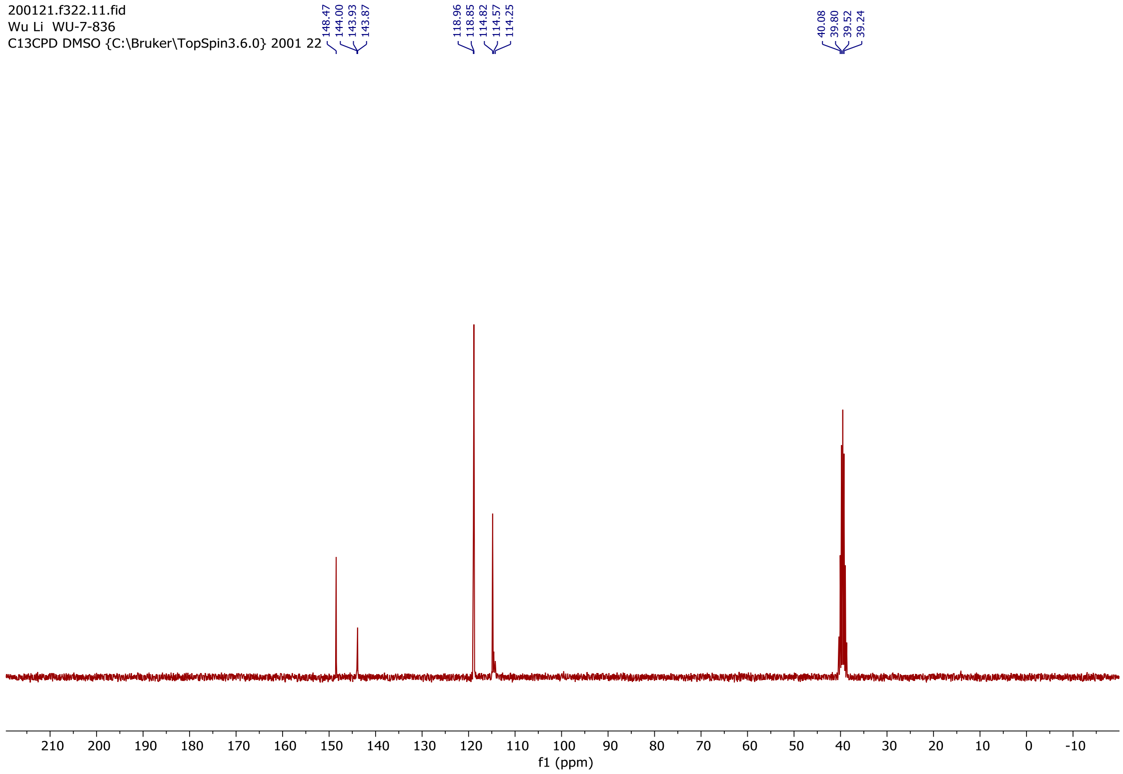

200121.f322.11.fid
Wu Li WU-7-836
C13CPD DMSO {C:\Bruker\TopSpin3.6.0} 2001 22
148.47 144.00 143.93 143.87
118.96 118.85 114.82 114.57 114.25
40.08 39.80 39.52 39.24
f1 (ppm)

^{1}H NMR for 25a:

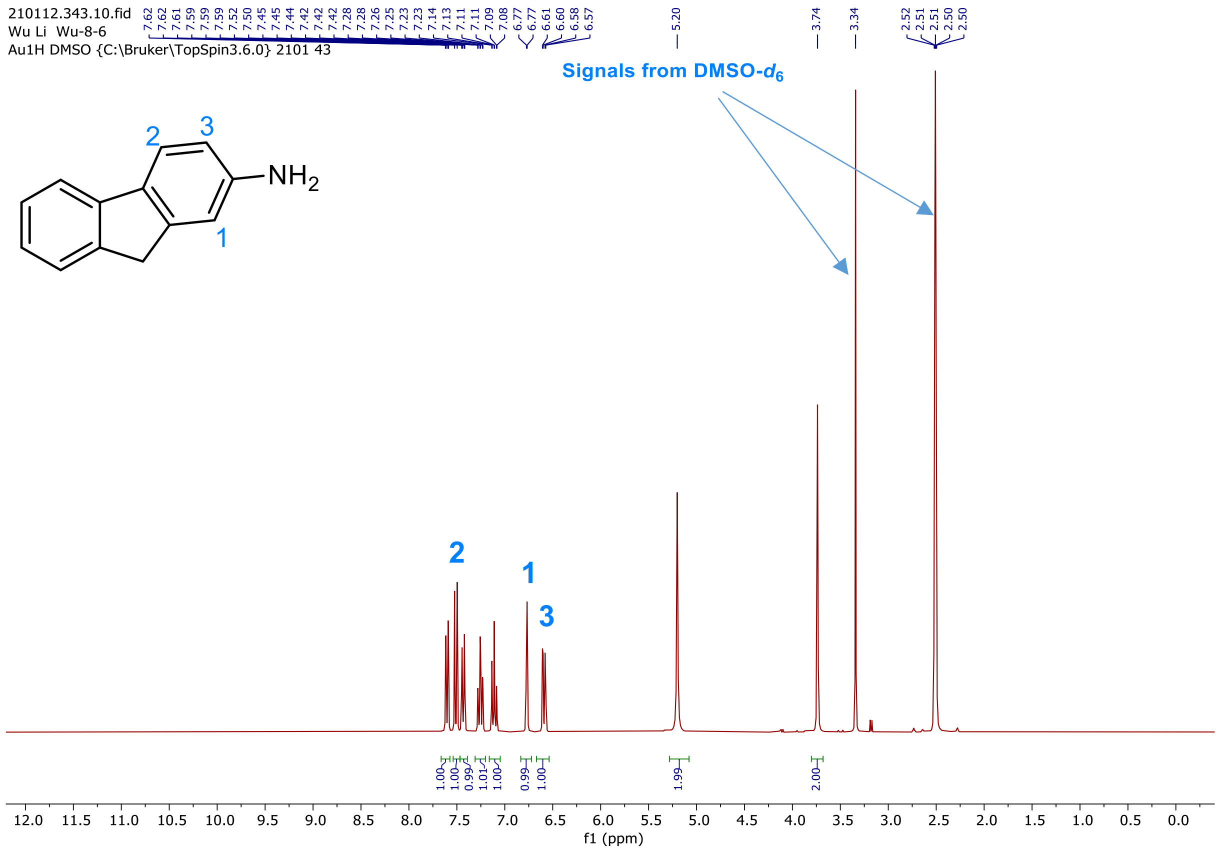

Original spectra for 25b:

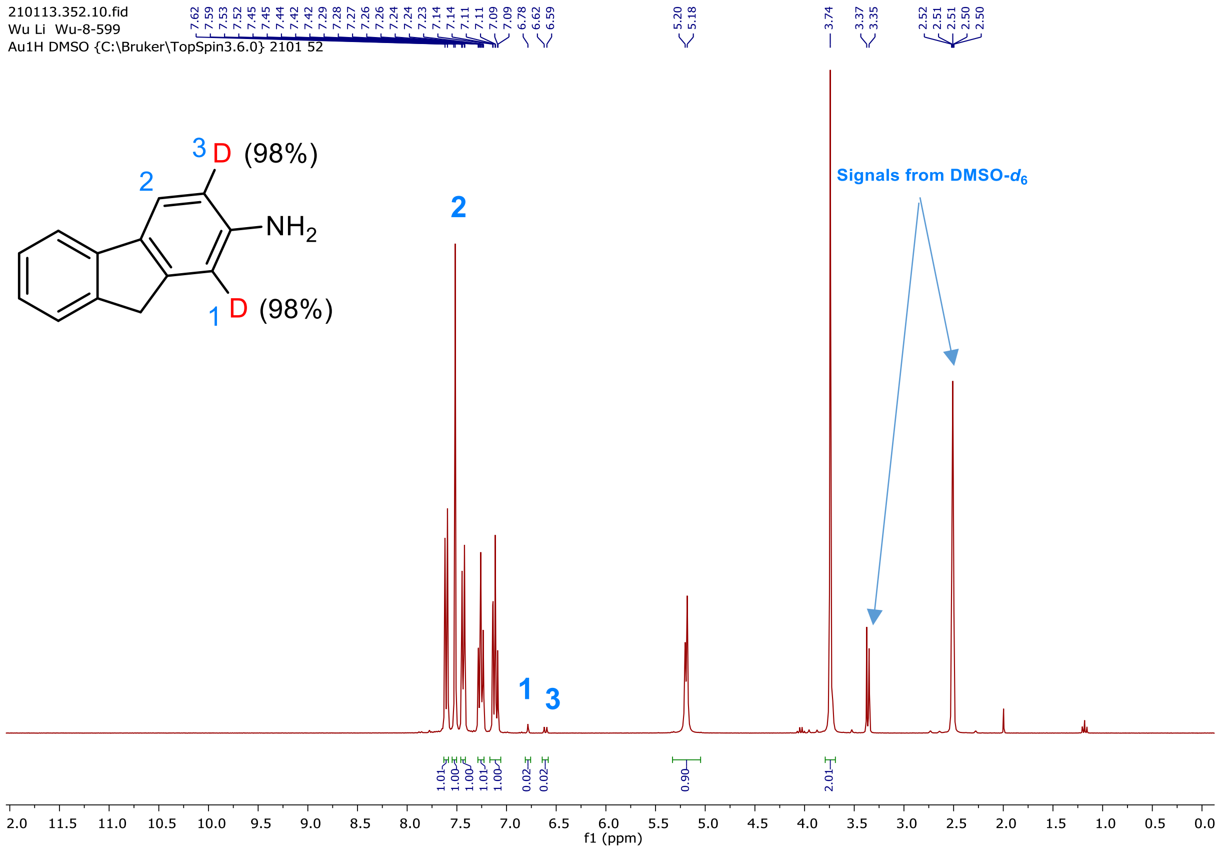

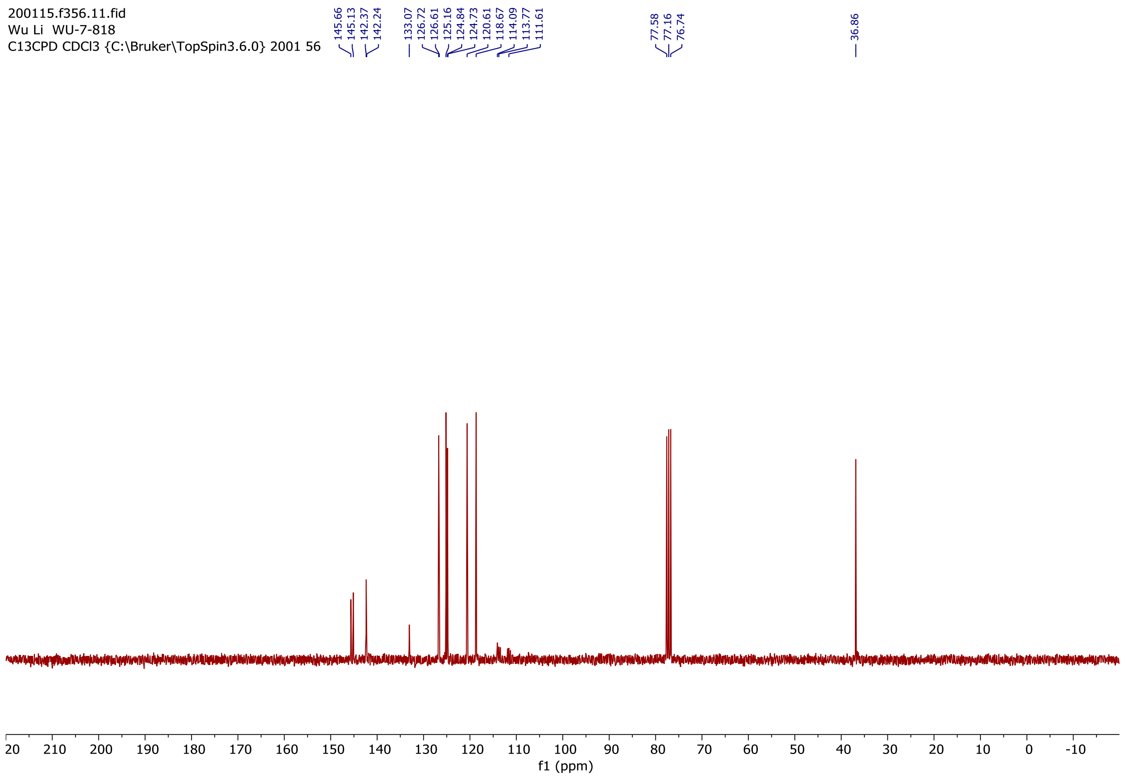

^{1}H NMR for 26a:

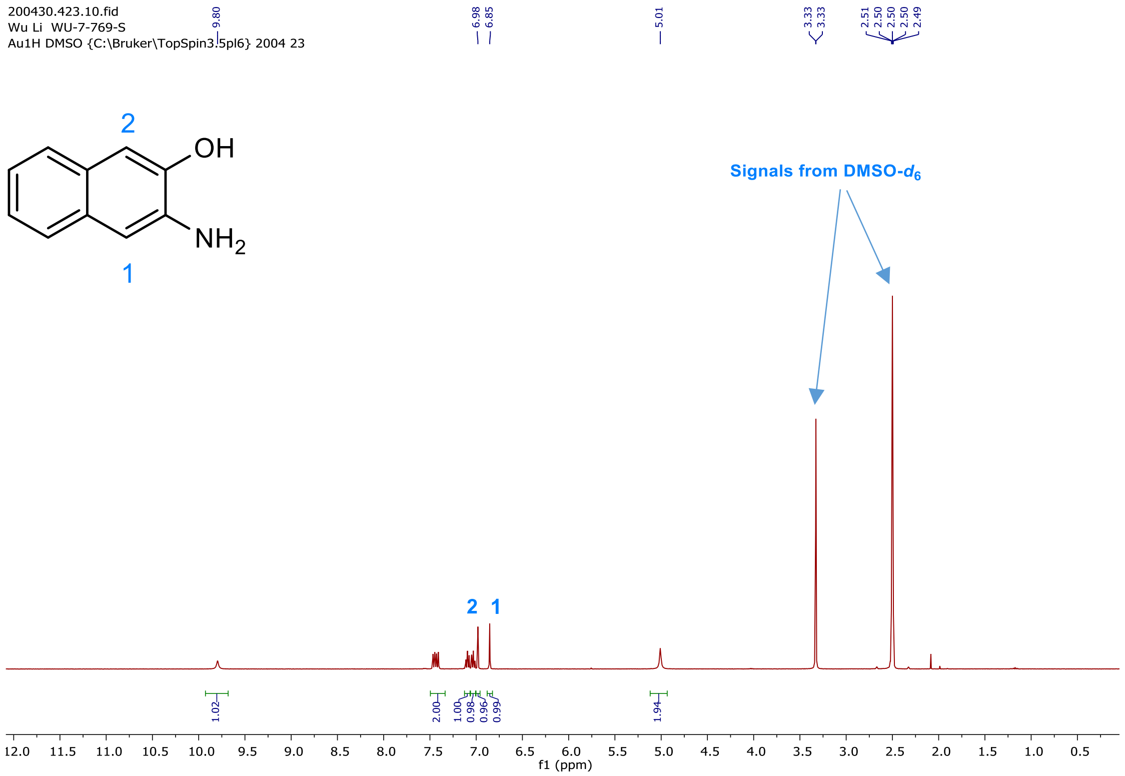

Original spectra for 26b:

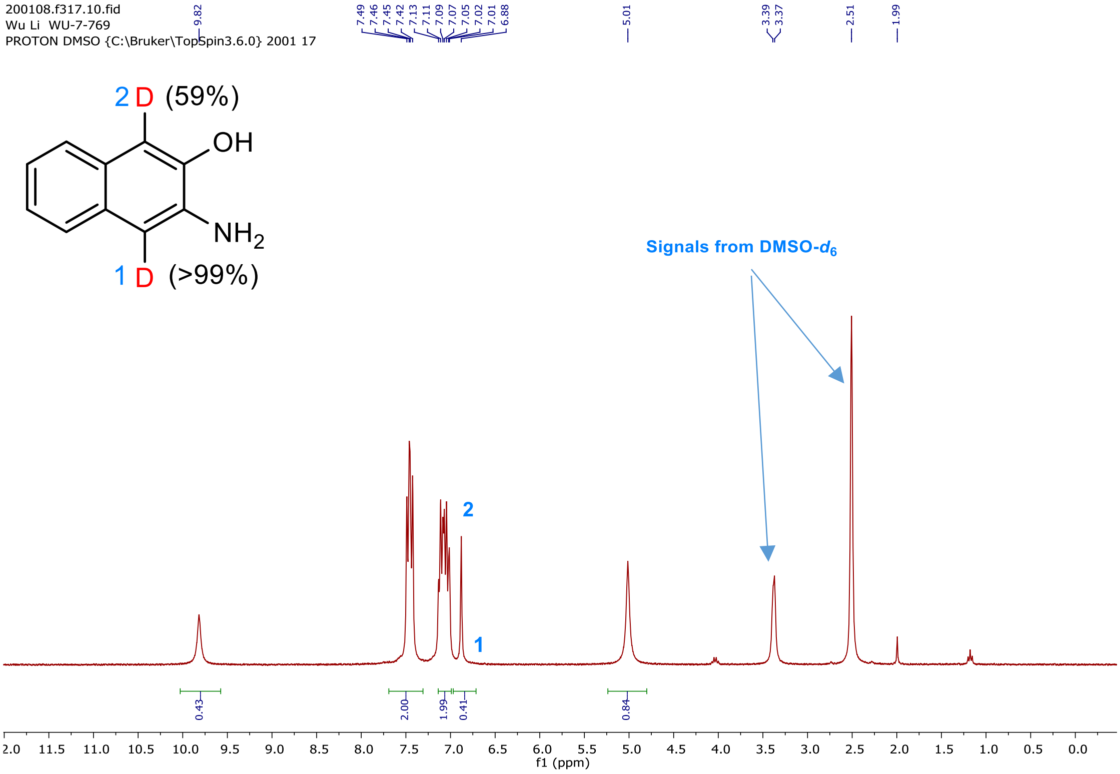

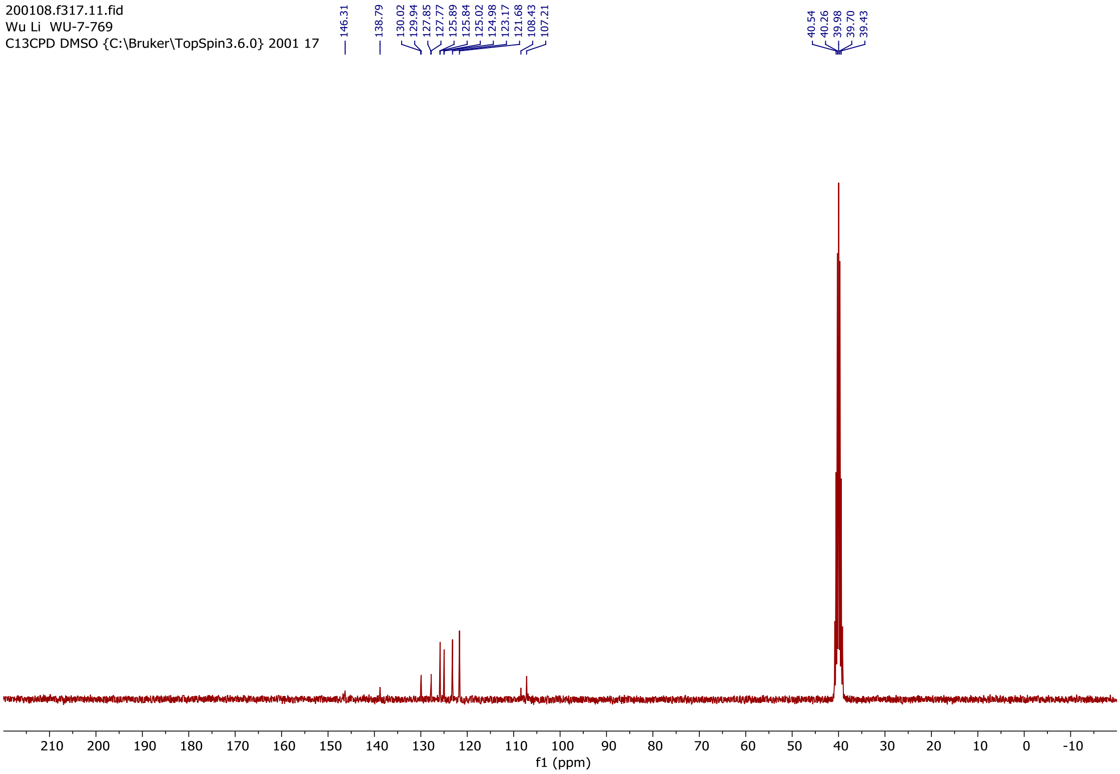

^{1}H NMR for 27a:

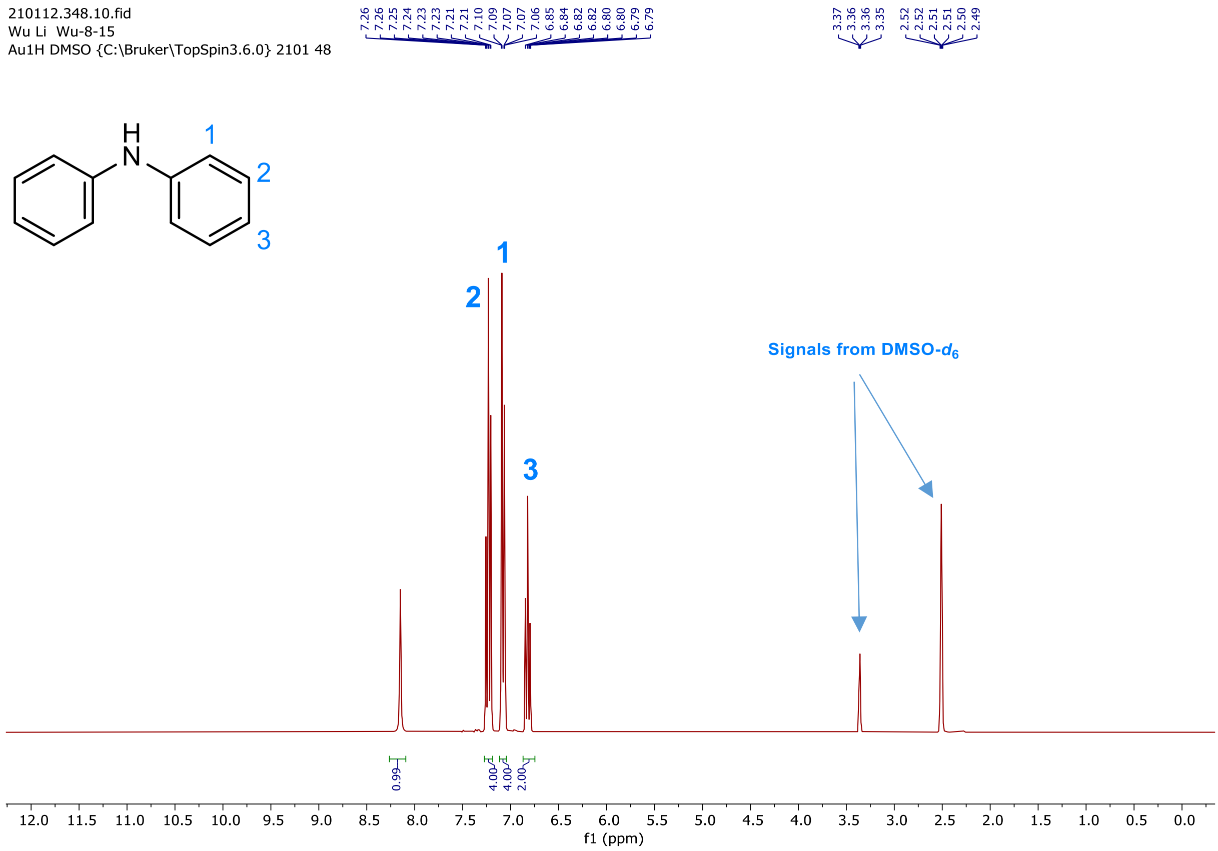

Original spectra for 27b:

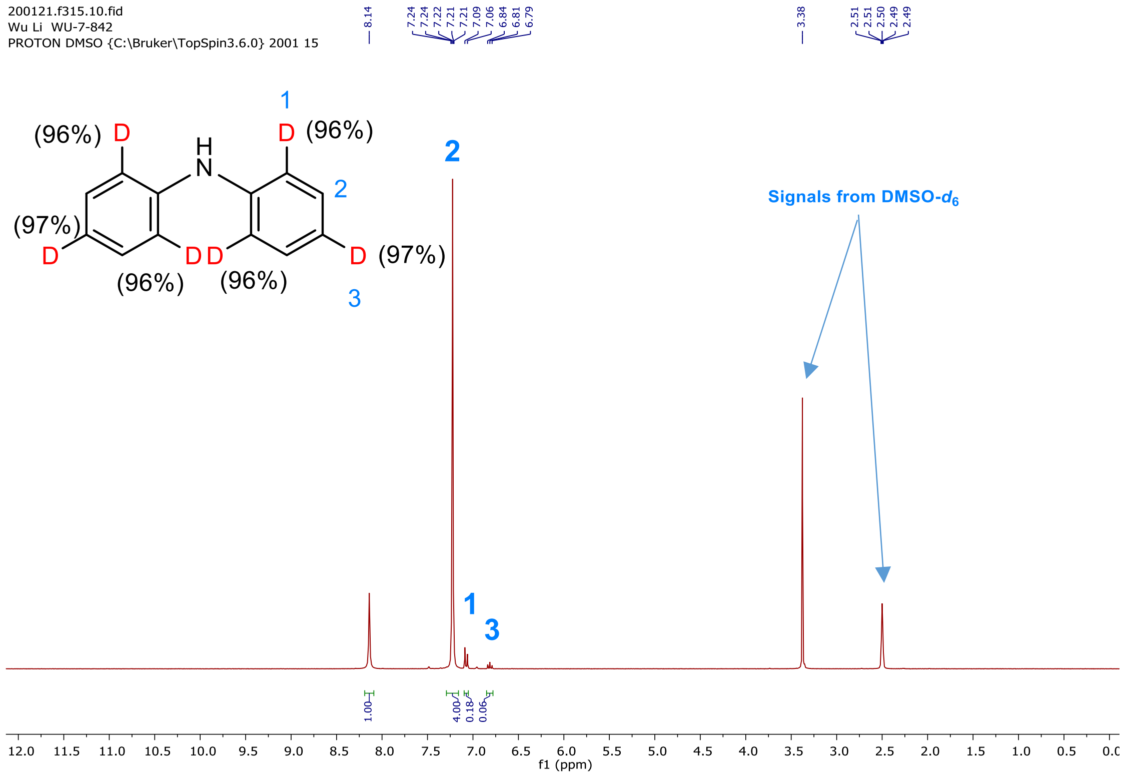

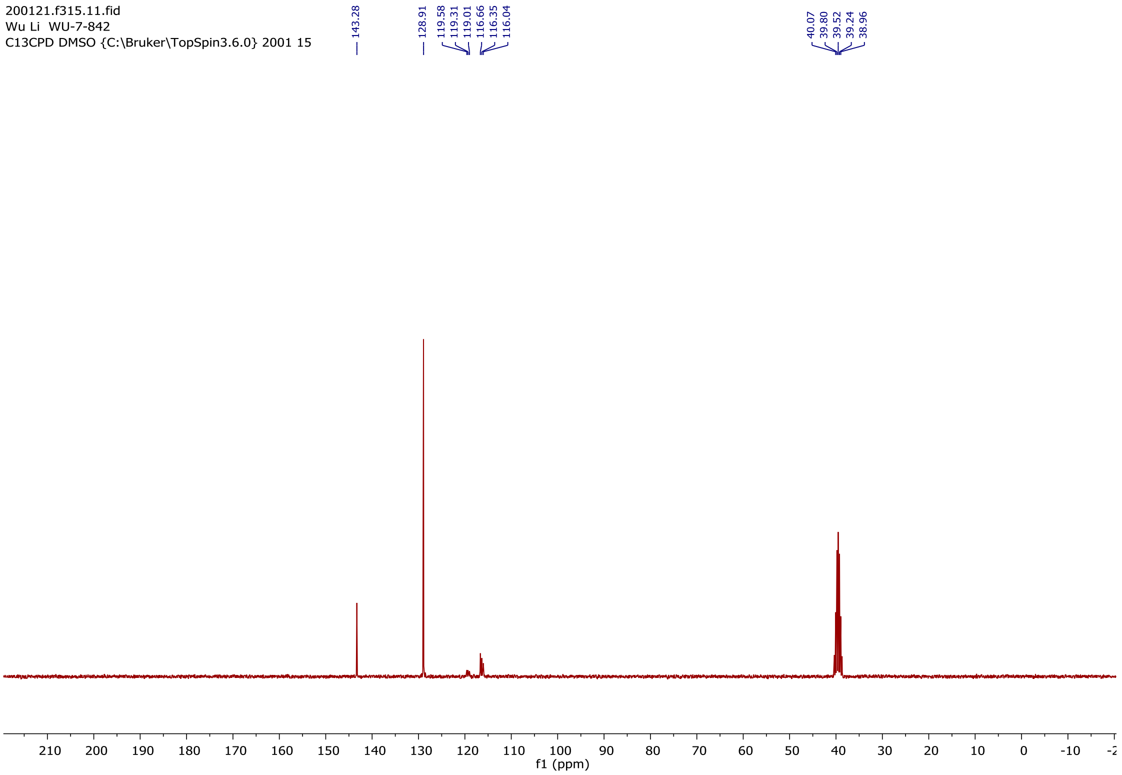

^{1}H NMR for 28a:

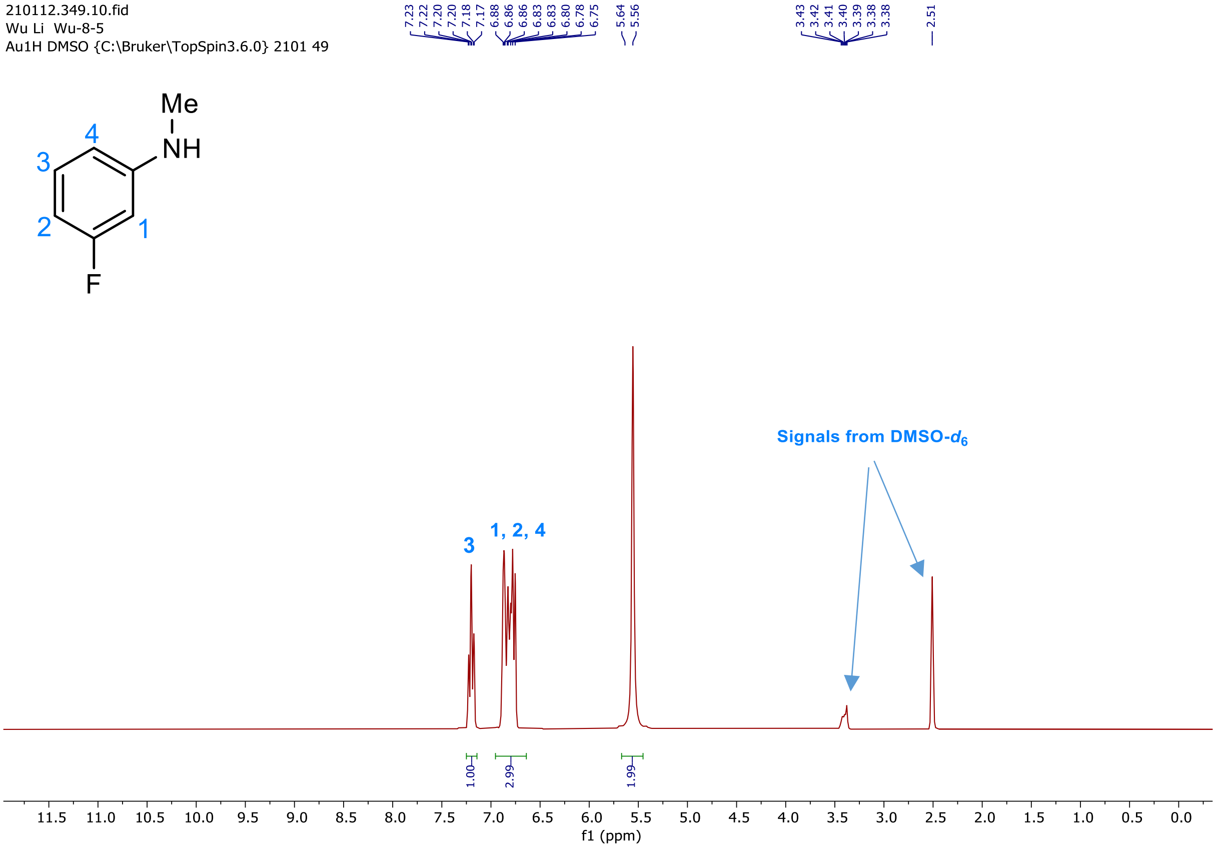

In $CDCl_3$:

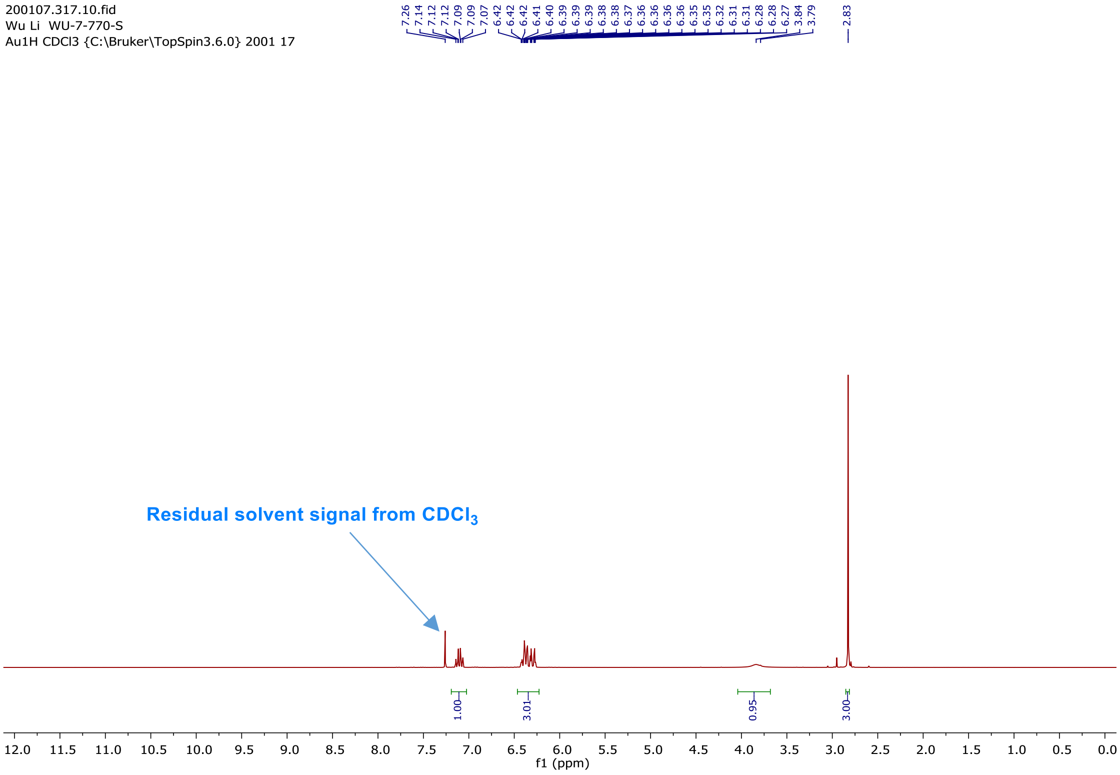

Original spectra for 28b:

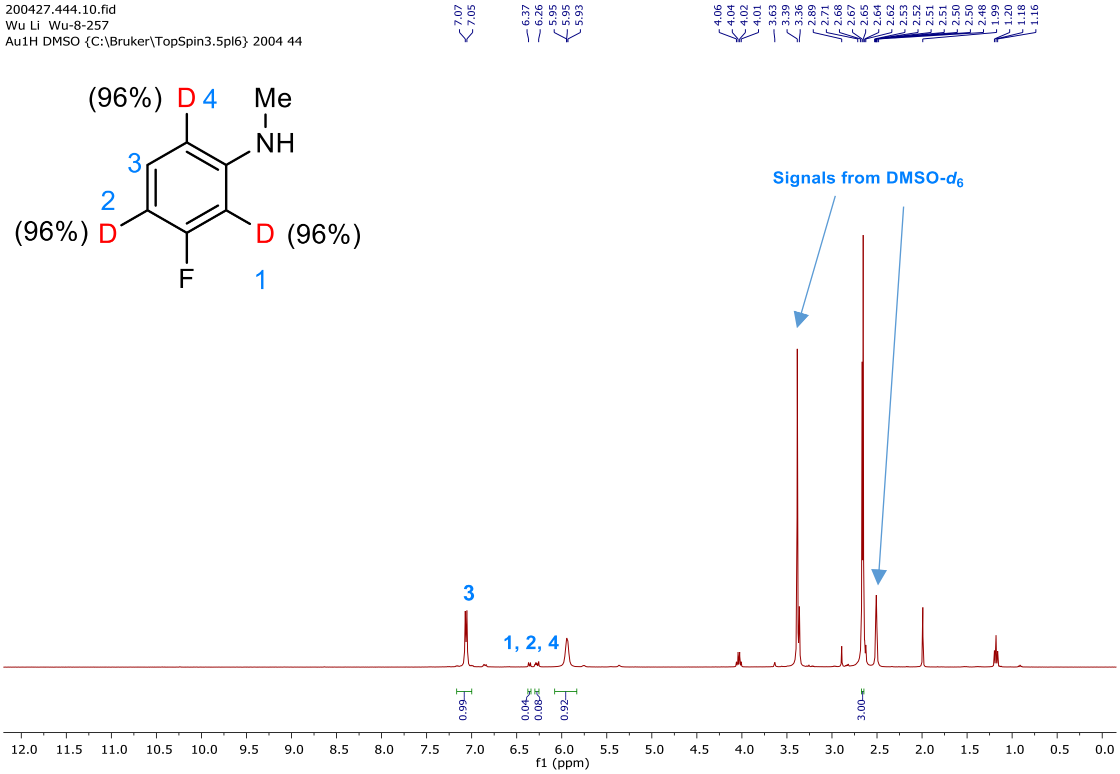

200427.444.11.fid
Wu Li Wu-8-257
Au13C DMSO {C:\Bruker\TopSpin3.5pl6} 2004 44

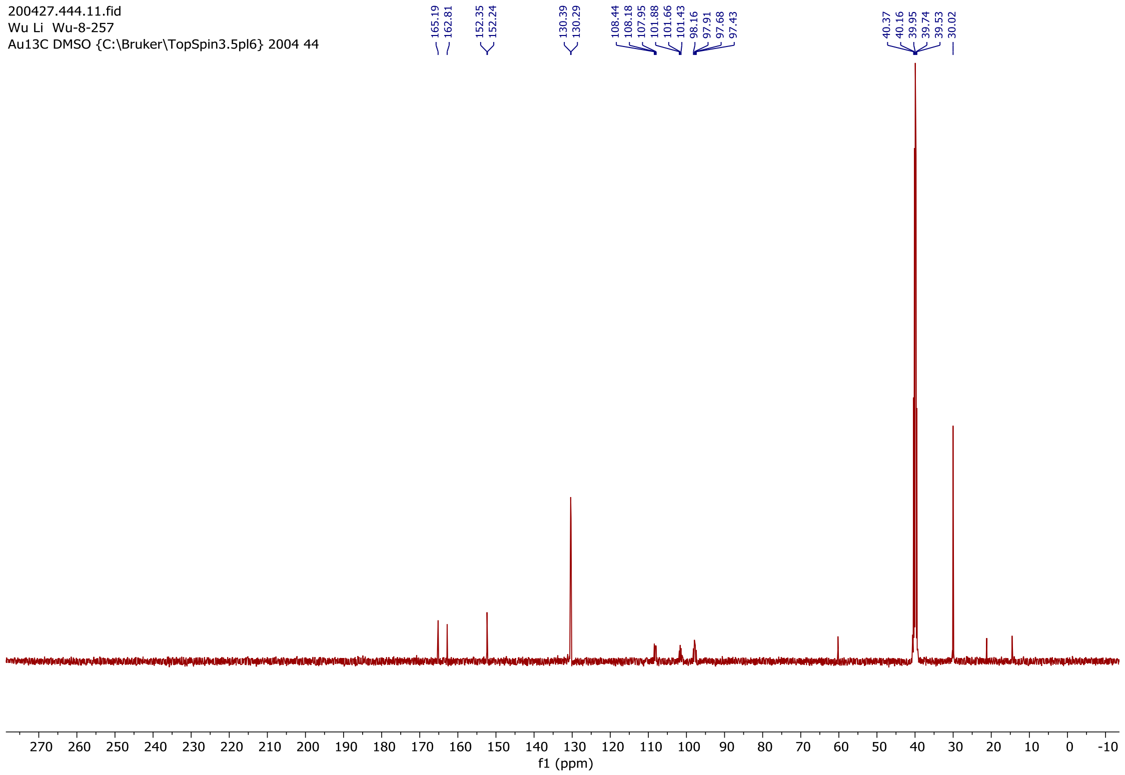

200107.f335.12.fid
Wu Li WU-7-770
19F(H-entk) CDCl3 {C:\Bruker\TopSpin3.6.0} 2001 35

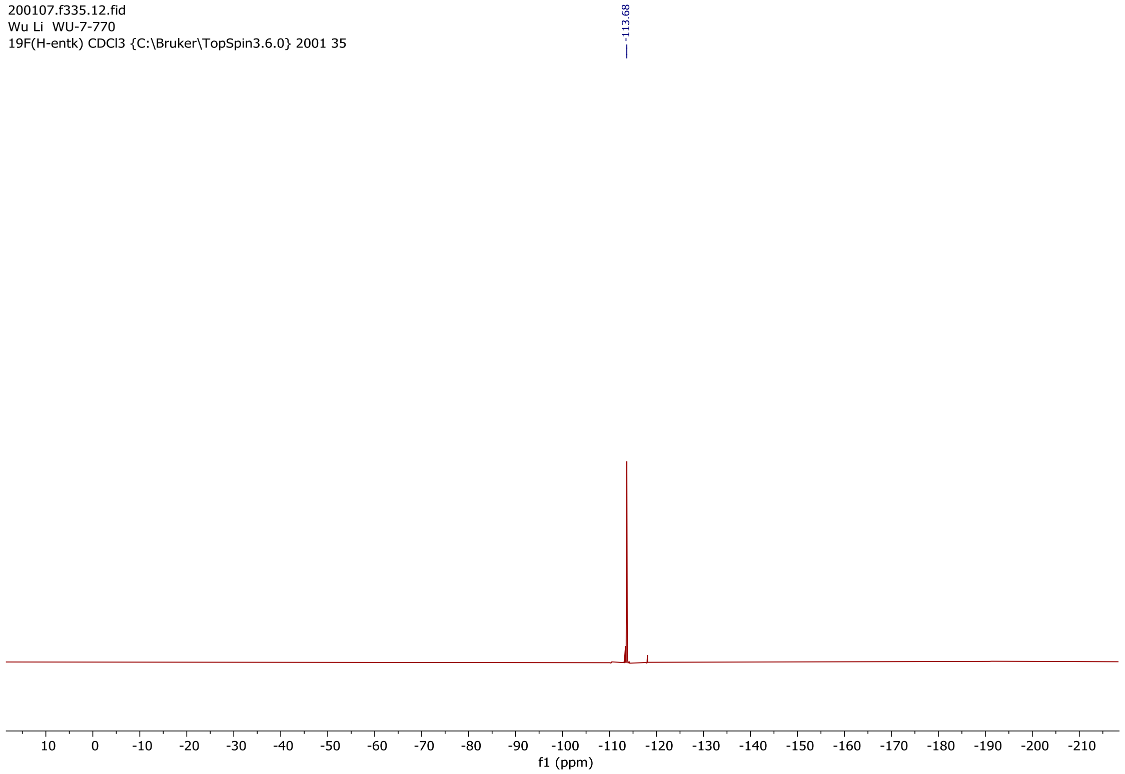

^{1}H NMR for 29a:

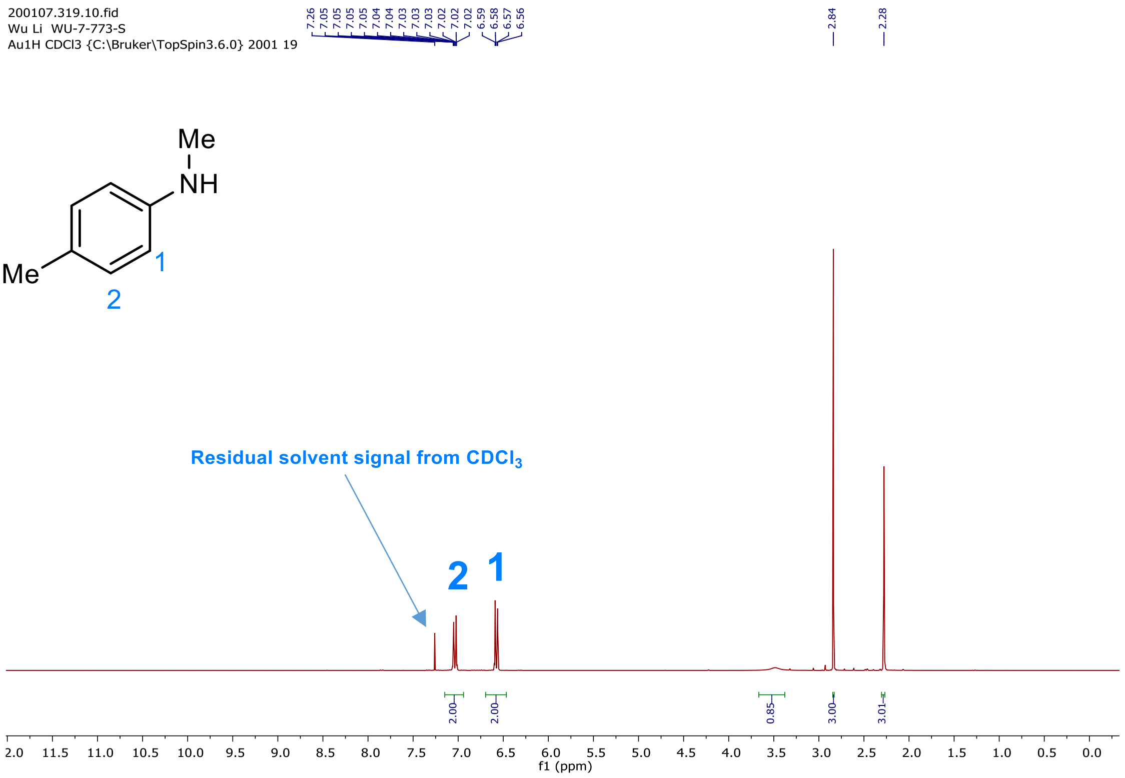

Original spectra for 29b:

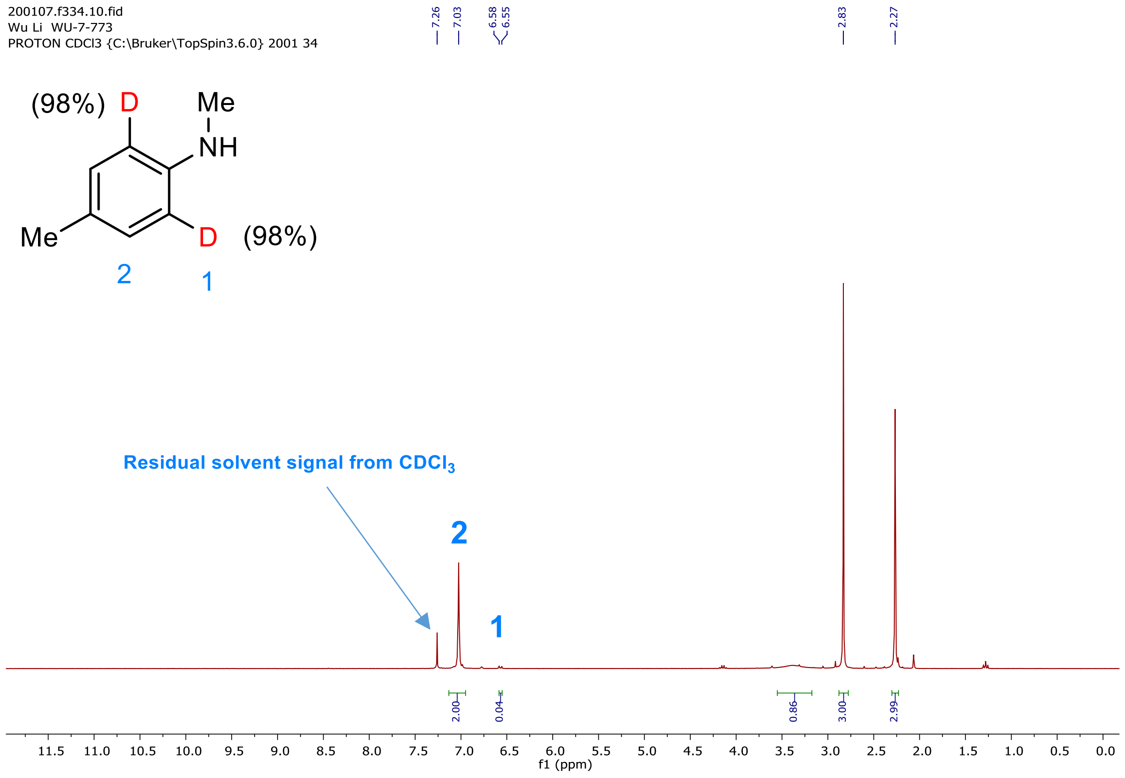

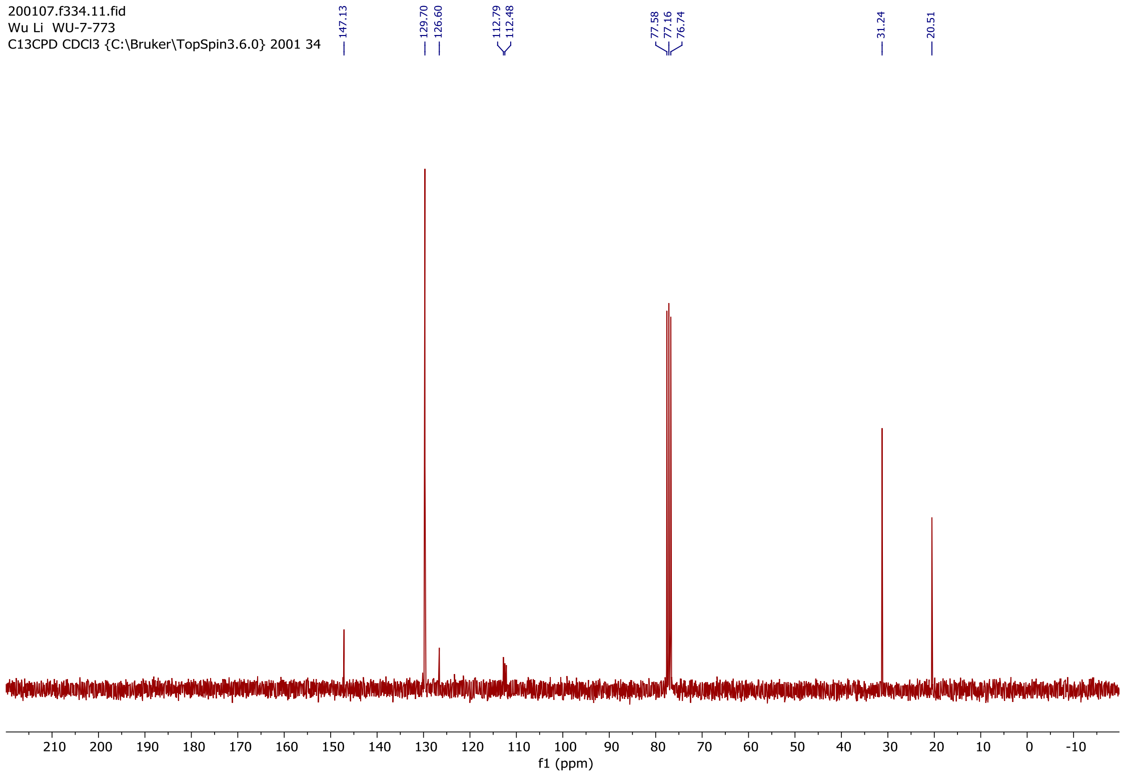

^{1}H NMR for 30a:

In DMSO-d_6:

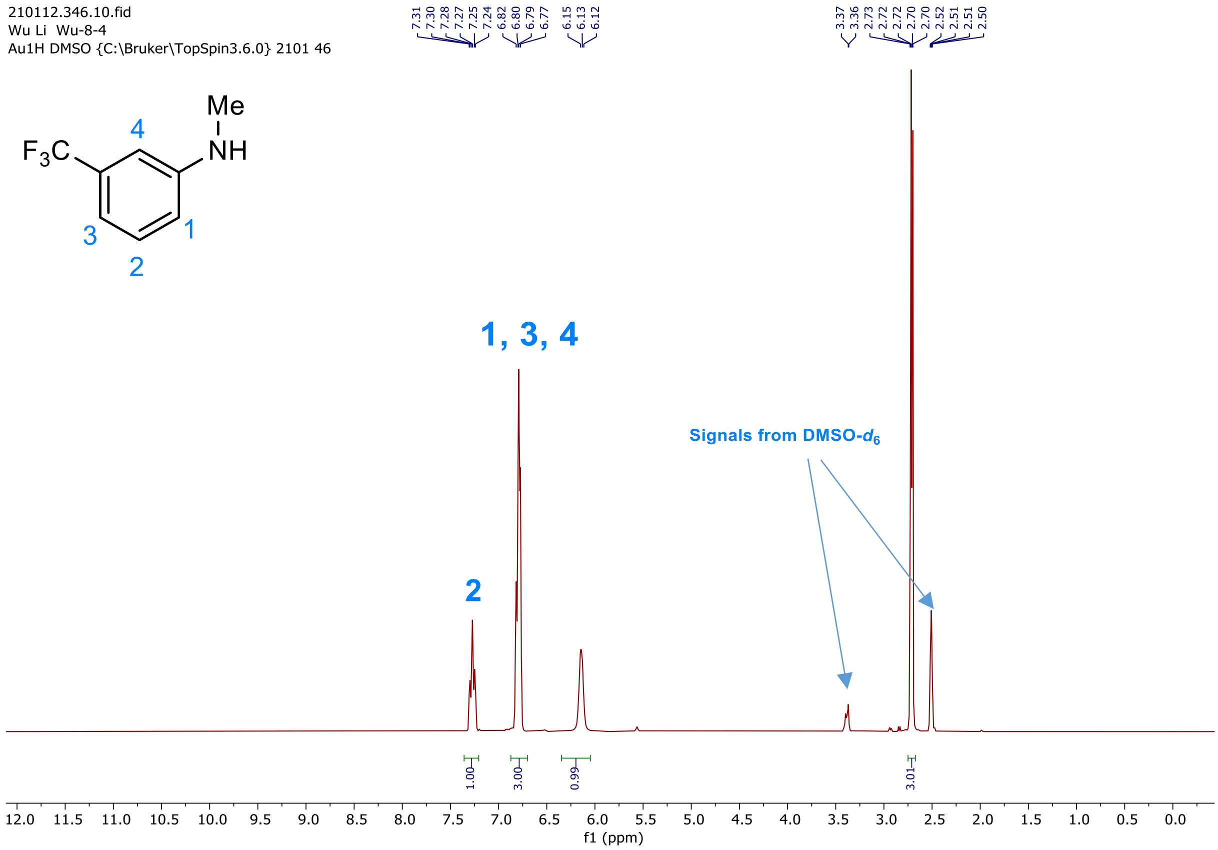

In $CDCl_3$:

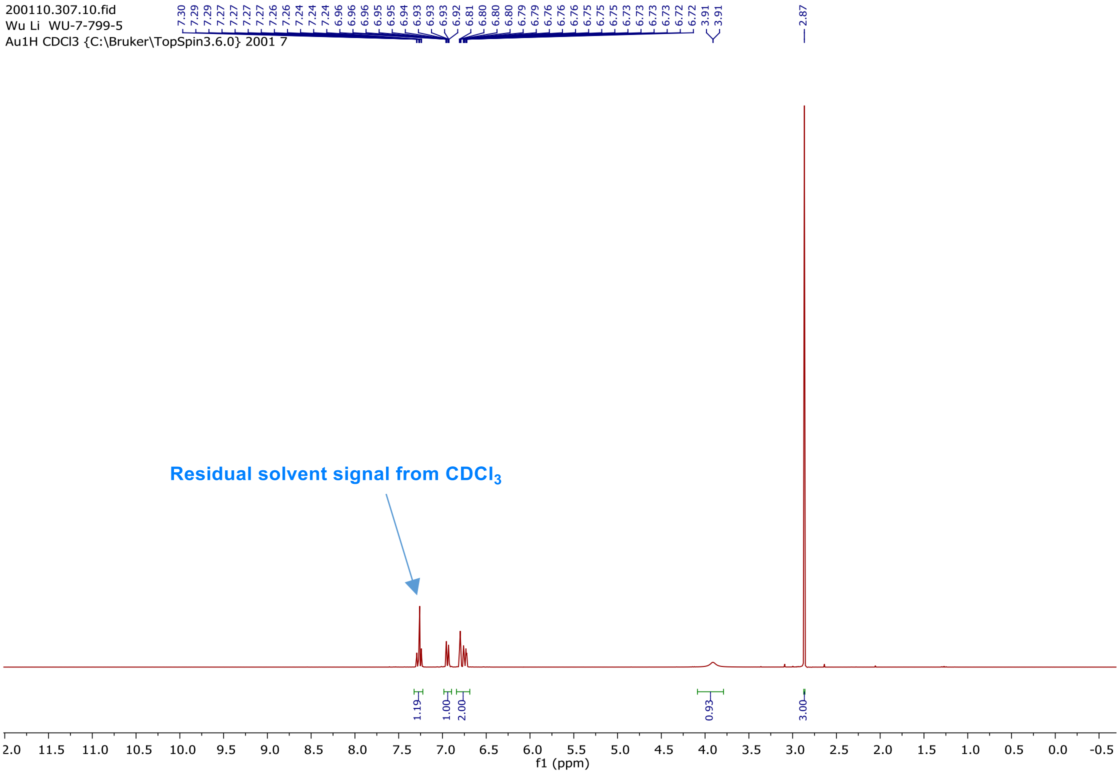

Original spectra for 30b:

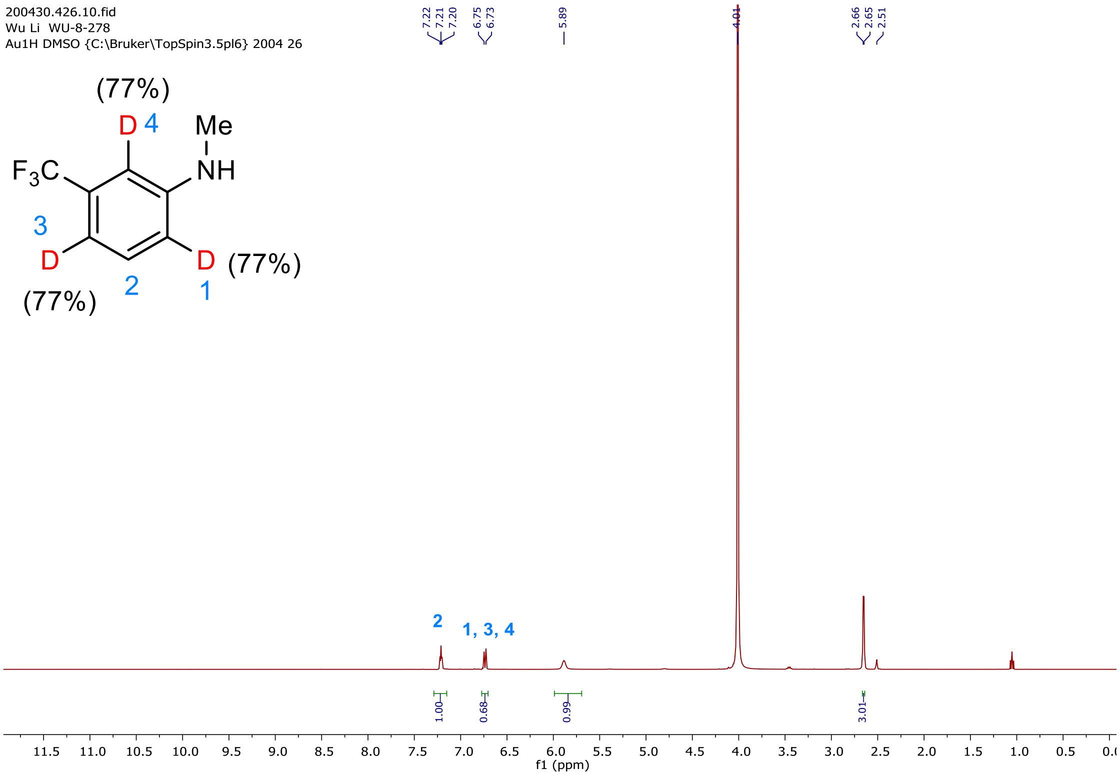

200430.426.11.fid
Wu Li WU-8-278
Au13C DMSO {C:\Bruker\TopSpin3.5pl6} 2004 26

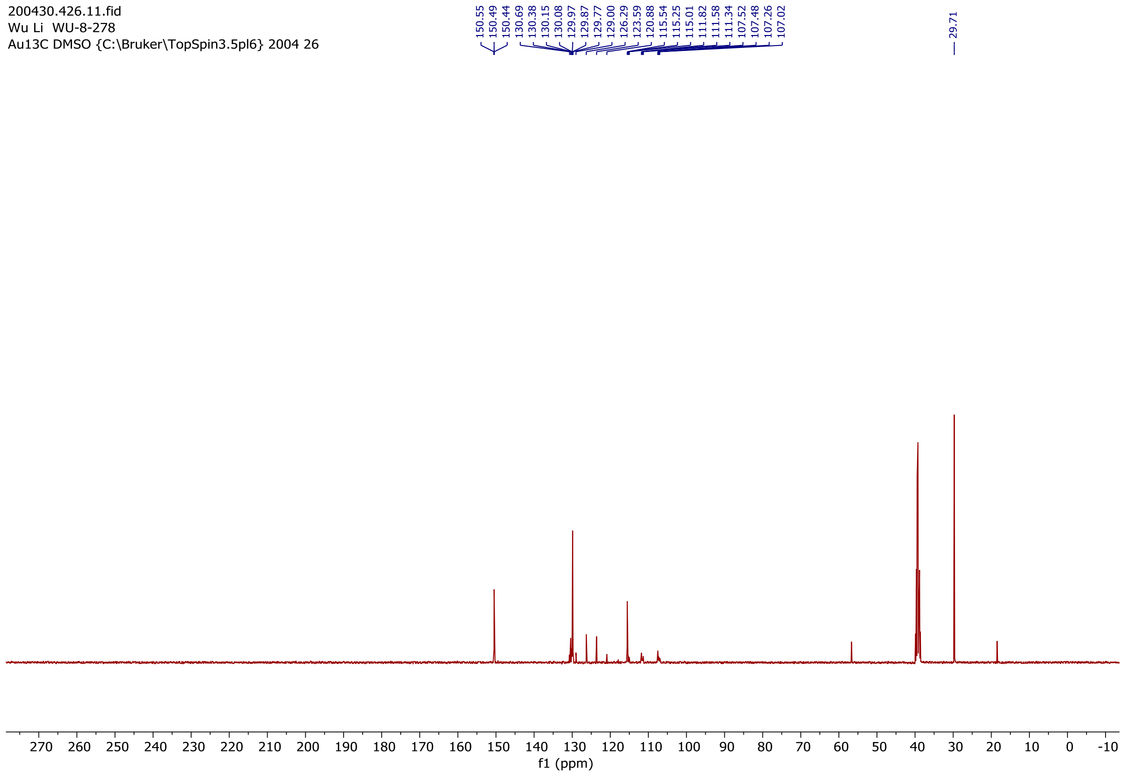

200428.f329.12.fid
Wu Li WU-8-278
19F(H-entk) DMSO {C:\Bruker\TopSpin3.6.0} 2004 29

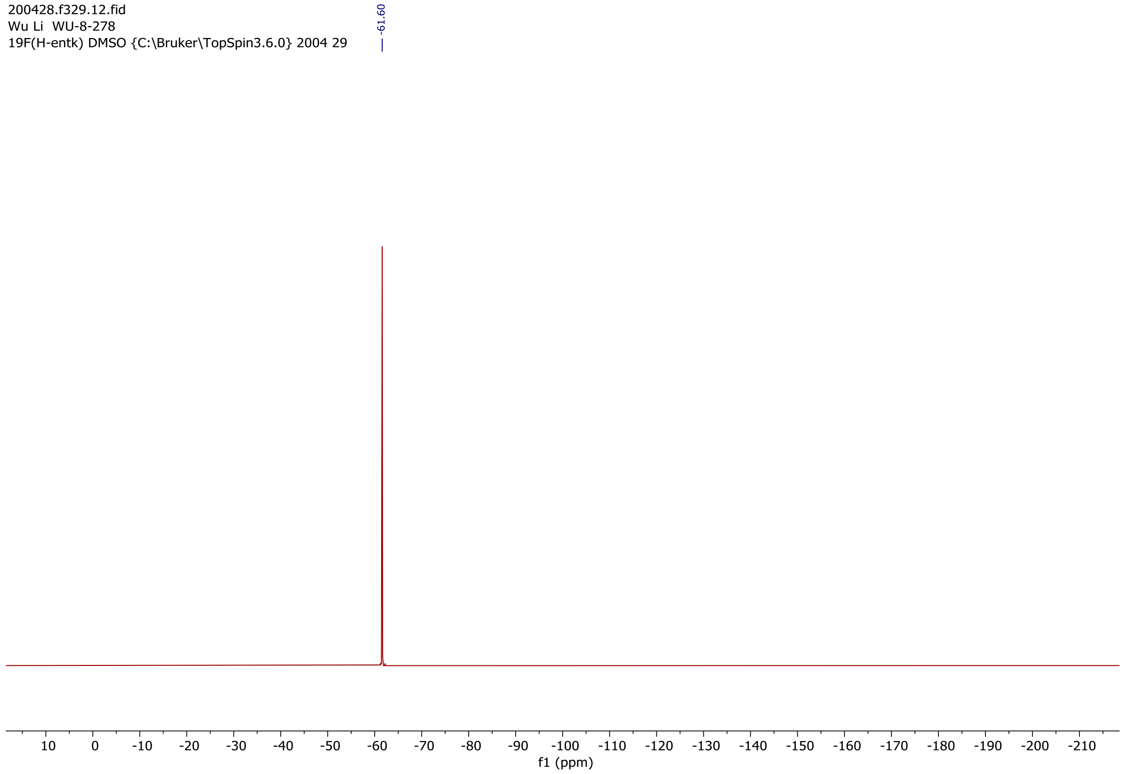

^{1}H NMR for 31a:

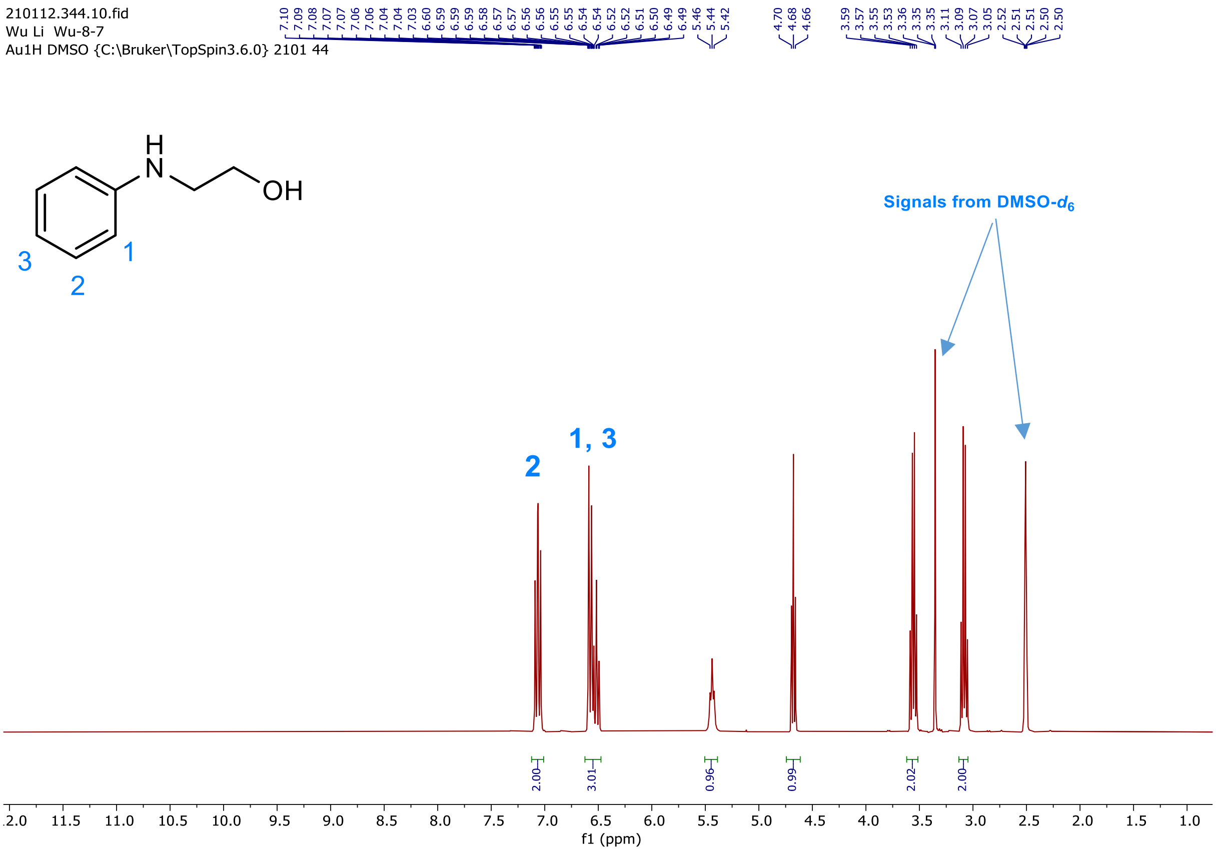

Original spectra for 31b:

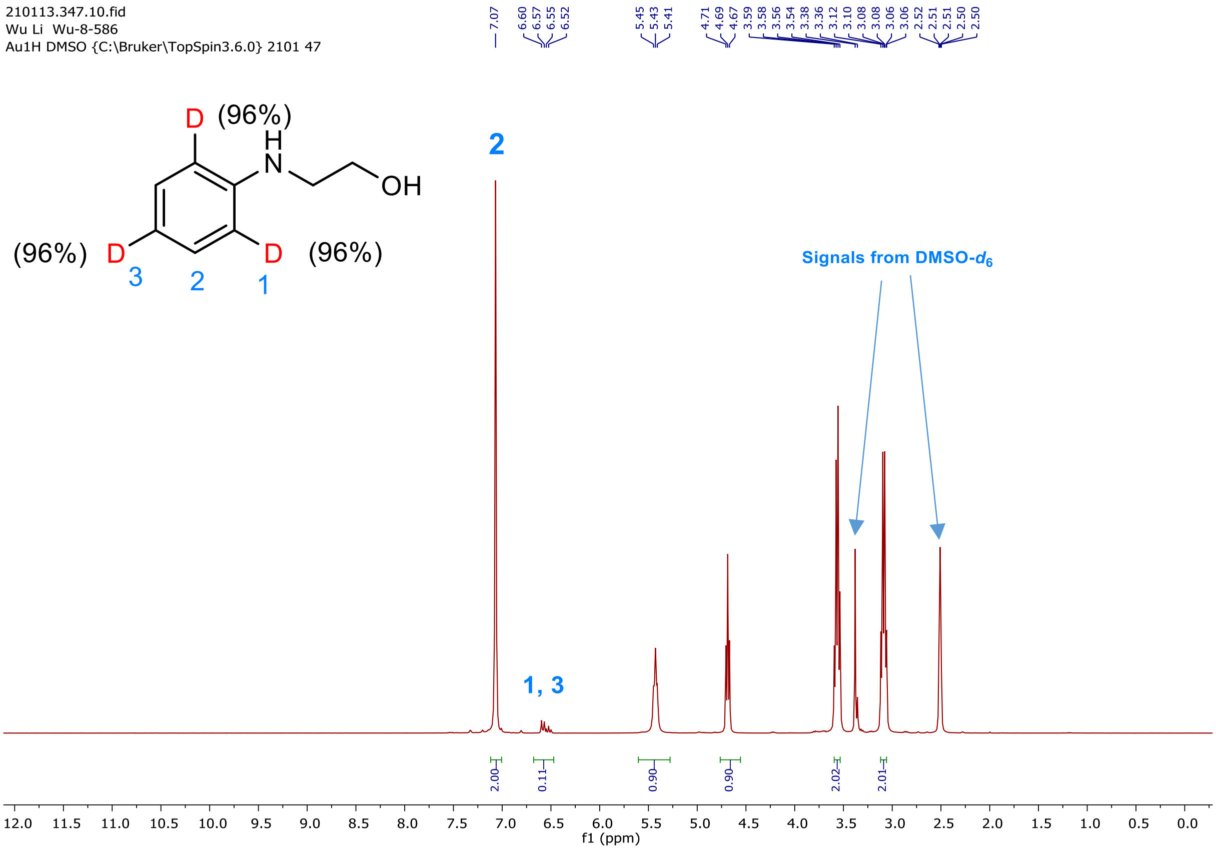

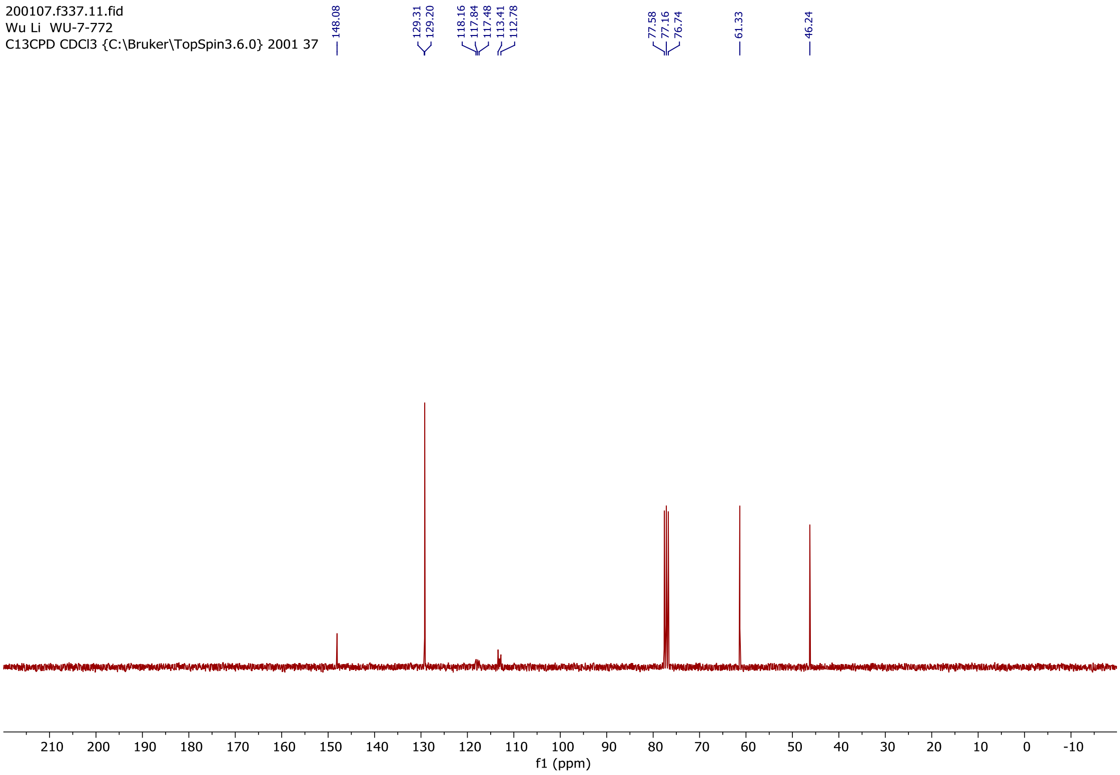

^{1}H NMR for 32a:

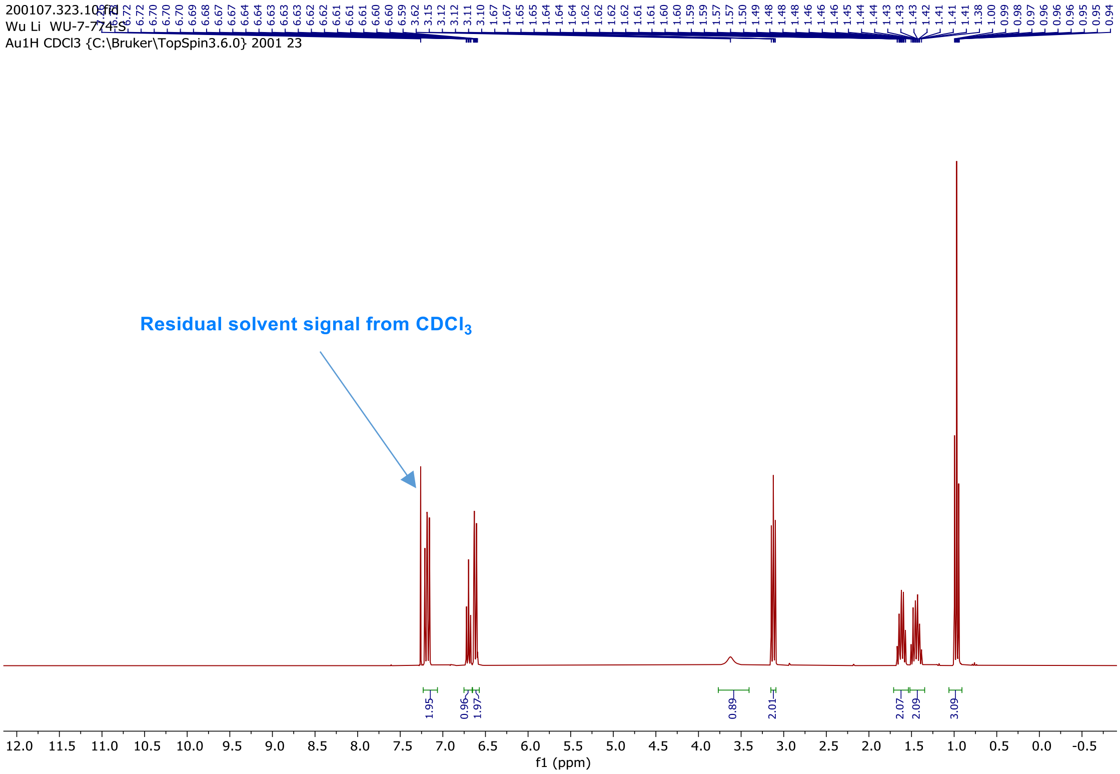

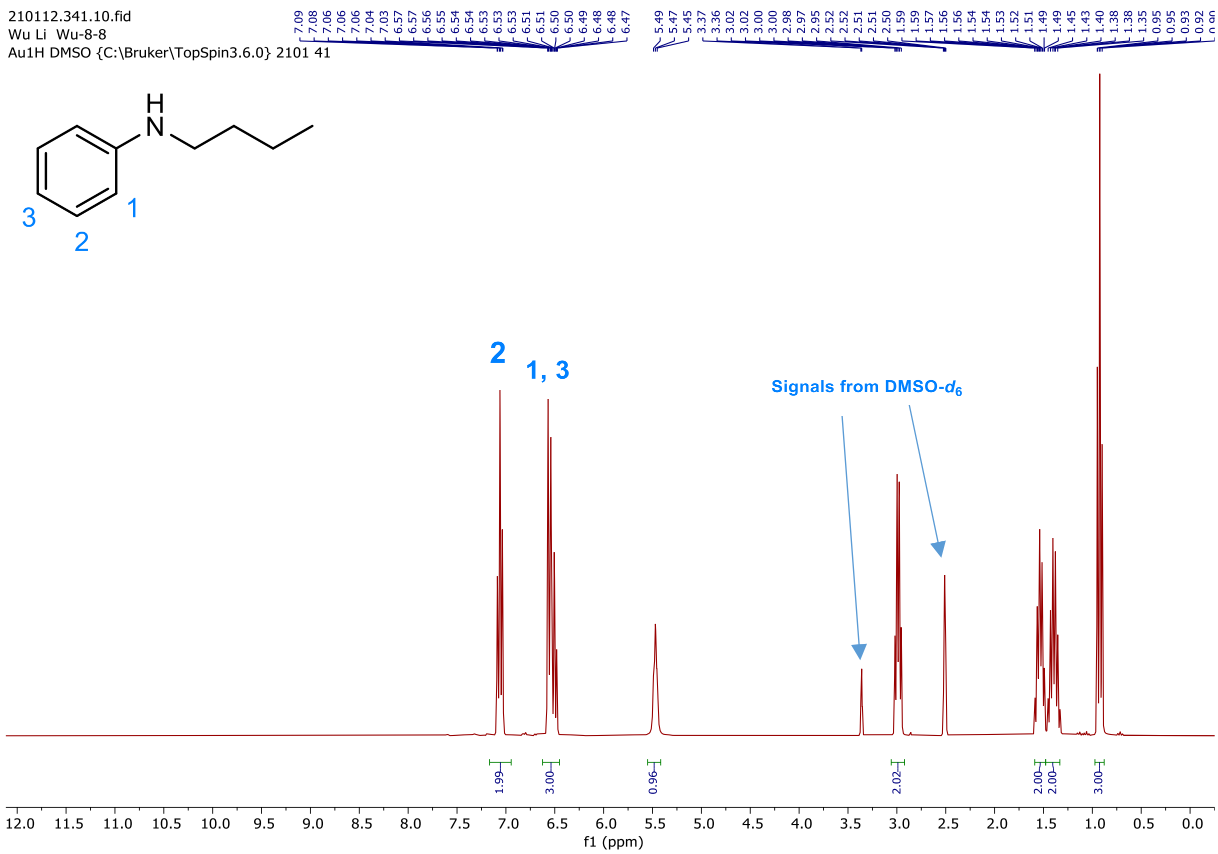

Original spectra for 32b:

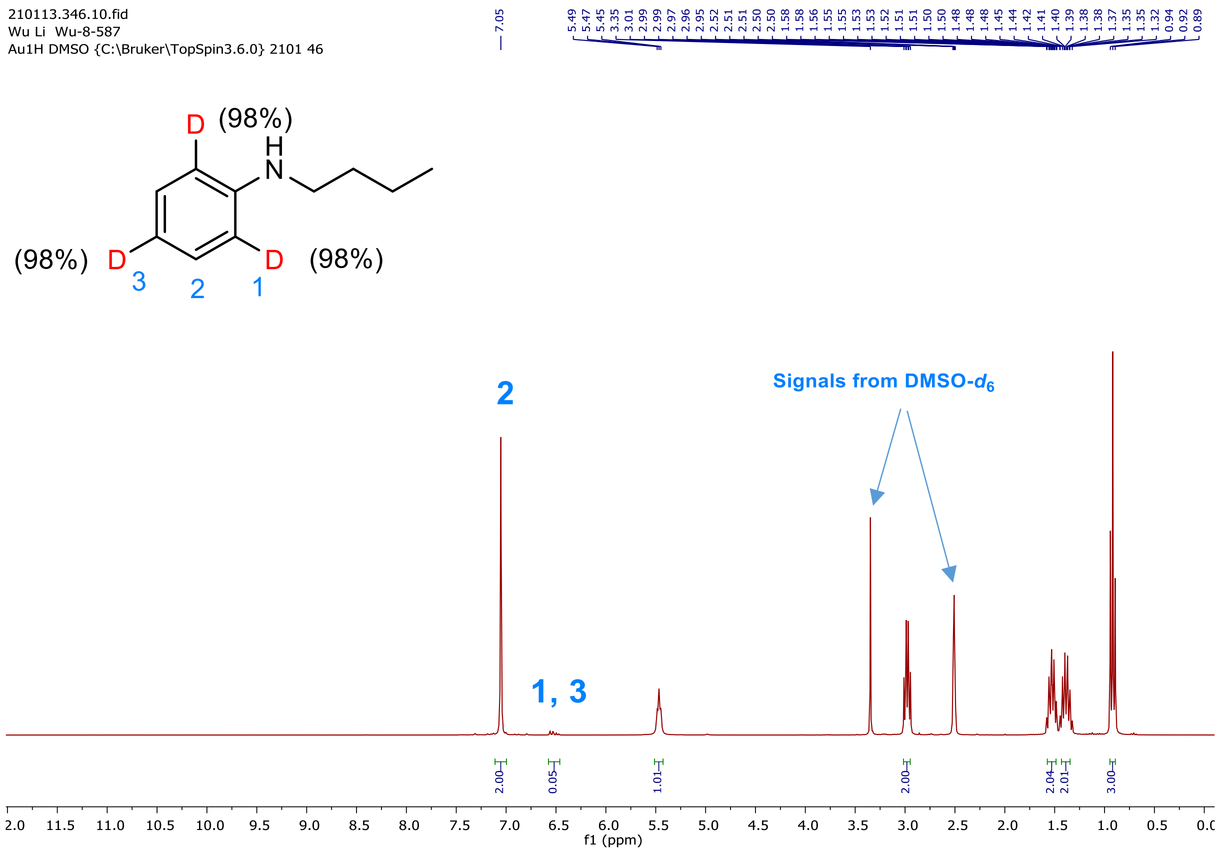

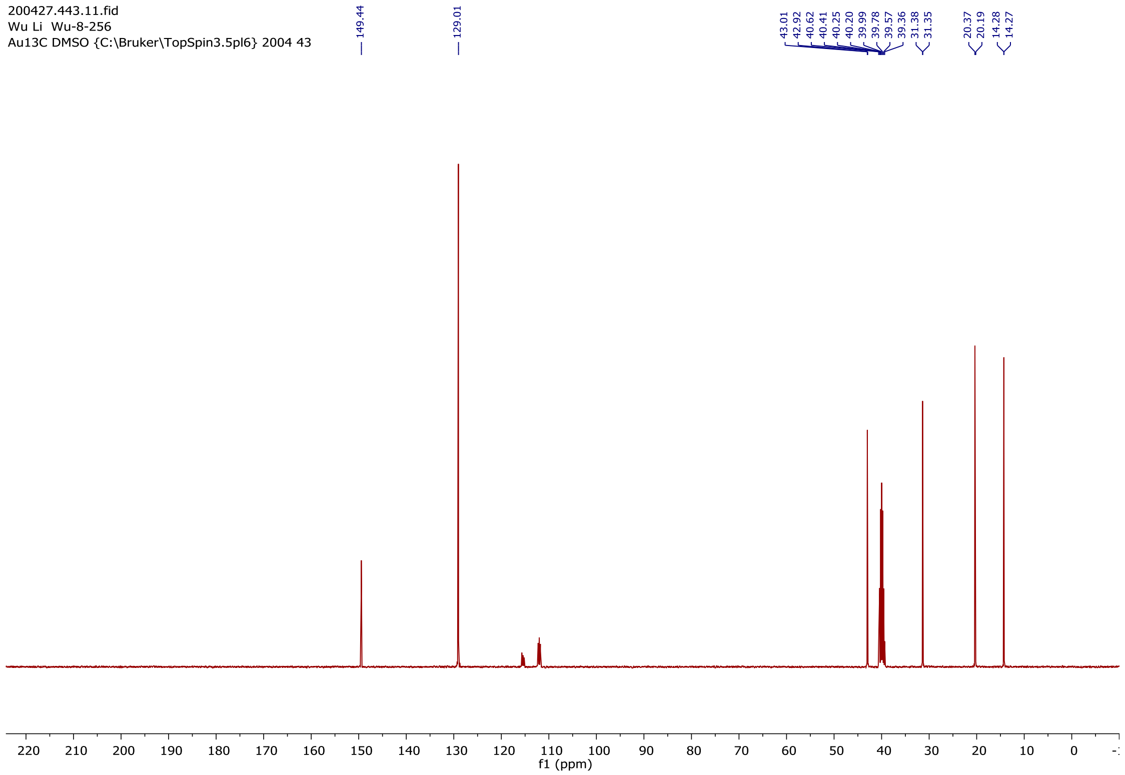

^{1}H NMR for 33a:

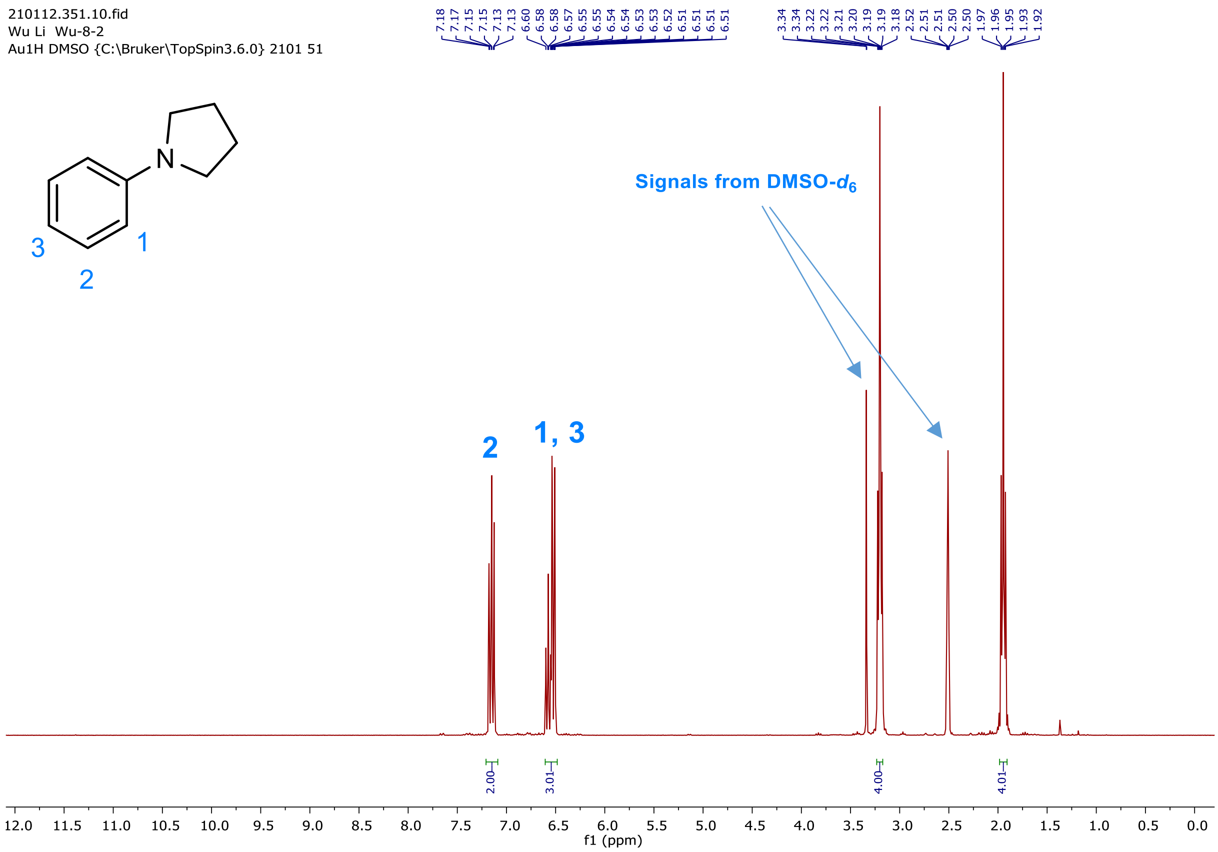

Original spectra for 33b:

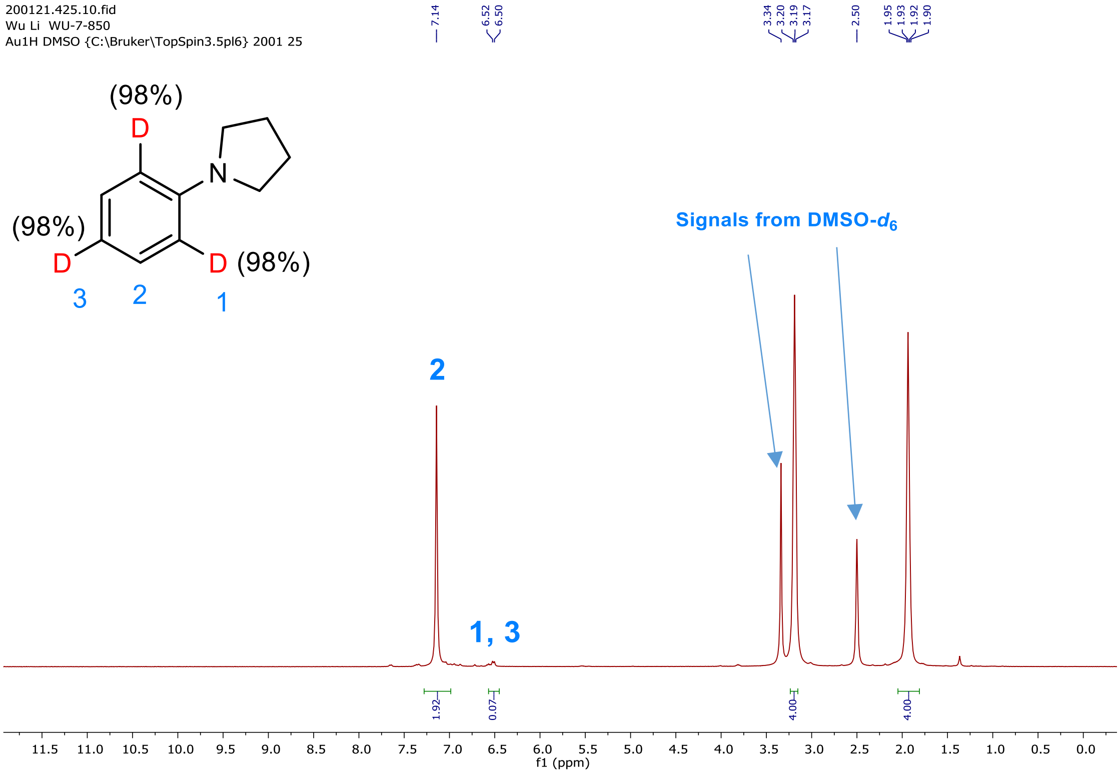

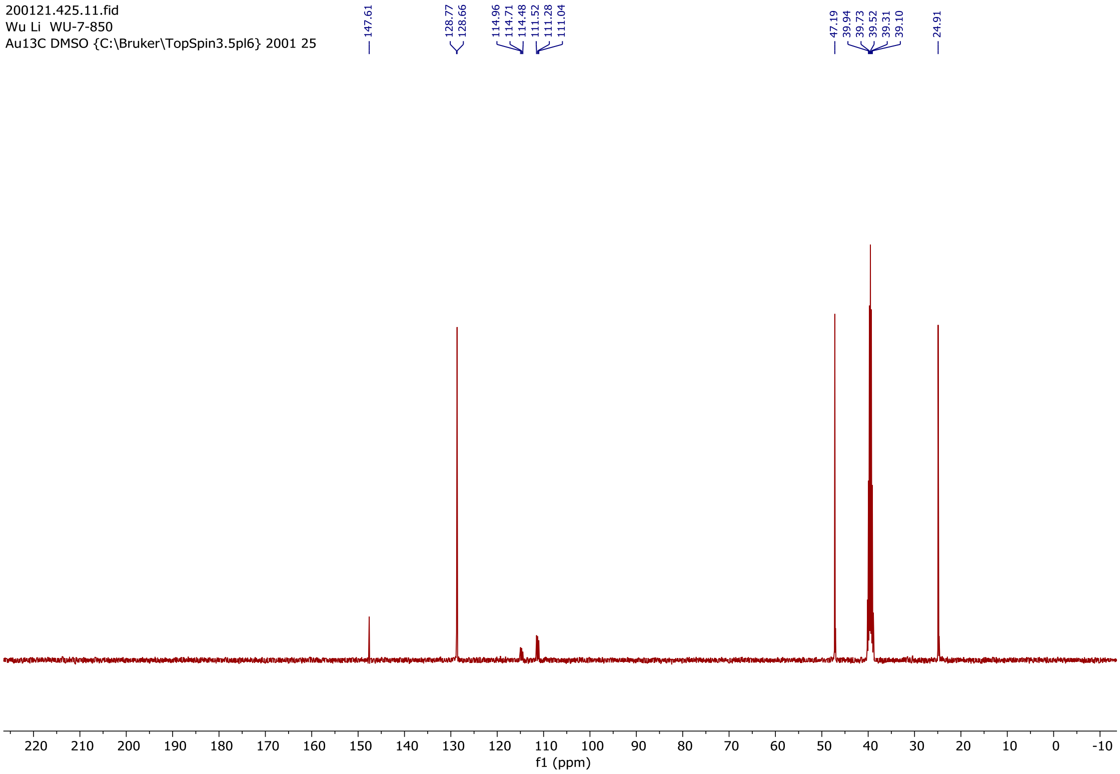

^{1}H NMR for 34a:

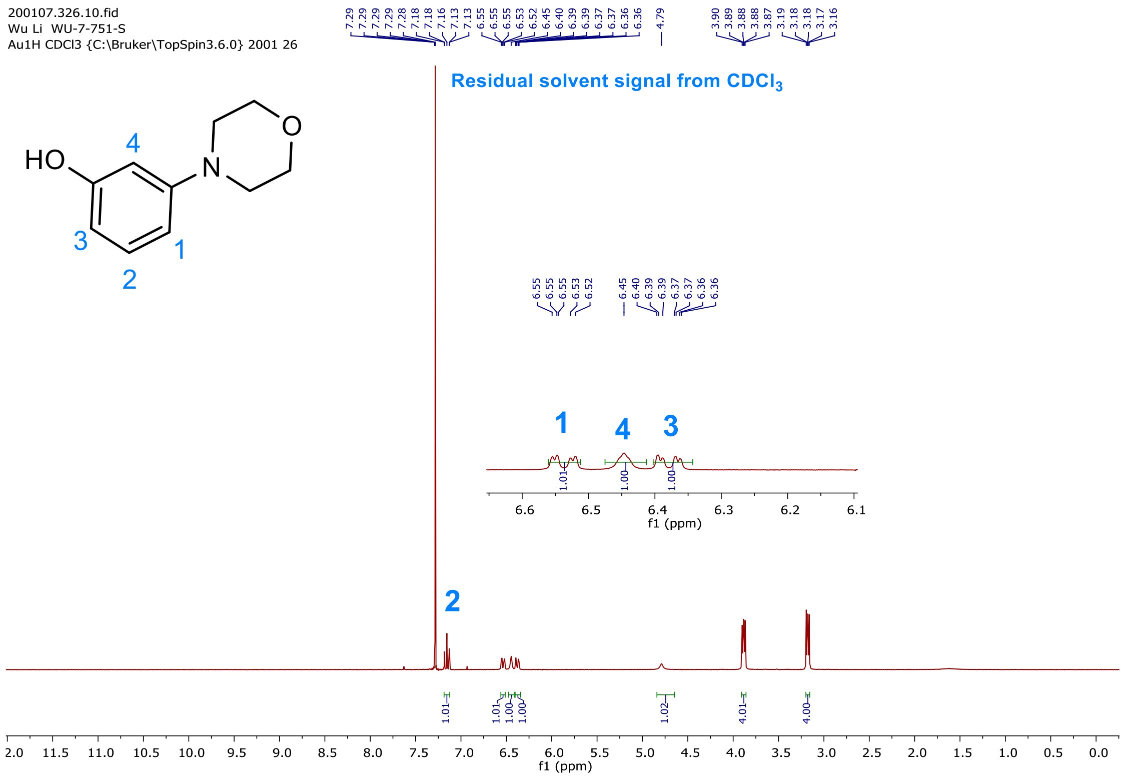

Original spectra for 34b:

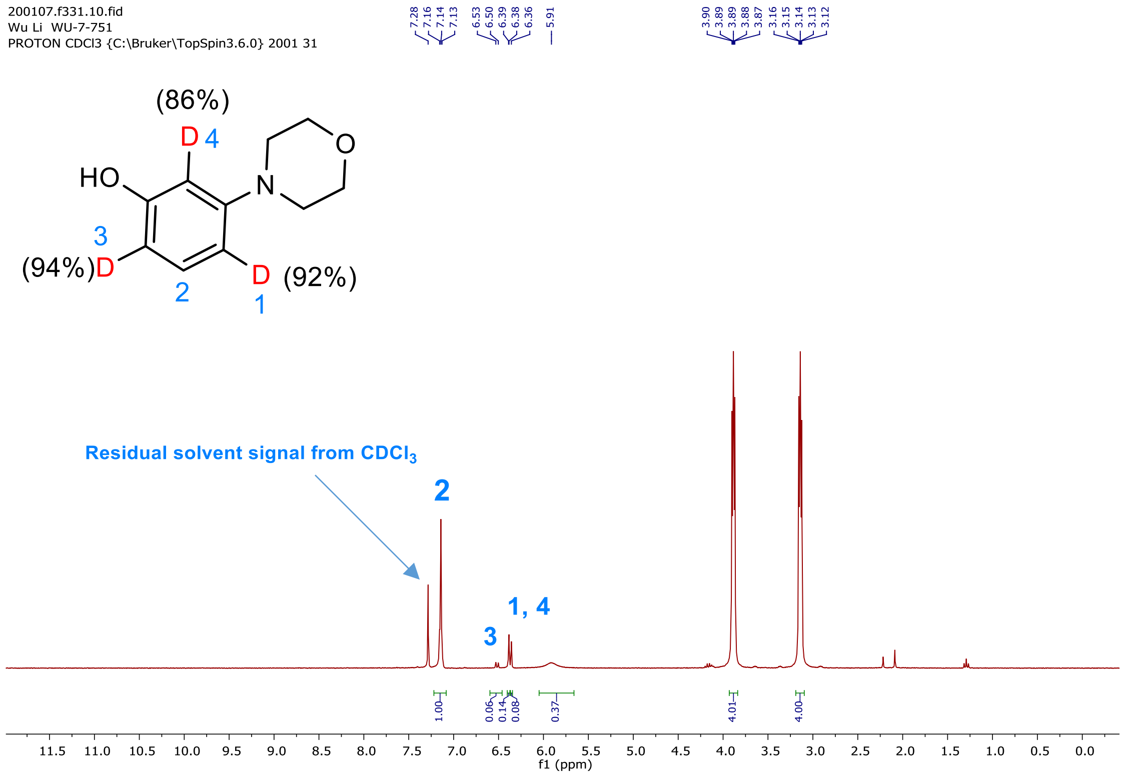

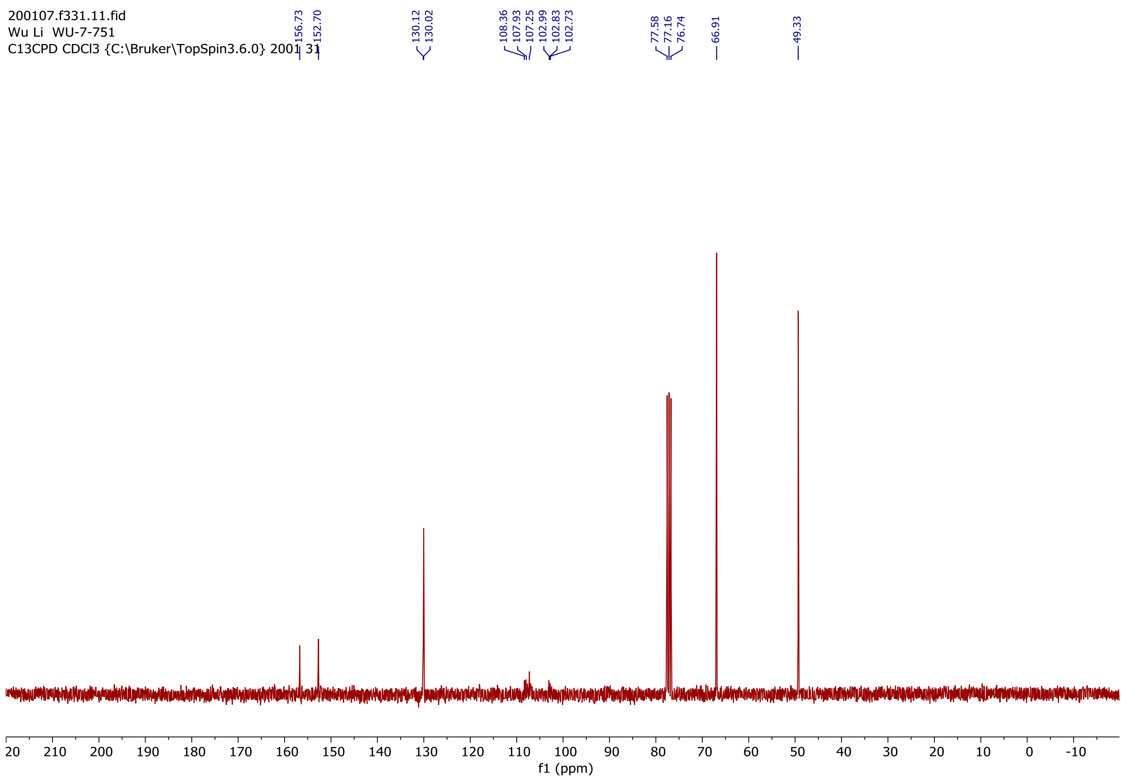

^{1}H NMR for 35a:

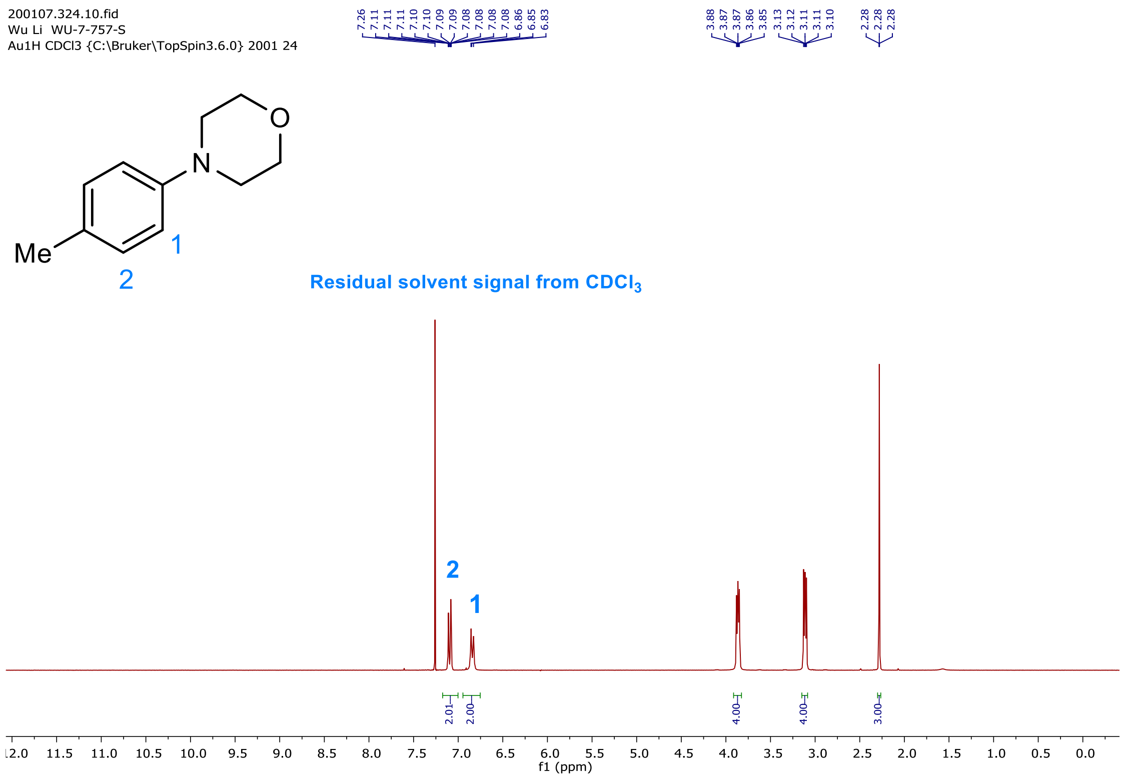

Original spectra for 35b:

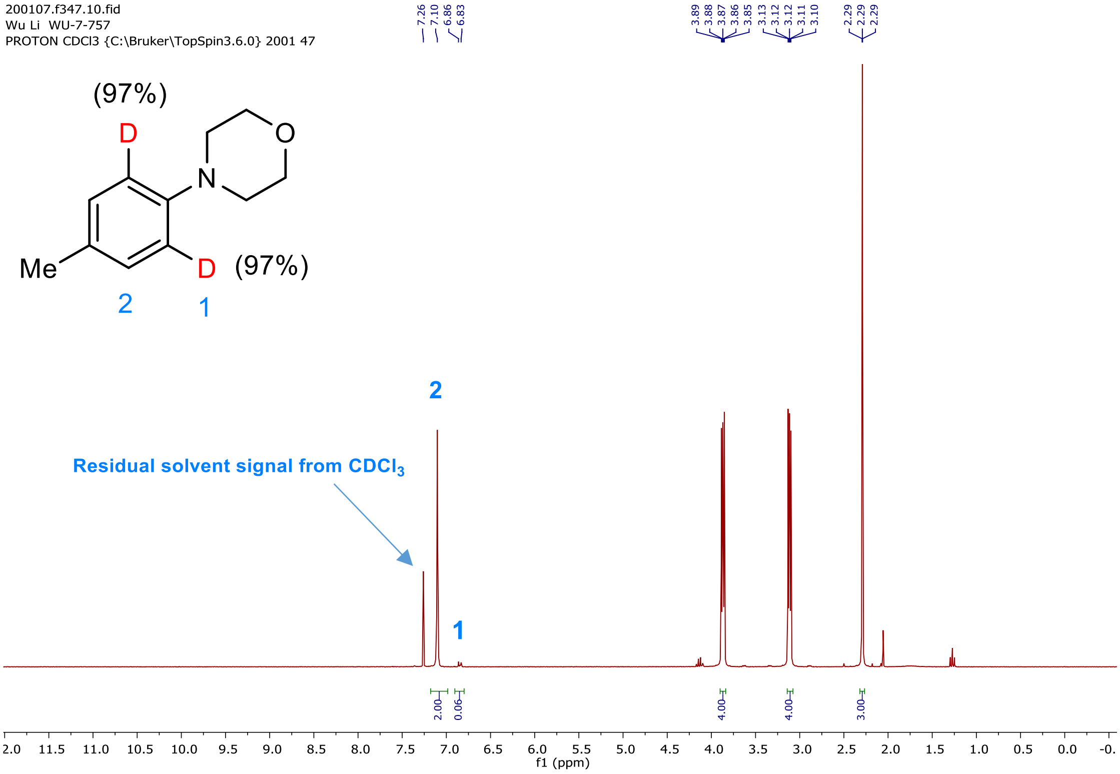

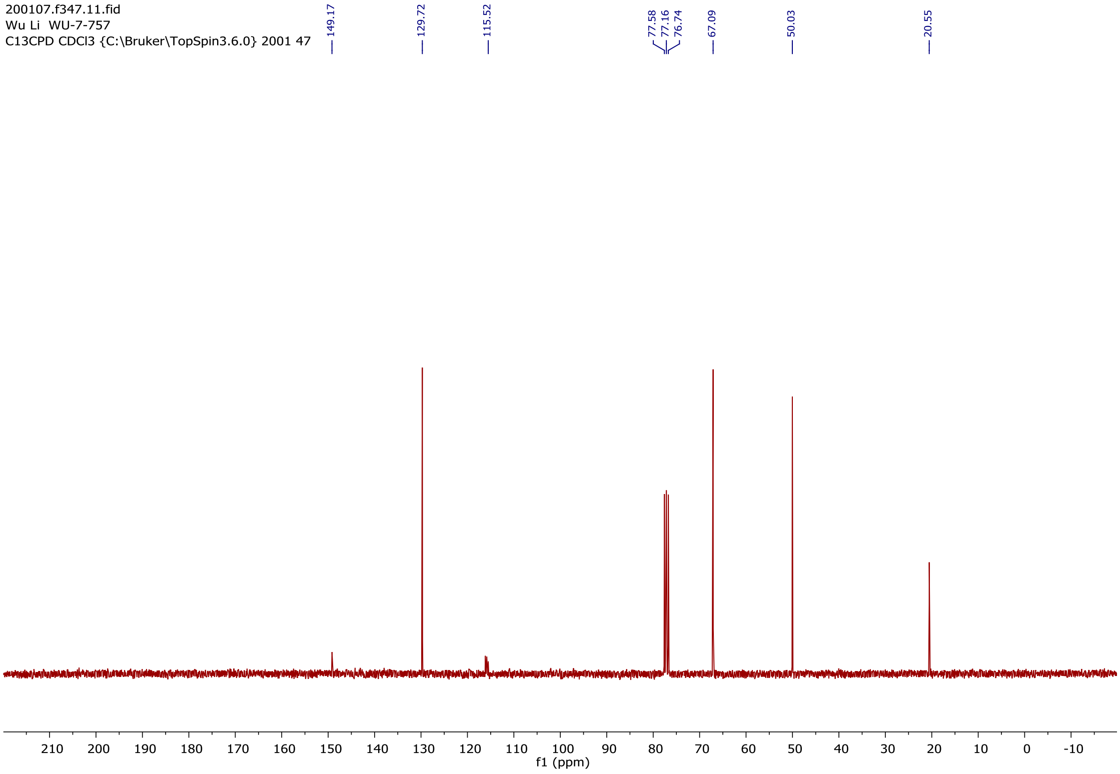

^{1}H NMR for 36a:

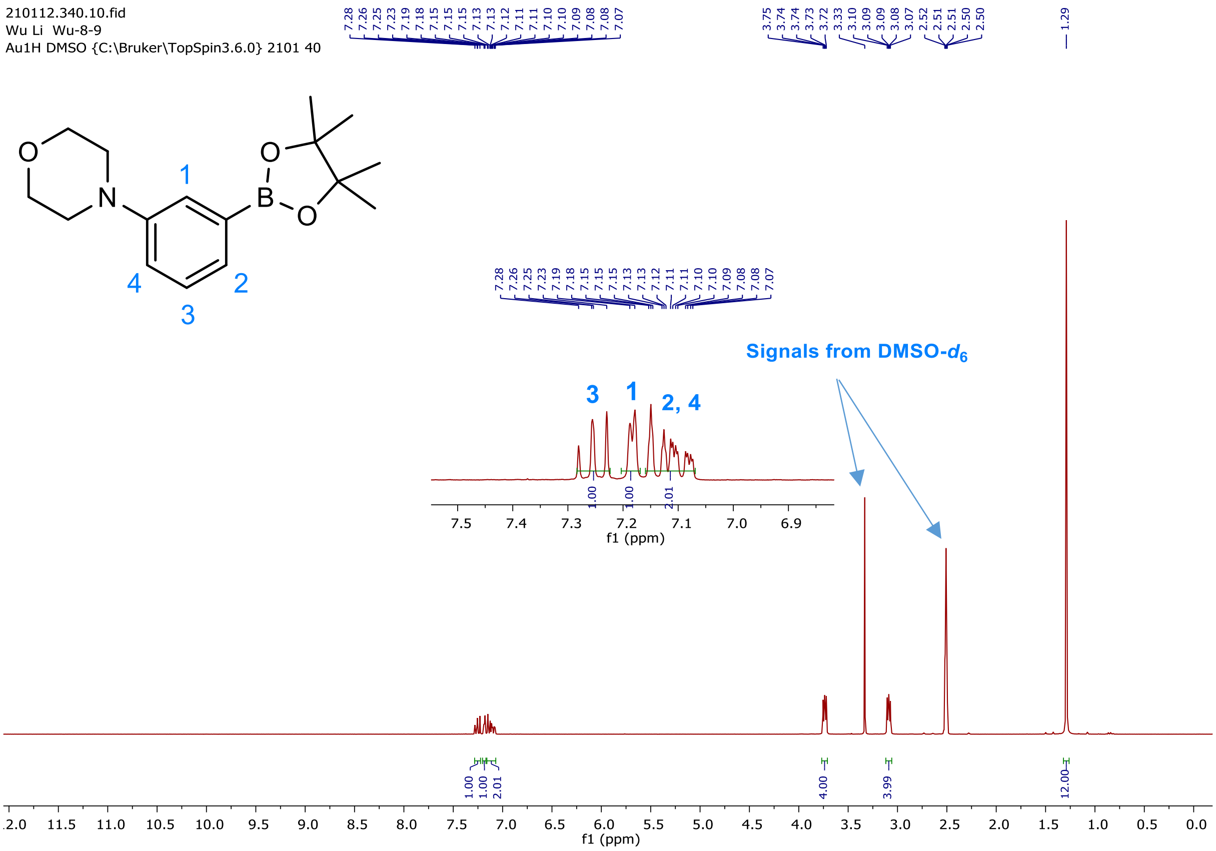

Original spectra for 36b:

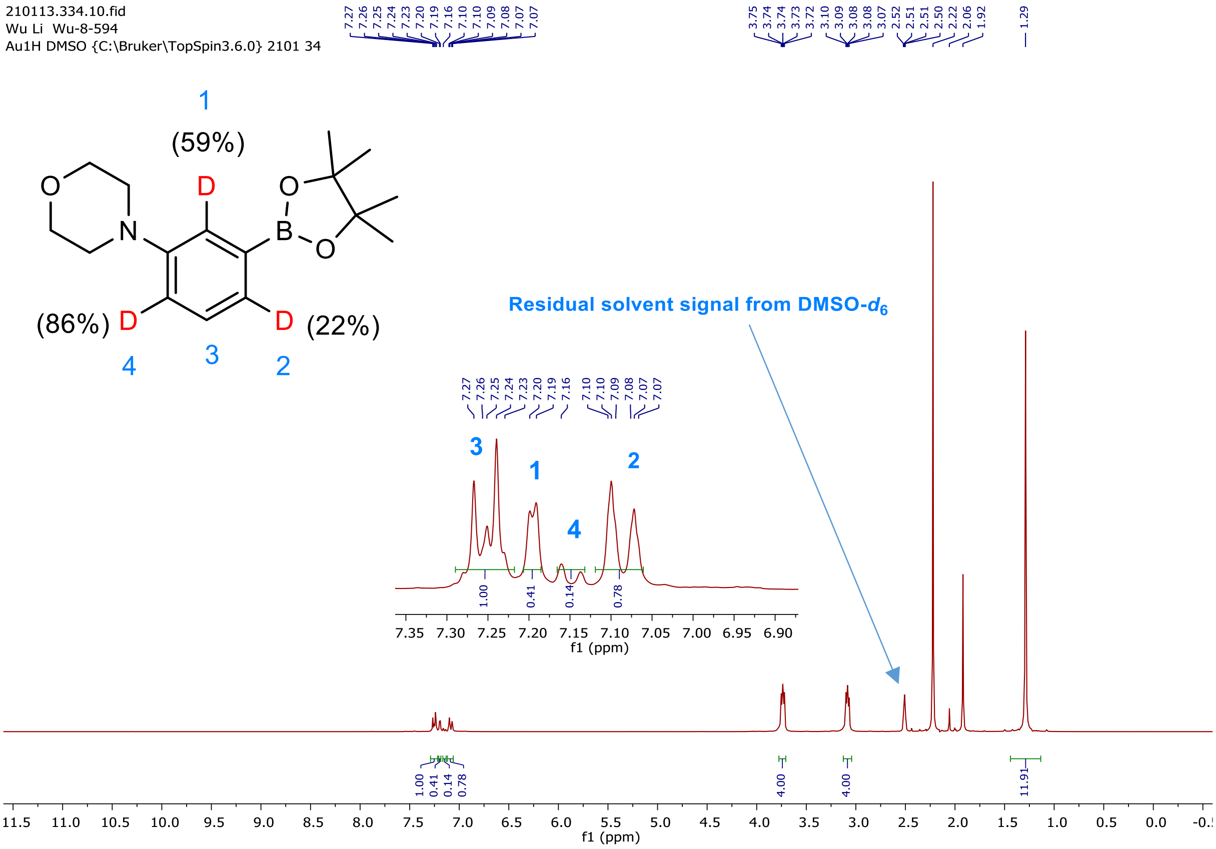

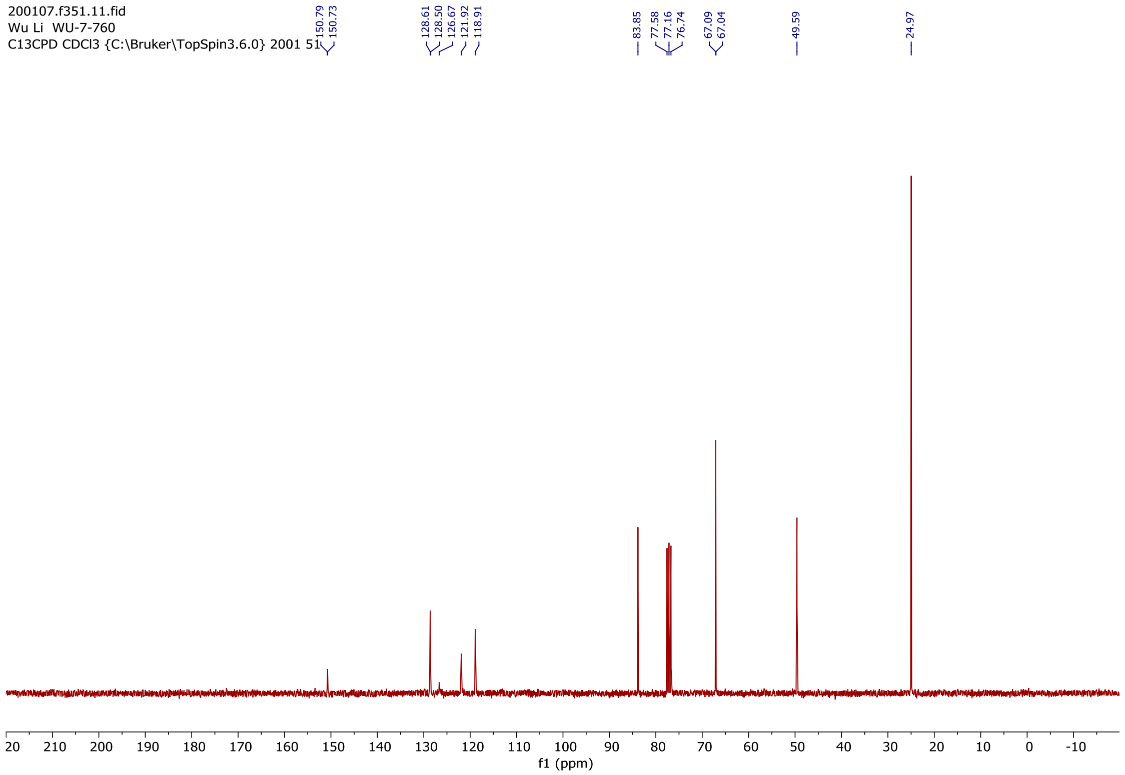

^{1}H NMR for 37a:

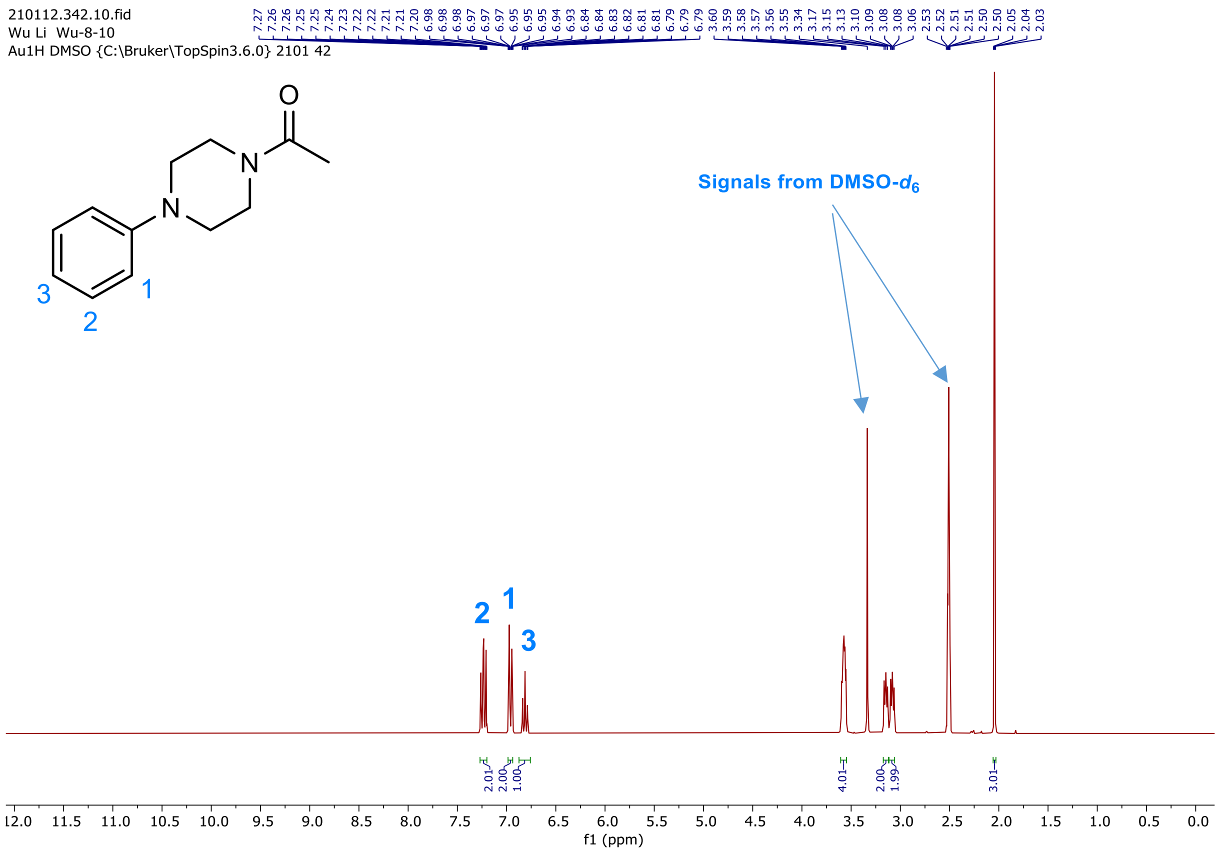

Original spectra for 37b:

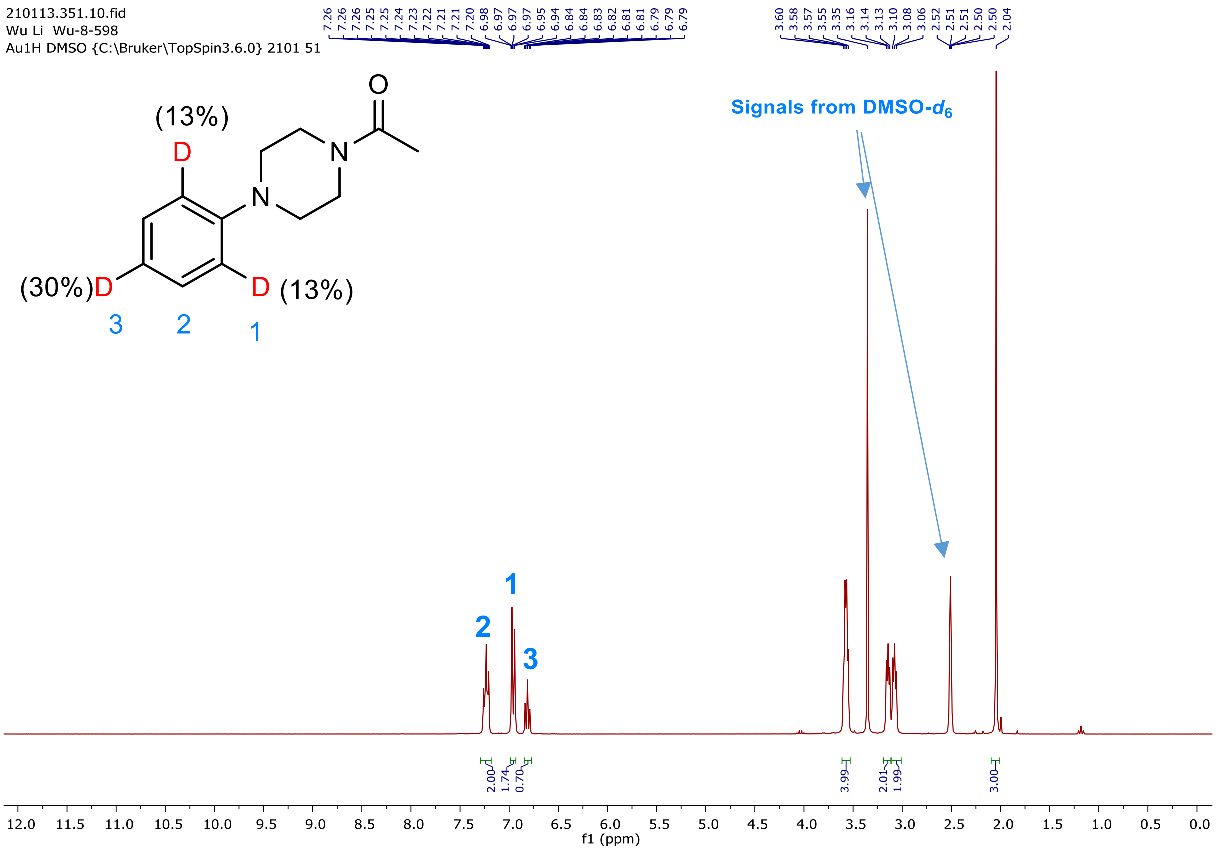

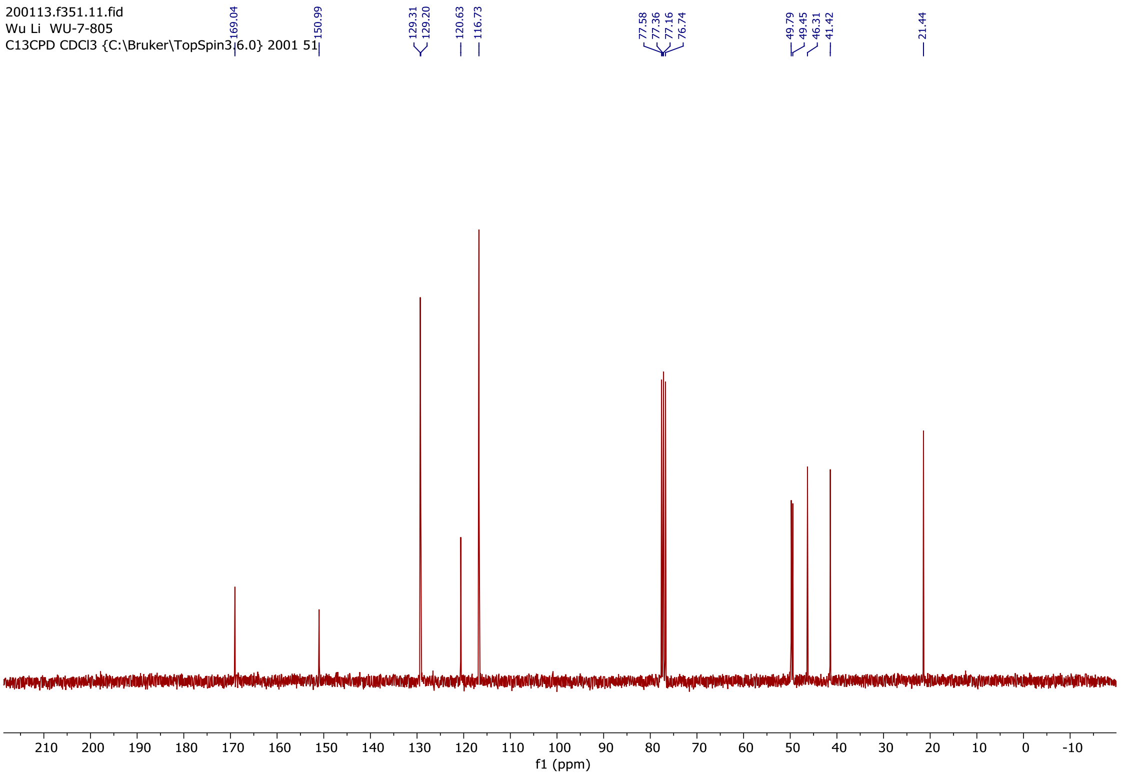

^{1}H NMR for 38a:

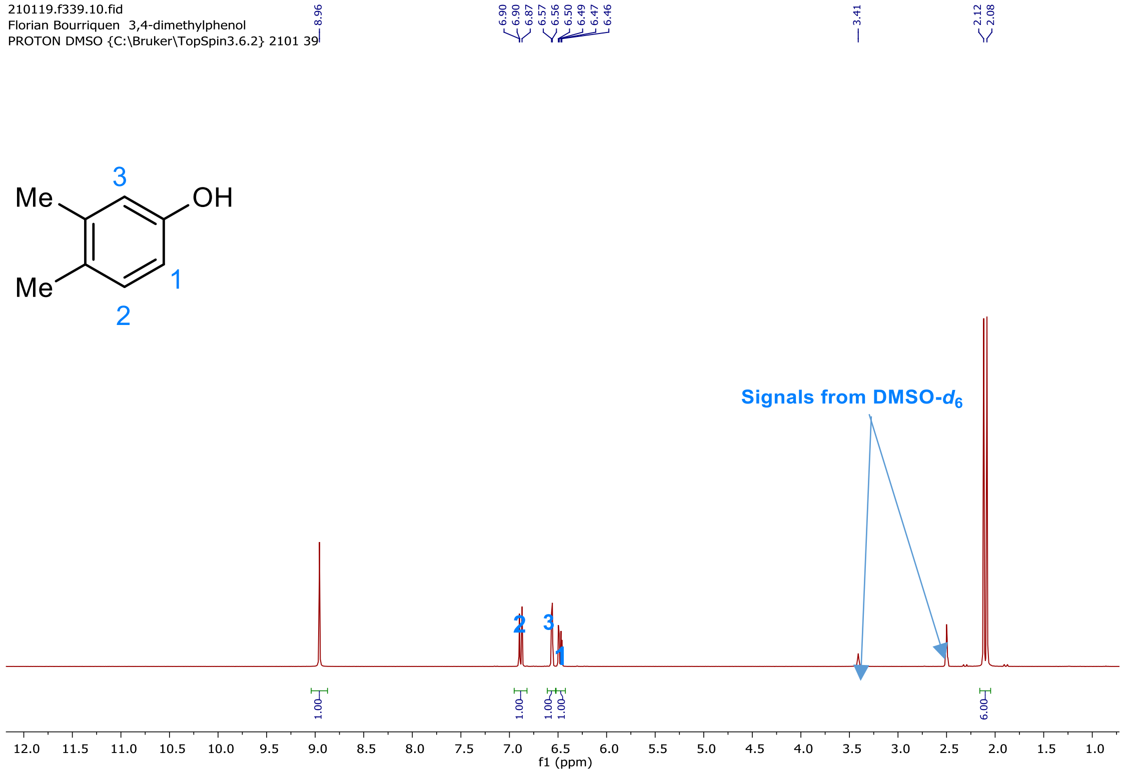

Original spectra for 38b:

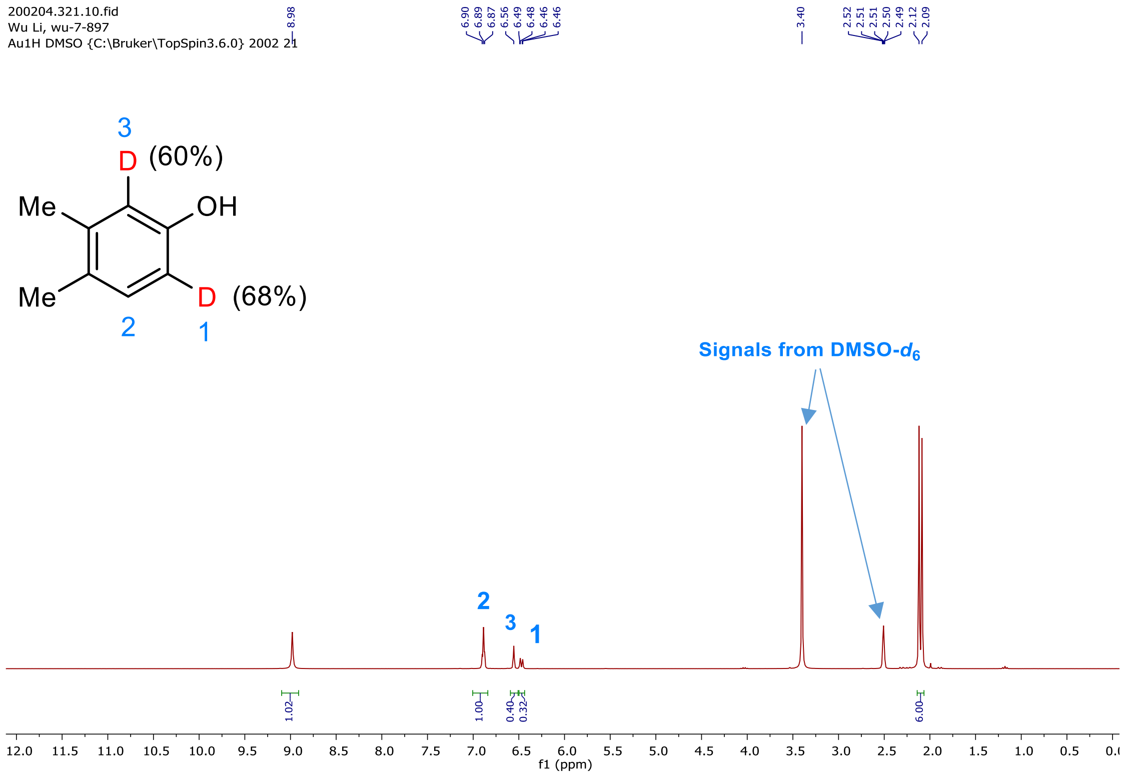

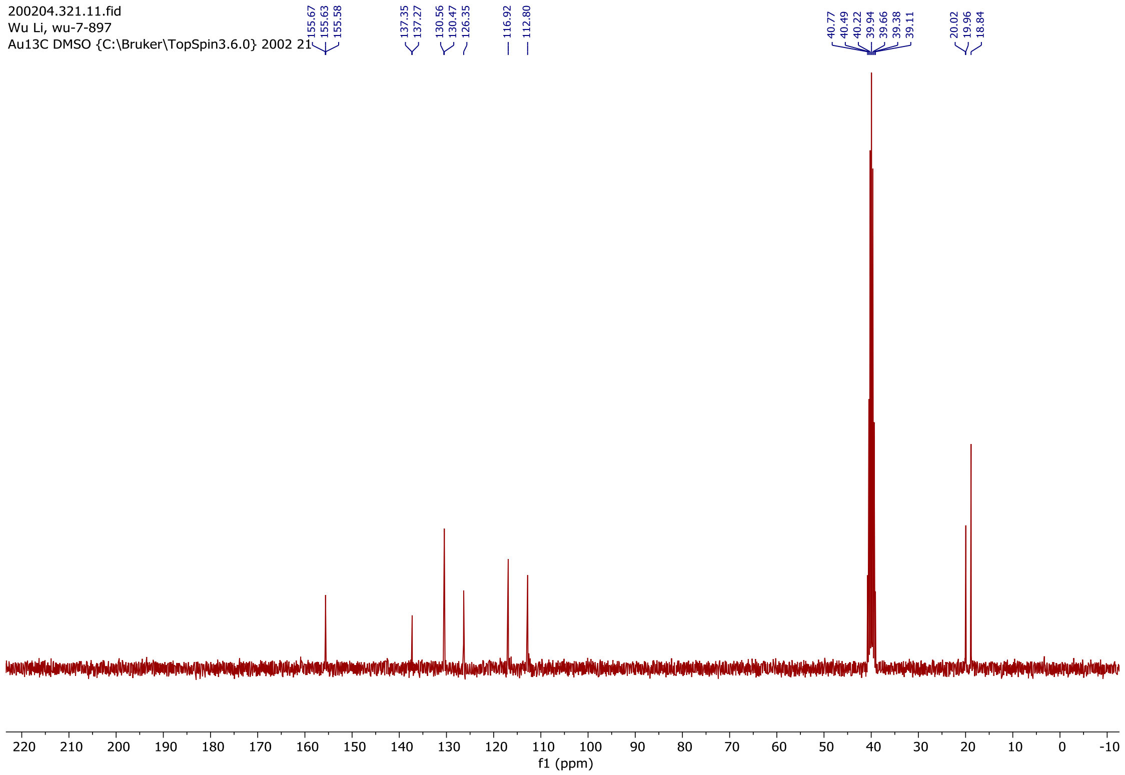

^{1}H NMR for 39a:

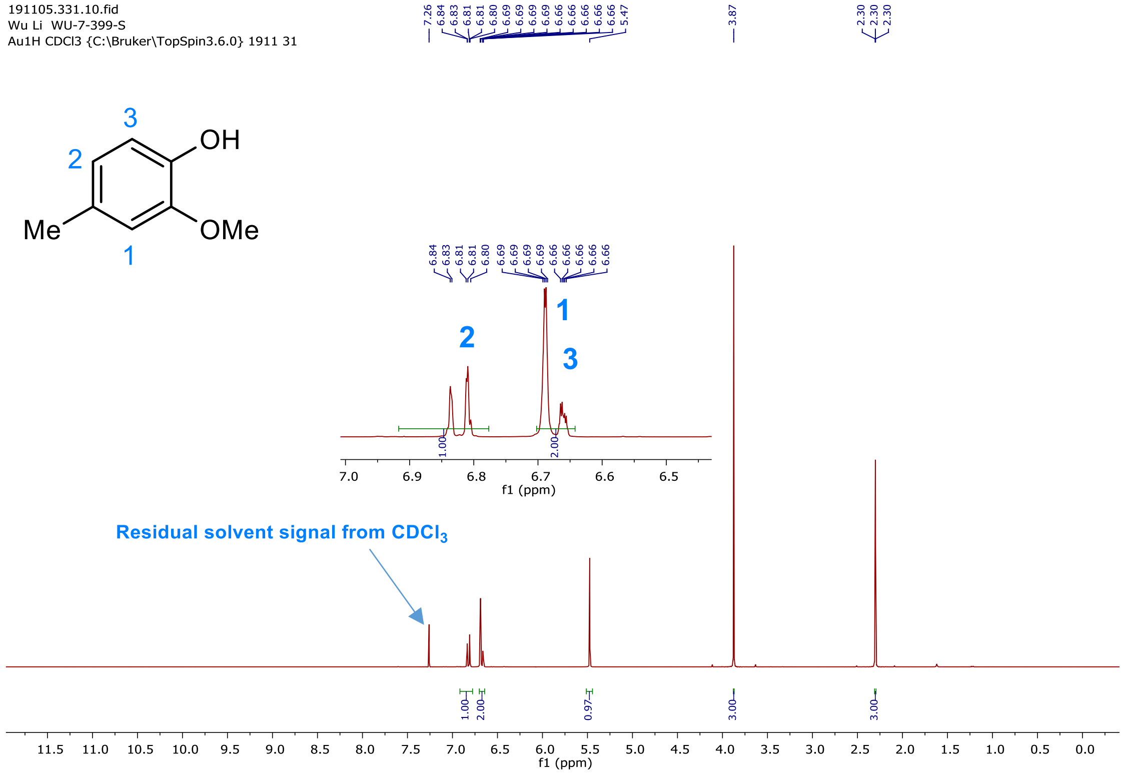

Original spectra for 39b:

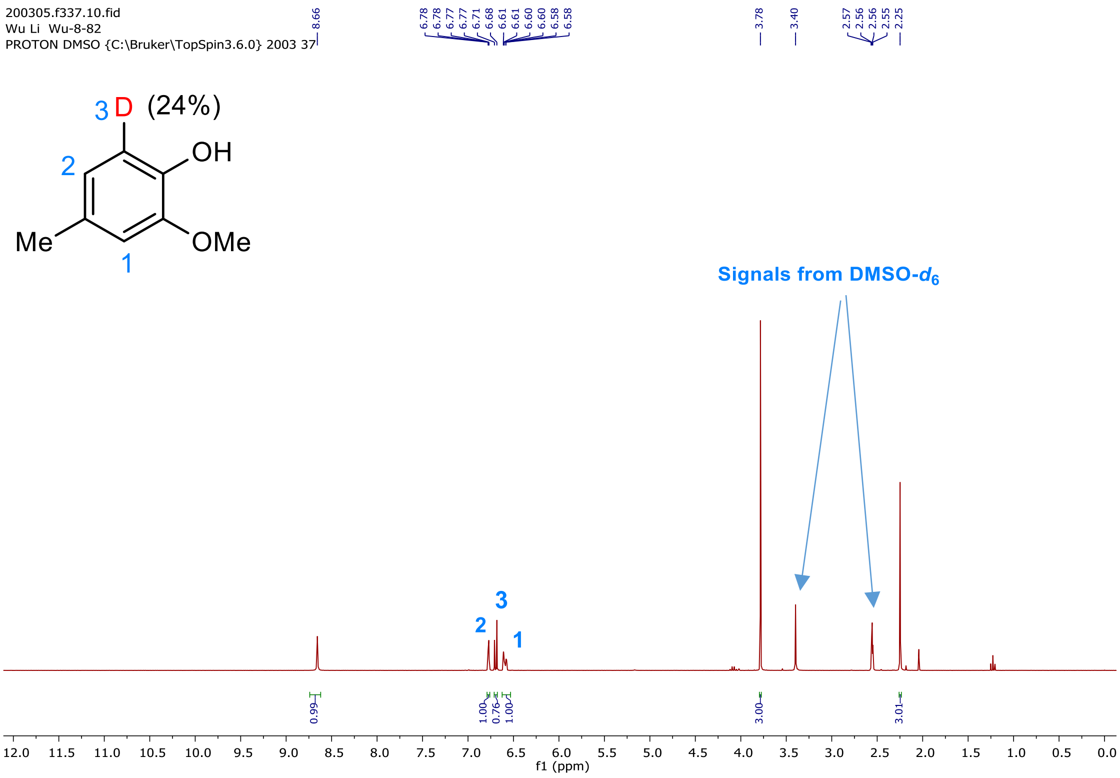

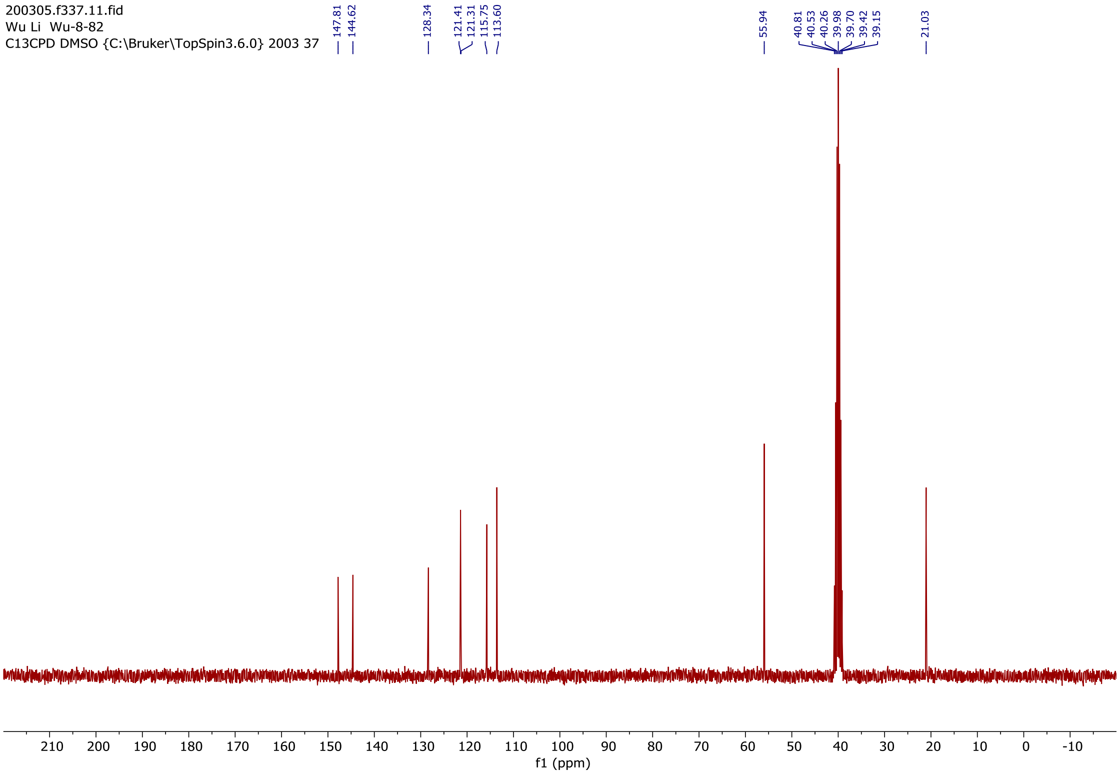

^{1}H NMR for 40a:

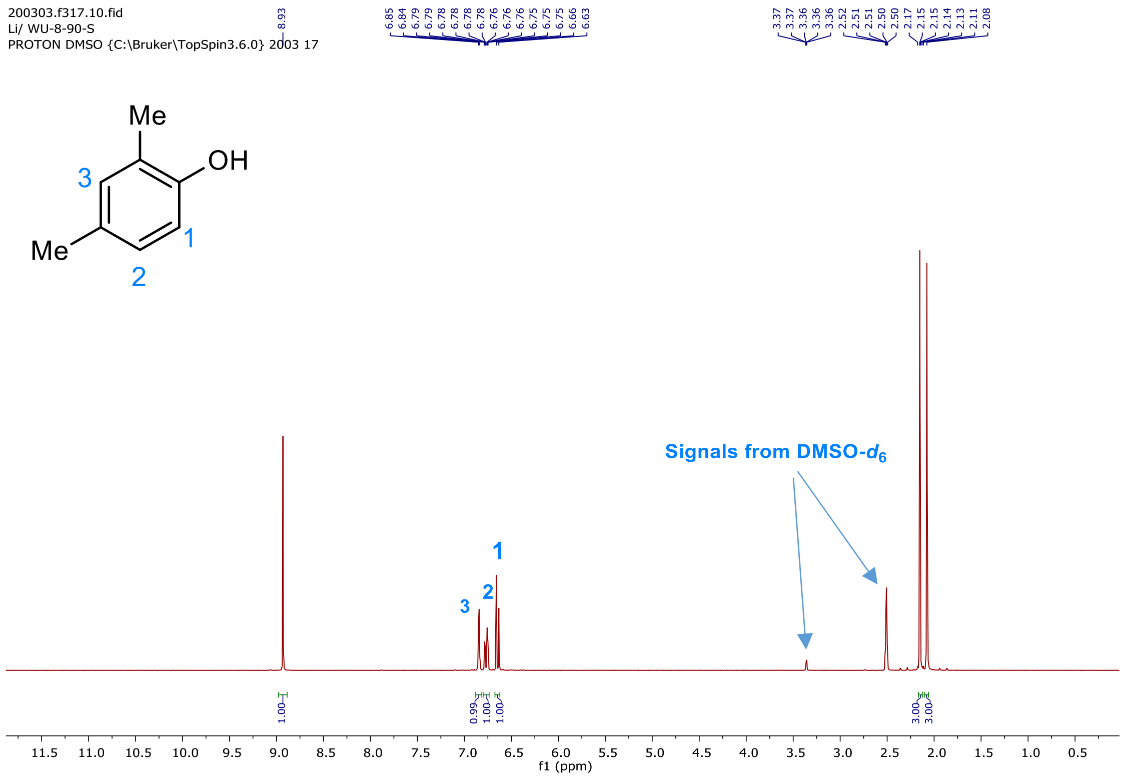

Original spectra for 40b:

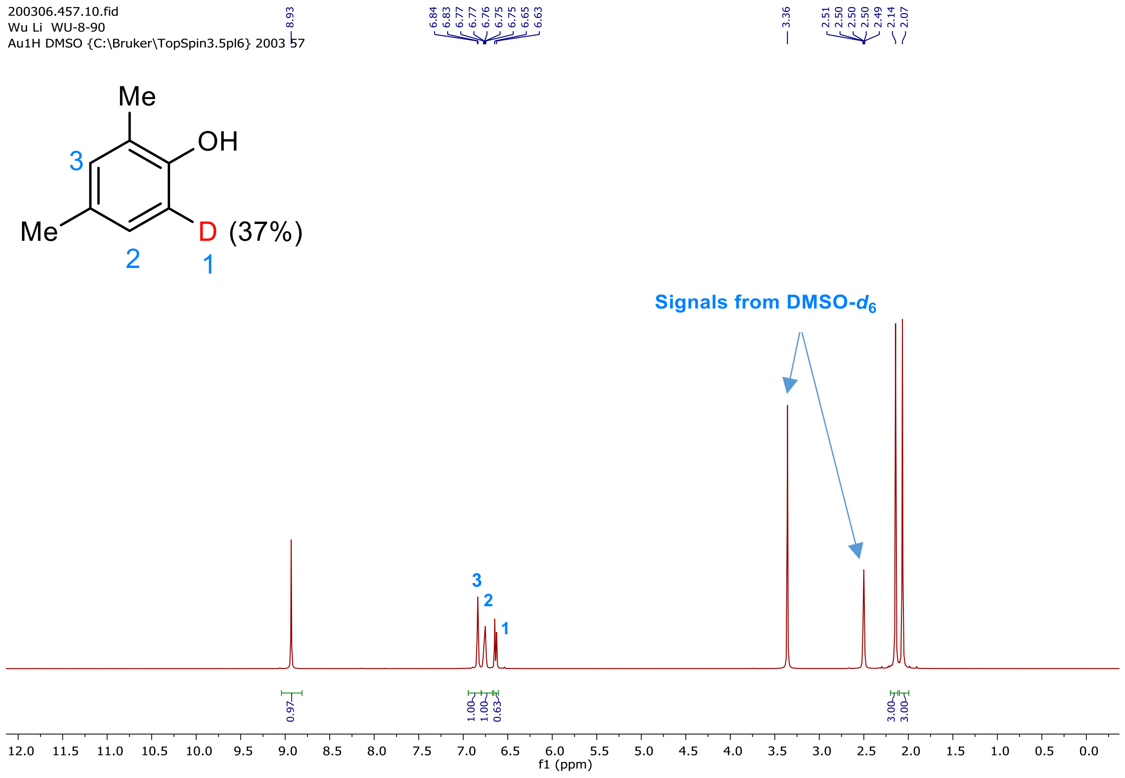

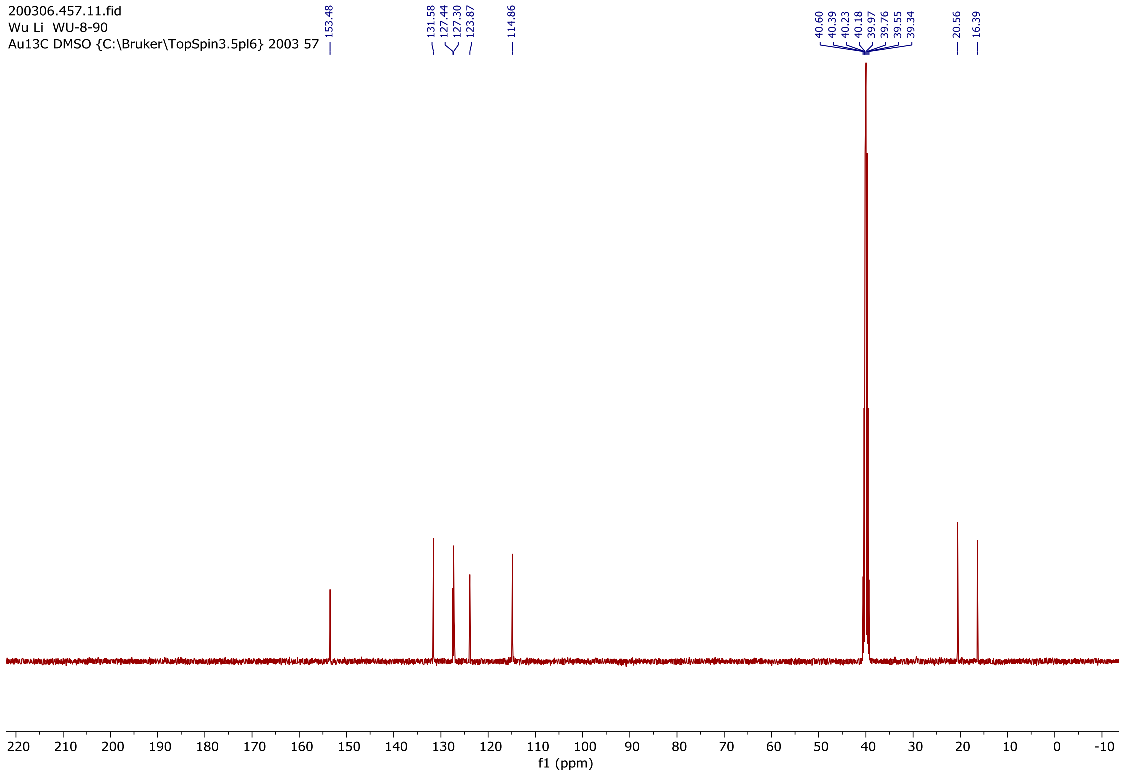

^{1}H NMR for 41a:

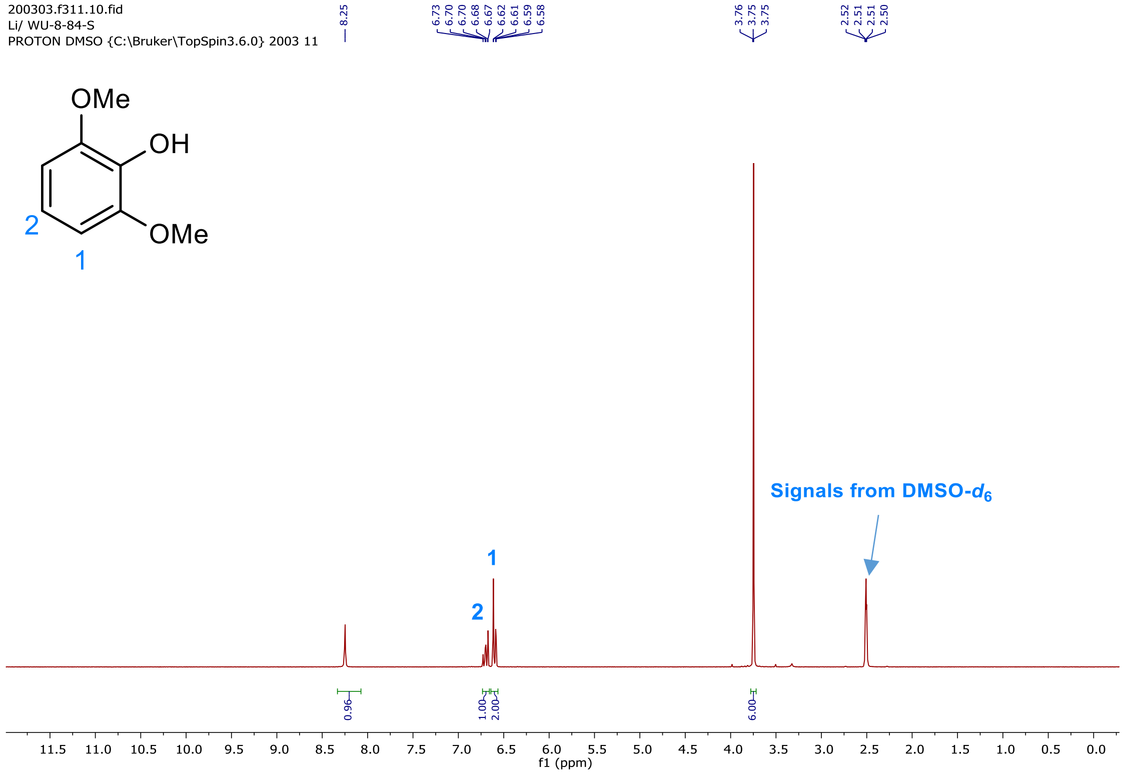

Original spectra for 41b:

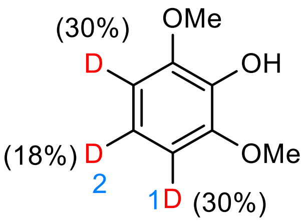

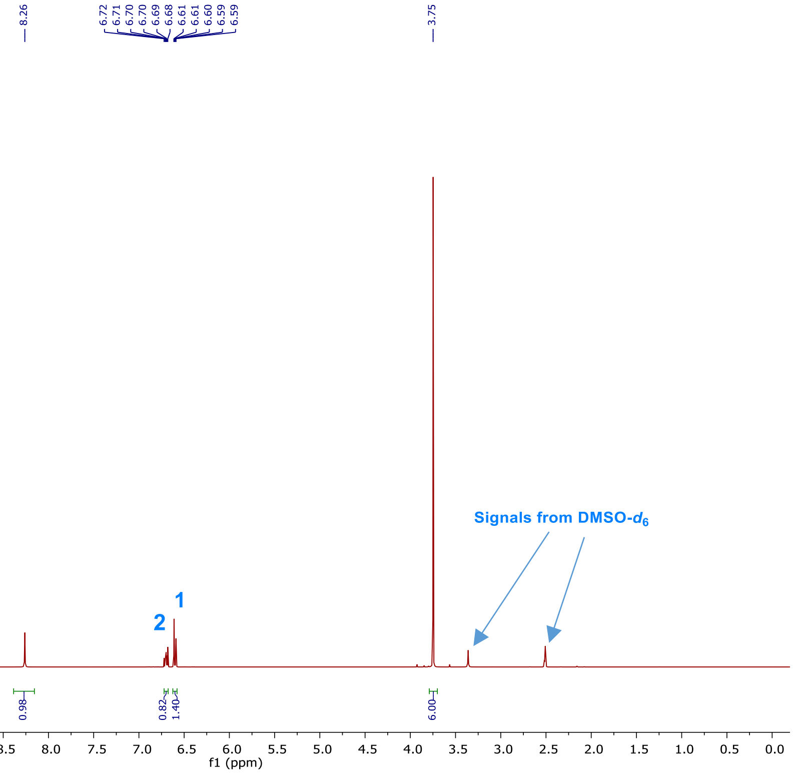

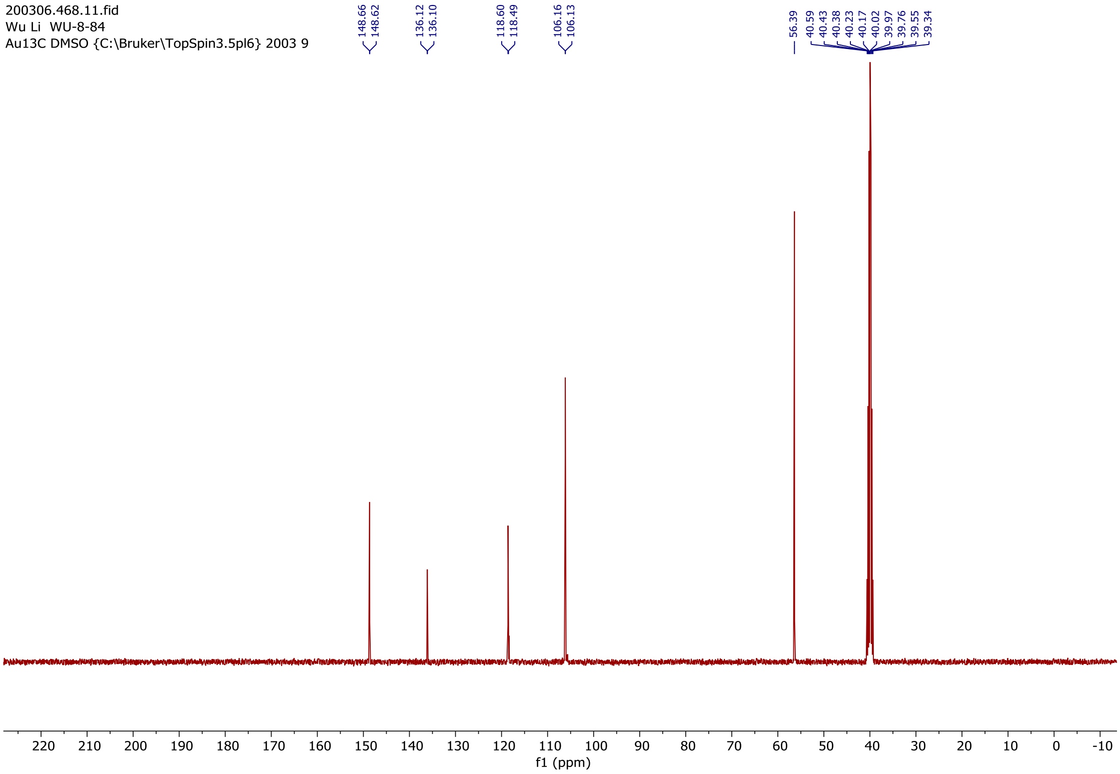

^{1}H NMR for 42a:

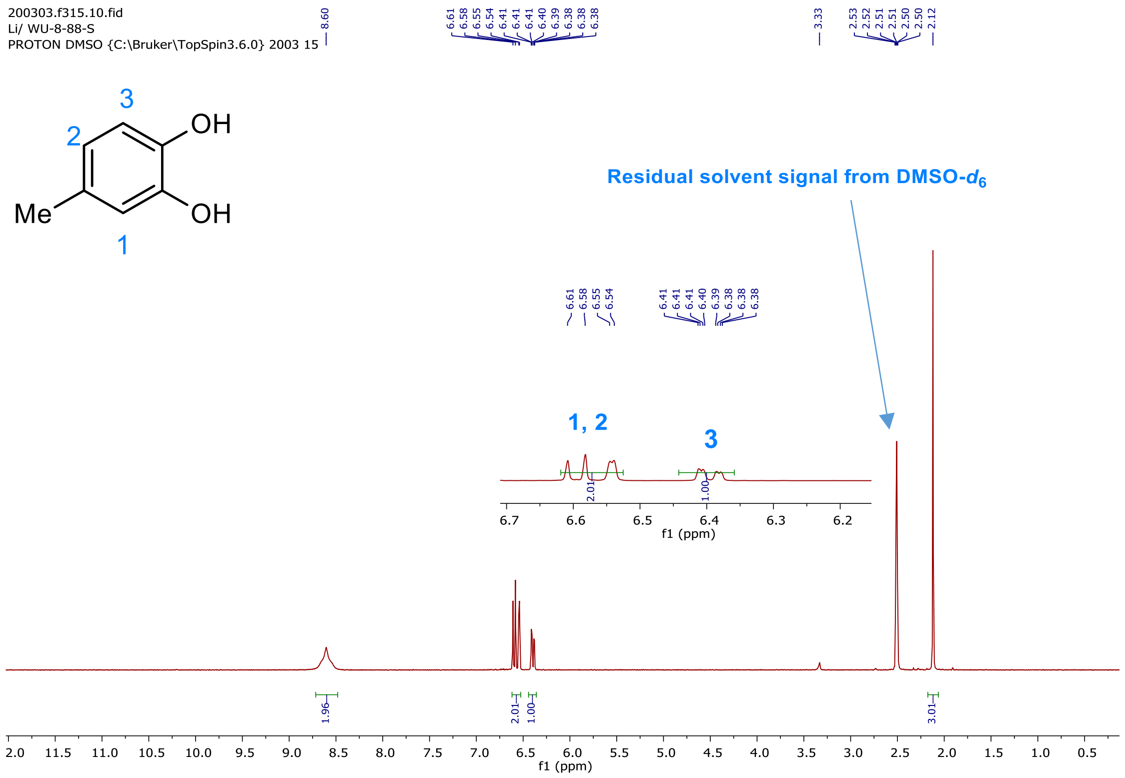

Original spectra for 42b:

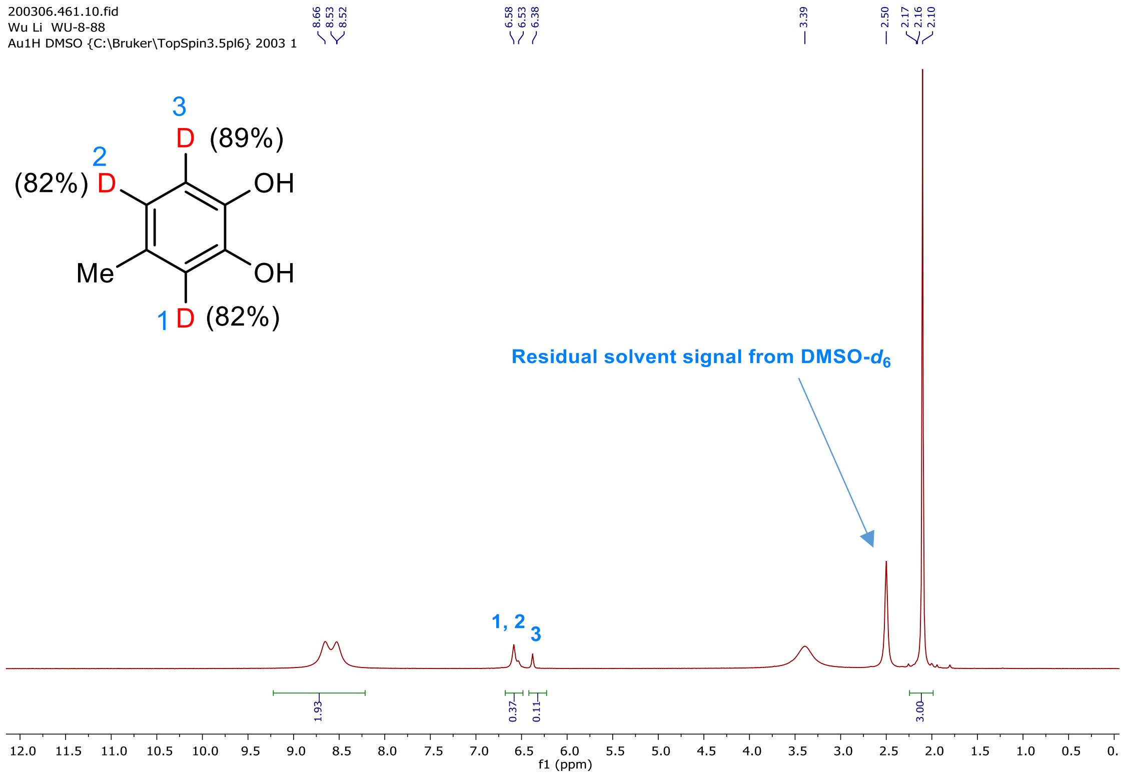

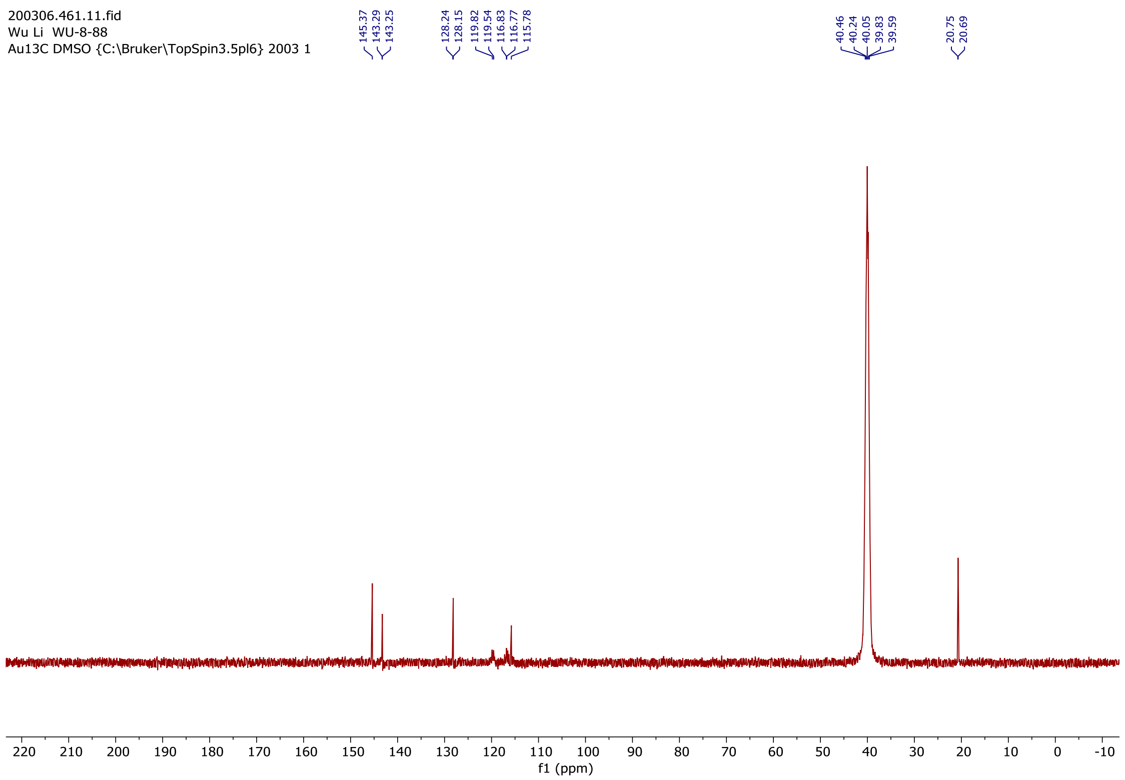

^{1}H NMR for 43a:

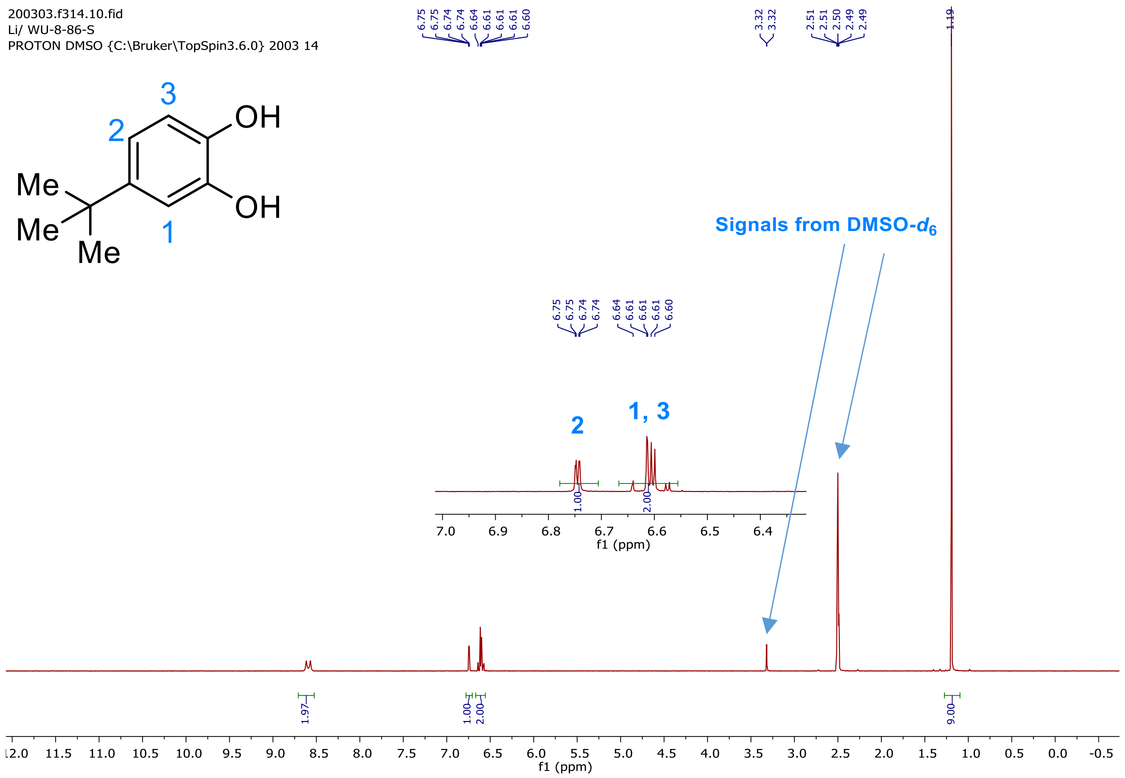

Original spectra for 43b:

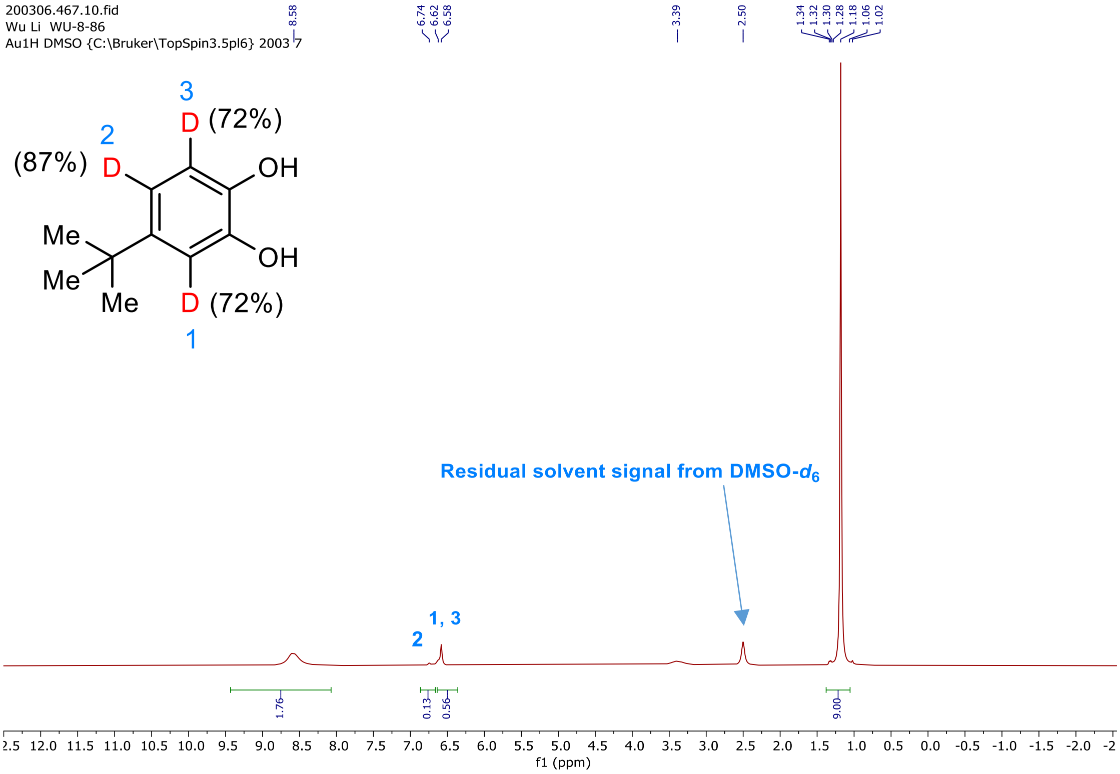

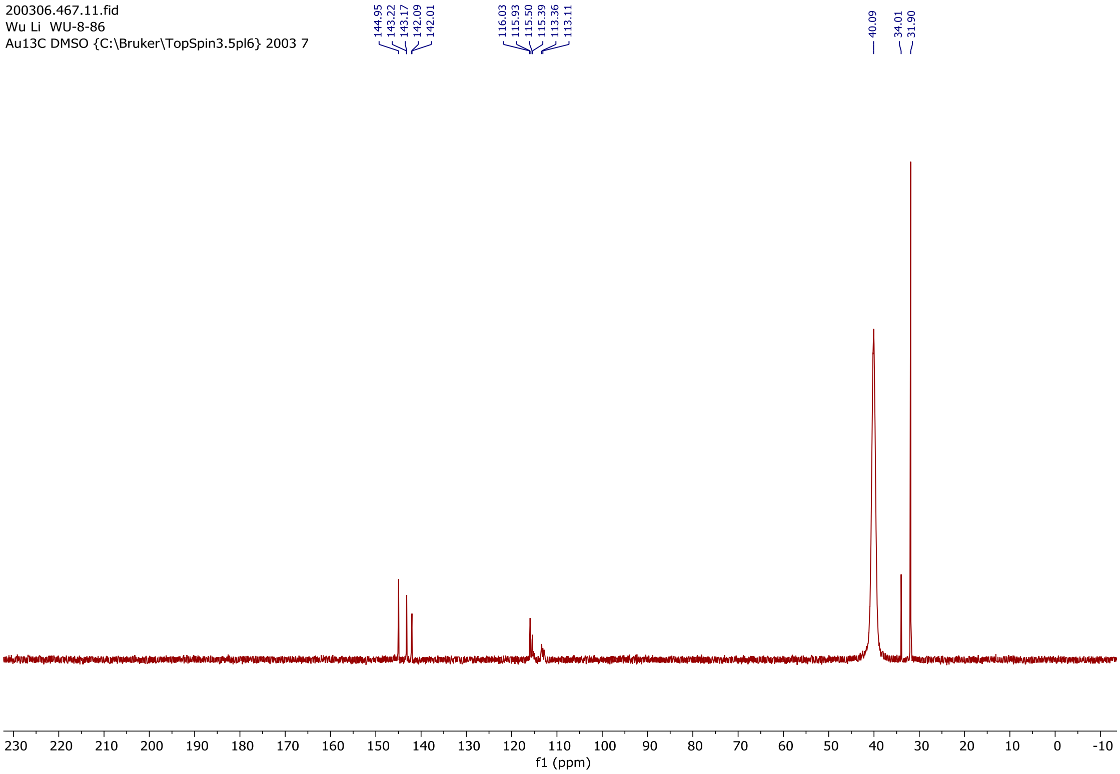

^{1}H NMR for 44a:

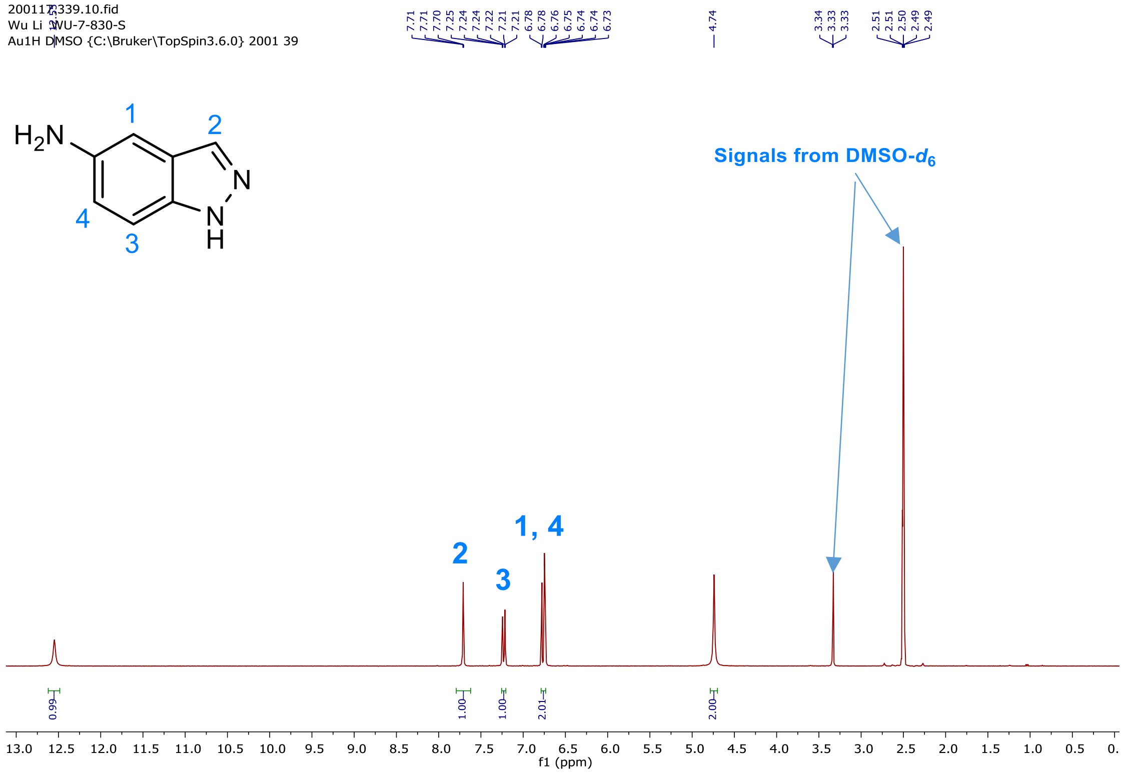

Original spectra for 44b:

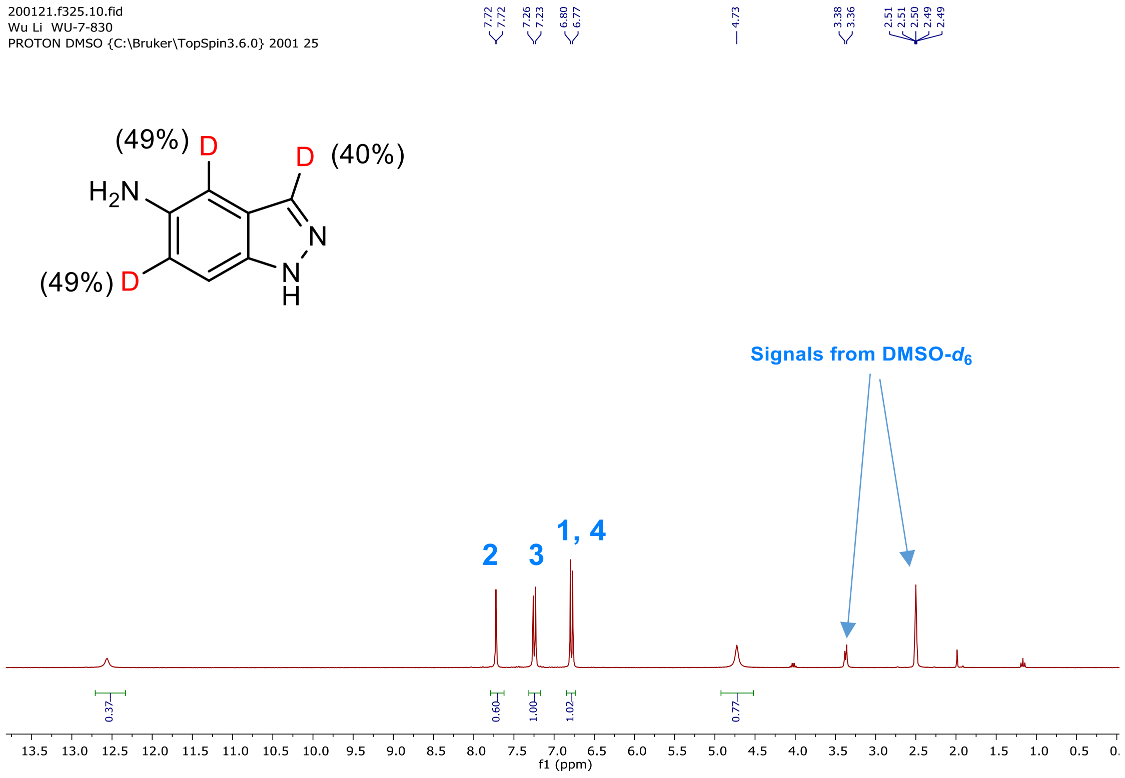

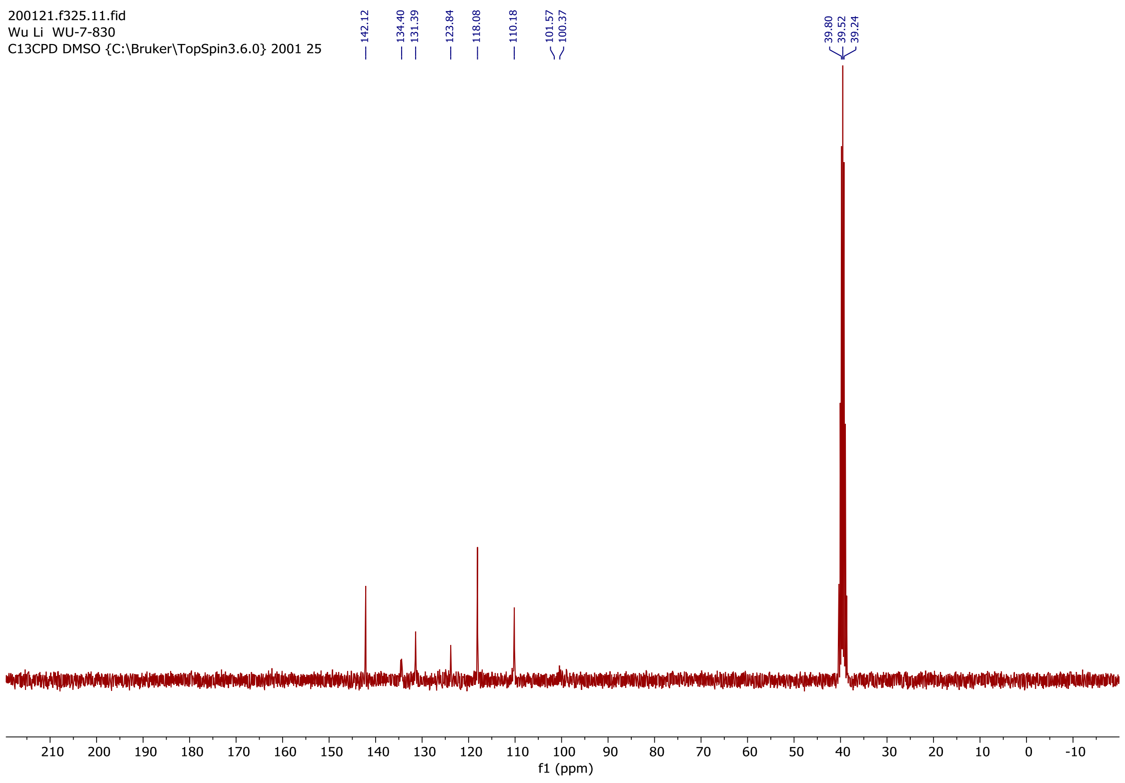

^{1}H NMR for 45a:

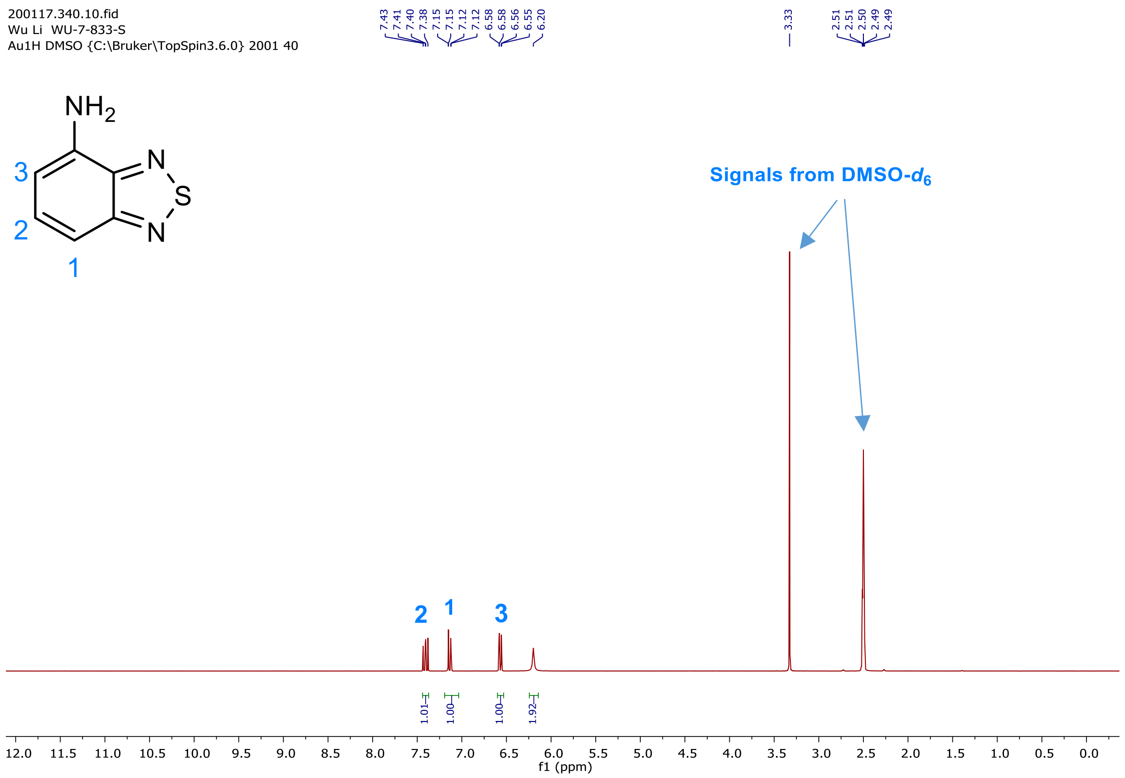

Original spectra for 45b:

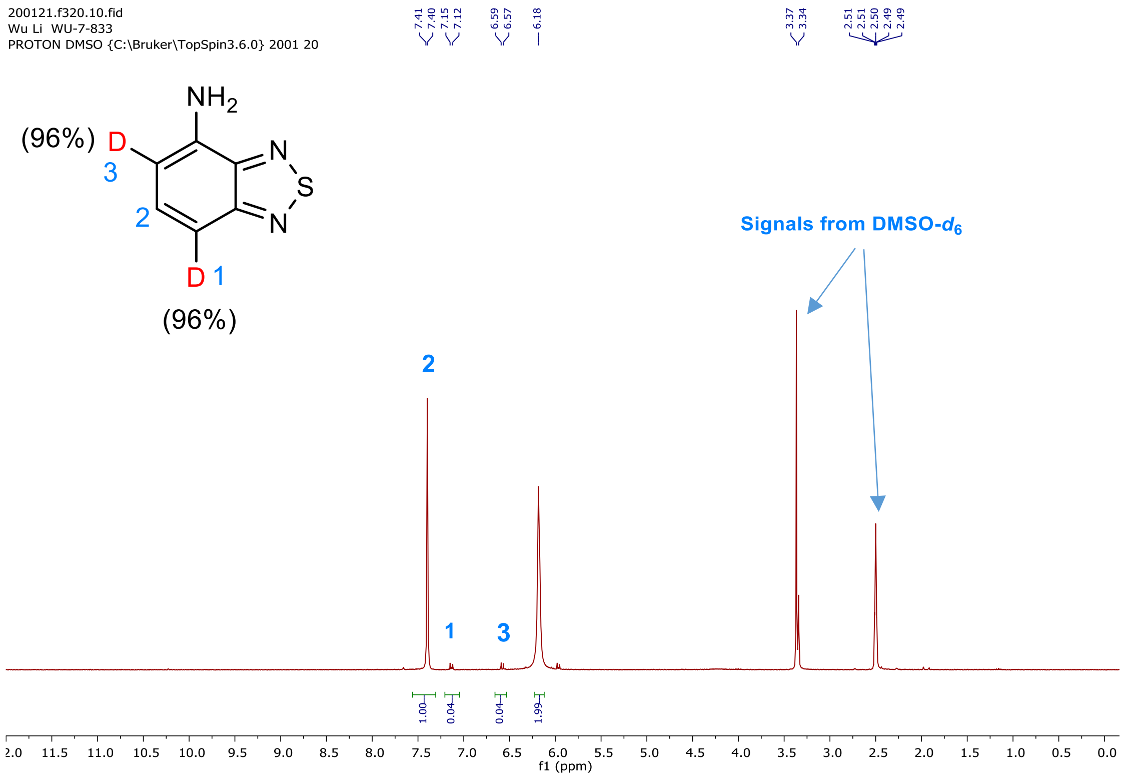

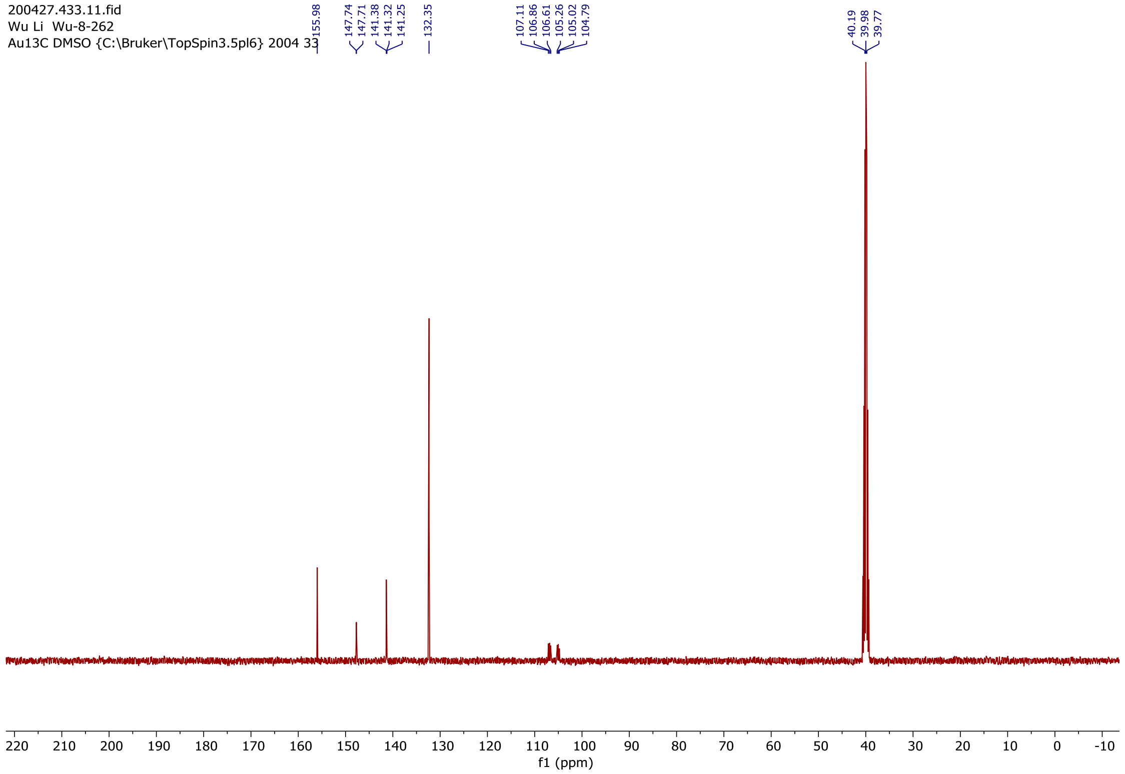

^{1}H NMR for 46a:

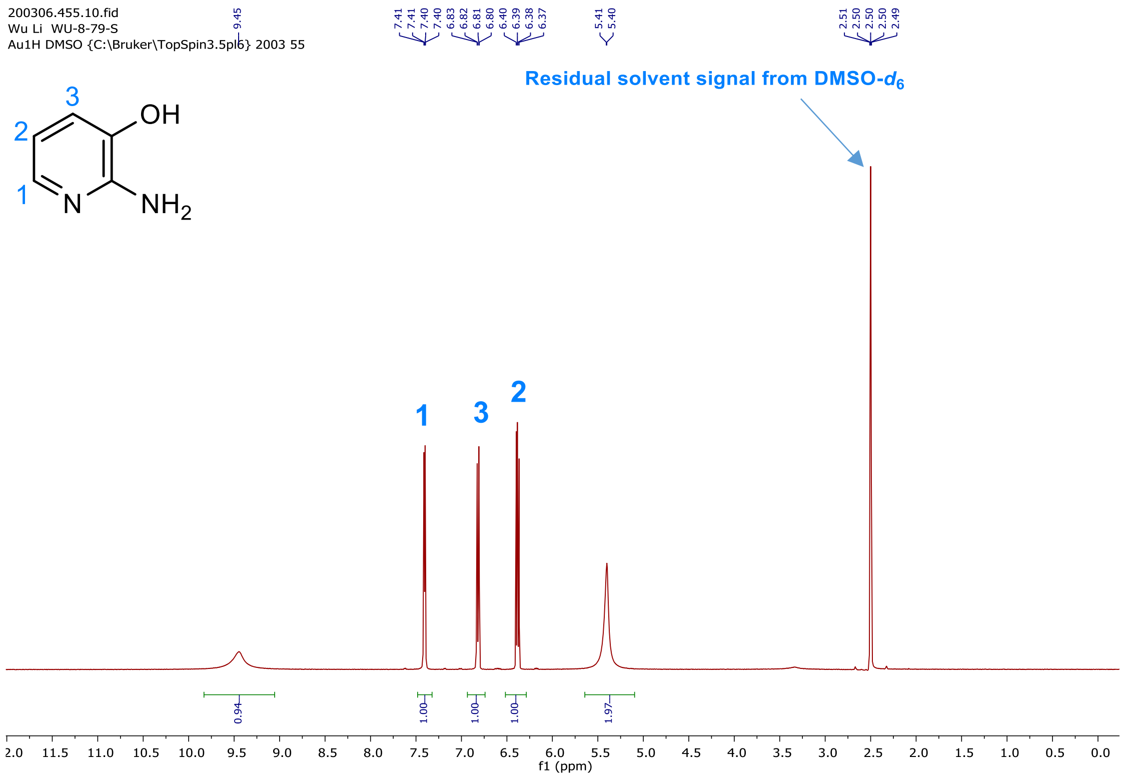

Original spectra for 46b:

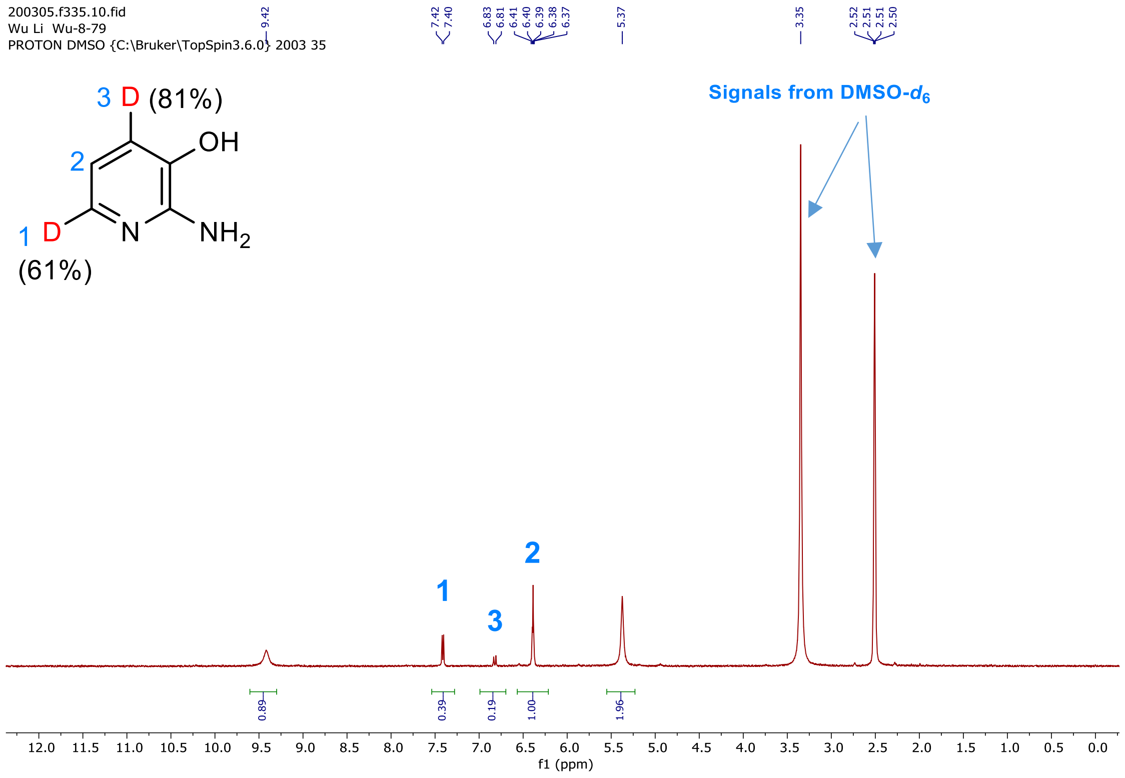

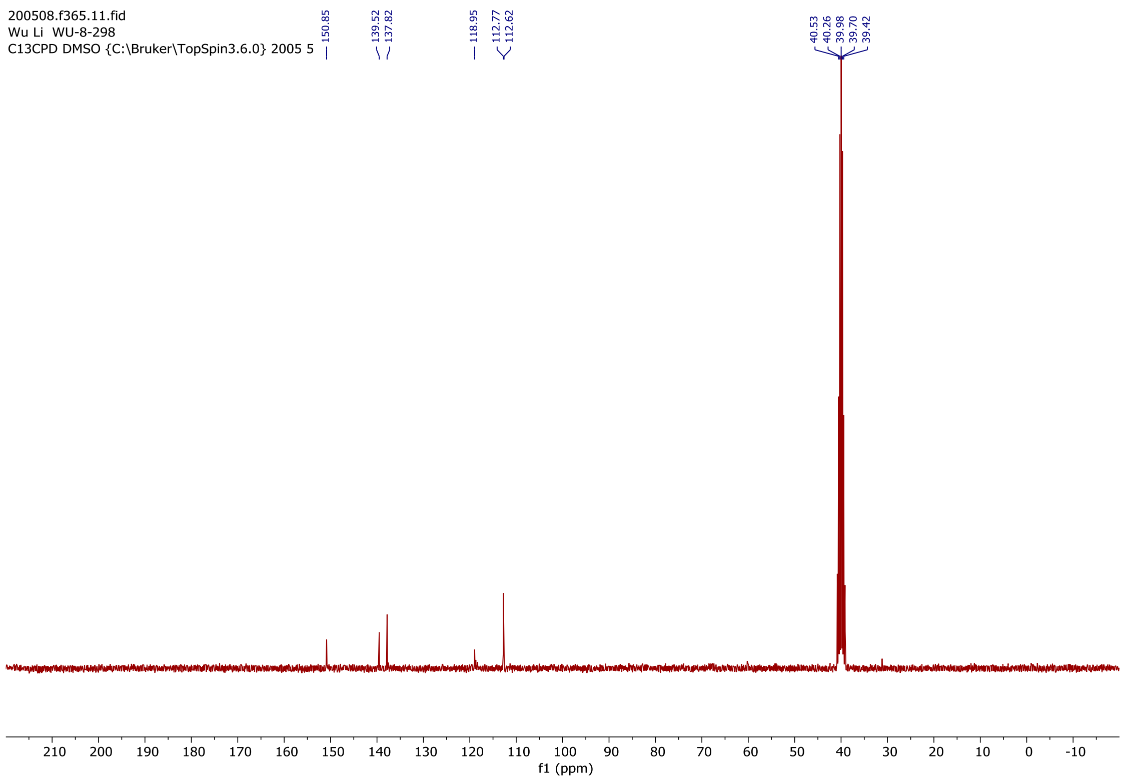

^{1}H NMR for 47a:

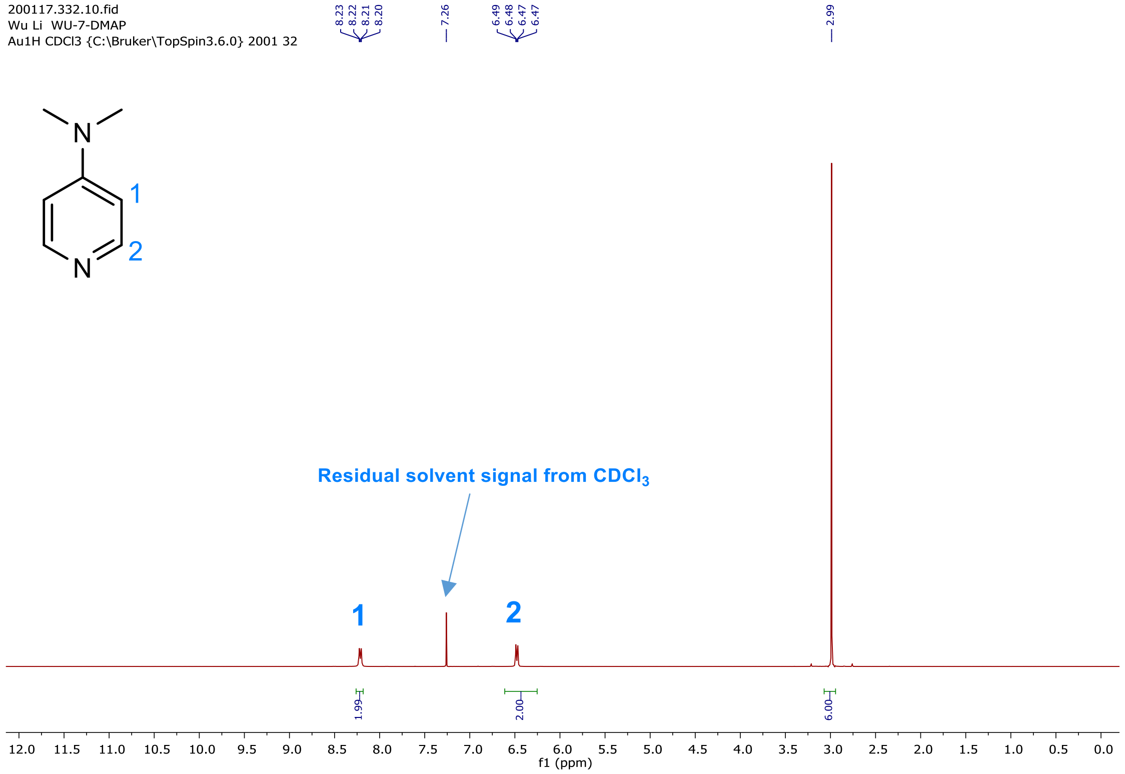

Original spectra for 47b:

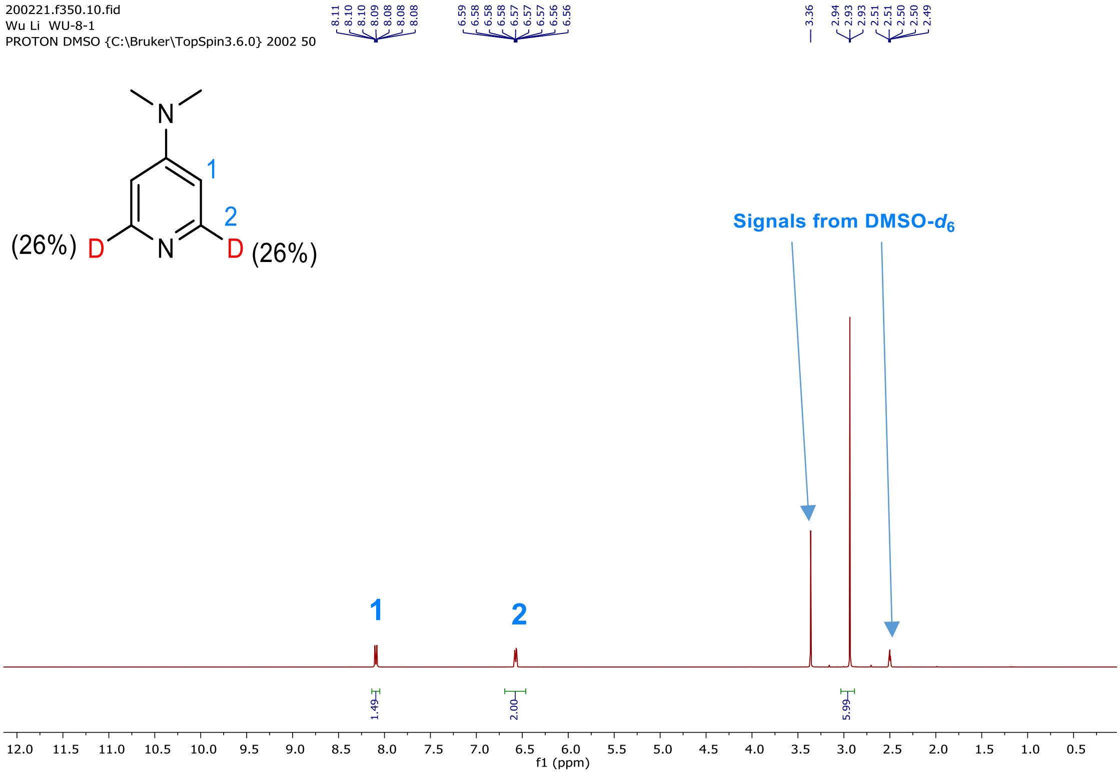

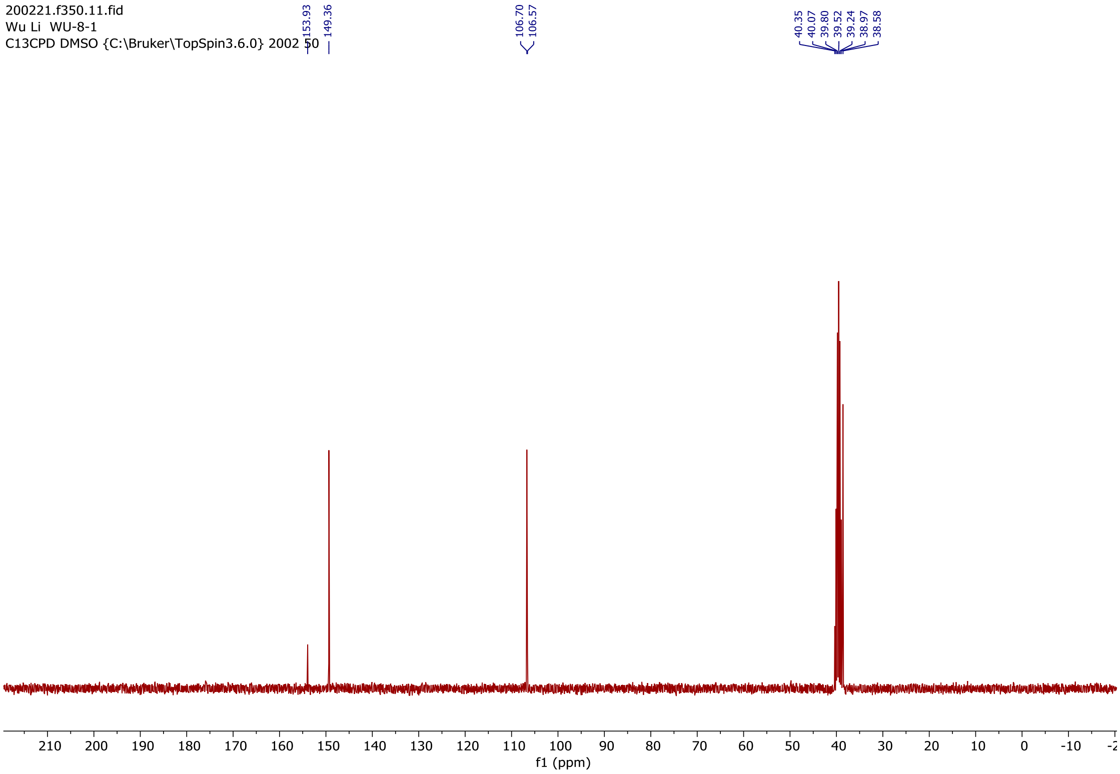

^{1}H NMR for 48a:

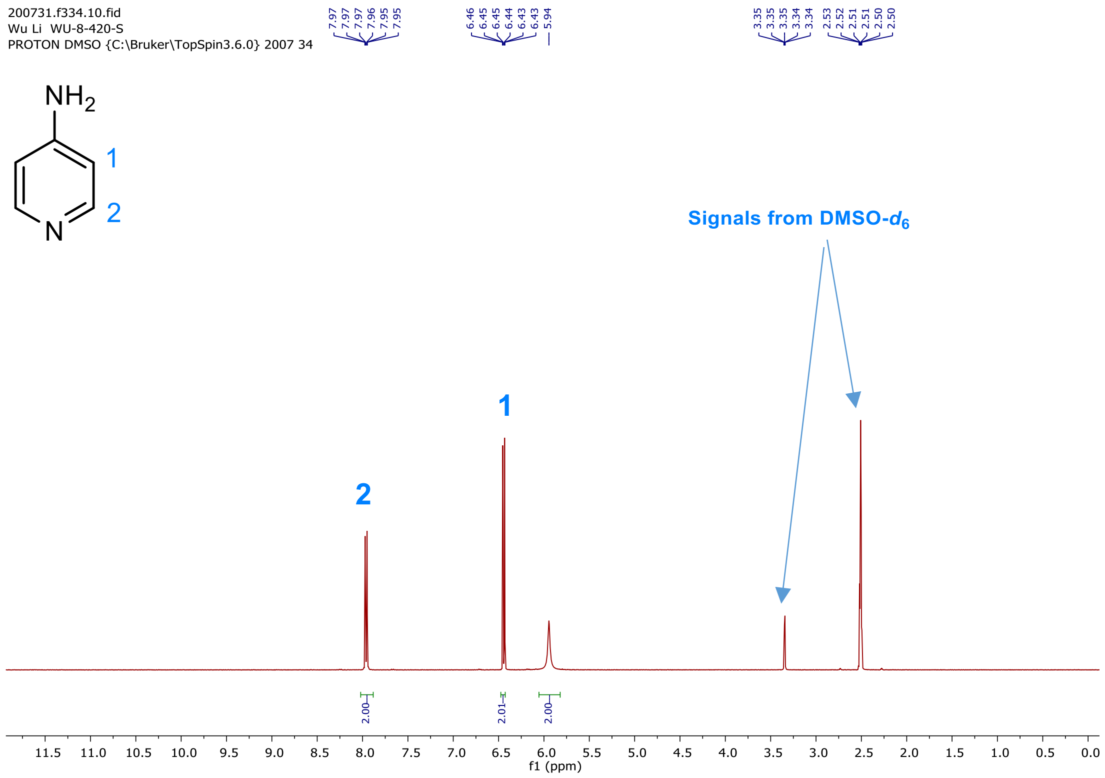

Original spectra for 48b:

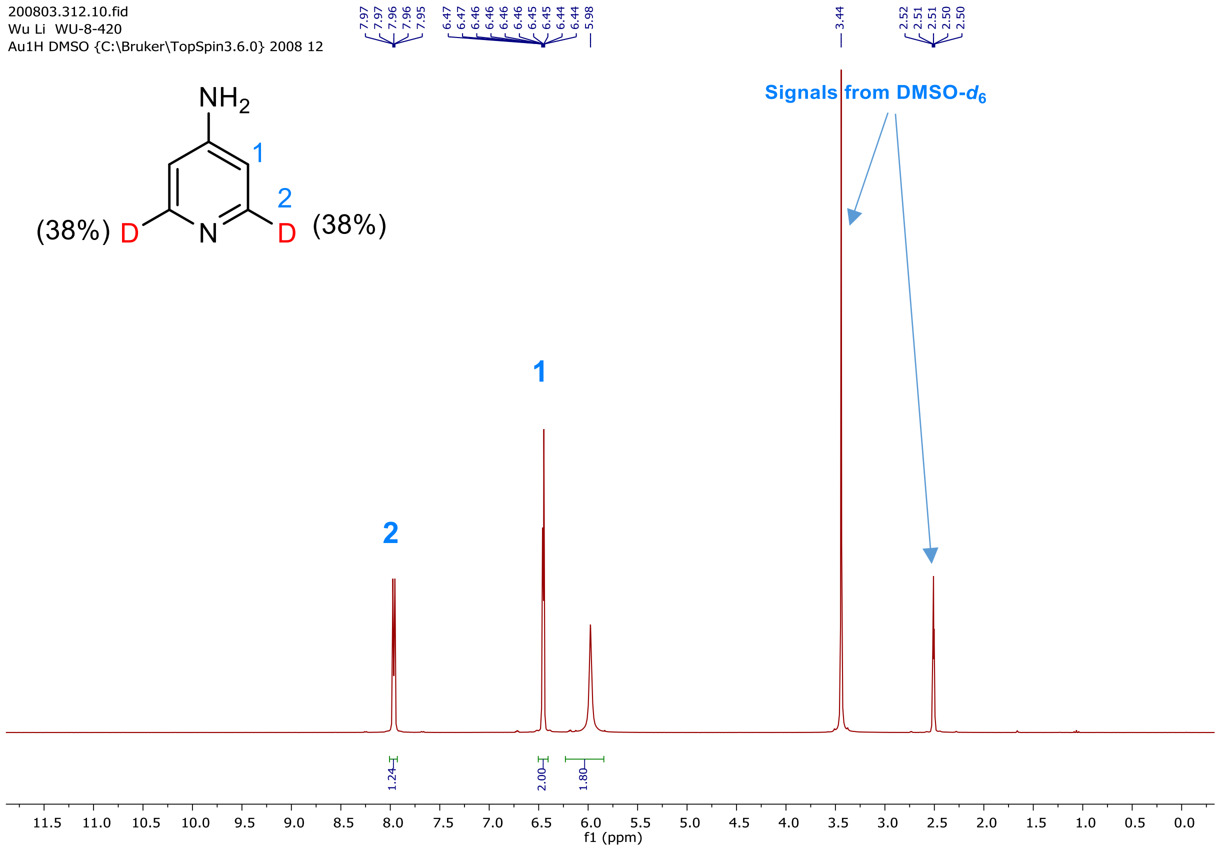

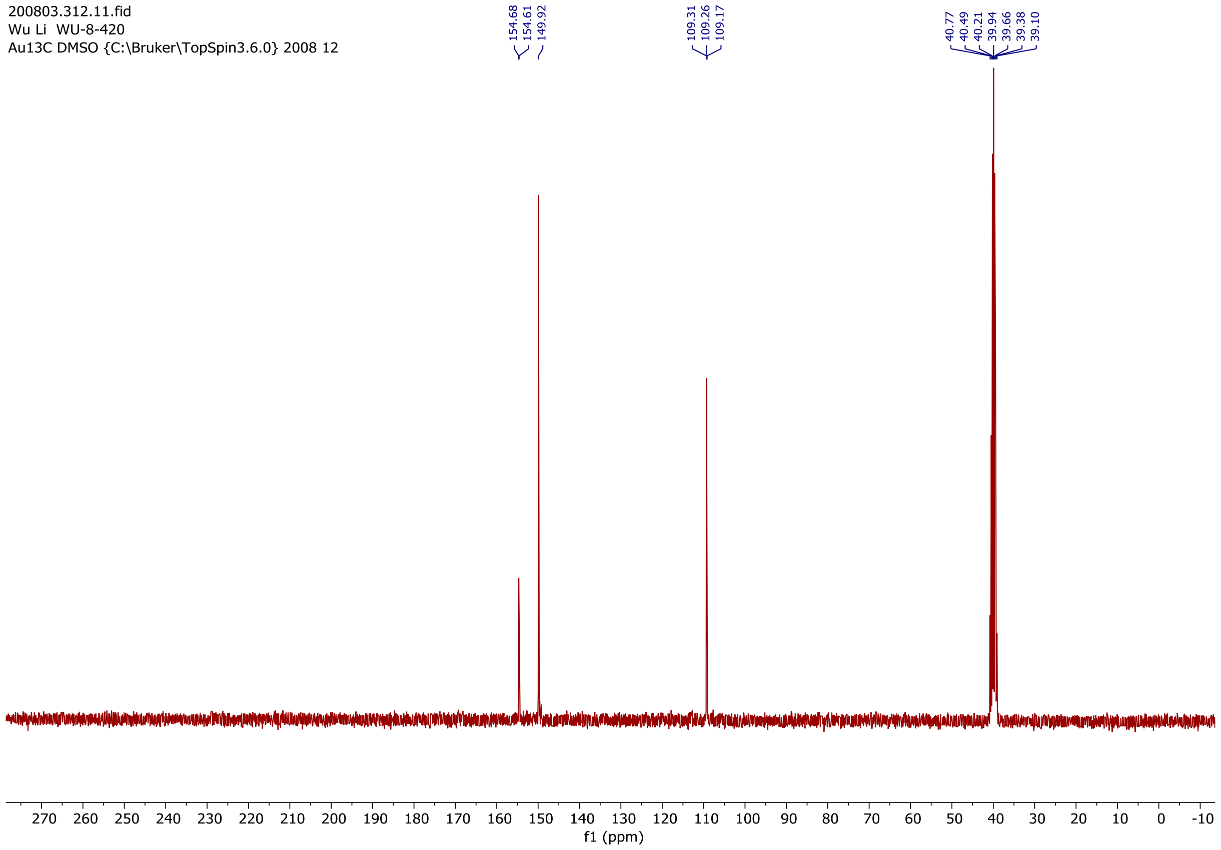

^{1}H NMR for 49a:

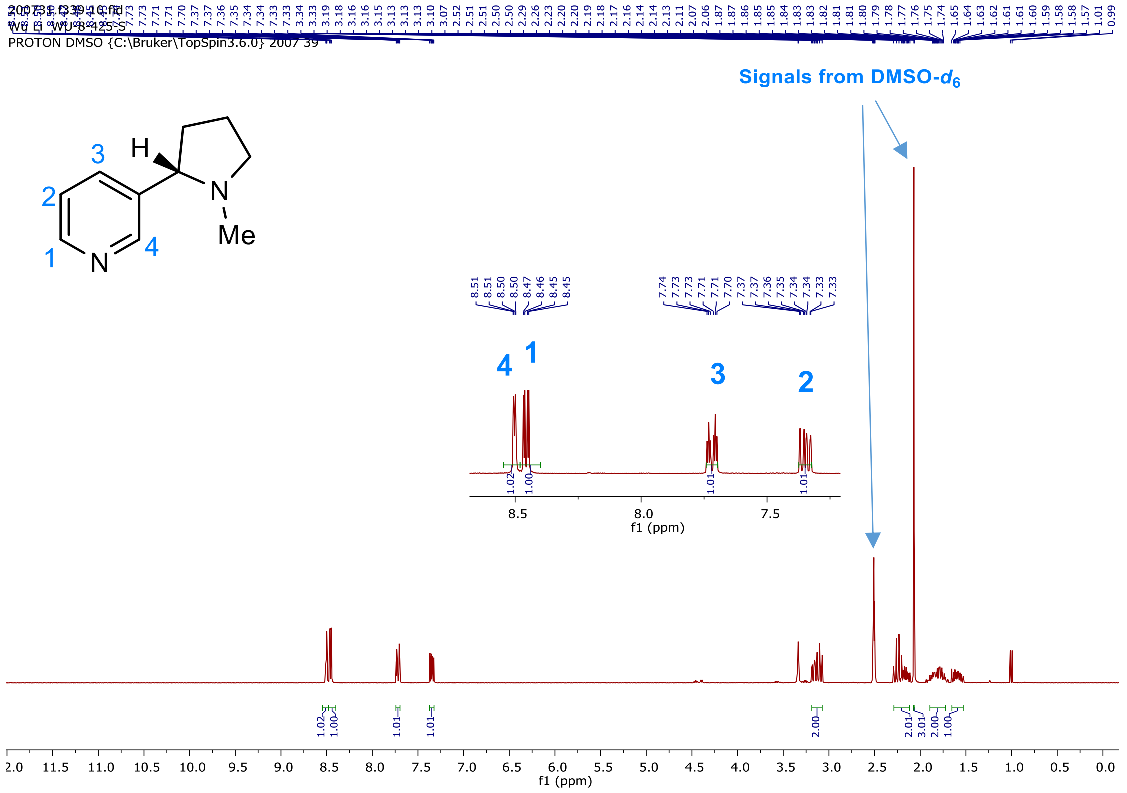

Original spectra for 49b:

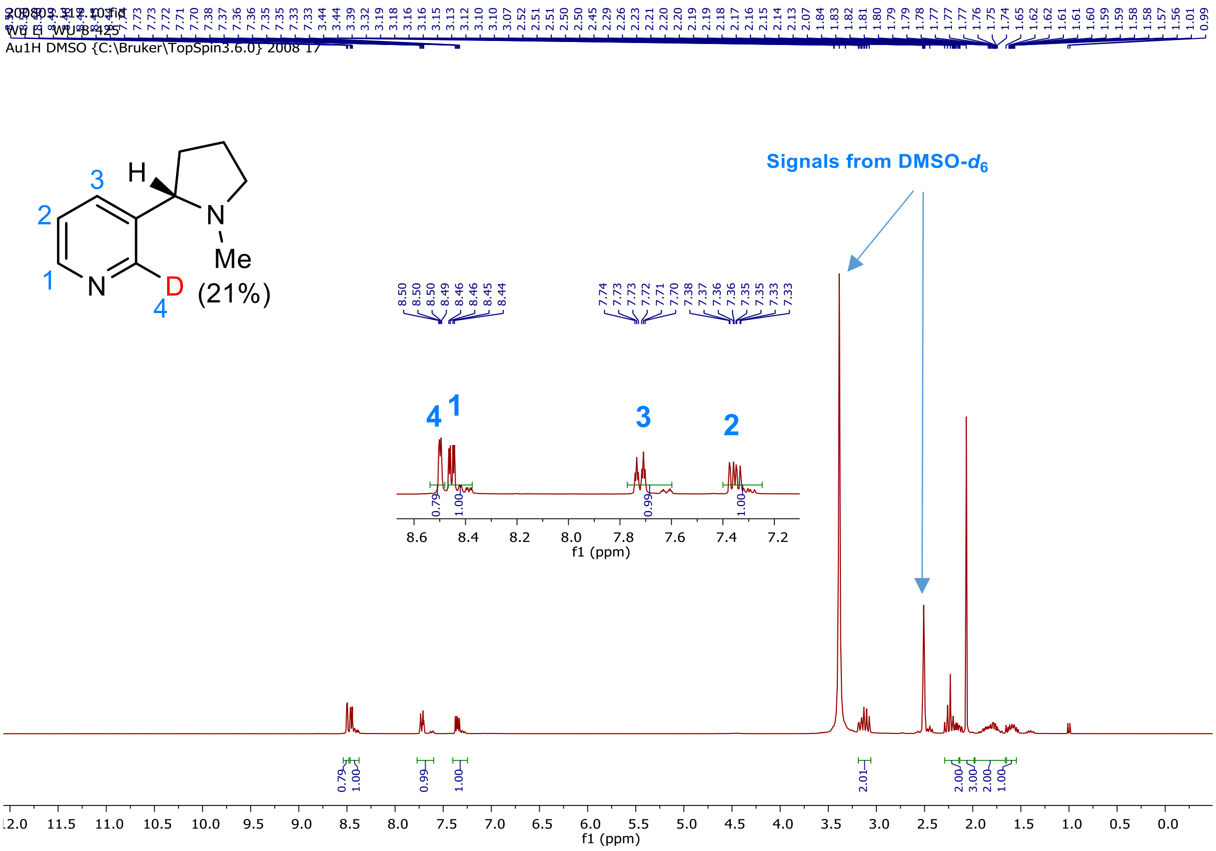

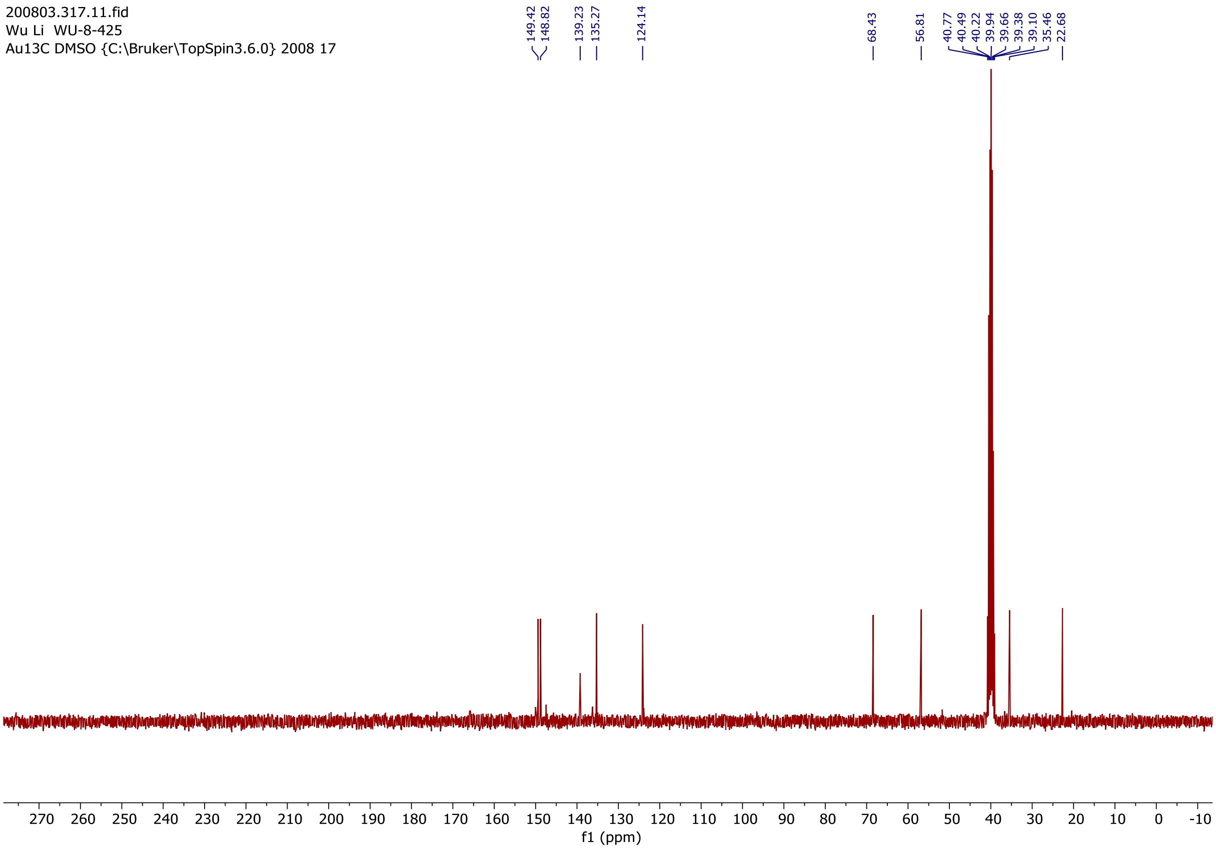

^{1}H NMR for 50a:

In DMSO-d_6:

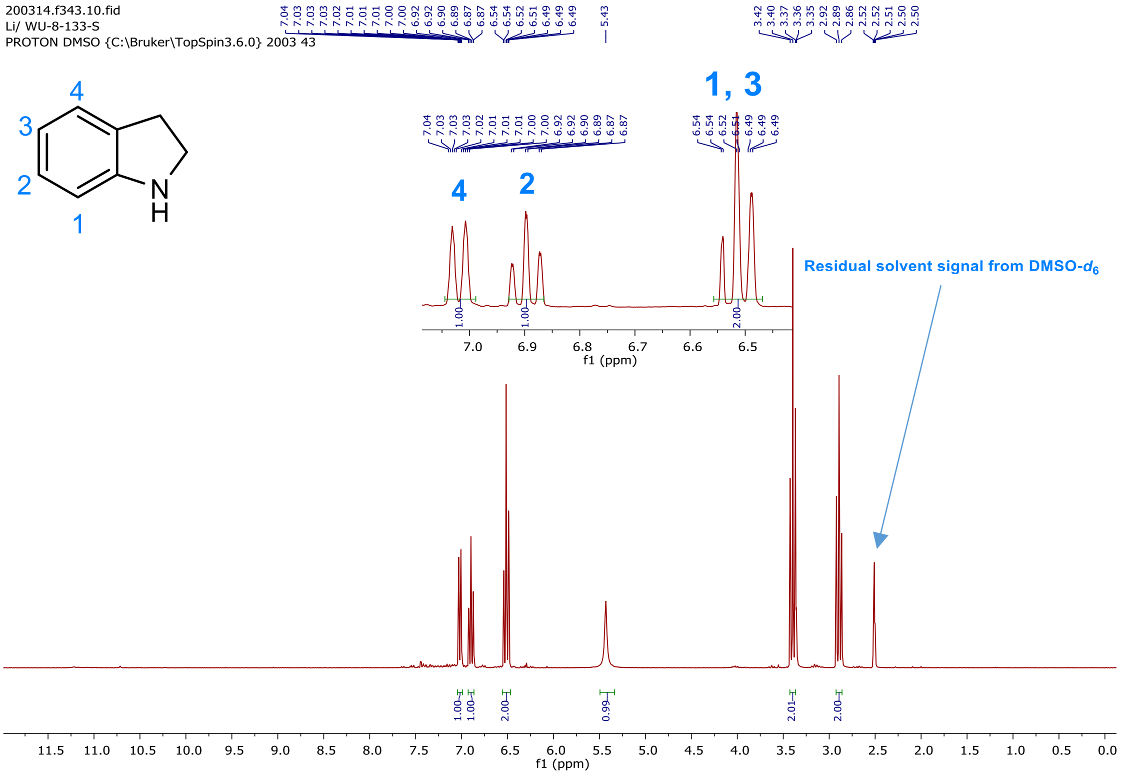

In CDCl$_3$:

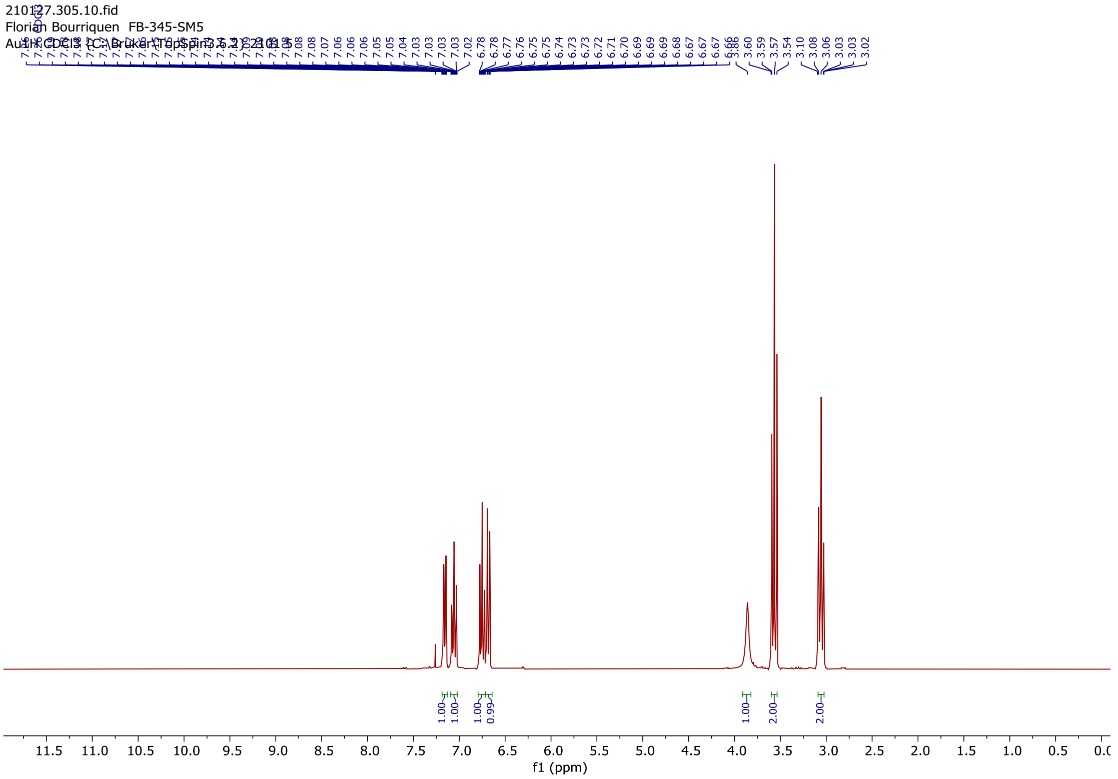

Original spectra for 50b:

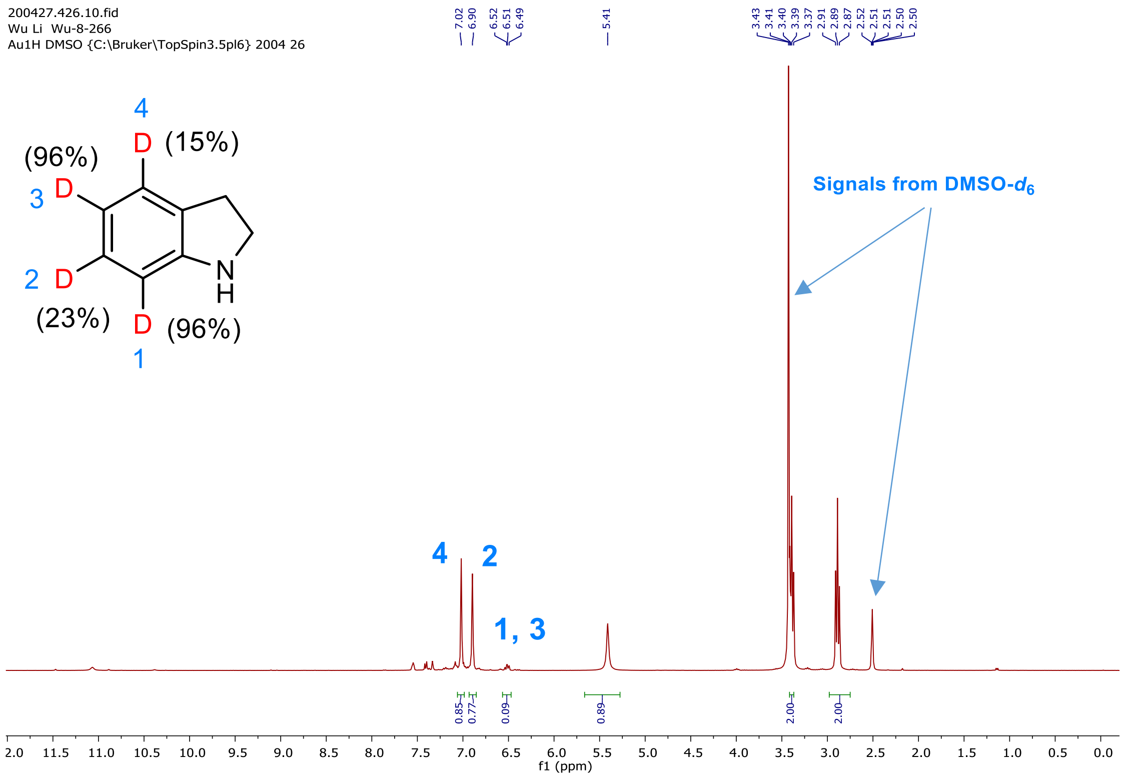

In $CDCl_3$:

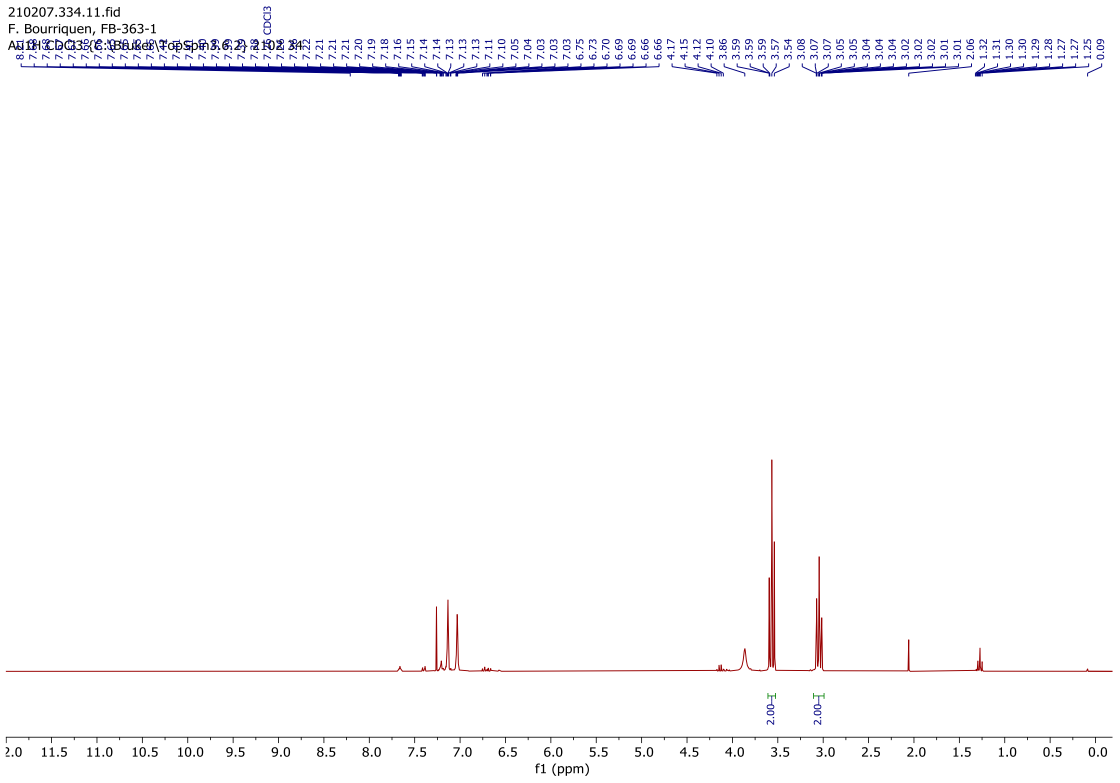

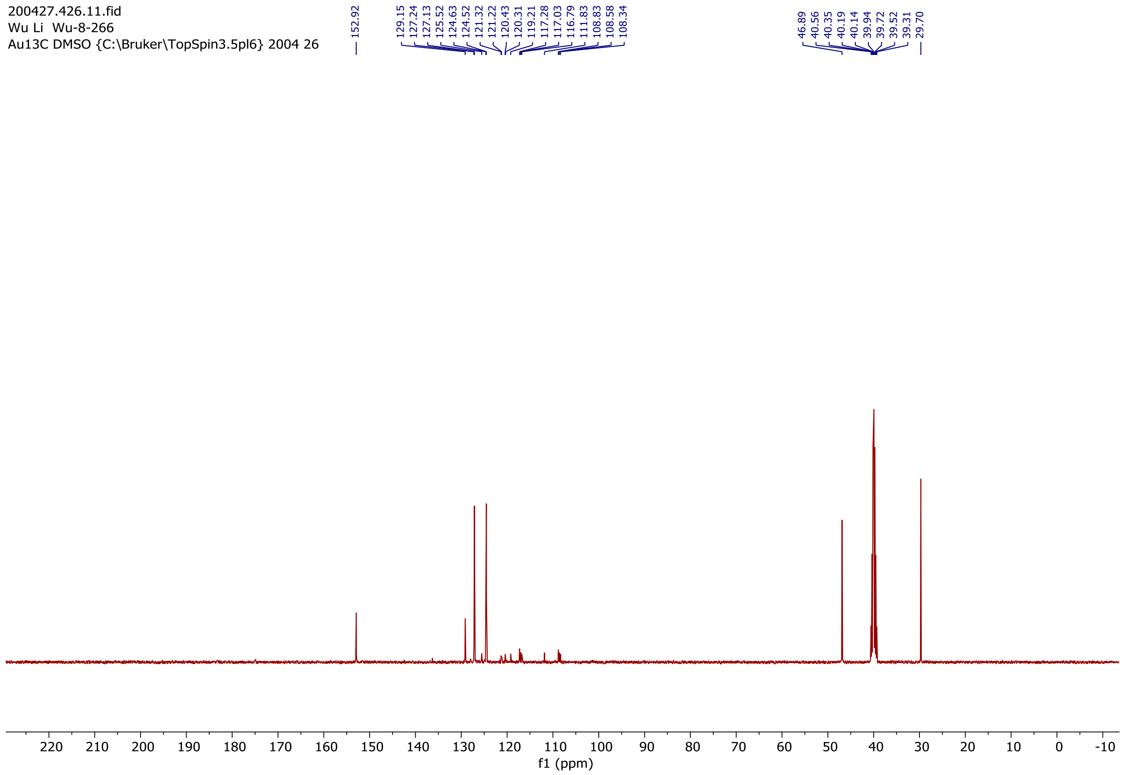

^{1}H NMR for 51a:

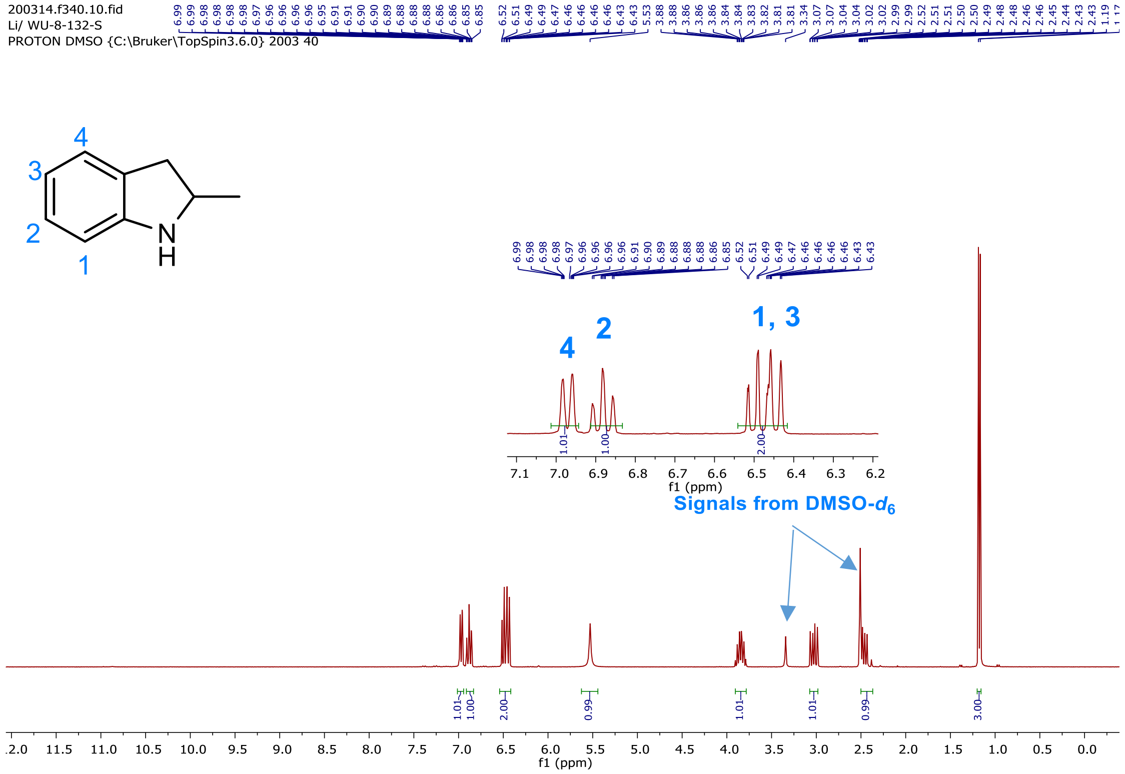

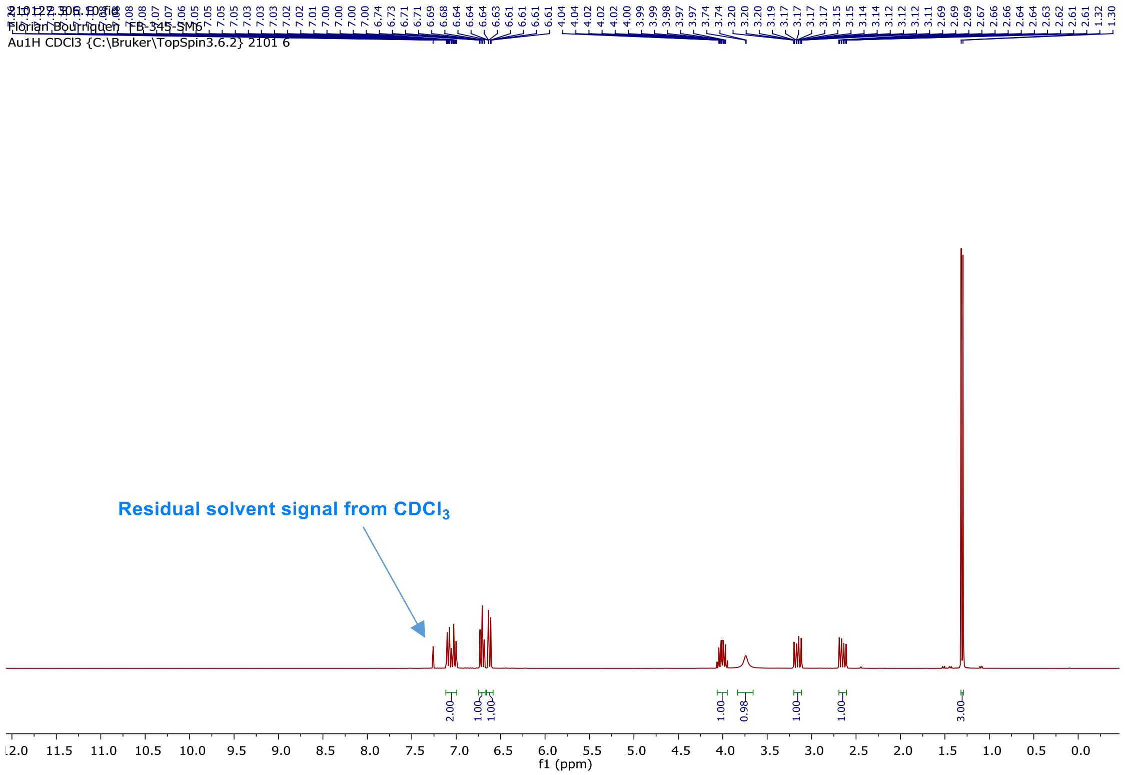

Original spectra for 51b:

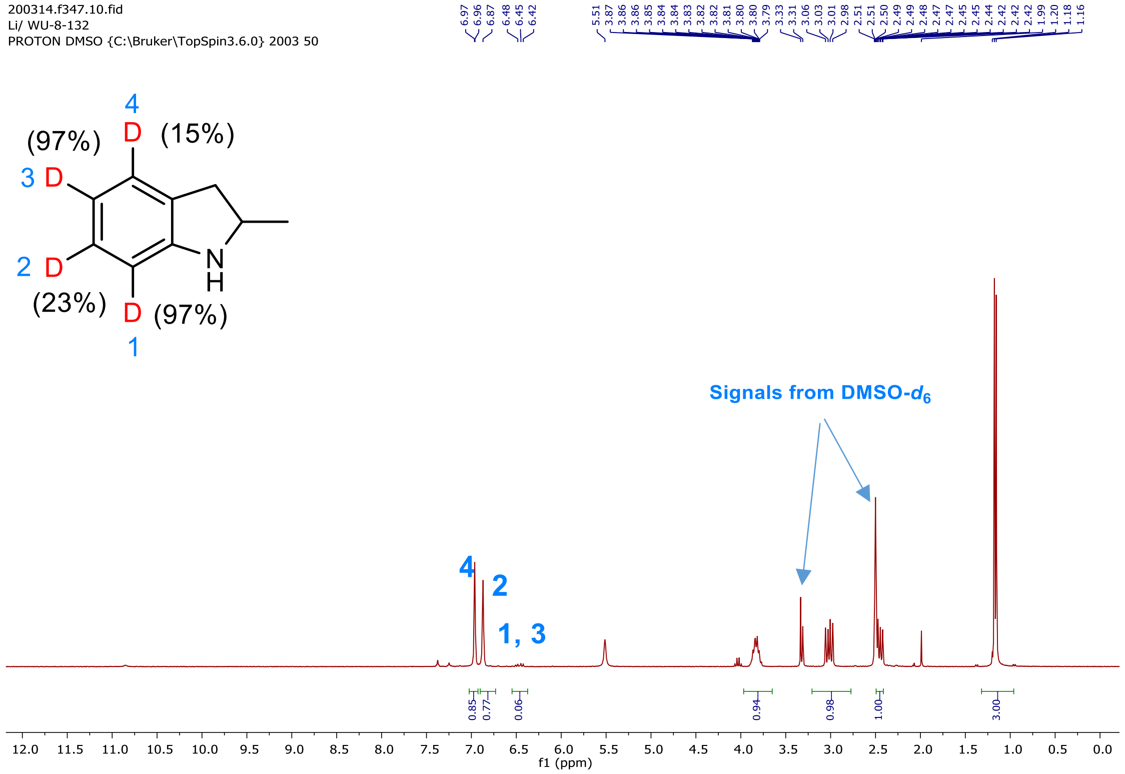

In $CDCl_3$:

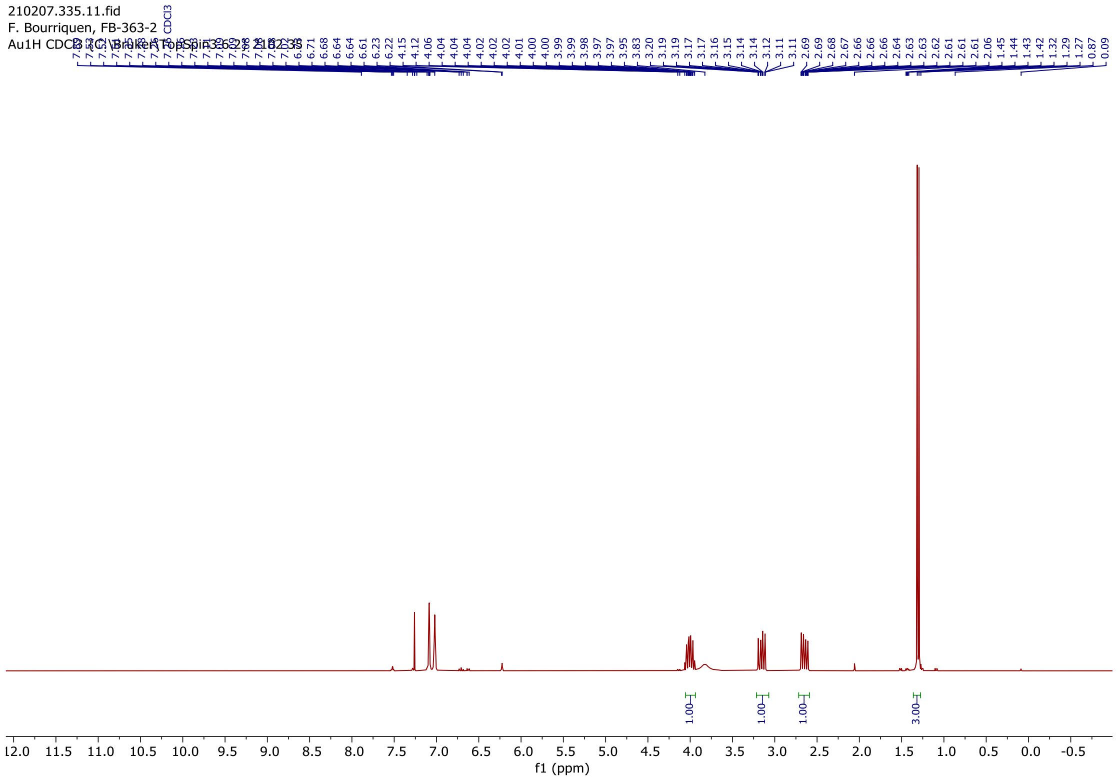

210207.335.11.fid
F. Bourriquen, FB-363-2
f1 (ppm)

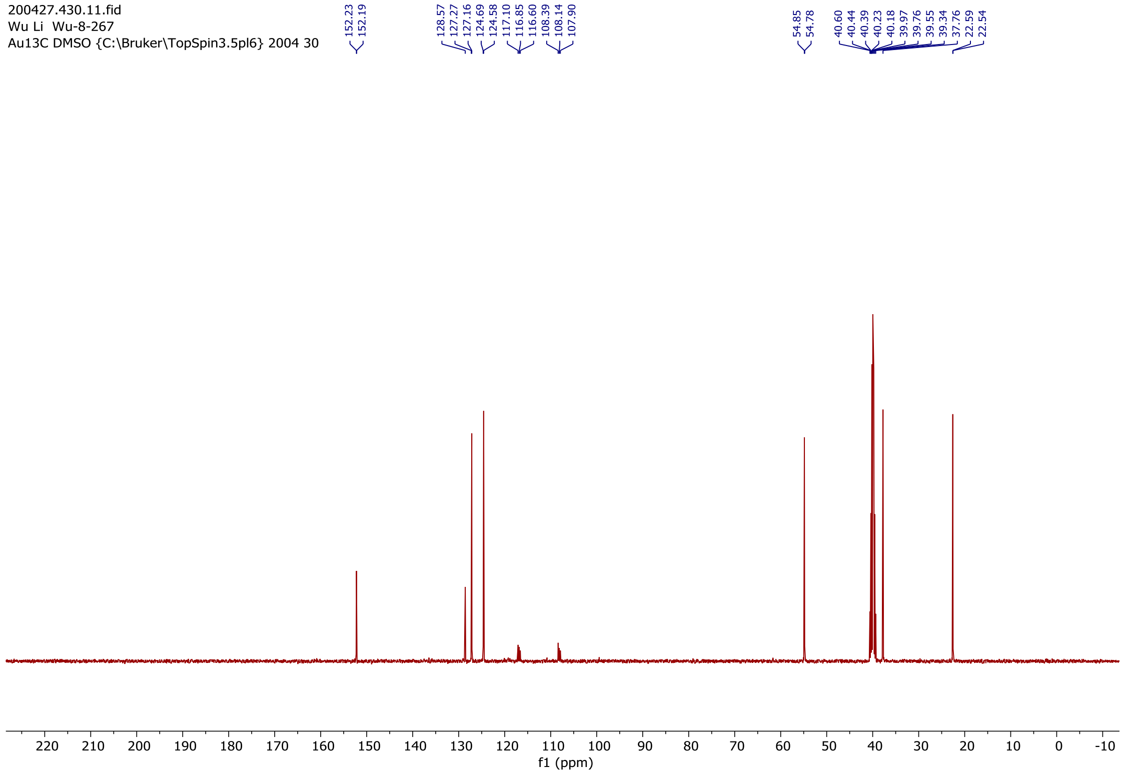

200427.430.11.fid
Wu Li Wu-8-267
Au13C DMSO {C:\Bruker\TopSpin3.5pl6} 2004 30
152.23
152.19
128.57
127.27
127.16
124.69
124.58
117.10
116.85
116.60
108.39
108.14
107.90
54.85
54.78
40.60
40.44
40.39
40.23
40.18
39.97
39.76
39.55
39.34
37.76
22.59
22.54
f1 (ppm)

^{1}H NMR for 52a:

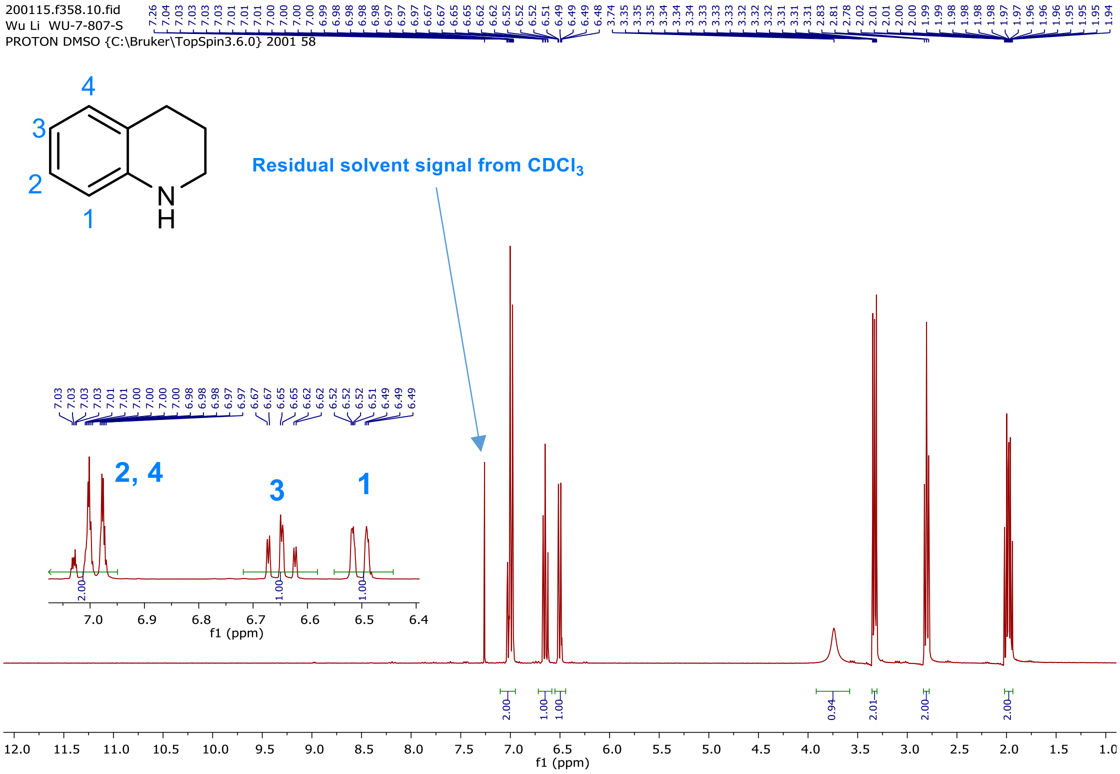

Original spectra for 52b:

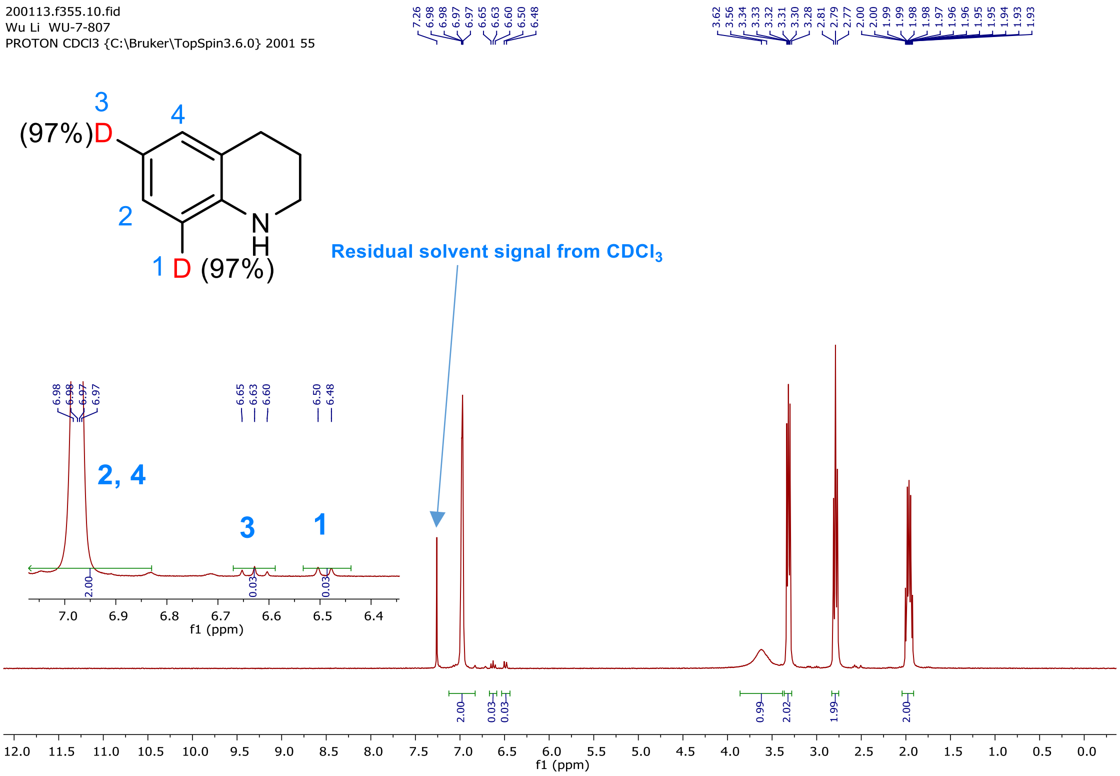

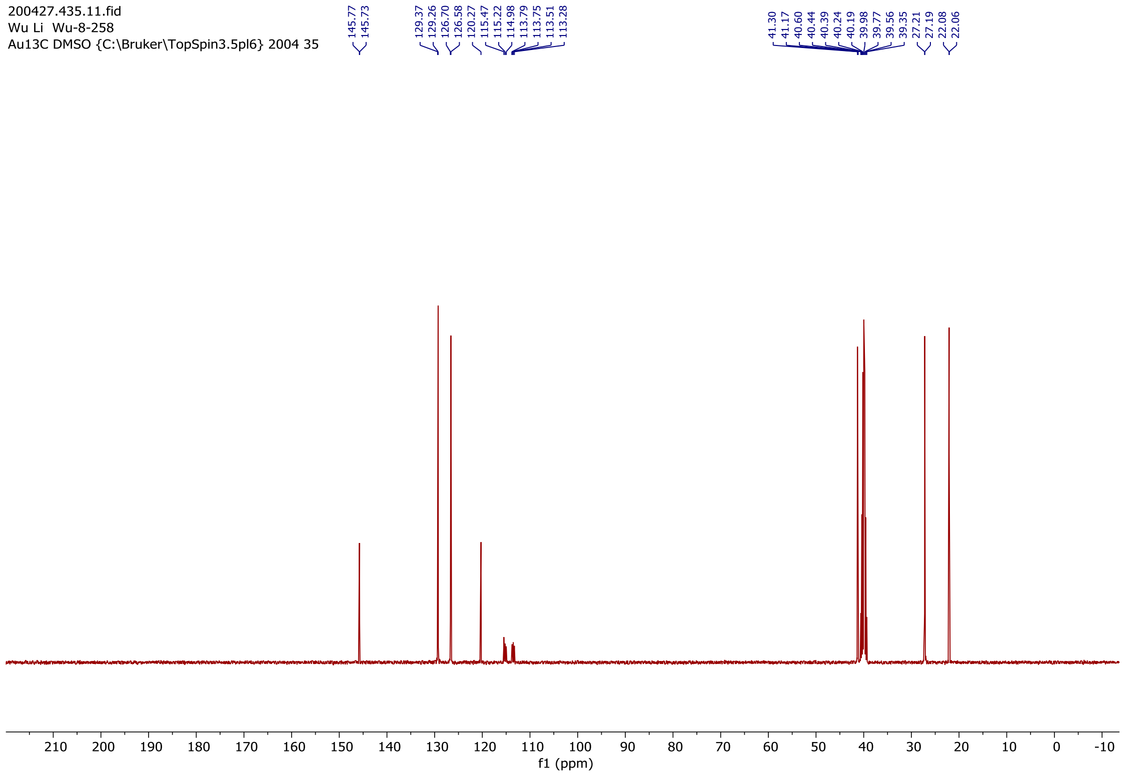

^{1}H NMR for 53a:

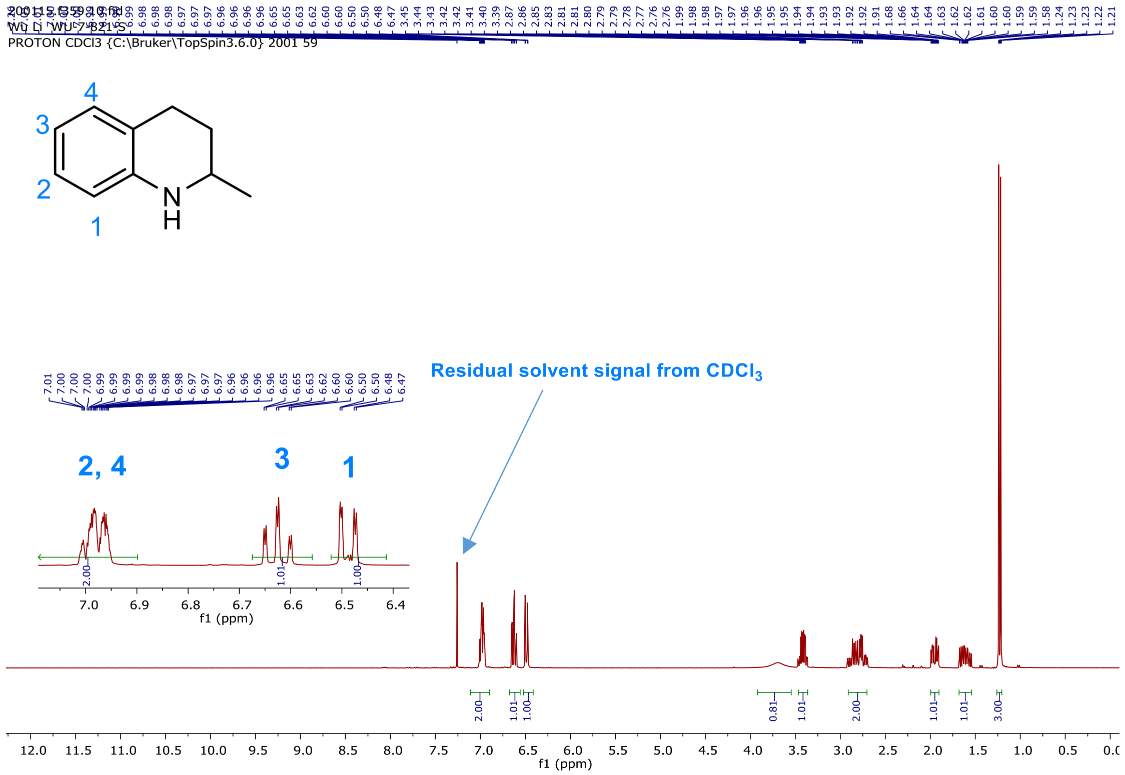

Original spectra for 53b:

200115.f364.10.fid
Wu Li WU-7-821
PROTON CDCl3 {C:\Bruker\TopSpin3.6.0} 2001 4

7.26, 6.97, 6.71, 6.65, 6.62, 6.50, 6.47, 3.47, 3.46, 3.45, 3.44, 3.43, 3.42, 3.40, 3.39, 3.38, 3.37, 3.36, 2.92, 2.90, 2.88, 2.86, 2.84, 2.82, 2.80, 2.78, 2.77, 2.76, 2.75, 2.73, 2.72, 2.71, 2.70, 1.99, 1.98, 1.97, 1.96, 1.95, 1.93, 1.92, 1.92, 1.90, 1.67, 1.65, 1.64, 1.63, 1.63, 1.62, 1.62, 1.61, 1.60, 1.60, 1.59, 1.58, 1.57, 1.56, 1.54, 1.24, 1.22

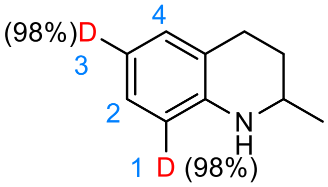

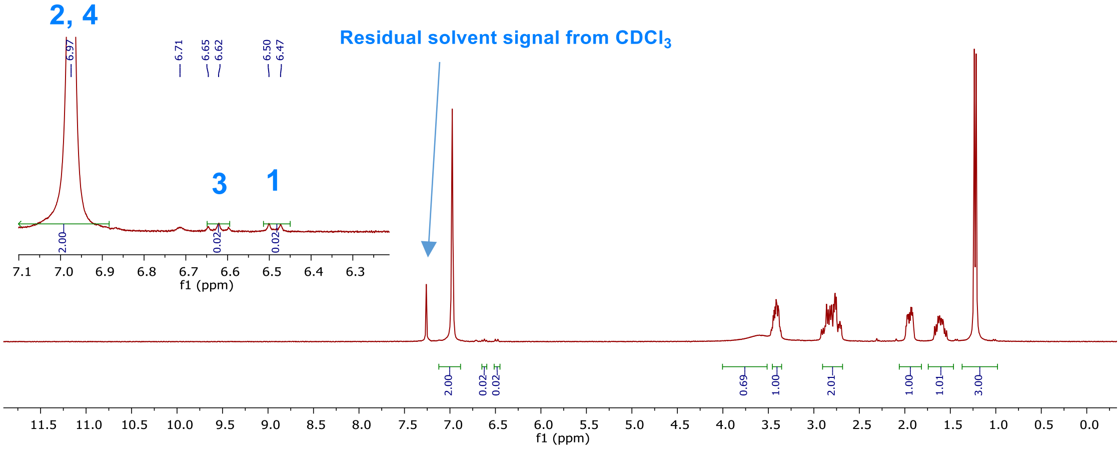

200427.436.11.fid
Wu Li Wu-8-258
Au13C DMSO {C:\Bruker\TopSpin3.5pl6} 2004 36

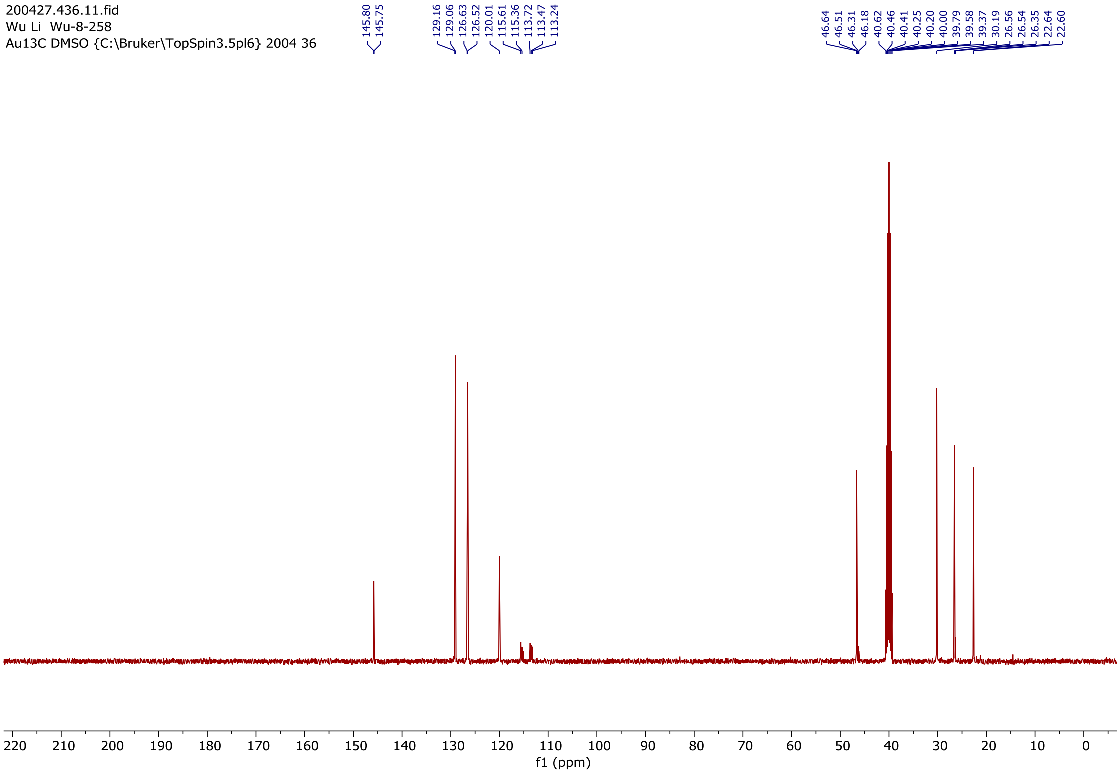

^{1}H NMR for 54a:

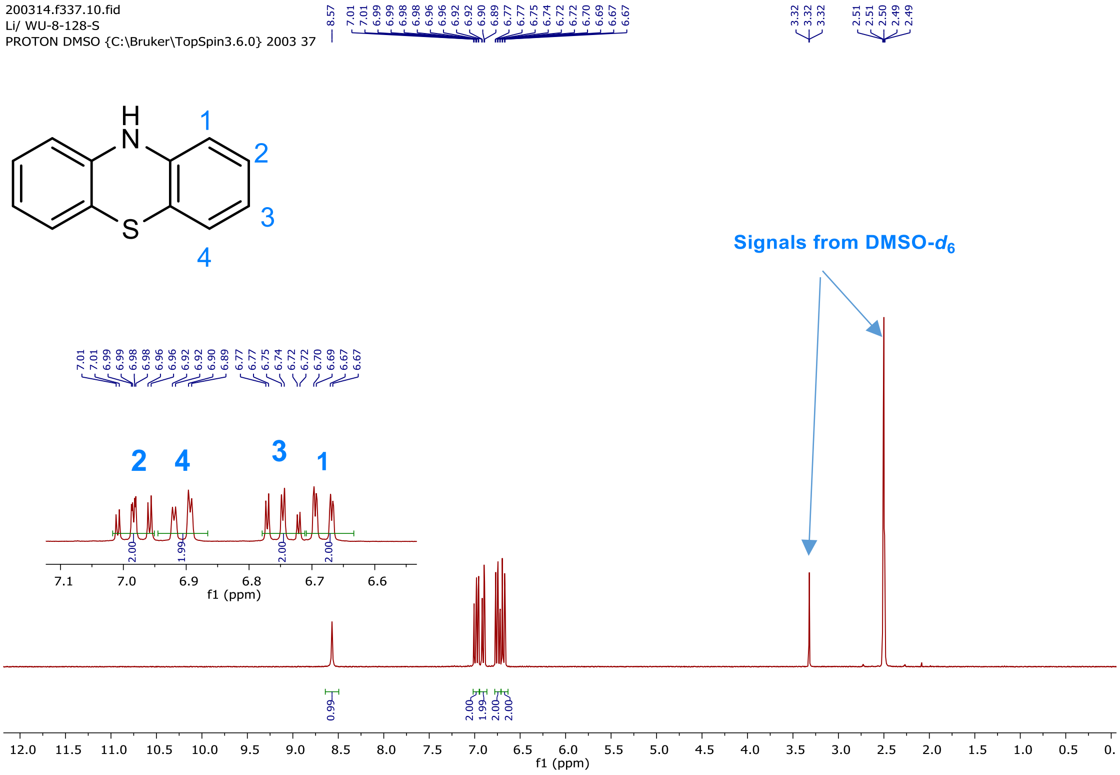

Original spectra for 54b:

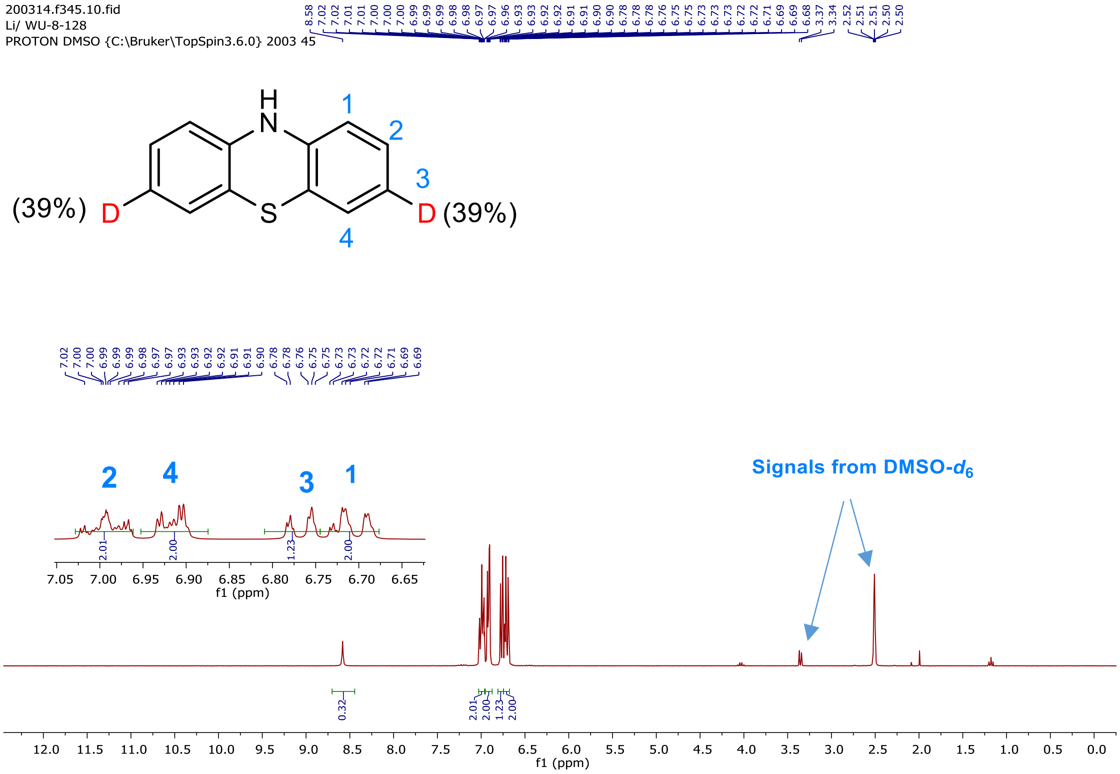

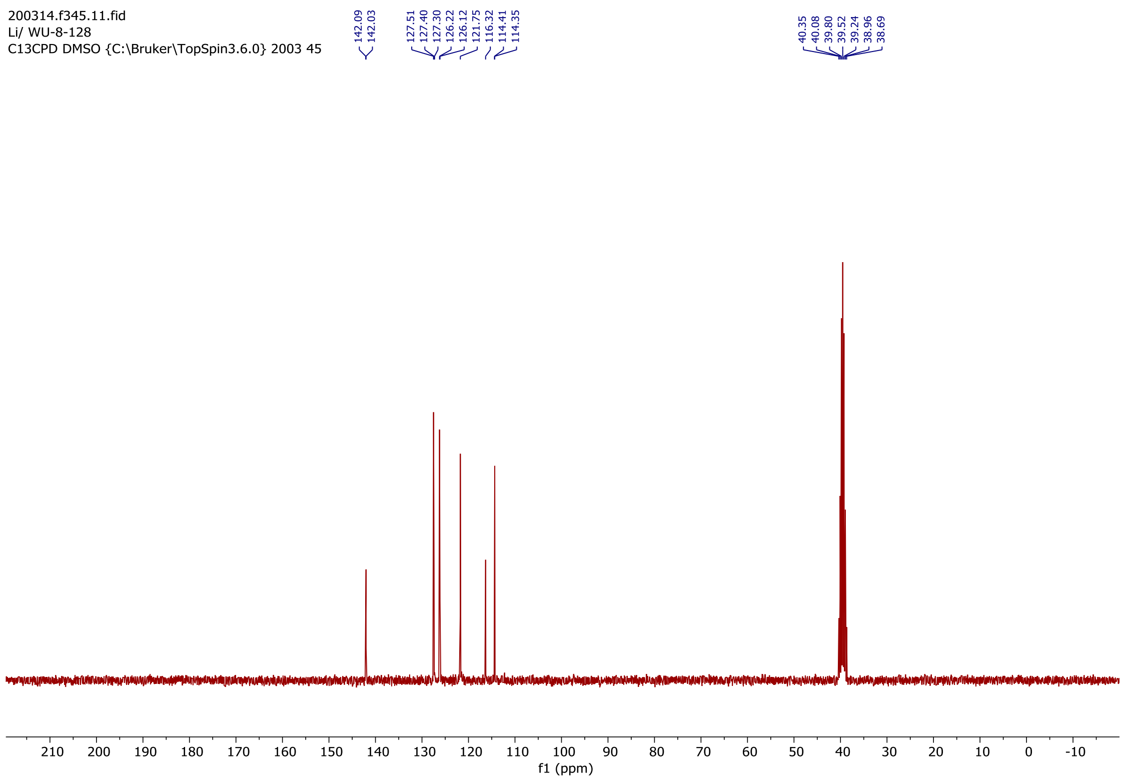

^{1}H NMR for 55a:

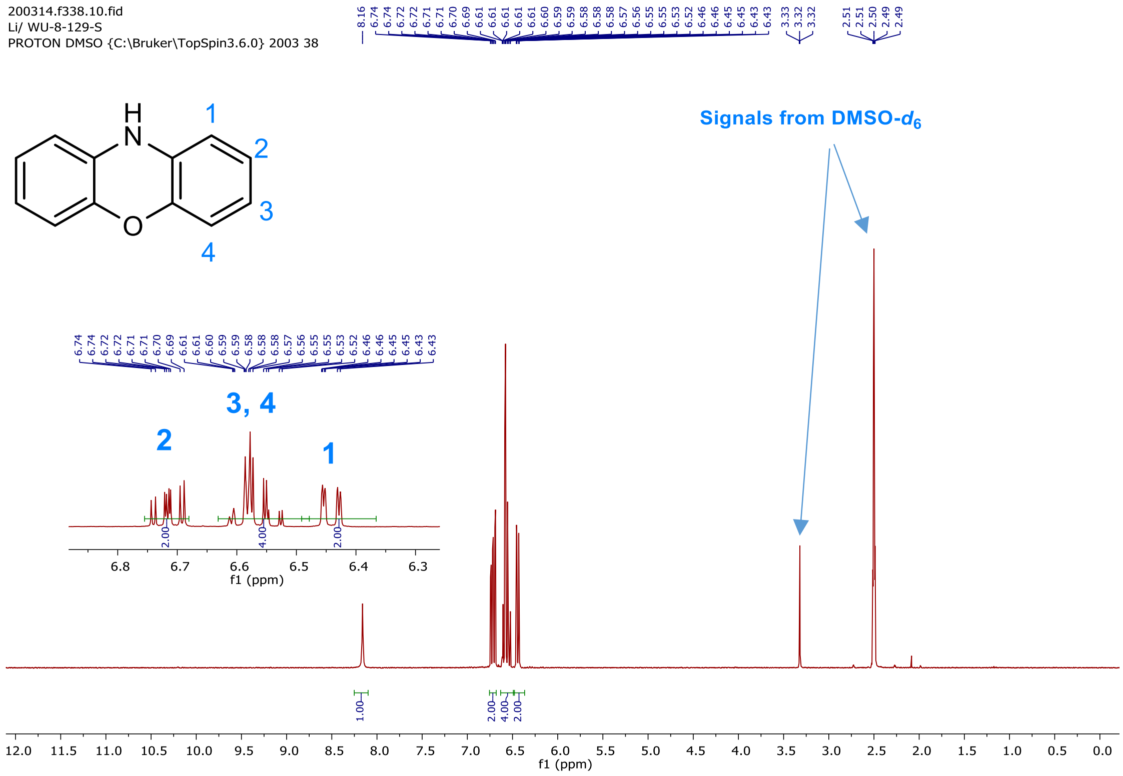

Original spectra for 55b:

200314.f349.10.fid
Li/ WU-8-129
PROTON DMSO {C:\Bruker\TopSpin3.6.0} 2003 49

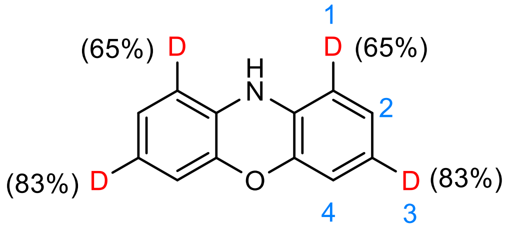

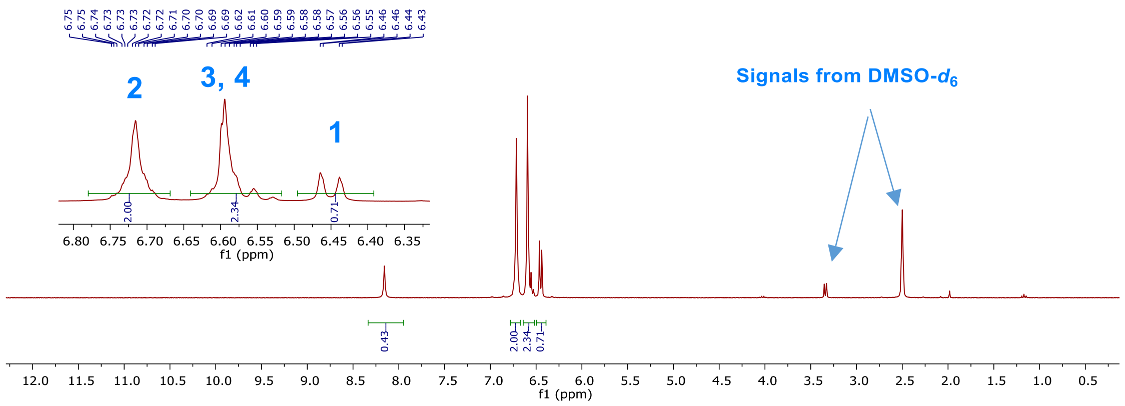

200314.f346.11.fid
Li/ WU-8-129
C13CPD DMSO {C:\Bruker\TopSpin3.6.0} 2003 49

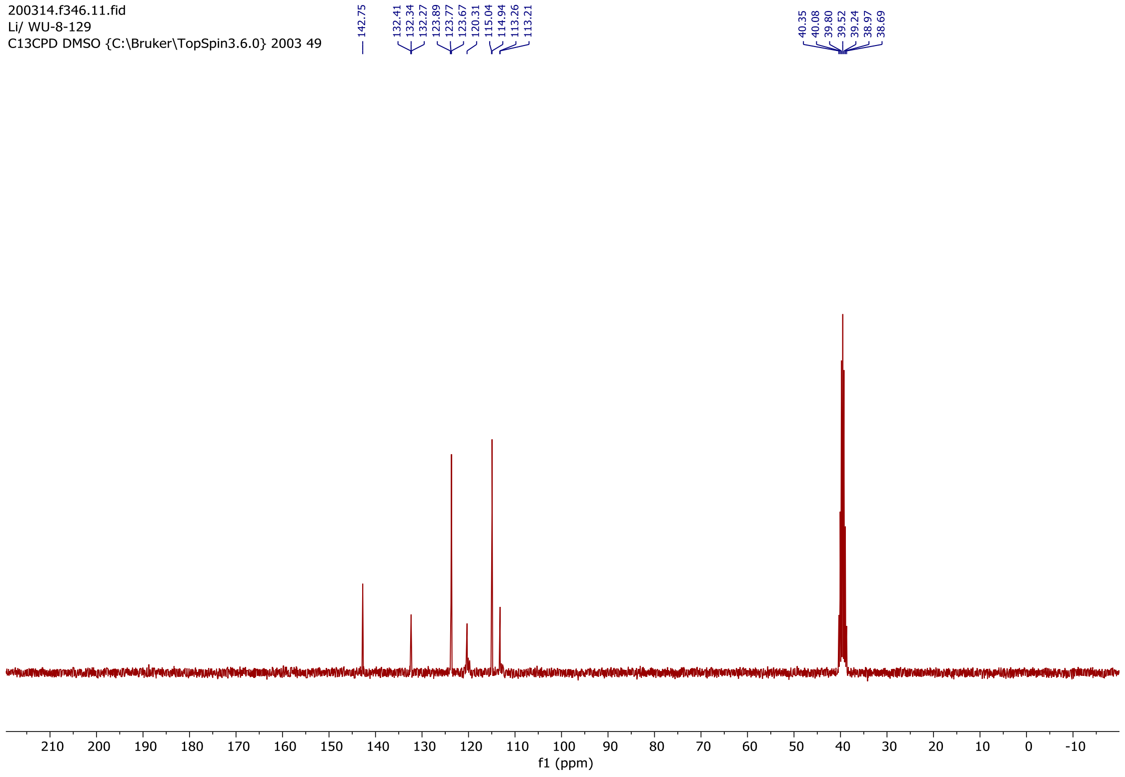

^{1}H NMR for 56a:

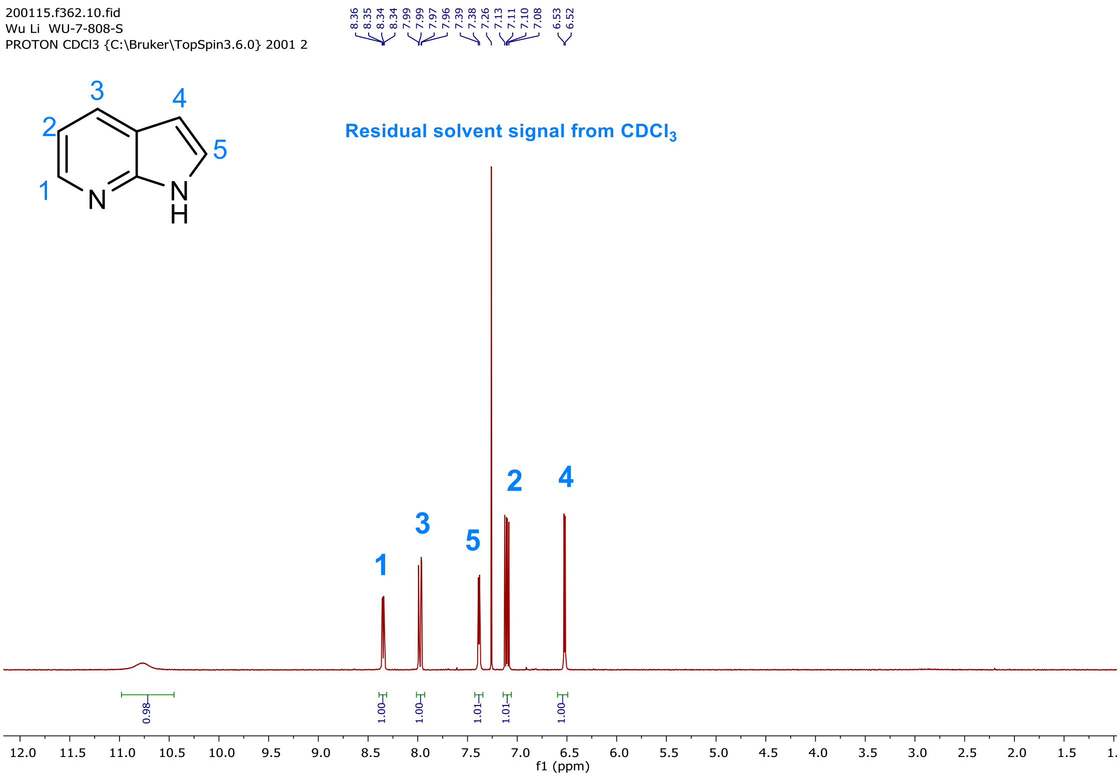

Original spectra for 56b:

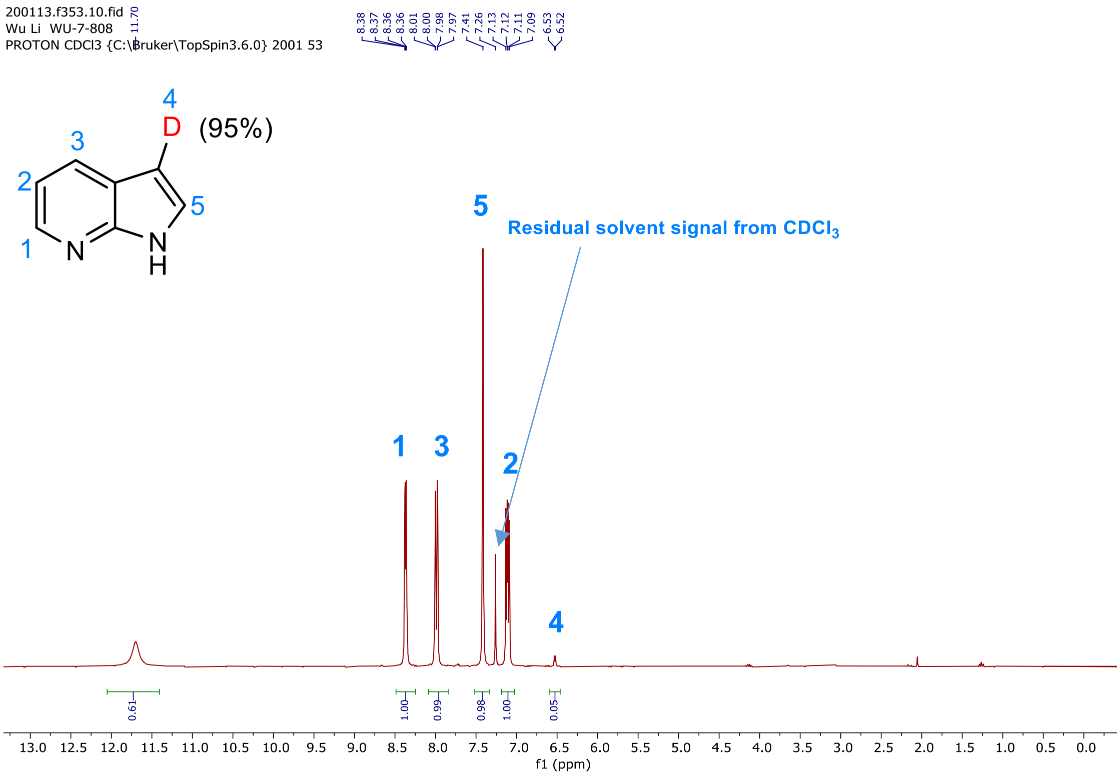

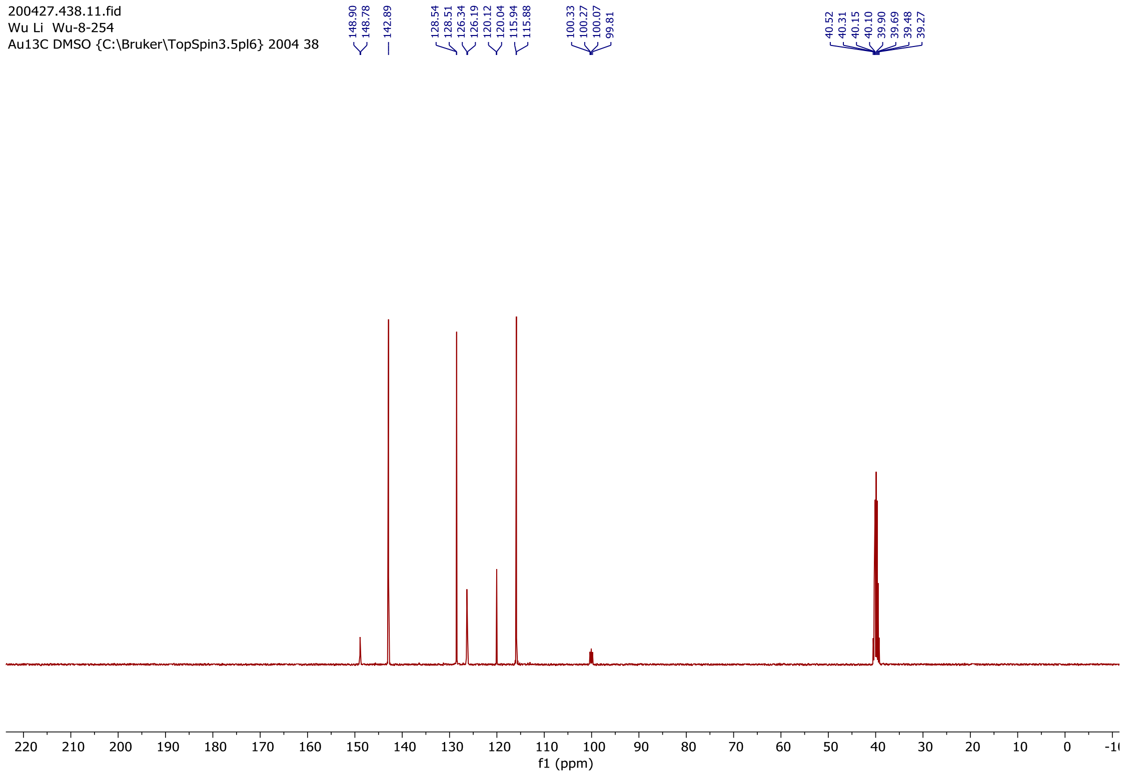

^{1}H NMR for 57a:

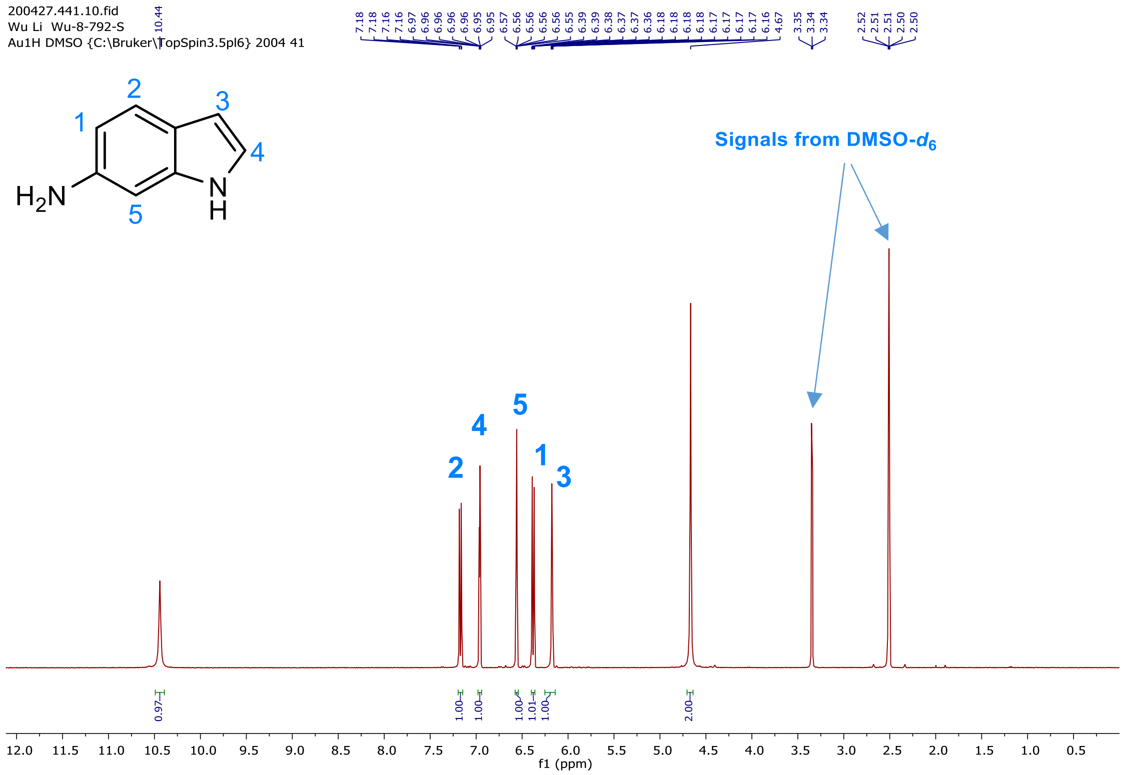

Original spectra for 57b:

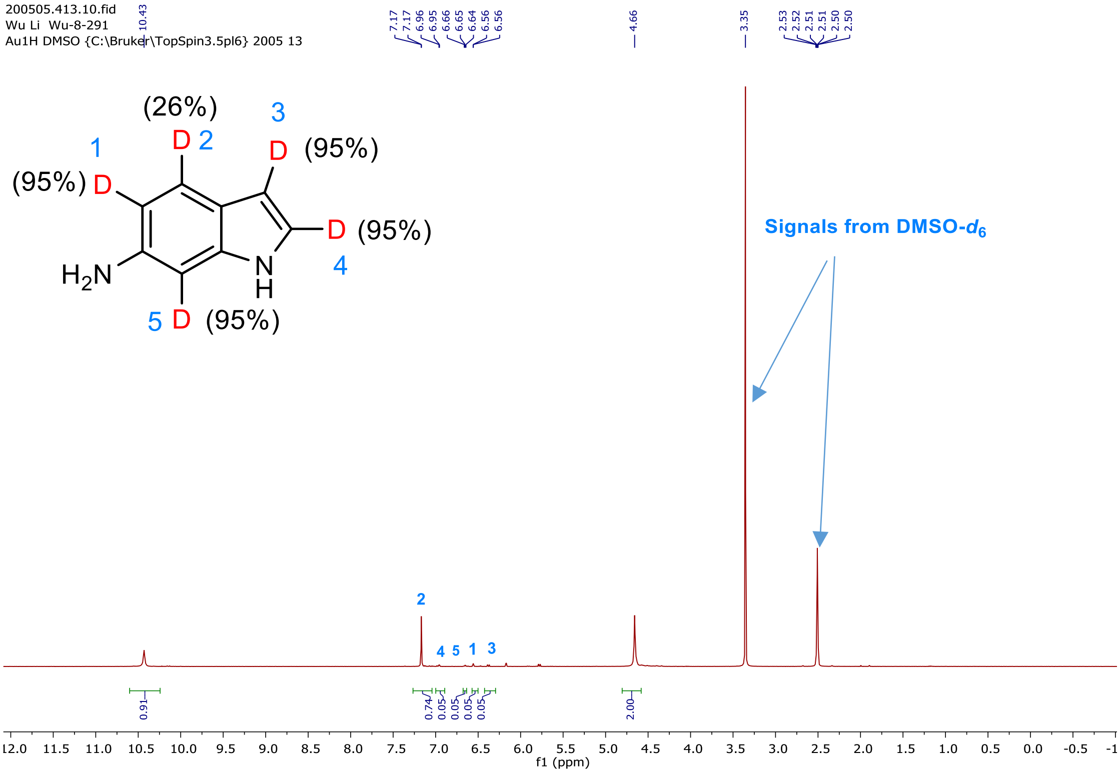

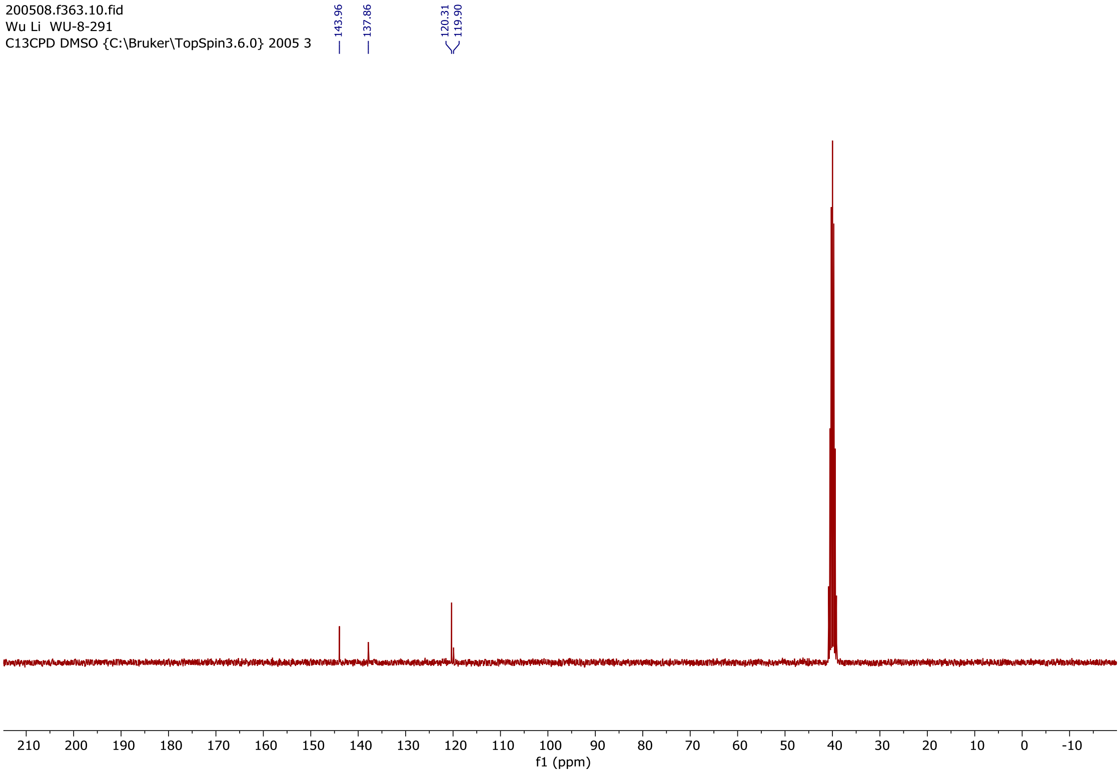

^{1}H NMR for 58a:

In $CDCl_3$:

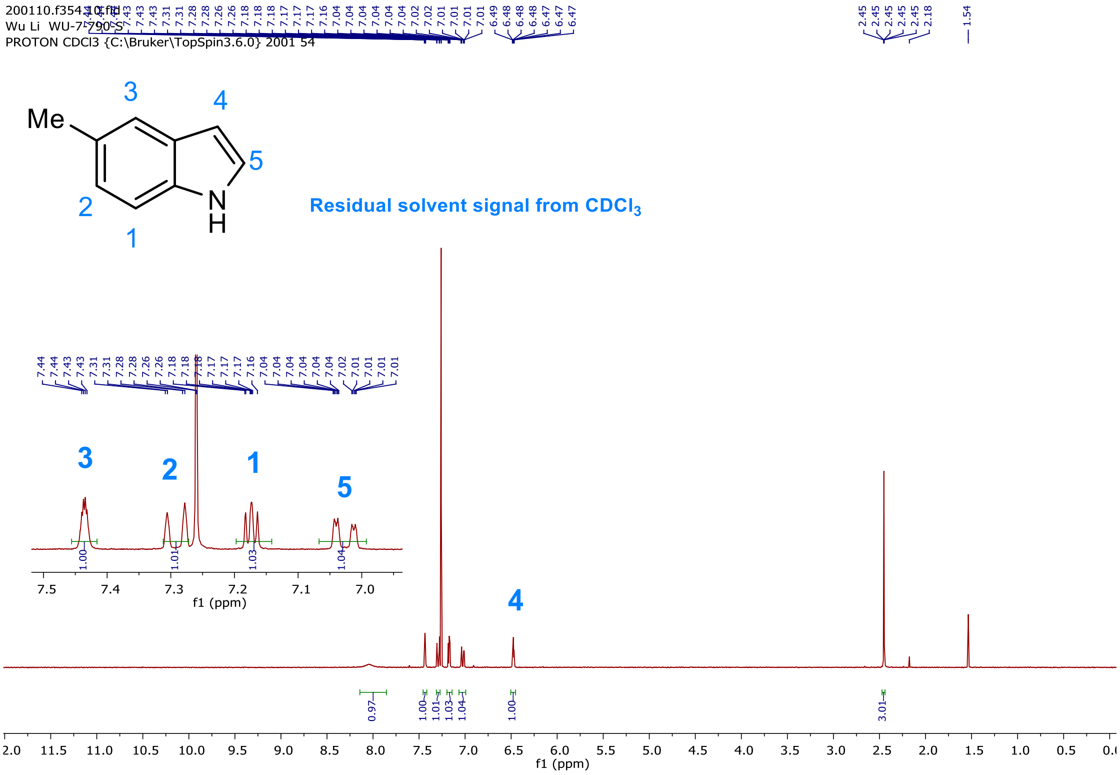

In DMSO-d_6:

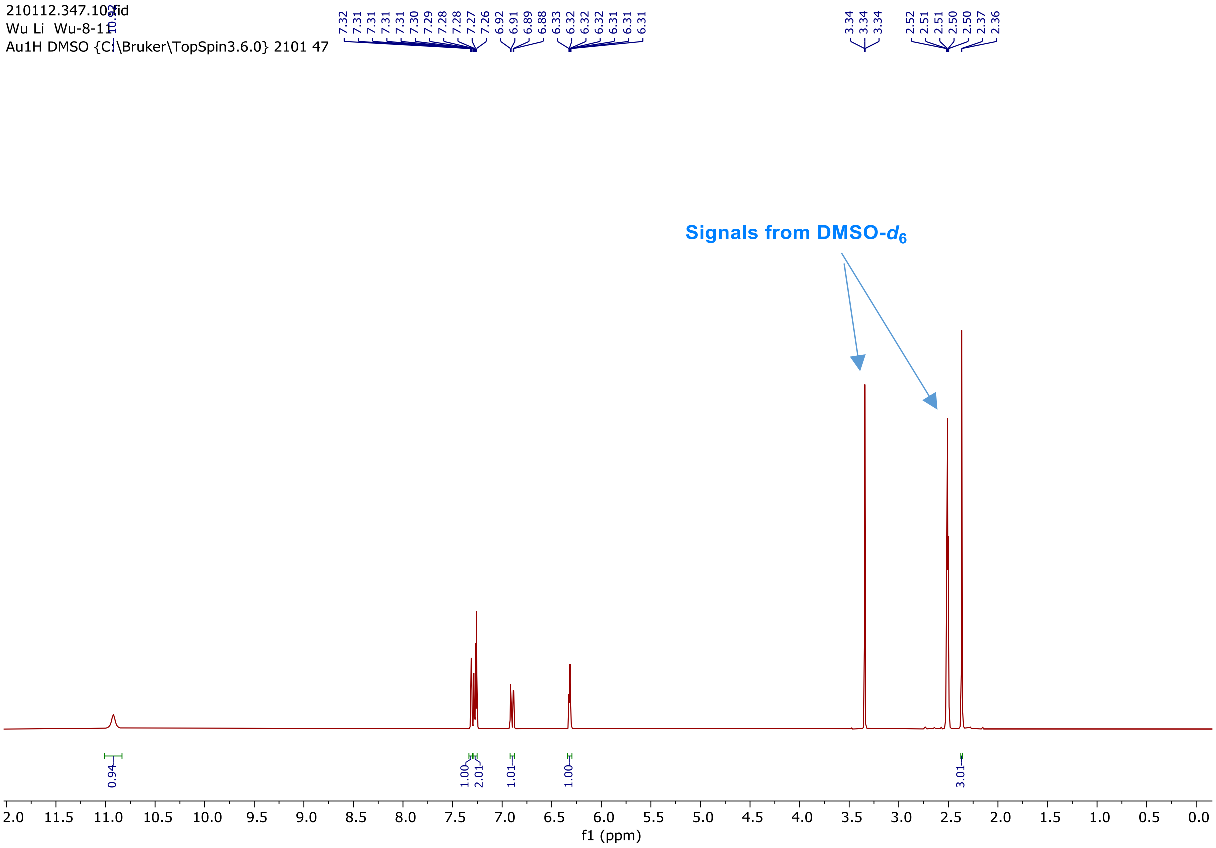

Original spectra for 58b:

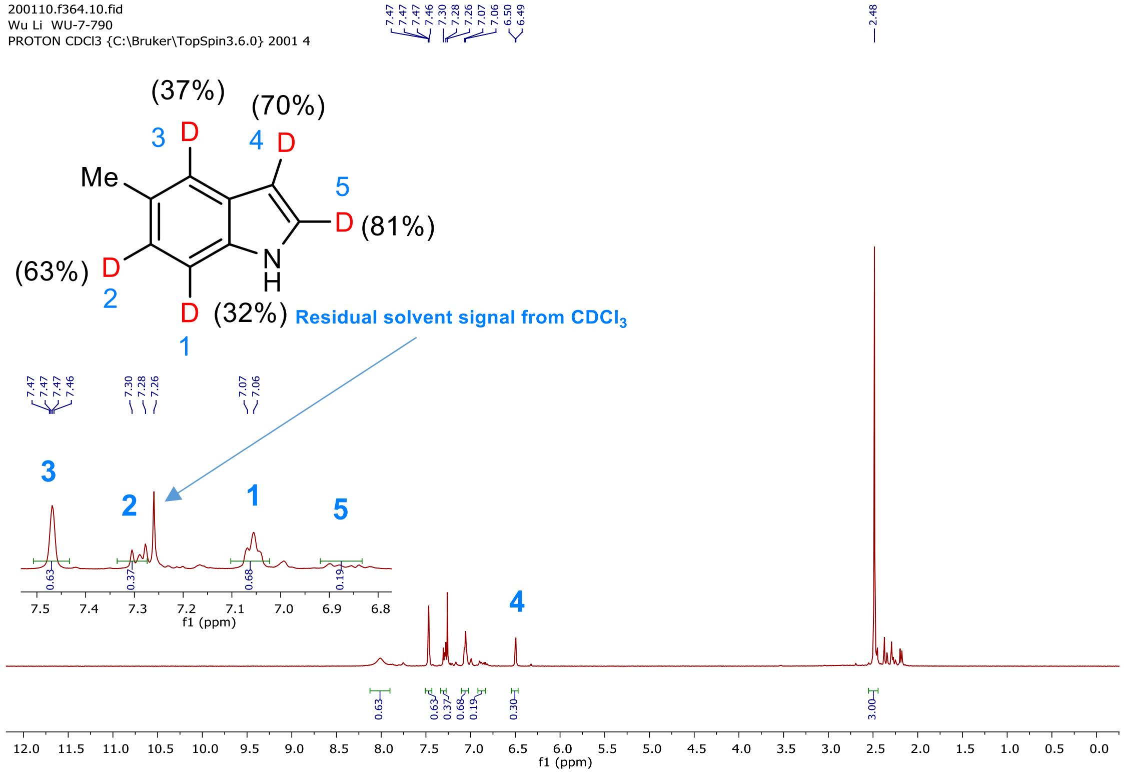

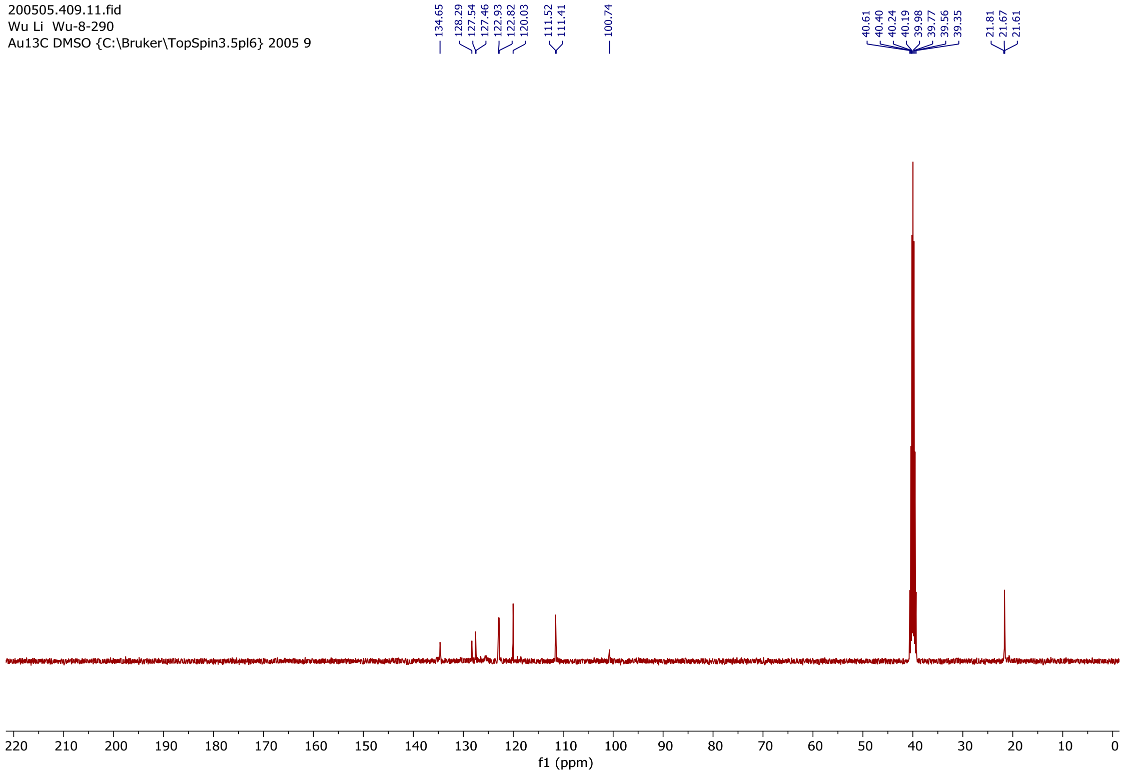

^{1}H NMR for 59a:

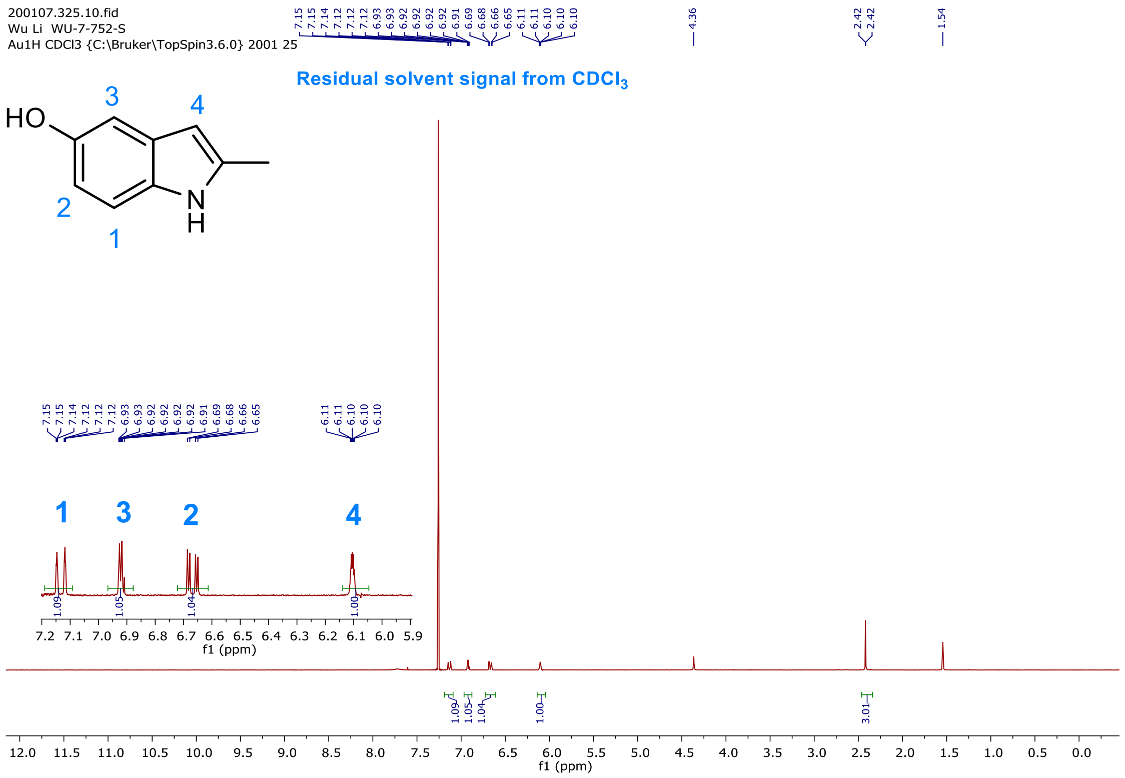

Original spectra for 59b:

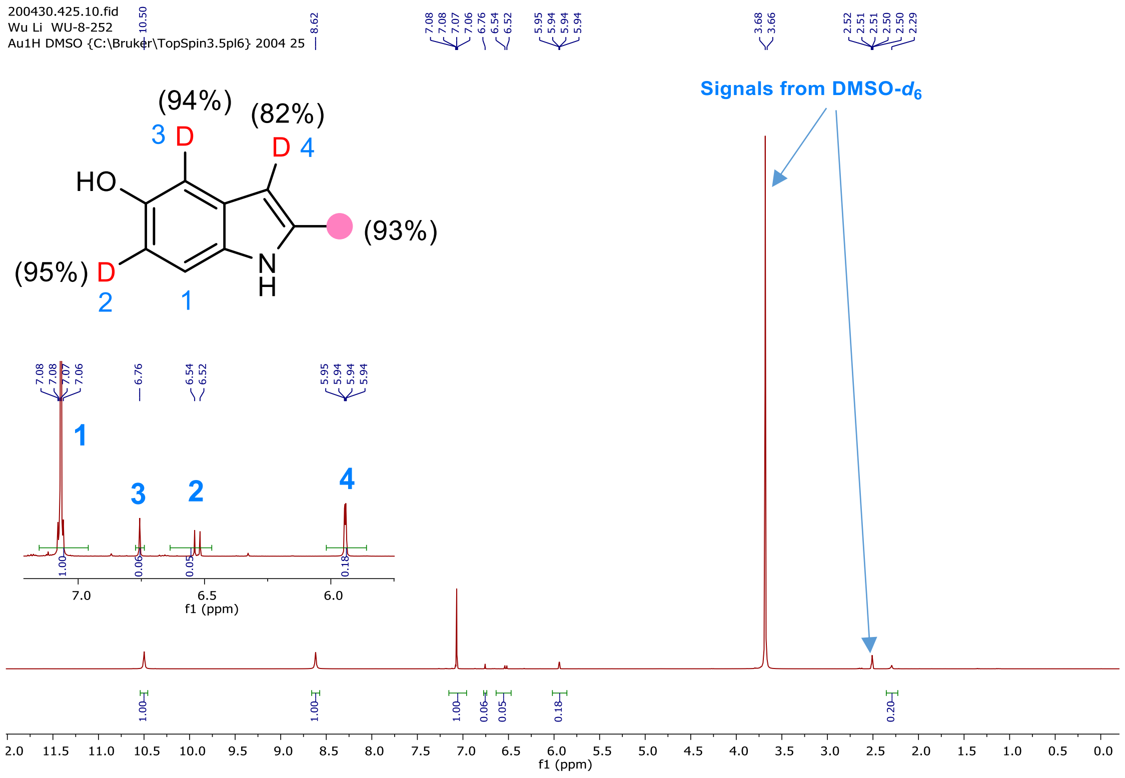

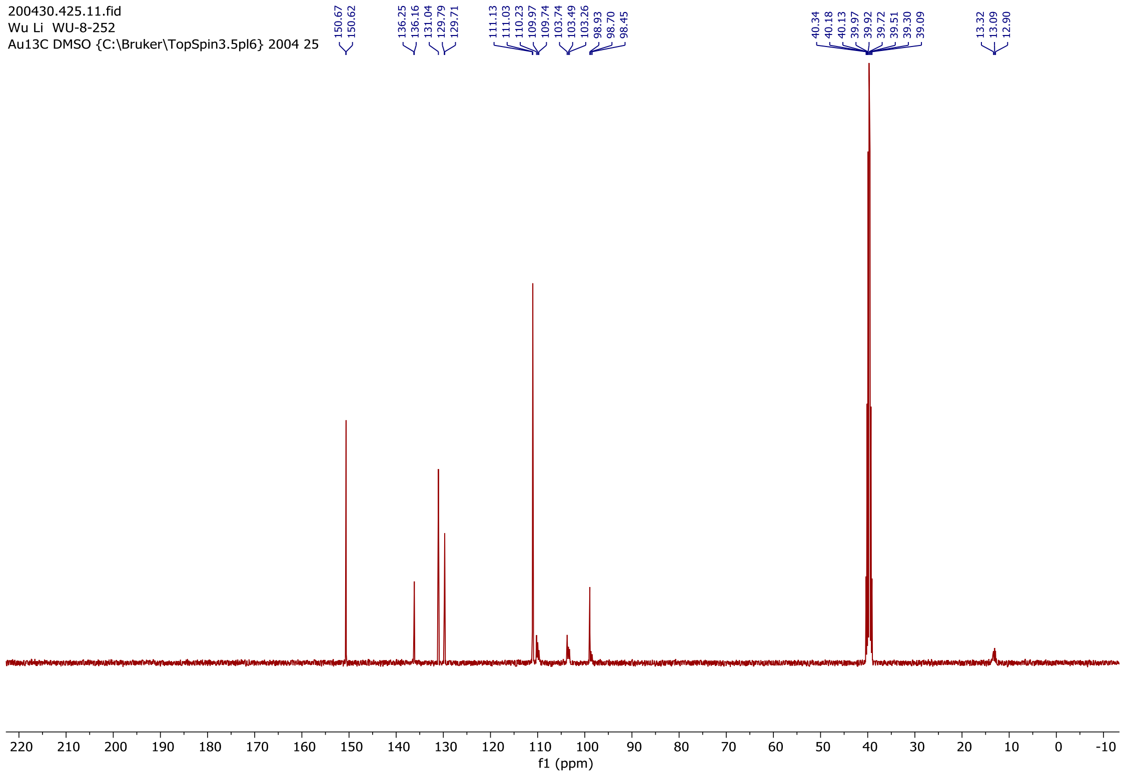

^{1}H NMR for 60a:

In $CDCl_3$:

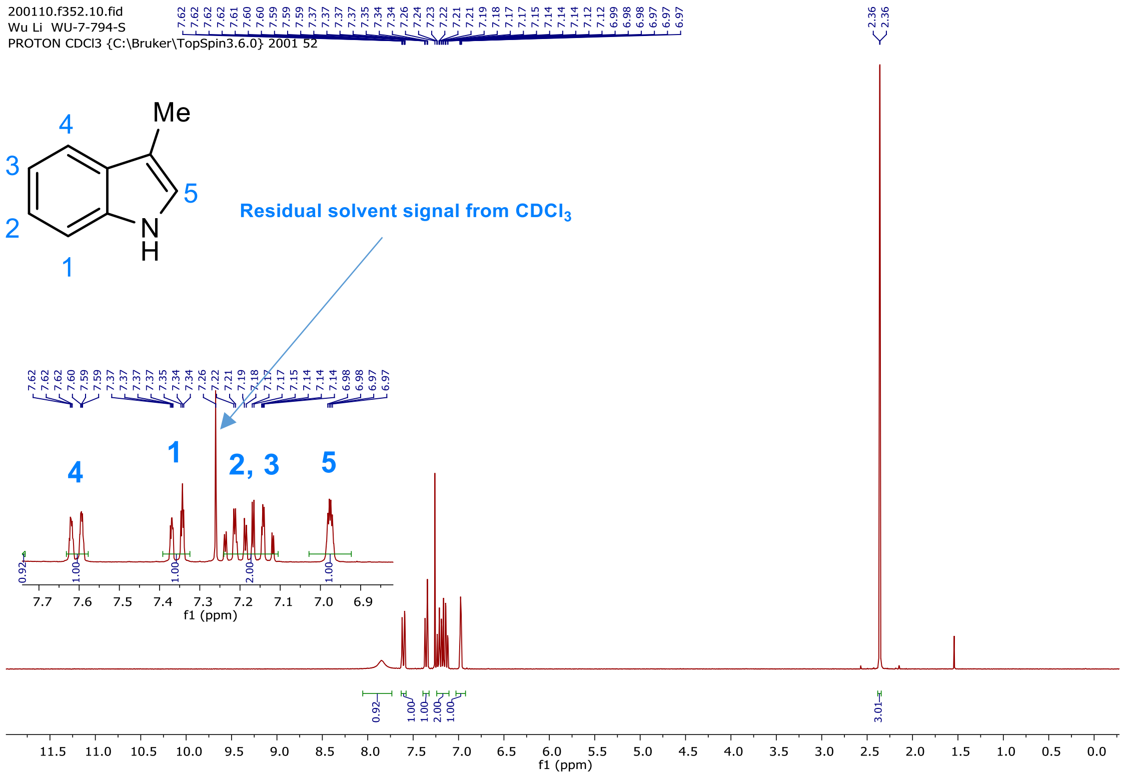

In DMSO-d_6:

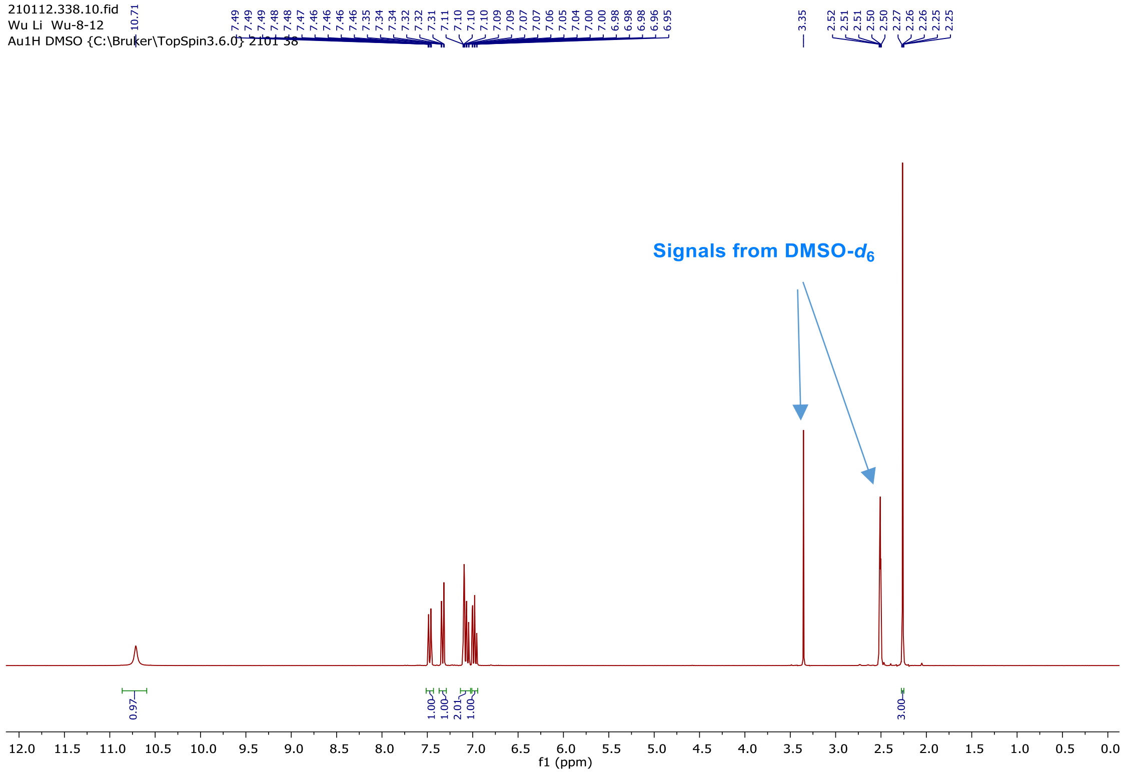

Original spectra for 60b:

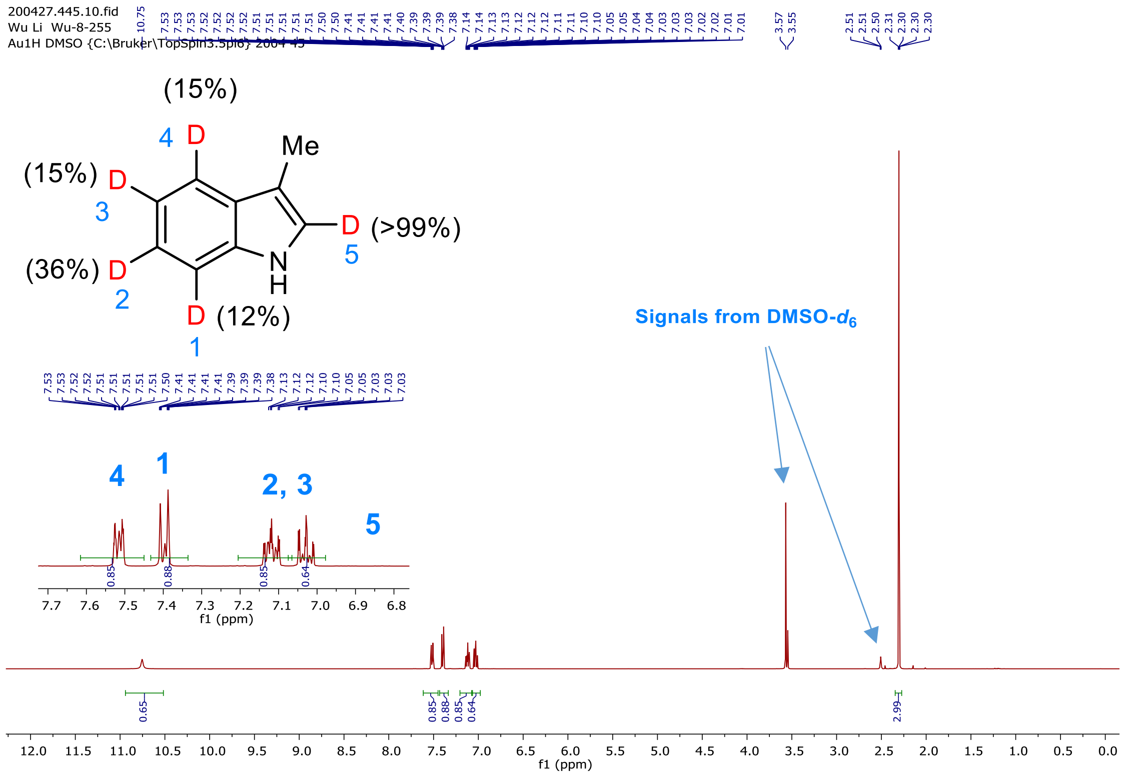

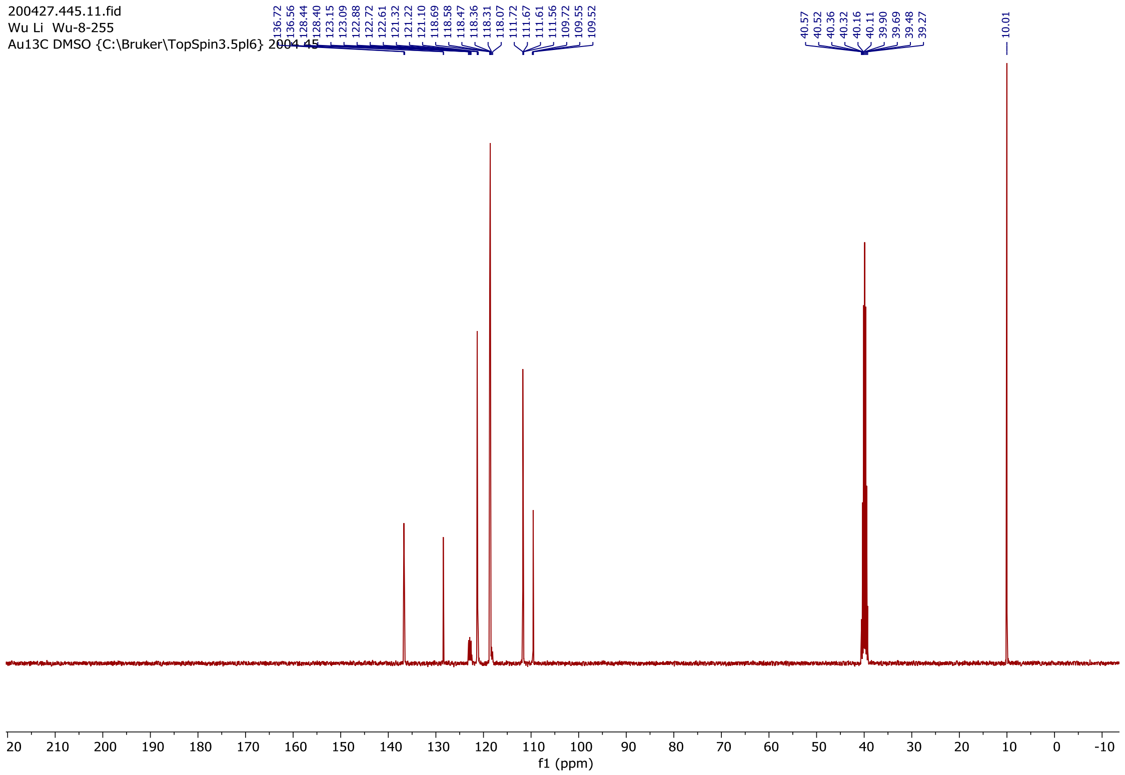

^{1}H NMR for 61a:

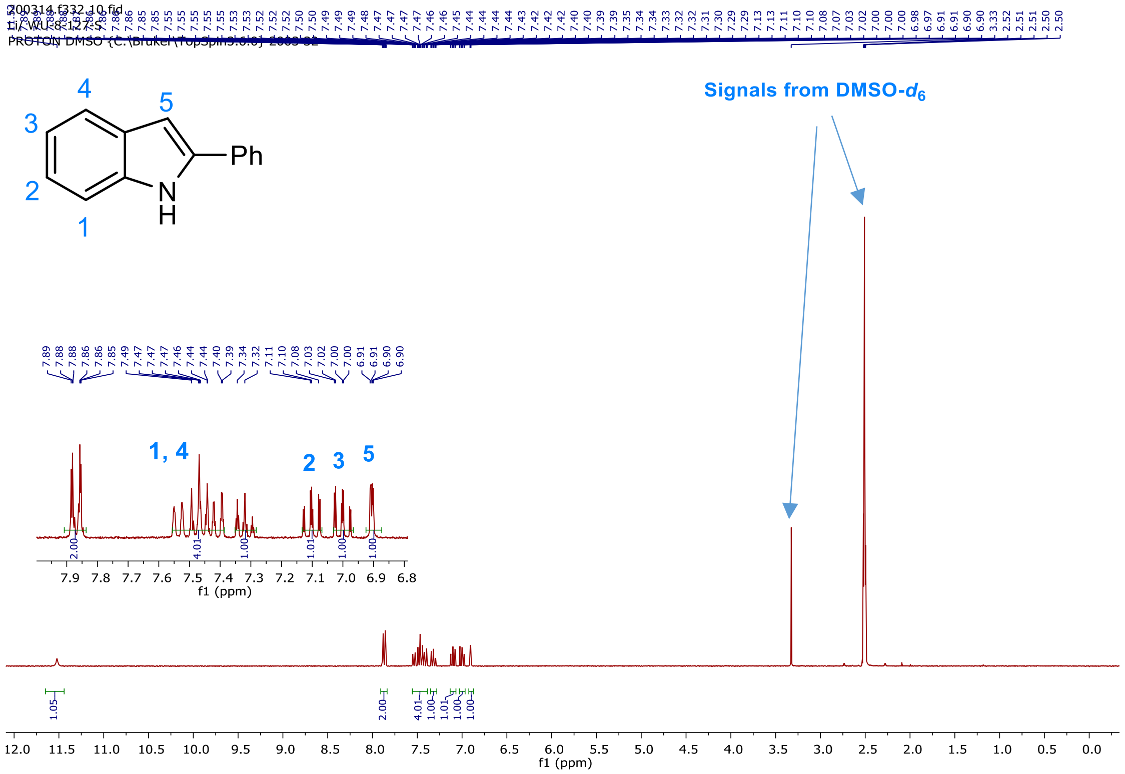

Original spectra for 61b:

200314.f344.10.fid
Li/ WU-8-127
PROTON DMSO {C:\Bruker\TopSpin3.6.0} 2003 44

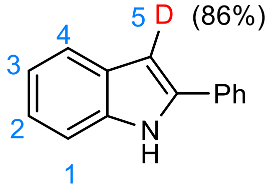

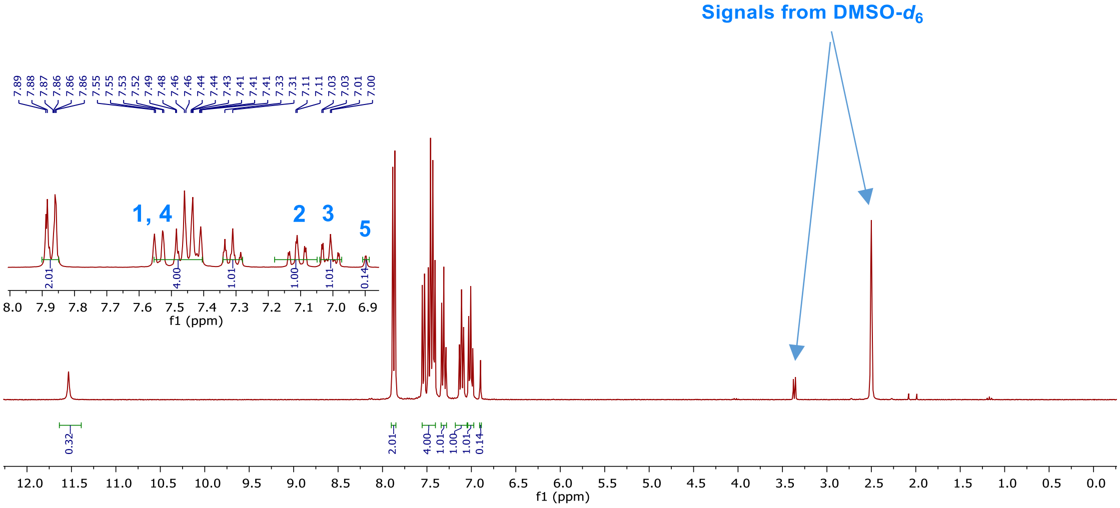

200314.f344.11.fid
Li/ WU-8-127
C13CPD DMSO {C:\Bruker\TopSpin3.6.0} 2003 44

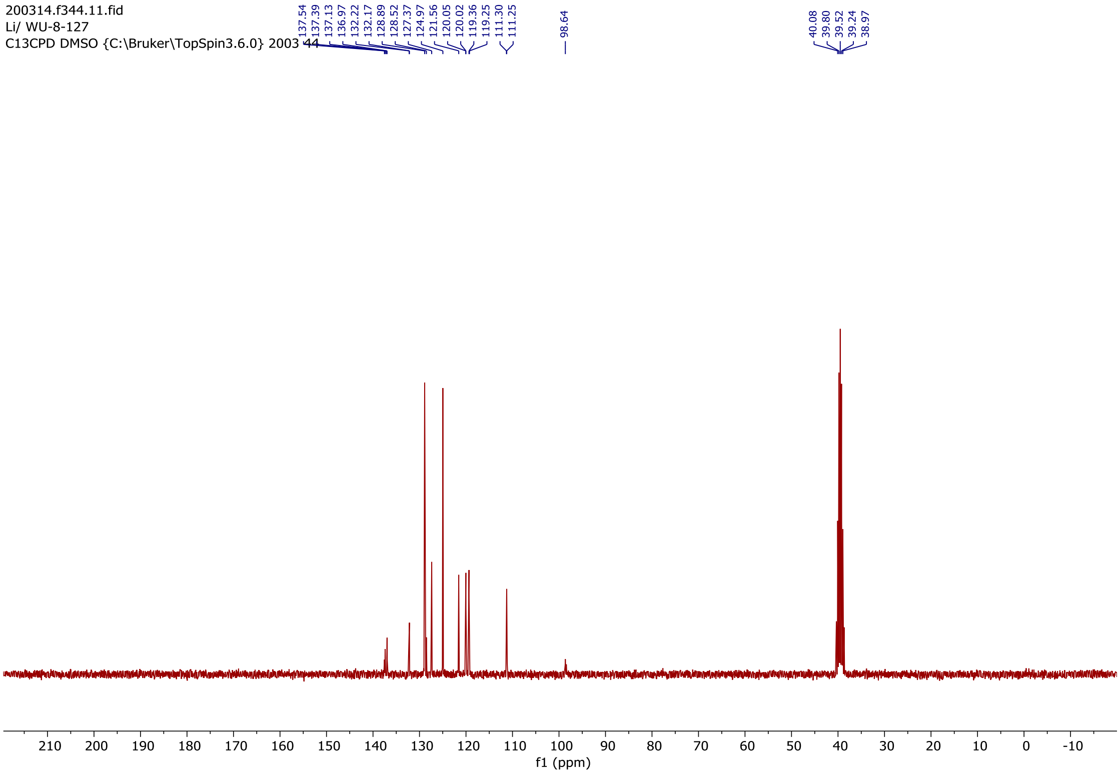

^{1}H NMR for 62a:

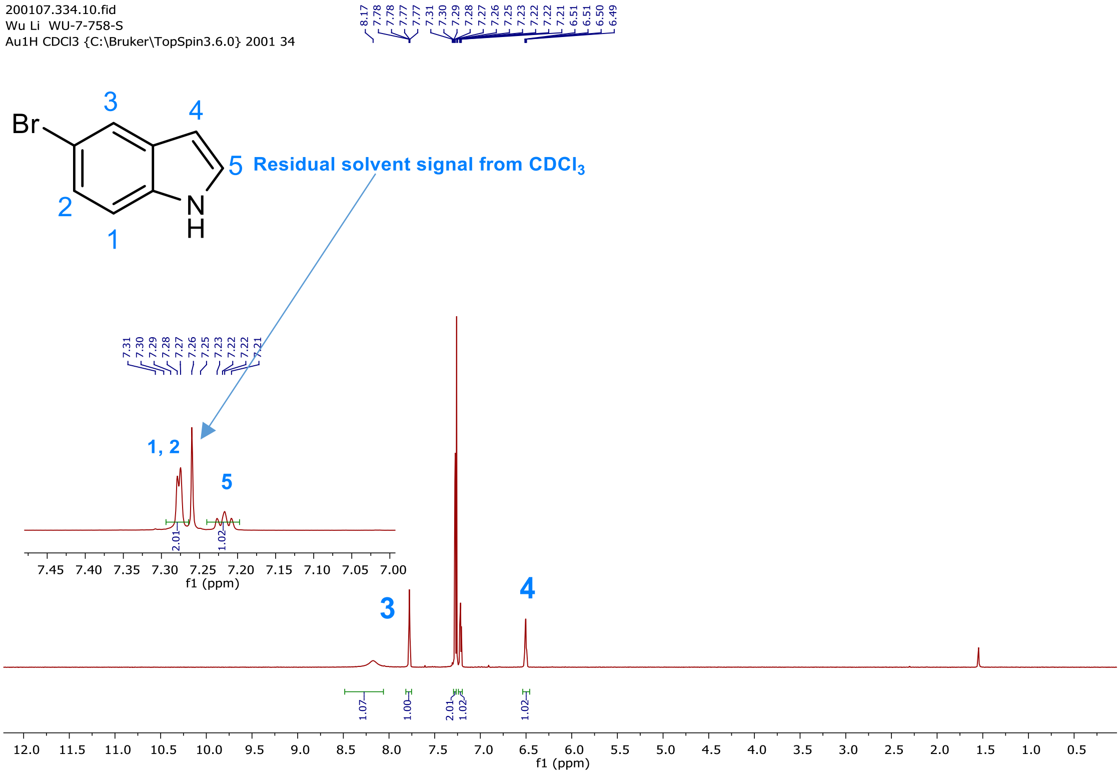

Original spectra for 62b:

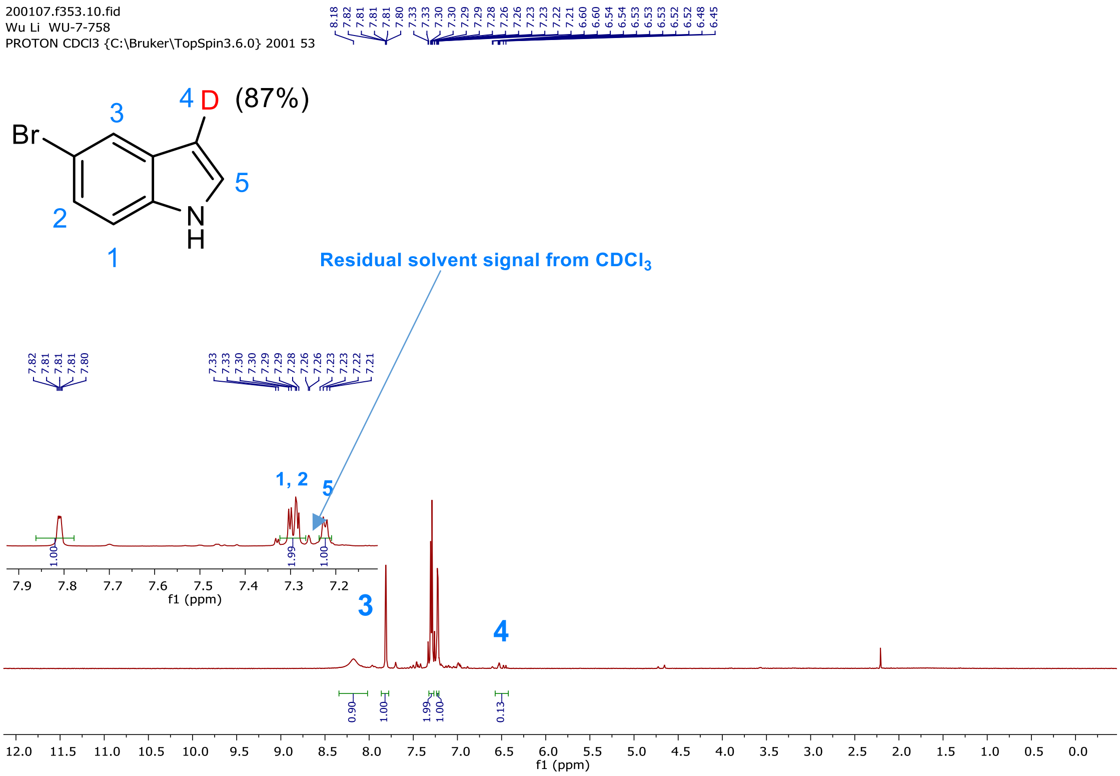

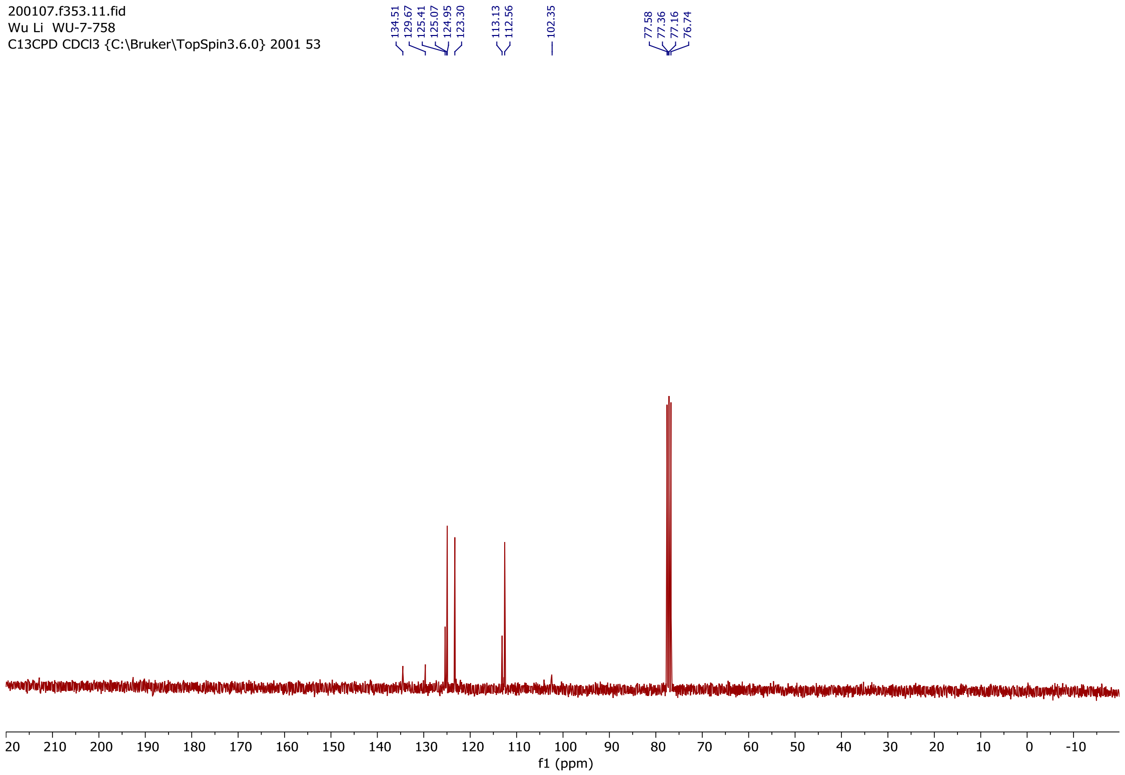

^{1}H NMR for 63a:

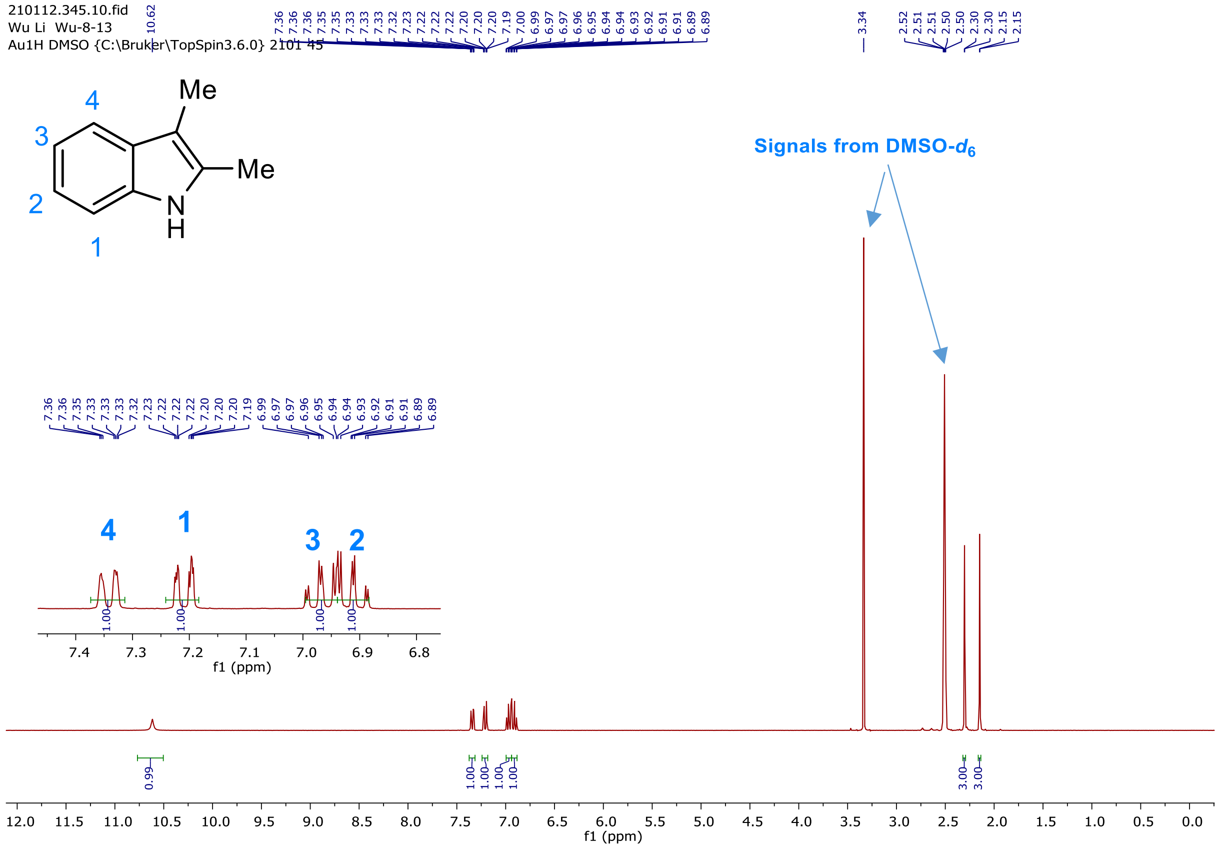

Original spectra for 63b:

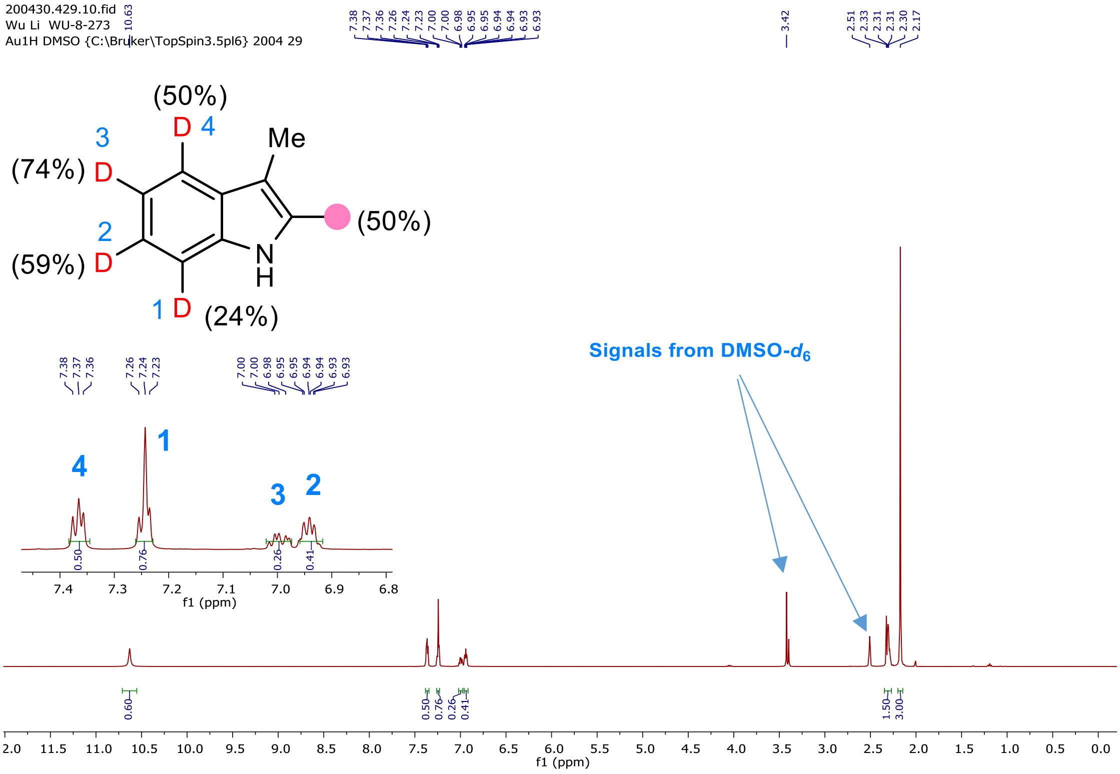

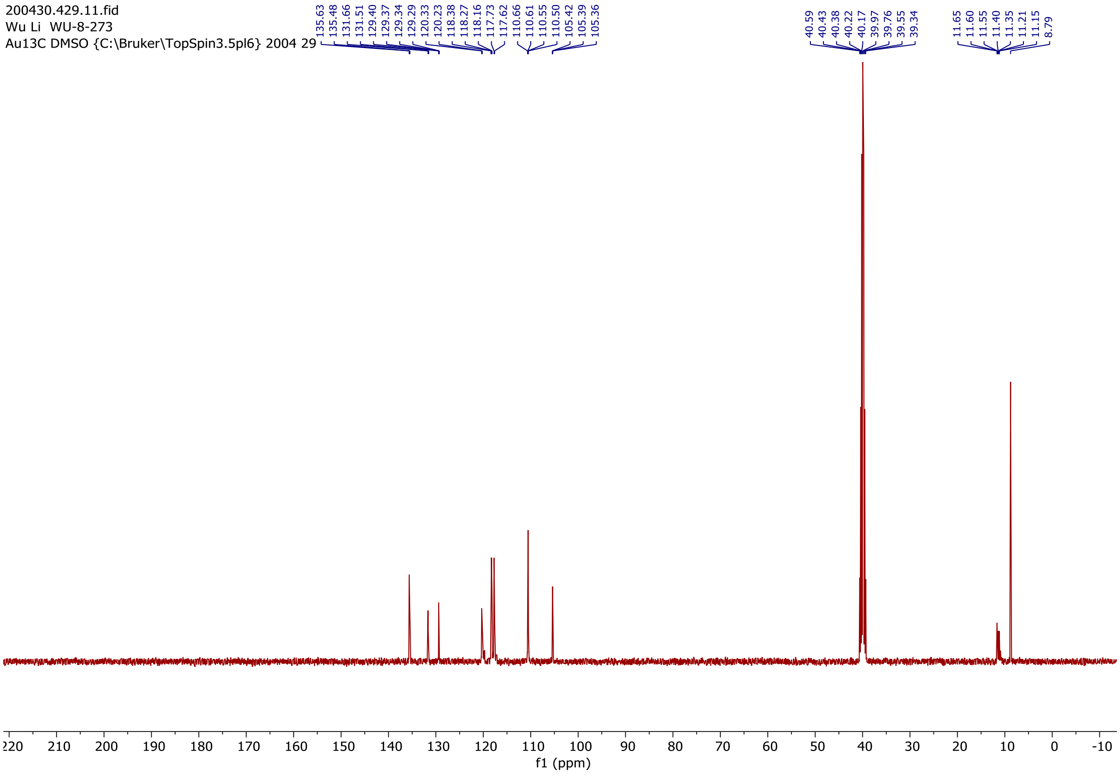

^{1}H NMR for 64a:

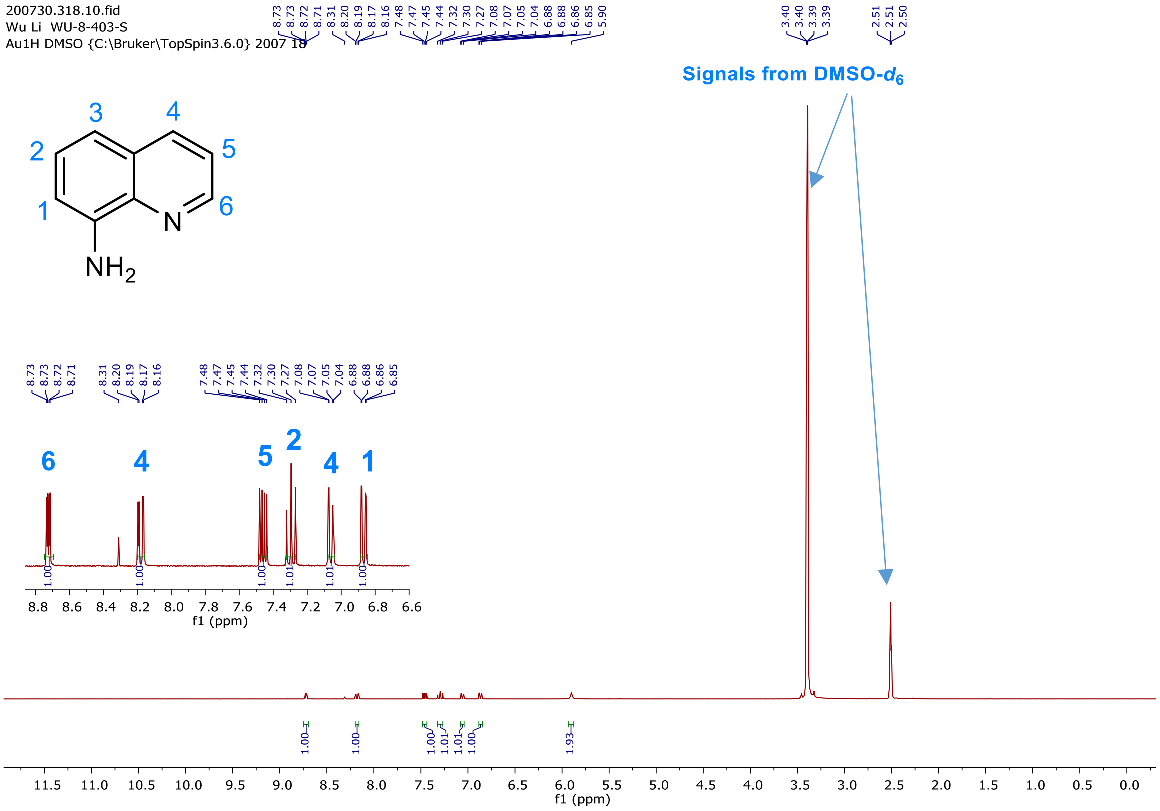

Original spectra for 64b:

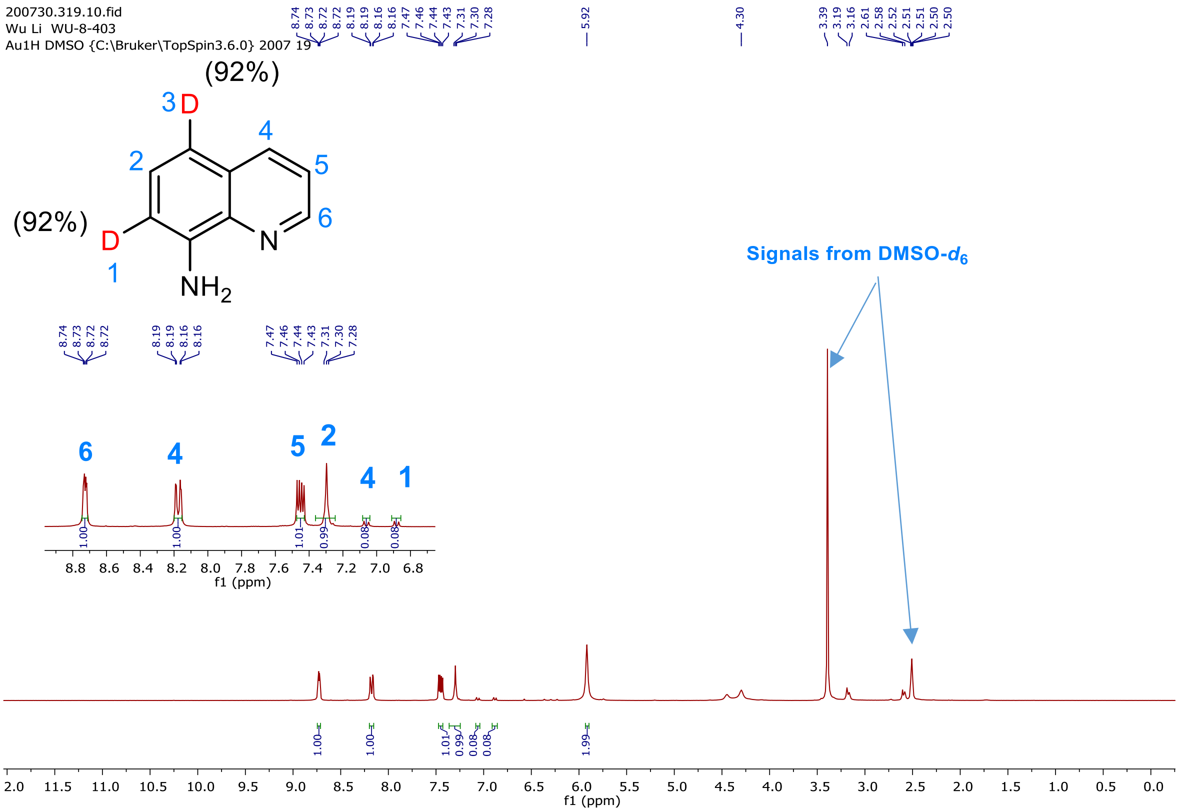

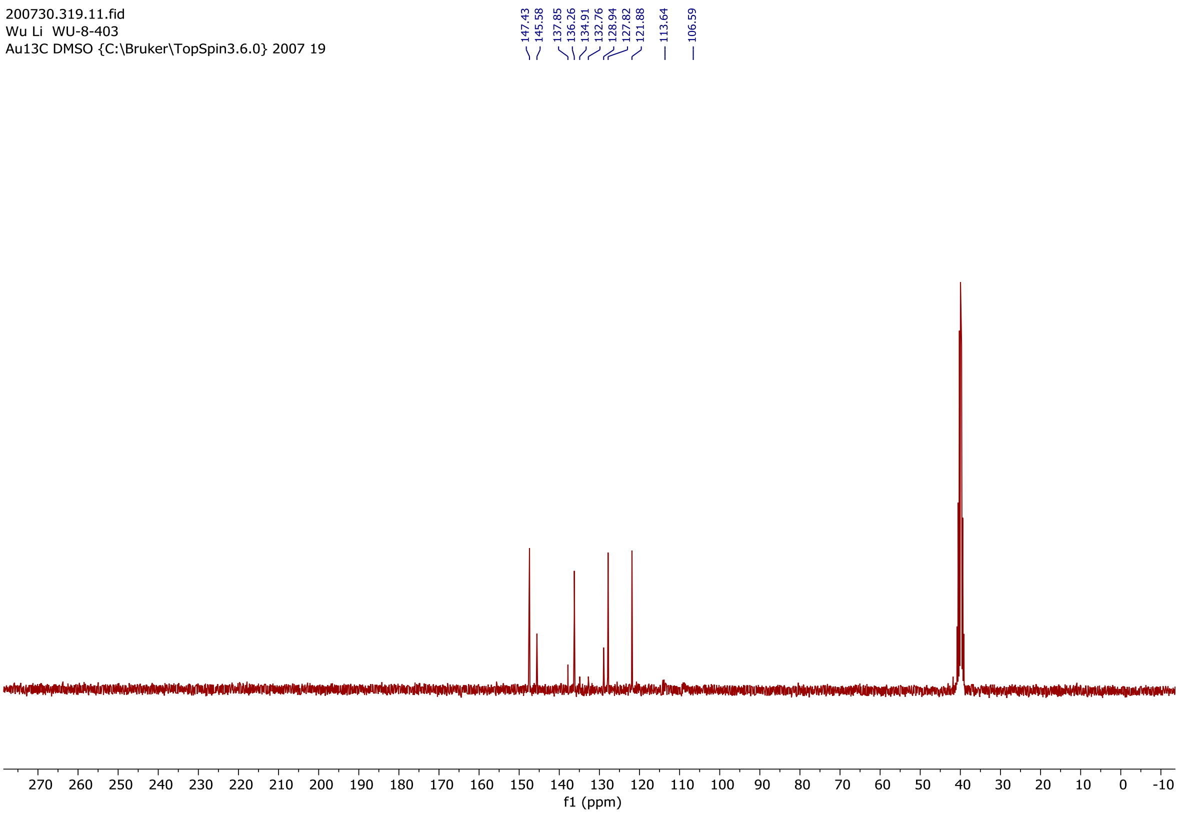

^{1}H NMR for 65a:

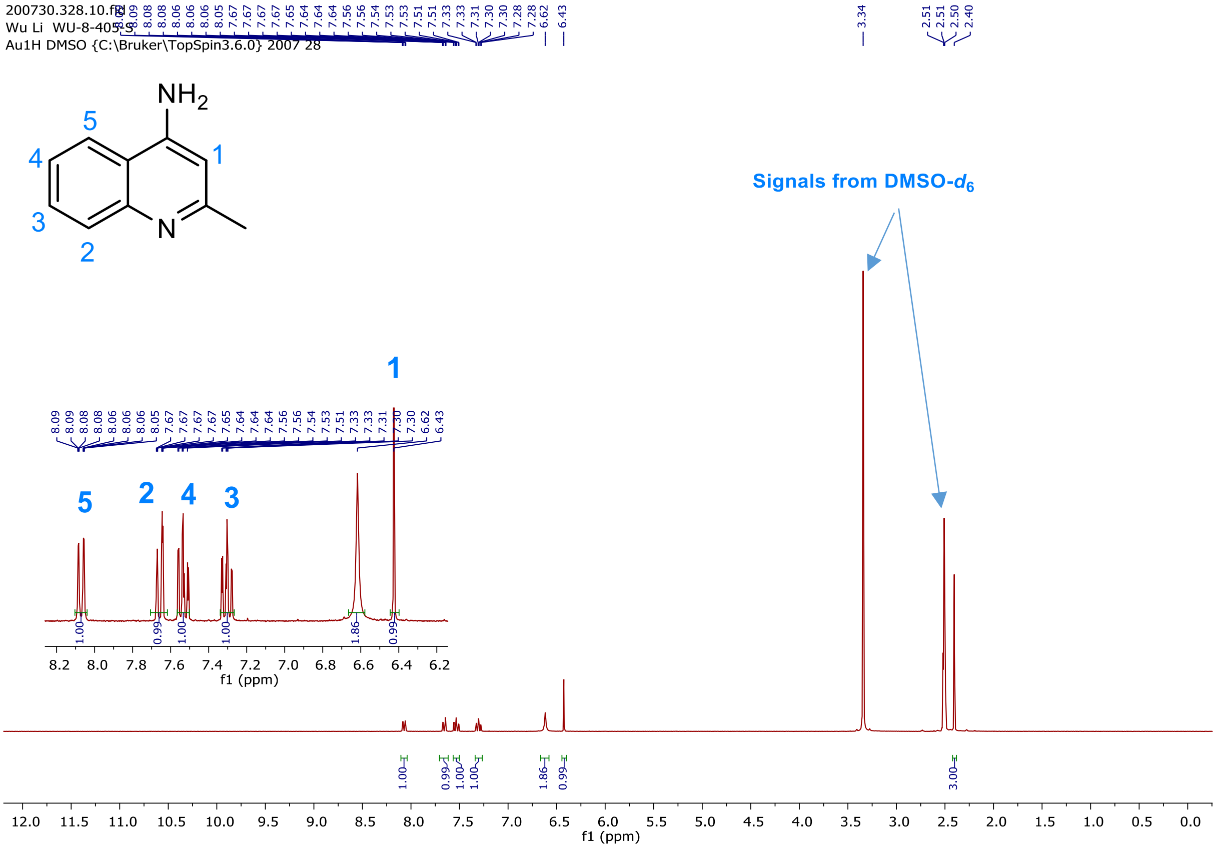

Original spectra for 65b:

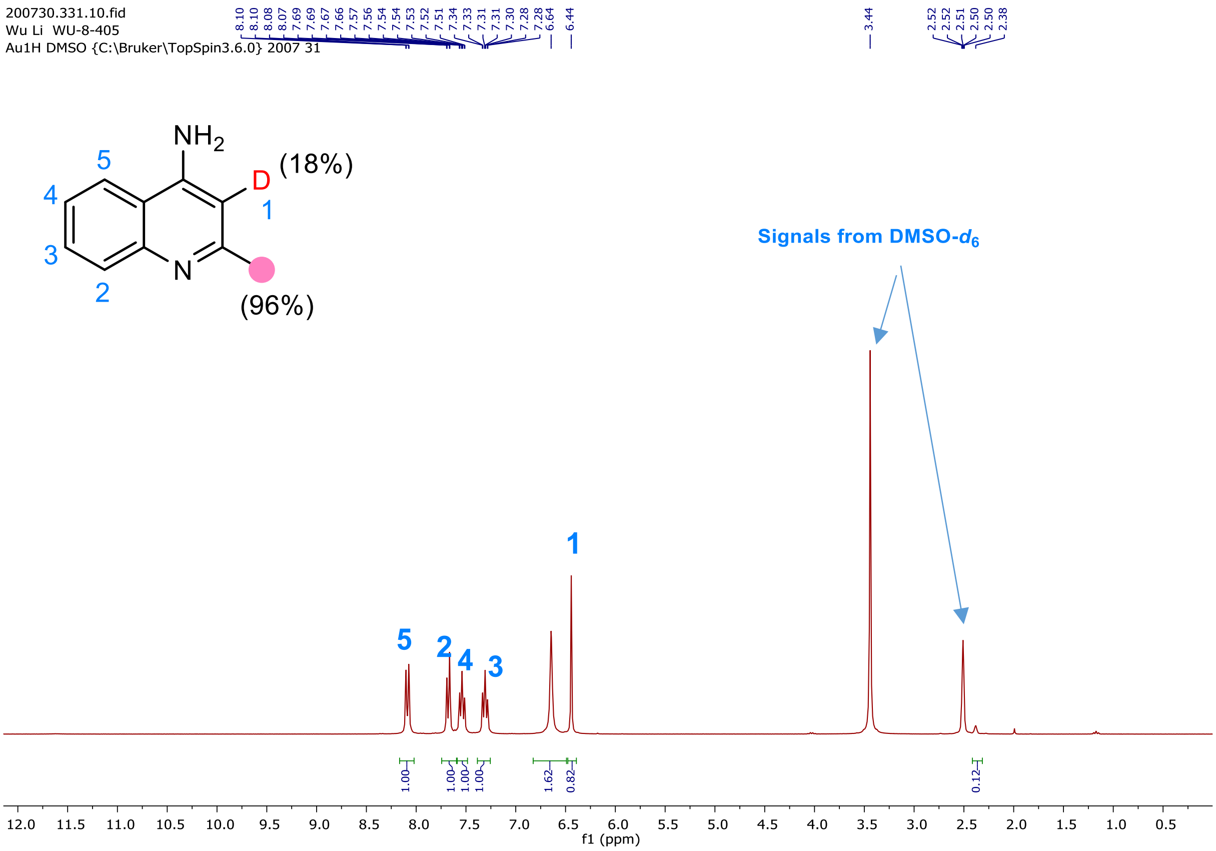

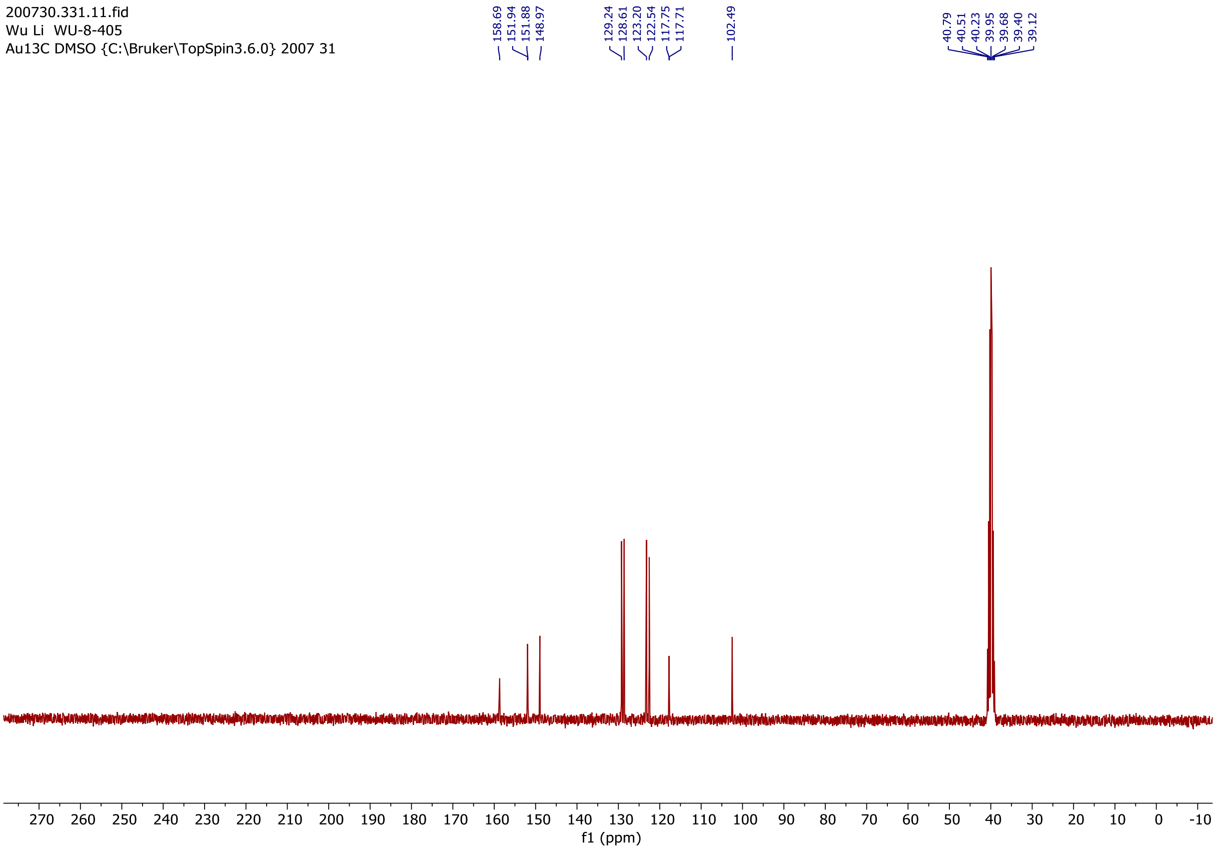

^{1}H NMR for 66a:

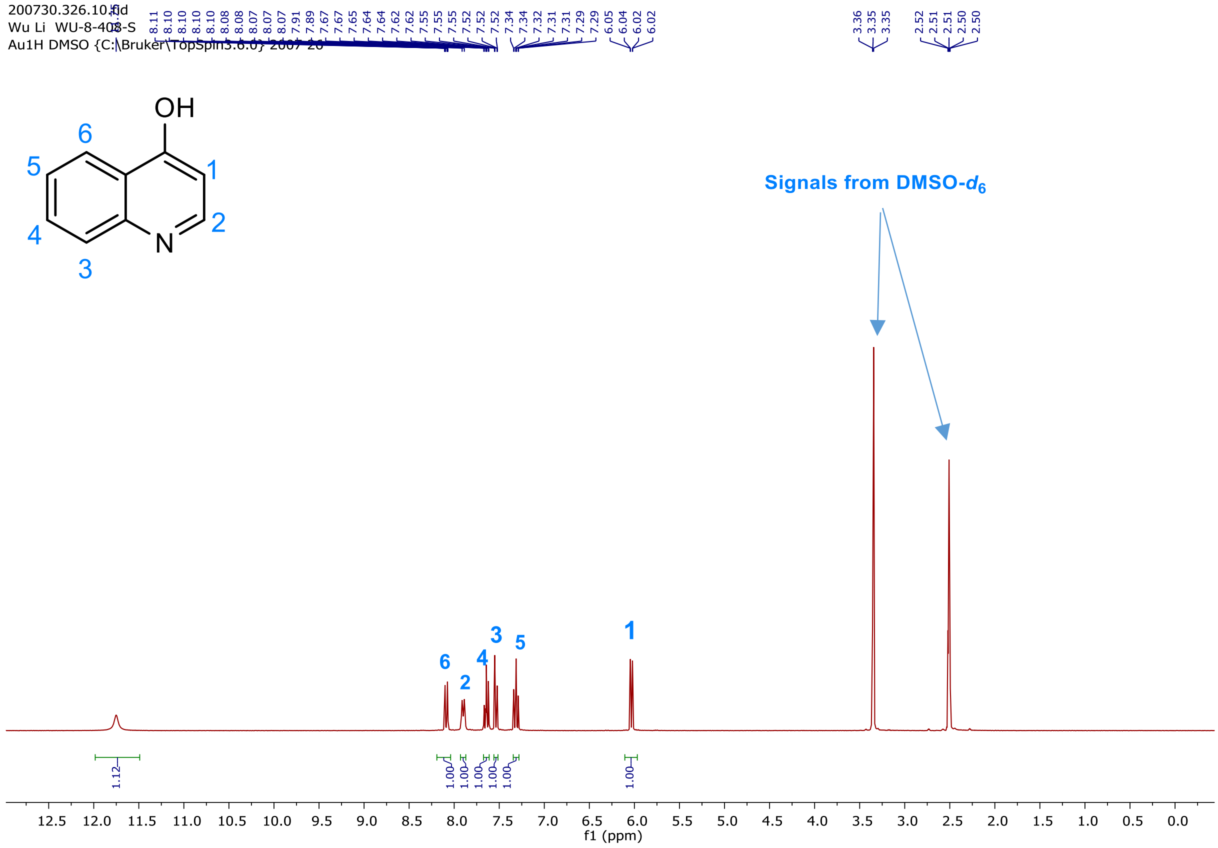

Original spectra for 66b:

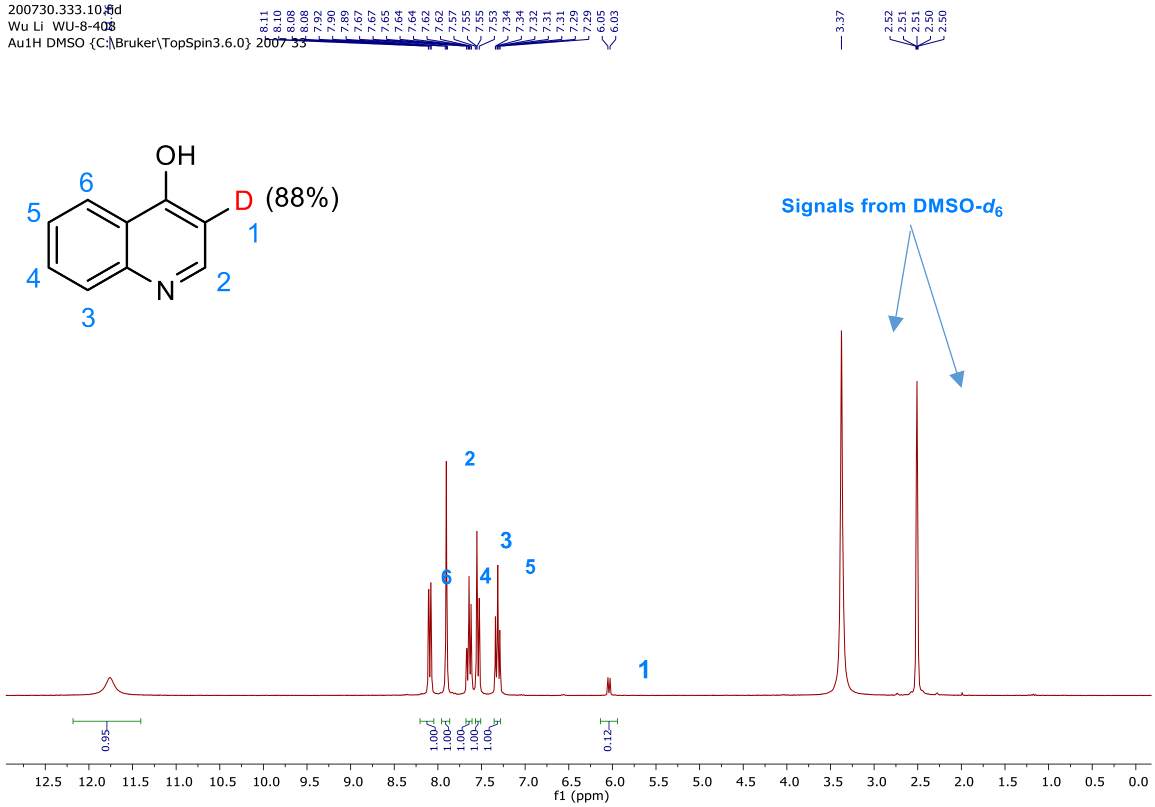

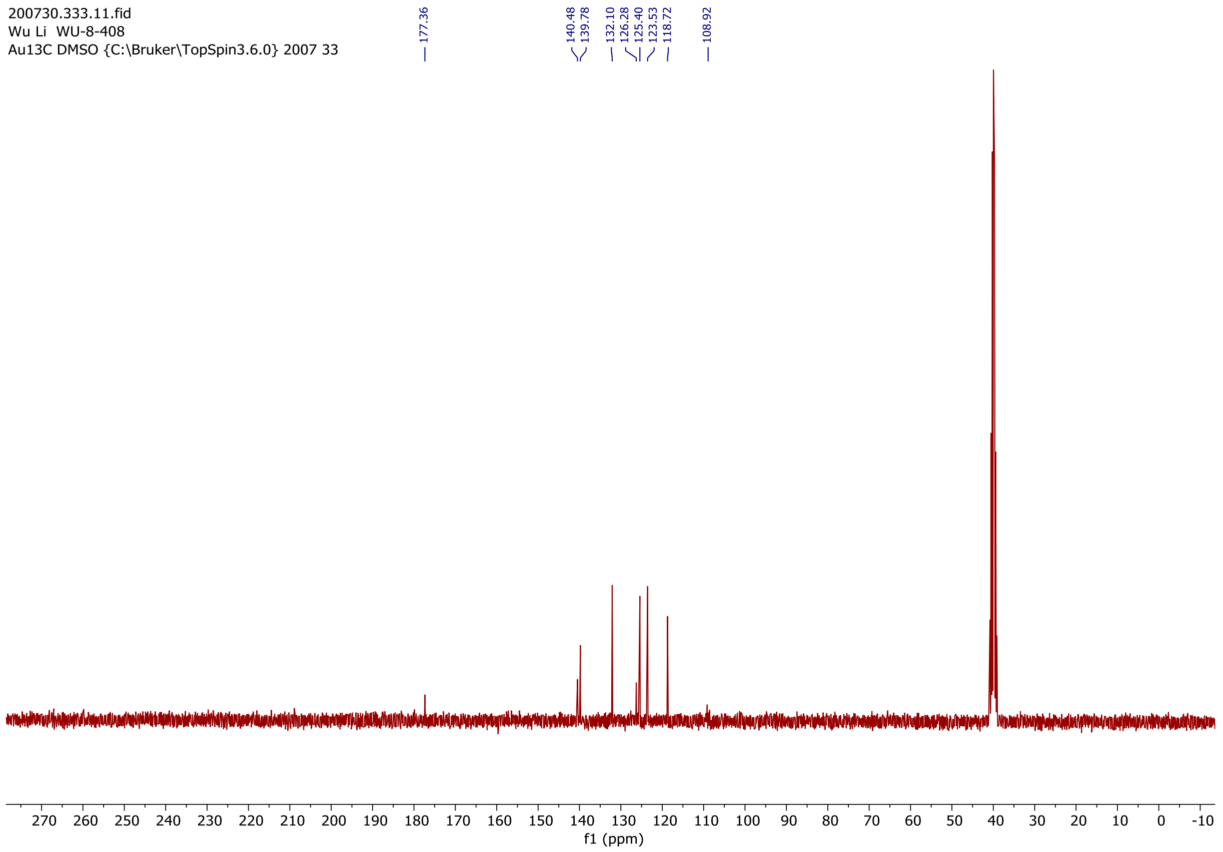

^{1}H NMR for 67a:

In $CDCl_3$:

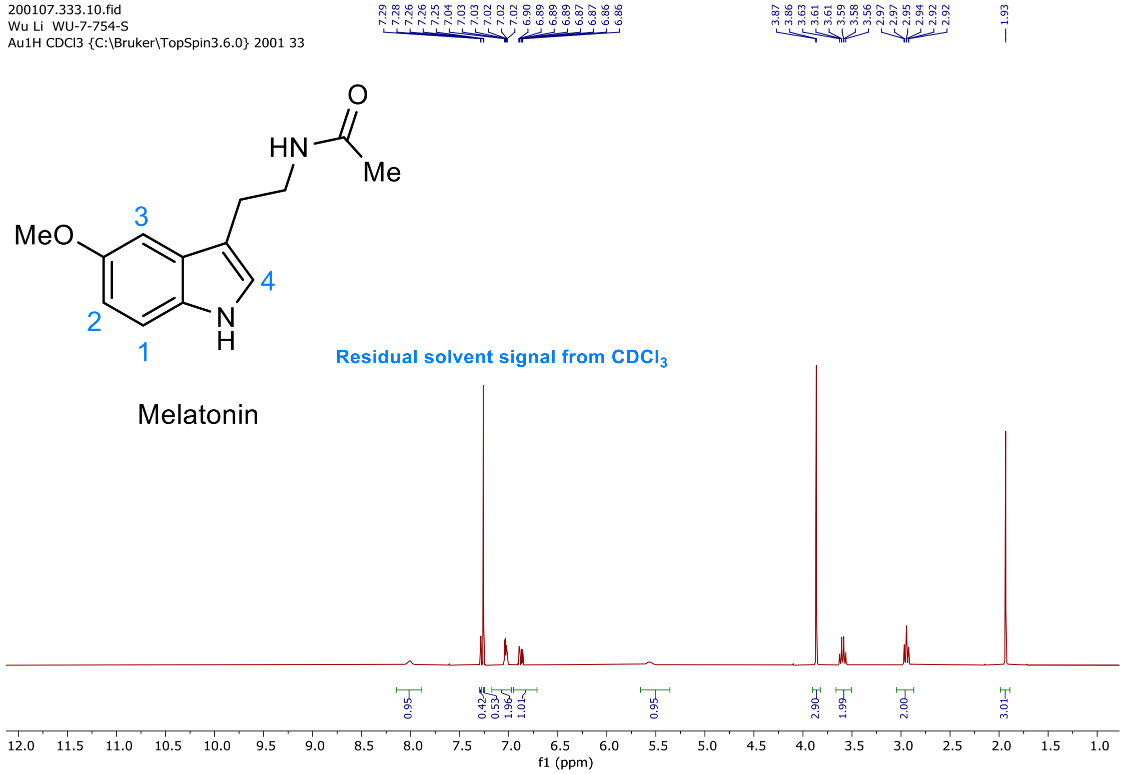

In DMSO-d_6:

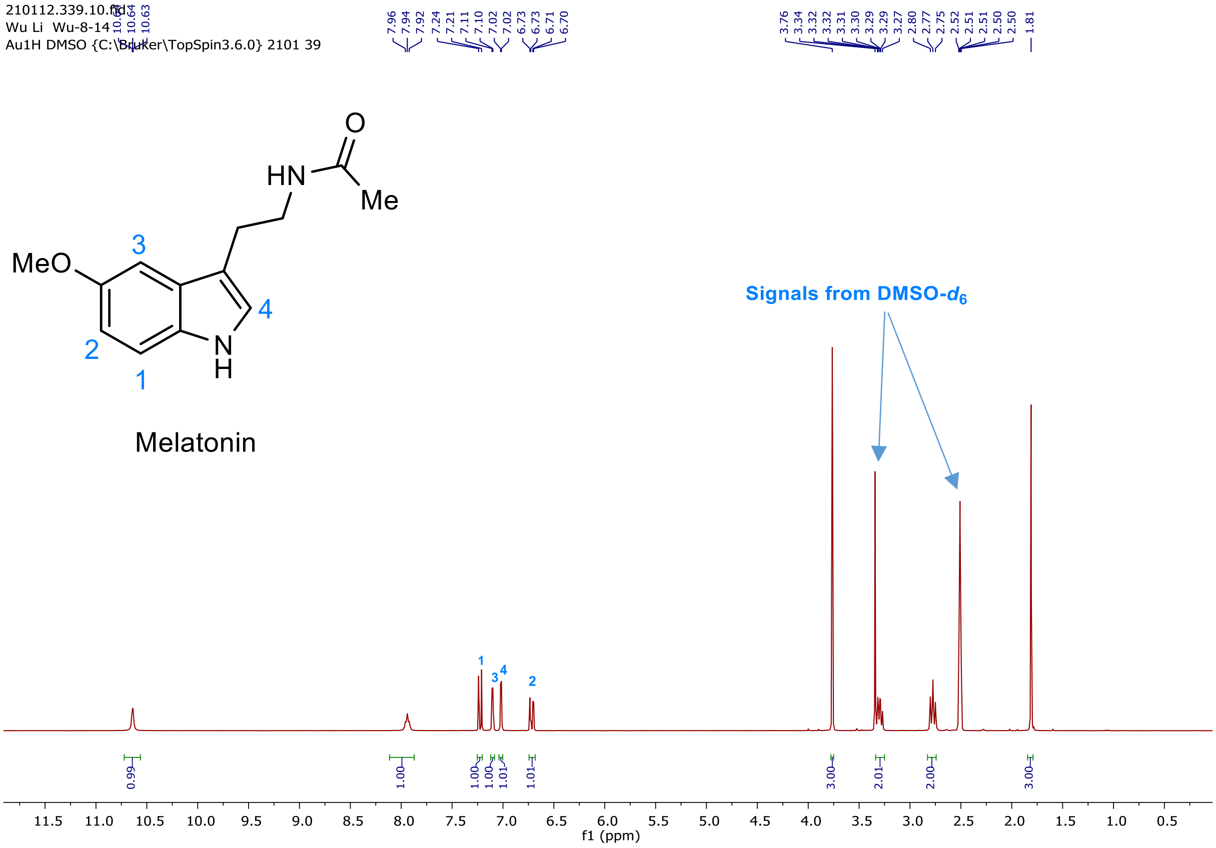

Original spectra for 67b:

In $CDCl_3$:

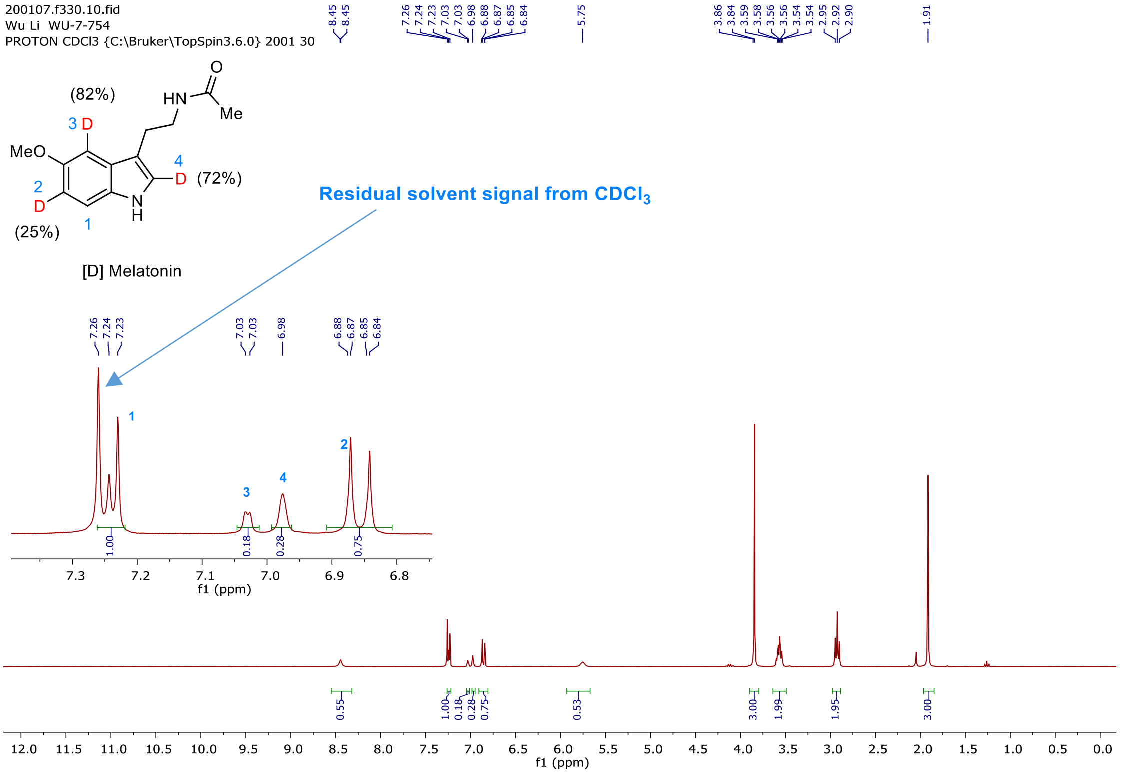

In DMSO-d_6:

210128.f321.10.fid
Florian Bourriquen FB-350-5
PROTON DMSO {C:\Bruker\TopSpin3.6.2} 2101 21

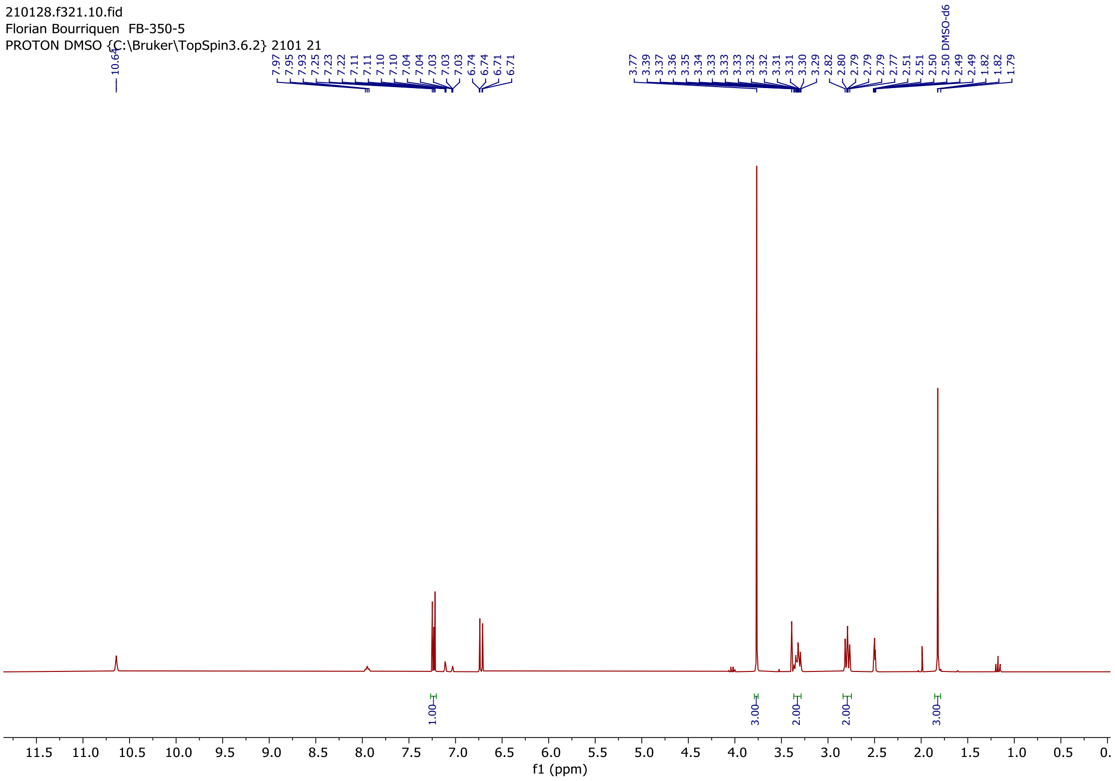

200107.f330.11.fid
Wu Li WU-7-754
C13CPD CDCl3 {C:\Bruker\TopSpin3.6.0} 2001 30

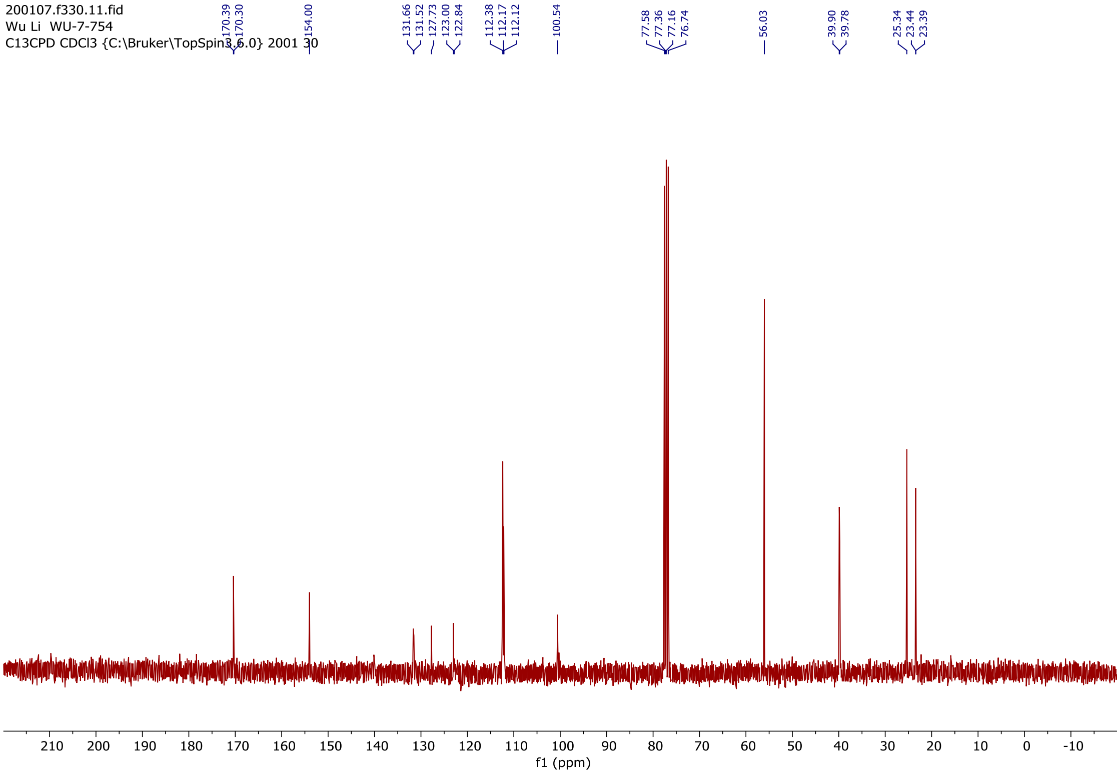

^{1}H NMR for 68a:

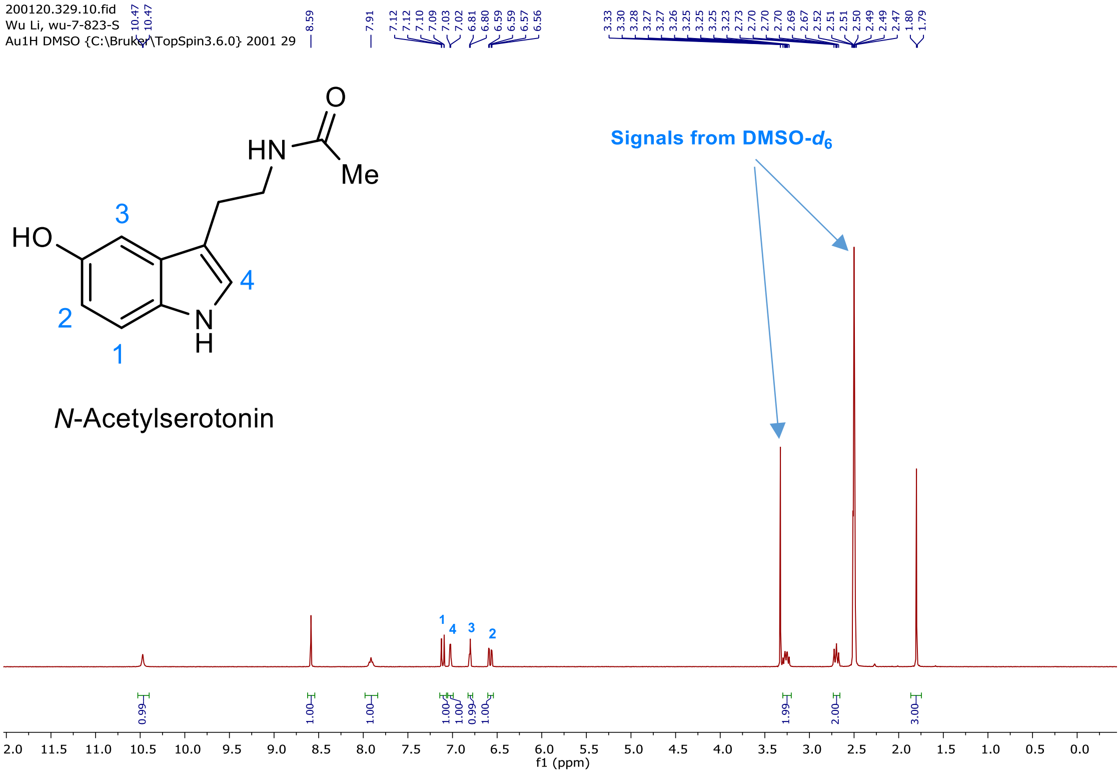

Original spectra for 68b:

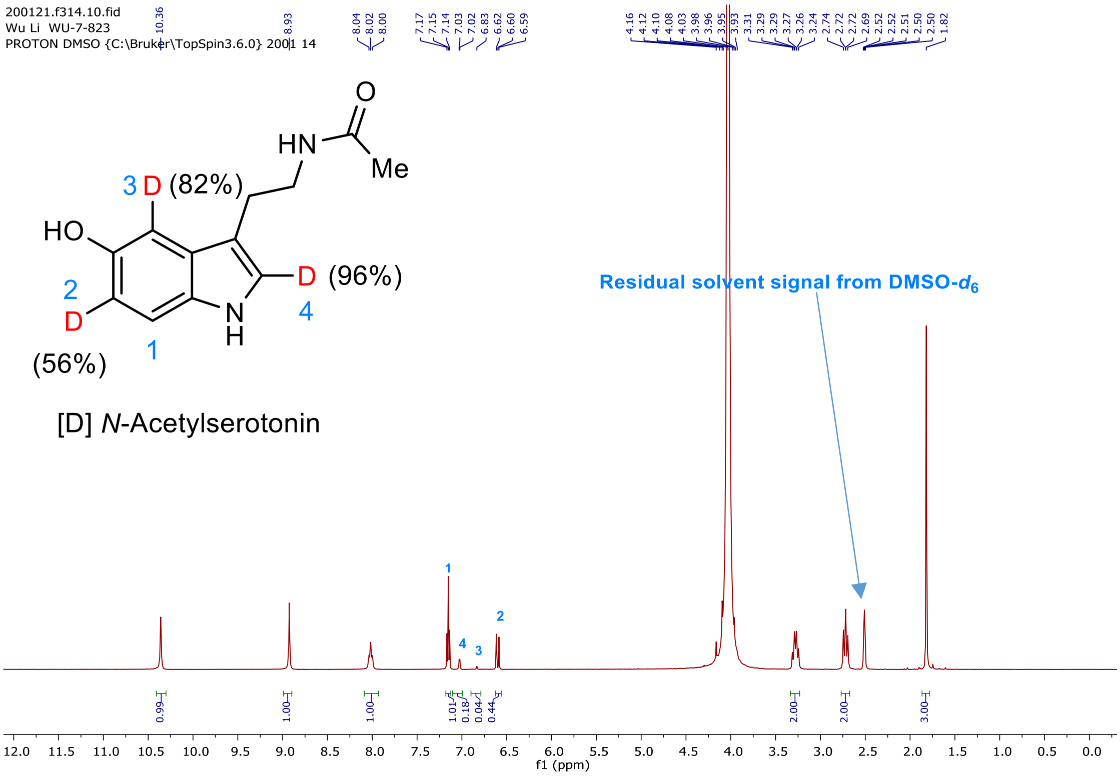

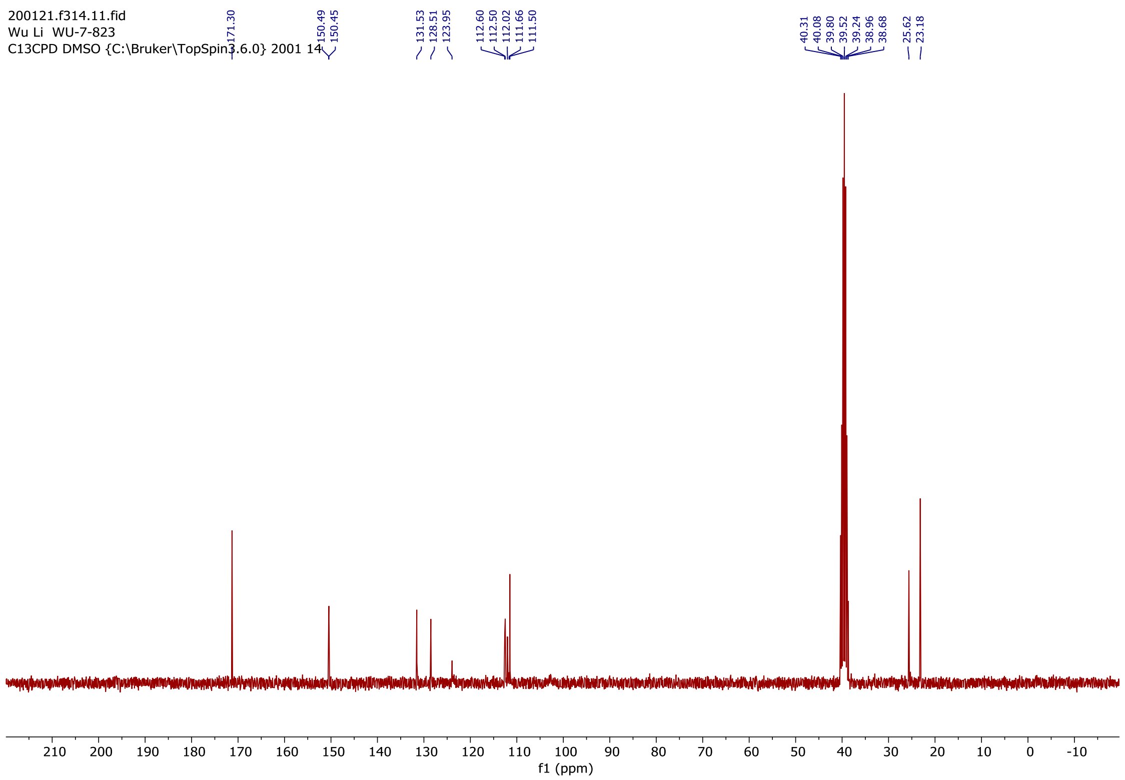

^{1}H NMR for 69a:

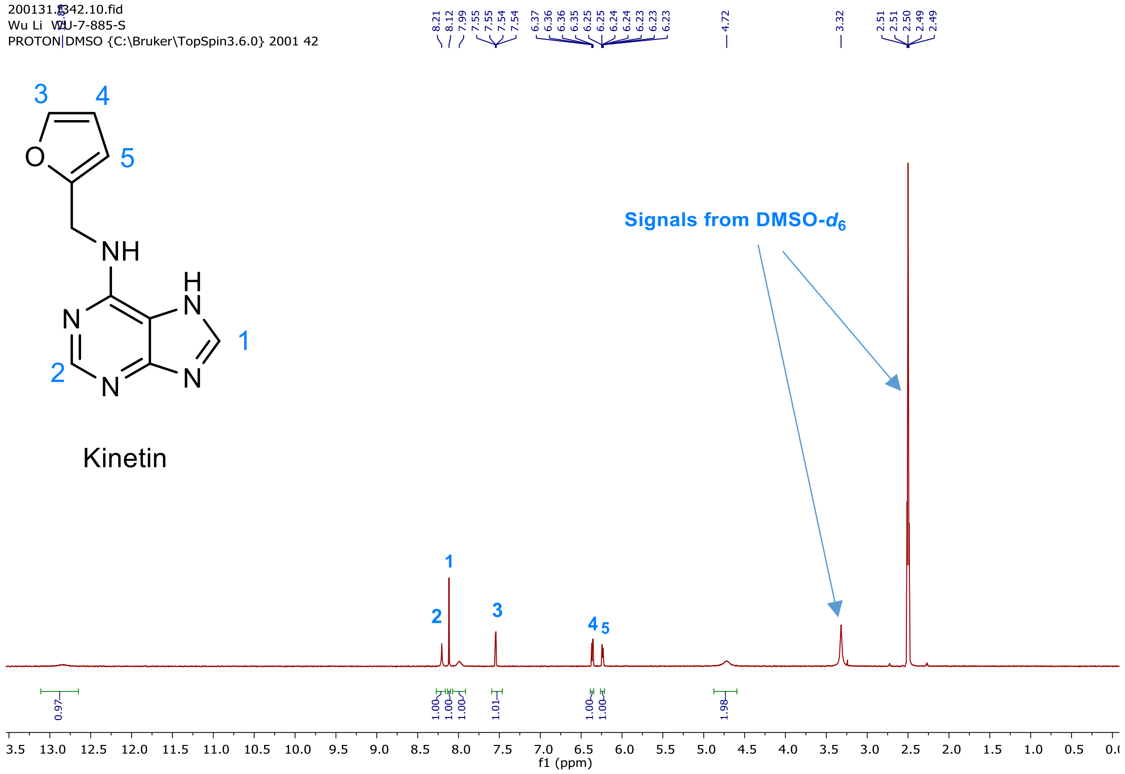

Original spectra for 69b:

200204.312.10.fid
Wu Li, wu-7-885
Au1H DMSO {C:\Bruker\TopSpin3.6.0} 2002 12

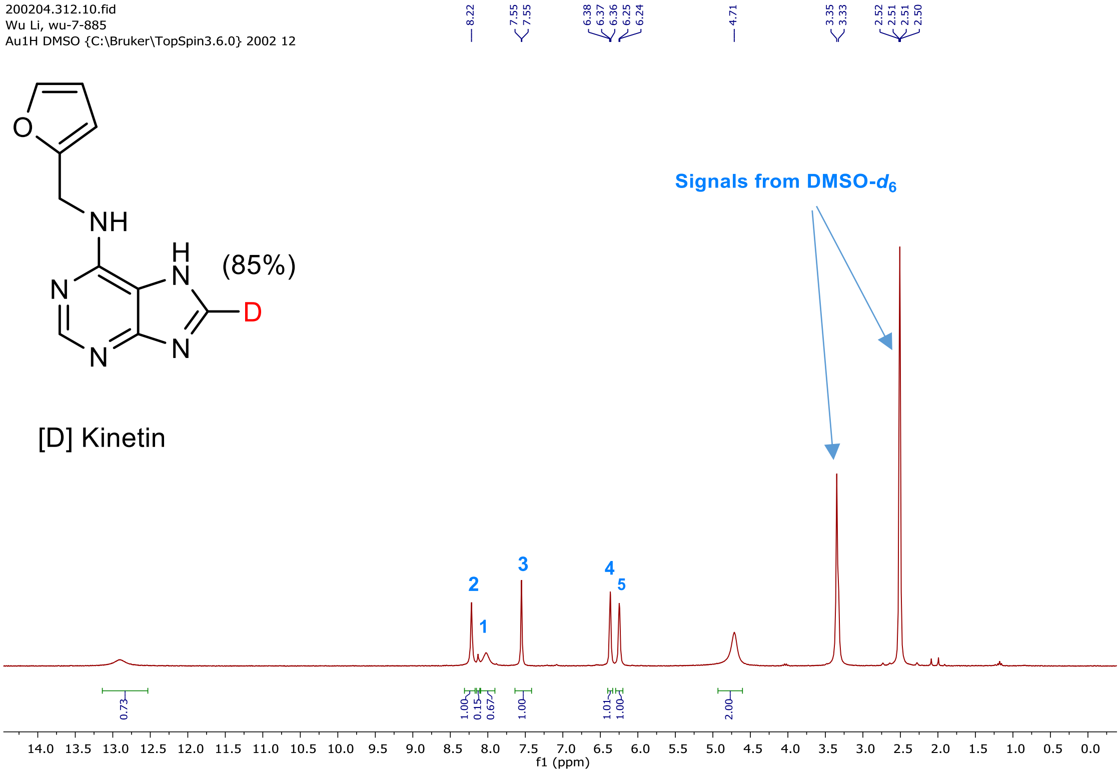

200430.421.10.fid
Wu Li WU-8-275
Au13C DMSO {C:\Bruker\TopSpin3.5pl6} 2004 21

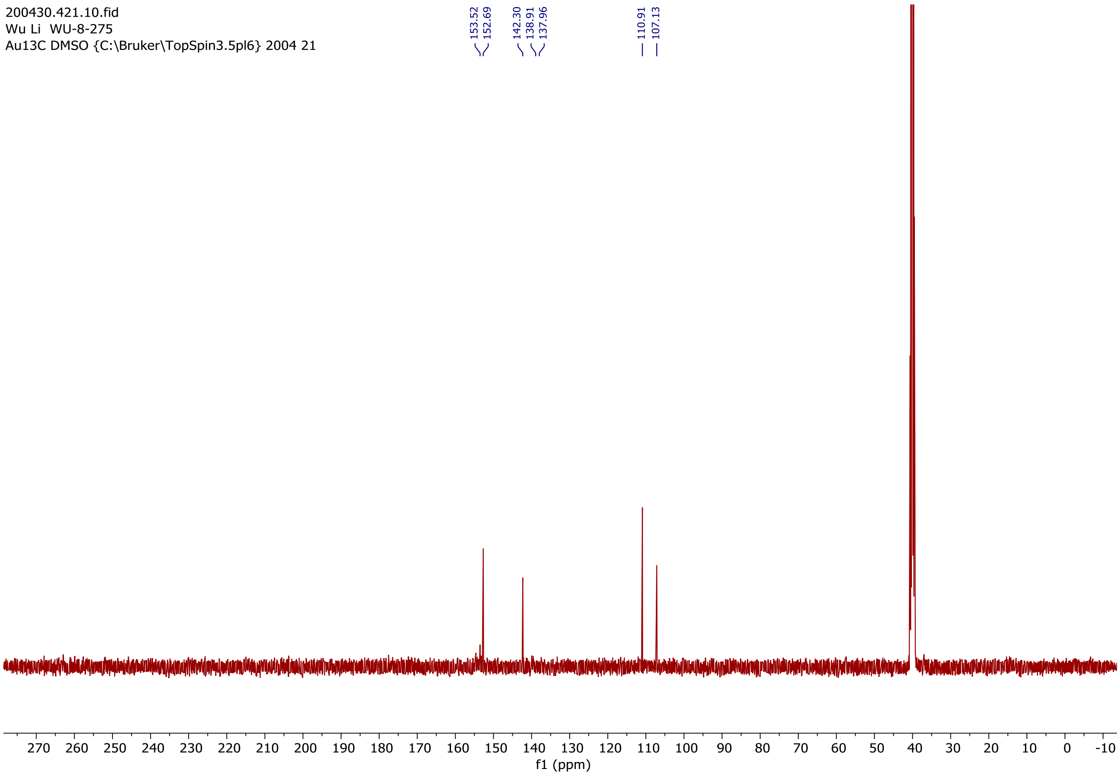

^{1}H NMR for 70a:

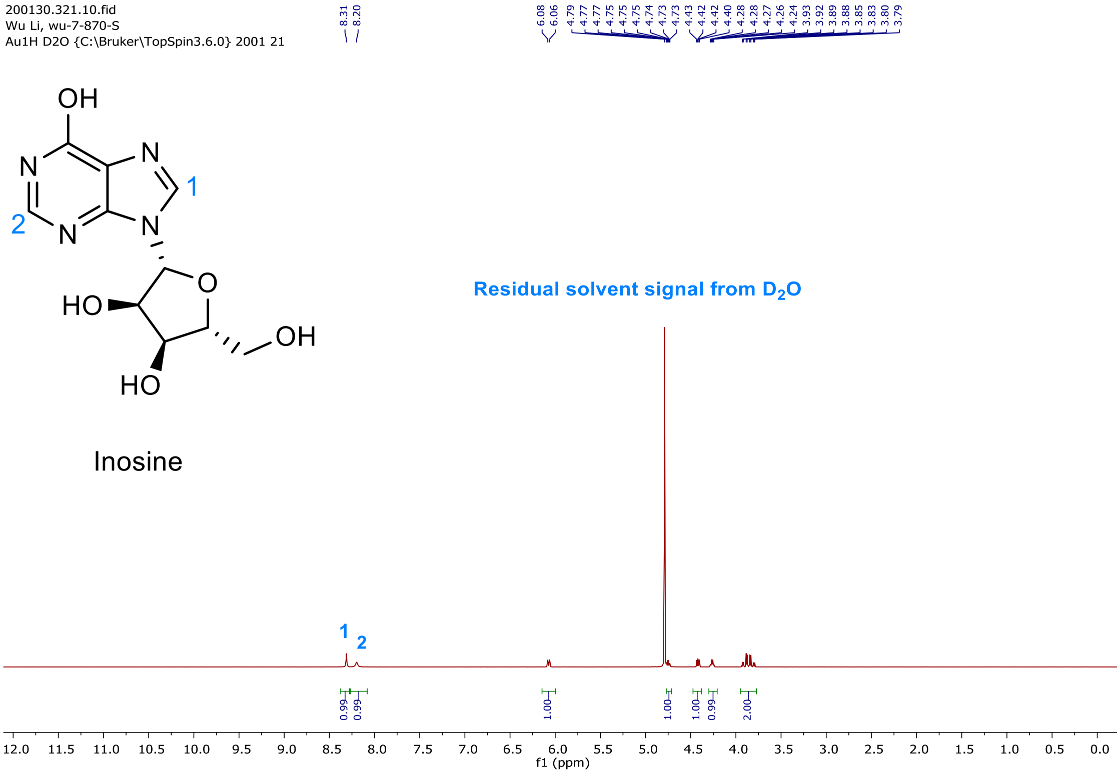

Original spectra for 70b:

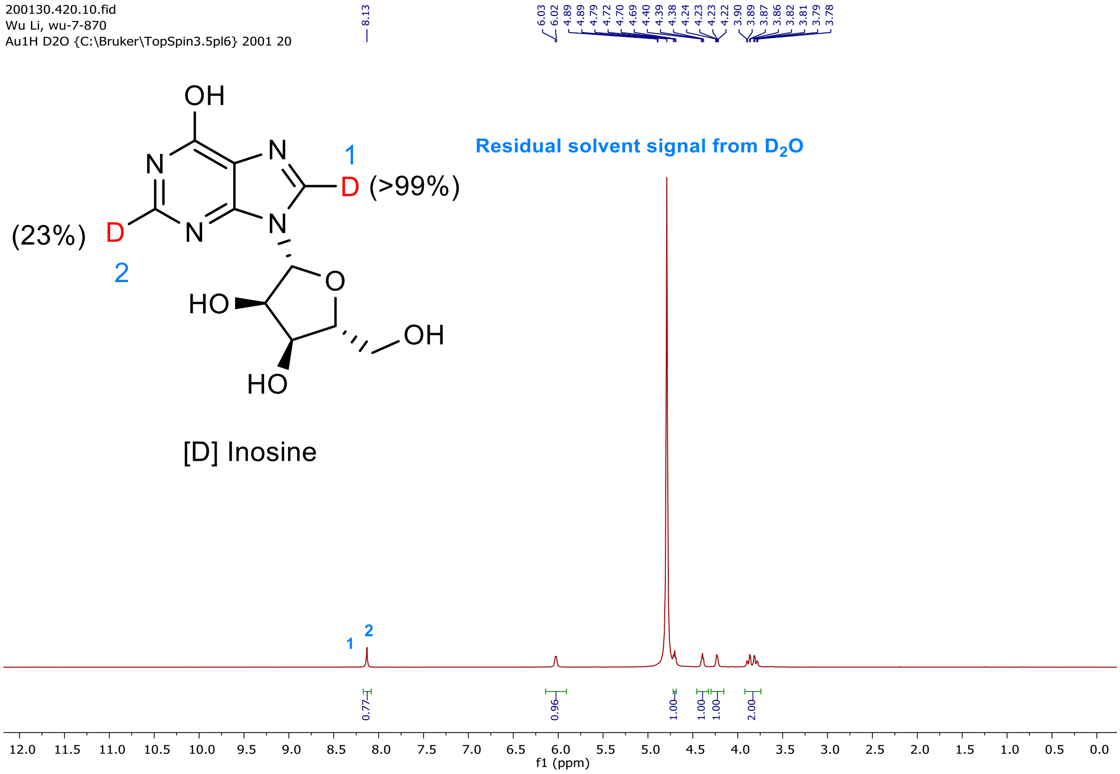

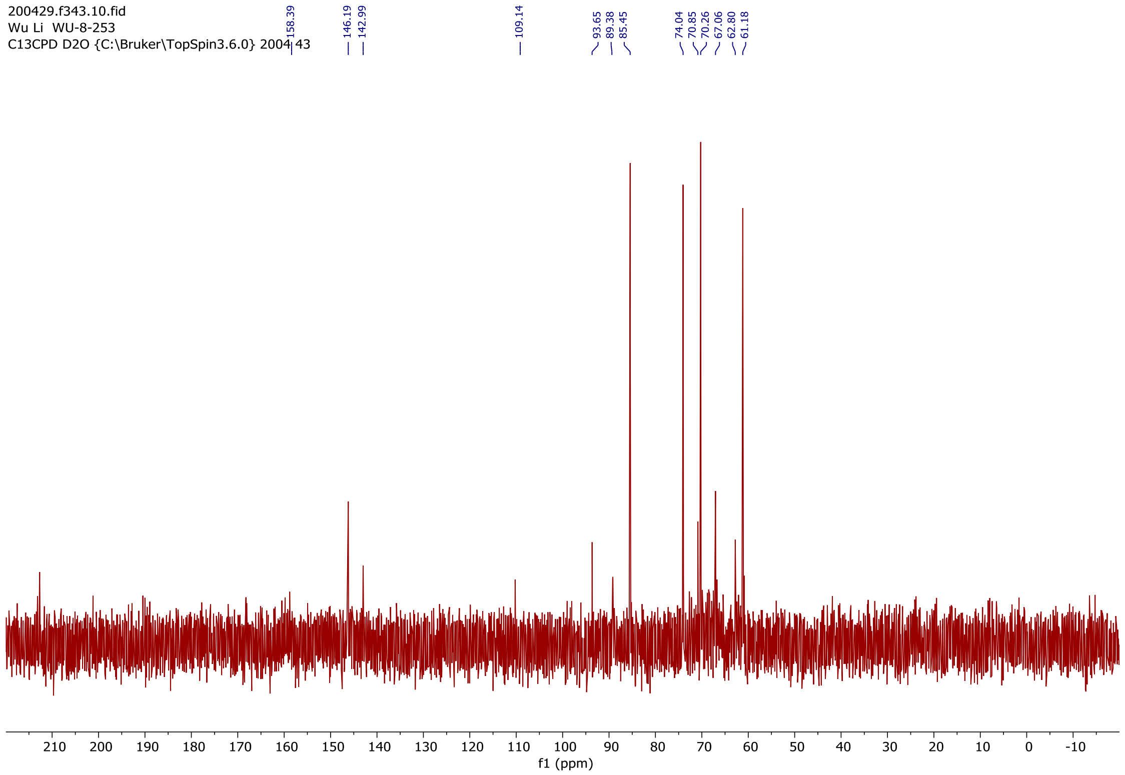

^{1}H NMR for 71a:

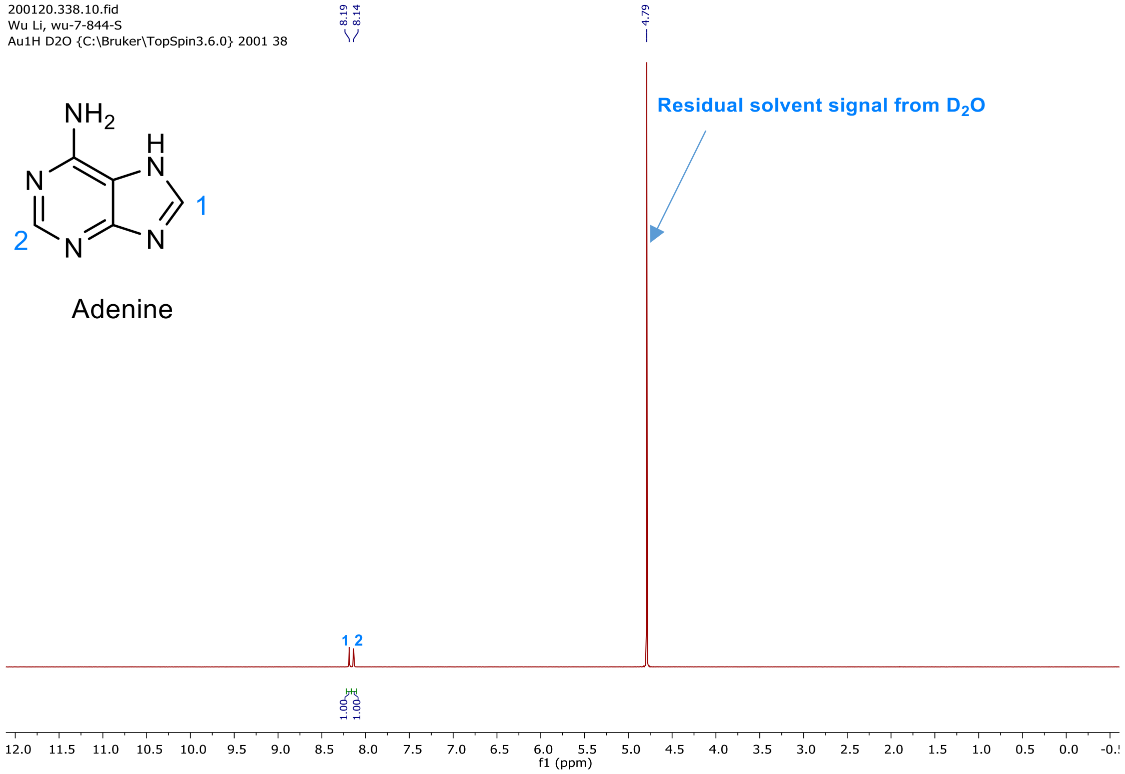

Original spectra for 71b:

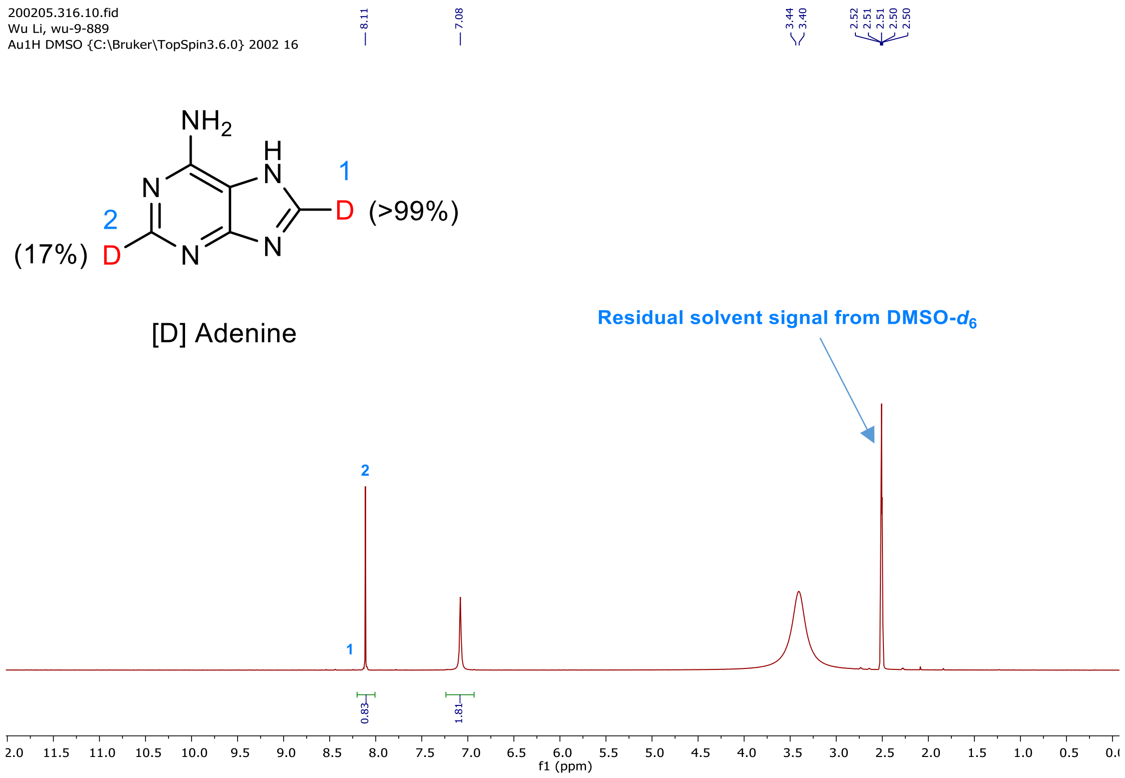

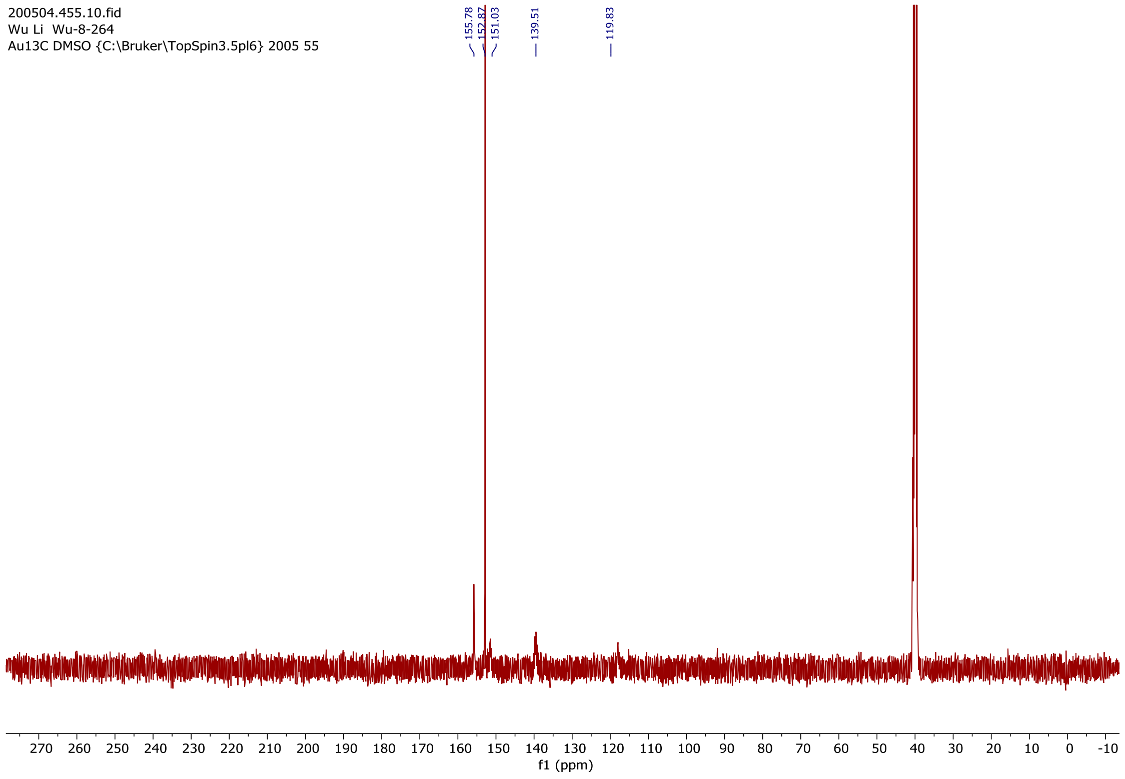

^{1}H NMR for 72a:

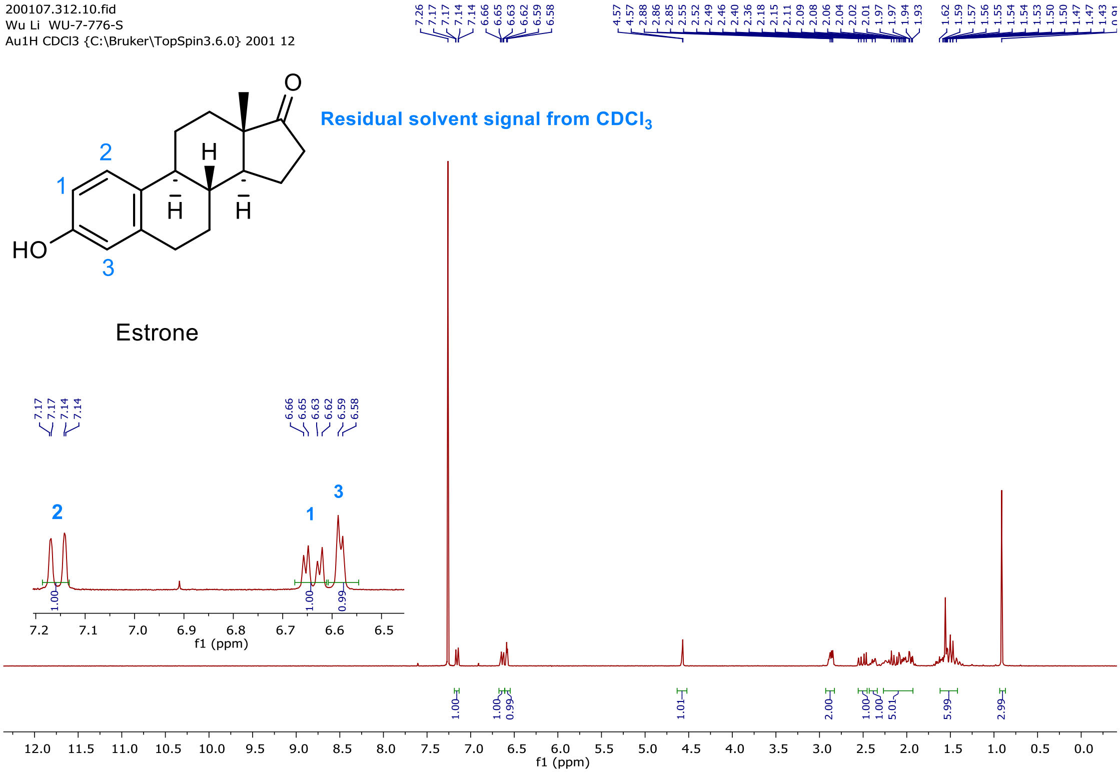

Original spectra for 72b:

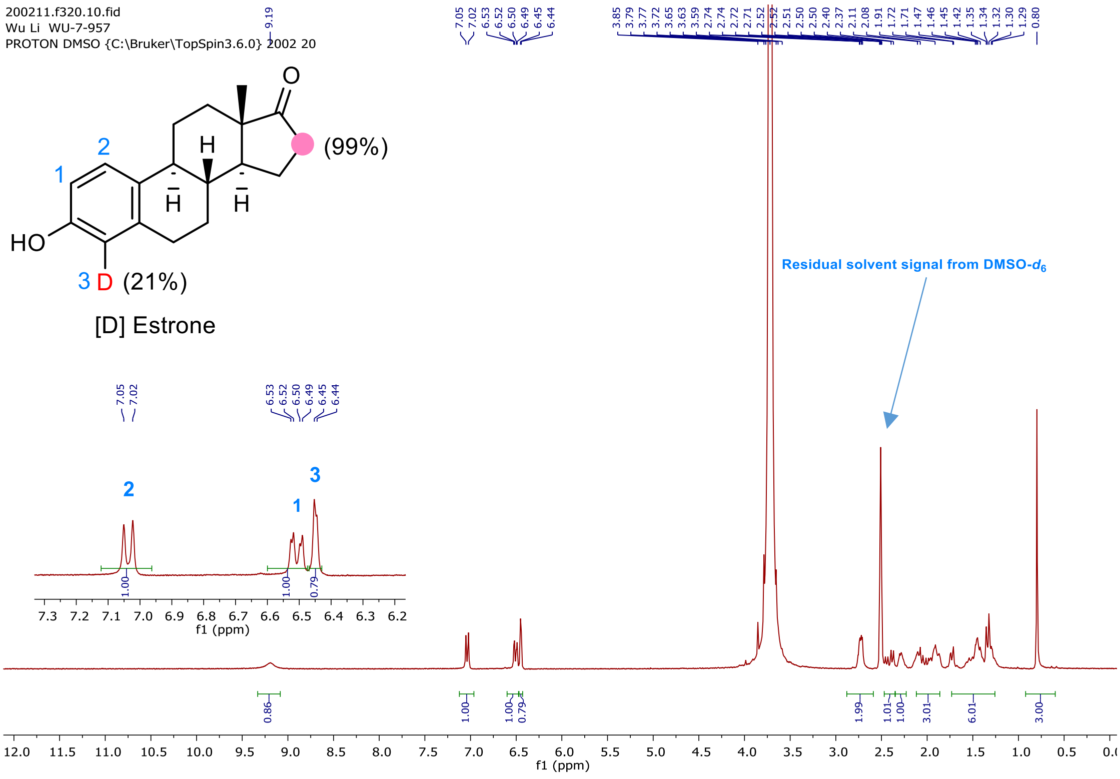

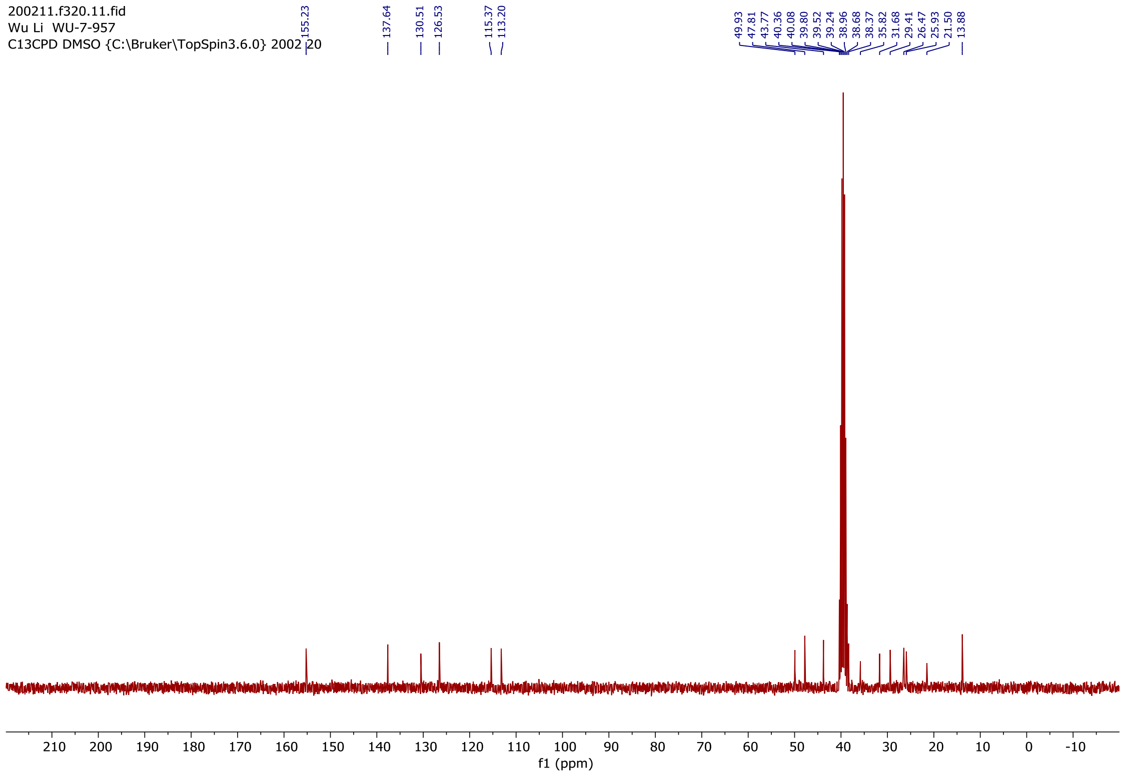

^{1}H NMR for 73a:

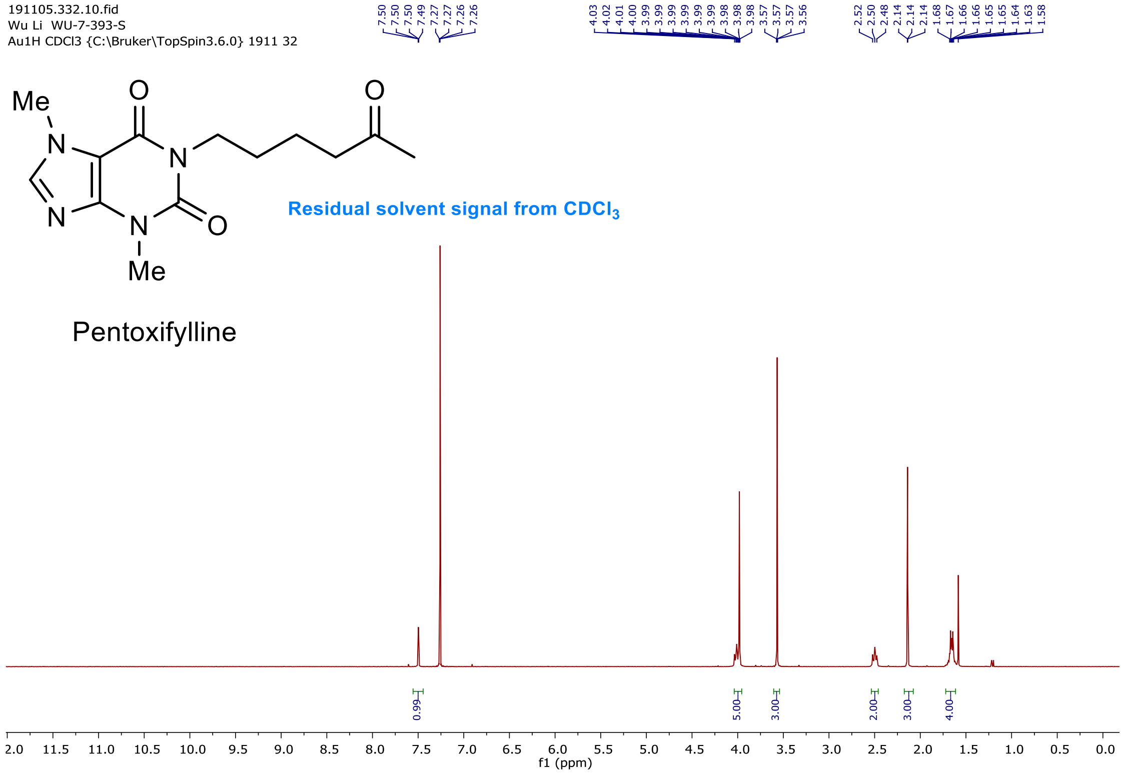

Pentoxifylline

Original spectra for 73b:

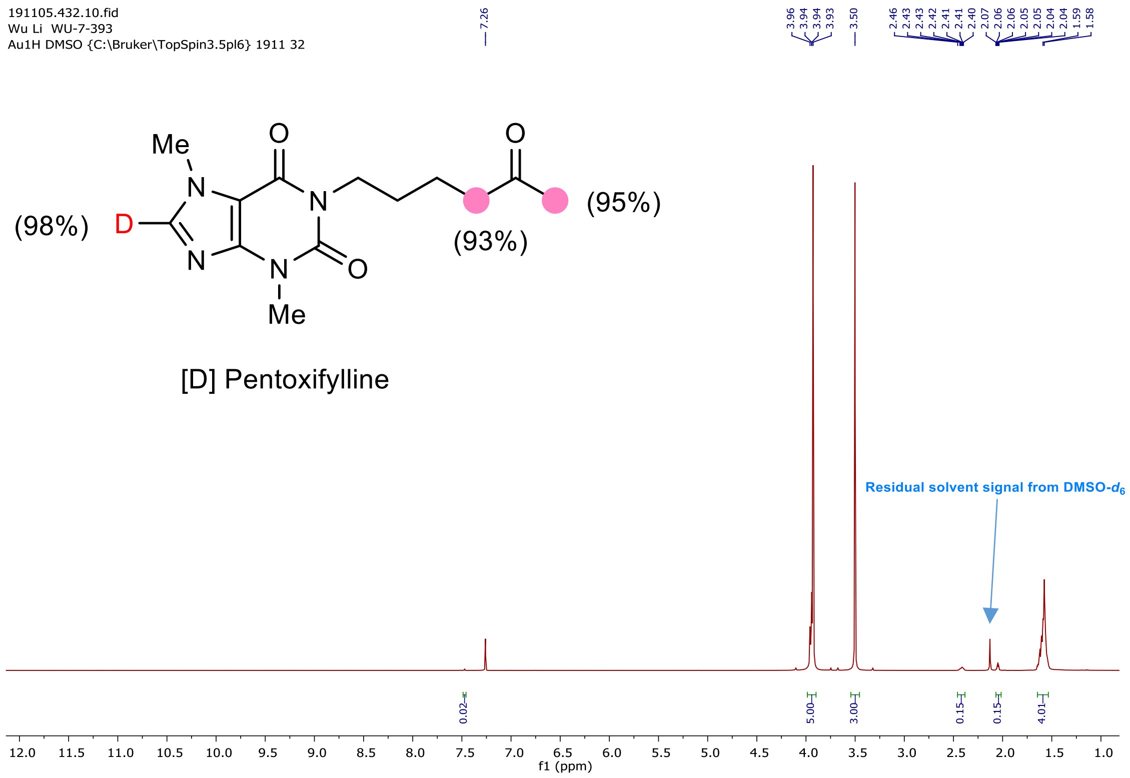

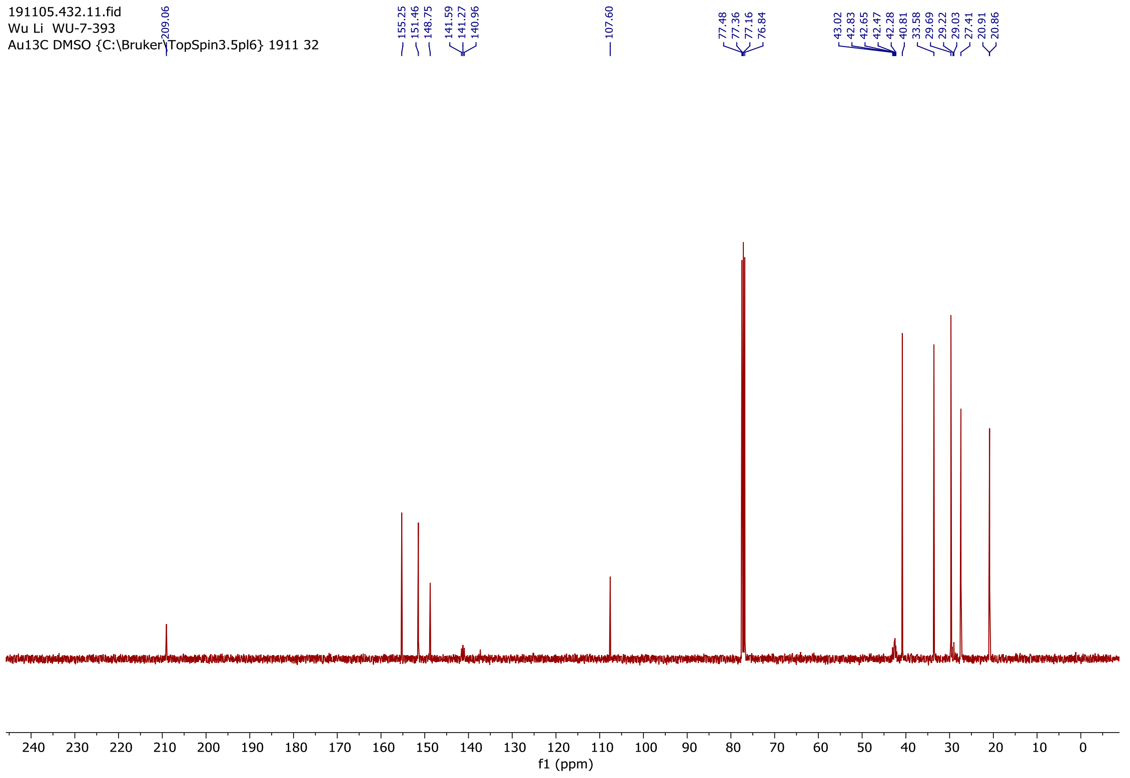

¹H NMR for 74a:

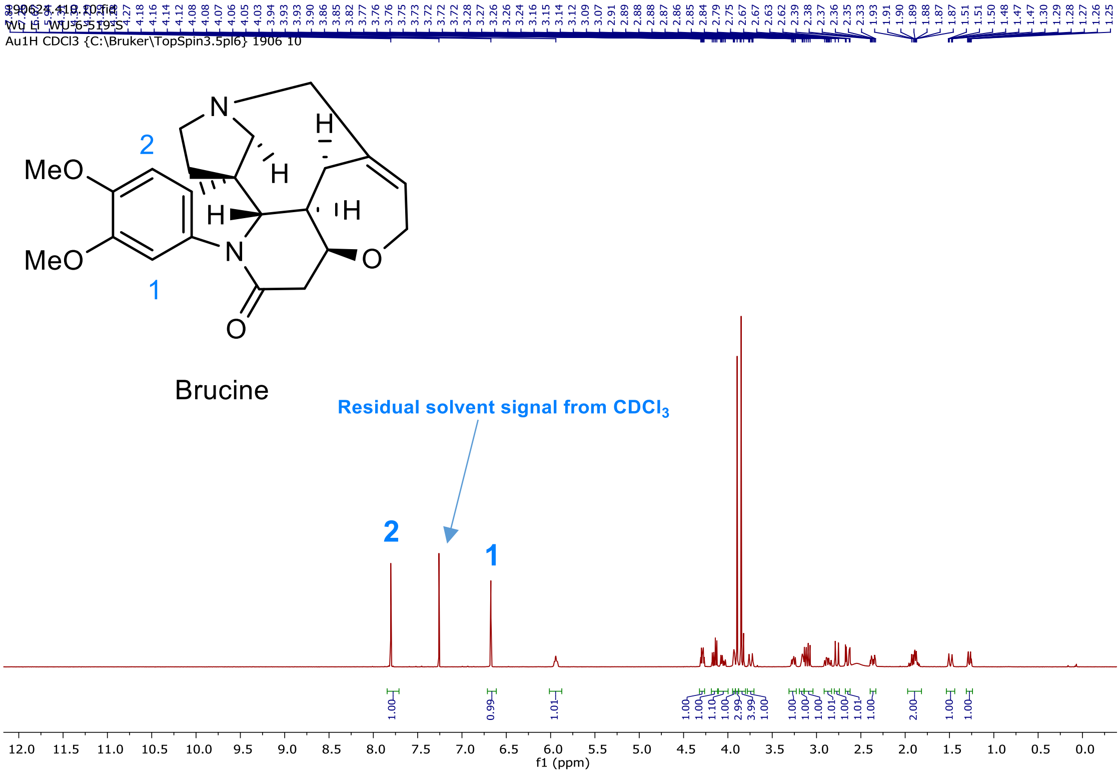

Original spectra for 74b:

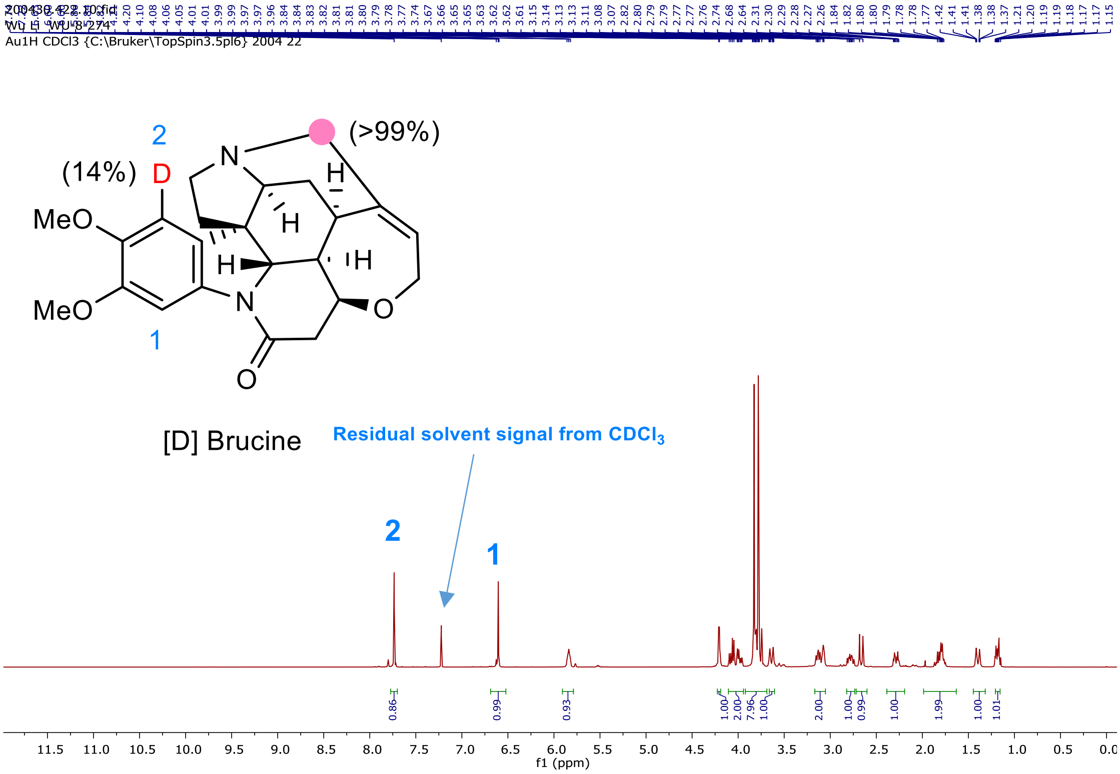

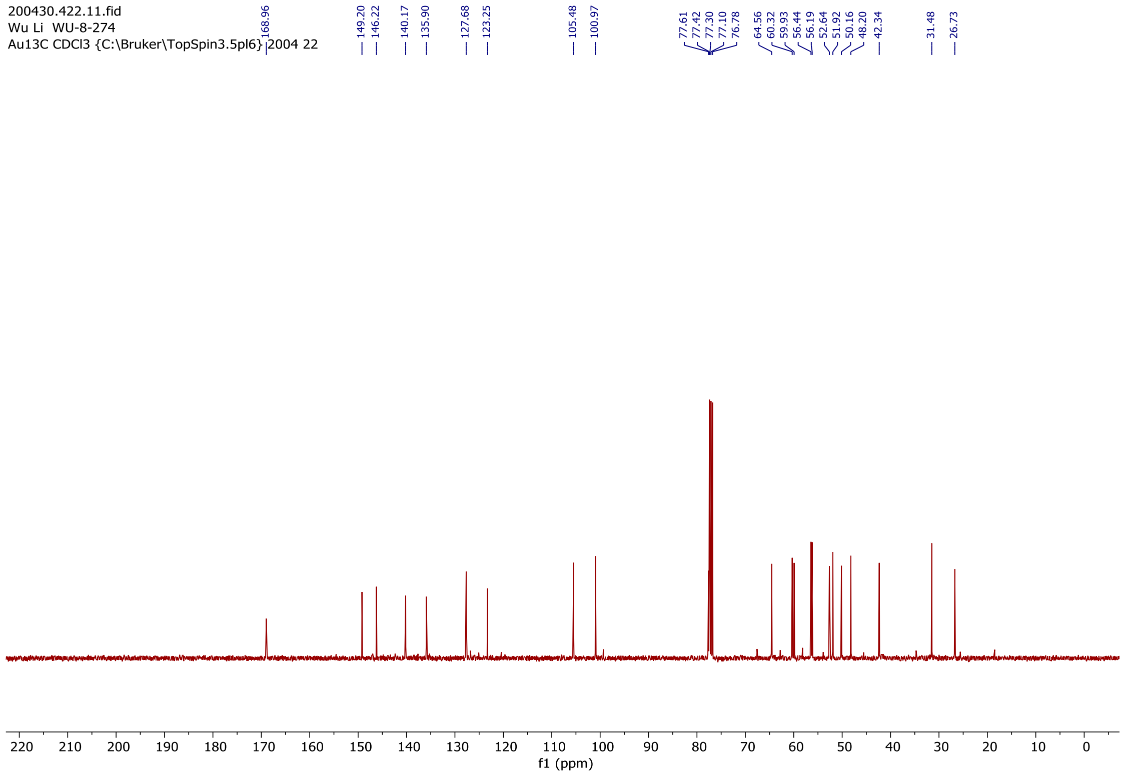

1H NMR for 75a:

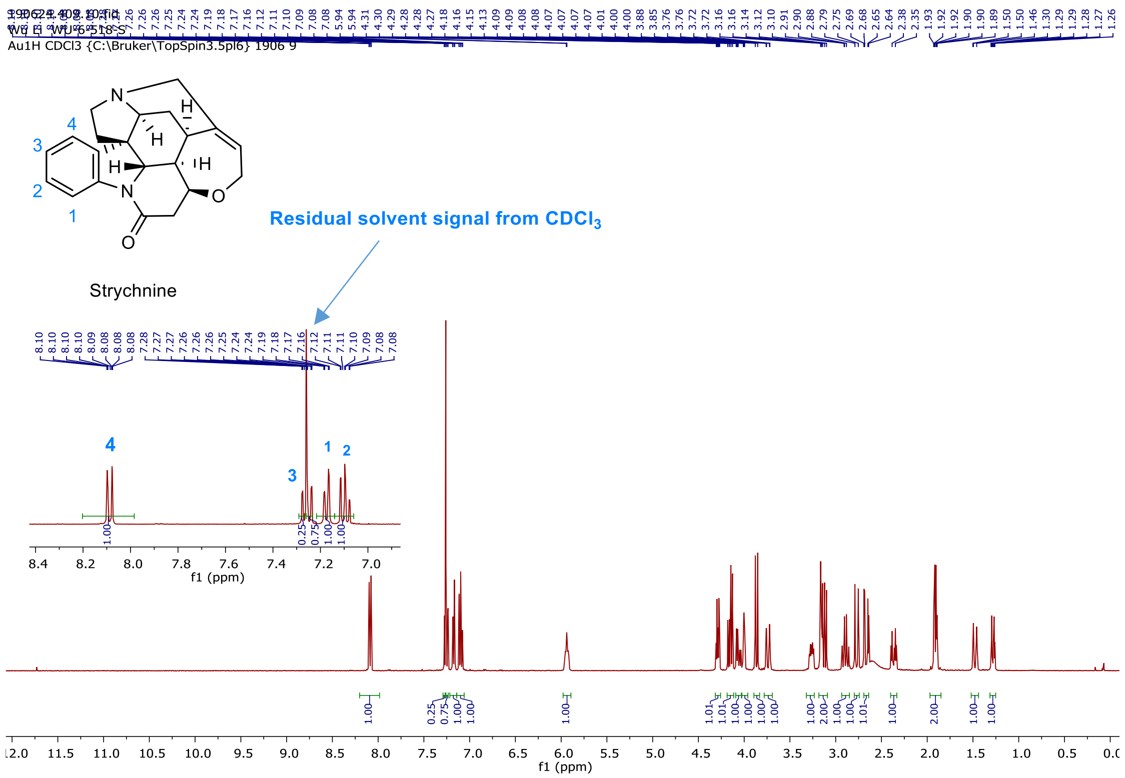

Original spectra for 75b:

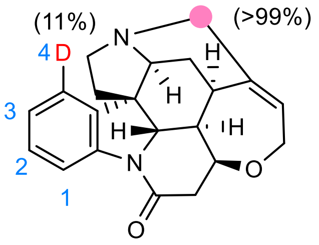

[D] Strychnine

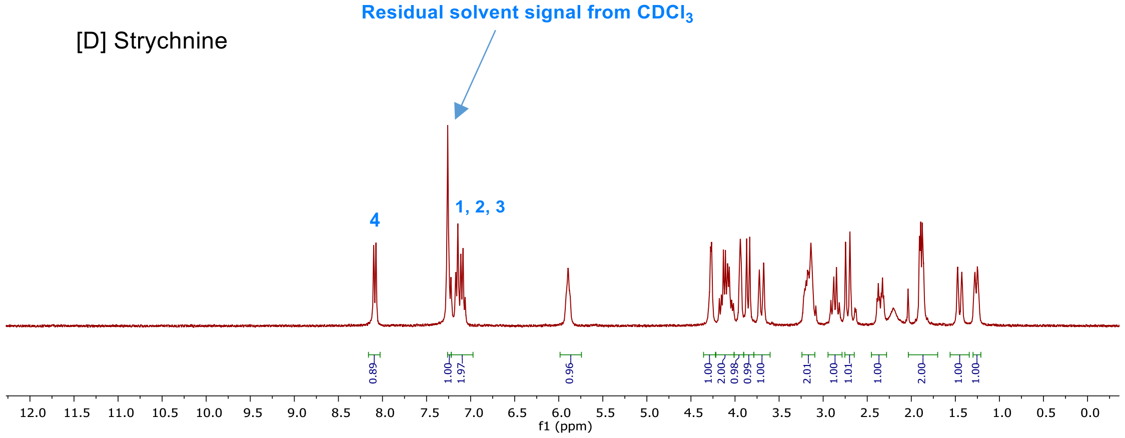

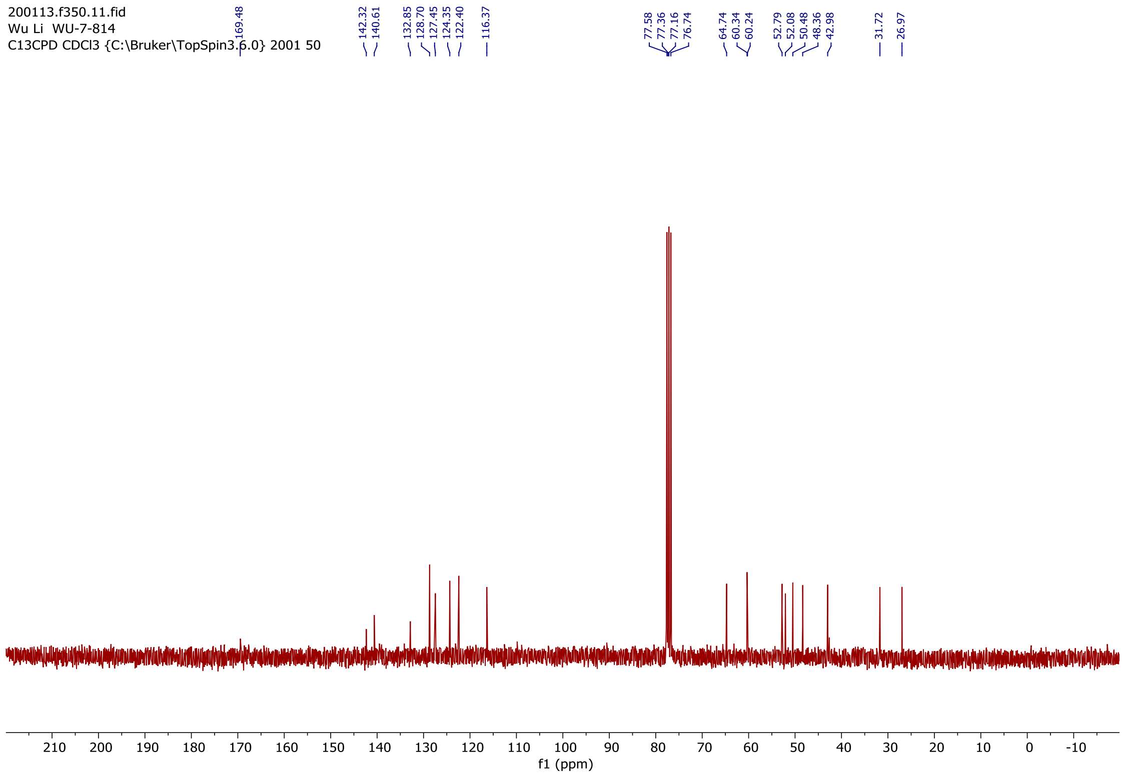

^{1}H NMR for 76a:

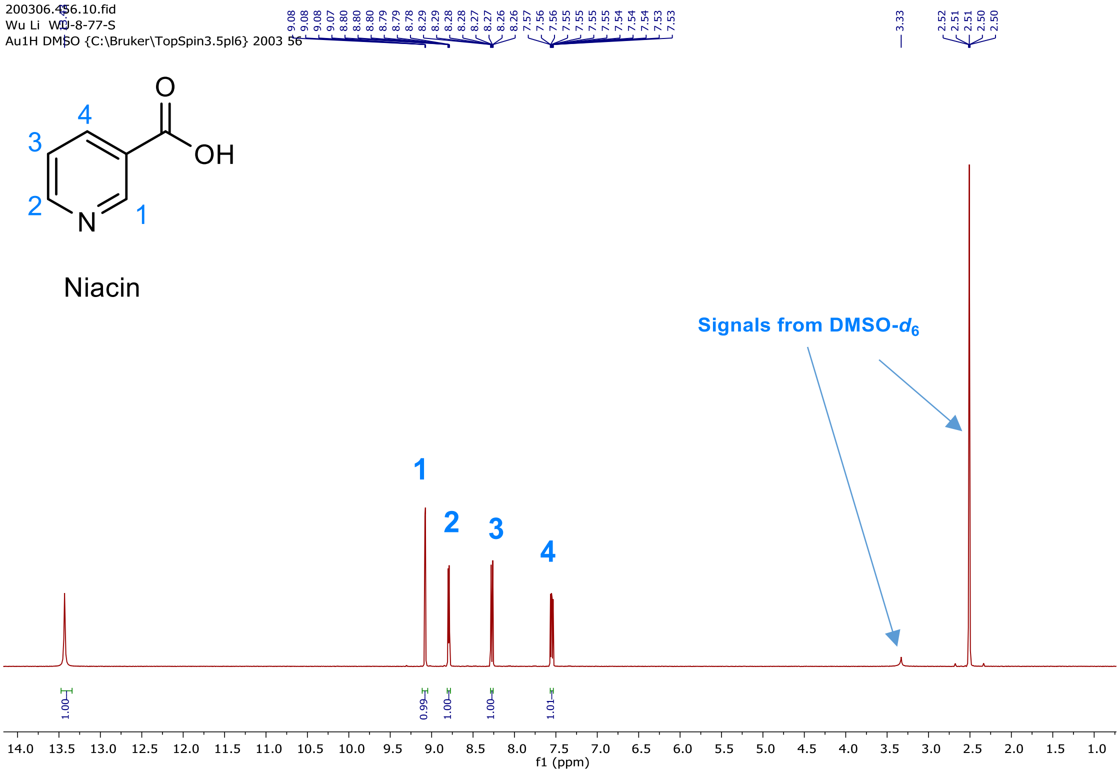

Original spectra for 76b:

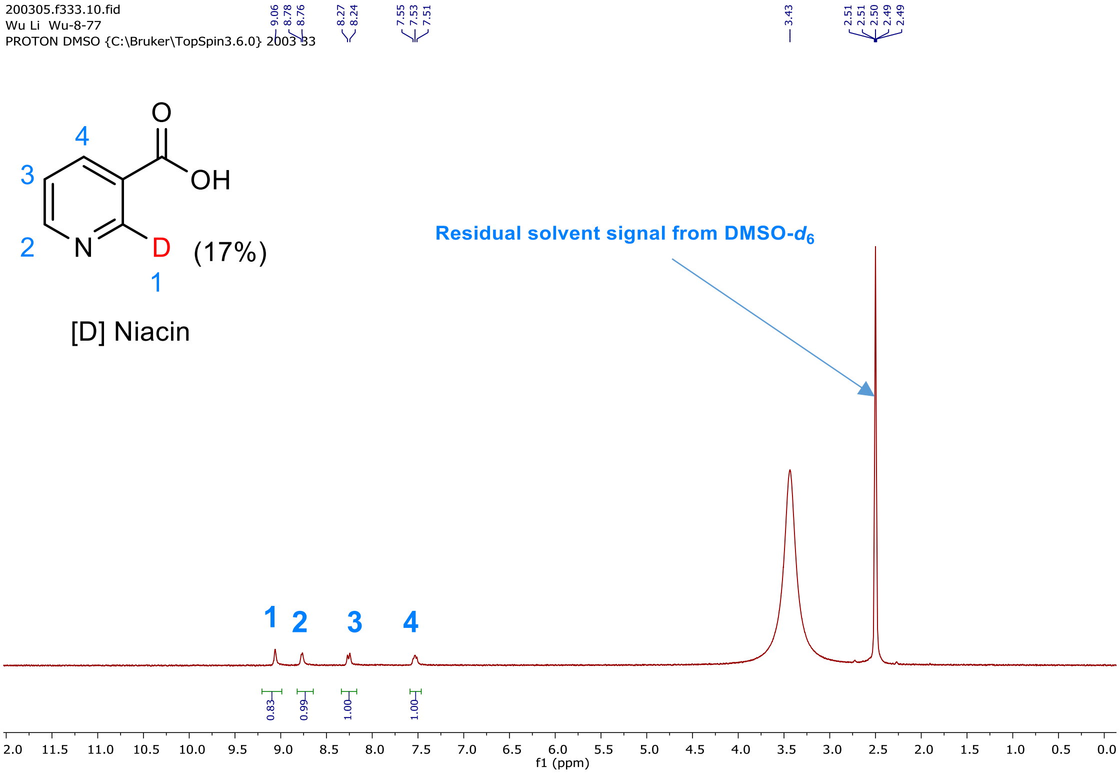

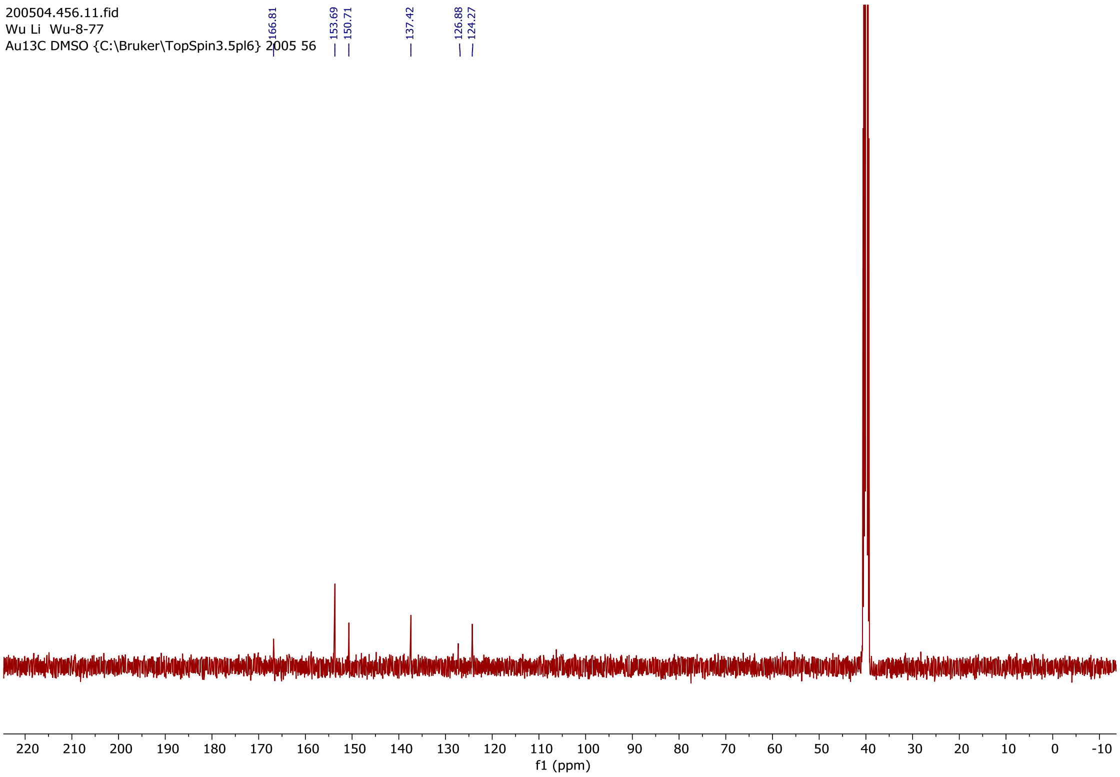

^{1}H NMR for 77a:

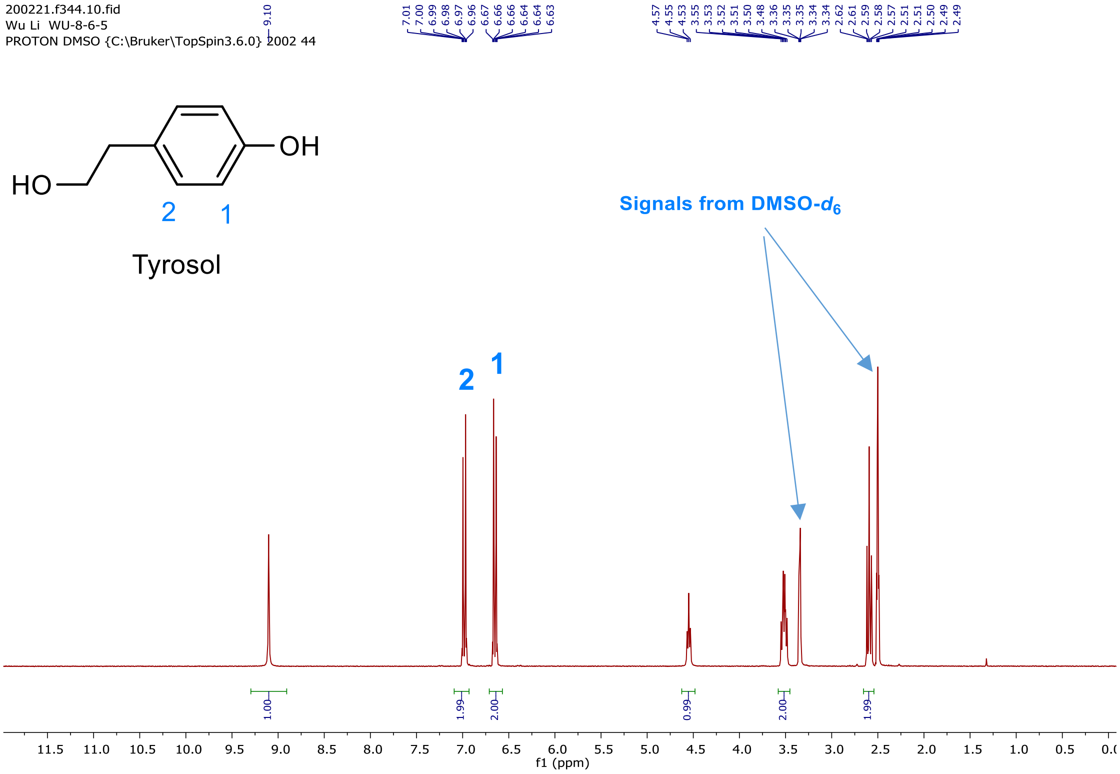

Original spectra for 77b:

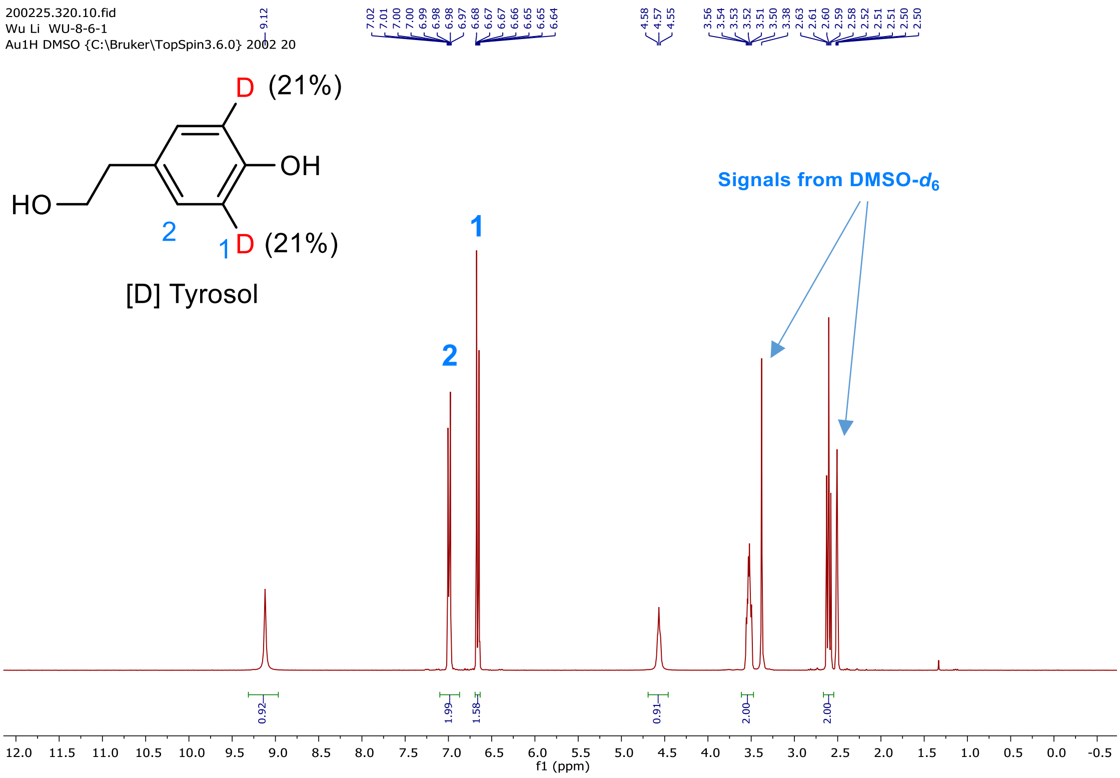

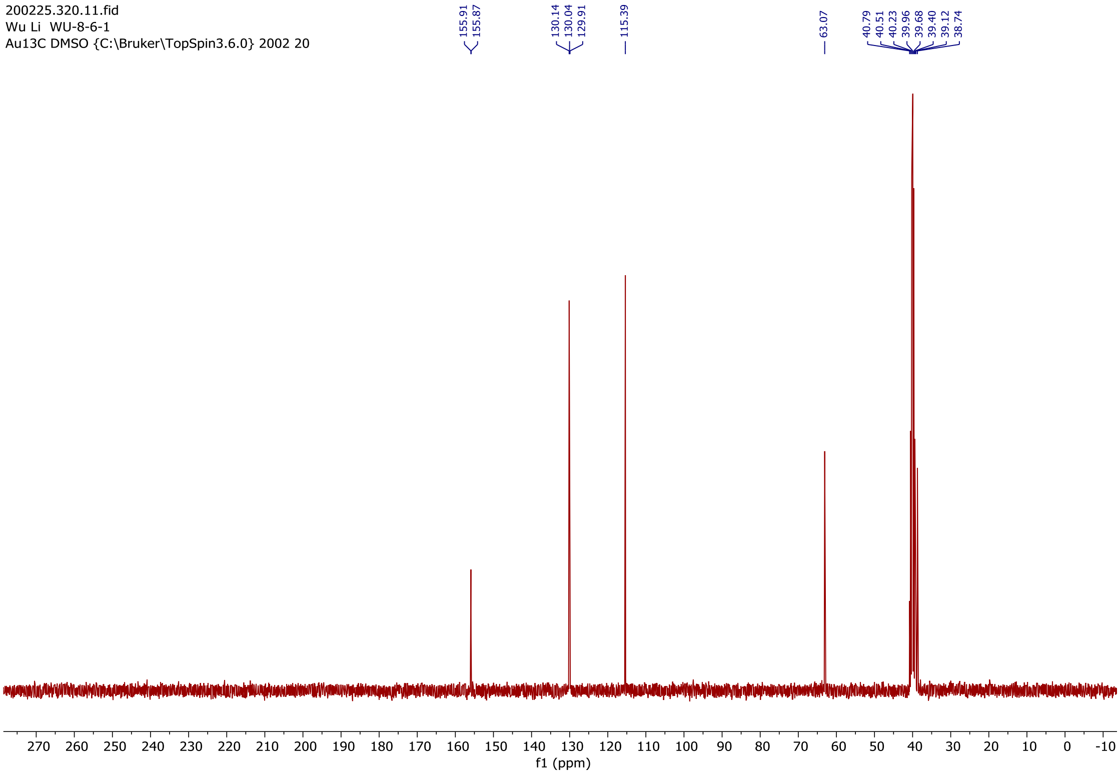

^{1}H NMR for 78a:

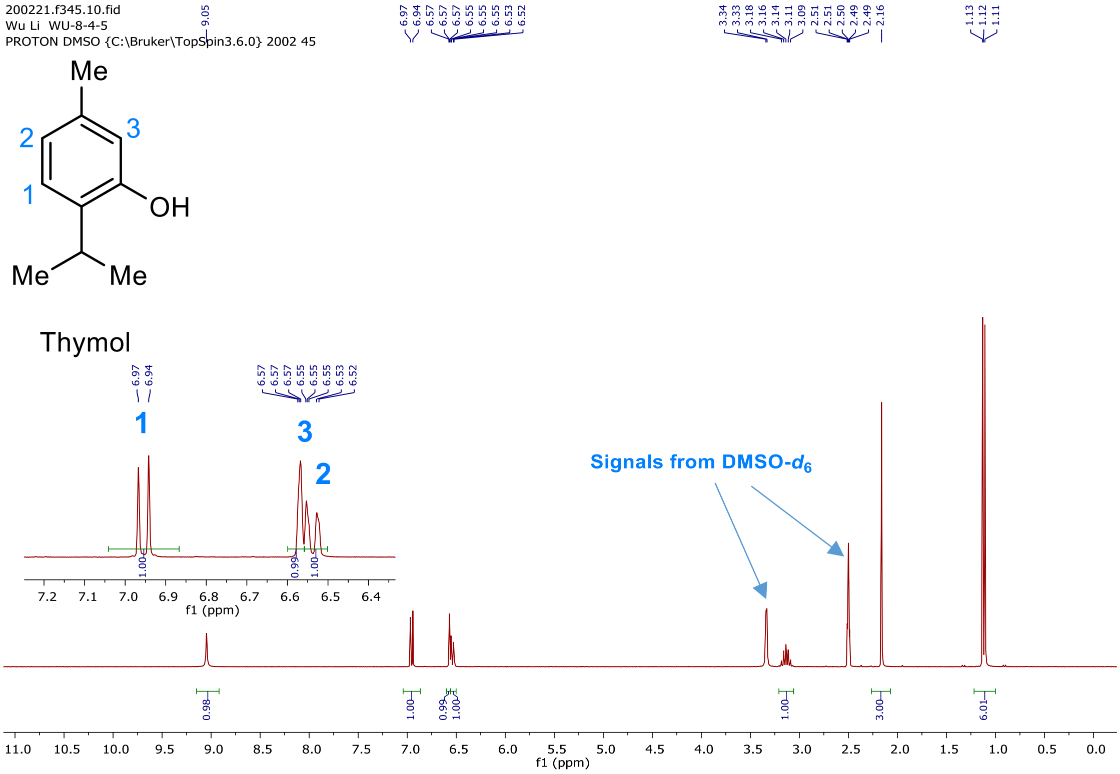

Original spectra for 78b:

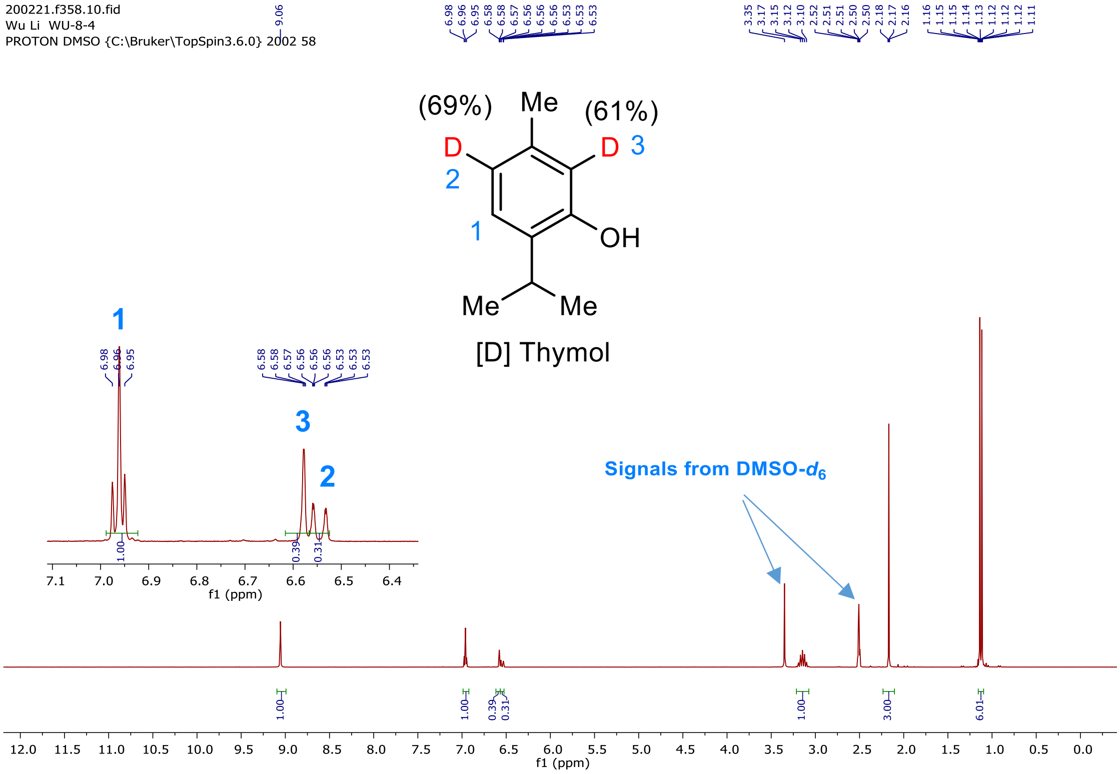

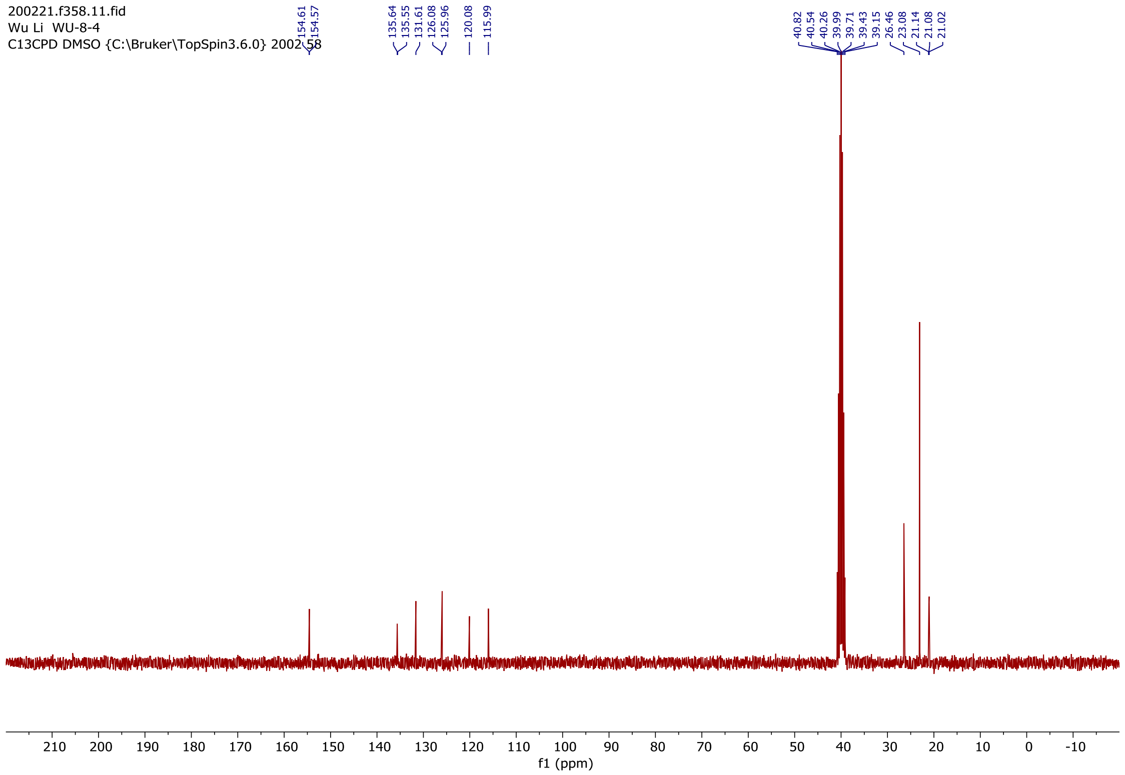

^{1}H NMR for 79a:

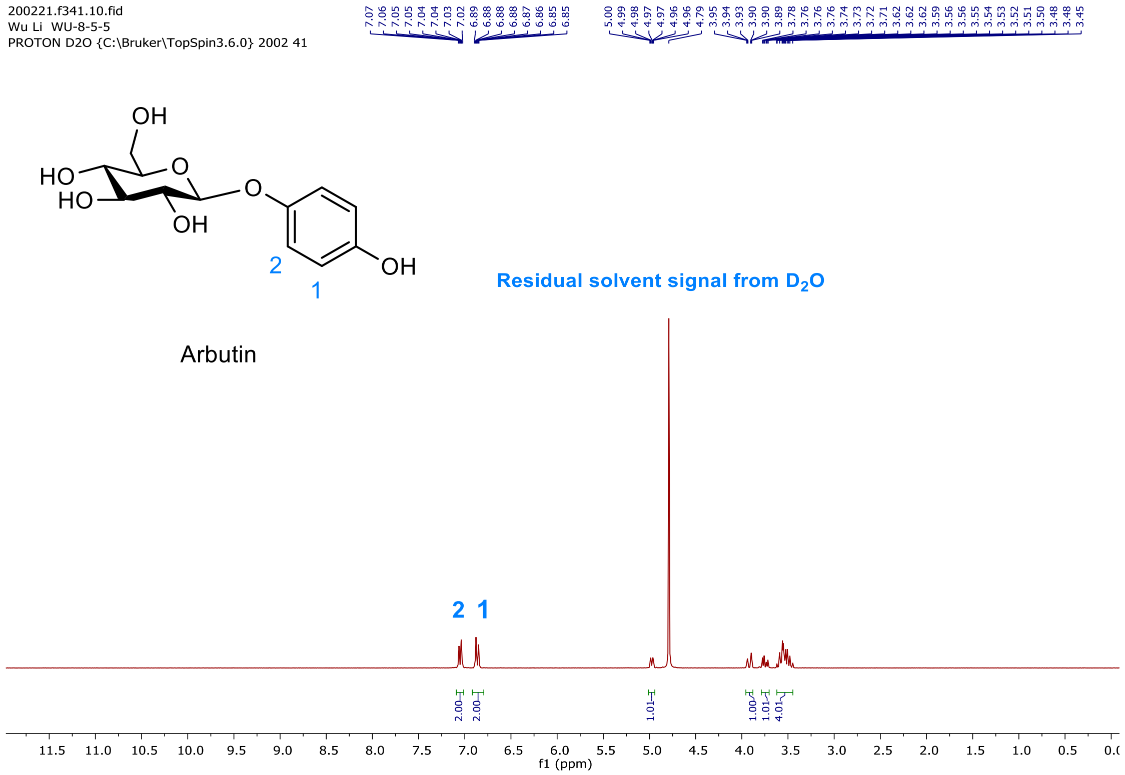

Original spectra for 79b:

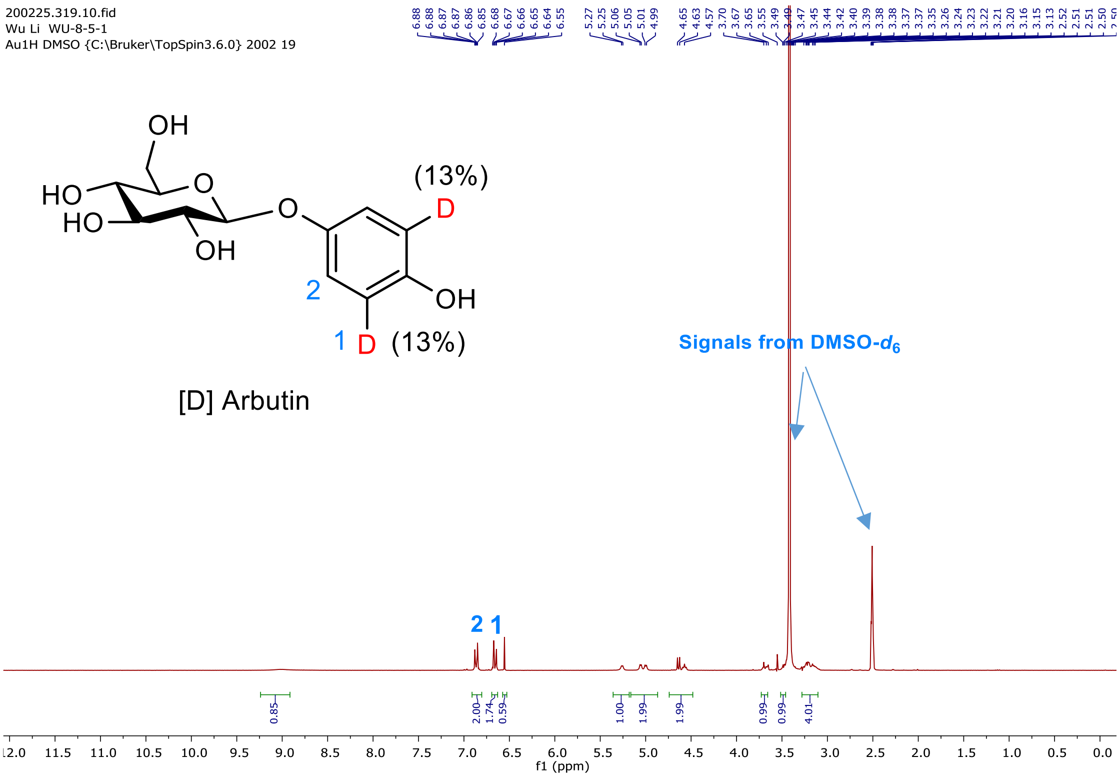

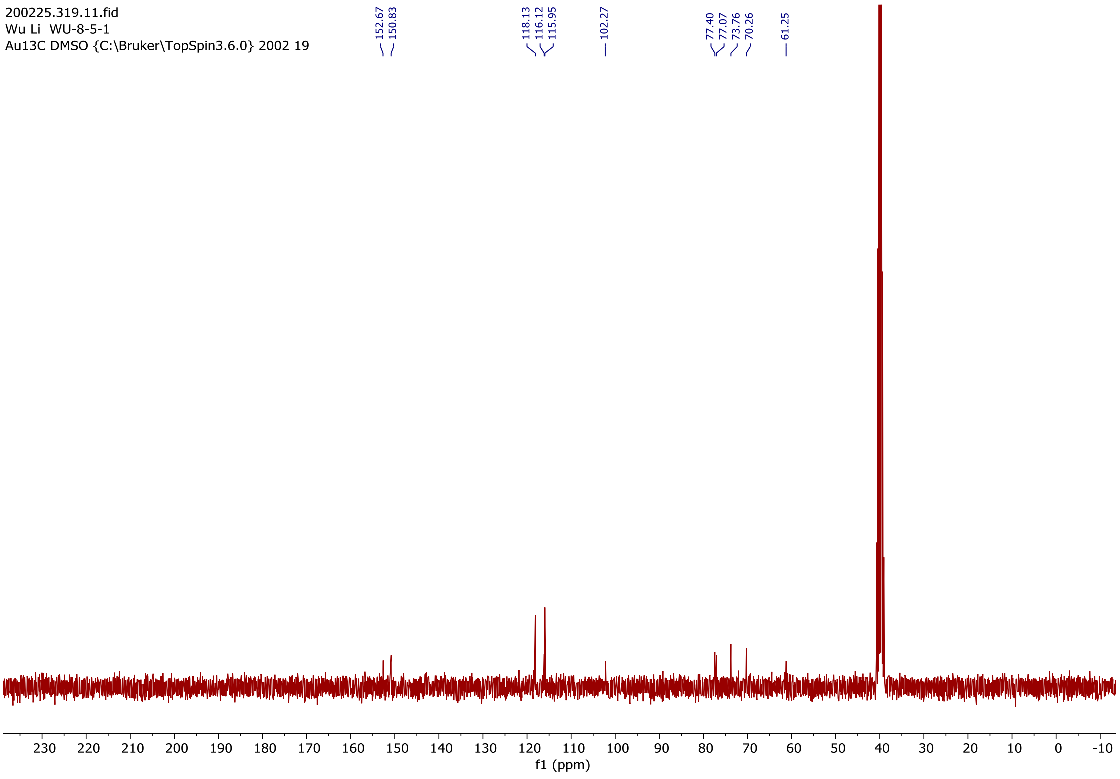

^{1}H NMR for 80a:

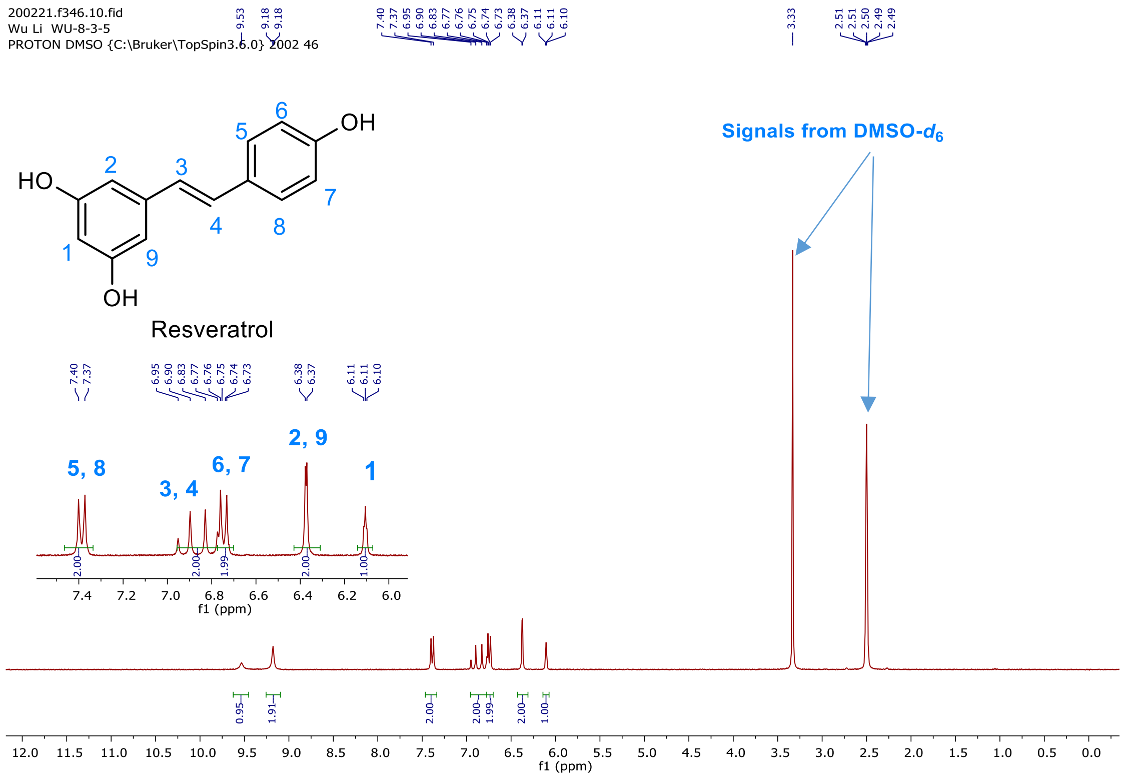

Original spectra for 80b:

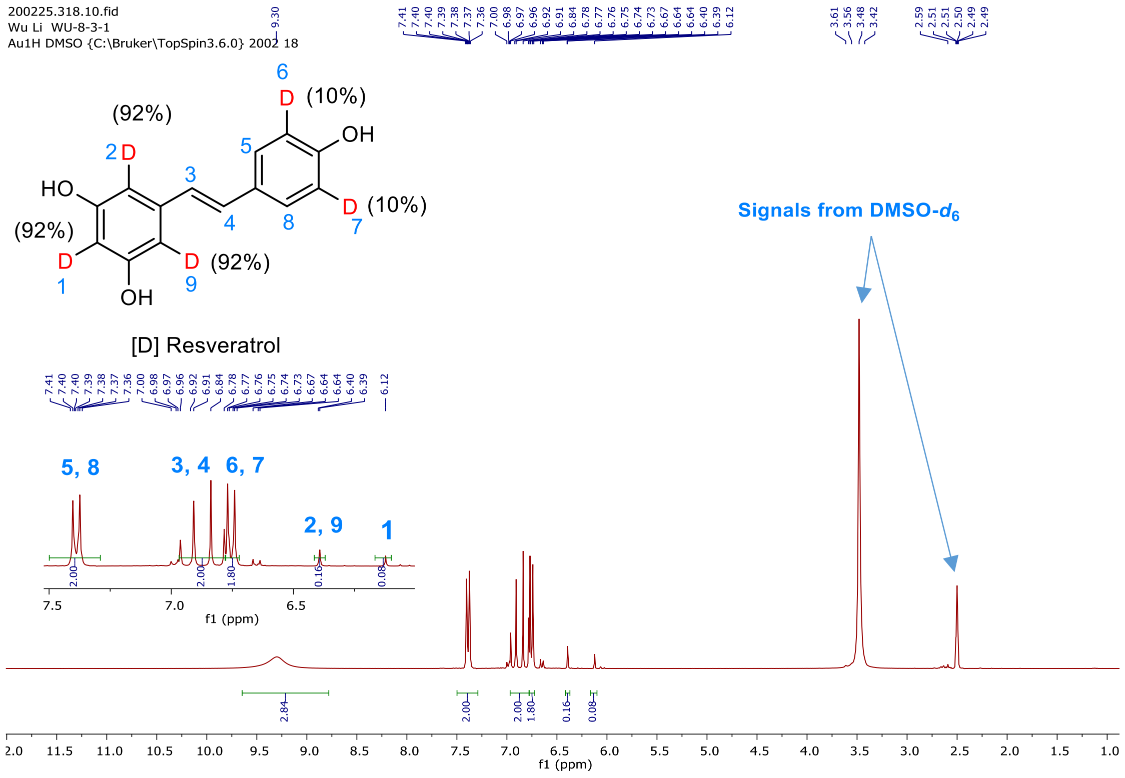

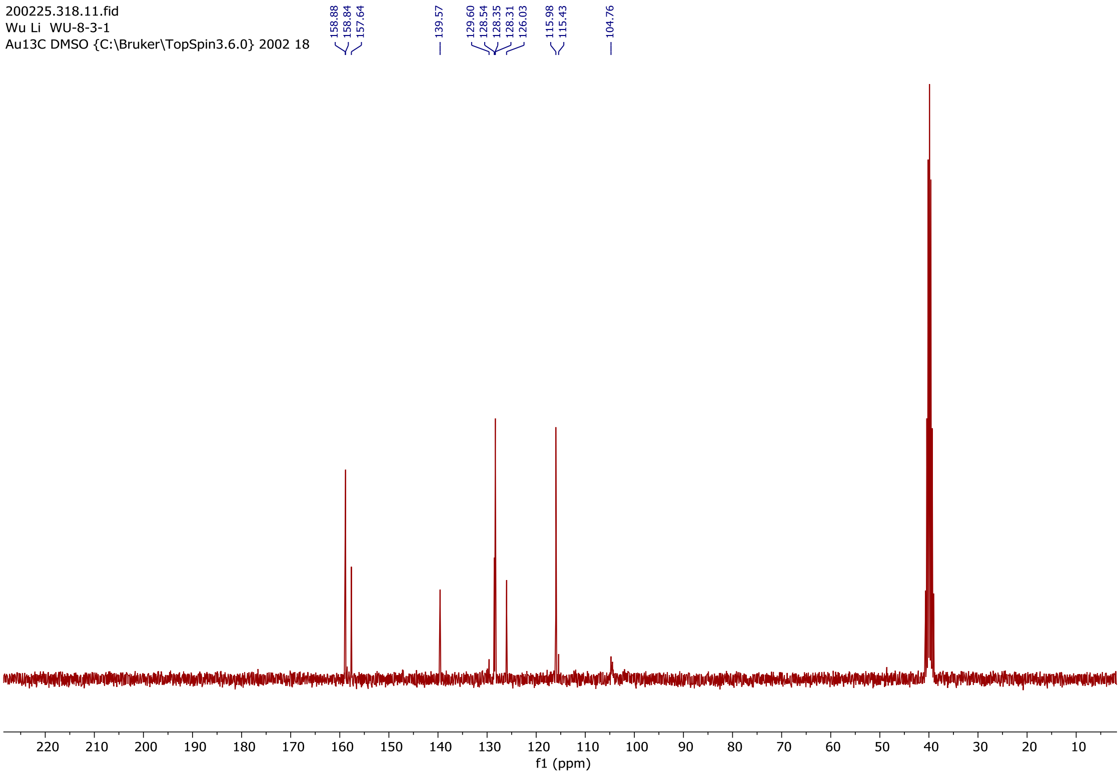

^{1}H NMR for 81a:

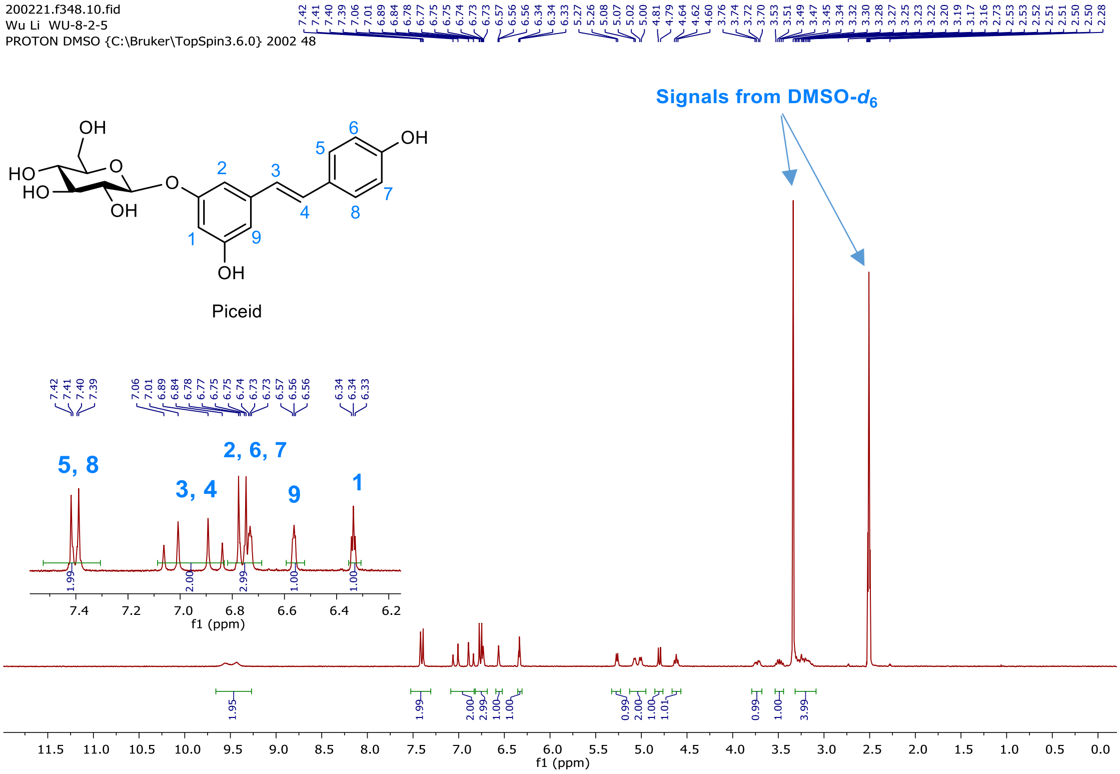

Original spectra for 81b:

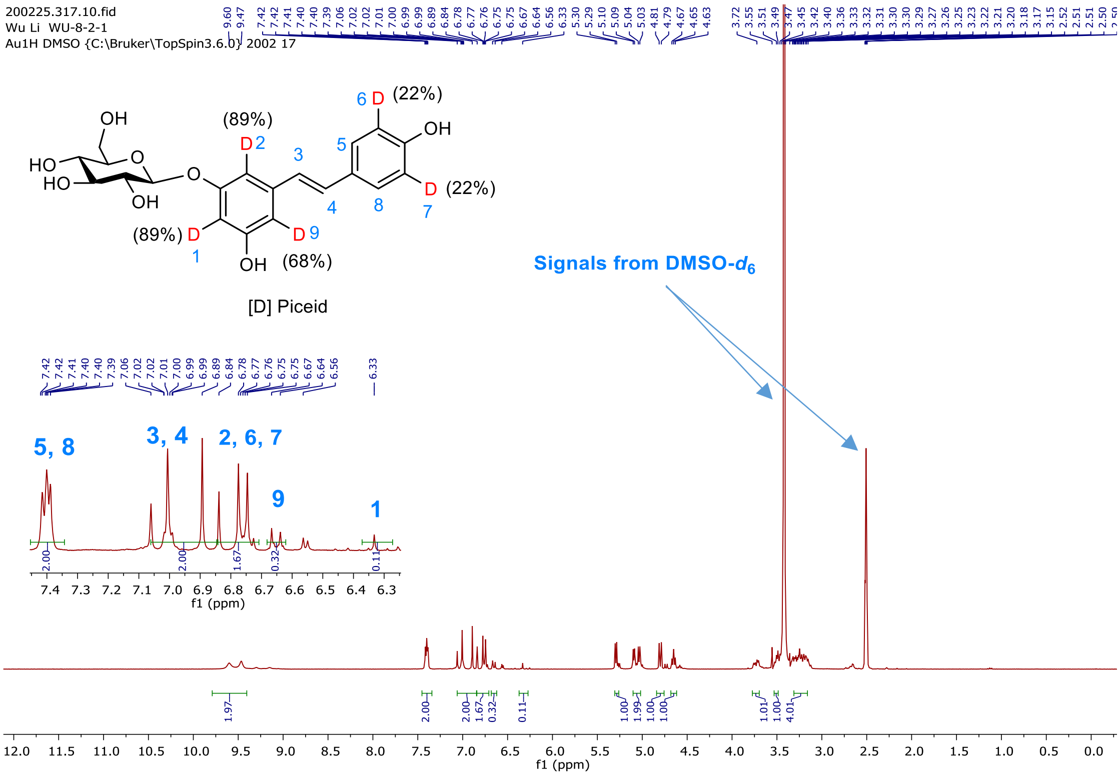

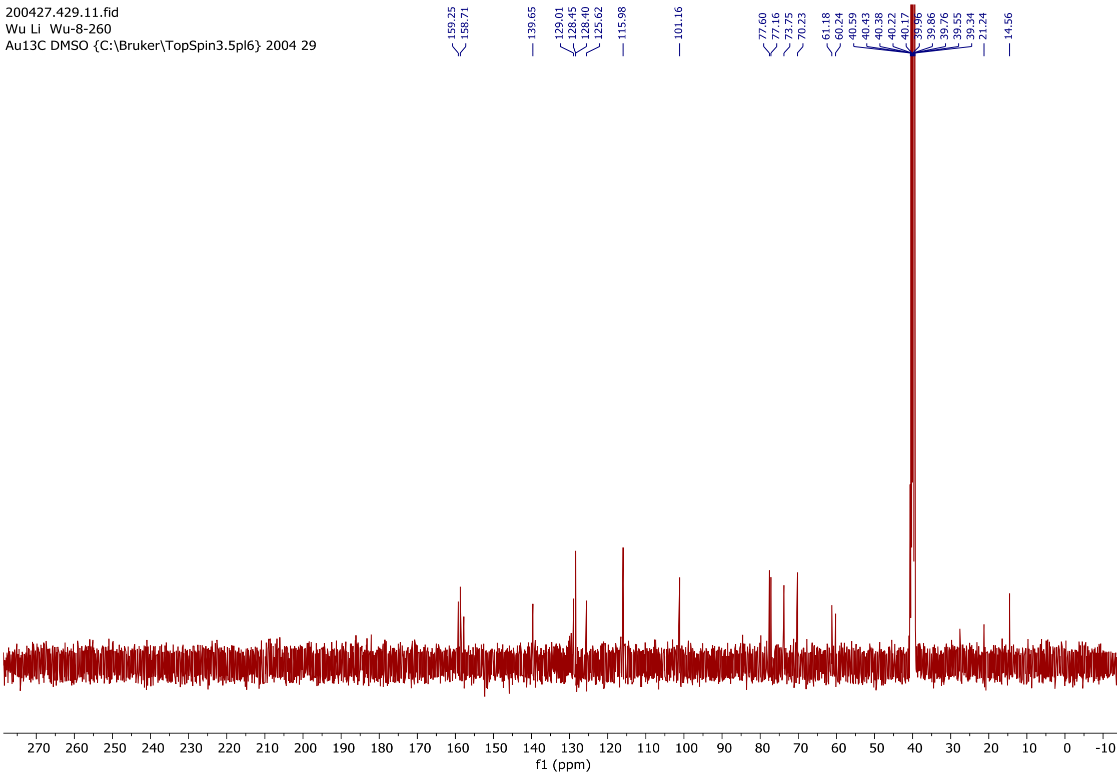

^{1}H NMR for 82a:

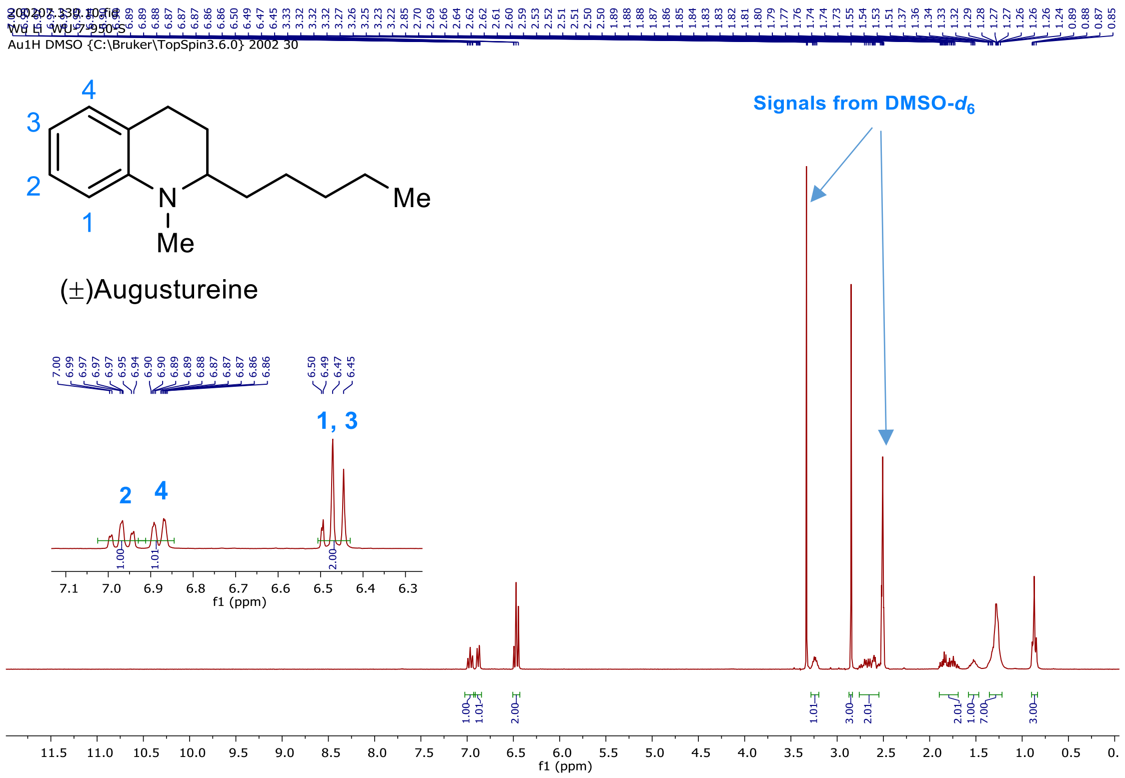

Original spectra for 82b:

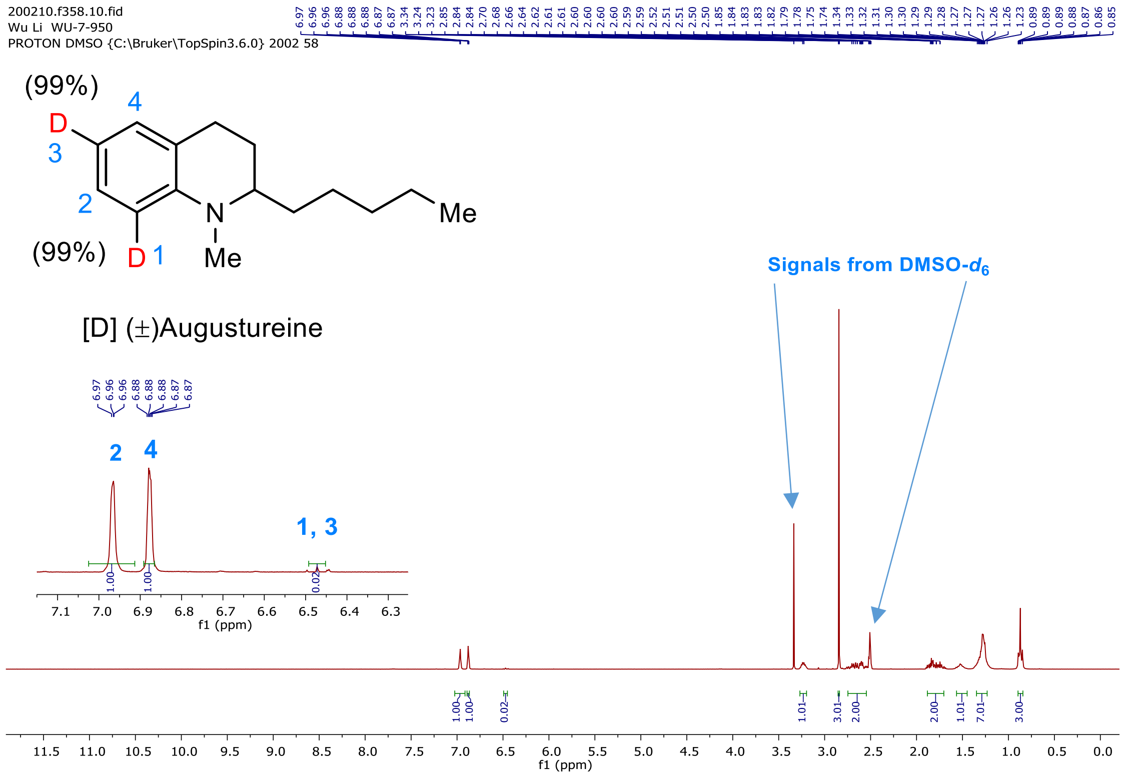

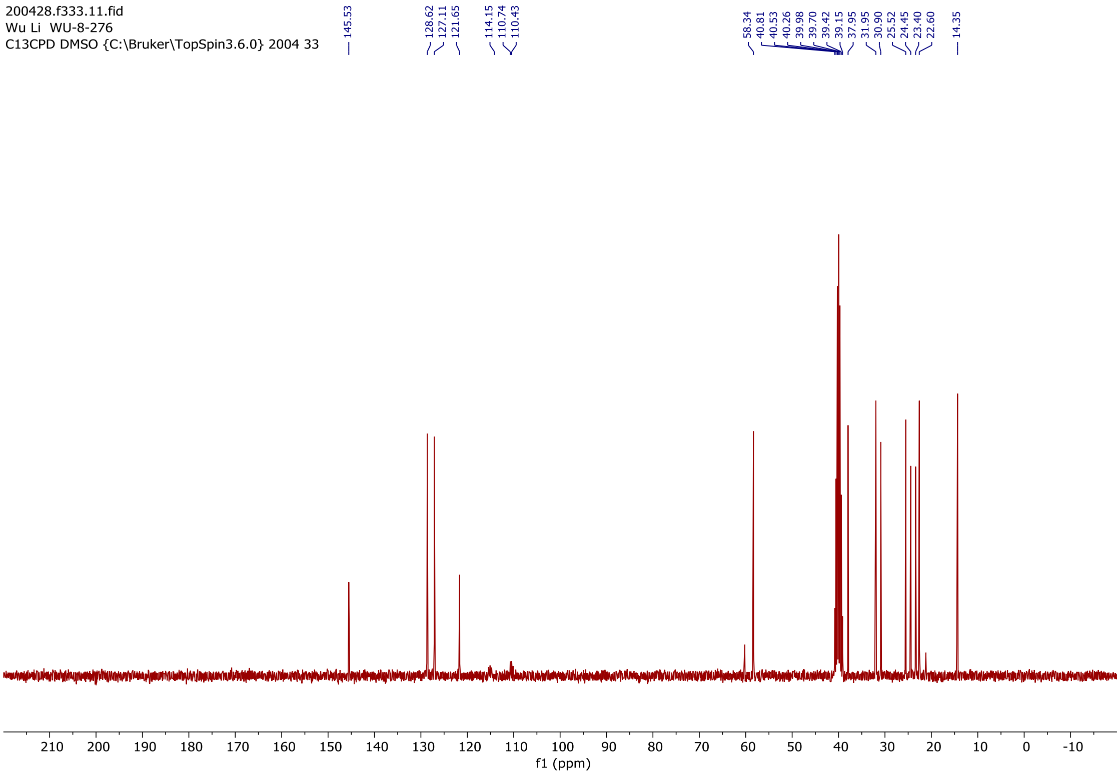

^{1}H NMR for 83a:

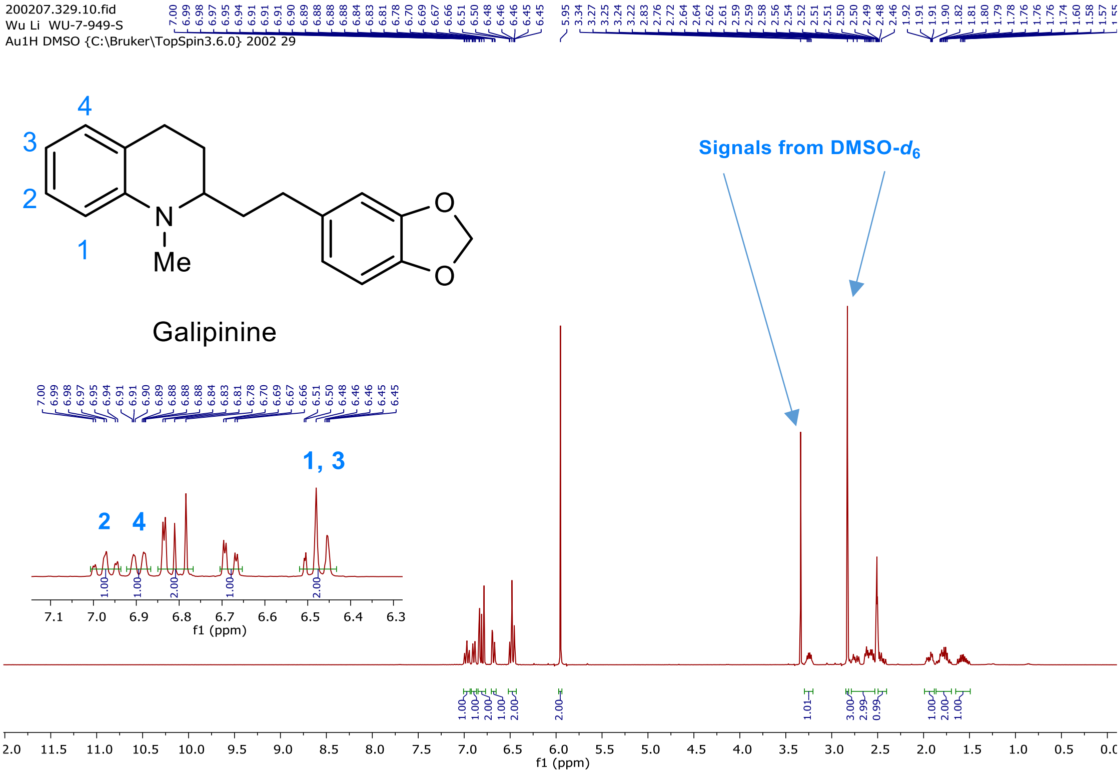

Original spectra for 83b:

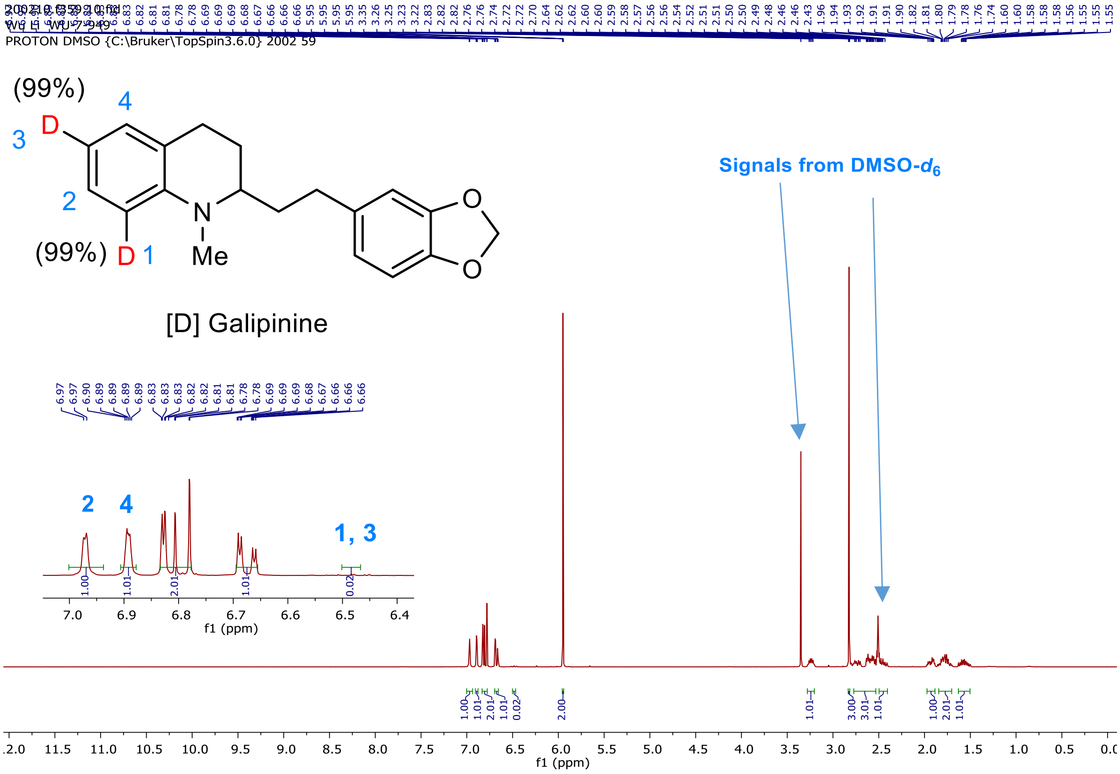

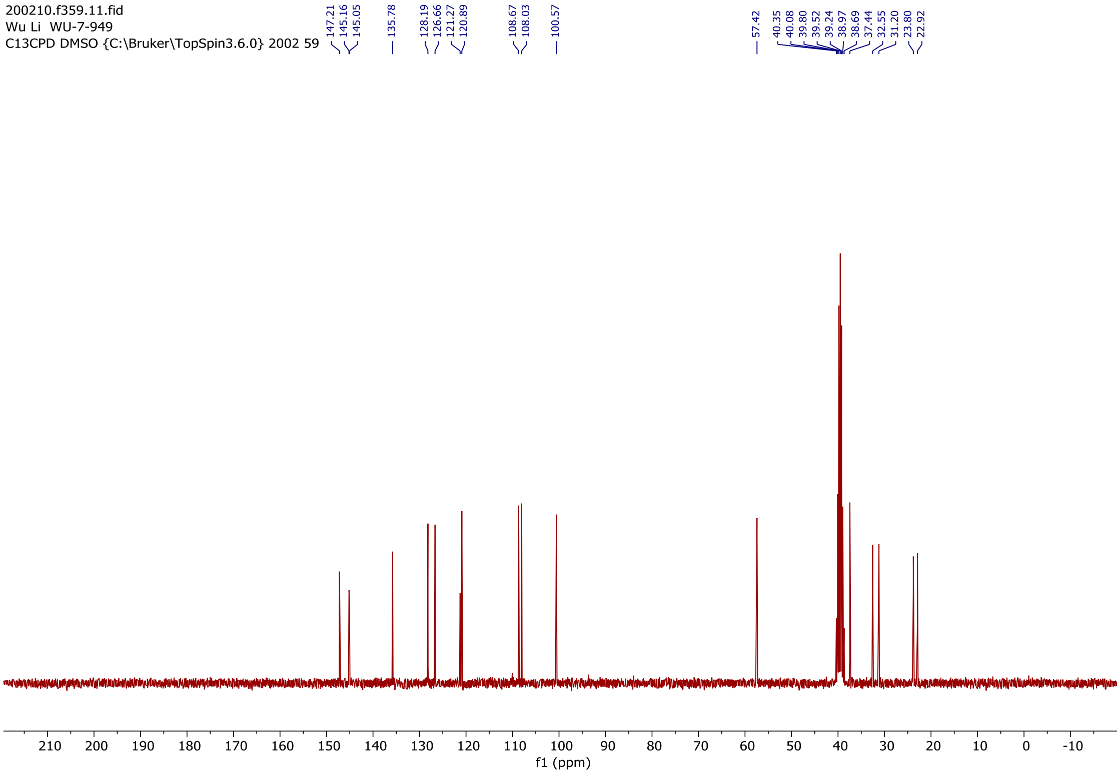

^{1}H NMR for 84a:

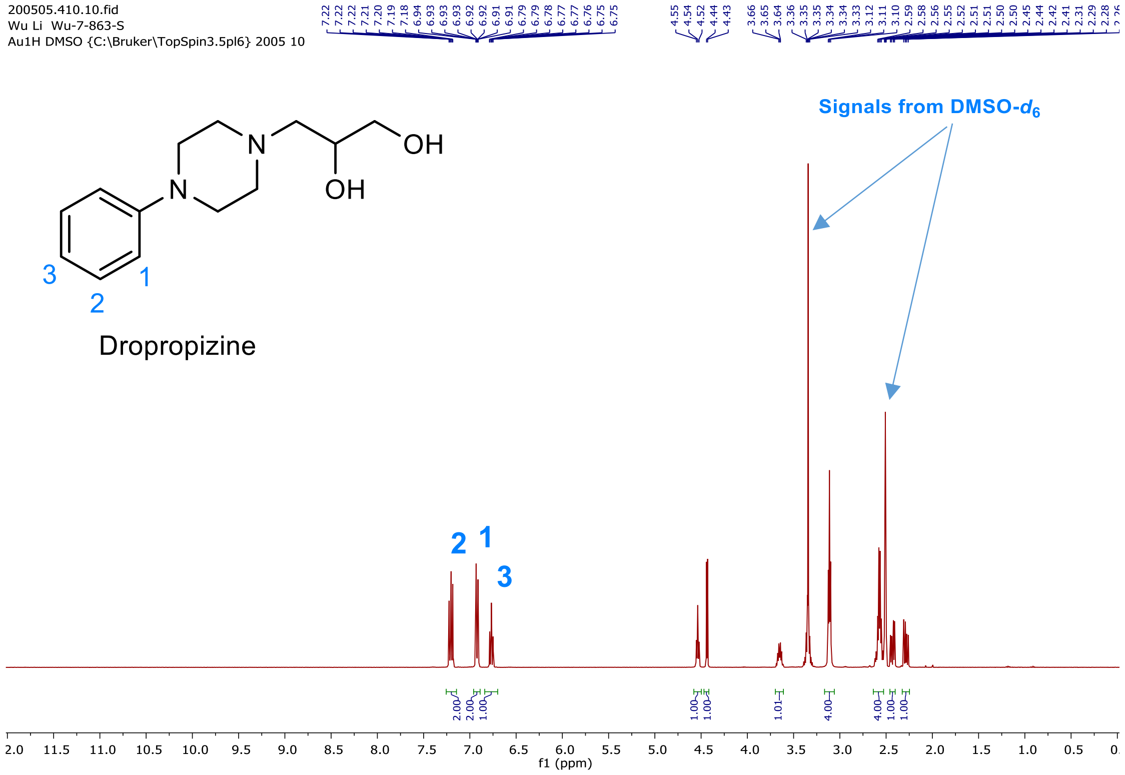

Original spectra for 84b:

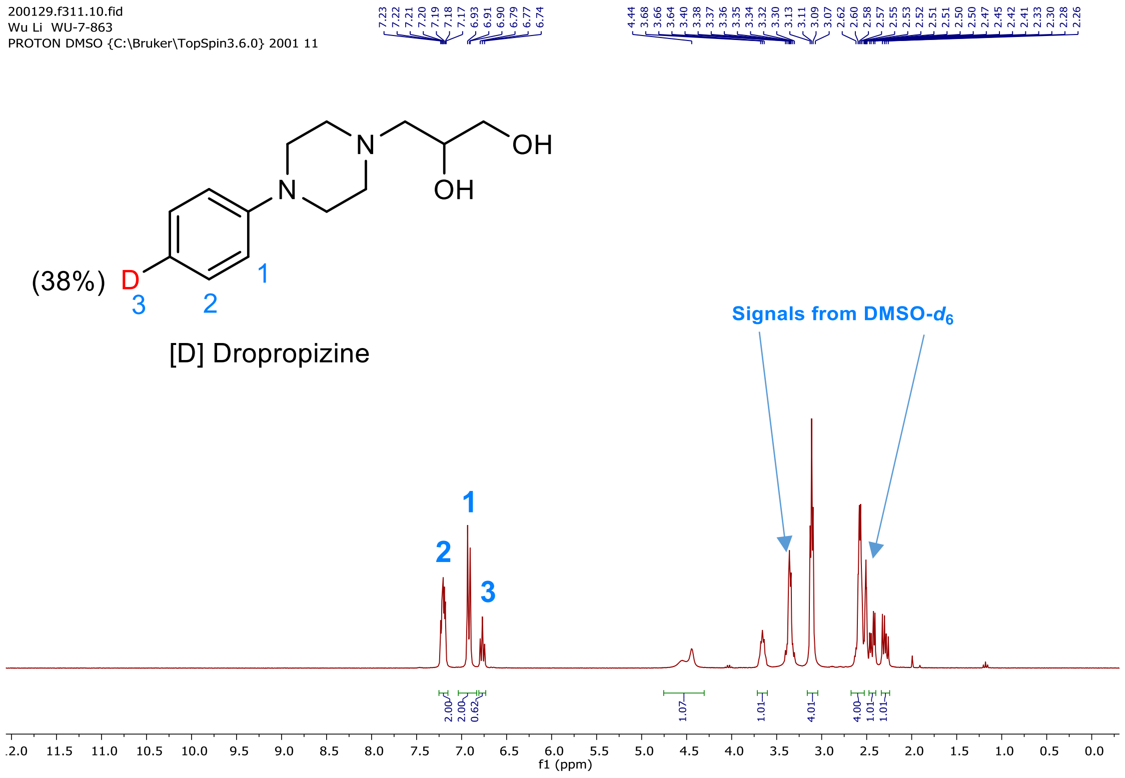

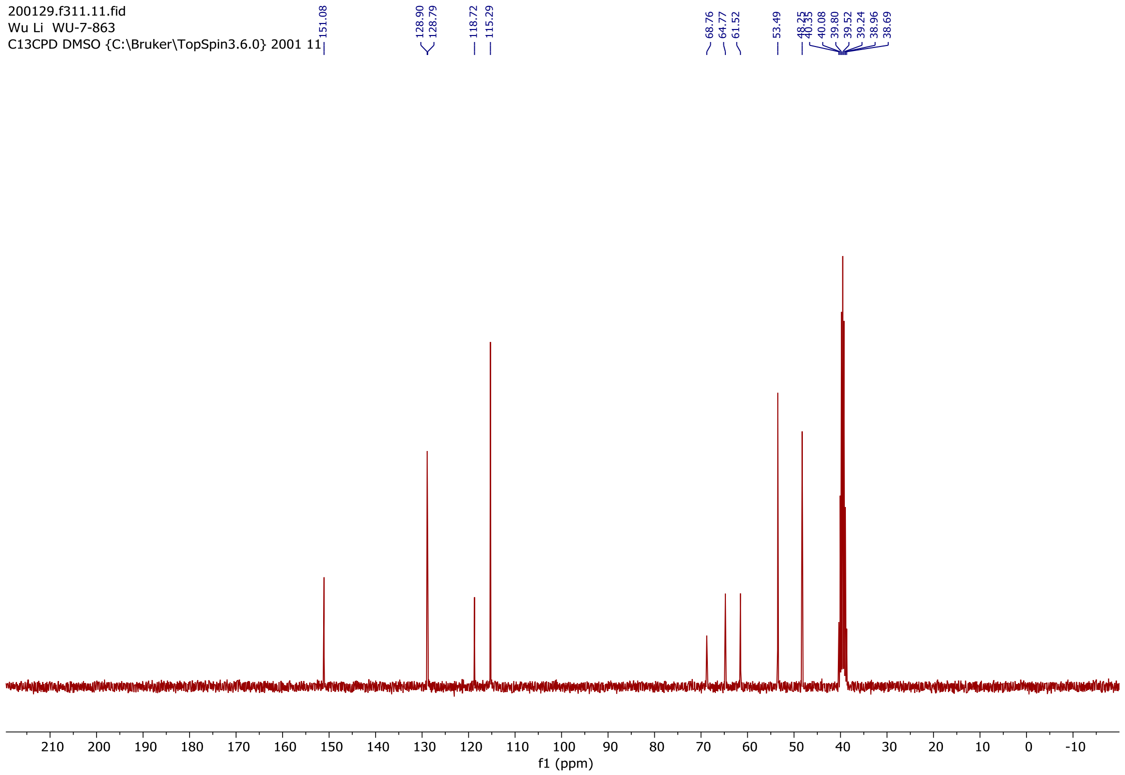

^{1}H NMR for 85a:

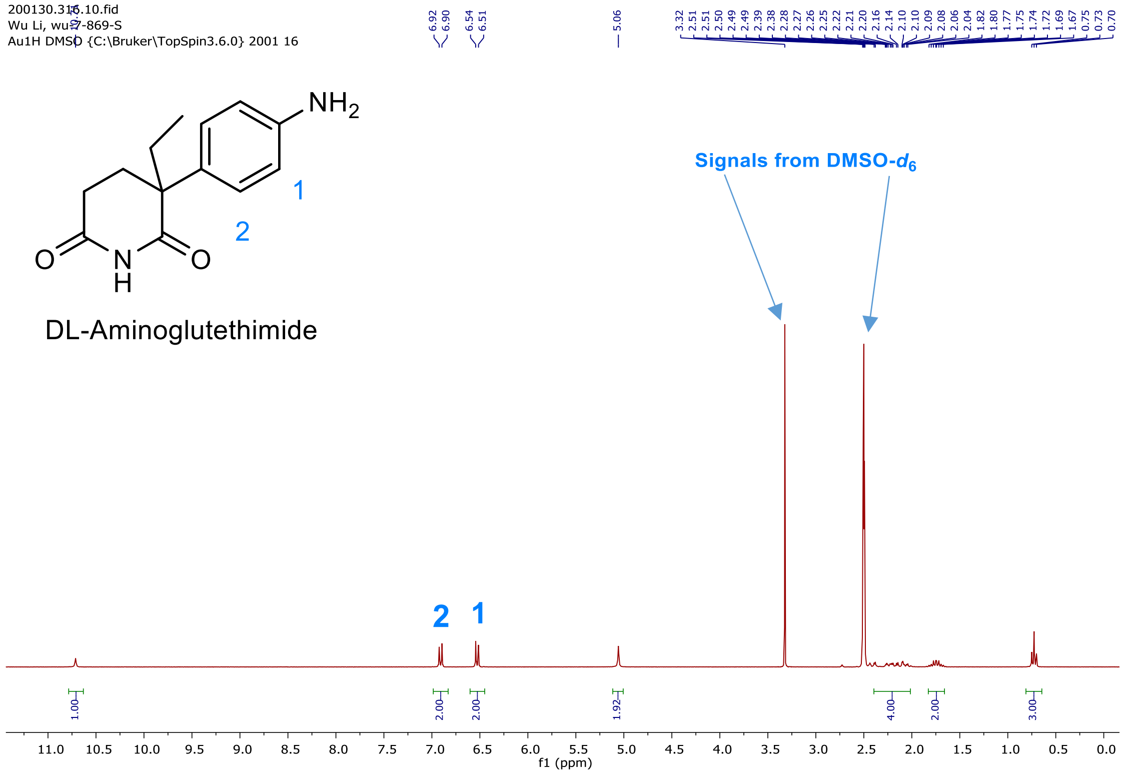

Original spectra for 85b:

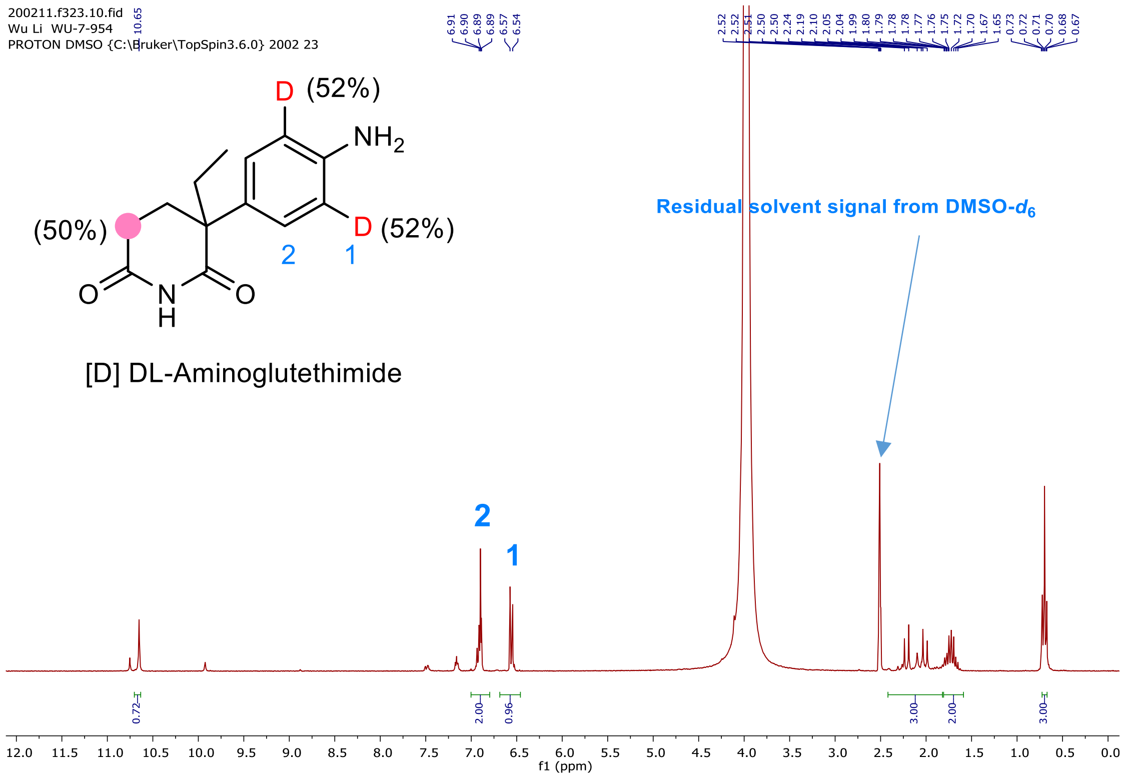

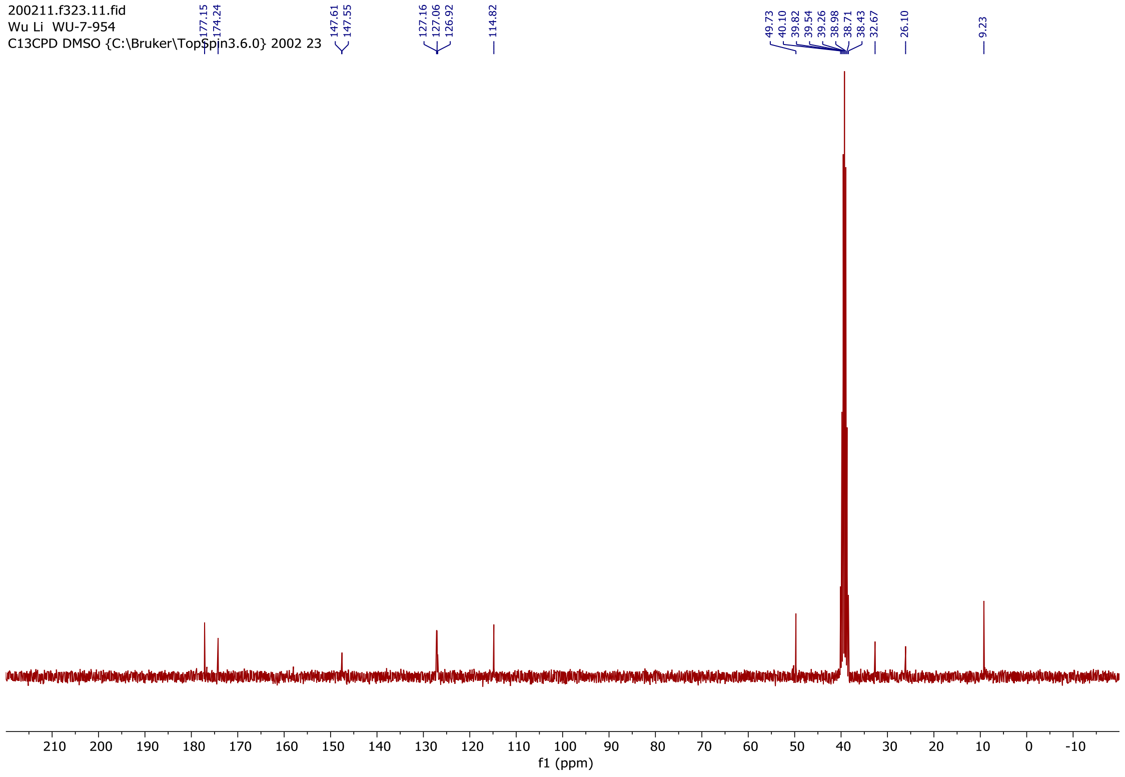

^{1}H NMR for 86a:

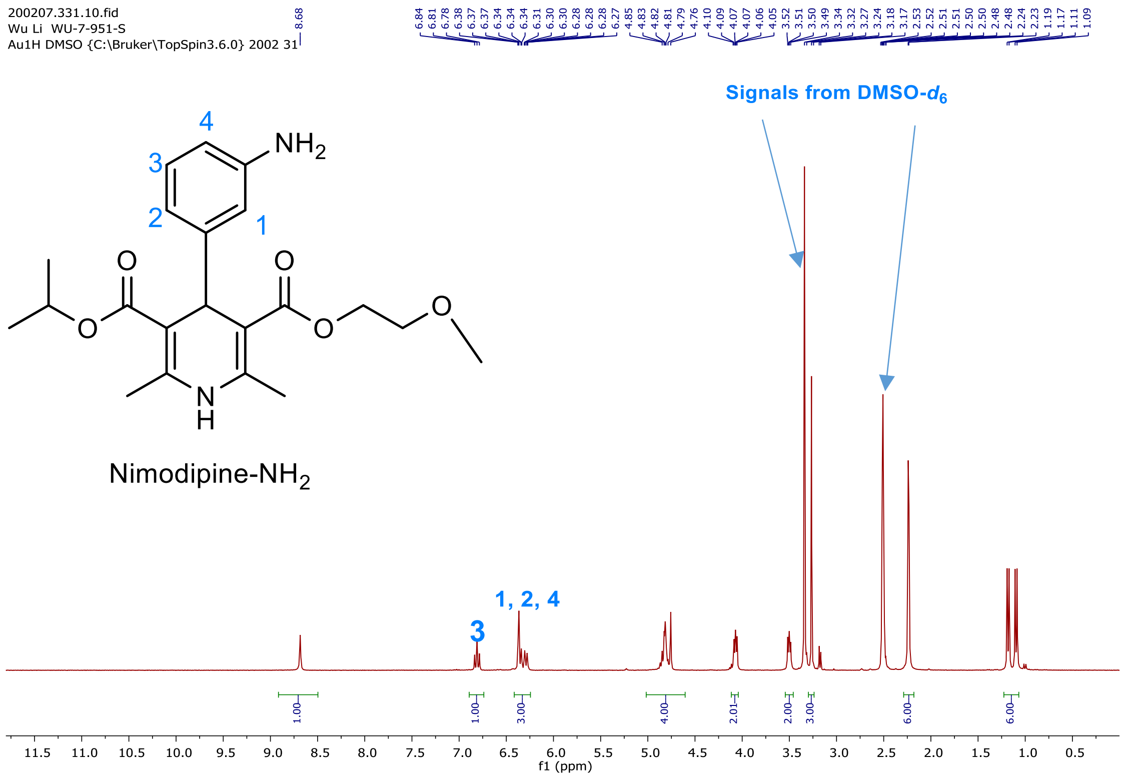

Original spectra for 86b:

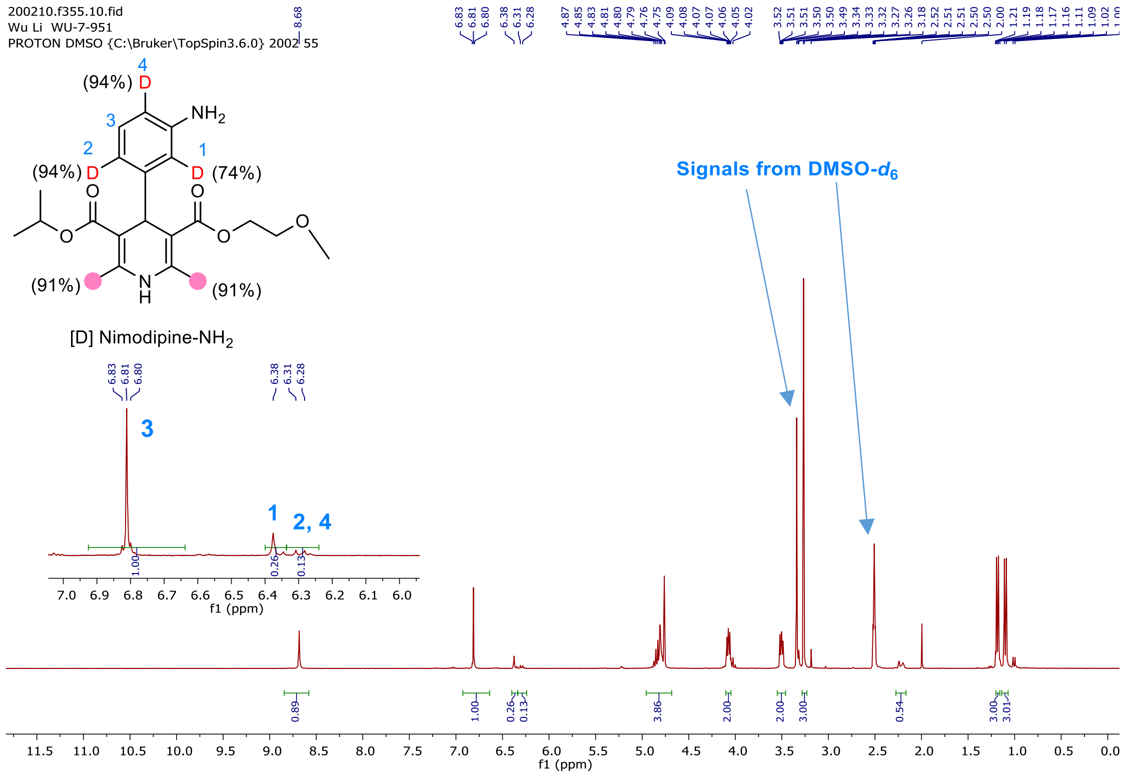

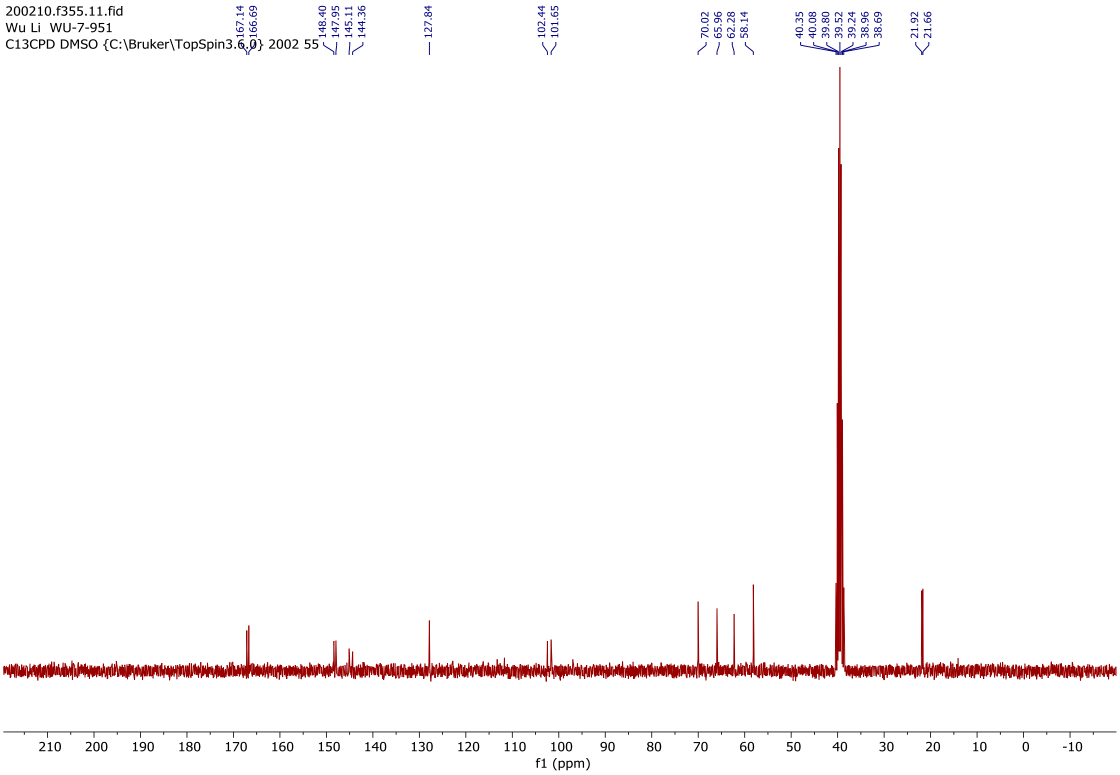

^{1}H NMR for 87a:

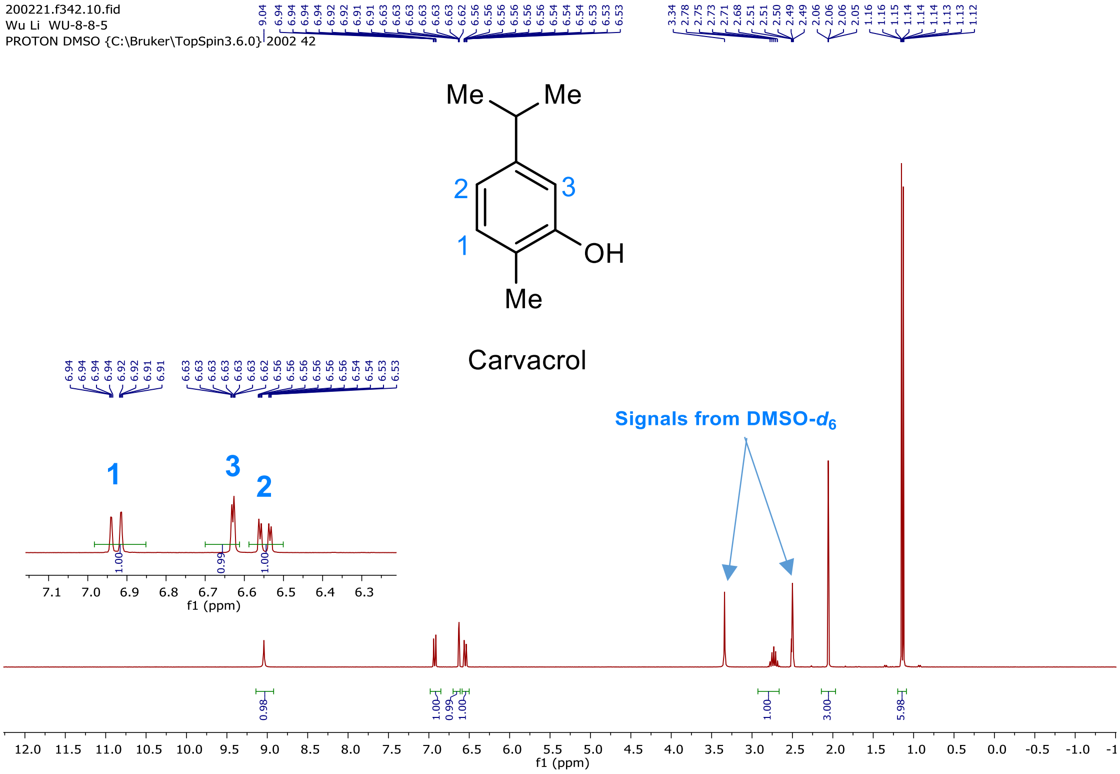

Original spectra for 87b:

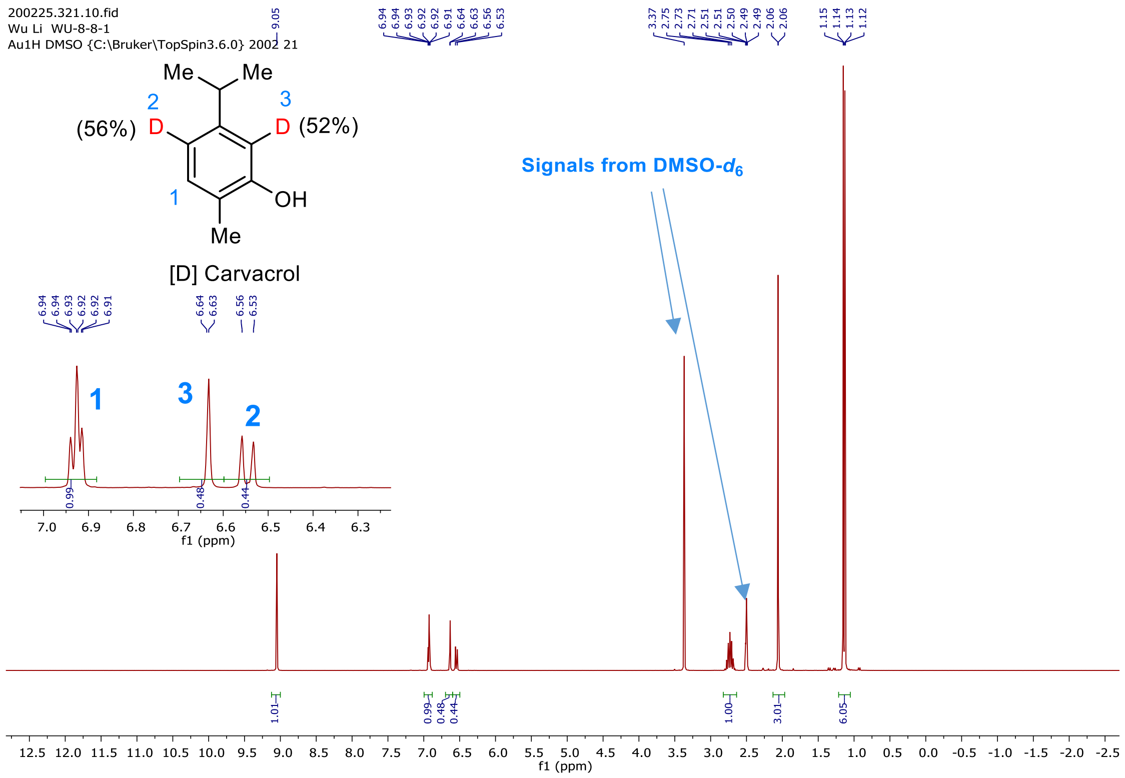

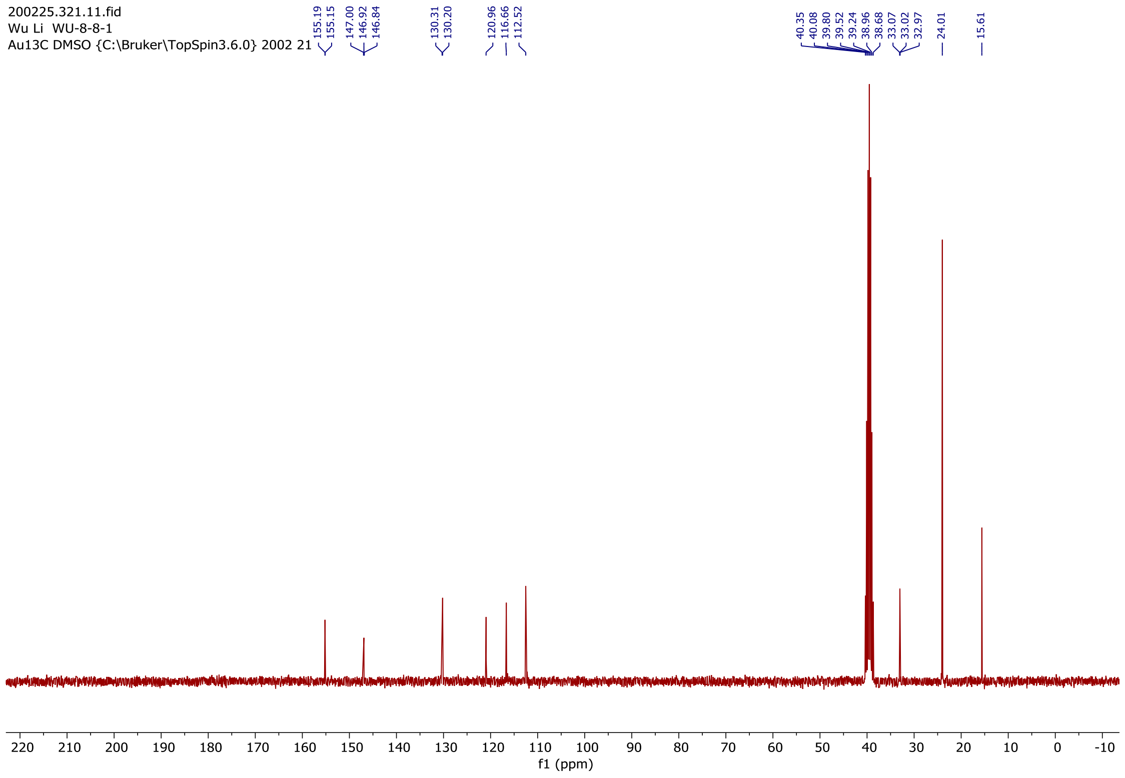

^{1}H NMR for 88a:

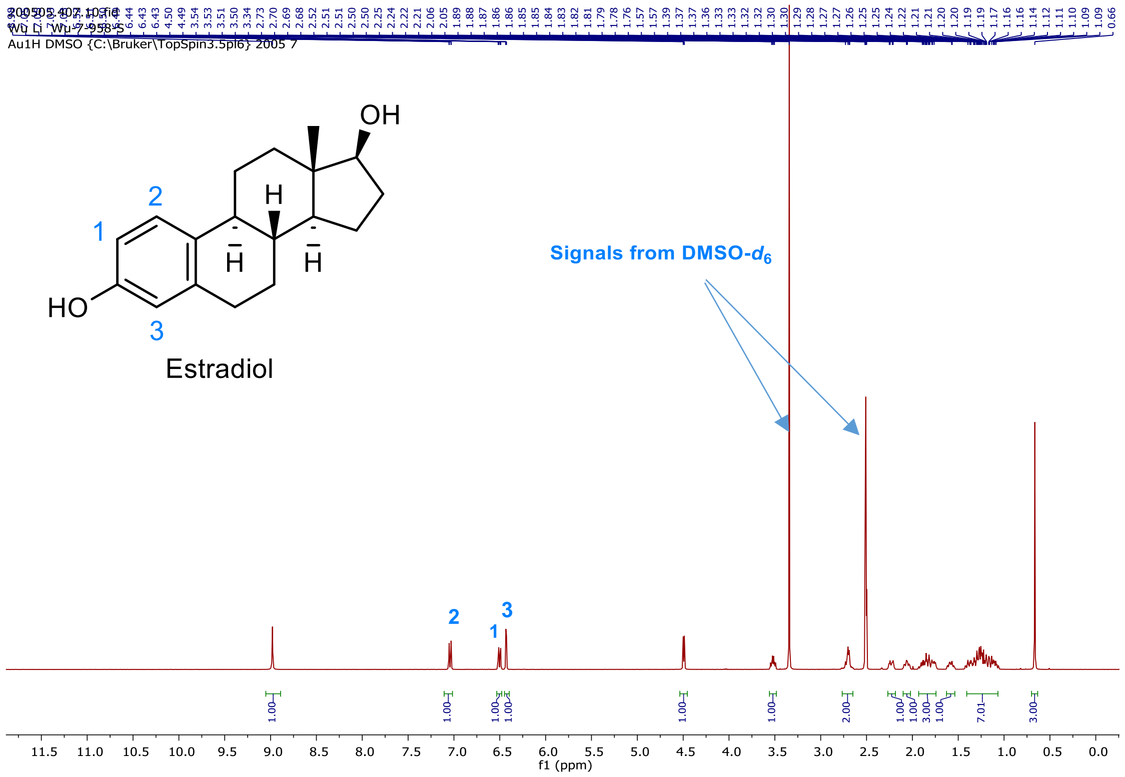

Original spectra for 88b:

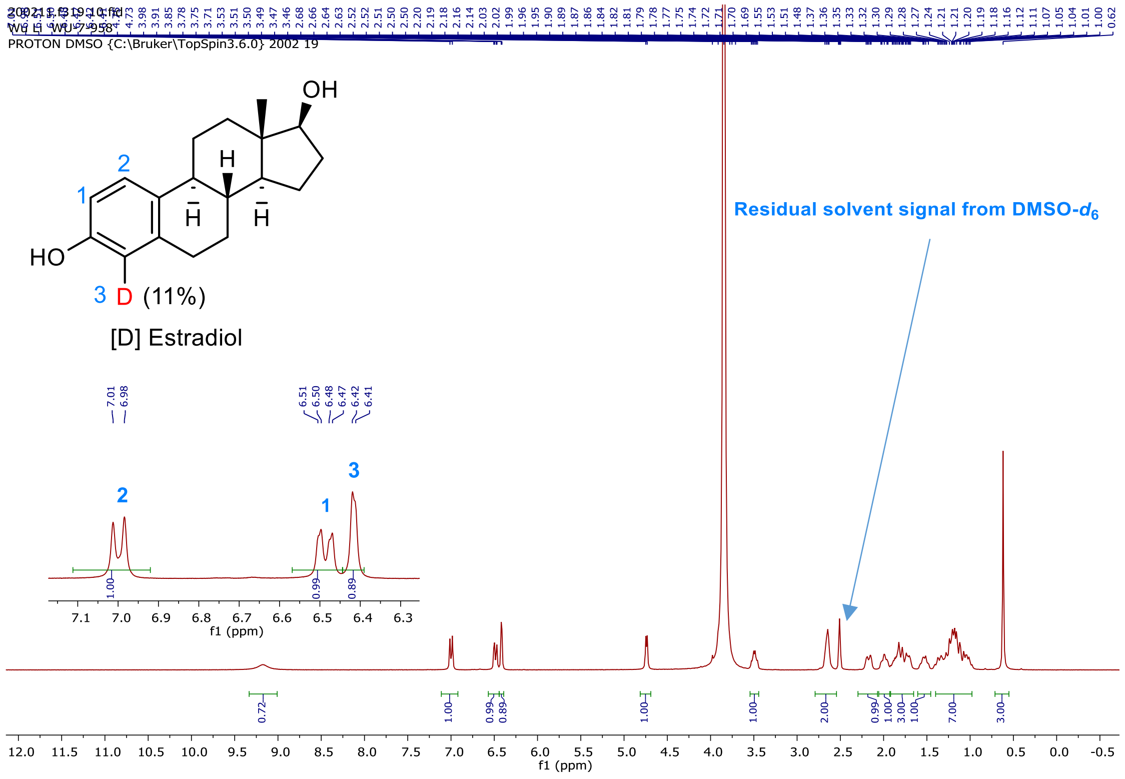

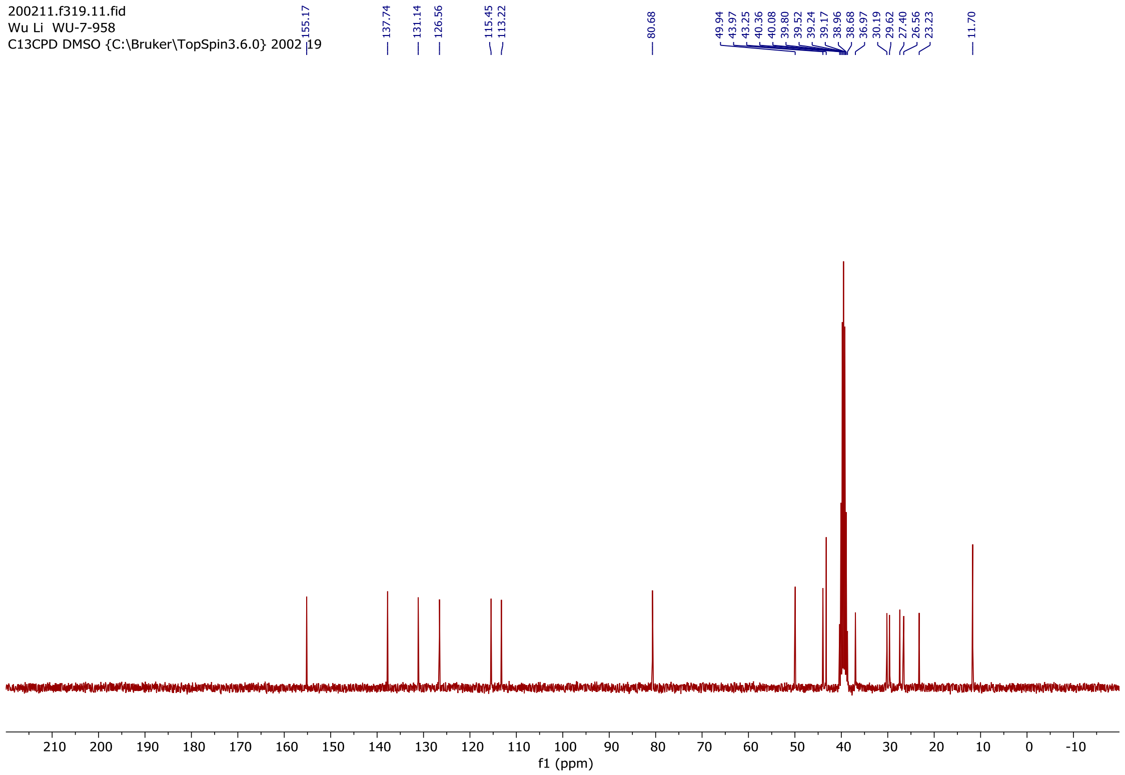

^{1}H NMR for the substrate of 89a:

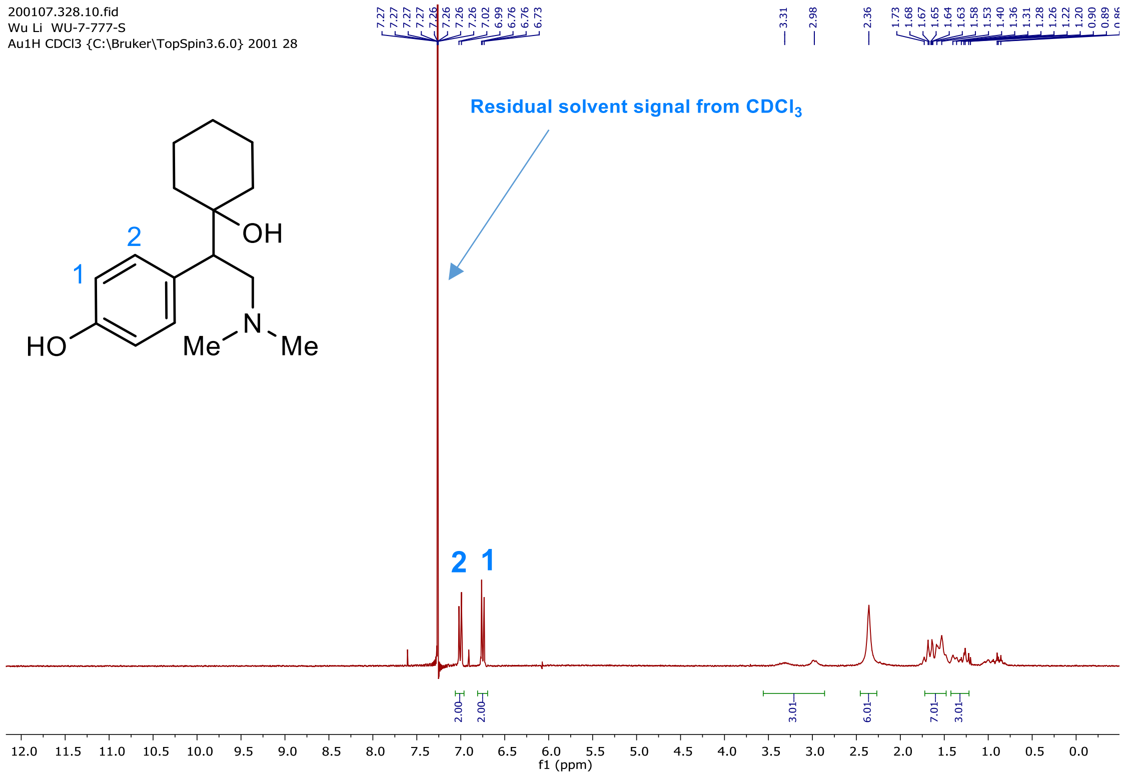

Original spectra for 89b:

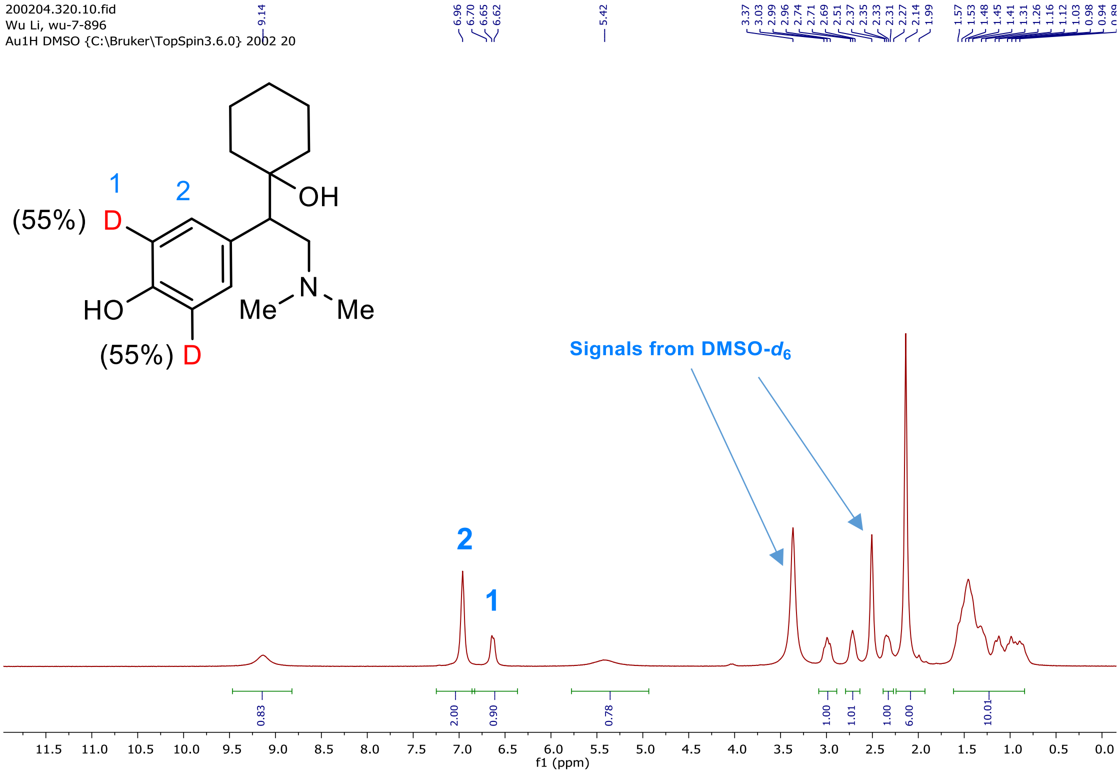

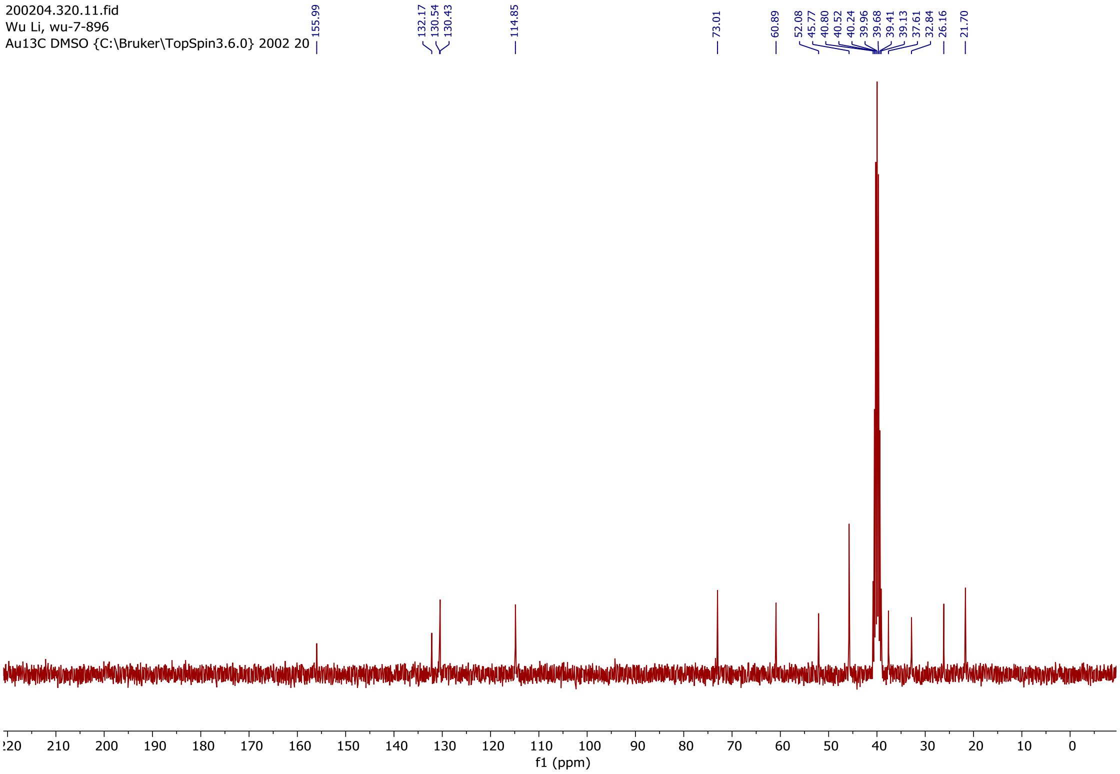

¹H NMR for 90a:

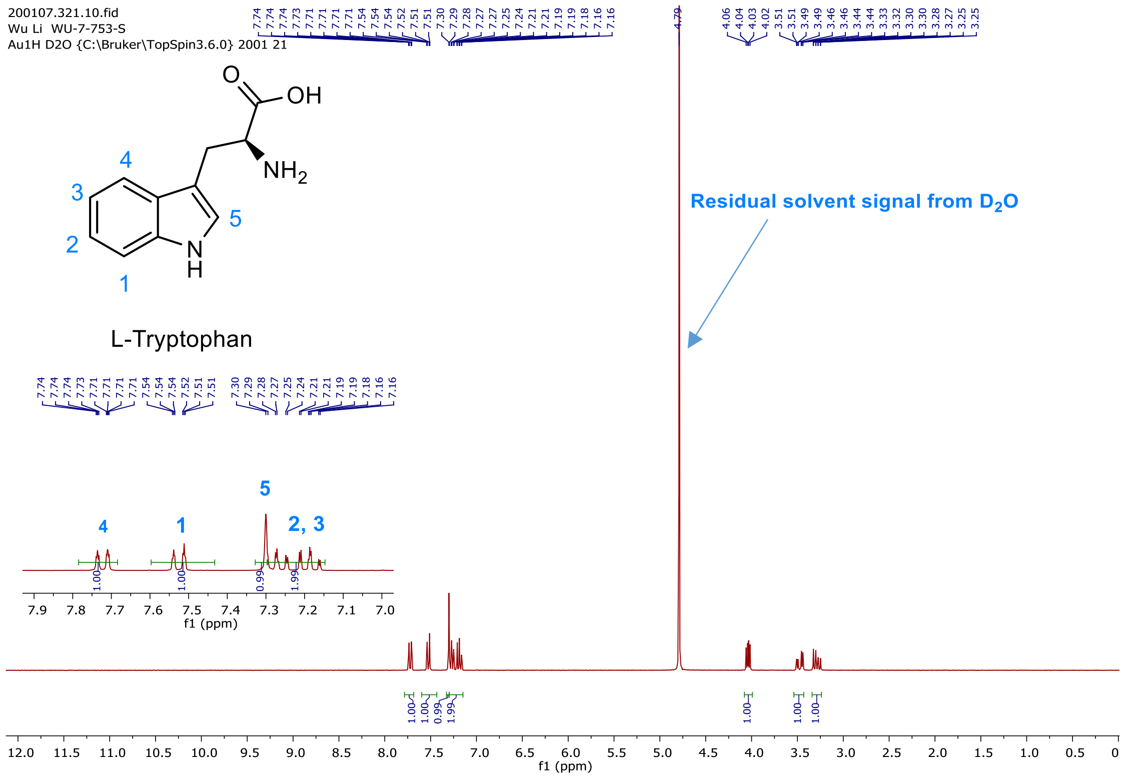

Original spectra for 90b:

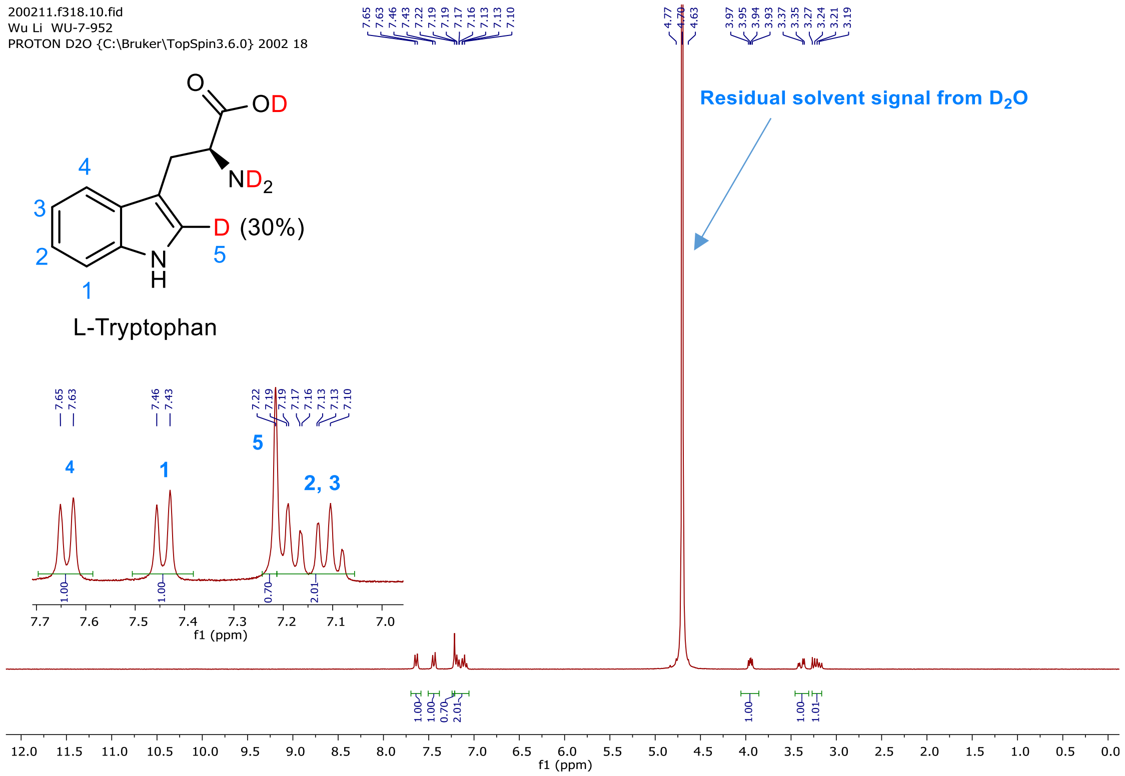

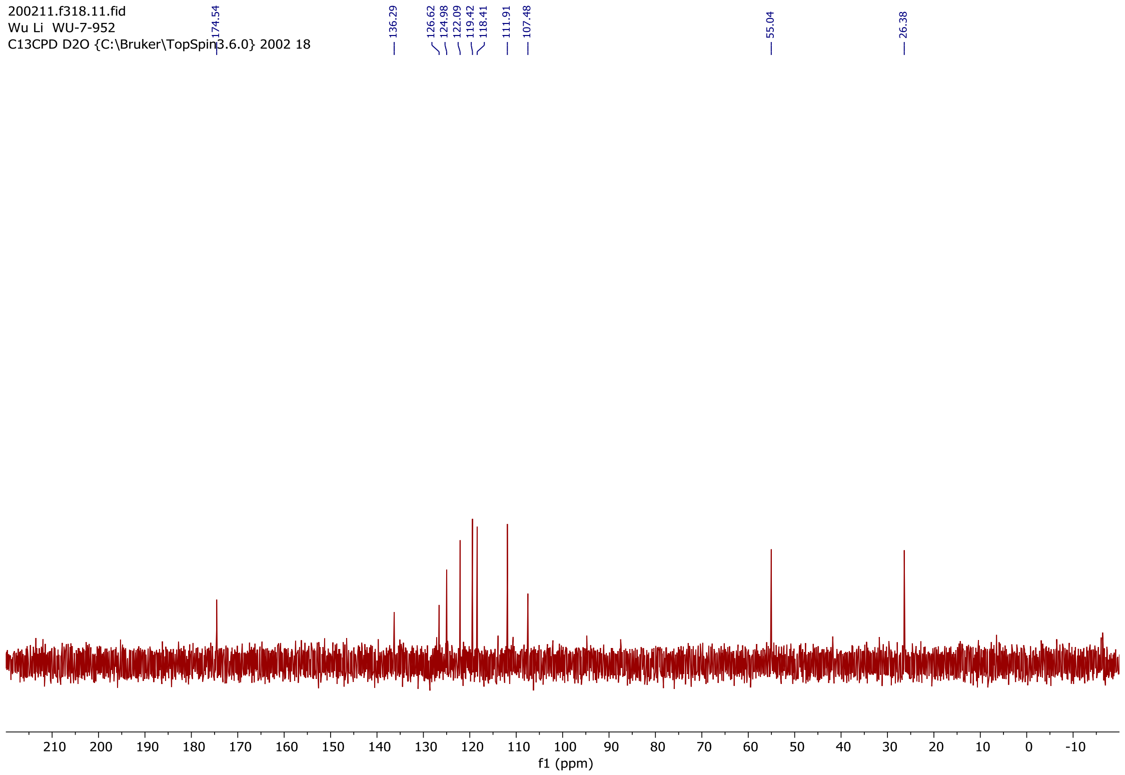

Original spectra for 91b:

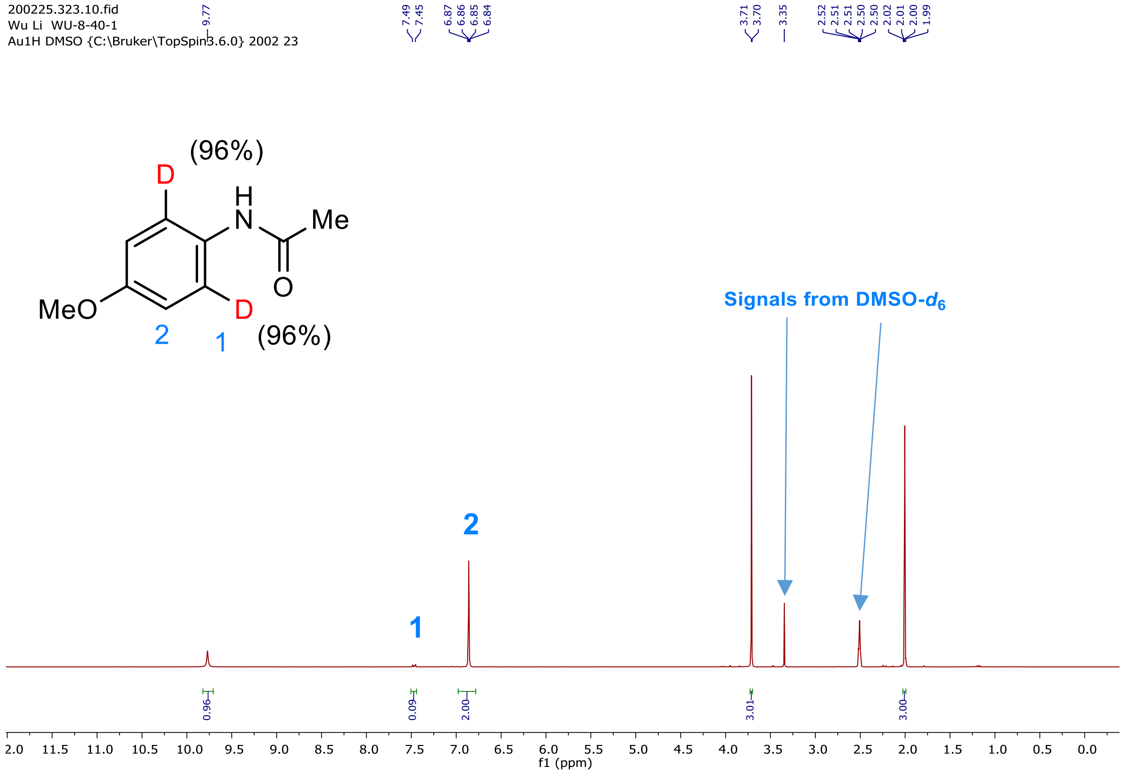

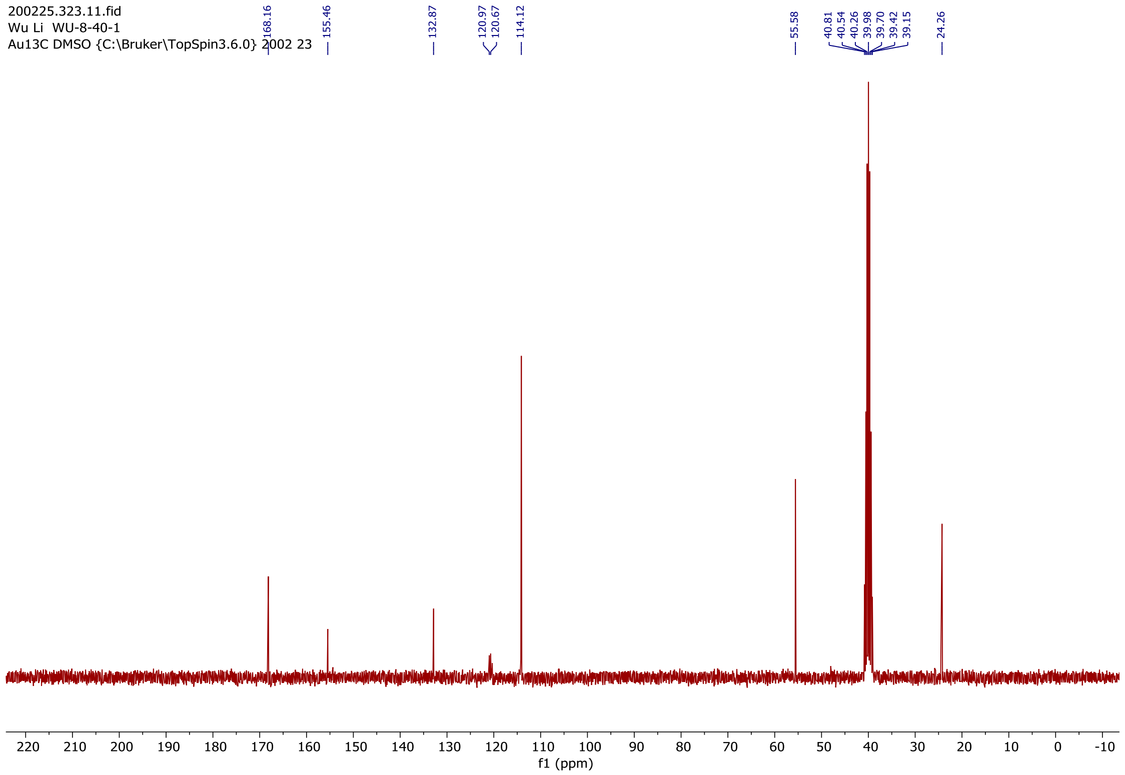

Original spectra for 92b:

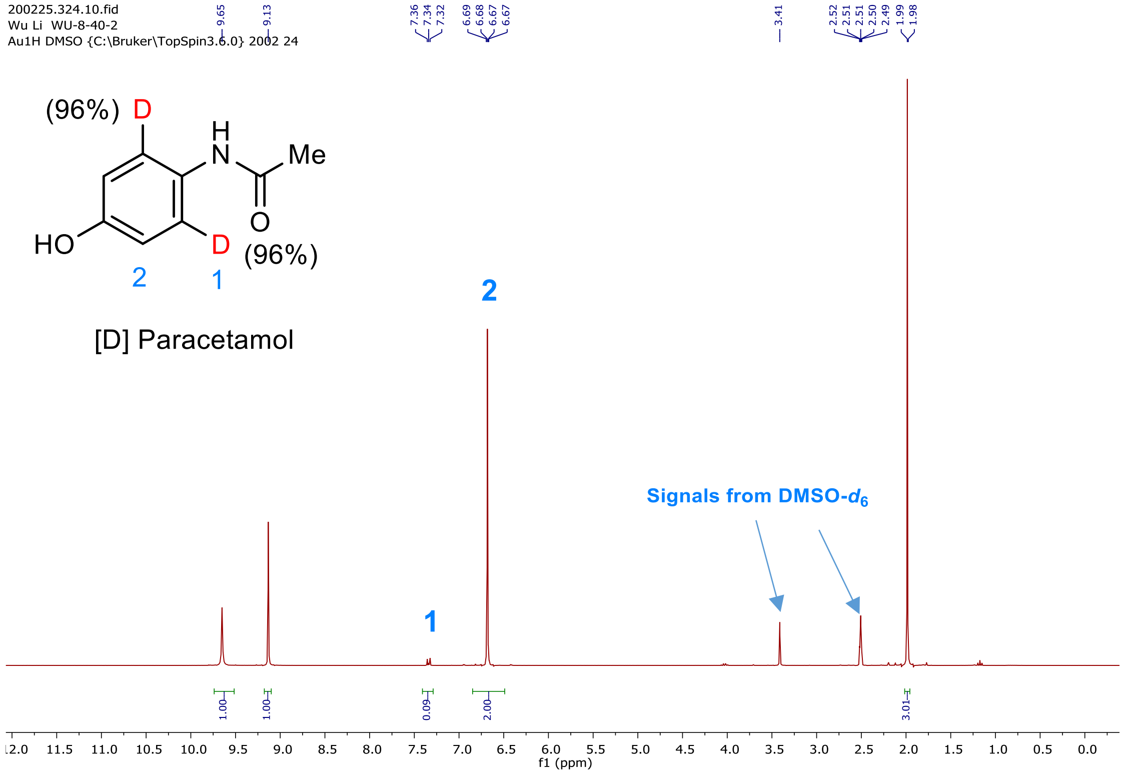

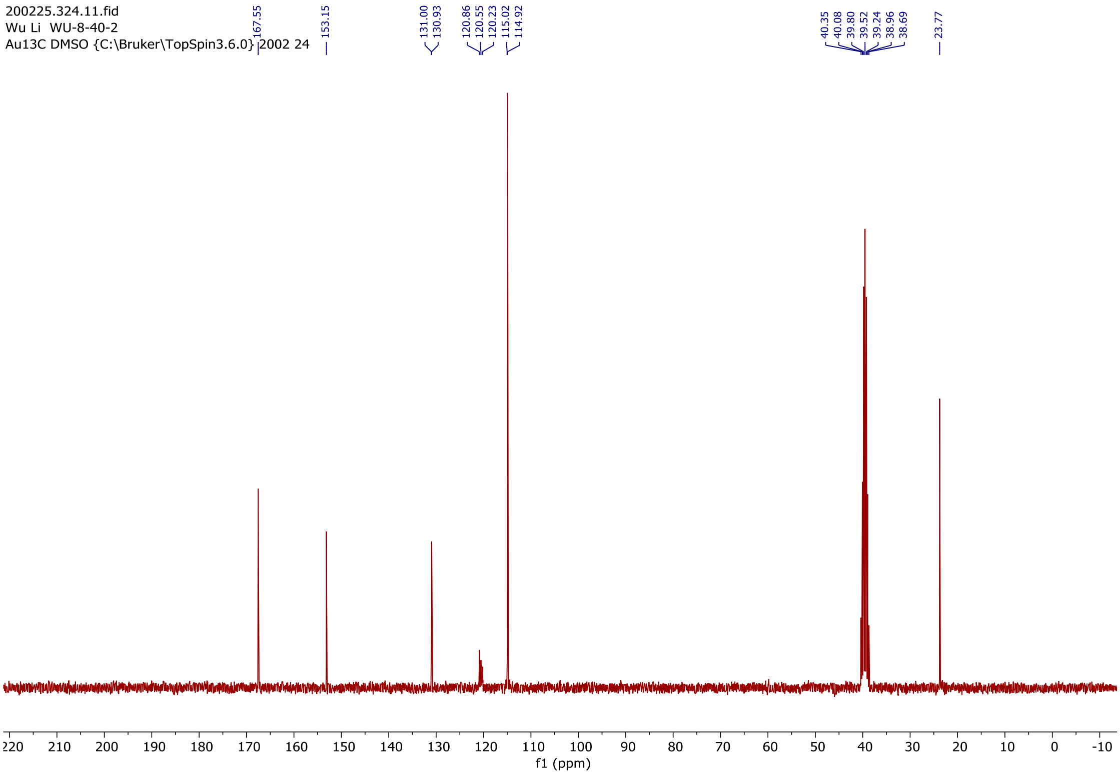

Original spectra for 93b:

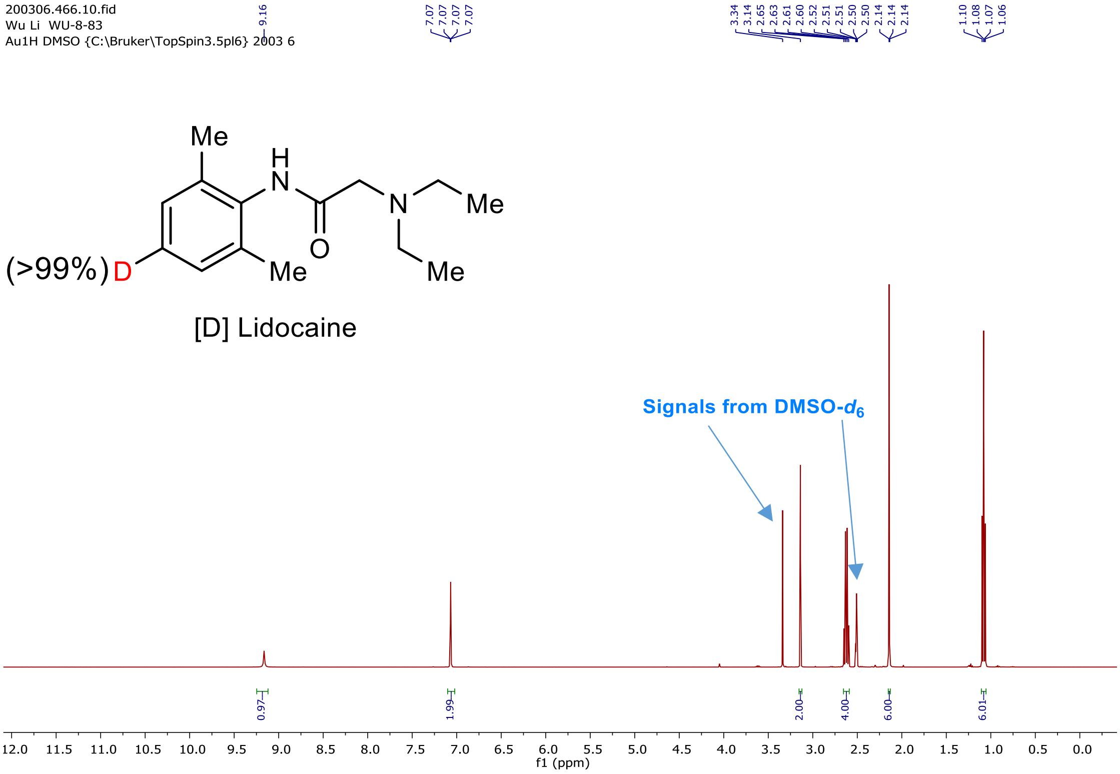

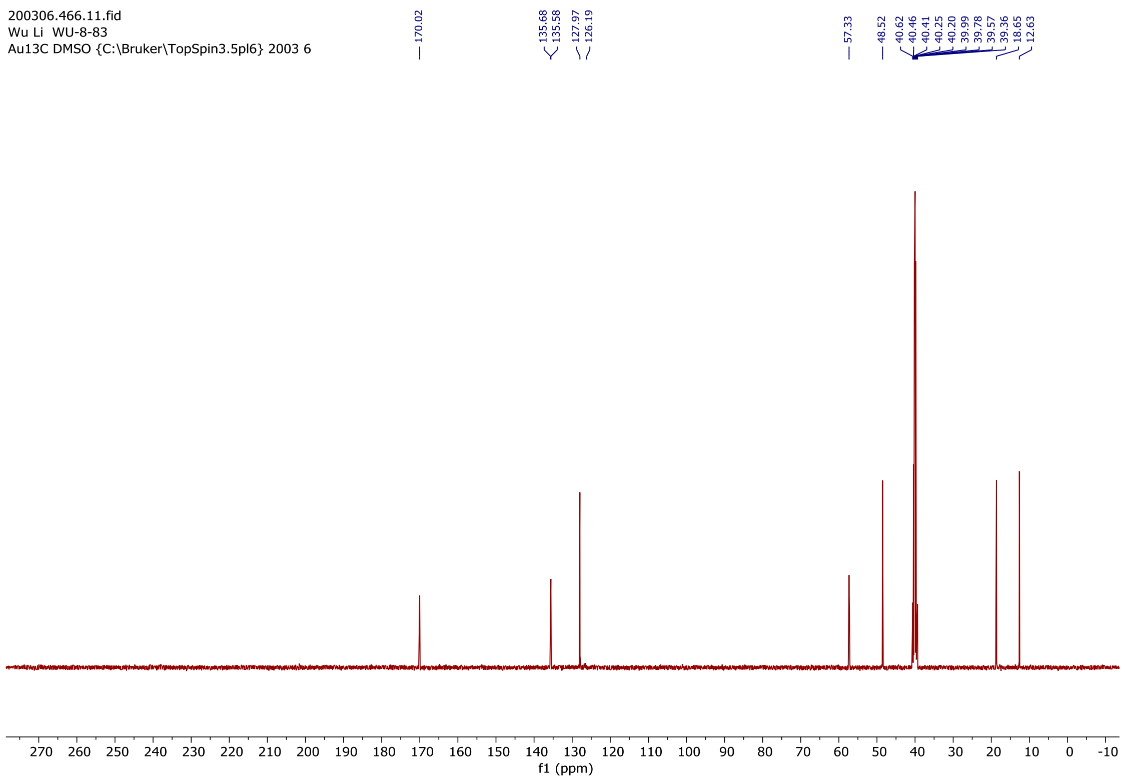

Original spectra for 94b:

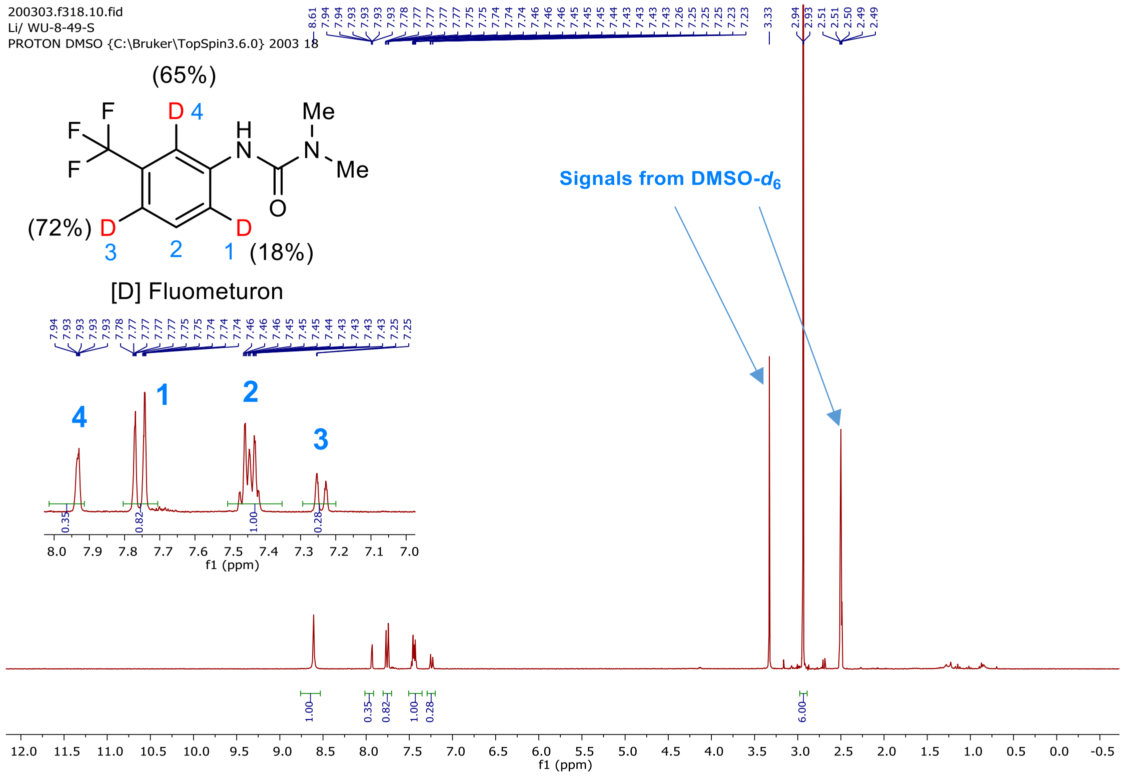

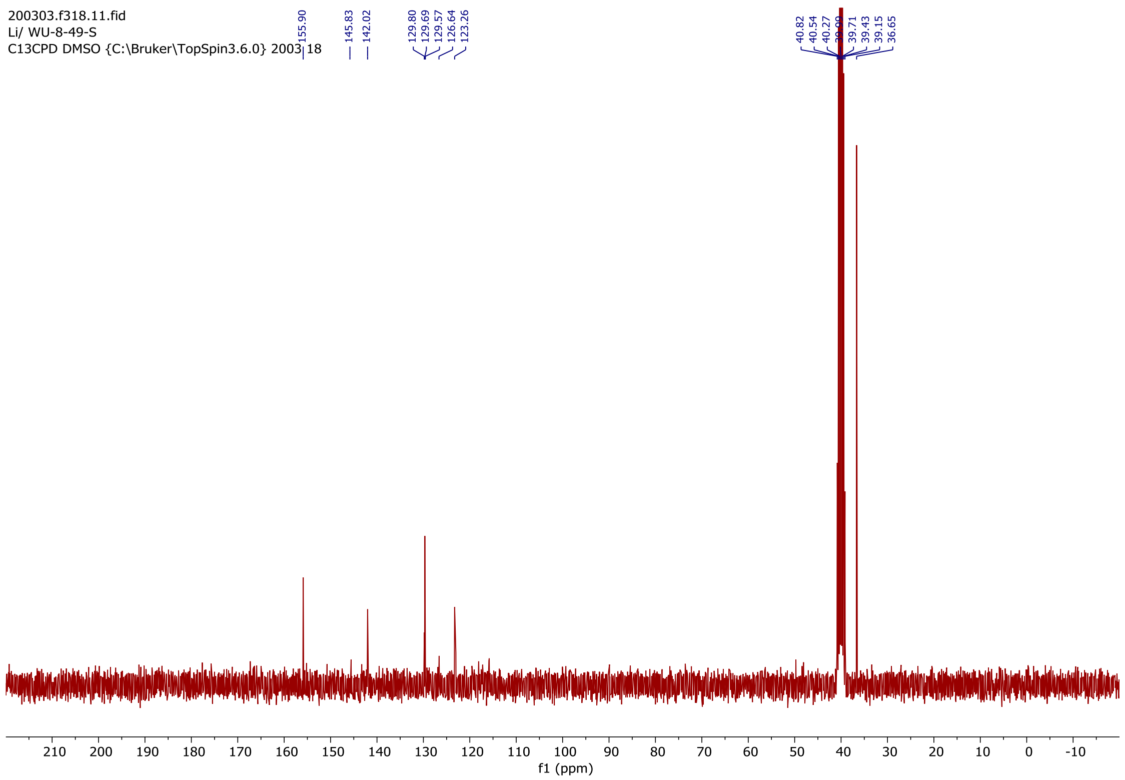
200303.f318.11.fid
Li/ WU-8-49-S
C13CPD DMSO {C:\Bruker\TopSpin3.6.0} 2003 18
155.90
145.83
142.02
129.80
129.69
129.57
126.64
123.26
40.82
40.54
40.27
39.71
39.43
39.15
36.65
f1 (ppm)

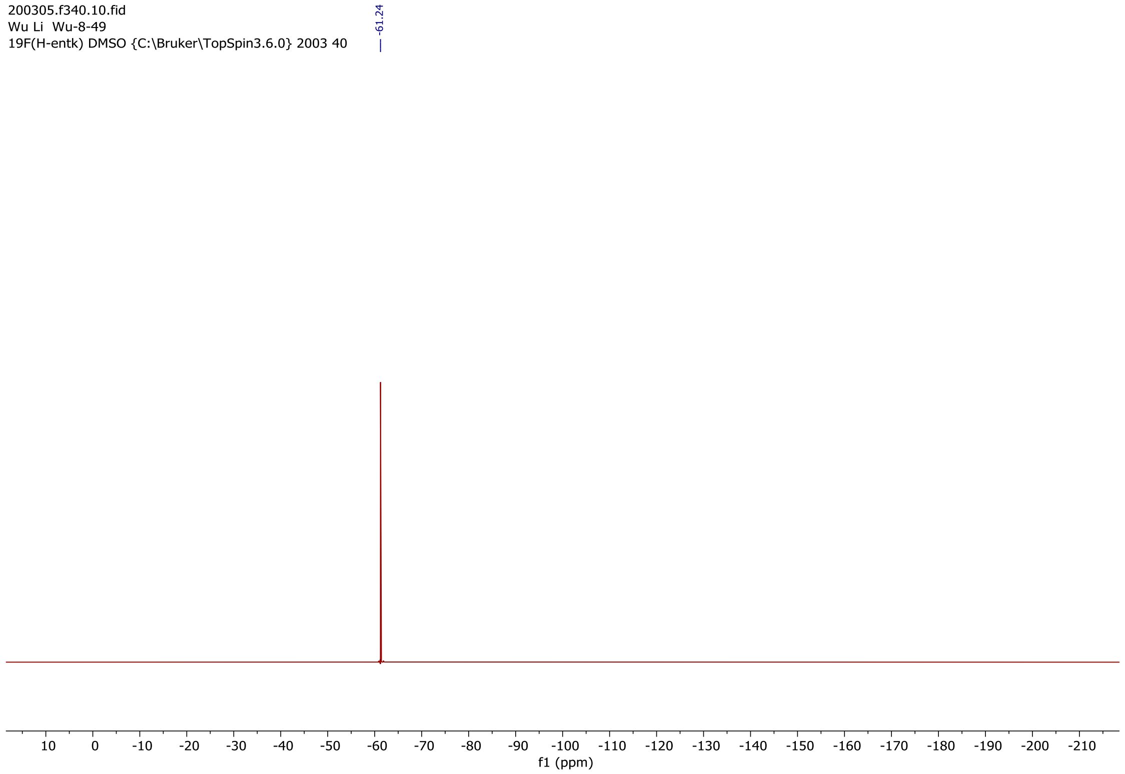
200305.f340.10.fid
Wu Li Wu-8-49
19F(H-entk) DMSO {C:\Bruker\TopSpin3.6.0} 2003 40
-61.24
f1 (ppm)

Original spectra for 95b:

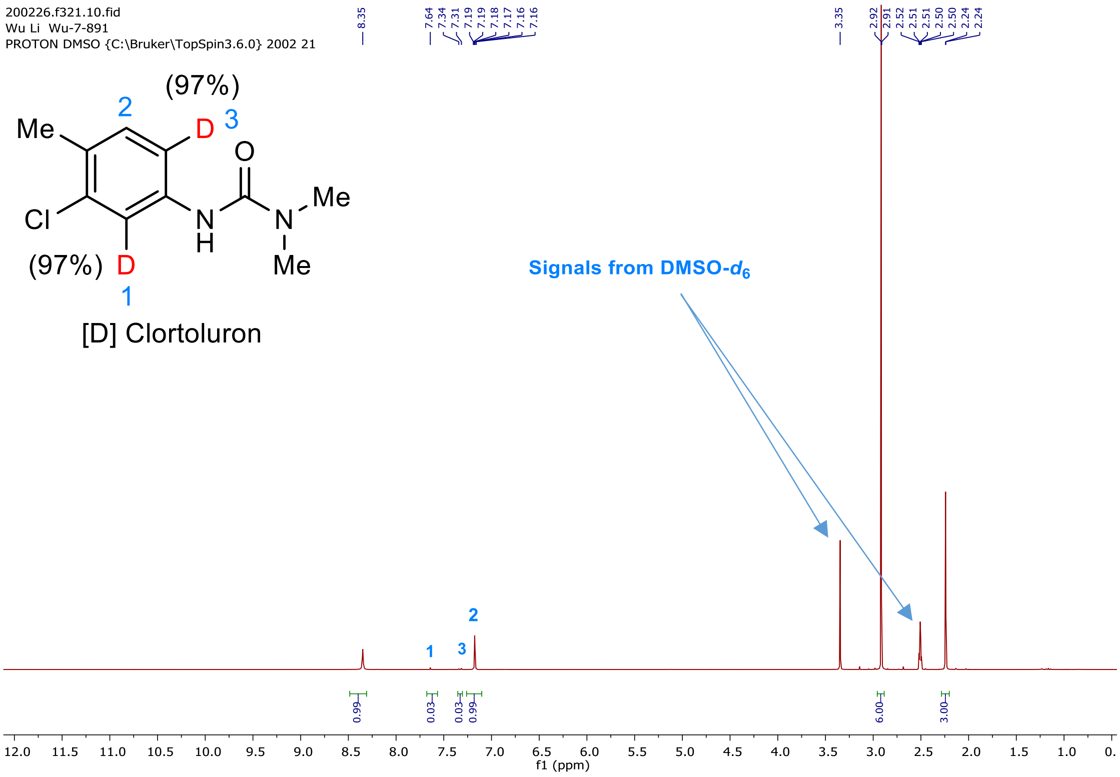

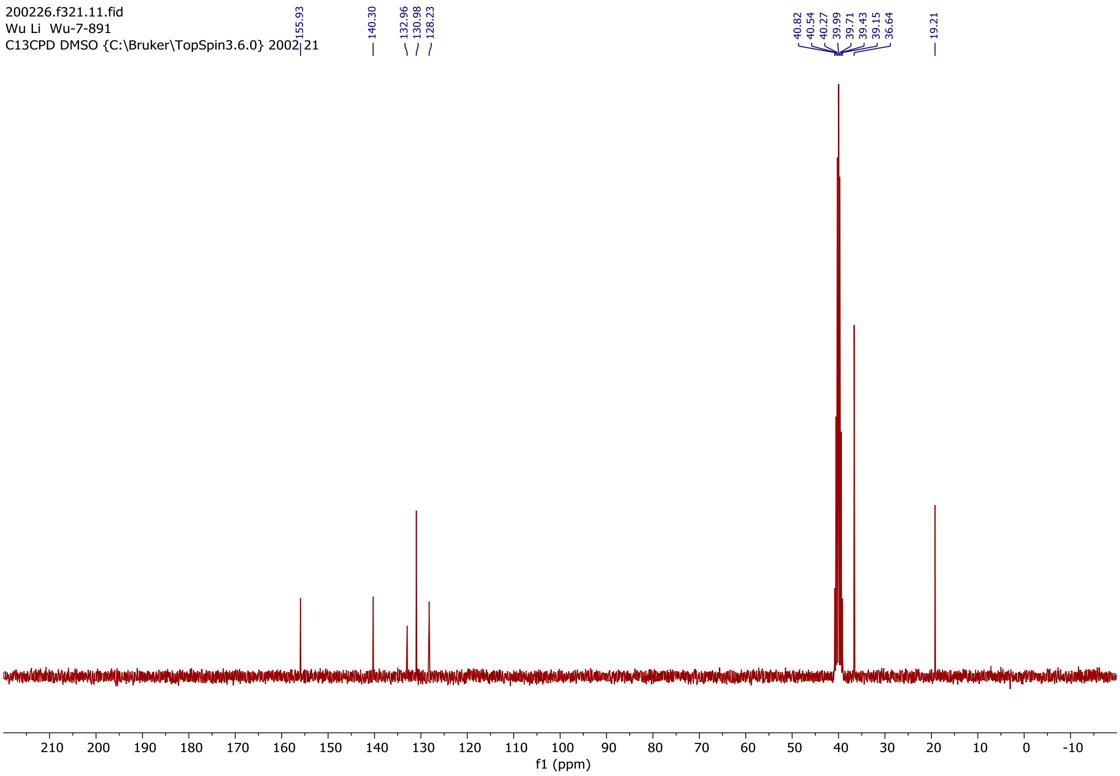

Original spectra for 96b:

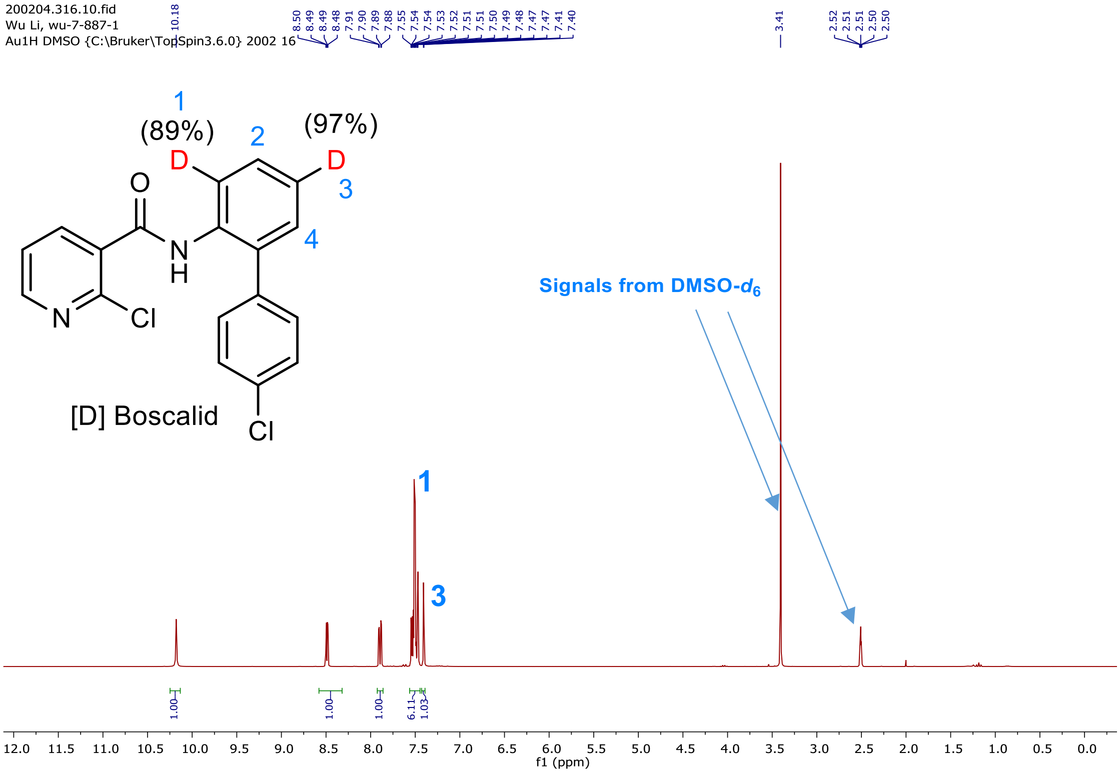

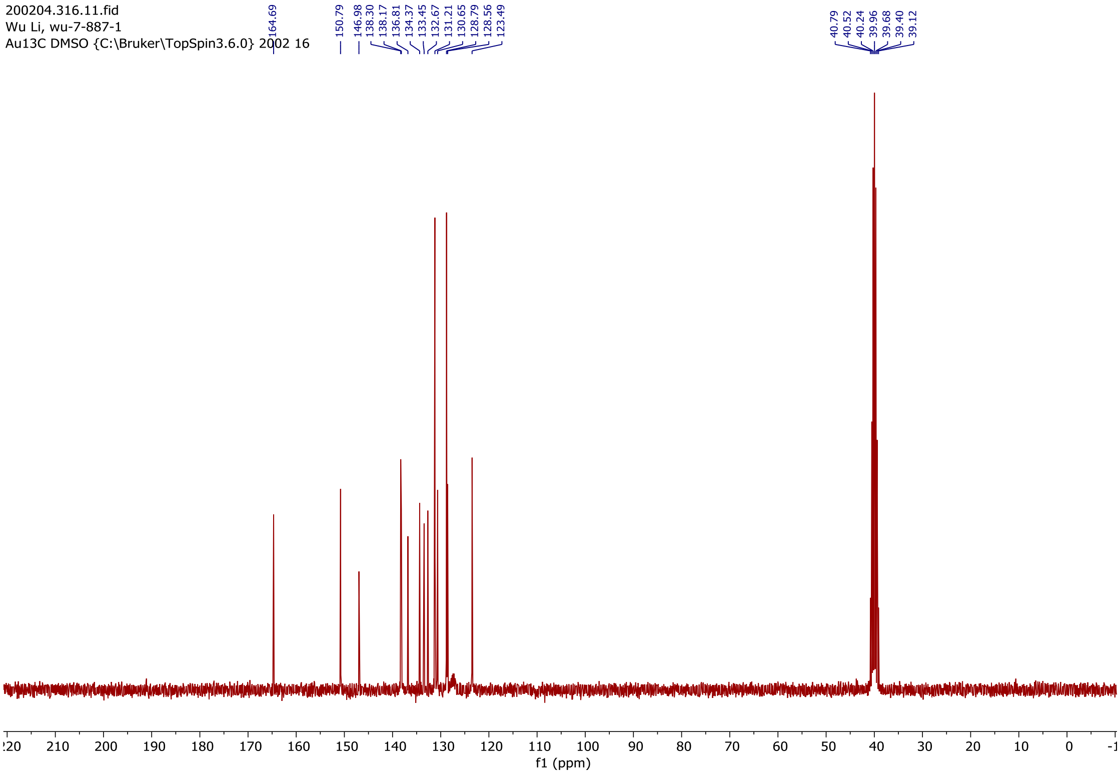

^{1}H NMR for the substrate of aniline (1a):

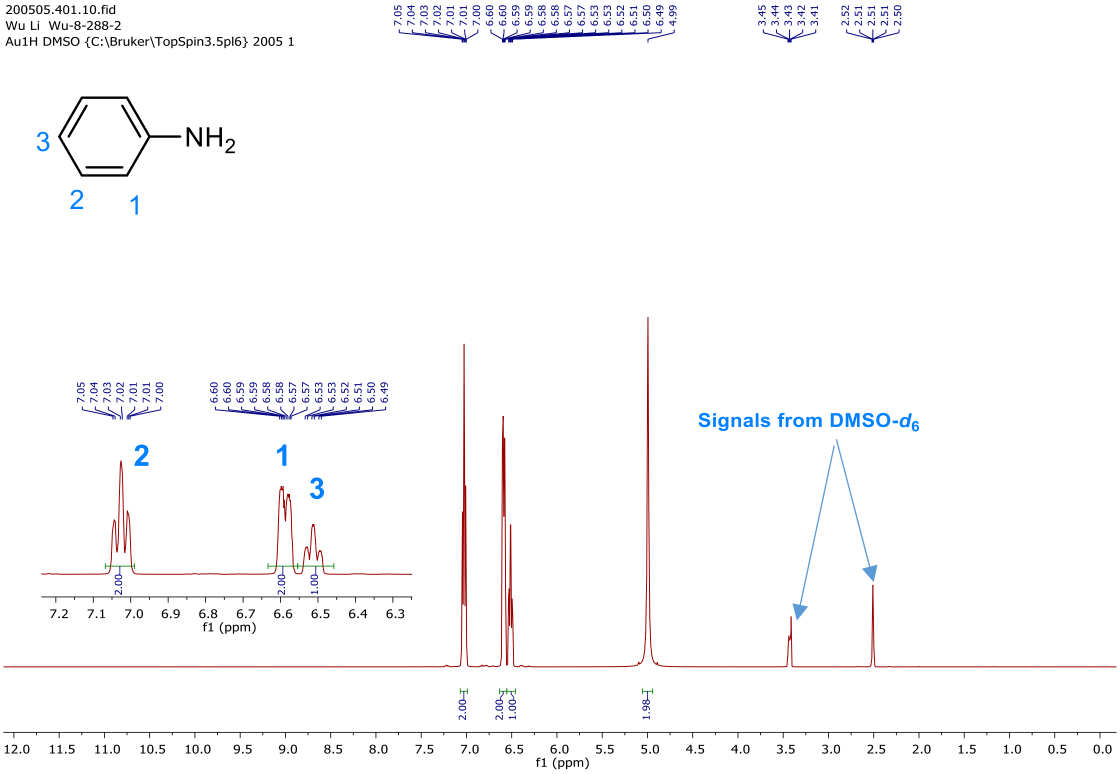

^{1}H NMR for 2b:

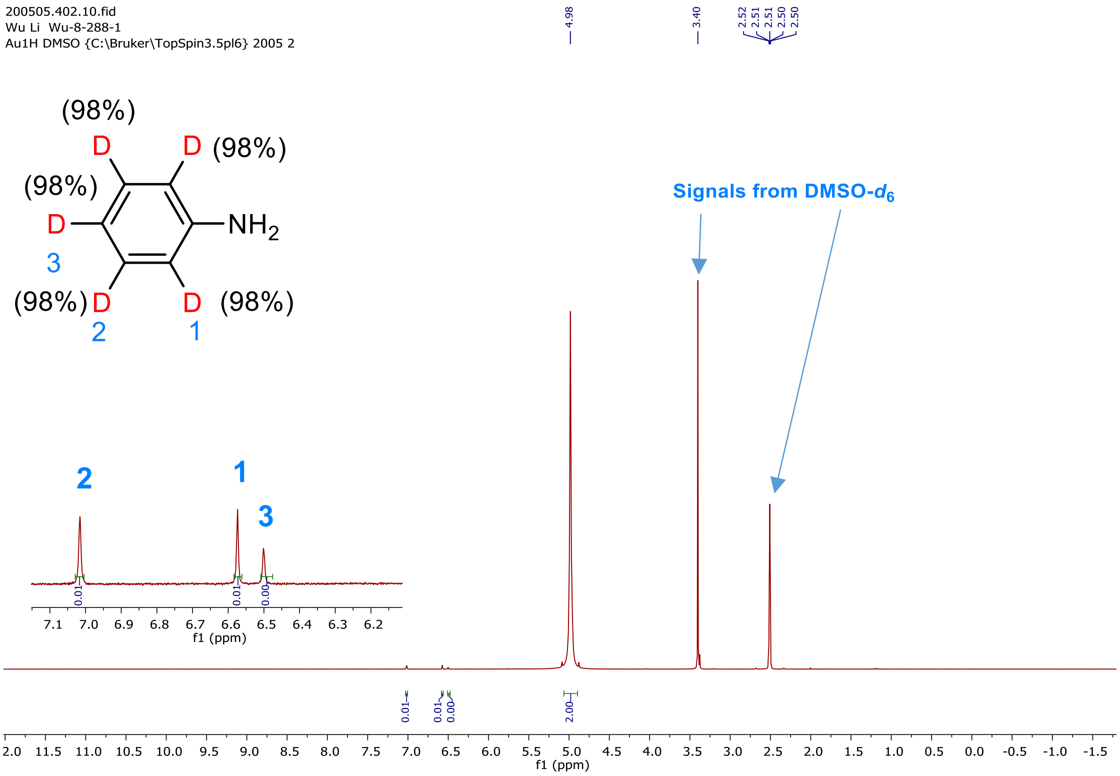

Original spectra for 2a:

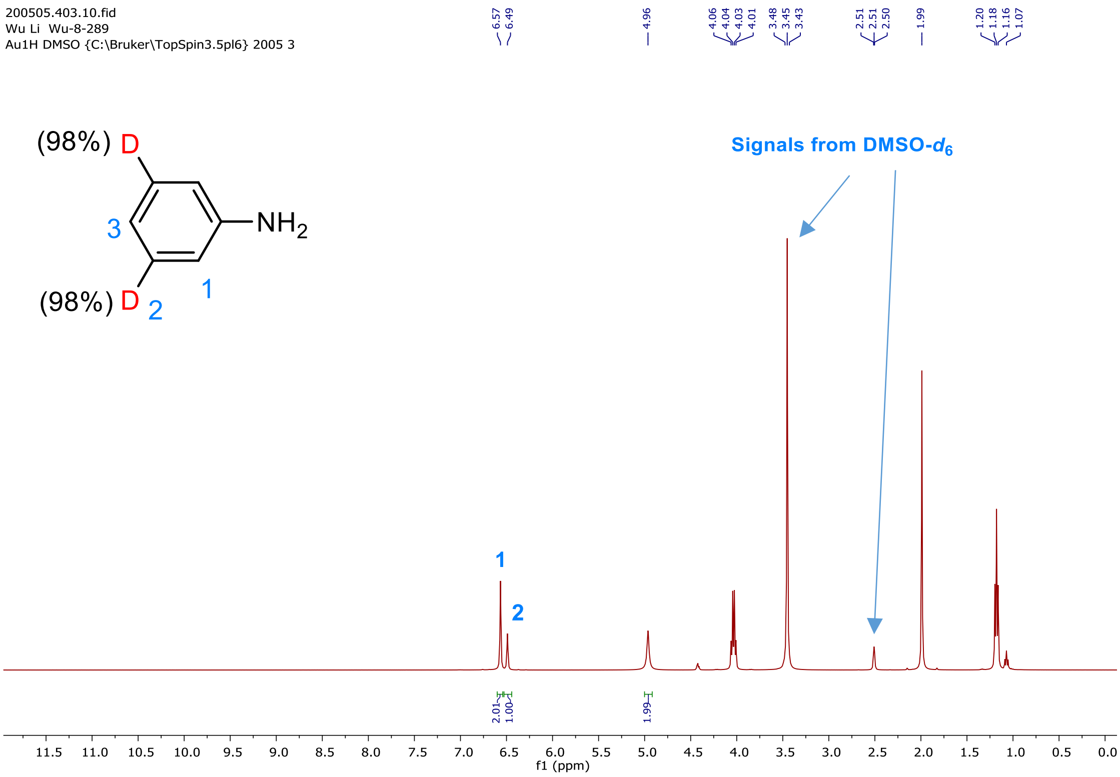

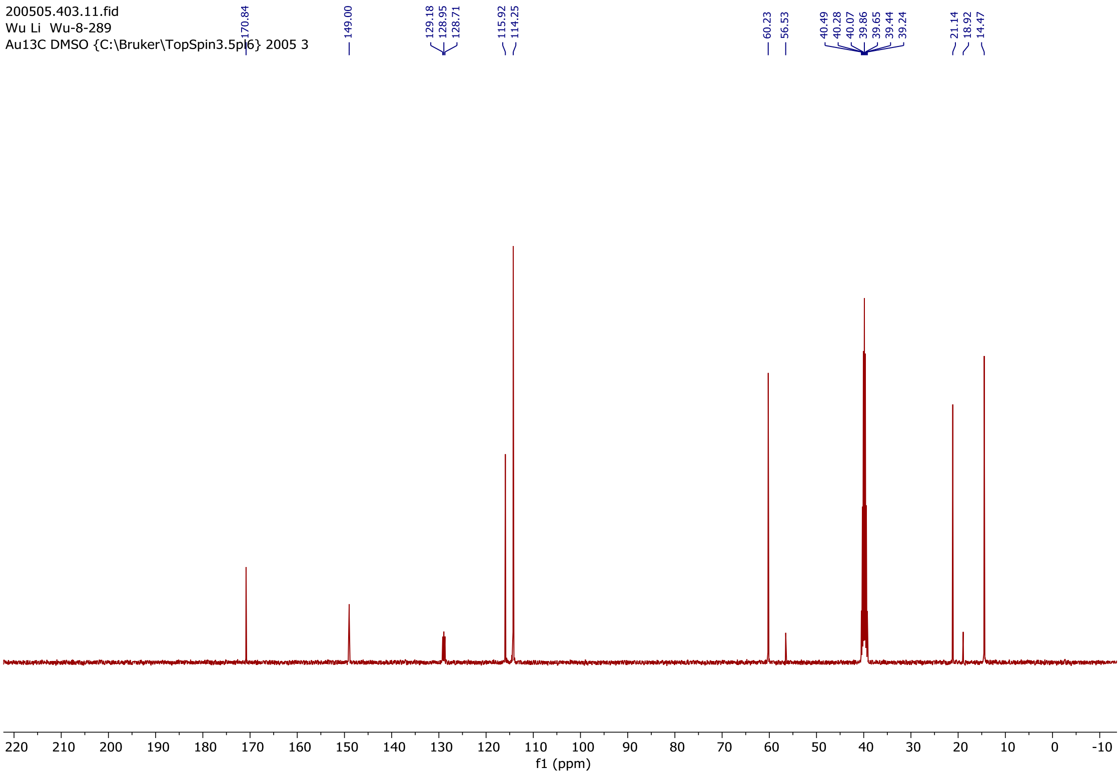

14. References

1 Sajiki, H. *et al.* Efficient and Selective Deuteration of Phenylalanine Derivatives Catalyzed by Pd/C. *Synlett*, 0845-0847, (2005).

2 Jagadeesh, R. V. *et al.* Nanoscale Fe_2O_3-based catalysts for selective hydrogenation of nitroarenes to anilines. *Science* **342**, 1073-1076, (2013).

3 Cui, X. *et al.* Synthesis and Characterization of Iron-Nitrogen-Doped Graphene/Core-Shell Catalysts: Efficient Oxidative Dehydrogenation of N-Heterocycles. *J. Am. Chem. Soc.* **137**, 10652-10658, (2015).

4 Formenti, D., Ferretti, F., Scharnagl, F. K. & Beller, M. Reduction of Nitro Compounds Using 3d-Non-Noble Metal Catalysts. *Chem. Rev.* **119**, 2611-2680, (2019).

5 Torres Galvis, H. M. *et al.* Supported iron nanoparticles as catalysts for sustainable production of lower olefins. *Science* **335**, 835-838, (2012).

6 Scofield, J. H. Hartree-Slater subshell photoionization cross-sections at 1254 and 1487 eV. *J. Electron Spectros. Relat. Phenomena.* **8**, 129-137, (1976).

7 Tomaszewski, P. E. Structural phase transitions in crystals. I. Database. *Phase Transitions* **38**, 127-220, (1992).

8 Mettler, M. S. *et al.* Revealing pyrolysis chemistry for biofuels production: Conversion of cellulose to furans and small oxygenates. *Energy Environ. Sci.* **5**, 5414-5424, (2012).

9 Campos, A. *et al.* An activity and XANES study of Mn-promoted, Fe-based Fischer–Tropsch catalysts. *Appl. Catal. A* **375**, 12-16, (2010).

10 Fruchart, D., Chaudouet, P., Fruchart, R., Rouault, A. & Senateur, J. P. Etudes structurales de compose´s de type ce´mentite: Effet de l'hydroge`ne sur Fe_3C suivi par diffraction neutronique. Spectrome´trie Mo¨ssbauer sur $FeCo_2B$ et Co_3B dope´s au ^{57}Fe. *J. Solid State Chem.* **51**, 246-252, (1984).

11 Stoll, S. & Schweiger, A. EasySpin, a comprehensive software package for spectral simulation and analysis in EPR. *J. Magn. Reson.* **178**, 42-55, (2006).

12 Dai, X. *et al.* Sustainable Co-Synthesis of Glycolic Acid, Formamides and Formates from 1,3-Dihydroxyacetone by a Cu/Al_2O_3 Catalyst with a Single Active Sites. *Angew. Chem. Int. Ed.* **58**, 5251-5255, (2019).

13 Vodnala, N. *et al.* Copper-Catalyzed Site-Selective Oxidative C−C Bond Cleavage of Simple Ketones for the Synthesis of Anilides and Paracetamol. *Adv Synth Catal* **361**, 135-145, (2019).

14 Reilly, T. J. The Preparation of Lidocaine. *J. Chem. Educ.* **76**, (1999).
